# Sulfamides direct radical-mediated chlorination of aliphatic C–H bonds†

**DOI:** 10.1039/c9sc03428e

**Published:** 2019-11-08

**Authors:** Melanie A. Short, Mina F. Shehata, Matthew A. Sanders, Jennifer L. Roizen

**Affiliations:** Duke University, Department of Chemistry Box 90346 Durham North Carolina 27709-0354 USA j.roizen@duke.edu

## Abstract

Given the prevalence of aliphatic amines in bioactive small molecules, amine derivatives are opportune as directing groups. Herein, sulfamides serve as amine surrogates to guide intermolecular chlorine-transfer at γ-C(sp^3^) centers. This unusual position-selectivity arises because accessed sulfamidyl radical intermediates engage preferentially in otherwise rare 1,6-hydrogen-atom transfer (HAT) processes through seven-membered transition states. The site-selectivity of C–H abstraction can be modulated by adjusting the steric and electronic properties of the sulfamide nitrogen substituents, an ability that has not been demonstrated with other substrate classes. The disclosed reaction relies on a light-initiated radical chain-propagation mechanism to oxidize C(sp^3^)–H bonds efficiently.

## Abstract 2

Amine-anchored sulfamides direct radical-mediated chlorination of aliphatic C–H bonds. The site of C–H abstraction can be modulated by varying the sulfamide nitrogen substituents, a feature that has not been demonstrated with other substrate classes.

## Graphical abstract

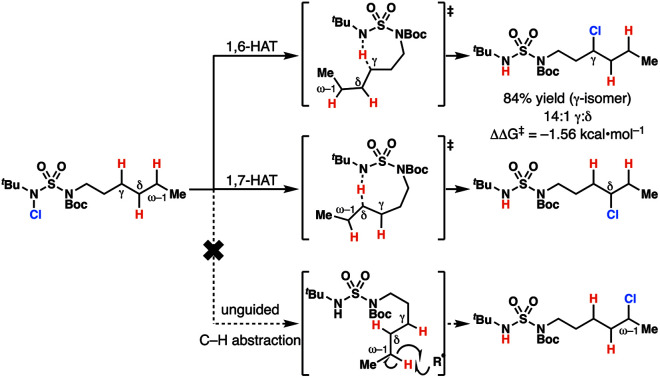

## Introduction

**Scheme 1:**
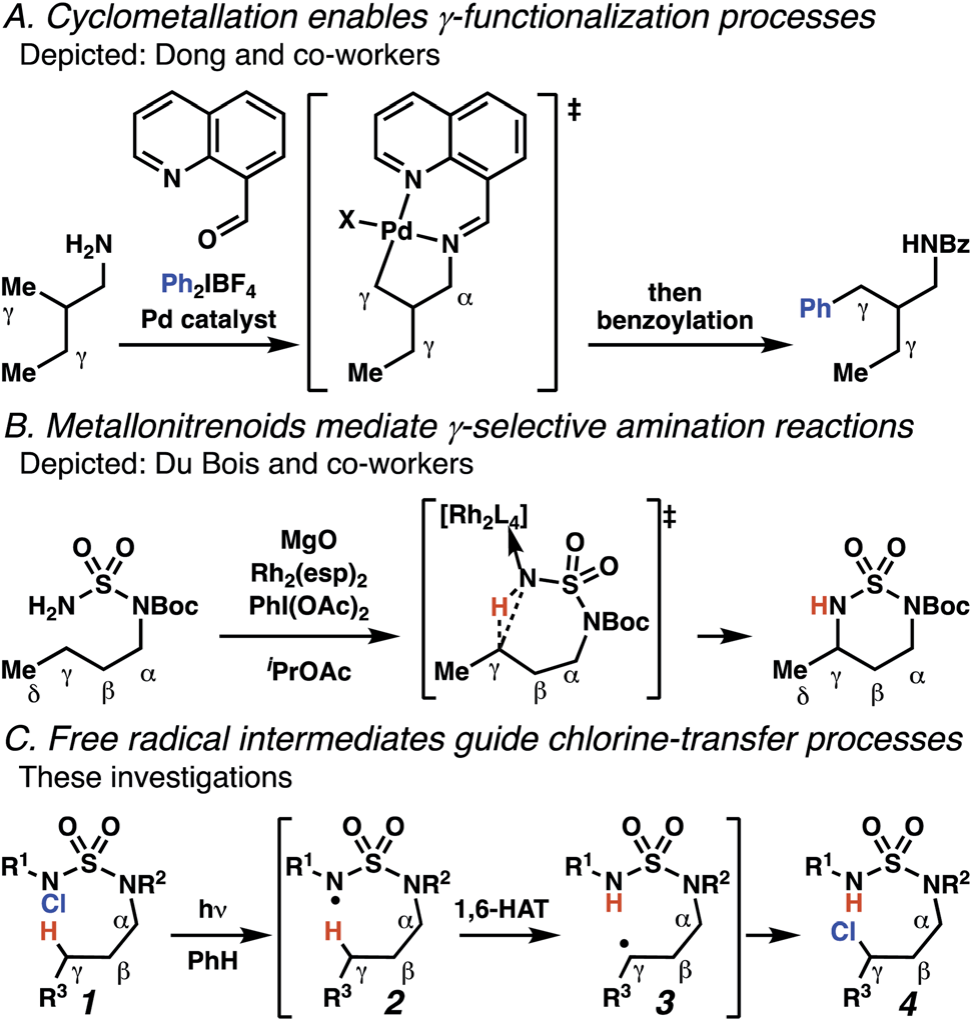
γ-C(sp^3^)–H reactions of amine derivatives.

Aliphatic amines are important structural motifs within organic molecules, making alkyl amine derivatives readily available. These derivatives can be used to guide position-selective C–H functionalization reactions^1^ to α-,^2^ β-, γ-, and δ-positions.^3^ Nevertheless, few strategies result in γ-C(sp^3^)–H functionalization. Amine derivatives template γ-selective cyclometallation processes^4–6^ (Scheme 1A), and can stabilize metallonitrenoid or metalloradical intermediates to facilitate C–H amination reactions (Scheme 1B).^7–9^ As a mechanistic complement to these approaches, herein disclosed is the first reaction in which an amine surrogate guides γ-C(sp^3^)–H functionalization by way of free radical intermediates,^10^ enabling a sulfamide-guided chlorine-transfer process (Scheme 1C).

In these reactions, intermediate sulfamidyl radicals **2** engage in otherwise rare 1,6-HAT processes. With the exception of the recent discovery of sulfamate ester-templated reactions,^11–13^ transformations that employ cleavable linkers in 1,6-HAT processes lack generality. Fortunately, sulfamides appear to direct reactions based on 1,6-HAT processes,^10^ consistent with the geometrically originated prediction that elongated S–N bonds (∼1.58–1.69 Å)^14^ kinetically favor a seven-membered transition state^15^ for C–H abstraction.

This concept is developed to enable position-selective chlorine-transfer reactions (Scheme 1C). Alkyl chlorides are durable, yet versatile synthetic intermediates,^16^ and can be found in bioactive small molecules.^17^ Yet, directed C(sp^3^)–H chlorination reactions^18^ can be plagued by competitive off-site chlorine-installation arising from unguided C–H abstraction. The developed sulfamide-directed reactions offer high levels of position-selectivity with the unusual ability to predictably modulate site-selectivity based on variations in the steric and electronic properties of the substituents on the sulfamide nitrogen atoms. With appropriate substituents, the site of chlorine-transfer is complementary to that available based on other techniques,^19^ including sulfamate ester-guided processes,^11^ traditional Hoffman–Löffler–Freytag protocols,^20^ templated methods,^21^ and unguided processes that rely on innate selectivity.^1*a*,*b*,22,23^

## Results and discussion

Sulfamide substrates present two chemically distinct nitrogen atoms that can support nitrogen-centered radicals as reaction intermediates. To simplify mechanistic investigations, we chose to access sulfamidyl radicals from *N*-chlorosulfamides **1** and **5***via* light-initiated nitrogen–chlorine bond homolysis. The requisite sulfamides are prepared from alcohols through Mitsunobu reactions,^24^ or from amines using a recently disclosed sulfamoylation strategy.^25^ The generated sulfamides react with an electrophilic chlorinating reagent to provide structurally diverse *N*-chlorosulfamides.

**Scheme 2:**
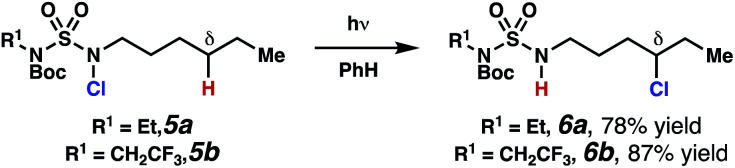
Chlorine-transfer from “internal” sulfamide nitrogen occurs through a 1,5-HAT process to provide δ-chlorinated alkanes.

As anticipated, *N*-chlorosulfamides prepared such that the chlorine atom initially resides on the “internal” sulfamide nitrogen (*i.e.*, **5**), engage in selective 1,5-HAT processes upon photoirradiation. These substrates provide δ-chlorinated alkanes **6** in good yield with exquisite selectivity (Scheme 2). As this selectivity mimics that observed in related HLF-type processes, our investigations primarily focus on reactions that target transformation of γ-C(sp^3^)–H bonds of amine derivatives, as technologies for γ-C(sp^3^)–H functionalization are limited (Scheme 1).

Building upon our laboratory's interest in transformations governed by 1,6-HAT processes,^11,13*a*,*d*^ we sought to exploit *N*-chlorosulfamides in reactions to access γ-chlorinated alkanes as a complement to more traditional chlorination methods. To our delight, when employing *N*-chlorosulfamides where the radical is generated on the “external” sulfamide nitrogen (*i.e.*, **1**), chlorine-transfer proceeds in synthetically useful yields at primary, secondary, and tertiary C–H bonds (Table 1). These C–H bonds have bond dissociation energies (BDEs) that cover a broad range (BDE ≈ 96–101 kcal mol^−1^),^26^ demonstrating the generality of the transformation. In particular, this chlorine-transfer reaction provides access to primary alkyl chlorides (**4a**) in excellent yield, outperforming related sulfamate ester-^11–13^ and sulfamide-guided^10^ methods in transforming strong primary C–H bonds. This sulfamide-guided process oxidizes C–H bonds at acyclic or cyclic centers (entries 4–6), and is compatible with pendant ester (entry 7) and masked amine (entry 8) functionalities. Moreover, naturally abundant amines, such as leucine-derivatives, are appropriate substrate precursors (*e.g.***4f**).

**Table 1:** Sulfamides guide γ-selective chlorine-transfer reactions

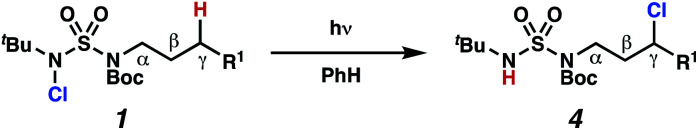			
Entry^a^	Product		Yield^b^
1	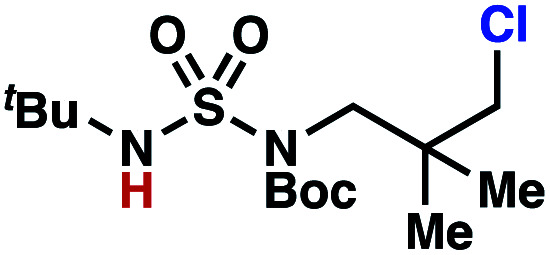	**4a**	94
2	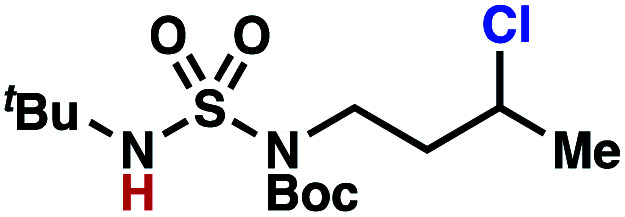	**4b**	93
3	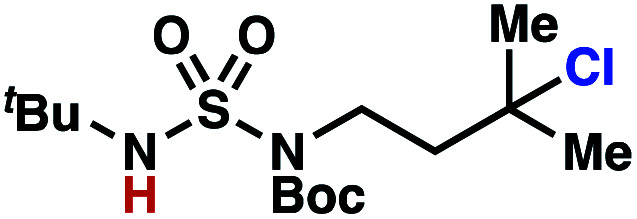	**4c**	98
4	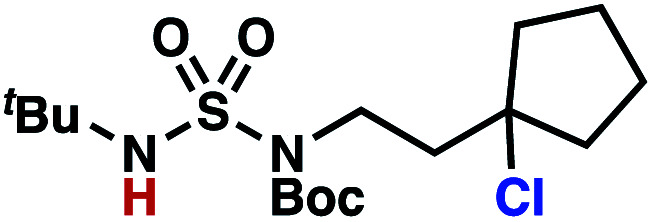	**4d**	94
5			94^c^
6	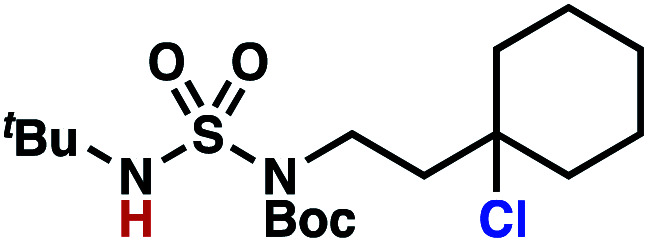	**4e**	95
7	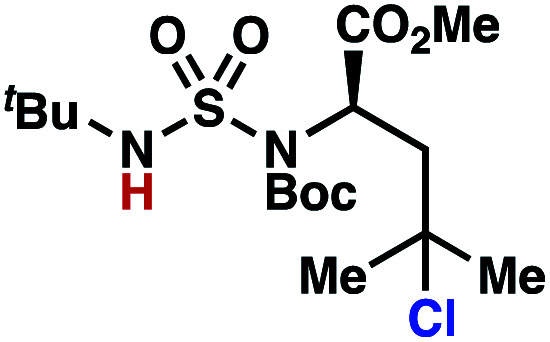	**4f**	81
8	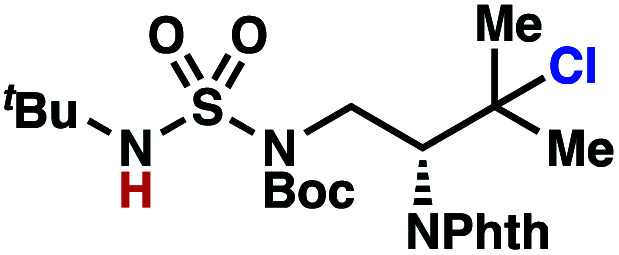	**4g**	85
9	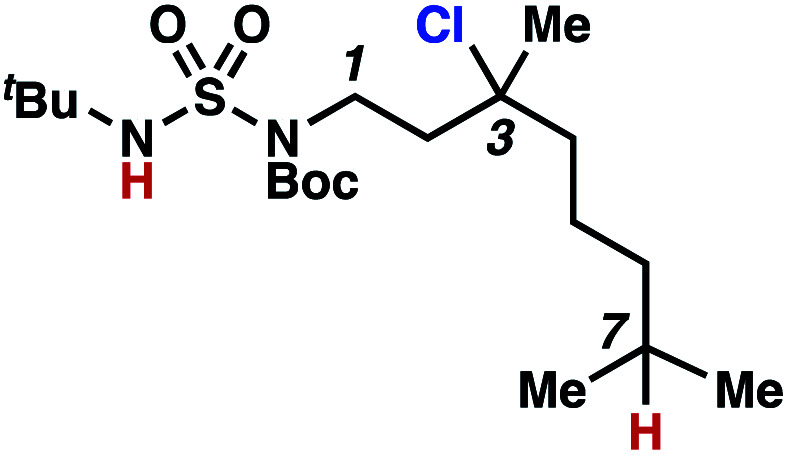	**4h**	92
10			95^c^

a Conditions A: 1.0 equiv. *N*-chlorosulfamide **1**, PhH (0.04 M), UV light.

b Isolated yield.

c 1.0 equiv. *N*-chlorosulfamide **1**, ^*i*^PrOAc (0.1 M), two 26 W CFL bulbs (1600 lumens).

This guided chlorine-transfer process overcomes site-selectivity arising from inductive deactivation. Electron-withdrawing groups, such as sulfamides, inductively deactivate proximate C–H bonds to abstraction by electrophilic radicals. Consequently, unguided C–H functionalization reactions engage more distal C–H bonds preferentially. This effect is particularly evident when employing unguided, radical-mediated reaction protocols with 3,7-dimethyloctyl derivatives where the C(7)–H bond serves as the predominant site of oxidation in azidation,^27^ amination,^28^ fluorination,^29^ trifluoromethylthiolation,^30^ and hydroxylation^31^ processes. By contrast, sulfamide **1h** undergoes templated chlorination at C(3)–H with exquisite site-selectivity (entries 9 and 10).

While the most consistently efficient protocol for chlorine-transfer relies on irradiation with UV light in benzene, some substrates react efficiently in ^*i*^PrOAc upon photolysis with compact fluorescent lights (CFLs, entries 5 and 10).

**Scheme 3:**
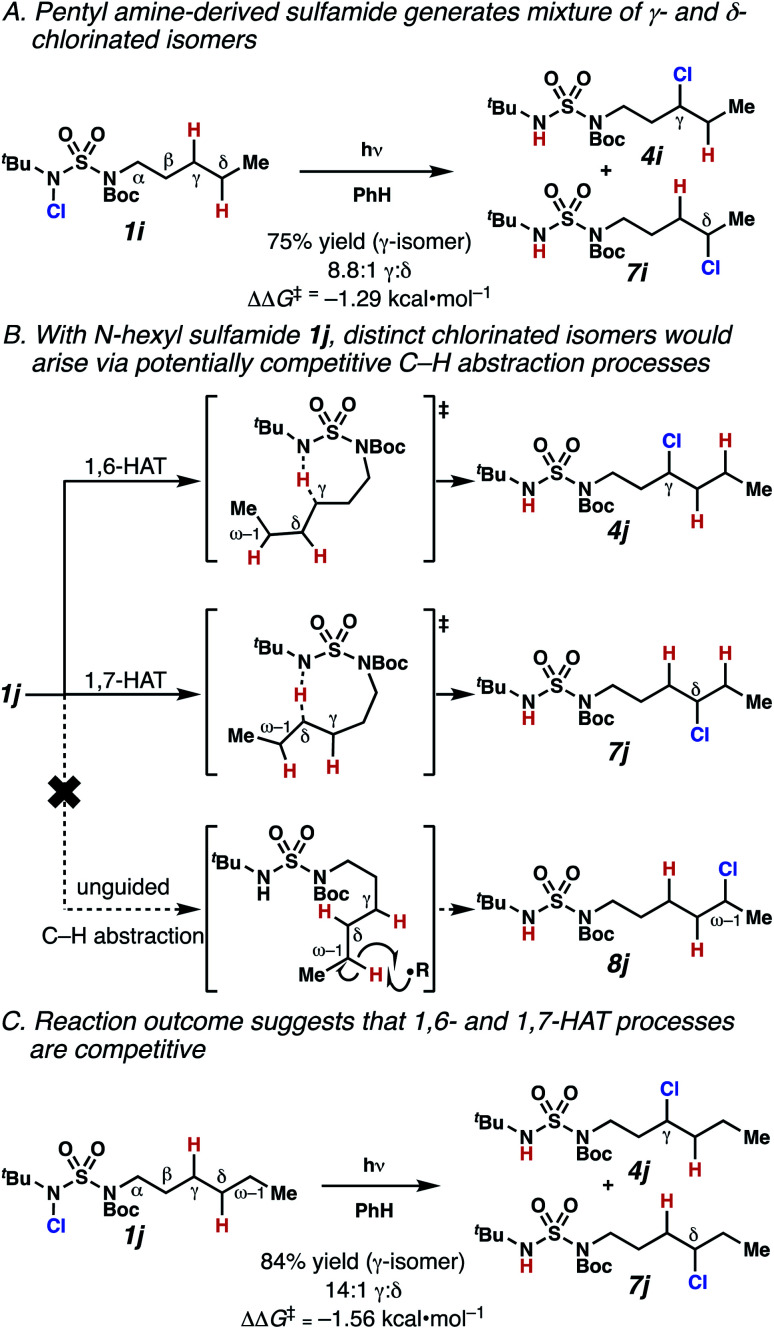
1,6- and 1,7-HAT processes appear competitive.

Surprisingly, sulfamide substrates undergo competitive γ- and δ-chlorination when they incorporate γ-C(sp^3^)–H bonds in proximity to weaker δ-C(sp^3^)–H bonds, a phenomenon not generally observed in related sulfamate ester-guided reactions.^11–13^ For example, *N*-pentyl sulfamide **1i** yields a crude 8.8 : 1 mixture of γ- and δ-chlorinated **4i** and **7i** from which γ-chlorinated **4i** can be isolated in 75% yield (Scheme 3A). In principle, δ-chlorinated minor product **7i** could form *via* either a substrate-guided 1,7-HAT process that relies on an eight-membered transition state, or an intermolecular C–H abstraction process.

To discriminate between these pathways, *N*-hexyl sulfamide **1j** was employed (Scheme 3B). With this substrate, a 1,7-HAT process would generate a δ-chlorinated product, whereas, a reaction reliant on innate selectivity would engage the most distal, secondary C–H bond to form (ω−1)-chlorinated **8j**. This reaction provides a crude 14 : 1 mixture of γ-chlorinated **4j** and δ-chlorinated **7j** (Scheme 3C). Fortuitously, (ω−1)-chlorinated **8j** is not detected, suggesting that δ-chlorinated **7j** may form through a guided 1,7-HAT process.

If a guided 1,7-HAT process provides the δ-chlorinated product, the ratio of γ-chlorinated **4** to δ-chlorinated **7** will quantitatively reflect the energetic difference between transition state barriers for competing 1,6- and 1,7-HAT processes (Scheme 3, Table 2). To relate the measured product ratios to the difference in the transition state barrier heights (Gibbs free energies), we employed the equation(ΔΔ*G*^‡^ = −*RT* ln(γ-chlorinated **4**/δ-chlorinated **7**)).

**Table 2:** Investigations into γ- *vs.* δ-selectivity

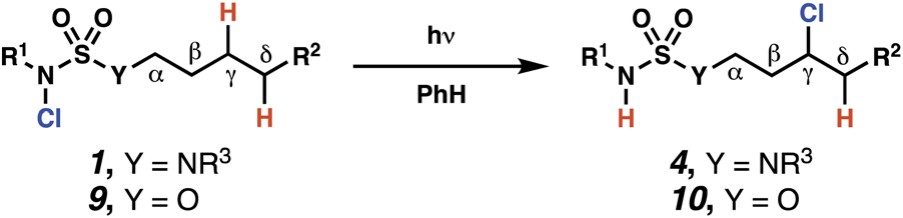					
Entry^a^	Product		γ : δ^b^	ΔΔ*G*^‡^^c^ (kcal mol^−1^)	Yield^d^
1	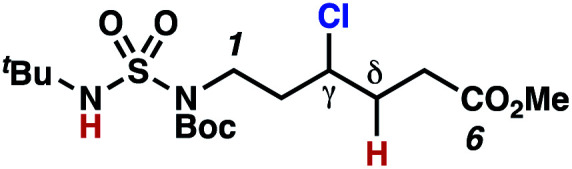	**4k**	—e	≤−1.77^f^	83
2	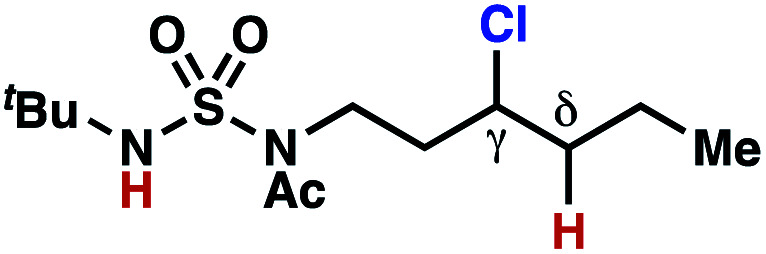	**4l**	>20 : 1	≤−1.77^f^	87
3	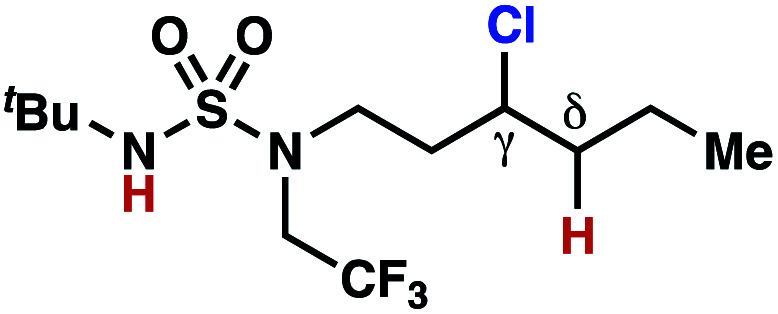	**4m**	—e	≤−1.77^f^	97
4	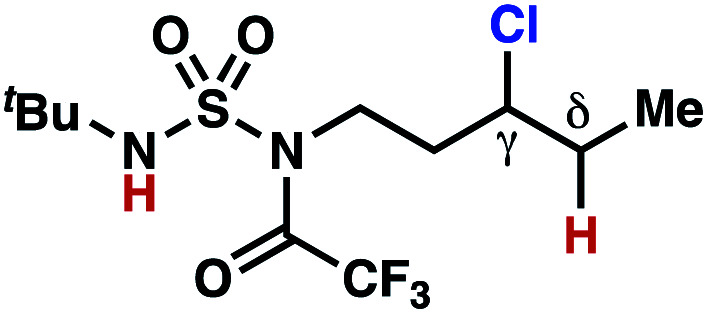	**4n**	—e	≤−1.77^f^	63
5	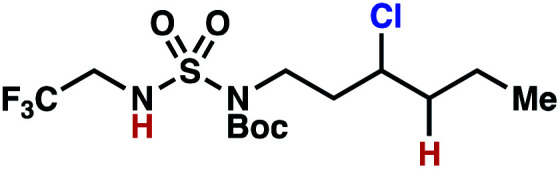	**4o**	11 : 1	−1.39	79
6	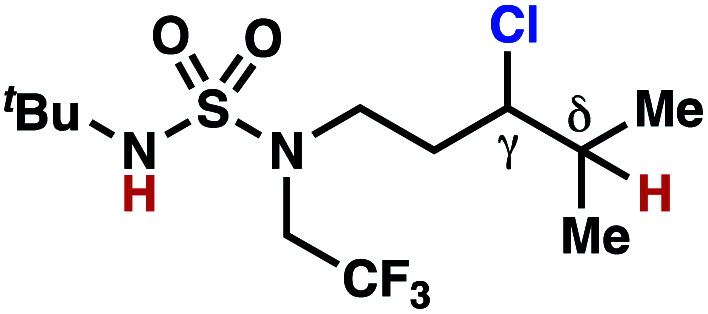	**4p**	13 : 1	−1.52	70
7	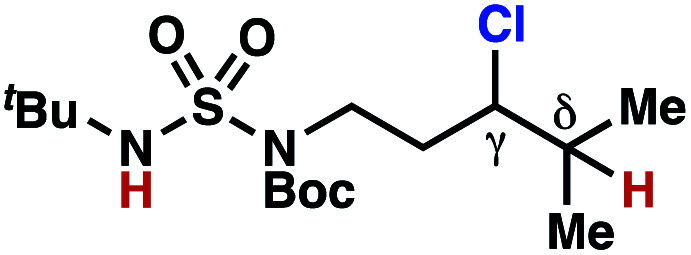	**4q**	2 : 1	−0.41	98^g^
8	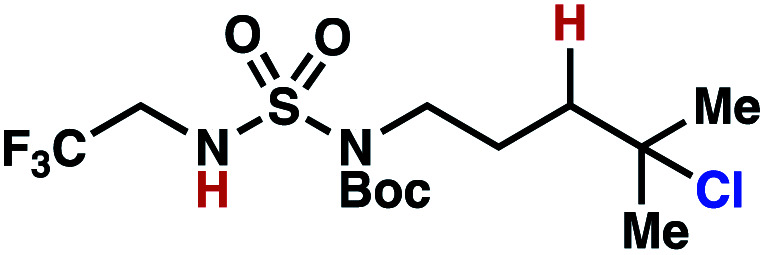	**7r**	1 : 2	+0.38	84^g^
9^h^	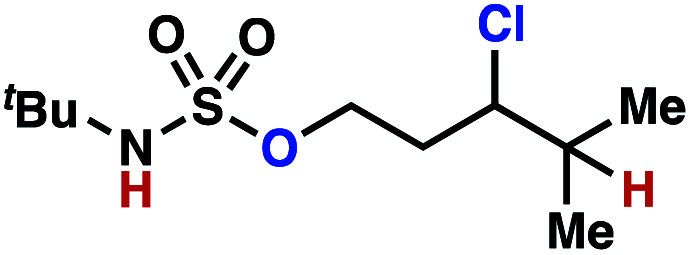	**10a**	3 : 1	−0.69	57^g^
10^h^	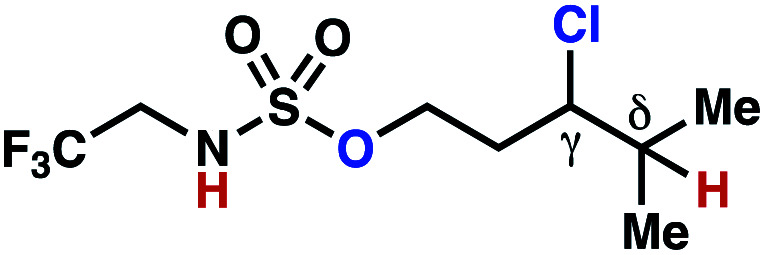	**10b**	4 : 1	−0.82	67^g^

a Conditions A.

b Determined by ^1^H or ^19^F NMR of crude mixture.

c Calculated from experimental product ratios.

d Isolated yield of depicted product.

e δ-Chlorinated-isomer not detected.

f Calculated assuming ≥20 : 1 ratio of **4** : **7**.

g Isolated as a mixture of γ- and δ-chlorinated isomers.

h Conditions: 1.0 equiv. *N*-chlorosulfamate **9**, PhH (0.07 M), 2 blue Kessil lamps.^11^

The ratio of γ- and δ-chlorinated **4** to **7** is sensitive to variations in substrate structure. Predictably, a C(6) ester inductively deactivates the δ-C–H bond to reaction, such that γ-chlorinated **4k** forms exclusively (entry 1). Unexpectedly, the ratio of γ- to δ-chlorinated **4** to **7** increases as substituents on the tertiary nitrogen of the sulfamide become more electron withdrawing, from *tert*-butoxycarbonyl-, to acetyl-, to trifluoroacetyl-, and 2,2,2-trifluoroethyl-groups (Scheme 3B; Table 2, entries 2–4). This is the opposite of the trend that would be predicted based on BDEs. By contrast, the ratio of γ- to δ-chlorinated products decreases as substituents on the secondary nitrogen of the sulfamide decrease in electron density from *tert*-butyl- to 2,2,2-trifluoroethyl-groups (Scheme 3B; Table 2, entries 5 and 8).

These trends are more evident with substrates bearing γ-secondary C–H bonds and weaker, δ-tertiary C–H bonds (entries 6–8). Moreover, the effects of these trends are synergistic. Indeed, the combination of these trends can be used to favor the formation of δ-chlorinated **7r** as the major product, possibly based on an eight-membered transition state (entry 8).

By contrast, with sulfamate esters **9**, substitution has a less pronounced effect on product ratios, with an influence over the ratio of γ- to δ-chlorinated product isomers that is not statistically significant (Table 2, entries 9–10). Apparently, sulfamate ester- and sulfamide-directed processes differ substantively owing to the marked influence of nitrogen substituents on the site of sulfamide-directed HAT processes. This pronounced effect distinguishes this method from comparable sulfamate ester-templated reactions.

To the best of our knowledge, this is the first series of experimental data to provide evidence of the relative transition state barriers for competitive intramolecular radical-mediated processes. As such, we anticipate that the data published herein can serve as a benchmark that can be used to gauge the quality of computational transition state calculation methods.

In general, care should be taken when calculating transition state energies between radical intermediates. Few data sets highlight differences in barrier heights for competitive radical-mediated reaction pathways.^32*c*^ Consequently, the quality of transition state calculations in radical pathways is often inferred based on agreement between computational methods. In such cases, extremely simple systems have been employed to provide limited experimental input regarding transition state energy measurements.^32^

**Fig. 1:**
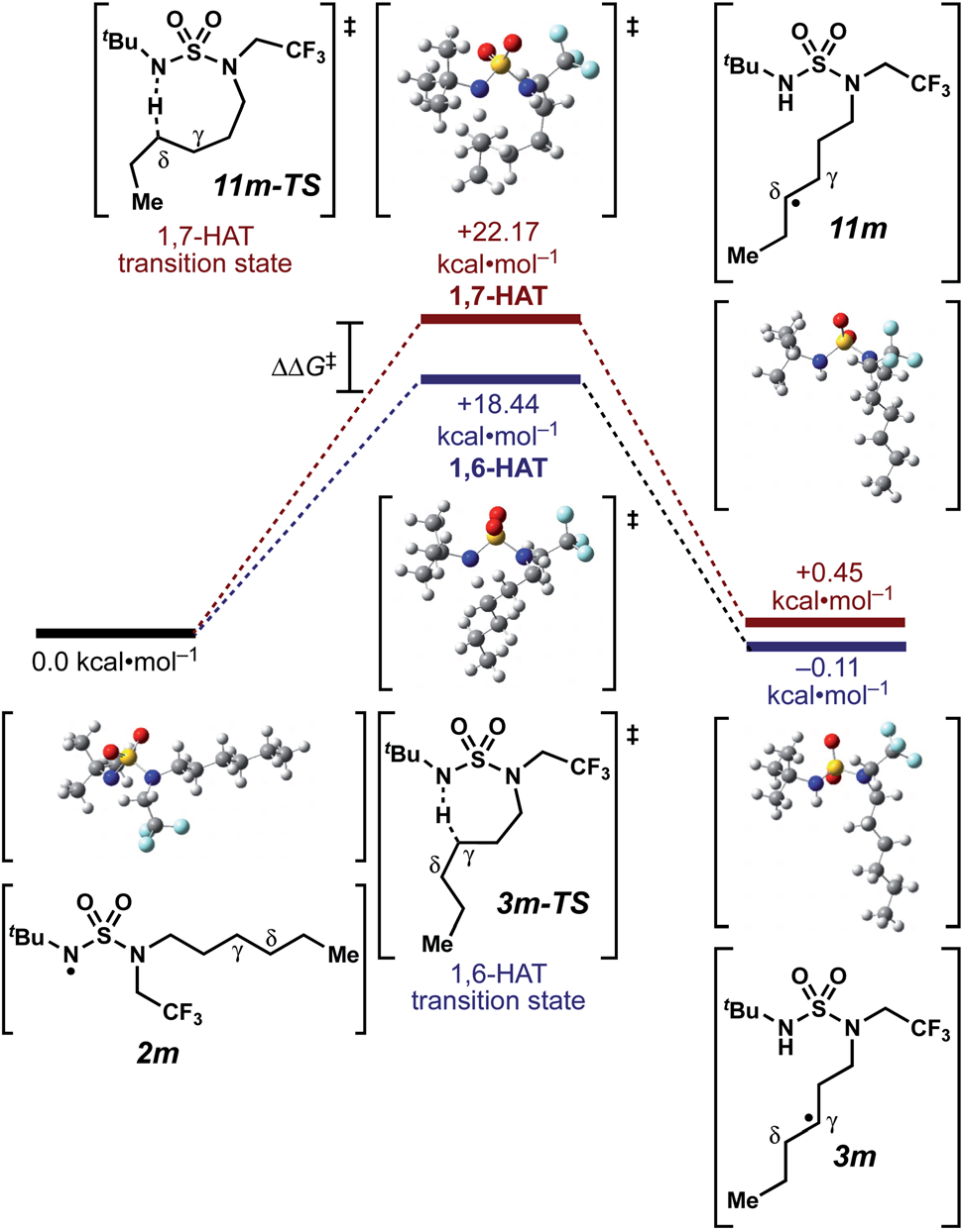
Representative example of calculated energies and structures for competing 1,6- and 1,7-HAT pathways. Density functional calculations were performed using Gaussian 09 (revision D.01) using the μB3LYP functional and the 6-31+G(d,p) basis set. See ESI† for further computational details.

We set out to evaluate the ability of one of the more commonly recommended functional/basis set combinations to recapitulate qualitative trends in barrier heights associated with the disclosed radical-mediated transformations. To this end, we have modeled the product-determining intermediates (**2**, **3**, **11**) and transition states (**3-TS**, **11-TS**) for a subset of sulfamide and sulfamate ester chlorine-transfer processes using density functional theory (DFT, Fig. 1, Table 3). Using the uB3LYP functional and the 6-31+G(d,p) basis set, we observe that the DFT method over-predicts the stability of the 7-membered ring transition states adopted for 1,6-HAT processes with both sulfamate ester substrates (entries 1 and 2) as well as the hexyl-derived sulfamide substrates (entries 3–5). Within these classes of compounds, calculations qualitatively correlate well with experimental results.

**Table 3:** Comparison of calculated to experimental ΔΔ*G*^‡^ values

Entry	Parent compound	Experimental ΔΔ*G*^‡^^a^ (kcal mol^−1^)	Calculated ΔΔ*G*^‡^^b^ (kcal mol^−1^)
1	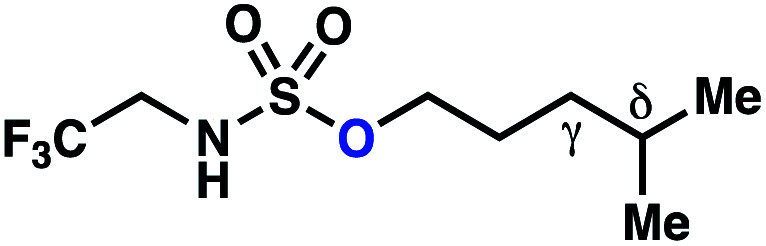	−0.82	−3.33
2	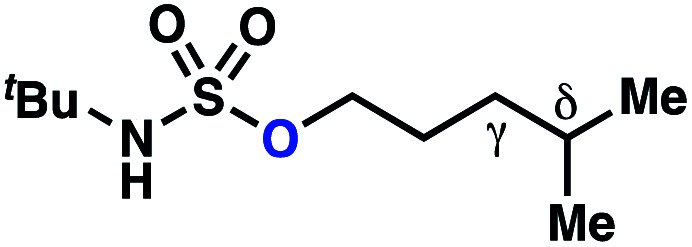	−0.69	−1.09
3	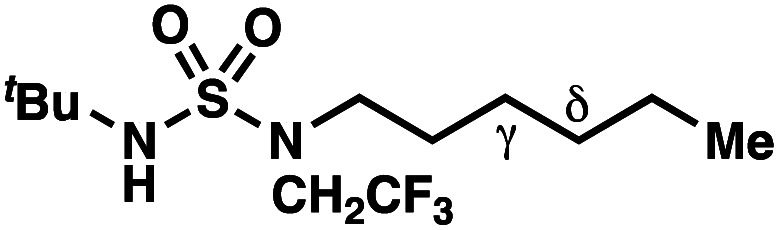	≤−1.77	−3.74
4	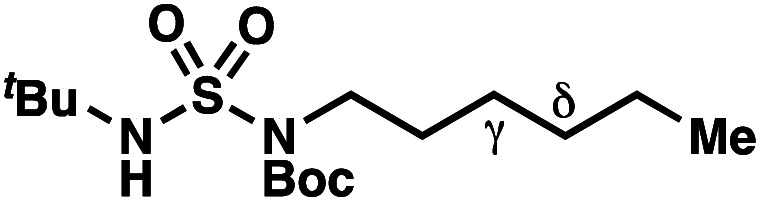	−1.56	−2.98
5	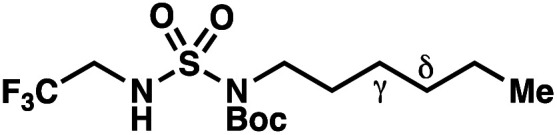	−1.39	−2.06
6	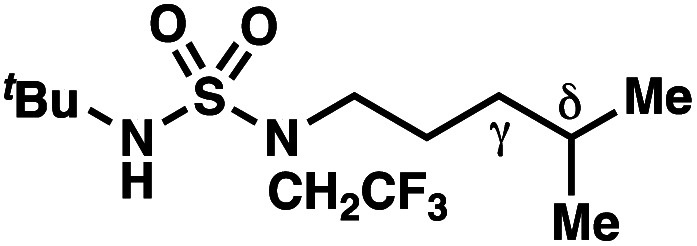	−1.52	+0.35
7	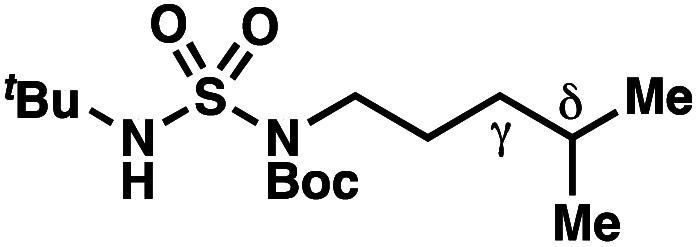	−0.41	+2.24
8	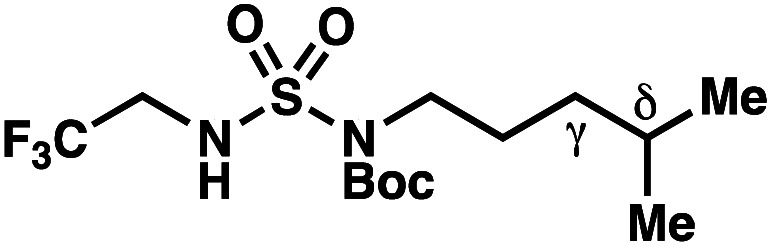	+0.38	+1.04

a ΔΔ*G*^‡^ = −*RT* ln(γ-chlorinated product/δ-chlorinated product) ad determined by ^1^H or ^19^F NMR of crude reaction mixture.

b ΔΔ*G*^‡^ = (Δ*G*(1,6-HAT TS) − Δ*G*(1,7-HAT TS)) as determined from the calculated Gibbs free energies using uB3LYP/6-31+G(d,p).

By contrast, experimental and calculated differences in transition state barriers are poorly correlated when performed on 4-methylpentyl-derived sulfamides (entries 6–8), where 1,6-HAT results in abstraction of a hydrogen atom from a secondary center (BDE ≈ 98 kcal mol^−1^) and 1,7-HAT requires abstraction from a weaker tertiary center (BDE ≈ 96 kcal mol^−1^). The qualitative inconsistency between our experimental and computational results is evidence that our synthetically oriented community should exercise extreme caution when making claims based on calculated energies for transition state barriers between radical intermediates.

Rigorous experiments can provide insight into the mechanism of these chlorine-transfer reactions. In principle, chlorine-transfer could involve a radical chain propagation mechanism or a closed reaction pathway. To initiate either of these processes, light-promoted N–Cl bond homolysis would convert *N*-chlorosulfamide **1h** to chlorine radical and nitrogen-centered radical **2h**. Sulfamidyl radical **2h** then performs a site-selective hydrogen-atom abstraction through a seven-membered transition state to generate carbon-centered radical **3h**.

**Scheme 4:**
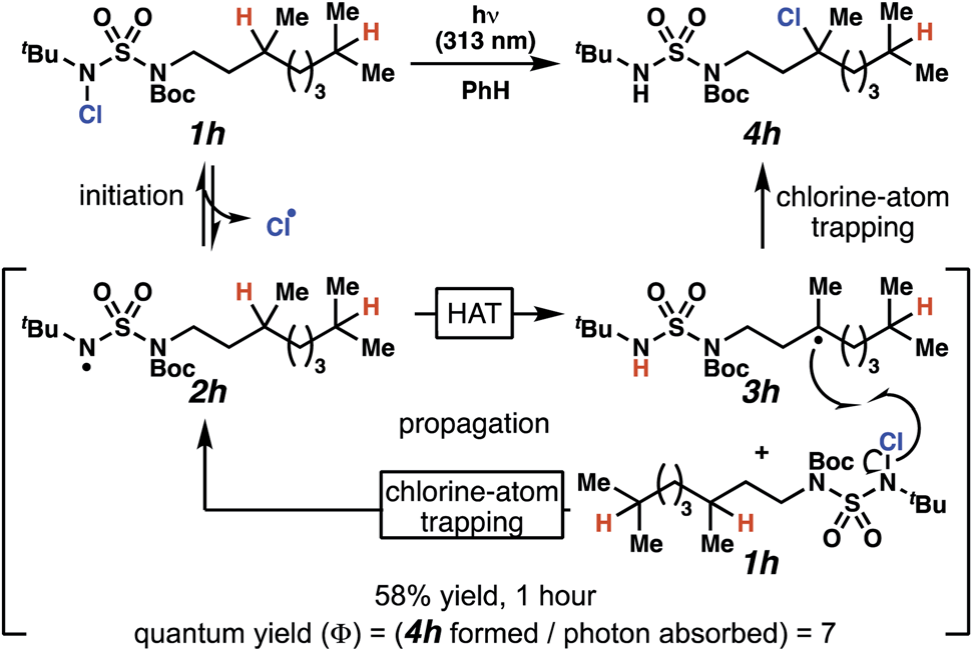
Chlorination proceeds through a light-initiated chain propagation.

The two feasible reaction paths differ in terms of the carbon–chlorine bond forming events. In a radical-chain propagation process, carbon-centered radical **3h** engages another equivalent of *N*-chlorosulfamide substrate **1h** in chlorine-atom abstraction (Scheme 4). This sequence would produce desired chlorinated **4h** along with another equivalent of nitrogen-centered radical **2h**, which would propagate this chain reaction. Alternatively, in a closed reaction mechanism, intermediate carbon-centered radical **3h** would recombine with the initially generated chlorine radical to terminate the reaction and afford chlorinated **4h** (not depicted).

These reaction pathways would differ in terms of the equivalents of product formed per absorbed photon, defined as the quantum yield (*Φ*). In a radical chain propagation process, each absorbed photon could initiate the formation of multiple equivalents of product (*Φ* > 1). By contrast, in a closed process, each absorbed photon could initiate the preparation of a maximum of one product molecule (*Φ* ≤ 1).

Quantum yield measurements suggest that the reaction engages a light-initiated chain propagation mechanism. To determine the number of photons available to a sample in a fluorimeter, we rely on standard chemical actinometry using potassium ferrioxalate at 313 nm.^33,34^ In the calibrated fluorimeter, 1 hour of irradiation of *N*-chlorinated **1h** in benzene furnishes chloroalkane **4h** in 58% isolated yield. This yield indicates that at least 7 equivalents of product have formed for each absorbed photon (*Φ* = 7), a value that is consistent with chlorination *via* a radical chain propagation process.

**Scheme 5:**
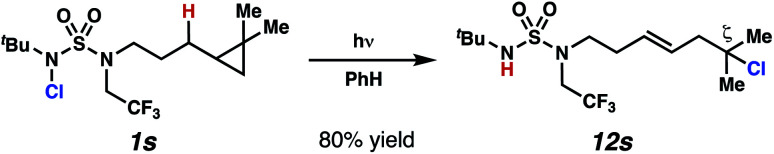
Cyclopropyl-containing substrate provides evidence for radical reaction pathway.

In spite of the rapid speed of radical chain propagation, the generated radical has a long enough lifetime to promote ring-opening of an appropriately positioned cyclopropane (Scheme 5). Upon photoirradiation, *N*-chlorosulfamide **1s** reacts to furnish ring-opened isomer **12s** in 80% yield, with exclusive detection of ring-opened products. This cascade sequence provides position-selective access to a more distally ζ-chlorinated product with an intervening olefin. The presence of an intact olefins is interesting, as alkenes are not tolerated under typical *N*-chlorination conditions.

## Conclusions

These investigations demonstrate that sulfamides guide 1,6-HAT processes. This mechanistic manifold has been employed to access alkyl chlorides, which are high-value synthetic intermediates. Consequently, this sulfamide-directed process establishes the premise for a broadly translatable γ-C(sp^3^)–H functionalization approach that complements known alkane functionalization technologies.

Furthermore, these investigations establish that sulfamide substitution can be used to predictably vary the site-selectivity of C–H abstraction processes. Initial calculations have not qualitatively recapitulated experimental trends. Fortunately, the mere ability to experimentally quantify the relative transition state barriers for two competing radical-mediated reaction steps is of benefit as a benchmark for computational methods, where quantitative data relating to barrier heights is scarce.

## Conflicts of interest

There are no conflicts to declare.

## Supplementary Material

SC-011-C9SC03428E-s001
