# Supplementary material for: Sulfamides direct radical-mediated chlorination of aliphatic C–H bonds
Source: Chem Sci. 2019 Nov 8;11(1):217–23. doi: 10.1039/c9sc03428e (PMC8132995; doi:10.1039/c9sc03428e)
Supplement: SC-011-C9SC03428E-s001 [file SC-011-C9SC03428E-s001.pdf]

Electronic Supplementary Material (ESI) for Chemical Science.
This journal is © The Royal Society of Chemistry 2019

Supporting Information

Sulfamides Direct Radical-Mediated Chlorination of Aliphatic C–H Bonds

M. A. Short, M. F. Shehata, M. A. Sanders, and J. L. Roizen*

Department of Chemistry, Duke University, Durham, NC 27708-0354

Table of Contents:

**I.**	**General considerations.**	**S2**
**II.**	**Reaction optimization.**	**S5**
**III.**	**Experimental procedures.**	**S6**
**IV.**	**Preparation and characterization of sulfamides.**	**S13**
**V.**	**Preparation and characterization of diversified sulfamides.**	**S16**
**VI.**	**Preparation and characterization of *N*-chlorosulfamides.**	**S27**
**VII.**	**Preparation and characterization of γ-chlorinated sulfamides.**	**S37**
**VIII.**	**Preparation and characterization of non-γ-chlorinated sulfamides.**	**S49**
**IX.**	**Preparation and characterization of sulfamate ester derivatives.**	**S52**
**X.**	**Sulfamide cleavage to access 3-chloroalkylamines.**	**S58**
**XI.**	**Preparation and characterization of compounds related to the radical clock experiment.**	**S59**
**XII.**	**Description of quantum yield experiment.**	**S61**
**XIII.**	**UV-Vis spectra.**	**S64**
**XIV.**	**S–N bond lengths in related sulfamides.**	**S66**
**XV.**	**Computational calculations.**	**S68**
**XVI.**	**References.**	**S122**
**XVII.**	**NMR spectra.**	**S125**

I. General considerations.

Reagents.

All reagents and chemicals were obtained commercially and used without further purification unless otherwise noted.

Acros	sulfur trioxide pyridine complex, 1,10-phenanthroline, potassium oxalate monohydrate, trimethylacetyl chloride, 1-hexanol, *tert*-butylamine
Alfa Aesar	oxone, triphenylphosphine
Ark Pharm	4-(trifluoromethyl)hydrocinnamic acid
BDH	ammonium hydroxide solution (28% w/w NH_3 in H_2O)
BTC Chemicals	trimethylacetonitrile
Chem Impex	di *tert*-butyl pyrocarbonate, iron(III) chloride hexahydrate, 2-iodobenzoic acid
Clorox	aqueous sodium hypochlorite solution (bleach, 8.25%)
EMD Millipore	benzene (anhydrous, PhH)
Fisher Scientific	acetonitrile (ACS grade), hexanes, sodium sulfate (anhydrous), magnesium sulfate (anhydrous)
Fluka	hydrogen bromides solution (32% in acetic acid)
Oakwood Chemical	2,2,2-trifluoroethylamine, triethylamine, trifluoroacetic anhydride, trifluoromethanesulfonic anhydride, trifluoroacetic acid, triphenylphosphine oxide, trichloroisocyanuric acid (TCICA), phthalimide, chlorosulfonyl isocyanate, 1,1'-carbonyldiimidazole (CDI), hexylamine, 2-cyclohexylethanol
Sigma-Aldrich	hydrochloric acid (concentrated), lithium aluminium hydride, 4-dimethylaminopyridine (DMAP), *tert*-butylamine, 4-aminobutyric acid, 3,7-dimethyloctan-1-ol, *tert*-butanol, 1,1'-carbonyldiimidazole (CDI), ethyl acetate (EtOAc), diethyl azidocarboxylate, acetic anhydride
TCI	amylamine, isopentylamine (isoamylamine)
Soap Goods	sodium acetate (anhydrous), sodium hydroxide (pellets)

Anhydrous 1,4-dioxane, tetrahydrofuran (THF), dichloromethane (CH_2Cl_2), and diethyl ether (Et_2O) were obtained from Sigma Aldrich and were purified, dried, and degassed by passage through two columns of neutral, activated alumina under N_2 using an Innovative Technologies solvent purification system. Anhydrous toluene was purified, dried, and degassed by passage through a column containing copper followed by a column of neutral, activated alumina under N_2 using an Innovative Technologies solvent purification system. Triethylamine (Et_3N) was distilled from CaH_2 and stored in a Schlenk flask for future use.

Preparation of Known Reagents.

Tert-butyl hypochlorite,[1] potassium ferrioxalate trihydrate,[2] triethylammonium sulfamate salts,[3] neopentylamine,[4] methyl 6-aminohexanoate hydrochloride,[5] 4-chloropentanol,[6] *tert*-butyl 4-aminobutyrate,[7] (*R*)-methyl-2-amino-4-methylpentanoate hydrochloride,[8] (*S*)-2-(1-hydroxy-3-methylbutan-2-yl)isoindoline-1,3-dione,[9] 2-chloro-4-methylpentanol,[10] *N*-(*tert*-butyl)-*N'*-(2,2,2-trifluoroethyl)sulfamide,[11] and 3-(2,2,-dimethylcyclopropyl)propan-1-ol[12] were prepared according to the literature and stored at –20 °C.

Procedures.

Moisture-sensitive reactions were performed using flame-dried glassware under an atmosphere of dry nitrogen (N_2). Where noted, air- and water-sensitive reactions were performed in an MBraun MB200 glovebox held under an atmosphere of nitrogen gas (working pressure 2–6 mbar). Flame-dried equipment was stored in a 130 °C oven before use and either allowed to cool in a cabinet dessicator or assembled hot and allowed to cool under an inert atmosphere. Air- and moisture-sensitive liquids and solutions were transferred *via* plastic or glass syringe. Chromatographic purification of products was accomplished using Silicycle Silica flash F60 (particle size 40–63 μm, 230–400 mesh), Teledyne Isco CombiFlashRf system using 12–220 g Redi Sep Rf normal phase silica columns (particle size 40–63 μm irregular, 230–400 mesh), or Alfa Aesar Florisil (100–200 mesh). Thin layer chromatography was performed on EMD Millipore silica gel 60 F254 glass-backed plates (layer thickness 250 μm, particle size 10–12 μm, impregnated with a fluorescent indicator). Visualization of the developed chromatogram was accomplished by fluorescence quenching under shortwave UV light and/or by staining with *p*-anisaldehyde or $KMnO_4$ stains. Unless otherwise noted, room temperature is 22 ºC.

Instrumentation.

NMR Spectrometry
NMR spectra were obtained on Varian iNOVA spectrometers operating at 400 or 500 MHz for ^{1}H NMR, 101 or 126 MHz for ^{13}C NMR, and 376 MHz for ^{19}F NMR at 23–25 °C, and are reported as chemical shifts (δ) in parts per million (ppm). Spectra were referenced internally according to residual solvent signals (^{1}H: $CDCl_3$, 7.26 ppm; C_6D_6, 7.16 ppm; CD_3CN, 1.94; ^{13}C: $CDCl_3$, 77.0 ppm; C_6D_6, 128.0; CD_3CN, 1.3, 118.3 ppm). Data for NMR spectra use the following abbreviations to describe multiplicity: s, singlet; br s, broad singlet; d, doublet; t, triplet; q, quartet; dd, doublet of doublets; td, triplet of doublets; ddd, doublet of doublet of doublets; ddt, doublet of doublet of triplets; app dd, apparent doublet of doublets; ABq, AB system quartet; m, multiplet. Coupling constant (*J*) are reported in units of Hertz (Hz).

IR Spectroscopy
IR spectra were obtained on a Nicolet 6700 FT-IR system. Peaks are reported in cm^{-1} with indicated relative intensities: s (strong, 0–33% T); m (medium, 34–66% T); w (weak, 67–100% T); and br (broad).

Mass Spectrometry
High resolution mass spectra (HRMS, m/z) were recorded on an Agilent LCMS-TOF-DART spectrometer using electrospray ionization (ESI, Duke University Department of Chemistry Instrumentation Center).

UV-Vis Spectroscopy
UV-vis spectra were obtained on an Agilent Cary 50 UV-Vis Spectrophotometer.

Light Source
• Southern New England Ultraviolet Co. RPR-100 Photochemical Reactor
Lamp: 35W RPR-2537A
Emission wavelength: 254 nm
Reactor Barrel Dimensions: 10” diameter, 15” depth
Power supply: 110/120V AC (Input), 50/60 Hz Cycle
Power usage: ~400W
https://rayonet.org/reactors.php?part=RPR-100

II. Optimization of Sulfamide-Guided Chlorine-Transfer.

General Procedure for Optimization.

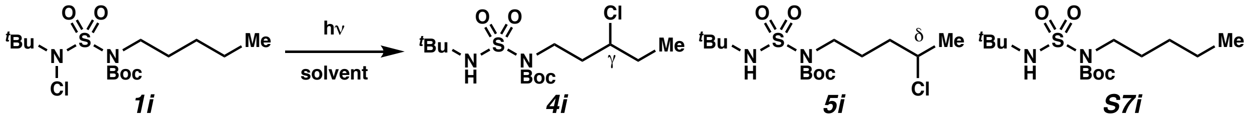

A flame-dried 1 dram vial equipped with a stir bar was charged with chlorosulfamide **1i** (11 mg, 0.03 mmol, 1.0 equiv). The vial was transferred to a nitrogen-filled glovebox where it was charged with anhydrous solvent (0.75 mL, 0.04 M or 0.3 mL, 0.1 M). The vial was sealed with a Teflon lined screw cap and removed from the glovebox. The vial was placed on a stir plate and irradiated for the reported time by the light source as described below.

Upon completion of the reaction, the solution was concentrated under reduced pressure and 1,2,4,5-tetrachloro-3-nitrobenzene (0.6 mL, 7.8 mg, 0.03 mmol, 1.0 equiv) was added from a stock solution (0.05 M). The crude was analyzed by ^{1}H NMR with 25 second relaxation delay and the yields were calculated.

Table S1. Optimization of reaction solvent and light source.

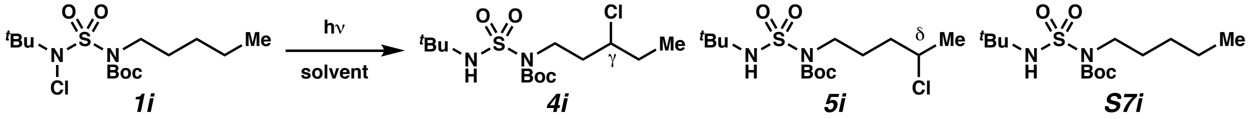

entry	solvent	light source	time	yield **4i**[b]	yield **5i**[b]	yield **S7i**[b]
1[c]	PhH	CFL lamps	2 h	55%	20%	23%
2	PhH	CFL lamps	2 h	65%	13%	17%
3[c]	PhH	Kessil lamps	1 h	60%	16%	20%
4	PhH	Kessil lamps	1 h	56%	16%	20%
5[c]	PhH	UV reactor	15 min	65%	12%	15%
**6[d]**	**PhH**	**UV reactor**	**15 min**	**88%**	**10%**	**4%**
7	MeCN	UV reactor	15 min	85%	9%	7%
8	^{i}PrOAc	UV reactor	15 min	85%	9%	8%
9	PhCl	UV reactor	15 min	61%	14%	19%
10	$PhCF_3$	UV reactor	15 min	78%	9%	6%

[a]Reactions performed on 0.03 mmol scale at 0.04 M in the listed solvent unless otherwise noted. [b]Yields from ^{1}H NMR of crude reaction mixture with 1,2,4,5-tetrachloro-3-nitrobenzene (1.0 equiv) added as an internal standard. [c]Performed at 0.1 M. [d]Reported as an average of two runs.

III. Experimental Procedures.

Preparation of 4-Chlorohexanol (S1)

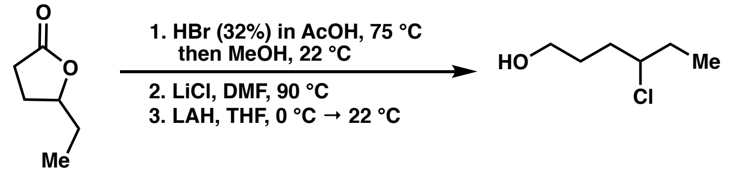

Inspired by a procedure reported by Alexanian and coworkers,[6] a flame-dried round bottom flask was charged with γ-valerolactone (2.2 mL, 20.0 mmol, 1.0 equiv) and hydrogen bromide solution (32% in acetic acid, 5 mL, 27 mmol, 1.4 equiv). The flask was fitted with a reflux condenser with rubber septum and nitrogen inlet and heated in an oil bath set at 75 °C for 4 h. After this time, the flask was removed from the heat and allowed to cool to room temperature. Once cooled, freshly distilled methanol (8.0 mL, 2.5 M) was added and the reaction was stirred at 22 °C for 16 h. After 16 h, the reaction was partially concentrated under reduced pressure (to remove methanol) and taken up in EtOAc (10 mL). The mixture was then transferred to a separatory funnel, rinsing the flask with EtOAc to achieve quantitative transfer. The organic phase was washed three times with saturated aqueous $NaHCO_3$ (3 x 25 mL) and once with brine (25 mL). The organic phase was dried with $MgSO_4$, filtered, and concentrated under reduced pressure. The crude bromoester was used in the next step without further purification.

The crude bromoester was taken up in anhydrous DMF (13 mL) and LiCl (1.67 g, 39.3 mmol, 2.0 equiv) was added in a single portion. The flask was fitted with a Vigreux column with rubber septum and nitrogen inlet and heated in an oil bath set at 90 °C for 18 h. After 18 h, the reaction flask was removed from the heat and allowed to cool to room temperature. Once cool, the reaction was diluted with Et_2O (30 mL) and 1 M HCl (30 mL). The biphasic mixture was transferred to a separatory funnel, rinsing the flask with Et_2O to achieve quantitative transfer. The aqueous phase was removed and the organic phase was washed twice more with 1 M HCl (2 x 30 mL) and once with brine (30 mL). The organic phase was dried with $MgSO_4$, filtered, and concentrated under reduced pressure. The crude chloroester was used in the next step without purification.

The crude chloroester was added dropwise to a suspension of lithium aluminum hydride (797 mg, 21.0 mmol, 1.1 equiv) in Et_2O (45 mL, 2.3 M) that had been cooled at 0 °C in an ice water bath. The reaction was left to stir at 0 °C for 30 minutes and then heated to reflux overnight. After refluxing overnight, the flask was again cooled to 0 °C, and the reaction was carefully quenched by sequential dropwise addition of H_2O (0.8 mL), 15% aqueous NaOH (0.8 mL), and H_2O (0.8 mL). The resulting suspension was filtered by vacuum filtration through a pad of celite in a glass fritted funnel. The filtrate was concentrated under reduced pressure and then purified by silica gel flash chromatography by dry loading the sample and eluting with hexanes:EtOAc (Gradient: 100% hexanes → 9:1 hexanes/EtOAc) to yield the desired product as a colorless oil (390 mg, 14% yield over 3 steps).

^{1}H NMR (400 MHz, $CDCl_3$) δ 3.88 (tt, J = 4.4 Hz, 8.3 Hz, 1H), 3.69 (t, J = 6.0 Hz, 2H), 1.91–1.66 (m, 6H), 1.44 (br s, 1H), 1.03 (t, J = 7.3 Hz, 3H).
^{13}C NMR (126 MHz, $CDCl_3$) δ 65.5, 62.2, 34.3, 31.5, 29.6, 10.9.

IR (neat) ν 3326 (br), 2940 (m), 2877 (m), 1456 (m), 1381 (m), 1310 (m), 1236 (m), 1173 (w), 1059 (s), 921 (s), 896 (m), 803 (m), 734 (m), 658 (m), 603 (s) cm^{-1}.
TLC R_f = 0.21 in 4:1 hexanes:EtOAc
HRMS (ESI) m/z: $[M + H – H_2O]^+$ Calcd for $C_6H_{13}ClO$ 119.0622; Found 119.0621.

Preparation of 3,7-Dimethyloctanoic acid (S2h).

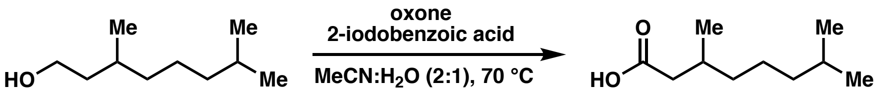

Following a modified literature procedure,[13] a round-bottom flask with stir bar was charged with 3,7-dimethyloctanol (6.0 mL, 31.4 mmol, 1.0 equiv) without taking any precautions to exclude air or moisture. The flask was charged with acetonitrile (285 mL, 0.11 M) and deionized water (143 mL, 0.22 M), followed by 2-iodobenzoic acid (2.34 g, 9.4 mmol, 0.3 equiv) and then oxone (25.13 g, 40.8 mmol, 1.3 equiv), each in single portions. The reaction flask was fitted with a reflux condenser and heated in an oil bath at 70 °C overnight (16 h) open to air.

After heating overnight, the flask was removed from the oil bath and allowed to cool to room temperature. Once cool, the solid material was removed by vacuum filtration using a Büchner funnel, and the filter cake was rinsed with additional acetonitrile (ca. 100 mL). The filtrate was concentrated under reduced pressure to remove acetonitrile. The remaining aqueous phase was extracted with CH_2Cl_2 (3 x ca. 200 mL). The combined organic phases were dried over Na_2SO_4, filtered, and concentrated under reduced pressure. The crude material was purified by silica gel flash chromatography by dry loading the sample and eluting with hexanes:EtOAc (9:1 hexanes/EtOAc) to yield the desired product as a colorless oil (3.78 g, 70% yield).

The characterization data for this compound were in agreement with previously published information.[14]

^1H NMR (400 MHz, $CDCl_3$) δ 2.36 (dd, *J* = 14.9 Hz, 5.9 Hz, 1H), 2.14 (dd, *J* =14.9 Hz, 8.2 Hz, 1H), 2.00–1.92 (m, 1H), 1.53 (heptet, *J* = 6.6 Hz, 1H), 1.33–1.13 (m, 6H), 0.97 (d, *J* = 6.6 Hz, 3H), 0.87 (d, *J* = 6.6 Hz, 6H).
TLC R_f = 0.27 in 9:1 hexanes:EtOAc

General Procedure A: Preparation of amides from carboxylic acids.

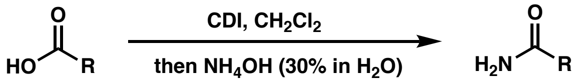

A flame-dried flask with stir bar was charged with carboxylic acid (1.0 equiv) and fitted with a rubber septum. The flask was evacuated and backfilled with N_2. ACS grade CH_2Cl_2 (0.3 M) was added via syringe. Carbonyl diimidazole (CDI, 1.25 equiv) was then added by briefly removing the septum and adding the solid as a single portion. The reaction was left to stir at 22 °C for 1 h. After 1 h, the reaction flask was cooled at 0 °C in an ice water bath and aqueous NH_4OH (30%) (3.6 equiv) was added via syringe. Following NH_4OH addition, the reaction was allowed to slowly warm to room temperature by not removing the ice bath and stirred overnight.

After stirring overnight, the biphasic suspension was transferred to a separatory funnel and diluted with deionized H_2O (ca. amount of CH_2Cl_2). The reaction flask was rinsed with an additional 5 mL CH_2Cl_2 to ensure quantitative transfer. The organic phase was removed and the aqueous was extracted twice more with CH_2Cl_2. The combined organic phases were dried over Na_2SO_4, filtered, and concentrated under reduced pressure. The crude material was purified by silica gel flash chromatography eluting with a CH_2Cl_2:EtOAc solvent system as noted below.

2-Cyclopentylacetamide (S3d)

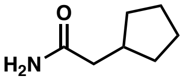

Prepared from cyclopentylacetic acid (0.96 g, 7.5 mmol, 1.0 equiv), CDI (1.52 g, 9.4 mmol, 1.25 equiv), and aq NH_4OH (3.5 mL, 26.0 mmol, 3.6 equiv) following general procedure A. The product was obtained as a white solid (763 mg, 80% yield) following silica gel column chromatography (1:1 CH_2Cl_2:EtOAc).

^{1}H NMR (400 MHz, $CDCl_3$) δ 5.85 (br s, 1H), 5.53 (br s, 1H), 2.24–2.15 (m, 3H), 1.87–1.82 (m, 2H), 1.64–1.50 (m, 4H), 1.19–1.11 (m, 2H).
^{13}C NMR (126 MHz, $CDCl_3$) δ 175.7, 42.1, 36.9, 32.5, 24.9.
IR (neat) ν 3346 (br m), 3168 (br m), 2951 (m), 2867 (m), 1661 (s), 1629 (s), 1412 (s), 1353 (m), 1308 (m), 1250 (m), 1156 (m), 952 (w), 882 (w), 809 (w), 672 (s) cm^{-1}.
TLC R_f = 0.19 in 1:1 CH_2Cl_2:EtOAc.
HRMS (ESI) m/z: $[M + H]^+$ Calcd for $C_7H_{13}NO$ 128.1070; Found 128.1068.

3,7-Dimethyloctylamide (S3h)

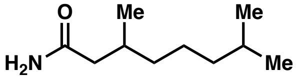

Prepared from 3,7-dimethyloctanoic acid (**S2h**) (5.17 g, 30.0 mmol, 1.0 equiv), CDI (6.1 g, 37.6 mmol, 1.25 equiv), and aq NH_4OH (14 mL, 108 mmol, 3.6 equiv) following general procedure A. The product was obtained as a white solid (4.38 g, 85% yield) following silica gel column chromatography (1:1 CH_2Cl_2:EtOAc).

The characterization data for this compound were in agreement with previously published information.[15]

^{1}H NMR (400 MHz, $CDCl_3$) δ 5.57 (br s, 1H), 5.42 (br s, 1H), 2.26–2.19 (m, 1H), 2.00–1.90 (m, 2H), 1.52 (heptet, J = 6.6 Hz, 1H), 1.36–1.10 (m, 6H), 0.95 (d, J = 6.2 Hz, 3H), 0.86 (d, J = 6.6 Hz, 6H).
TLC R_f = 0.39 in 1:1 CH_2Cl_2:EtOAc

3-(4-Trifluoromethylphenyl)propamide (S3u)

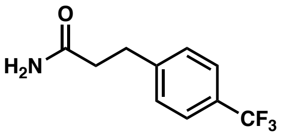

Prepared from 4-(trifluoromethyl)hydrocinnamic acid (2.2 g, 10.0 mmol, 1.0 equiv), CDI (2.03 g, 12.5 mmol, 1.25 equiv), and aq NH_4OH (4.7 mL, 36.2 mmol, 3.6 equiv) following general procedure A. The product was obtained as a white solid (1.78 g, 82% yield) following silica gel column chromatography (1:1 CH_2Cl_2:EtOAc).

^{1}H NMR (400 MHz, $CDCl_3$) δ 7.54 (d, J = 8.0 Hz, 2H), 7.33 (d, J = 8.0 Hz, 2H), 5.50 (br s, 1H), 5.37 (br s, 1H), 3.03 (t, J = 7.6 Hz, 2H), 2.54 (t, J = 7.6 Hz, 2H).

^{13}C NMR (126 MHz, $CDCl_3$, ^{1}H and ^{19}F decoupled) δ 173.9, 144.8, 128.7, 125.4, 124.2, 36.9, 31.0.
^{19}F NMR (376 MHz, $CDCl_3$) δ –62.45.
IR (neat) ν 3399 (br w), 3199 (br w), 2947 (w), 1652 (m), 1615 (m), 1453 (w), 1418 (m), 1320 (s), 1246 (w), 1167 (s), 1108 (s), 1066 (s), 1018 (m), 954 (w), 912 (w), 896 (w), 837 (s), 825 (s), 733 (w), 592 (s) cm^{-1}.
TLC R_f = 0.22 in 1:1 CH_2Cl_2:EtOAc.
HRMS (ESI) m/z: $[M + H]^+$ Calcd for $C_{10}H_{10}F_3NO$ 218.0787; Found 218.0783.

General Procedure B: Preparation of amines from amides.

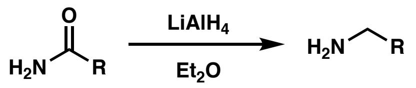

A flame-dried flask with stir bar and fitted with a reflux condenser was charged with lithium aluminum hydride ($LiAlH_4$) (2.1 equiv). The flask was fitted with a rubber septum and evacuated and back-filled with N_2. Anhydrous Et_2O (0.2 M) was added and the reaction flask was cooled at 0 °C in an ice/water bath. Amide (1.0 equiv) was carefully added portion-wise as a solid (ca. 3 portions) by briefly removing the reflux condenser and then replacing it. Once all amide had been added, the ice/water bath was replaced with an oil bath and the reaction flask was heated at reflux and stirred overnight.

After refluxing overnight, the reaction was removed from the heat and allowed to cool to room temperature and then further cooled at 0 °C in an ice/water bath. The reaction was then carefully quenched by careful, sequential addition of deionized H_2O (1 mL/g $LiAlH_4$), 15% w/v aq. NaOH (1 mL/g $LiAlH_4$), and deionized H_2O (1 mL/g $LiAlH_4$). The quenched reaction was left stirring at 0 °C for ca. 30 minutes to ensure complete quenching of remaining $LiAlH_4$. Once fully quenched, the reaction was filtered through a pad of celite in a glass-fritted funnel by vacuum filtration eluting with Et_2O (ca. ½ reaction volume). The filtrate was then concentrated under reduced pressure. The concentrated crude material was generally pure without need for further purification.

3,7-Dimethyloctylamine (S4h)

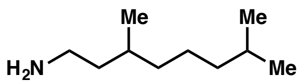

Prepared from 3,7-dimethyloctylamide (**S3h**, 3.8 g, 22.0 mmol, 1.0 equiv) and $LiAlH_4$ (1.75 g, 46.2 mmol, 2.1 equiv) in anhydrous Et_2O (100 mL) following general procedure B. The product was obtained as a colorless oil (3.28 g, 95% yield).

The characterization data for this compound were in agreement with previously published information. [16]

^{1}H NMR (400 MHz, $CDCl_3$) δ 2.76–2.63 (m, 2H), 1.55–1.40 (m, 3H), 1.33–1.20 (m, 5H), 1.17–1.08 (m, 2H), 0.87–0.85 (m, 9H).

3-(4-(Trifluoromethyl)phenyl)propylamine (S4u)

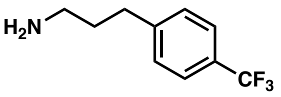

Prepared from 3-(4-trifluoromethylphenyl)propamide (**S3u**) (1.3 g, 62.0 mmol, 1.0 equiv) and $LiAlH_4$ (478 mg, 12.6 mmol, 2.1 equiv) in anhydrous Et_2O (30 mL) following general procedure B. The product was obtained as a colorless oil (1.18 g, 97% yield).

^{1}H NMR (400 MHz, $CDCl_3$) δ 7.53 (d, *J* = 8.0 Hz, 2H), 7.29 (d, *J* = 8.0 Hz, 2H), 2.76–2.70 (m, 4H), 1.79 (dt, *J* = 7.1, 14.8 Hz, 2H), 1.50 (br s, 2H).
^{13}C NMR (126 MHz, $CDCl_3$, ^{1}H and ^{19}F decoupled) δ 146.2, 128.6, 128.1, 125.2, 124.3, 41.5, 34.8, 33.0.
^{19}F NMR (376 MHz, $CDCl_3$) δ –62.35.
IR (neat) ν 2932 (br w), 2860 (br w), 1618 (w), 1418 (w), 1321 (s), 1159 (m), 1111 (s), 1065 (s), 1018 (m), 840 (m), 814 (m), 731 (w), 631 (w), 614 (w), 594 (m) cm^{-1}.
HRMS (ESI) m/z: $[M + H]^+$ Calcd for $C_{10}H_{12}F_3N$ 204.0995; Found 204.0995.

General Procedure C: Preparation of *N*-Boc free sulfamides

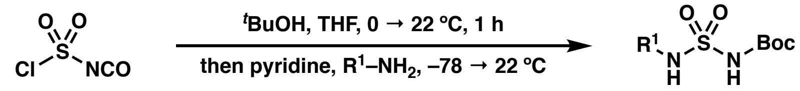

Following a modified literature procedure,[17] to a flame-dried round bottom flask fitted with a rubber septum and under an atmosphere of N_2 was added anhydrous THF (0.5 M) and chlorosulfonyl isocyanate (1.0 equiv). The solution was cooled at 0 °C in an ice water bath and ^{t}BuOH (1.0 equiv) was added dropwise via syringe. Upon complete addition of ^{t}BuOH, the solution was allowed to slowly warm to room temperature by not removing the ice water bath or adding any additional ice and was stirred for 1 h.

After 1 hour, the solution was cooled at –78 ºC in a dry ice/^{i}PrOH bath and pyridine (5.0 equiv) and amine (R^1–NH_2, 2.0 equiv) were added dropwise via syringe. Upon complete addition of amine, the cloudy mixture was allowed to slowly warm to room temperature by not removing the cooling bath or adding any additional dry ice and was stirred overnight. After stirring overnight, the suspension was cooled at 0 ºC in an ice water bath and the pH was adjusted to 1 by dropwise addition of a 6 M HCl solution. Once reaching pH = 1, the solution was diluted with H_2O (ca. 1 M) and transferred to a separatory funnel rinsing the flask with EtOAc (ca. 5 mL) to ensure quantitative transfer. The aqueous phase was extracted with EtOAc (3 x ca. 1 mL) The combined organic layers were dried over Na_2SO_4, filtered, and concentrated under reduced pressure to afford a white solid. The crude solid was either used without further purification or recrystallized as noted below.

***N*-*tert*-Butoxycarbonyl-*N'*-*tert*-butylsulfamide (S5a)**

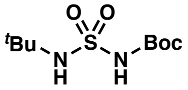

Prepared from chlorosulfonyl isocyanate (1.7 mL, 19.8 mmol), ^{t}BuOH (1.9 mL, 19.8 mmol), pyridine (8.0 mL, 99.3 mmol), and *tert*-butylamine (4.1 mL, 39.4 mmol) following general procedure C. The product was obtained as a white solid (3.32 g, 66% yield)) following recrystallization performed by dissolving the crude solid in a minimal amount of a warm solution of hexanes:EtOAc (1:4) and allowing the solution to slowly cool to room temperature before further cooling at –20 °C.

^{1}H NMR (500 MHz, CD_3CN) δ 8.44 (br s, 1H), 5.56 (br s, 1H), 1.46 (s, 9H), 1.29 (s, 9H).
^{13}C NMR (126 MHz, CD_3CN) δ 151.5, 83.1, 55.2, 29.6, 28.2.
IR (neat) ν 3277 (br), 2973 (w), 1709 (m), 1477 (w), 1454 (w), 1421 (w), 1393 (m), 1369 (m), 1348 (m), 1250 (m), 1206 (w), 1167 (m), 1138 (m), 1041 (w), 1000 (m), 914 (m), 862 (w), 817 (m), 780 (w), 720 (m), 606 (m), 575 (m) cm^{-1}.
HRMS (ESI) m/z: $[M - H]^-$ Calcd for $C_9H_{19}N_2O_4S$ 251.1065; Found 251.1063.

***N-tert*-Butoxycarbonyl-*N'*-(2,2,2-trifluoroethyl)sulfamide (S5b)**

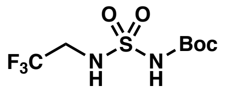

Prepared from chlorosulfonyl isocyanate (1.7 mL, 19.8 mmol), ^{t}BuOH (1.9 mL, 19.8 mmol), pyridine (8.0 mL, 99.3 mmol), and 2,2,2-trifluoroethylamine (3.1 mL, 39.4 mmol) following general procedure C. The product was obtained as a white solid (5.28 g, 96% yield) following workup.

^{1}H NMR (400 MHz, CD_3CN) δ 8.60 (br s, 1H), 6.44 (br s, 1H), 3.76 (qd, J = 9.2, 6.9 Hz, 2H), 1.46 (s, 9H).
^{13}C NMR (126 MHz, CD_3CN) δ 151.2, 125.1 (q, J = 277.7 Hz), 83.9, 45.3 (q, J = 35.2 Hz), 28.1.
^{19}F NMR (376 MHz, CD_3CN) δ –73.16 (t, J = 9.2 Hz).
IR (neat) ν 3290 (br), 2999 (w), 1713 (m), 1478 (w), 1468 (w), 1427 (m), 1396 (w), 1371 (w), 1356 (m), 1299 (w), 1282 (w), 1252 (m), 1141 (m), 1111 (m), 1069 (w), 1032 (w), 970 (m), 921 (w), 848 (w), 835 (m), 820 (m), 778 (w), 761 (w), 731 (m), 667 (m), 589 (m), 545 (m) cm^{-1}.
HRMS (ESI) m/z: $[M - H]^-$ Calcd for $C_7H_{12}F_3N_2O_4S$ 277.0469; Found 277.0457.

***N-tert*-Butoxycarbonyl-*N'*-ethylsulfamide (S5c)**

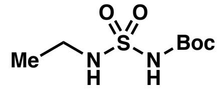

Prepared from chlorosulfonyl isocyanate (1.7 mL, 19.8 mmol), ^{t}BuOH (1.9 mL, 19.8 mmol), pyridine (8.0 mL, 99.3 mmol), and ethylamine (20 mL of 2 M solution in THF, 40.0 mmol) following general procedure C. The product was obtained as a white solid (2.40 g, 54% yield) following recrystallization performed by dissolving the crude solid in a minimal amount of a warm solution of hexanes:EtOAc (1:4) and allowing the solution to slowly cool to room temperature before further cooling at –20 °C.

^{1}H NMR (500 MHz, CD_3CN) δ 8.37 (br s, 1H), 5.65 (br s, 1H), 3.03 (qd, J = 7.3, 6.0 Hz, 2H), 1.46 (s, 9H), 1.13 (t, J = 7.2 Hz, 3H).
^{13}C NMR (126 MHz, CD_3CN) δ 151.5, 83.4, 39.4, 28.2, 14.8.
IR (neat) ν 3286 (br), 3212 (m), 2993 (w), 2938 (w), 2873 (w), 1698 (s), 1427 (s), 1394 (m), 1371 (m), 1341 (s), 1250 (m), 1153 (m), 1128 (s), 1102 (s), 1063 (w), 952 (s), 912 (m), 858 (m), 816 (s), 780 (s), 717 (s), 583 (s) cm^{-1}.
HRMS (ESI) m/z: $[M - H]^-$ Calcd for $C_7H_{15}N_2O_4S$ 223.0752; Found 223.0752.

***N-tert*-Butoxycarbonyl-*N'*-hexylsulfamide (S5d)**

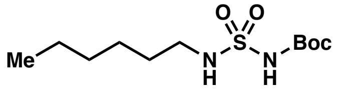

Prepared from chlorosulfonyl isocyanate (1.7 mL, 19.8 mmol), ^{t}BuOH (1.9 mL, 19.8 mmol), pyridine (8.0 mL, 99.3 mmol), and hexylamine (5.2 mL, 39.4 mmol) following general procedure C. The product was obtained as a white solid (3.89 g, 70% yield) following silica gel column chromatography using hexanes:EtOAc (Gradient: 100% hexanes → 9:1 hexanes/EtOAc).

^{1}H NMR (400 MHz, $CDCl_3$) δ 7.07 (br s, 1H), 5.02 (t, J = 6.6 Hz, 1H), 3.06 (dt, J = 6.6, 6.6 Hz, 2H), 1.59–1.54 (m, 2H), 1.50 (s, 9H), 1.36–1.27 (m, 6H), 0.89 (t, J = 6.8 Hz, 3H).
^{13}C NMR (126 MHz, $CDCl_3$) δ 150.2, 83.7, 43.9, 31.2, 29.0, 28.0, 26.2, 22.4, 13.9.
IR (neat) ν 3280 (br), 3219 (br), 2959 (w), 2929 (w), 2861 (w), 1696 (s), 1430 (m), 1395 (m), 1371 (m), 1349 (m), 1252 (m), 1176 (m), 1136 (s), 1077 (m), 1002 (w), 931 (m), 818 (m), 784 (m), 720 (s), 583 (s), 516 (m), 458 (m), 434 (m) cm^{-1}.
TLC R_f = 0.27 in 4:1 hexanes:EtOAc.
HRMS (ESI) m/z: $[M + NH_4]^+$ Calcd for $C_{11}H_{24}N_2O_4S•NH_4$ 298.1795; Found 298.1797.

IV. Preparation and characterization of sulfamides.

General Procedure D: Preparation of sulfamides from sulfamic acid salts with amine nucleophiles.

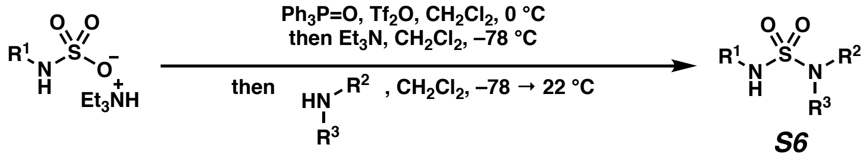

A flame-dried round bottom flask equipped with magnetic stir bar was charged with triphenylphosphine oxide (1.65 equiv) and fitted with a rubber septum. The flask was evacuated and backfilled with N_2. Anhydrous CH_2Cl_2 (0.22 M with respect to amine) was added and the flask was cooled at 0 °C in an ice water bath. Trifluoromethanesulfonic anhydride (1.5 equiv) that had been freshly removed from the glovebox was then added to the cooled solution dropwise via syringe. The reaction was allowed to stir at 0 °C in an ice water bath for 15 minutes. This suspension was treated with a solution of sulfamate salt (1.5 equiv) in CH_2Cl_2 (1.25 M with respect to amine) via cannula transfer. The flask containing sulfamate salt solution was rinsed with CH_2Cl_2 (ca. 5.0 M with respect to amine) to achieve quantitative transfer. The resulting yellow solution was stirred for 15 minutes at 0 °C. During this time, a clean, flame-dried round bottom flask equipped with stir bar was fitted with a rubber septum and subsequently evacuated and backfilled with N_2. This process was repeated two more times. The flask was then charged with Et_3N (3.0 equiv) and CH_2Cl_2 (0.22 M with respect to amine) and the mixture was cooled at –78 °C in an ^{i}PrOH/dry ice bath. The sulfamate salt solution was transferred dropwise to the Et_3N solution via cannula (during which time a yellow to intense red color often developed), rinsing the flask with CH_2Cl_2 (ca. 2.0 M with respect to amine) to achieve quantitative transfer. The resultant solution was stirred at –78 °C for 15 minutes. The amine ((R^2)(R^3)–NH, 1.0 equiv) was then added as a solution in CH_2Cl_2 (1.25 M) to the triethylamine solution via cannula. The amine-containing flask was rinsed with CH_2Cl_2 (ca. 5.0 M with respect to amine) to achieve quantitative transfer. Without removing the cooling bath, the reaction was then stirred overnight, during which time no additional dry ice was added to the bath and the mixture gradually warmed to room temperature.

After stirring overnight, the reaction was diluted with 1 M HCl (ca. 5 mL/mmol of sulfamide) and H_2O (ca. 5 mL/mmol of sulfamide). The biphasic mixture was transferred to a separatory funnel, rinsing the flask with CH_2Cl_2 to achieve quantitative transfer. The organic phase was separated and the aqueous phase was extracted twice more with CH_2Cl_2 (ca. ½ reaction volume). The combined organic phases were dried with $MgSO_4$, filtered, and concentrated under reduced pressure. The crude reaction mixtures were then purified by silica gel flash chromatography by dry loading the samples and eluting with a hexanes:EtOAc solvent system as noted below.

N-*tert*-Butyl-*N'*-isopentylsulfamide (S6c)

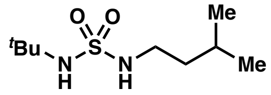

Prepared from triethylammonium *tert*-butylsulfamate (3.8 g, 15.0 mmol) and isoamylamine (1.16 mL, 10.0 mmol) following general procedure D. The product was obtained as a white solid (2.06 g, 93% yield) after silica gel column chromatography using hexanes:EtOAc (9:1 to 4:1).

^{1}H NMR (400 MHz, $CDCl_3$) δ 4.26 (br s, 1H), 4.14 (br s, 1H), 3.04 (dt, *J* = 7.0, 7.0 Hz, 2H), 1.70–1.60 (m, 1H), 1.43 (dt, *J* = 7.0, 7.0 Hz, 2H), 1.34 (s, 9H), 0.91 (d, *J* = 6.6 Hz).
^{13}C NMR (126 MHz, $CDCl_3$) δ 53.8, 41.5, 38.2, 29.8, 29.7, 25.6, 22.3.
IR (neat) ν 3295 (m, br), 2956 (w), 2867 (w), 1466 (w), 1431 (m), 1392 (m), 1364 (w), 1307 (s), 1211 (w), 1170 (w), 1135 (s), 1079 (m), 1042 (w), 1006 (m), 918 (m), 898 (m), 860 (w), 821 (w), 761 (w), 622 (m), 590 (m) cm^{-1}.
TLC R_f = 0.24 in 4:1 hexanes:EtOAc
HRMS (ESI) m/z: [M – H]$^-$ Calcd for $C_9H_{22}N_2O_2S$ 221.1329; Found 221.1332.

N-*tert*-Butyl-*N'*-(3,7-dimethyloct-1-yl)sulfamide (S6h)

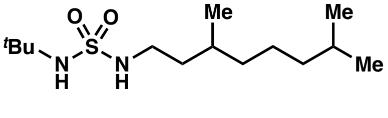

Prepared from triethylammonium *N*-*tert*-butylsulfamate (4.19 g, 16.5 mmol, 1.65 equiv) and 3,7-dimethyloctylamine (**S4h**, 1.57 g, 10.0 mmol, 1.00 equiv) according to general procedure D. The product was isolated as a viscous, colorless oil (2.96 g, 51% yield) after purification on a Teledyne Isco CombiFlashR$_f$ system using a Redi Sep R$_f$ normal phase silica gel column (15:1 hexanes/EtOAc).

^{1}H NMR (400 MHz, $CDCl_3$) δ 4.01 (br s, 1H), 3.92 (t, *J* = 6.7 Hz, 1H), 3.13–2.99 (m, 2H), 1.61–1.47 (m, 3H), 1.36 (s, 9H), 1.32–1.21 (m, 4H), 1.16–1.09 (m, 3H), 0.90 (d, *J* = 6.5 Hz, 3H), 0.87 (d, *J* = 6.6 Hz, 6H).
^{13}C NMR (126 MHz, $CDCl_3$) δ 53.9, 41.4, 39.2, 37.0, 36.5, 30.4, 29.8, 27.9, 24.6, 22.7, 22.6, 19.4.
IR (neat) ν 3276 (br), 2954 (w), 2925 (m), 2869 (w), 1464 (w), 1428 (w), 1391 (w), 1367 (w), 1301 (m), 1230 (w), 1137 (s), 1080 (w), 1041 (w), 988 (m), 899 (w), 765 (w), 610 (m) cm^{-1}.
TLC R_f = 0.29 in 8:2 hexanes/EtOAc.
HRMS (ESI) m/z: [M – H]$^-$ Calcd for $C_{14}H_{31}N_2O_2S$ 291.2106; Found 291.2107.

***N*-(*tert*-Butyl butano-4-yl)-*N'*-*tert*-butylsulfamide (S6t)**

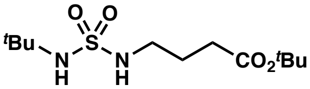

Prepared from triethylammonium *N*-*tert*-butylsulfamate (1.86 g, 7.30 mmol, 1.65 equiv) and *tert*-butyl 4-aminobutyrate (700.0 mg, 4.40 mmol, 1.00 equiv) according to general procedure D. The product was isolated as a white solid (659.4 mg, 51% yield) after purification on a Teledyne Isco CombiFlashR$_f$ system using a Redi Sep R$_f$ normal phase silica gel column (7:3 hexanes/EtOAc).

^{1}H NMR (400 MHz, $CDCl_3$) δ 4.26 (t, *J* = 5.3 Hz, 1H), 4.04 (br s, 1H), 3.09 (dt, *J* = 6.7, 6.7 Hz, 2H), 2.32 (t, *J* = 7.0 Hz, 2H), 1.88–1.83 (m, 2H), 1.45 (s, 9H), 1.35 (s, 9H).
^{13}C NMR (126 MHz, $CDCl_3$) δ 172.5, 80.5, 53.8, 42.6, 32.7, 29.7, 28.0, 24.5.
IR (neat) ν 3303 (br), 3262 (br), 2976 (m), 1704 (s), 1474 (w), 1438 (m), 1389 (m), 1365 (m), 1310 (s), 1296 (s), 1267 (m), 1234 (m), 1207 (m), 1159 (s), 1127 (s), 1092 (s), 1078 (m), 1039 (m), 993 (s), 957 (s), 929 (m), 872 (m), 846 (m), 785 (m), 774 (m), 613 (s), 573 (s) cm^{-1}.
TLC R_f = 0.07 in 8:2 hexanes/EtOAc.
HRMS (ESI) m/z: [M – H]$^-$ Calcd for $C_{12}H_{25}N_2O_4S$ 293.1535; Found 293.1540.

***N-tert*-Butyl-*N'*-(3-(4-trifluoromethyl)phenyl)prop-1-yl)sulfamide (S6u)**

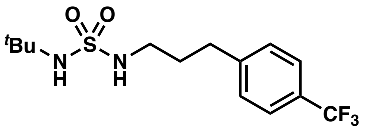

Prepared from triethylammonium *tert*-butylsulfamate (1.90 g, 7.5 mmol) and 3-(4-(trifluoromethyl)phenyl)propyl amine (**S4u**, 1.02 g, 5.0 mmol) following general procedure D. The product was obtained as a white solid (664 mg, 39% yield) after silica gel column chromatography (4:1 hexanes/EtOAc).

^{1}H NMR (400 MHz, $CDCl_3$) δ 7.54 (d, *J* = 8.1 Hz, 2H), 7.30 (d, *J* = 8.1 Hz, 2H), 4.20 (t, *J* = 5.5 Hz, 1H), 4.13 (br s, 1H), 3.07 (dt, *J* = 6.8, 6.8 Hz, 2H), 2.75 (dd, *J* = 7.7, 7.7 Hz, 2H), 1.95–1.87 (m, 2H), 1.33 (s, 9H).
^{13}C NMR (126 MHz, $CDCl_3$, ^{1}H and ^{19}F decoupled) δ 145.2, 128.7, 128.4, 125.4, 124.3, 54.0, 42.6, 32.7, 30.8, 29.7.
^{19}F NMR (376 MHz, $CDCl_3$) δ –62.41.
IR (neat) ν 3292 (br m), 2978 (w), 1672 (w), 1618 (w), 1419 (w), 1395 (w), 1369 (w), 1326 (s), 1300 (m), 1235 (w), 1162 (m), 1141 (s), 1113 (s), 1069 (m), 1047 (w), 1007 (m), 903 (w), 846 (w), 821 (w), 734 (w), 635 (w), 619 (w) cm^{-1}.
TLC R_f = 0.17 in 4:1 hexanes:EtOAc
HRMS (ESI) m/z: $[M + H]^+$ Calcd for $C_{14}H_{21}F_3N_2S_2S$ 339.1349; Found 339.1347.

V. Preparation and characterization of Boc-protected sulfamides.

General Procedure E: Preparation of Boc-protected sulfamides.

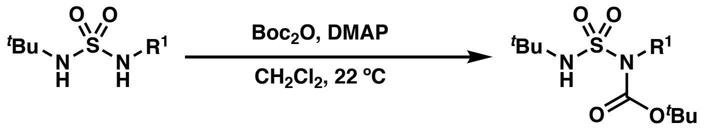

A flame-dried round bottom flask equipped with a stir bar was charged with sulfamide (1.0 equiv), 4-dimethylaminopyridine (DMAP, 1.1 equiv), and di-*tert*-butyl dicarbonate (Boc_2O, 1.3 equiv). The flask was fitted with a rubber septum and evacuated and backfilled with N_2, and charged with CH_2Cl_2 (0.2 M). The resulting solution was stirred at 22 ºC overnight.

After stirring overnight, the reaction was either concentrated under reduced pressure and purified by silica gel chromatography by dry loading samples and eluting with a hexanes/EtOAc solvent system as noted below, or subjected to an aqueous workup and subsequently purified. When an aqueous workup was performed, the reaction was diluted with deionized H_2O (0.2 M) and the biphasic mixture was transferred to a separatory funnel. The organic phase was removed and the aqueous was extracted twice more with CH_2Cl_2 (2 x 0.2 M). The combined organic layers were dried with $MgSO_4$, filtered, and concentrated under reduced pressure. The crude mixture was purified by silica gel chromatography by dry loading samples and eluting with a hexanes/EtOAc solvent system as noted below.

General Procedure F: Preparation of Boc-protected sulfamides from alcohol precursors.

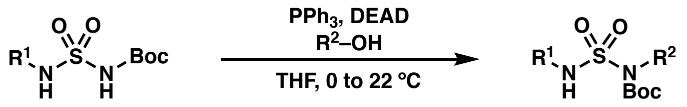

Following a procedure reported by Du Bois and co-workers,[18] a flame-dried round bottom flask equipped with stir bar was charged with *N*-Boc sulfamide **S5** (1.3 equiv) and triphenylphosphine (Ph_3P, 1.3 equiv). The flask was fitted with a rubber septum and evacuated and backfilled with N_2. Anhydrous THF (0.2 M with respect to alcohol) was added and the reaction flask was cooled at 0 °C in an ice water bath. Alcohol (1.0 equiv) was added via syringe followed by dropwise addition of diethylazodicarboxylate (DEAD, 1.3 equiv). Following complete addition of all reagents, the reaction was allowed to slowly warm to room temperature by not adding any more ice to the ice water bath and stirred overnight.

After stirring overnight, the reaction was concentrated under reduced pressure and purified by silica gel chromatography by dry loading samples and eluting with a hexanes/EtOAc solvent system as noted below.

***N-tert*-Butoxycarbonyl-*N*-ethyl-*N'*-hexylsulfamide (S7a)**

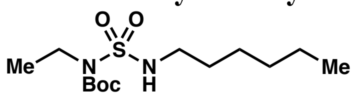

Prepared from sulfamide **S5d** (925 mg, 3.3 mmol) and ethanol (0.15 mL, 2.5 mmol) following general procedure F. The product was obtained as a white solid (711 mg, 92% yield) after silica gel

column chromatography (Gradient: 100% hexanes → 20:1 hexanes/EtOAc).

^{1}H NMR (400 MHz, $CDCl_3$) δ 5.20 (t, J = 6.6 Hz, 1H), 3.72 (q, J = 7.0 Hz, 2H), 2.94 (dt, J = 6.6, 6.6 Hz, 2H), 1.58–1.54 (m, 2H), 1.53 (s, 9H), 1.35–1.28 (m, 6H), 1.24 (t, J = 7.0 Hz, 3H), 0.89 (t, J = 6.8 Hz, 3H).
^{13}C NMR (126 MHz, $CDCl_3$) δ 151.9, 83.7, 43.8, 42.8, 31.2, 28.8, 28.1, 26.3, 22.4, 15.0, 13.9.
IR (neat) ν 3319 (br), 2933 (w), 2860 (w), 1705 (m), 1458 (w), 1424 (w), 1368 (m), 1335 (m), 1280 (m), 1256 (m), 1185 (m), 1138 (s), 1085 (w), 1000 (w), 953 (w), 910 (w), 845 (m), 816 (w), 774 (w), 714 (s), 631 (m), 616 (m), 569 (m), 464 (w) cm^{-1}.
TLC R_f = 0.27 in 9:1 hexanes:EtOAc.
HRMS (ESI) m/z: $[M + Na]^+$ Calcd for $C_{13}H_{28}N_2O_4S$•Na 331.1662; Found 331.1663.

***N-tert*-Butoxycarbonyl-*N'*-hexyl-*N*-(2,2,2-trifluoro)ethylsulfamide (S7b)**

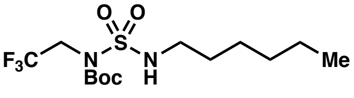

Prepared from sulfamide **S5d** (925 mg, 3.3 mmol) and (2,2,2-trifluoro)ethanol (0.18 mL, 2.5 mmol) following general procedure F. The product was obtained as a white solid (213 mg, 24% yield) after silica gel column chromatography (Gradient: 100% hexanes → 20:1 hexanes/EtOAc).

^{1}H NMR (400 MHz, $CDCl_3$) δ 5.23 (t, J = 6.6 Hz, 1H), 4.31 (q, J = 8.3 Hz, 2H), 3.03 (dt, J = 6.6 Hz, 2H), 1.58–1.54 (m, 2H), 1.54 (s, 9H), 1.36–1.26 (m, 6H), 0.89 (t, J = 6.8 Hz, 3H).
^{13}C NMR (126 MHz, $CDCl_3$) δ 151.0, 123.5 (q, J = 280.3 Hz), 85.5, 47.1 (q, J = 35.7 Hz), 43.9, 31.2, 28.8, 27.8, 26.2, 22.4, 13.9.
^{19}F NMR (376 MHz, $CDCl_3$) δ –70.85 (t, J = 8.4 Hz).
IR (neat) ν 3315 (br), 2933 (w), 2861 (w), 1720 (m), 1423 (w), 1397 (w), 1369 (m), 1344 (m), 1298 (w), 1251 (m), 1140 (s), 1082 (w), 1047 (w), 1027 (m), 957 (w), 850 (w), 830 (w), 794 (w), 775 (w), 706 (m), 677 (w), 642 (m), 576 (m), 530 (w), 463 (w) cm^{-1}.
TLC R_f = 0.25 in 9:1 hexanes:EtOAc.
HRMS (ESI) m/z: $[M + NH_4]^+$ Calcd for $C_{13}H_{25}F_3N_2O_4S$•NH_4 380.1825; Found 380.1832.

***N-tert*-Butoxycarbonyl-*N'*-*tert*-butyl-*N*-neopentylsulfamide (S8a)**

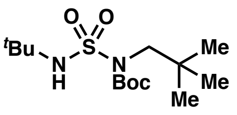

Prepared from *N-tert*-butyl-*N'*-neopentylsulfamide[19] (445 mg, 2.0 mmol), Boc_2O (567 mg, 2.6 mmol), and DMAP (269 mg, 2.2 mmol) in CH_2Cl_2 (10 mL, 0.2 M) following general procedure E. The product was obtained as a white solid (441 mg, 68% yield) after silica gel column chromatography (9:1 hexanes:EtOAc).

^{1}H NMR (400 MHz, $CDCl_3$) δ 5.44 (br s, 1H), 3.54 (s, 2H), 1.52 (s, 9H), 1.26 (s, 9H), 0.97 (s, 9H).
^{13}C NMR (101 MHz, $CDCl_3$) δ 152.7, 83.4, 57.5, 54.8, 33.4, 29.4, 28.1, 28.0.
IR (neat) ν 3312 (br m), 2967 (m), 1703 (s), 1478 (m), 1433 (m), 1392 (m), 1368 (m), 1352 (s), 1285 (m), 1256 (m), 1220 (m), 1156 (s), 1139 (s), 1071 (m), 1041 (w), 1005 (s), 931 (w), 864 (w), 844 (m), 789 (m), 775 (m), 710 (s), 603 (s), 575 (s) cm^{-1}.
TLC R_f = 0.33 in 9:1 hexanes:EtOAc.
HRMS (ESI) m/z: $[M – H]^–$ Calcd for $C_{14}H_{30}N_2O_4S$ 321.1854; Found 321.1852.

***N-tert*-Butoxycarbonyl-*N*-butyl-*N'*-*tert*-butylsulfamide (S8b)**

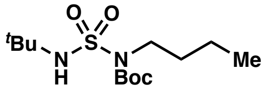

Prepared from sulfamide **S5a** (820 mg, 3.3 mmol) and 1-butanol (0.23 mL, 2.5 mmol) following general procedure F. The product was obtained as a white solid (741 mg, 96% yield) after silica gel column chromatography (Gradient: 100% hexanes → 20:1 hexanes/EtOAc).

^{1}H NMR (400 MHz, $CDCl_3$) δ 5.30 (br s, 1H), 3.64 (dd, J = 7.5, 7.5 Hz, 2H), 1.70–1.62 (m, 2H), 1.52 (s, 9H), 1.37–1.31 (m, 2H), 1.30 (s, 9H), 0.93 (t, J = 7.4 Hz, 3H).
^{13}C NMR (126 MHz, $CDCl_3$) δ 152.3, 83.3, 55.0, 47.3, 31.7, 29.4, 28.1, 19.8, 13.7.
IR (neat) ν 3282 (m), 2956 (m), 2931 (w), 2872 (w), 1703 (s), 1455 (m), 1441 (m), 1397 (m), 1370 (s), 1339 (s), 1294 (m), 1256 (m), 1230 (m), 1208 (w), 1167 (s), 1137 (s), 1046 (m), 1002 (m), 935 (w), 885 (w), 843 (m), 785 (m), 770 (m), 714 (s), 639 (s), 591 (s), 578 (s), 564 (s)cm^{-1}.
TLC R_f = 0.24 in 9:1 hexanes:EtOAc.
HRMS (ESI) m/z: [M – H]$^-$ Calcd for $C_{13}H_{28}N_2O_4S$ 307.1697; Found 307.1694.

***N-tert*-Butoxycarbonyl-*N'*-*tert*-butyl-*N*-isopentylsulfamide (S8c)**

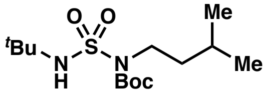

Prepared from sulfamide **S6c** (222.0 mg, 1.00 mmol), Boc_2O (283 mg, 1.30 mmol), and DMAP (122 mg, 1.00 mmol) in CH_2Cl_2 (5 mL, 0.2 M) according to general procedure E. The product was isolated as a white solid (245.0 mg, 76% yield) after purification by Silicycle Silica flash F60 silica gel chromatography (9:1 hexanes/EtOAc).

^{1}H NMR (500 MHz, $CDCl_3$) δ 5.31 (br s, 1H), 3.66–3.63 (m, 2H), 1.60–1.56 (m, 3H), 1.52 (s, 9H), 1.30 (s, 9H), 0.92 (d, J = 5.8 Hz, 6H).
^{13}C NMR (126 MHz, $CDCl_3$) δ 152.2, 83.3, 55.0, 46.0, 38.4, 29.4, 28.1, 25.9, 22.4.
IR (neat) ν 3308 (br), 2962 (w), 2871 (w), 1710 (m), 1470 (w), 1431 (m), 1392 (w), 1348 (m), 1293 (m), 1253 (m), 1146 (s), 1045 (m), 1009 (m), 964 (w), 932 (m), 847 (m), 798 (w), 772 (m), 754 (w), 711 (m), 629 (m), 579 (m) cm^{-1}.
TLC R_f = 0.38 in 8:2 hexanes/EtOAc.
HRMS (ESI) m/z: [M – H]$^-$ Calcd for $C_{14}H_{29}N_2O_4S$ 321.1848; Found 321.1858.

***N-tert*-Butoxycarbonyl-*N'*-*tert*-butyl-*N*-(2-(cyclopentyl)eth-1-yl)sulfamide (S8d)**

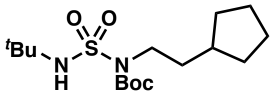

Prepared from *N-tert*-butyl-*N'*-2-cyclopentylethylsulfamide[19] (373 mg, 1.5 mmol), Boc_2O (426 mg, 1.95 mmol), and DMAP (202 mg, 1.65 mmol) in CH_2Cl_2 (7.5 mL, 0.2 M) following general procedure E. The product was obtained as a white solid (494 mg, 94% yield) after silica gel column chromatography (Gradient: 100% hexanes → 9:1 hexanes/EtOAc).

^{1}H NMR (400 MHz, $CDCl_3$) δ 5.31 (br s, 1H), 3.63 (dd, J = 9.7, 7.9 Hz, 2H), 1.81–1.75 (m, 3H), 1.73–1.66 (m, 3H), 1.62–1.57 (m, 3H), 1.52 (s, 9H), 1.29 (s, 9H), 1.16–1.10 (m, 2H).
^{13}C NMR (126 MHz, $CDCl_3$) δ 152.2, 83.3, 55.0, 47.0, 37.4, 35.9, 32.4, 29.4, 28.1, 25.1.
IR (neat) ν 3332 (br w), 2966 (m), 2861(w), 1715 (s), 1453 (w), 1428 (m), 1392 (m), 1366 (m), 1344 (s), 1289 (m) 1259 (m), 1149 (s), 1123 (s), 1041 (m), 1011 (s), 977 (m), 920 (m), 851 (m), 808 (w), 773 (m), 746 (m), 645 (m), 605 (s), 592 (s), 543 (m) cm^{-1}.
TLC R_f = 0.33 in 9:1 hexanes:EtOAc.
HRMS (ESI) m/z: [M + Na]$^+$ Calcd for $C_{16}H_{32}N_2O_4S$•Na 371.1975; Found 371.1969.

N-tert-Butoxycarbonyl-N'-tert-butyl-N-(2-(cyclohexyl)eth-1-yl)sulfamide (S8e)

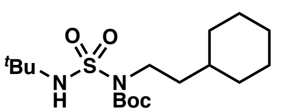

Prepared from sulfamide **S5a** (820 mg, 3.3 mmol) and 2-cyclohexylethanol (0.35 mL, 2.5 mmol) following general procedure F. The product was obtained as a colorless oil (696 mg, 77% yield) after silica gel column chromatography (Gradient: 100% hexanes → 50:1 hexanes/EtOAc).

^{1}H NMR (400 MHz, $CDCl_3$) δ 5.31 (br s, 1H), 3.68–3.64 (m, 2H), 1.74–1.64 (m, 5H), 1.52 (s, 9H), 1.29 (s, 9H), 1.24–1.13 (m, 6H), 0.98–0.89 (m, 2H).
^{13}C NMR (126 MHz, $CDCl_3$) δ 152.1, 83.2, 54.8, 45.6, 36.9, 35.2, 32.9, 29.3, 28.0, 26.3, 26.0.
IR (neat) ν 3339 (br), 2979 (w), 2922 (m), 2851 (w), 1707 (m), 1449 (w), 1425 (m), 1392 (m), 1355 (m), 1310 (w), 1292 (m), 1247 (w), 1138 (s), 986 (m), 966 (m), 914 (w), 850 (m), 808 (w), 774 (w), 713 (s), 604 (m), 582 (m), 55 (w) cm^{-1}.
TLC R_f = 0.51 in 4:1 hexanes:EtOAc.
HRMS (ESI) m/z: $[M – H]^-$ Calcd for $C_{17}H_{34}N_2O_4S$ 361.2167; Found 361.2161.

N-tert-Butoxycarbonyl-N'-tert-butyl-N-((2R)-methyl 4-methylpentano-2-yl)sulfamide (S8f)

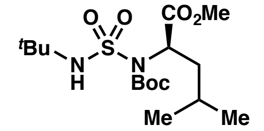

Prepared from *N'-tert*-butyl-*N*-((2*R*)-methyl 4-methylpentano-2-yl)sulfamide [19] (200 mg, 0.713 mmol), Boc_2O (202 mg, 0.927 mmol), and DMAP (95.9 mg, 0.7846 mmol) in CH_2Cl_2 (3.5 mL, 0.2 M) following general procedure E. The product was obtained as a colorless oil (92.3 mg, 34% yield) after silica gel column chromatography (9:1 hexanes/EtOAc) and subsequent alumina column chromatography (95:5 hexanes/EtOAc).

^{1}H NMR (400 MHz, $CDCl_3$) δ 5.32 (br s, 1H), 4.85 (dd, *J* = 7.4, 6.0 Hz, 1H), 3.73 (s, 3H), 2.06 (ddd, *J* = 13.8, 7.6, 5.8 Hz, 1H), 1.86–1.80 (m, 1H), 1.74 (ddd, *J* = 13.8, 7.6, 5.8, 1H), 1.48 (s, 9H), 1.37 (s, 9H), 0.96 (t, *J* = 6.4 Hz, 6H).
^{13}C NMR (126 MHz, $CDCl_3$) δ 170.8, 151.4, 84.2, 58.0, 55.9, 52.1, 40.3, 29.5, 27.9, 25.0, 22.8, 22.0.
IR (neat) ν 3301 (br), 2956 (w), 2871 (w), 1747 (m), 1715 (m), 1435 (w), 1395 (m), 1368 (m), 1351 (m), 1285 (m), 1252 (m), 1232 (m), 1209 (m), 1145 (s), 997 (m), 922 (w), 892 (w), 843 (m), 833 (m), 807 (w), 773 (m), 711 (m), 611 (s), 573 (s) cm^{-1}.
TLC R_f = 0.39 in 8:2 hexanes:EtOAc.
HRMS (ESI) m/z: $[M + Na]^+$ Calcd for $C_{16}H_{32}N_2O_6S{\bullet}Na$ 403.1878; Found 403.1871.

N-tert-Butoxycarbonyl-N'-tert-butyl-N-((S)-2-(1,3-dioxoisoindolin-2-yl)-3-methylbutyl)sulfamide (S8g)

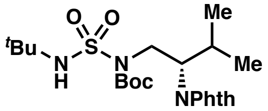

Prepared from sulfamide **S5a** (820 mg, 3.3 mmol) and (*S*)-2-(1-hydroxy-3-methylbutan-2-yl)isoindoline-1,3-dione (583 mg, 2.5 mmol) following general procedure F. The product was obtained as a white solid (981 mg, 84% yield) after silica gel column chromatography (Gradient: 20:1 hexanes:EtOAc → 9:1 hexanes/EtOAc).

^{1}H NMR (400 MHz, $CDCl_3$) δ 7.83–7.82 (m, 2H), 7.67 (dd, *J* = 5.6, 2.9 Hz, 2H), 5.19 (br s, 1H), 4.41 (dd, *J* = 10.9, 14.7 Hz, 1H), 4.17 (ddd, *J* = 2.7, 2.7, 10.9 Hz, 1H), 3.93 (dd, *J* = 2.7, 14.7 Hz,

1H), 2.63–2.54 (m, 1H), 1.54 (s, 9H), 1.22 (s, 9H), 1.12 (d, *J* = 6.6 Hz, 3H), 0.85 (d, *J* = 6.6 Hz, 3H).
^{13}C NMR (126 MHz, $CDCl_3$) δ 168.3, 151.3, 133.4, 131.7, 122.9, 122.6, 83.7, 56.8, 54.6, 46.7, 29.0, 27.8, 27.1, 20.3, 19.8.
IR (neat) ν 3309 (br), 2975 (w), 1771 (w), 1710 (s), 1615 (w), 1468 (m), 1432 (m), 1392 (m), 1370 (s), 1354 (s), 1322 (s), 1290 (m), 1237 (m), 1162 (m), 1144 (s), 1098 (m), 1068 (m), 1042 (m), 1025 (m), 990 (m), 933 (w), 888 (w), 876 (m), 844 (m), 798 (m),772 (m), 716 (s), 709 (s), 681 (m), 601 (s), 581 (s), 551 (m), 531 (m) cm^{-1}.
TLC R_f = 0.10 in 4:1 hexanes:EtOAc.
HRMS (ESI) m/z: $[M – H]^–$ Calcd for $C_{22}H_{33}N_3O_6S$ 466.2017; Found 466.2005.

***N-tert*-Butoxycarbonyl-*N'-tert*-butyl-*N*-(3,7-dimethyloct-1-yl)sulfamide (S8h)**

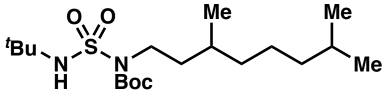

Prepared from sulfamide **S6h** (500 mg, 1.71 mmol) Boc_2O (485 mg, 2.22 mmol), and DMAP (229 mg, 1.88 mmol) in CH_2Cl_2 (8.5 mL, 0.2 M) following general procedure E. The product was isolated as a colorless oil (635 mg, 95% yield) after purification on a Teledyne Isco CombiFlashR_f system using a Redi Sep R_f normal phase silica gel column (9:1 hexanes/EtOAc).

^{1}H NMR (400 MHz, $CDCl_3$) δ 5.30 (br s, 1H), 3.69–3.56 (m, 2H), 1.73–1.66 (m, 1H), 1.50 (s, 9H), 1.48–1.37 (m, 4H), 1.28 (s, 9H), 1.27–1.20 (m, 2H), 1.13–1.08 (m, 3H), 0.88 (d, *J* = 6.4 Hz, 3H), 0.83 (d, *J* = 6.7 Hz, 6H).
^{13}C NMR (126 MHz, $CDCl_3$) δ 152.2, 83.3, 54.9, 45.9, 39.1, 37.0, 36.5, 30.7, 29.4, 28.1, 27.9, 24.6, 22.6, 22.5, 19.4.
IR (neat) ν 3319 (br), 2955 (w), 2927 (w), 2869 (w), 1707 (m), 1459 (w), 1426 (w), 1392 (m), 1353 (m), 1291 (m), 1255 (w), 1137 (s), 986 (m), 848 (w), 804 (w), 773 (w), 713 (m), 636 (m), 606 (m), 578 (m) cm$^{–1}$.
TLC R_f = 0.56 in 8:2 hexanes/EtOAc.
HRMS (ESI) m/z: $[M – H]^–$ Calcd for $C_{19}H_{39}N_2O_4S$ 391.2630; Found 391.2636.

***N-tert*-Butoxycarbonyl-*N'-tert*-butyl-*N*-pentylsulfamide (S8i)**

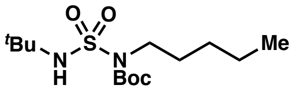

Prepared from *N-tert*-butyl-*N'*-pentylsulfamide[19] (1.00 g, 4.49 mmol) Boc_2O (1.27 g, 5.84 mmol), and DMAP (602 mg, 4.92 mmol) in CH_2Cl_2 (21 mL, 0.2 M) following general procedure E. The product was isolated as a white solid (1.17 g, 80% yield) after purification on a Teledyne Isco CombiFlashR_f system using a Redi Sep R_f normal phase silica gel column (9:1 hexanes/EtOAc).

^{1}H NMR (400 MHz, $CDCl_3$) δ 5.30 (br s, 1H), 3.63 (dd, *J* = 7.6, 7.6 Hz, 2H), 1.72–1.64 (m, 2H), 1.53 (s, 9H), 1.36–1.30 (m, 4H), 1.30 (s, 9H), 0.90 (t, *J* = 6.9 Hz, 3H).
^{13}C NMR (126 MHz, $CDCl_3$) δ 152.3, 83.3, 55.0, 47.5, 29.4, 29.3, 28.8, 28.1, 22.2, 13.9.
IR (neat) ν 3293 (br), 2969 (m), 2934 (m), 2861 (w), 1702 (s), 1440 (m), 1396 (m), 1382 (m), 1368 (m), 1342 (s), 1306 (m), 1278 (m), 1246 (m), 1209 (m), 1165 (s), 1136 (s), 1045 (m), 1000 (m), 935 (m), 867 (w), 843 (m), 794 (m), 769 (m), 713 (s), 640 (s), 590 (m), 564 (s) cm$^{–1}$.
TLC R_f = 0.57 in 8:2 hexanes/EtOAc.
HRMS (ESI) m/z: $[M + Na]^+$ Calcd for $C_{14}H_{30}N_2O_4S{\bullet}Na$ 345.1824; Found 345.1819.

N-tert-Butoxycarbonyl-N'-tert-butyl-N-hexylsulfamide (S8j)

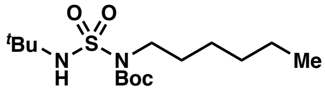

Prepared from *N-tert*-butyl-*N'*-hexylsulfamide[19] (1.18 g, 5.0 mmol), Boc_2O (1.40 g, 6.5 mmol), and DMAP (671 mg, 5.5 mmol) in CH_2Cl_2 (25 mL, 0.2 M) following general procedure E. The product was obtained as a white solid (1.37 g, 82% yield) after silica gel column chromatography (20:1 hexanes/EtOAc).

^{1}H NMR (400 MHz, $CDCl_3$) δ 5.30 (br s, 1H), 3.63 (dd, J = 7.6, 7.6 Hz, 2H), 1.71–1.64 (m, 2H), 1.52 (s, 9H), 1.30 (s, 9H), 1.33–1.26 (m, 6H), 0.87 (t, J = 6.7 Hz, 3H).
^{13}C NMR (101 MHz, $CDCl_3$) δ 152.2, 83.3, 54.9, 47.5, 31.3, 29.5, 29.4, 28.1, 26.2, 22.5, 13.9.
IR (neat) ν 3343 (br w), 2960 (w), 2932 (w), 2860 (w), 1705 (m), 1457 (w), 1425 (w), 1392 (m), 1368 (m), 1353 (s), 1294 (m), 1256 (m), 1164 (s), 1138 (s), 1042 (w), 982 (m), 848 (w), 774 (w), 736 (s), 714 (s), 637 (m), 606 (m), 578 (m) cm^{-1}.
TLC R_f = 0.34 in 9:1 hexanes:EtOAc.
HRMS (ESI) m/z: [M – H]$^-$ Calcd for $C_{15}H_{32}N_2O_4S$ 335.2010; Found 335.2003.

N-tert-Butyoxycarbonyl-N'-tert-butyl-N-(methyl hexano-6-yl)sulfamide (S7k)

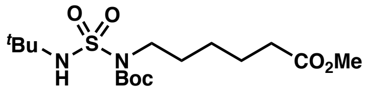

Prepared from *N'-tert*-butyl-*N*-(methyl hexano-6-yl)sulfamide[19] (500 mg, 1.78 mmol), Boc_2O (505 mg, 2.31 mmol), and DMAP (239 mg, 1.96 mmol) in CH_2Cl_2 (9.0 mL, 0.2 M) following general procedure E. The product was isolated as a colorless oil (390 mg, 57% yield) after purification on a Teledyne Isco CombiFlashR_f system using a Redi Sep R_f normal phase silica gel column (8:2 hexanes/EtOAc).

^{1}H NMR (400 MHz, $CDCl_3$) δ 5.29 (br s, 1H), 3.66 (s, 3H), 3.66–3.62 (m, 2H), 2.32 (t, J = 7.5 Hz, 2H), 1.74–1.62 (m, 4H), 1.53 (s, 9H), 1.39–1.33 (m, 2H), 1.30 (s, 9H).
^{13}C NMR (126 MHz, $CDCl_3$) δ 173.9, 152.2, 83.5, 55.0, 51.4, 47.2, 33.9, 29.4, 29.2, 28.1, 26.1, 24.5.
IR (neat) ν 3312 (br), 2974 (w), 1707 (m), 1426 (w), 1392 (m), 1352 (s), 1293 (w), 1253 (w), 1137 (s), 1040 (w), 987 (m), 918 (w), 847 (m), 773 (w), 712 (s), 635 (m), 577 (m) cm^{-1}.
TLC R_f = 0.25 in 8:2 hexanes/EtOAc.
HRMS (ESI) m/z: [M – H]$^-$ Calcd for $C_{16}H_{31}N_2O_6S$ 379.1902; Found 379.1901.

N-Acetyl-N'-tert-butyl-N-hexylsulfamide (S8l)

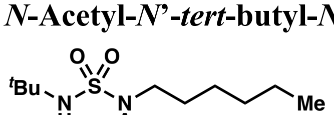

A flame-dried round bottom flask equipped with a stir bar was charged with *N-tert*-butyl-*N'*-hexylsulfamide[19] (595 mg, 2.5 mmol, 1.0 equiv) and DMAP (336 mg, 2.8 mmol, 1.1 equiv). The flask was fitted with a rubber septum and evacuated and backfilled with N_2, and CH_2Cl_2 (13 mL, 0.2 M) was added followed by acetic anhydride (Ac_2O, 0.5 mL, 5.0 mmol, 2.0 equiv). The resulting solution was stirred at 22 ºC overnight.

After stirring overnight, the reaction was diluted with deionized H_2O (0.2 M) and the biphasic mixture was transferred to a separatory funnel. The organic phase was removed and the aqueous was extracted twice more with CH_2Cl_2 (2 x 0.2 M). The combined organic layers were dried with $MgSO_4$, filtered, and concentrated under reduced pressure. The product was obtained as a

colorless oil (678 mg, 97% yield) after silica gel column chromatography (20:1 hexanes/EtOAc).

^{1}H NMR (400 MHz, $CDCl_3$) δ 5.41 (br s, 1H), 3.67 (dd, *J* = 7.9, 7.9 Hz, 2H), 2.28 (s, 3H), 1.76–1.69 (m, 2H), 1.35–1.30 (m, 6H), 1.29 (s, 9H), 0.88 (t, *J* = 6.7 Hz, 3H).
^{13}C NMR (101 MHz, $CDCl_3$) δ 171.0, 55.2, 47.9, 31.2, 29.8, 29.3, 26.3, 23.7, 22.4, 13.8.
IR (neat) ν 3290 (br), 2958 (w), 2931 (w), 2859 (w), 1669 (m), 1427 (m), 1393 (m), 1348 (s), 1258 (m), 1232 (m), 1205 (m), 1151 (s), 1092 (m), 1039 (m), 1000 (m), 946 (m), 848 (m), 759 (w), 727 (w), 696 (m), 667 (m), 598 (s), 582 (s), 546 (s) cm^{-1}.
TLC R_f = 0.22 in 4:1 hexanes:EtOAc.
HRMS (ESI) m/z: $[M – H]^-$ Calcd for $C_{12}H_{26}N_2O_3S$ 277.1591; Found 277.1584.

N-tert-Butyl-_N'_-hexyl-_N'_-(2,2,2-trifluoroethyl)sulfamide (S8m)

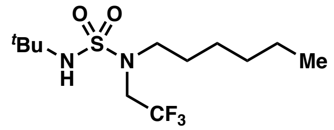

Prepared from *N*-(*tert*-butyl)-*N'*-(2,2,2-trifluoroethyl)sulfamide[11] (761 mg, 3.3 mmol) and 1-hexanol (0.3 mL, 2.5 mmol) following general procedure F. The product was obtained as a colorless oil (575 mg, 72% yield) after silica gel column chromatography (Gradient: 100% hexanes → 50:1 hexanes/EtOAc).

^{1}H NMR (400 MHz, $CDCl_3$) δ 3.98 (br s, 1H), 3.80 (q, *J* = 9.0 Hz, 2H), 3.27 (dd, *J* = 8.0, 8.0 Hz, 2H), 1.68–1.60 (m, 2H), 1.34 (s, 9H), 1.31–1.23 (m, 6H), 0.89 (t, *J* = 6.7 Hz, 3H).
^{13}C NMR (126 MHz, $CDCl_3$) δ 124.5 (q, *J* = 280.4 Hz), 54.7, 49.3, 48.0 (q, *J* = 34.2 Hz), 31.3, 29.5, 26.9, 26.3, 22.4, 13.8.
^{19}F NMR (376 MHz, $CDCl_3$) δ –69.49 (t, *J* = 9.0 Hz).
IR (neat) ν 3286 (br), 2959 (w), 2932 (w), 2861 (w), 1780 (w), 1468 (w), 1428 (w), 1393 (w), 1369 (w), 1321 (m), 1273 (m), 1233 (m), 1141 (s), 1040 (m), 986 (m), 867 (m), 832 (w), 771 (m), 725 (w), 665 (m), 607 (m), 562 (m) cm^{-1}.
TLC R_f = 0.24 in 9:1 hexanes:EtOAc.
HRMS (ESI) m/z: $[M – H]^-$ Calcd for $C_{12}H_{25}F_3N_2O_2S$ 317.1516; Found 317.1510.

N'-tert-Butyl-_N_-trifluoroacetyl-_N_-pentylsulfamide (S8n)

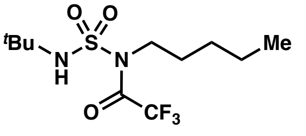

A flame-dried round bottom flask equipped with stir bar was charged with *N-tert*-butyl-*N'*-pentylsulfamide[19] (150 mg, 0.67 mmol, 1.0 equiv) and DMAP (90.7 mg, 0.74 mmol, 1.1 equiv). The flask was fitted with a rubber septum and evacuated and backfilled with N_2. CH_2Cl_2 (3.3 mL, 0.2 M) was added followed by triluoroacetic anhydride (TFAA, 0.12 mL, 0.87 mmol, 1.3 equiv). The resulting solution was stirred at 22 ºC overnight. After stirring overnight, the solution was concentrated under reduced pressure. The product was isolated as a colorless oil (0.67 mmol scale, 129.3 mg, 60% yield) after purification on a Teledyne Isco CombiFlashR$_f$ system using a Redi Sep R$_f$ normal phase silica gel column (9:1 hexanes/EtOAc).

^{1}H NMR (400 MHz, $CDCl_3$) δ 5.54 (br s, 1H), 3.79–3.74 (m, 2H), 1.85–1.77 (m, 2H), 1.39–1.34 (m, 4H), 1.31 (s, 9H), 0.91 (t, *J* = 6.9 Hz, 3H).
^{13}C NMR (126 MHz, $CDCl_3$) δ 156.5 (q, *J* = 38.3 Hz), 115.5 (q, *J* = 288.9 Hz), 56.2, 47.9, 30.1, 29.3, 28.6, 22.0, 13.8.
^{19}F NMR (376 MHz, $CDCl_3$) δ –70.70.

IR (neat) ν 3310 (br), 2960 (w), 2875 (w), 1703 (m), 1431 (m), 1396 (m), 1364 (m), 1286 (w), 1232 (m), 1208 (m), 1168 (s), 1148 (s), 1064 (s), 862 (w), 792 (w), 758 (m), 697 (m), 684 (m), 640 (w), 613 (s), 580 (m), 528 (m) cm^{-1}.
TLC R_f = 0.57 in 8:2 hexanes/EtOAc.
HRMS (ESI) m/z: $[M-H]^-$ Calcd for $C_{11}H_{20}F_3N_2O_3S$ 317.1146; Found 317.1151.

***N*-*tert*-Butoxycarbonyl-*N'*-(2,2,2-trifluoroethyl)-*N*-hexylsulfamide (S8o)**

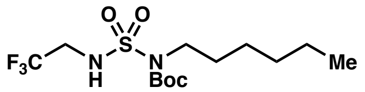

Prepared from sulfamide **S5b** (1.8 g, 6.5 mmol) and 1-hexanol (0.63 mL, 5.0 mmol) following general procedure F. The product was obtained as a colorless oil (1.59 g, 88% yield) after silica gel column chromatography (hexanes/EtOAc (20:1).

^{1}H NMR (400 MHz, $CDCl_3$) δ 5.79 (t, J = 6.5 Hz, 1H), 3.70–3.61 (m, 4H), 1.65–1.59 (m, 2H), 1.52 (s, 9H), 1.32–1.29 (m, 6H), 0.88 (t, J = 6.7 Hz, 3H).
^{13}C NMR (126 MHz, $CDCl_3$) δ 151.8, 123.4 (q, J = 279.0 Hz), 84.4, 48.2, 45.1, (q, J = 35.3 Hz), 31.2, 29.6, 27.8, 26.1, 22.4, 13.9.
^{19}F NMR (376 MHz, $CDCl_3$) δ –72.10 (t, J = 8.9 Hz).
IR (neat) ν 3302 (br w), 2933 (w), 2861 (w), 1705 (m), 1458 (w), 1369 (m), 1277 (m), 1256 (m), 1139 (s), 1047 (w), 963 (m), 832 (m), 773 (w), 718 (s), 664 (m), 630 (m), 581 (m), 549 (m) cm^{-1}.
TLC R_f = 0.26 in 9:1 hexanes:EtOAc.
HRMS (ESI) m/z: $[M-H]^-$ Calcd for $C_{13}H_{25}F_3N_2O_4S$ 361.1414; Found 361.1405.

***N*-*tert*-Butyl-*N'*-4-methylpentyl-*N'*-(2,2,2-trifluoroethyl)sulfamide (S8p)**

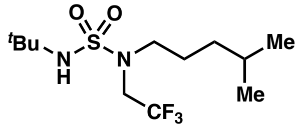

Prepared from *N*-(*tert*-butyl)-*N'*-(2,2,2-trifluoroethyl)sulfamide[11] (761 mg, 3.3 mmol) and 4-methylpentanol (255 mg, 2.5 mmol) following general procedure F. The product was obtained as a colorless oil (726 mg, 91% yield) after silica gel column chromatography (Gradient: 100% hexanes → 20:1 hexanes/EtOAc).

^{1}H NMR (400 MHz, $CDCl_3$) δ 3.96 (br s, 1H), 3.81 (q, J = 9.0 Hz, 2H), 3.26 (dd, J = 8.0, 8.0 Hz, 2H), 1.69–1.58 (m, 3H), 1.34 (s, 9H), 1.18–1.13 (m, 2H), 0.89 (d, J = 6.7 Hz, 6H).
^{13}C NMR (126 MHz, $CDCl_3$) δ 124.5 (q, J = 280.6 Hz), 54.7, 49.5, 48.0 (q, J = 34.6 Hz), 35.7, 29.5, 27.6, 24.8, 22.4.
^{19}F NMR (376 MHz, $CDCl_3$) δ –69.50 (t, J = 8.8 Hz).
IR (neat) ν 3285 (br), 2958 (w), 2873 (w), 1469 (w), 1429 (w), 1393 (w), 1368 (w), 1321 (m), 1274 (m), 1234 (w), 1141 (s), 1041 (m), 985 (s), 929 (w), 904 (w), 871 (m), 831 (w), 770 (m), 731 (m), 664 (m), 607 (m), 562 (m) cm^{-1}.
TLC R_f = 0.30 in 9:1 hexanes:EtOAc.
HRMS (ESI) m/z: $[M-H]^-$ Calcd for $C_{12}H_{25}F_3N_2O_2S$ 317.1516; Found 317.1508.

***N*-*tert*-Butoxycarbonyl-*N'*-*tert*-butyl-*N*-4-methylpentylsulfamide (S8q)**

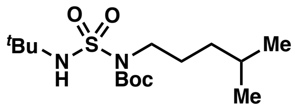

Prepared from sulfamide **S5a** (820 mg, 3.3 mmol) and 4-methylpentanol (255 mg, 2.5 mmol) following general procedure F The product was obtained as a white solid (794 mg, 94% yield) after silica gel column chromatography (Gradient: 100% hexanes → 20:1 hexanes/EtOAc).

^{1}H NMR (400 MHz, $CDCl_3$) δ 5.29 (br s, 1H), 3.60 (dd, *J* = 7.7, 7.7 Hz, 2H), 1.71–1.63 (m, 3H), 1.51 (s, 9H), 1.28 (s, 9H), 1.20–1.14 (m, 2H), 0.87 (d, *J* = 6.6 Hz, 6H).
^{13}C NMR (126 MHz, $CDCl_3$) δ 152.2, 83.3, 55.0, 47.7, 35.7, 29.4, 28.1, 27.5, 27.4, 22.5.
IR (neat) ν 3296 (m), 2971 (m), 2871 (w), 1702 (s), 1457 (m), 1442 (m), 1396 (m), 1380 (m), 1369 (m), 1347 (s), 1322 (s), 1283 (m), 1250 (m), 1166 (s), 1137 (s), 1044 (m), 996 (m), 936 (w), 866 (w), 843 (m), 791 (m), 770 (m), 711 (s), 641 (s), 591 (s), 565 (s) cm^{-1}.
TLC R_f = 0.32 in 9:1 hexanes:EtOAc.
HRMS (ESI) m/z: [M – H]$^-$ Calcd for $C_{15}H_{32}N_2O_4S$ 335.2010; Found 335.2005.

***N-tert*-Butoxycarbonyl-*N*-4-methylpentyl-*N'*-(2,2,2-trifluoroethyl)sulfamide (S8r)**

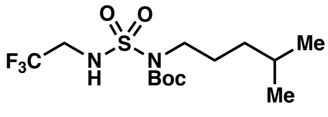

Prepared from sulfamide **S5b** (820 mg, 3.3 mmol) and 4-methylpentanol (255 mg, 2.5 mmol) following general procedure F The product was obtained as a pale yellow oil (693 mg, 76% yield) after silica gel column chromatography (Gradient: 100% hexanes → 20:1 hexanes/EtOAc).

^{1}H NMR (400 MHz, $CDCl_3$) δ 5.82 (t, *J* = 7.2 Hz, 1H), 3.71–3.59 (m, 4H), 1.68–1.58 (m, 3H), 1.52 (s, 9H), 1.21–1.15 (m, 2H), 0.89 (d, *J* = 6.6 Hz, 6H).
^{13}C NMR (126 MHz, $CDCl_3$) δ 151.8, 123.4 (q, *J* = 278.6 Hz), 84.3, 48.3, 45.0 (q, *J* = 35.4 Hz), 35.5, 27.8, 27.4, 27.4, 22.3.
^{19}F NMR (376 MHz, $CDCl_3$) δ –71.56 (t, *J* = 8.8 Hz).
IR (neat) ν 3302 (br), 2956 (w), 2872 (w), 1704 (m), 1458 (w), 1368 (m), 1278 (w), 1139 (s), 1049 (w), 963 (m), 832 (m), 791 (m), 773 (m), 719 (w), 664 (m), 630 (m), 582 (m), 549 (m) cm^{-1}.
TLC R_f = 0.46 in 4:1 hexanes:EtOAc.
HRMS (ESI) m/z: [M – H]$^-$ Calcd for $C_{13}H_{25}F_3N_2O_4S$ 361.1414; Found 361.1407.

***N-tert*-Butoxycarbonyl-*N'*-*tert*-butyl-*N*-(*tert*-butyl butano-4-yl)sulfamide (S8t)**

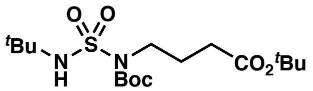

Prepared from sulfamide **S6s (**642 mg, 2.18 mmol), Boc_2O (619 mg, 2.83 mmol), and DMAP (293 mg, 2.40 mmol) in CH_2Cl_2 (11 mL, 0.2 M) following general procedure E. The product was isolated as a colorless oil (651 mg, 76% yield) after purification on a Teledyne Isco CombiFlashR$_f$ system using a Redi Sep R$_f$ normal phase silica gel column (9:1 hexanes/EtOAc).

^{1}H NMR (500 MHz, $CDCl_3$) δ 5.29 (br s, 1H), 3.71–3.68 (m, 2H), 2.28 (t, *J* = 7.5 Hz, 2H), 1.98–1.92 (m, 2H), 1.53 (s, 9H), 1.44 (s, 9H), 1.30 (s, 9H).
^{13}C NMR (101 MHz, $CDCl_3$) δ 171.8, 151.9, 83.4, 80.0, 54.8, 46.5, 32.3, 29.2, 27.9, 27.8, 24.8.
IR (neat) ν 3321 (br), 2970 (w), 1706 (m), 1476 (w), 1453 (w), 1432 (w), 1394 (m), 1367 (m), 1346 (s), 1286 (m), 1258 (m), 1139 (s), 1098 (m), 1041 (w), 994 (m), 946 (w), 864 (w), 847 (m), 794 (w), 774 (m), 753 (w), 735 (w), 710 (m), 631 (m), 591 (m), 575 (m) cm^{-1}.
TLC R_f = 0.47 in 8:2 hexanes/EtOAc.
HRMS (ESI) m/z: [M – H]$^-$ Calcd for $C_{17}H_{33}N_2O_6S$ 393.2059; Found 393.2056.

N-tert-Butoxycarbonyl-N'-tert-butyl-N-(3-((4-trifluoromethyl)phenyl)prop-1-yl)sulfamide (S8u)

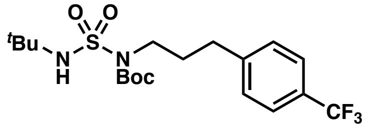

Prepared from sulfamide **S6u** (664 mg, 1.9 mmol), Boc_2O (539 mg, 2.5 mmol), and DMAP (262 mg, 2.1 mmol) in CH_2Cl_2 (10 mL, 0.2 M) following general procedure E. The product was obtained as a white solid (742 mg, 86% yield) after silica gel column chromatography (9:1 hexanes/EtOAc).

^{1}H NMR (400 MHz, $CDCl_3$) δ 7.54 (d, J = 7.9 Hz, 2H), 7.31 (d, J = 7.9 Hz, 2H), 5.30 (br s, 1H), 3.69 (t, J = 7.5 Hz, 2H), 2.72 (t, J = 7.7 Hz, 2H), 2.07–1.99 (m, 2H), 1.48 (s, 9H), 1.30 (s, 9H).
^{13}C NMR (126 MHz, $CDCl_3$) δ 152.0, 145.4, 128.6, 128.2 (q, J = 32.3 Hz), 125.2 (q, J = 3.7 Hz), 124.3 (q, J = 271.7 Hz), 83.6, 55.1, 46.8, 32.7, 30.7, 29.4, 28.0.
^{19}F NMR (376 MHz, $CDCl_3$) δ –62.38.
IR (neat) ν 3296 (br w), 2977 (w), 1718 (m), 1618 (w), 1477 (w), 1462 (w), 1433 (w), 1395 (w), 1381 (w), 1369 (m), 1348 (m), 1328 (s), 1302 (m), 1270 (m), 1253 (m), 1160 (s), 1138 (s), 1112 (s), 1067 (s) 1020 (m), 991 (m), 848 (m), 838 (m), 824 (m), 792 (m), 775 (m), 717 (s), 680 (w), 624 (s), 608 (m), 595 (m), 577 (s) cm^{-1}.
TLC R_f = 0.24 in 9:1 hexanes:EtOAc.
HRMS (ESI) m/z: $[M + Na]^+$ Calcd for $C_{19}H_{29}F_3N_2O_4S$•Na 461.1692; Found 461.1691.

N-tert-Butoxycarbonyl-N'-(2,2,2-trfiluoroethyl)-N-pentylsulfamide (S8v)

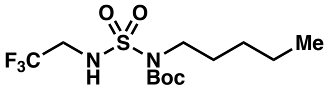

Prepared from sulfamide **S5b** (1.01 g, 3.65 mmol) and 1-pentanol (0.30 mL, 2.80 mmol) following general procedure F. The product was obtained as a colorless oil (947 mg, 97% yield) after purification on a Teledyne Isco CombiFlashRf system using a Redi Sep R_f normal phase silica gel column (9:1 hexanes/EtOAc).

^{1}H NMR (400 MHz, $CDCl_3$) δ 5.78 (t, J = 6.9 Hz, 1H), 3.71–3.61 (m, 4H), 1.68–1.60 (m, 2H), 1.53 (s, 9H), 1.39–1.24 (m, 4H), 0.91 (t, J = 6.9 Hz, 3H).
^{13}C NMR (126 MHz, $CDCl_3$) δ 152.0, 123.7 (q, J = 278.1 Hz), 84.5, 48.4, 45.2 (q, J = 36.2 Hz), 29.5, 28.8, 28.0, 22.3, 14.0.
^{19}F NMR (376 MHz, $CDCl_3$) δ –72.13 (t, J = 8.7 Hz).
IR (neat) ν 3300 (br), 2961 (w), 2934 (w), 2873 (w), 1704 (m), 1457 (w), 1368 (m), 1276 (m), 1139 (s), 1044 (w), 1017 (w), 961 (m), 912 (w), 832 (m), 799 (m), 773 (w), 718 (s), 664 (m), 629 (m), 580 (m), 548 (m) cm^{-1}.
TLC R_f = 0.21 in 8:2 hexanes/EtOAc.
HRMS (ESI) m/z: $[M + Na]^+$ Calcd for $C_{12}H_{23}F_3N_2O_4S$•Na 371.1228; Found 371.1212.

N-tert-Butoxycarbonyl-N'-ethyl-N-pentylsulfamide (S8w)

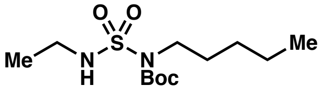

Prepared from sulfamide **S5c** (729 mg, 3.25 mmol) and 1-pentanol (0.27 mL, 2.50 mmol) following general procedure F. The product was obtained as a colorless oil (520 mg, 71% yield) after purification on a Teledyne Isco CombiFlashRf system using a Redi Sep R_f normal phase silica gel column (9:1 hexanes:EtOAc).

^{1}H NMR (400 MHz, $CDCl_3$) δ 5.19 (t, *J* = 6.0 Hz, 1H), 3.65–3.61 (m, 2H), 3.06–2.99 (m, 2H), 1.68–1.61 (m, 2H), 1.52 (s, 9H), 1.36–1.26 (m, 4H), 1.19 (t, *J* = 7.3 Hz, 3H), 0.90 (t, *J* = 6.8 Hz, 3H).
^{13}C NMR (126 MHz, $CDCl_3$) δ 151.8, 83.4, 47.4, 38.6, 29.1, 28.4, 27.7, 21.9, 14.1, 13.6.
IR (neat) ν 3311 (br), 2959 (w), 2933 (w), 2872 (w), 1703 (m), 1455 (w), 1424 (w), 1359 (m), 1279 (m), 1257 (m), 1136 (s), 1063 (m), 1044 (w), 1016 (w), 949 (w), 845 (m), 798 (w), 773 (m), 714 (s), 630 (m), 571 (s) cm^{-1}.
TLC R_f = 0.43 in 8:2 hexanes/EtOAc.
HRMS (ESI) m/z: $[M + Na]^+$ Calcd for $C_{12}H_{26}N_2O_4S$•Na 317.1511; Found 317.1498.

VI. Preparation and characterization of *N*-chlorosulfamides.

General Procedure G: Preparation of *N*-Chlorosulfamides with Trichloroisocyanuric acid

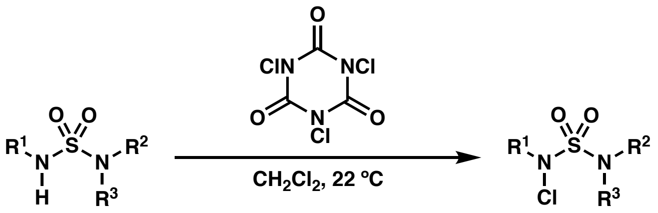

A flame-dried round bottom flask equipped with stir bar was charged with sulfamide (1.0 equiv) and trichloroisocyanuric acid (TCICA, 1.2 equiv). The flask was evacuated and backfilled with N_2. Anhydrous CH_2Cl_2 (0.2 M) was then added via syringe. The suspension was stirred at 21 ºC until complete consumption of the starting material as evidenced by TLC.

The suspension was quenched by addition of H_2O (~0.2 M) and the biphasic mixture was transferred to a separatory funnel rinsing the flask with CH_2Cl_2 (ca. 5 mL) to ensure quantitative transfer. The organic phase was separated and the aqueous phase was extracted with CH_2Cl_2 (2 x ca. 5 mL/mmol sulfamide). The combined organic extracts were dried over $MgSO_4$, filtered, and concentrated under reduced pressure. The crude reaction mixtures were then purified by flash column chromatography on silica gel or florisil eluting with a hexanes/EtOAc solvent system as noted below.

General Procedure H: Preparation of *N*-Chlorosulfamides with *tert*-butyl hypochlorite

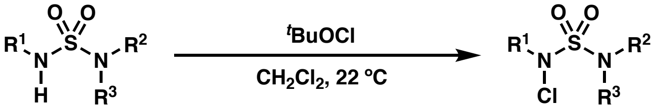

A flame-dried round bottom flask equipped with stir bar was charged with sulfamide (1.0 equiv). The flask was evacuated and backfilled with N_2. Anhydrous CH_2Cl_2 (0.2 M) was then added via syringe. To the stirring solution was added neat *tert*-butyl hypochlorite (^{t}BuOCl, equiv indicated below) in portions via syringe until complete consumption of the starting material as evidenced by TLC.

Upon complete consumption of the starting material as indicated by TLC, all volatiles were removed under reduced pressure to afford the pure compound or crude mixtures to be purified as indicated below.

N-tert-Butoxycarbonyl-*N'*-chloro-*N*-ethyl-*N'*-hexylsulfamide (5a)

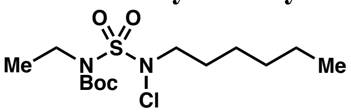

Prepared from sulfamide **S7a** (308 mg, 1.0 mmol) and TCICA (279 mg, 1.2 mmol) in CH_2Cl_2 (5 mL) following general procedure G. The product was obtained as a colorless oil (326 mg, 95% yield) after silica gel column chromatography (Gradient: 100% hexanes → 20:1 hexanes/EtOAc).

^{1}H NMR (400 MHz, $CDCl_3$) δ 3.84 (q, *J* = 7.0 Hz, 2H), 3.61 (dd, *J* = 7.1 Hz, 2H), 1.74–1.67 (m, 2), 1.53 (s, 9H), 1.38–1.32 (m, 6H), 1.30 (t, *J* = 7.0 Hz, 3H), 0.89 (t, *J* = 6.8 Hz, 3H).
^{13}C NMR (126 MHz, $CDCl_3$) δ 150.5, 84.4, 58.3, 45.9, 31.3, 28.0, 27.5, 25.7, 22.5, 15.0, 14.0.
IR (neat) ν 2934 (w), 2860 (w), 1731 (m), 1458 (w), 1383 (m), 1368 (m), 1331 (w), 1282 (m), 1254 (m), 1185 (m), 1137 (s), 1087 (w), 1041 (w), 997 (m), 954 (m), 844 (m), 770 (m), 720 (m), 646 (m), 590 (m), 559 (m), 465 (w) cm^{-1}.
TLC R_f = 0.50 in 9:1 hexanes:EtOAc.
HRMS (ESI) m/z: $[M + Na]^+$ Calcd for $C_{13}H_{27}ClN_2O_4S$•Na 365.1272; Found 365.1274.

***N-tert*-Butoxycarbonyl-*N'*-chloro-*N'*-hexyl-*N*-(2,2,2-trifluoro)ethylsulfamide (5b)**

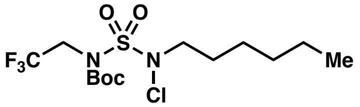

Prepared from sulfamide **S7b** (181 mg, 0.5 mmol) and TCICA (139 mg, 0.6 mmol) in CH_2Cl_2 (5 mL) following general procedure G. The product was obtained as a colorless oil (191 mg, 96% yield) after silica gel column chromatography (Gradient: 100% hexanes → 20:1 hexanes/EtOAc).

^{1}H NMR (400 MHz, $CDCl_3$) δ 4.42 (q, *J* = 8.1 Hz, 2H), 3.67 (dd, *J* = 7.0 Hz, 2H), 1.75–1.68 (m, 2H), 1.54 (s, 9H), 1.38–1.30 (m, 6H), 0.90 (t, *J* = 6.7 Hz, 3H).
^{13}C NMR (126 MHz, $CDCl_3$) δ 149.5, 123.2 (q, *J* = 280 Hz), 86.2, 58.7, 49.3 (q, *J* = 36.3 Hz), 31.2, 27.7, 27.5, 25.6, 22.4, 13.9.
^{19}F NMR (376 MHz, $CDCl_3$) δ –70.54 (t, *J* = 8.2 Hz).
IR (neat) ν 2935 (w), 1746 (m), 1459 (w), 1389 (m), 1371 (m), 1338 (m), 1296 (w), 1250 (m), 1139 (s), 1046 (m), 1027 (m), 909 (w), 847 (w), 831 (w), 797 (w), 772 (w), 728 (w), 711 (m), 680 (w), 646 (w), 611 (m), 580 (m), 567 (m), 529 (w), 463 (w) cm^{-1}.
TLC R_f = 0.50 in 9:1 hexanes:EtOAc.
HRMS (ESI) m/z: $[M + NH_4]^+$ Calcd for $C_{13}H_{24}ClF_3N_2O_4S$•NH_4 414.1436; Found 414.1444.

***N-tert*-Butoxycarbonyl-*N'*-*tert*-butyl-*N'*-chloro-*N*-neopentylsulfamide (1a)**

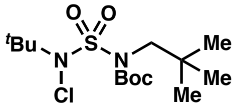

Prepared from sulfamide **S8a** (322 mg, 1.0 mmol) and TCICA (279 mg, 1.2 mmol) in CH_2Cl_2 (5 mL) following general procedure G. The product was obtained as a white solid (158 mg, 44% yield) after silica gel column chromatography (Gradient: 100% hexanes → 50:1 hexanes/EtOAc).

^{1}H NMR (400 MHz, $CDCl_3$) δ 3.71 (s, 2H), 1.54 (s, 9H), 1.52 (s, 9H), 0.93 (s, 9H).
^{13}C NMR (101 MHz, $CDCl_3$) δ 151.0, 84.1, 68.3, 59.4, 33.3, 28.9, 27.8, 27.6.
IR (neat) ν 2968 (w), 1737 (m), 1478 (w), 1380 (m), 1365 (s), 1334 (w), 1278 (m), 1252 (m), 1186 (m), 1146 (s), 1073 (m), 1039 (w), 1004 (m), 871 (m), 849 (m), 819 (m), 800 (m), 771 (m), 731 (w), 656 (m), 587 (w) cm^{-1}.
TLC R_f = 0.56 in 9:1 hexanes:EtOAc.
HRMS (ESI) m/z: $[M + Na]^+$ Calcd for $C_{14}H_{29}ClN_2O_4S$•Na 379.1429; Found 379.1429.

***N-tert*-Butoxycarbonyl-*N*-butyl-*N'*-*tert*-butyl-*N'*-chlorosulfamide (1b)**

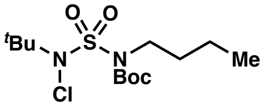

Prepared from sulfamide **S8b** (308 mg, 1.0 mmol) and TCICA (279 mg, 1.2 mmol) in CH_2Cl_2 (5 mL) following general procedure G. The product was obtained as a colorless oil (258 mg, 75% yield) after silica gel column chromatography (Gradient: 100% hexanes → 50:1 hexanes/EtOAc).

^{1}H NMR (400 MHz, $CDCl_3$) δ 3.75 (dd, *J* = 7.6, 7.6 Hz, 2H), 1.70–1.62 (m, 2H), 1.53 (s, 9H), 1.53 (s, 9H), 1.38–1.29 (m, 2H), 0.92 (t, *J* = 7.3 Hz, 3H).
^{13}C NMR (126 MHz, $CDCl_3$) δ 150.3, 84.1, 68.3, 50.1, 31.8, 29.0, 28.0, 19.8, 13.7.
IR (neat) ν 2979 (w), 2938 (w), 2877 (w), 1715 (s), 1466 (w), 1440 (w), 1382 (m), 1367 (s), 1336 (m), 1294 (s), 1257 (m), 1184 (m), 1148 (s), 1046 (m), 1021 (m), 1009 (m), 925 (m), 875 (m), 848 (s), 830 (m), 770 (m), 763 (m), 731 (m), 648 (s), 581 (s), 548 (m) cm^{-1}.
TLC R_f = 0.47 in 9:1 hexanes:EtOAc.
HRMS (ESI) m/z: $[M + Na]^+$ Calcd for $C_{13}H_{27}ClN_2O_4S{\bullet}Na$ 365.1272; Found 365.1281.

N-tert-Butoxycarbonyl-_N'-tert_-butyl-_N'_-chloro-_N_-isopentylsulfamide (1c)

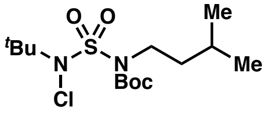

Prepared from sulfamide **S8c** (208 mg, 0.65 mmol) and TCICA (180 mg, 0.77 mmol) in CH_2Cl_2 (3.2 mL) following general procedure G. The product was isolated as a white solid (161 mg, 70% yield) after silica gel column chromatography (95:5 hexanes/EtOAc).

^{1}H NMR (500 MHz, C_6D_6) δ 3.90–3.86 (m, 2H), 1.68–1.62 (m, 2H), 1.47–1.42 (m, 1H), 1.35 (s, 9H) 1.33 (s, 9H), 0.76 (d, *J* = 6.6 Hz, 6H).
^{13}C NMR (126 MHz, $CDCl_3$) δ 150.3, 84.1, 68.4, 49.0, 38.5, 29.0, 28.0, 26.0, 22.4.
IR (neat) ν 2957 (w), 2872 (w), 1728 (m), 1380 (m), 1366 (s), 1287 (m), 1257 (m), 1187 (m), 1146 (s), 1046 (m), 1022 (m), 873 (m), 771 (m), 745 (m), 730 (m) cm^{-1}.
TLC R_f = 0.53 in 8:2 hexanes/EtOAc.
HRMS (ESI) m/z: $[M + Na]^+$ Calcd for $C_{14}H_{29}ClN_2O_4S{\bullet}Na$ 379.1434; Found 379.1428.

N-tert-Butoxycarbonyl-_N'-tert_-butyl-_N'_-chloro-_N_-(2-(cyclopentyl)eth-1-yl)sulfamide (1d)

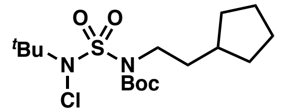

Prepared from sulfamide **S8d** (439 mg, 1.0 mmol) and TCICA (279 mg, 1.2 mmol) in CH_2Cl_2 (5 mL) following general procedure G. The product was obtained as a colorless oil that solidified upon storage at –20 °C (331 mg, 86% yield) after silica gel column chromatography (20:1 hexanes/EtOAc).

^{1}H NMR (400 MHz, $CDCl_3$) δ 3.75 (dd, *J* = 7.7, 7.7 Hz, 2H), 1.80–1.75 (m, 3H), 1.74–1.67 (m, 3H), 1.62–1.57 (m, 2H), 1.53 (s, 9H), 1.53 (s, 9H), 1.11–1.18 (m, 3H).
^{13}C NMR (126 MHz, $CDCl_3$) δ 150.3, 84.1, 68.3, 49.8, 37.4, 36.0, 32.4, 29.0, 28.0, 25.1.
IR (neat) ν 2943 (w), 2867 (w), 1728 (m), 1454 (w), 1380 (m), 1366 (s), 1335 (m), 1286 (m), 1257 (m), 1187 (m), 1146 (s), 1046 (m), 978 (w), 873 (m), 849 (m), 771 (m), 747 (w), 731 (w), 634 (m), 611 (m), 586 (m) cm^{-1}.
TLC R_f = 0.57 in 9:1 hexanes:EtOAc.
HRMS (ESI) m/z: $[M + Na]^+$ Calcd for $C_{16}H_{31}ClN_2O_4S{\bullet}Na$ 405.1585; Found 405.1580.

N-tert-Butoxycarbonyl-_N'-tert_-butyl-_N'_-chloro-_N_-(2-(cyclohexyl)eth-1-yl)sulfamide (1e)

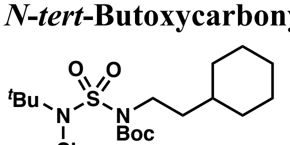

Prepared from sulfamide **S8e** (363 mg, 1.0 mmol) and TCICA (279 mg, 1.2 mmol) in CH_2Cl_2 (5 mL) following general procedure G. The product was obtained as a colorless oil (293 mg, 74% yield) after silica gel column chromatography (Gradient: 100% hexanes → 50:1 hexanes/EtOAc).

^{1}H NMR (400 MHz, $CDCl_3$) δ 3.79–3.75 (m, 2H), 1.74–1.65 (m, 7H), 1.53 (s, 9H), 1.52 (s, 9H), 1.23–1.13 (m, 4H), 0.98–0.90 (m, 2H).
^{13}C NMR (126 MHz, $CDCl_3$) δ 150.2, 84.0, 68.2, 48.5, 37.0, 35.4, 33.0, 28.9, 27.9, 26.4, 26.1.
IR (neat) ν 2979 (w), 2923 (m), 2851(w), 1730 (m), 1449 (w), 1380 (m), 1367 (s), 1334 (w), 1310 (w), 1287 (m), 1257 (m), 1185 (m), 1146 (s), 1043 (w), 1025 (w), 966 (m), 870 (m), 850 (m), 829 (m), 771 (m), 730 (w), 648 (m), 601 (m), 586 (m), 554 (w) cm^{-1}.
TLC R_f = 0.63 in 4:1 hexanes:EtOAc
HRMS (ESI) m/z: $[M + Na]^+$ Calcd for $C_{17}H_{33}ClN_2O_4S$•Na 419.1742; Found 419.1738.

***N*-*tert*-Butoxycarbonyl-*N'*-*tert*-butyl-*N'*-chloro-*N*-((*2R*)-methyl 4-methylpentano-2-yl)sulfamide (1f)**

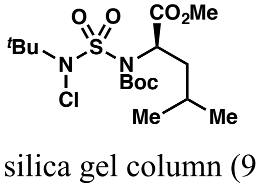

Prepared from sulfamide **S8f** (280 mg, 0.74 mmol) and TCICA (205 mg, 0.88 mmol) in CH_2Cl_2 (3.7 mL) following general procedure G. The product was obtained as a colorless oil (228 mg, 75% yield) after purification on a Teledyne Isco CombiFlashR$_f$ system using a Redi Sep R$_f$ normal phase silica gel column (9:1 hexanes/EtOAc).

^{1}H NMR (400 MHz, $CDCl_3$) δ 4.77 (dd, *J* = 7.1, 5.5 Hz, 1H), 3.74 (s, 3H), 2.22 (ddd, *J* = 13.9, 6.8, 6.8 Hz, 1H), 1.92–1.82 (m, 1H), 1.70 (ddd, *J* = 14.0, 7.2, 5.4 Hz, 1H), 1.56 (s, 9H), 1.50 (s, 9H), 0.96 (t, *J* = 7.0 Hz, 6H).
^{13}C NMR (101 MHz, $CDCl_3$) δ 170.5, 149.4, 84.9, 69.2, 59.8, 52.3, 40.4, 29.0, 27.8, 25.7, 22.6, 22.3.
IR (neat) ν 2956 (w), 2871 (w), 1746 (m), 1459 (w), 1382 (m), 1366 (s), 1295 (m), 1236 (m), 1209 (m), 1186 (m), 1143 (s), 1044 (m), 1029 (m), 986 (w), 948 (w), 919 (w), 868 (m), 835 (m), 770 (m), 723 (m), 664 (w), 613 (s), 589 (m) cm^{-1}.
TLC R_f = 0.49 in 8:2 hexanes:EtOAc.
HRMS (ESI) m/z: $[M + Na]^+$ Calcd for $C_{16}H_{31}ClN_2O_6S$•Na 437.1489; Found 437.1485.

***N*-*tert*-Butoxycarbonyl-*N'*-*tert*-butyl-*N'*-chloro-*N*-((*S*)-2-(1,3-dioxoisoindolin-2-yl)-3-methylbutyl)sulfamide (1g)**

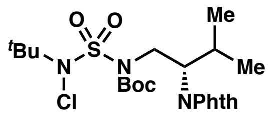

Prepared from sulfamide **S8g** (468 mg, 1.0 mmol) and TCICA (279 mg, 1.2 mmol) in CH_2Cl_2 (5 mL) following general procedure G. The product was obtained as a white foam (403 mg, 80% yield) after silica gel column chromatography (Gradient: 20:1 hexanes:EtOAc → 9:1 hexanes/EtOAc).

^{1}H NMR (400 MHz, $CDCl_3$) δ 7.84 (dd, *J* = 3.1, 5.3 Hz, 2H), 7.68 (dd, *J* = 3.1, 5.3 Hz, 2H), 4.44 (dd, *J* = 10.8, 14.5, 1H), 4.17 (ddd, *J* = 2.5, 2.5, 10.8, 1H), 4.09 (dd, *J* = 2.5, 14.5 Hz, 1H), 2.64–2.55 (m, 1H), 1.45 (s, 9H), 1.43 (s, 9H), 1.15 (d, *J* = 6.6 Hz, 3H), 0.84 (d, *J* = 6.6 Hz, 3H).
^{13}C NMR (126 MHz, $CDCl_3$) δ 168.4, 149.8, 133.6, 131.9, 131.4, 123.1, 122.8, 84.5, 68.2, 57.3, 49.3, 28.7, 27.7, 27.2, 20.3, 20.0.
IR (neat) ν 2976 (w), 1776 (w), 1713 (s), 1468 (w), 1384 (m), 1368 (m), 1318 (w), 1288 (w), 1258 (w), 1189 (w), 1150 (m), 1064 (w), 1009 (w), 991 (w), 875 (w), 845 (w), 771 (w), 722 (m), 644 (w), 588 (w), 530 (w) cm^{-1}.
TLC R_f = 0.33 in 4:1 hexanes:EtOAc
HRMS (ESI) m/z: $[M + NH_4]^+$ Calcd for $C_{22}H_{32}ClN_3O_6S$•NH_4 519.2039; Found 519.2045.

***N-tert*-Butoxycarbonyl-*N'*-*tert*-butyl-*N'*-chloro-*N*-(3,7-dimethyloct-1-yl)sulfamide (1h)**

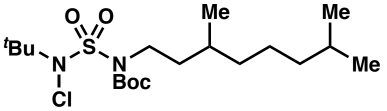

Prepared from sulfamide **S8h** (709 mg, 1.8 mmol) and TCICA (504 mg, 2.2 mmol) in CH_2Cl_2 (9 mL) following general procedure G. The product was isolated as a colorless oil (662 mg, 86% yield) after purification on a Teledyne Isco CombiFlashR$_f$ system using a Redi Sep R$_f$ normal phase silica gel column (95:5 hexanes/EtOAc).

^{1}H NMR (400 MHz, $CDCl_3$) δ 3.79–3.70 (m, 2H), 1.75–1.67 (m, 2H), 1.57–1.48 (m, 19H), 1.33–1.22 (m, 4H), 1.16–1.11 (m, 3H), 0.91 (d, J = 6.4 Hz, 3H), 0.86 (d, J = 6.7 Hz, 6H).
^{13}C NMR (126 MHz, $CDCl_3$) δ 150.2, 84.0, 68.3, 48.8, 39.1, 37.0, 36.7, 30.8, 29.0, 28.0, 27.9, 24.6, 22.7, 22.6, 19.4.
IR (neat) ν 2954 (w), 2928 (w), 2869 (w), 1730 (m), 1458 (w), 1380 (m), 1366 (s), 1284 (m), 1257 (m), 1147 (s), 1042 (w), 1018 (w), 957 (w), 873 (m), 850 (m), 771 (w), 731 (m), 645 (m), 586 (m) cm^{-1}.
TLC R$_f$ = 0.66 in 8:2 hexanes/EtOAc.
HRMS (ESI) m/z: [M + Na]$^+$ Calcd for $C_{19}H_{39}ClN_2O_4S$•Na 449.2216; Found 449.2213.

***N-tert*-Butoxycarbonyl-*N'*-*tert*-butyl-*N'*-chloro-*N*-pentylsulfamide (1i)**

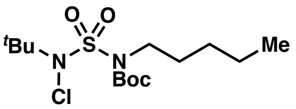

Prepared from sulfamide **S8i** (500 mg, 1.55 mmol) and TCICA (432 mg, 1.86 mmol, 1.20 equiv) in CH_2Cl_2 (8.3 mL) following general procedure G. The product was isolated as a colorless oil (503 mg, 91% yield) after purification on Florisil by solid deposition (95:5 hexanes/EtOAc).

^{1}H NMR (400 MHz, $CDCl_3$) δ 3.76–3.72 (m, 2H), 1.72–1.64 (m, 2H), 1.53 (s, 9H), 1.53 (s, 9H), 1.38–1.26 (m, 4H), 0.89 (t, J = 7.0 Hz, 3H).
^{13}C NMR (126 MHz, $CDCl_3$) δ 150.3, 84.1, 68.3, 50.2, 29.3, 28.9, 28.7, 28.0, 22.2, 13.9.
IR (neat) ν 2958 (w), 2934 (w), 2872 (w), 1729 (m), 1458 (w), 1380 (m), 1366 (s), 1333 (w), 1282 (m), 1256 (m), 1186 (m), 1147 (m), 1043 (m), 1017 (m), 962 (w), 874 (m), 848 (m), 823 (m), 770 (m), 727 (m), 644 (m), 633 (m), 585 (s) cm^{-1}.
TLC R$_f$ = 0.49 in 9:1 hexanes/EtOAc.
HRMS (ESI) m/z: [M + Na]$^+$ Calcd for $C_{14}H_{29}ClN_2O_4S$•Na 379.1434; Found 379.1427.

***N-tert*-Butoxycarbonyl-*N'*-*tert*-butyl-*N'*-chloro-*N*-hexylsulfamide (1j)**

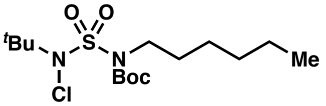

Prepared from sulfamide **S8j** (336 mg, 1.0 mmol) and TCICA (279 mg, 1.2 mmol) in CH_2Cl_2 (5 mL) following general procedure G. The product was obtained as a colorless oil (291 mg, 79% yield) after silica gel column chromatography (Gradient: 100% hexanes → 50:1 hexanes/EtOAc).

^{1}H NMR (400 MHz, $CDCl_3$) δ 3.74 (dd, J = 7.6, 7.6 Hz, 2H), 1.69–1.65 (m, 2H), 1.53 (s, 9H), 1.53 (s, 9H), 1.32–1.27 (m, 6H), 0.87 (t, J = 6.7 Hz, 3H).
^{13}C NMR (101 MHz, $CDCl_3$) δ 150.3, 84.1, 68.3, 50.3, 31.3, 29.6, 28.9, 28.0, 26.2, 22.5, 14.0.
IR (neat) ν 2932 (w), 2859 (w), 1729 (m), 1458 (w), 1381 (m), 1367 (s), 1333 (w), 1285 (m), 1257 (m), 1185 (m), 1148 (s), 1046 (m), 984 (w), 912 (w), 873 (m), 849 (m), 770 (m), 731 (s), 646 (m), 634 (m), 587 (s) cm^{-1}.

TLC R_f = 0.57 in 9:1 hexanes:EtOAc.
HRMS (ESI) m/z: $[M + Na]^+$ Calcd for $C_{15}H_{31}ClN_2O_4S$•Na 393.1585; Found 393.1585.

***N*-*tert*-Butyoxycarbonyl-*N'*-*tert*-butyl-*N'*-chloro-*N*-(methyl hexano-6-yl)sulfamide (1k)**

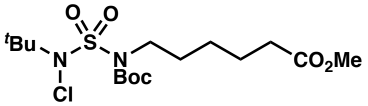

Prepared from sulfamide **S8k** (266 mg, 0.7 mmol) and TCICA (195 mg, 0.84 mmol) in CH_2Cl_2 (3.5 mL) following general procedure G. The product was obtained as a colorless oil (217 mg, 75% yield) after silica gel column chromatography (Gradient: 100% hexanes → 9:1 hexanes/EtOAc).

^{1}H NMR (400 MHz, $CDCl_3$) δ 3.74 (d, *J* = 7.4, 7.4 Hz, 2H), 3.65 (s, 3H), 2.31 (t, *J* = 7.5 Hz, 2H), 1.72–1.61 (m, 4H), 1.53 (s, 9H), 1.52 (s, 9H), 1.38–1.30 (m, 2H).
^{13}C NMR (101 MHz, $CDCl_3$) δ 173.9, 150.3, 84.2, 68.4, 51.4, 49.9, 33.9, 29.3, 28.9, 28.0, 26.0, 24.4.
IR (neat) ν 2981 (w), 2940 (w), 1279 (s), 1437 (w), 1380 (m), 1366 (s), 1335 (w), 1285 (m), 1257 (m), 1146 (s), 1042 (w), 1009 (w), 874 (m), 848 (m), 770 (w), 730 (m), 634 (m), 586 (s) cm^{-1}.
TLC R_f = 0.44 in 4:1 hexanes:EtOAc.
HRMS (ESI) m/z: $[M + NH_4]^+$ Calcd for $C_{16}H_{31}ClN_2O_6S$•NH_4 432.1930; Found 432.1937.

***N*-Acetyl-*N'*-*tert*-butyl-*N'*-chloro-*N*-hexylsulfamide (1l)**

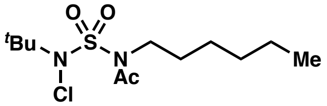

Prepared from sulfamide **S8l** (278 mg, 1.0 mmol) and ^{t}BuOCl (217 mg, 2.0 mmol) in CH_2Cl_2 (5 mL) following general procedure H. The product was obtained as a colorless oil (235 mg, 75% yield) after silica gel column chromatography (Gradient: 100% hexanes → 50:1 hexanes/EtOAc).

^{1}H NMR (400 MHz, $CDCl_3$) δ 3.78 (dd, *J* = 7.8, 7.8 Hz, 2H), 2.54 (s, 3H), 1.66–1.59 (m, 2H), 1.55 (s, 9H), 1.26–1.31 (m, 6H), 0.88 (t, *J* = 6.8 Hz, 3H).
^{13}C NMR (126 MHz, C_6D_6) δ 169.5, 68.5, 49.1, 31.6, 29.4, 28.7, 26.7, 24.6, 22.8, 14.1.
^{13}C NMR (101 MHz, $CDCl_3$) δ 170.2, 68.9, 49.0, 31.3, 28.9, 26.3, 24.7, 22.5, 14.0.
IR (neat) ν 2931 (w), 2858 (w), 1709 (m), 1462 (w), 1400 (w), 1365 (s), 1329 (w), 1232 (m), 1177 (s), 1162 (s), 1092 (m), 1040 (w), 948 (m), 912 (w), 864 (m), 823 (m), 781 (m), 732 (m), 709 (w), 678 (w), 649 (w), 602 (s), 583 (s), 554 (m) cm^{-1}.
TLC R_f = 0.43 in 9:1 hexanes:EtOAc.
HRMS (ESI) m/z: $[M + Na]^+$ Calcd for $C_{12}H_{25}ClN_2O_3S$•Na 335.1167; Found 335.1175.

***N*-*tert*-Butyl-*N*-chloro-*N'*-hexyl-*N'*-(2,2,2-trifluoroethyl)sulfamide (1m)**

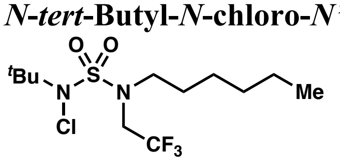

Prepared from sulfamide **S8m** (318 mg, 1.0 mmol) and TCICA (279 mg, 1.2 mmol) in CH_2Cl_2 (5 mL) following general procedure G. The product was obtained as a colorless oil (318 mg, 90% yield) after silica gel column chromatography (Gradient: 100% hexanes → 50:1 hexanes/EtOAc).

^{1}H NMR (400 MHz, $CDCl_3$) δ 3.93 (q, *J* = 8.7 Hz, 2H), 3.38 (dd, *J* = 8.1, 8.1 Hz, 2H), 1.73–1.65 (m, 2H), 1.50 (s, 9H), 1.34–1.28 (m, 6H), 0.89 (t, *J* = 6.8 Hz, 3H).

^{13}C NMR (126 MHz, $CDCl_3$) δ 123.8 (q, *J* = 280.1 Hz), 67.7, 50.8, 50.5 (q, *J* = 35.2 Hz), 31.2, 28.9, 26.8, 26.2, 22.5, 13.9.
^{19}F NMR (376 MHz, $CDCl_3$) δ –70.11 (t, *J* = 8.7 Hz).
IR (neat) ν 2933 (w), 2860 (w), 1466 (w), 1399 (w), 1359 (m), 1314 (w), 1274 (m), 1238 (w), 1151 (s), 1119 (m), 1043 (m), 994 (m), 875 (m), 820 (m), 785 (m), 733 (m), 663 (m), 603 (m), 564 (m) cm^{-1}.
TLC R_f = 0.57 in 9:1 hexanes:EtOAc.
HRMS (ESI) m/z: $[M + NH_4]^+$ Calcd for $C_{12}H_{24}ClF_3N_2O_2S{\bullet}NH_4$ 370.1537; Found 370.1539.

***N'-tert*-Butyl-*N'*-chloro-*N*-trifluoroacetyl-*N*-pentylsulfamide (1n)**

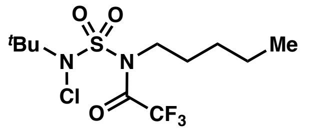

Prepared from sulfamide **S8n** (284 mg, 0.89 mmol) and ^{t}BuOCl (4.2 mL, 37.2 mmol, 2 x 21 equiv portions, 2nd portion added after 24 h) following general procedure H. The product was isolated as a colorless oil (273 mg, 87% yield) without purification.

^{1}H NMR (400 MHz, $CDCl_3$) δ 3.87–3.82 (m, 2H), 1.78–1.72 (m, 2H), 1.57 (s, 9H), 1.38–1.28 (m, 4H), 0.91 (t, *J* = 7.0 Hz, 3H).
^{13}C NMR (126 MHz, $CDCl_3$) δ 155.8 (q, *J* = 40.4 Hz), 115.3 (q, *J* = 291.0 Hz), 70.3, 50.1, 29.5, 29.0, 28.5, 22.0, 13.8.
^{19}F NMR (376 MHz, $CDCl_3$) δ –69.75.
IR (neat) ν 2960 (w), 2874 (w), 1741 (m), 1464 (w), 1390 (m), 1371 (m), 1231 (m), 1209 (m), 1155 (s), 1128 (s), 1060 (m), 969 (w), 874 (m) 784 (m), 754 (m), 715 (m), 688 (m), 615 (s), 581 (m), 537 (m) cm^{-1}.
TLC R_f = 0.29 in 98:2 hexanes/EtOAc.
HRMS (ESI) m/z: $[M – H]^–$ Calcd for $C_{11}H_{19}ClF_3N_2O_3S$ 351.0757; Found 351.0764.

***N*-*tert*-Butoxycarbonyl-*N'*-chloro-*N'*-(2,2,2-trifluoroethyl)-*N*-hexylsulfamide (1o)**

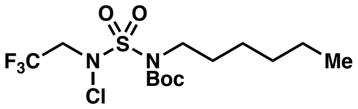

Prepared from sulfamide **S8o** (382 mg, 1.0 mmol) and TCICA (279 mg, 1.2 mmol) in CH_2Cl_2 (5 mL) following general procedure G. The product was obtained as a colorless oil (378 mg, 95% yield) after silica gel column chromatography (Gradient: 100% hexanes → 50:1 hexanes/EtOAc).

^{1}H NMR (400 MHz, $CDCl_3$) δ 4.28 (q, *J* = 8.2 Hz, 2H), 3.74 (dd, *J* = 7.7, 7.7 Hz, 2H), 1.75–1.68 (m, 2H), 1.54 (s, 9H), 1.36–1.27 (m, 6H), 0.90 (t, *J* = 6.7 Hz, 3H).
^{13}C NMR (126 MHz, $CDCl_3$) δ 150.8, 123.2 (q, *J* = 279.3 Hz), 85.2, 59.5 (q, *J* = 36.1 Hz), 50.8, 31.3, 29.5, 27.9, 26.1, 22.5, 13.9.
^{19}F NMR (376 MHz, $CDCl_3$) δ –71.64 (t, *J* = 8.3 Hz).
IR (neat) ν 2934 (w), 2860 (w), 1730 (m), 1458 (w), 1382 (m), 1372 (m), 1338 (w), 1305 (m), 1274 (m), 1255 (m), 1137 (s), 1045 (w), 967 (m), 841 (m), 820 (m), 772 (w), 721 (s), 697 (m), 641 (w), 617 (w), 563 (s) cm^{-1}.
TLC R_f = 0.50 in 9:1 hexanes:EtOAc.
HRMS (ESI) m/z: $[M – H]^–$ Calcd for $C_{13}H_{24}ClF_3N_2O_4S$ 395.1025; Found 395.1023.

***N-tert*-Butyl-*N*-chloro-*N*'-4-methylpentyl-*N*'-(2,2,2-trifluoroethyl)sulfamide (1p)**

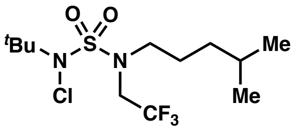

Prepared from sulfamide **S8p** (318 mg, 1.0 mmol) and TCICA (279 mg, 1.2 mmol) in CH_2Cl_2 (5 mL) following general procedure G. The product was obtained as a colorless oil (306 mg, 87% yield) after silica gel column chromatography (Gradient: 100% hexanes → 50:1 hexanes/EtOAc).

^{1}H NMR (400 MHz, $CDCl_3$) δ 3.93 (q, *J* = 8.5 Hz, 2H), 3.37 (dd, *J* = 7.7, 7.7 Hz, 2H), 1.74–1.66 (m, 2H), 1.61–1.54 (m, 1H), 1.49 (s, 9H), 1.20–1.15 (m, 2H), 0.90 (d, *J* = 6.6 Hz, 6H).
^{13}C NMR (126 MHz, $CDCl_3$) δ 123.8 (q, *J* = 280.1 Hz), 67.7, 51.0, 50.4 (q, *J* = 35.5 Hz), 35.6, 28.9, 27.6, 24.7, 22.4.
^{19}F NMR (376 MHz, $CDCl_3$) δ –70.13 (t, *J* = 8.7 Hz).
IR (neat) ν 2957 (w), 2873 (w), 1466 (w), 1360 (m), 1313 (m), 1275 (m), 1238 (w), 1180 (m), 1152 (s), 1120 (m), 1042 (m), 991 (m), 875 (m), 822 (m), 786 (m), 765 (w), 735 (w), 663 (m), 604 (m), 564 (m) cm^{-1}.
TLC R_f = 0.57 in 9:1 hexanes:EtOAc.
HRMS (ESI) m/z: $[M + H]^+$ Calcd for $C_{12}H_{24}ClF_3N_2O_2S$ 353.1272; Found 353.1279.

***N-tert*-Butoxycarbonyl-*N'-tert*-butyl-*N*'-chloro-*N*-4-methylpentylsulfamide (1q)**

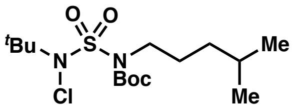

Prepared from sulfamide **S8q** (336 mg, 1.0 mmol) and TCICA (279 mg, 1.2 mmol) in CH_2Cl_2 (5 mL) following general procedure G. The product was obtained as a white solid (284 mg, 77% yield) after silica gel column chromatography (Gradient: 100% hexanes → 50:1 hexanes/EtOAc).

^{1}H NMR (400 MHz, $CDCl_3$) δ 3.72 (dd, *J* = 7.4 Hz, 2H), 1.71–1.64 (m, 3H), 1.53 (s, 9H), 1.52 (s, 9H), 1.20–1.15 (m, 2H), 0.87 (d, *J* = 6.6 Hz, 6H).
^{13}C NMR (126 MHz, $CDCl_3$) δ 150.3, 84.1, 68.3, 50.5, 35.6, 29.0, 28.0, 27.6, 27.5, 22.5.
IR (neat) ν 2956 (w), 2871 (w), 1729 (m), 1458 (w), 1380 (m), 1366 (s), 1331 (w), 1284 (m), 1257 (m), 1186 (m), 1147 (s), 1048 (m), 981 (m), 874 (m), 793 (m), 771 (m), 730 (m), 645 (m), 634 (m), 586 (s) cm^{-1}.
TLC R_f = 0.55 in 9:1 hexanes:EtOAc.
HRMS (ESI) m/z: $[M + Na]^+$ Calcd for $C_{15}H_{31}ClN_2O_4S$•Na 393.1585; Found 393.1587.

***N-tert*-Butoxycarbonyl-*N*'-chloro-*N*-4-methylpentyl-*N'*-(2,2,2-trifluoroethyl)sulfamide (1r)**

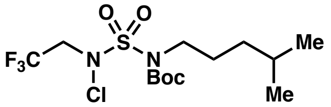

Prepared from sulfamide **S8r** (362 mg, 1.0 mmol) and TCICA (279 mg, 1.2 mmol) in CH_2Cl_2 (5 mL) following general procedure G. The product was obtained as a colorless oil (357 mg, 90% yield) after silica gel column chromatography (Gradient: 100% hexanes → 50:1 hexanes/EtOAc).

^{1}H NMR (400 MHz, $CDCl_3$) δ 4.28 (q, *J* = 8.2 Hz, 2H), 3.72 (dd, *J* = 7.7, 7.7 Hz, 2H), 1.75–1.68 (m, 2H), 1.59–1.55 (m, 1H), 1.54 (s, 9H), 1.22–1.16 (m, 2H), 0.89 (d, *J* = 6.5 Hz, 6H).
^{13}C NMR (101 MHz, $CDCl_3$) δ 150.8, 123.2 (q, *J* = 279.4 Hz), 85.2, 59.5 (q, *J* = 35.4 Hz), 50.9, 35.5, 27.8, 27.5, 27.4, 22.4.
^{19}F NMR (376 MHz, $CDCl_3$) δ –71.56 (t, *J* = 8.3 Hz).

IR (neat) ν 2958 (w), 2872 (w), 1730 (m), 1459 (w), 1382 (m), 1371 (m), 1335 (w), 1305 (m), 1274 (m), 1138 (s), 1047 (w), 967 (m), 841 (m), 822 (m), 794 (m), 772 (w), 721 (s), 697 (m), 641 (w), 617 (w), 563 (s) cm^{-1}.
TLC R_f = 0.49 in 9:1 hexanes:EtOAc.
HRMS (ESI) m/z: $[M + H]^+$ Calcd for $C_{13}H_{24}ClF_3N_2O_4S$ 397.1170; Found 397.1166.

***N-tert*-Butoxycarbonyl-*N'*-tert-butyl-*N*-(*tert*-butyl butano-4-yl)-*N'*-chlorosulfamide (S9t)**

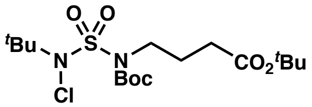

Following a modified literature procedure,[6] a flame-dried round bottom flask equipped with a stir bar and shielded from light with aluminum foil was charged with sulfamide **S8t** (200 mg, 0.51 mmol, 1.0 equiv) by brief removal of the septum. The septum was tightly replaced and the flask was sequentially charged with EtOAc (0.91 mL, 0.56 M), ^{t}BuOH (48 μL, 0.51 mmol, 1.0 equiv), 0.9 M aqueous bleach solution (3.8 mL, 3.39 mmol, 6.7 equiv), and AcOH (0.29 mL, 5.07 mmol, 10 equiv) via syringe. The biphasic mixture was stirred until complete consumption of the starting material as was evident by TLC.

Upon complete consumption of the starting material as indicated by TLC, the reaction was diluted with EtOAc (19.7 mL/mmol of sulfamide) and the biphasic mixture was partitioned in a separatory funnel. The aqueous phase was separated and the organic phase was rinsed with saturated aqueous $NaHCO_3$ solution (19.7 mL/mmol sulfamide), saturated aqueous NaCl solution (19.7 mL/mmol sulfamide), and H_2O (19.7 mL/mmol sulfamide). The organic phase was collected, dried over Na_2SO_4, filtered, & concentrated to afford the crude product. The product was isolated as a colorless oil (168 mg, 77% yield) after purification on a Teledyne Isco CombiFlashR_f system using a Redi Sep R_f normal phase silica gel column (9:1 hexanes/EtOAc).

^{1}H NMR (400 MHz, $CDCl_3$) δ 3.81 (t, J = 7.1 Hz, 2H), 2.26 (t, J = 7.5 Hz, 2H), 1.99–1.93 (m, 2H), 1.54 (s, 9H), 1.53 (s, 9H), 1.44 (s, 9H).
^{13}C NMR (126 MHz, $CDCl_3$) δ 172.0, 150.2, 84.4, 80.3, 49.3, 32.5, 28.9, 28.0, 28.0, 25.0.
IR (neat) ν 2979 (w), 2935 (w), 1726 (s), 1457 (w), 1380 (m), 1365 (s), 1334 (w), 1285 (m), 1256 (m), 1143 (s), 1099 (m), 1040 (w), 997 (w), 874 (m), 847 (m), 769 (m), 730 (w), 632 (m), 586 (m) cm^{-1}.
TLC R_f = 0.61 in 8:2 hexanes/EtOAc.
HRMS (ESI) m/z: $[M + Na]^+$ Calcd for $C_{17}H_{33}ClN_2O_6S$•Na 451.1645; Found 451.1644.

***N-tert*-Butoxycarbonyl-*N'*-*tert*-butyl-*N'*-chloro-*N*-(3-((4-trifluoromethyl)phenyl)prop-1-yl) sulfamide (S9u)**

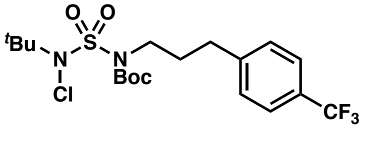

Prepared from sulfamide **S8u** (439 mg, 1.0 mmol) and TCICA (279 mg, 1.2 mmol) in CH_2Cl_2 (5 mL, 0.2 M) following general procedure G. The product was obtained as colorless oil that solidified upon storage at –20 °C (449 mg, 95% yield) after silica gel column chromatography (20:1 hexanes/EtOAc).

^{1}H NMR (400 MHz, $CDCl_3$) δ 7.53 (d, J = 8.1 Hz, 2H), 7.31 (d, J = 8.1 Hz, 2H), 3.81 (t, J = 7.5 Hz, 2H), 2.71 (t, J = 7.9 Hz, 2H), 2.08–2.00 (m, 2H), 1.53 (s, 9H), 1.52 (s, 9H).
^{13}C NMR (126 MHz, $CDCl_3$) δ 150.2, 145.3, 128.6, 128.3 (q, J = 32.4 Hz), 125.3 (q, J = 3.7 Hz), 124.3 (q, J = 271.6 Hz), 84.4, 68.5, 49.6, 32.7, 30.8, 28.9, 27.9.

^{19}F NMR (376 MHz, $CDCl_3$) δ –62.37.
IR (neat) ν 2985 (w), 1731 (w), 1618 (w), 1458 (w), 1380 (m), 1368 (m), 1324 (m), 1285 (w), 1265 (m), 1148 (m), 1121 (m), 1067 (m), 1018 (w), 996 (w), 951 (w), 875 (w), 843 (w), 798 (w), 782 (w), 734 (s), 704 (m), 627 (m), 587 (m) cm^{-1}.
TLC R_f = 0.5 in 9:1 hexanes:EtOAc.
HRMS (ESI) m/z: $[M + NH_4]^+$ Calcd for $C_{19}H_{28}ClF_3N_2O_4S{\bullet}NH_4$ 490.1749; Found 490.1740.

***N-tert*-Butoxycarbonyl-*N'*-chloro-*N'*-(2,2,2-trifluoroethyl)-*N*-pentylsulfamide (S9v)**

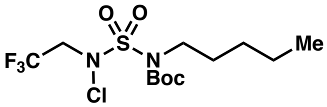

Prepared from sulfamide **S8v** (228 mg, 0.65 mmol) and TCICA (182 mg, 0.78 mmol) following general procedure G. The product was isolated as a colorless oil (229 mg, 92% yield) after purification on a Teledyne Isco CombiFlashR$_f$ system using a Redi Sep R$_f$ normal phase silica gel column (9:1 hexanes/EtOAc).

^{1}H NMR (400 MHz, $CDCl_3$) δ 4.28 (q, J = 8.2 Hz, 2H), 3.76–3.72 (m, 2H), 1.75–1.68 (m, 2H), 1.53 (s, 9H), 1.36–1.29 (m, 4H), 0.91 (t, J = 7.0 Hz, 3H).
^{13}C NMR (126 MHz, $CDCl_3$) δ 150.8, 123.2 (q, J = 279.3 Hz), 85.2, 59.5 (q, J = 35.3 Hz), 50.7, 29.2, 28.6, 27.9, 22.2, 13.9.
^{19}F NMR (376 MHz, $CDCl_3$) δ –71.55 (t, J = 8.2 Hz).
IR (neat) ν 2961 (w), 2873 (w), 1729 (m), 1458 (w), 1381 (m), 1371 (m), 1338 (w), 1305 (m), 1272 (m), 1137 (s), 1043 (w), 1017 (w), 966 (m), 840 (m), 817 (m), 771 (w), 720 (s), 697 (m), 640 (w), 616 (w), 563 (s) cm^{-1}.
TLC R_f = 0.61 in 8:2 hexanes/EtOAc.
HRMS (ESI) m/z: $[M + NH_4]^+$ Calcd for $C_{12}H_{22}ClF_3N_2O_4S{\bullet}NH_4$ 400.1284; Found 400.1269.

***N-tert*-Butoxycarbonyl-*N'*-chloro-*N'*-ethyl-*N*-pentylsulfamide (S9w)**

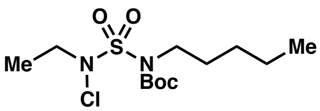

Prepared from sulfamide **S8w** (175 mg, 0.59 mmol) and ^{t}BuOCl (1.4 mL, 12.4 mmol) following general procedure H. The product was isolated as a colorless oil (180 mg, 92% yield) after purification on a Teledyne Isco CombiFlashR$_f$ system using a Redi Sep R$_f$ normal phase silica gel column (9:1 hexanes/EtOAc).

^{1}H NMR (400 MHz, $CDCl_3$) δ 3.77–3.73 (m, 2H), 3.69 (q, J = 7.0 Hz, 2H), 1.75–1.68 (m, 2H), 1.52 (s, 9H), 1.37–1.25 (m, 4H), 1.32 (t, J = 7.0 Hz, 3H), 0.90 (t, J = 7.0 Hz, 3H).
^{13}C NMR (126 MHz, $CDCl_3$) δ 150.5, 84.4, 53.3, 50.4, 29.3, 28.6, 27.9, 22.2, 13.9, 13.1.
IR (neat) ν 2934 (w), 2872 (w), 1730 (m), 1457 (w), 1368 (m), 1335 (w), 1280 (m), 1256 (m), 1179 (m), 1136 (s), 1042 (m), 1015 (m), 961 (w), 846 (m), 802 (w), 770 (m), 720 (m), 642 (m), 606 (m), 584 (m), 570 (m), 534 (m) cm^{-1}.
TLC R_f = 0.61 in 8:2 hexanes/EtOAc.
HRMS (ESI) m/z: $[M + Na]^+$ Calcd for $C_{12}H_{25}ClN_2O_4S{\bullet}Na$ 351.1121; Found 351.1108.

VII. Preparation and characterization of γ-chlorinated sulfamides.

General Procedure I: Chlorine Transfer from *N*-chlorosulfamides.

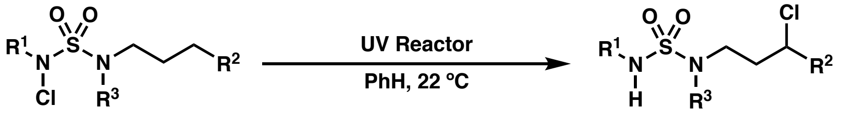

A flame-dried vial equipped with a stir bar was charged with sulfamide (1.0 equiv, 0.2 mmol). The vial was transferred to a nitrogen-filled glovebox where it was charged with anhydrous benzene (PhH, 0.04 M). The vial was sealed with a Teflon lined screw cap and removed from the glovebox. The vial was placed on a stir plate in the center of a Southern New England Ultraviolet Co. RPR-100 Photochemical Reactor barrel and irradiated with 16 ultraviolet lamps until consumption of the starting material as indicated by TLC or ^{1}H NMR analysis of a smaller scale reaction set up simultaneously.

Upon consumption of starting material as evidenced by TLC or ^{1}H NMR, the solution was concentrated under reduced pressure and the crude material was purified as noted below.

***N-tert*-Butoxycarbonyl-*N'*-(4-chloro-hex-1-yl)-*N*-ethylsulfamide (6a)**

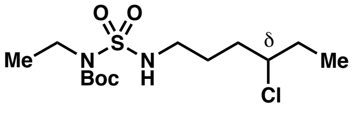

Prepared from chlorosulfamide **5a** (69 mg, 0.2 mmol) according to general procedure I. After irradiating for 15 minutes, the product was isolated as a white solid (54 mg, 78% yield) following purification by silica gel column chromatography (Gradient: 100% hexanes → 20:1 hexanes/EtOAc).

^{1}H NMR (400 MHz, $CDCl_3$) δ 5.27 (t, *J* = 6.4 Hz, 1H), 3.86–3.80 (m, 1H), 3.72 (q, *J* = 7.0 Hz, 2H), 2.98 (dt, *J* = 6.4, 6.4 Hz, 2H), 1.85–1.68 (m, 6H), 1.53 (s, 9H), 1.2 (t, *J* = 7.0 Hz, 3H), 1.03 (t, *J* = 7.3 Hz, 3H).
^{13}C NMR (126 MHz, $CDCl_3$) δ 151.9, 83.8, 64.7, 43.2, 42.8, 34.8, 31.6, 28.0, 26.0, 15.0, 10.9.
IR (neat) ν 3315 (br), 2977 (w), 2937 (w), 1704 (m), 1457 (w), 1425 (w), 1368 (m), 1335 (m), 1280 (m), 1257 (m), 1185 (m), 1138 (s), 1085 (m), 1000 (w), 954 (w), 897 (w), 844 (w), 816 (w), 774 (w), 736 (m), 715 (s), 631 (m), 569 (m), 464 (w) cm^{-1}.
TLC R_f = 0.21 in 9:1 hexanes:EtOAc.
HRMS (ESI) m/z: $[M + Na]^+$ Calcd for $C_{13}H_{27}ClN_2O_4S$•Na 365.1272; Found 365.1271.

***N-tert*-Butoxycarbonyl-*N'*-(4-chloro-hex-1-yl)-*N*-(2,2,2-trifluoro)ethylsulfamide (6b)**

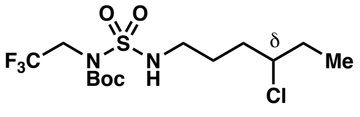

Prepared from chlorosulfamide **5b** (79 mg, 0.2 mmol) according to general procedure I. After irradiating for 15 minutes, the product was isolated as a white solid (69 mg, 87% yield) following purification by silica gel column chromatography (Gradient: 100% hexanes → 20:1 hexanes/EtOAc).

^{1}H NMR (400 MHz, $CDCl_3$) δ 5.33 (t, *J* = 6.2 Hz, 1H), 4.31 (q, *J* = 8.3 Hz, 2H), 3.86–3.80 (m, 1H), 3.07 (dt, *J* = 6.3 Hz, 2H), 1.84–1.68 (m, 6H), 1.53 (s, 9H), 1.03 (t, *J* = 7.3 Hz, 3H).
^{13}C NMR (126 MHz, $CDCl_3$) δ 150.9, 123.4 (q, *J* = 280.5 Hz), 85.6, 64.7, 47.1 (q, *J* = 35.4 Hz), 43.3, 34.7, 31.5, 27.8, 26.0, 10.9.

^{19}F NMR (376 MHz, $CDCl_3$) δ –70.85 (t, *J* = 8.3 Hz).
IR (neat) ν 3323 (br), 2979 (w), 1723 (m), 1423 (w), 1397 (w), 1370 (m), 1344 (m), 1298 (w), 1265 (m), 1252 (m), 1142 (s), 1079 (w), 1046 (w), 1028 (w), 965 (w), 897 (w), 849 (w), 830 (w), 795 (w), 775 (w), 736 (s), 704 (s), 643 (m), 577 (m), 530 (w), 463 (w), 421 (w) cm^{-1}.
TLC R_f = 0.21 in 9:1 hexanes:EtOAc.
HRMS (ESI) m/z: $[M + NH_4]^+$ Calcd for $C_{13}H_{24}ClF_3N_2O_4S•NH_4$ 414.1436; Found 414.1438.

N-tert-Butoxycarbonyl-_N'-tert_-butyl-_N_-(3-chloro-2,2-dimethylprop-1-yl)sulfamide (4a)

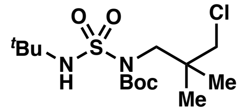

Prepared from chlorosulfamide **1a** (71 mg, 0.2 mmol) according to general procedure I. After irradiating for 15 minutes, the product was isolated as a white solid (67 mg, 94% yield) following purification by silica gel column chromatography (Gradient: 100% hexanes → 9:1 hexanes/EtOAc).

^{1}H NMR (400 MHz, $CDCl_3$) δ 5.43 (br s, 1H), 3.73 (s, 2H), 3.44 (s, 2H), 1.54 (s, 9H), 1.28 (s, 9H), 1.08 (s, 6H).
^{13}C NMR (101 MHz, $CDCl_3$) δ 152.4, 84.0, 54.9, 54.3, 54.1, 38.0, 29.4, 28.0, 23.6.
IR (neat) ν 3296 (br m), 2969 (m), 2938 (w), 1705 (s), 1471 (m), 1441 (m), 1394 (m), 1366 (m), 1350 (s), 1293 (m), 1278 (m), 1254 (m), 1164 (s), 1141 (s), 1041 (w), 1005 (m), 912 (m), 894 (w), 864 (w), 844 (m), 791 (m), 773 (m), 757 (m), 707 (s), 605 (s), 576 (s) cm^{-1}.
TLC R_f = 0.24 in 9:1 hexanes:EtOAc.
HRMS (ESI) m/z: $[M – H]^–$ Calcd for $C_{14}H_{29}ClN_2O_4S$ 355.1464; Found 355.1461.

N-tert-Butoxycarbonyl-_N'-tert_-butyl-_N_-(3-chlorobut-1-yl)sulfamide (4b)

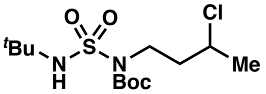

Prepared from chlorosulfamide **1b** (69 mg, 0.2 mmol) according to general procedure I. After irradiating for 15 minutes, the product was isolated as a white solid (64 mg, 93% yield) following purification by silica gel column chromatography (Gradient: 100% hexanes → 20:1 hexanes/EtOAc).

^{1}H NMR (400 MHz, C_6D_6) δ 5.37 (br s, 1H), 3.98–3.91 (m, 1H), 3.85–3.79 (m, 1H), 3.76–3.68 (m, 1H), 2.17–2.09 (m, 1H), 2.05–1.96 (m, 1H), 1.32 (s, 9H), 1.14 (d, *J* = 6.6 Hz, 3H), 1.04 (s, 9H).
^{1}H NMR (400 MHz, $CDCl_3$) δ 5.32 (br s, 1H), 4.09–4.00 (m, 1H), 3.92–3.85 (m, 1H), 3.82–3.75 (m, 1H), 2.18–2.10 (m, 1H), 2.08–1.98 (m, 1H), 1.55 (d, *J* = 6.3 Hz, 3H), 1.54 (s, 9H), 1.31 (s, 9H).
^{13}C NMR (126 MHz, $CDCl_3$) δ 152.0, 83.9, 55.7, 55.1, 45.0, 39.8, 29.4, 28.1, 25.4.
IR (neat) ν 3292 (br), 2978 (w), 2927 (w), 1714 (m), 1474 (w), 1440 (m), 1394 (m), 1365 (m), 1348 (s), 1335 (s), 1301 (m), 1256 (m), 1204 (w), 1163 (s), 1137 (s), 1073 (m), 1043 (m), 993 (m), 956 (m), 872 (m), 850 (m), 810 (m), 767 (m), 714 (s), 684 (m), 601 (s), 576 (s) cm^{-1}.
TLC R_f = 0.22 in 9:1 hexanes:EtOAc.
HRMS (ESI) m/z: $[M – H]^–$ Calcd for $C_{13}H_{27}ClN_2O_4S$ 341.1307; Found 341.1302.

N-tert-Butoxycarbonyl-_N'-tert_-butyl-_N_-(3-chloro-3-methylbut-1-yl)sulfamide (4c)

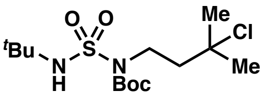

Prepared from chlorosulfamide **1c** (71 mg, 0.2 mmol) according to general procedure I. After irradiating for 1 hour, the product was isolated as a white solid (70 mg, 98% yield) after purification by filtration over a pad of Florisil (EtOAc).

^{1}H NMR (400 MHz, $CDCl_3$) δ 5.32 (br s, 1H), 3.91–3.87 (m, 2H), 2.15–2.11 (m, 2H), 1.61 (s, 6H), 1.54 (s, 9H), 1.32 (s, 9H).
^{13}C NMR (126 MHz, $CDCl_3$) δ 152.0, 83.8, 68.2, 55.1, 44.4, 44.2, 32.5, 29.4, 28.1.
IR (neat) ν 3321 (br), 2970 (w), 1713 (m), 1431 (w), 1394 (w), 1370 (w), 1349 (w), 1297 (m), 1250 (w), 1219 (w), 1161 (m), 1135 (m), 1000 (m), 932 (w), 844 (m), 804 (w), 775 (w), 759 (w), 712 (m), 600 (m), 579 (m) cm^{-1}.
HRMS (ESI) m/z: $[M – H]^-$ Calcd for $C_{14}H_{28}ClN_2O_4S$ 355.1458; Found 355.1464.

***N-tert*-Butoxycarbonyl-*N'*-*tert*-butyl-*N*-(2-(1-chlorocyclopent-1-yl)ethyl)sulfamide (4d)**

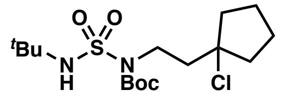

Prepared from chlorosulfamide **1d** (76 mg, 0.2 mmol) according to general procedure I. After irradiating for 2 hours, the product was isolated as a white solid (72 mg, 94% yield) following purification by silica gel column chromatography (9:1 hexanes/EtOAc).

The product was obtained following purification in 94% yield (72 mg) after irradiating for 3 h in ^{i}PrOAc (2.0 mL, 0.1 M) in front of two 26W CFL bulbs (1600 lumens).

^{1}H NMR (400 MHz, $CDCl_3$) δ 5.32 (br s, 1H), 3.96–3.92 (m, 2H), 2.24–2.20 (m 2H), 2.15–2.08 (m, 2H), 1.97–1.91 (m, 2H), 1.79–1.71 (m, 4H), 1.53 (s, 9H), 1.31 (s, 9H).
^{13}C NMR (126 MHz, $CDCl_3$) δ 152.1, 83.7, 80.0, 55.1, 44.9, 42.4, 42.3, 29.4, 28.1, 23.0.
IR (neat) ν 3325 (br w), 2974 (w), 1711 (m), 1426 (w), 1393 (w), 1355 (m), 1290 (w), 1247 (w), 1164 (m), 1142 (m), 1068 (w), 982 (w), 850 (w), 775 (w), 716 (w), 622 (w), 574 (w) cm^{-1}.
TLC R_f = 0.29 in 9:1 hexanes:EtOAc.
HRMS (ESI) m/z: $[M + Na]^+$ Calcd for $C_{16}H_{31}ClN_2O_4S$•Na 405.1585; Found 405.1584.

***N-tert*-Butoxycarbonyl-*N'*-*tert*-butyl-*N*-(2-(1-chlorocyclohex-1-yl)ethyl)sulfamide (4e)**

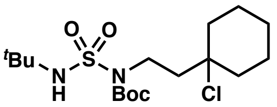

Prepared from chlorosulfamide **1e** (79 mg, 0.2 mmol) according to general procedure I. After irradiating for 15 minutes, the product was isolated as a white solid (75 mg, 95% yield) following purification by silica gel column chromatography (Gradient: 100% hexanes → 9:1 hexanes/EtOAc).

^{1}H NMR (400 MHz, $CDCl_3$) δ 5.32 (br s, 1H), 3.94–3.90 (m, 2H), 2.14–2.10 (m, 2H), 1.96–1.91 (m, 2H), 1.73–1.66 (m, 2H), 1.61–1.57 (m, 4H), 1.53 (s, 9H), 1.30 (s, 9H), 1.26–1.20 (m, 2H).
^{13}C NMR (126 MHz, $CDCl_3$) δ 152.1, 83.7, 73.4, 55.1, 43.7, 43.4, 39.7, 29.4, 28.1, 25.3, 22.1.
IR (neat) ν 3221 (br), 2976 (w), 2934 (m), 2864 (w), 1708 (m), 1446 (w), 1425 (w), 1393 (m), 1352 (s), 1300 (m), 1277 (m), 1254 (m), 1229 (m), 1160 (s), 1138 (s), 1040 (w), 977 (m), 849 (m), 798 (w), 774 (m), 713 (s), 612 (m), 589 (m), 569 (m) cm^{-1}.
TLC R_f = 0.45 in 4:1 hexanes:EtOAc.
HRMS (ESI) m/z: $[M + Na]^+$ Calcd for $C_{17}H_{33}ClN_2O_4S$•Na 419.1742; Found 419.1742.

***N-tert*-Butoxycarbonyl-*N'*-*tert*-butyl-*N*-((*2R*)-methyl 4-chloro-4-methylpentano-2-yl)sulfamide (4f)**

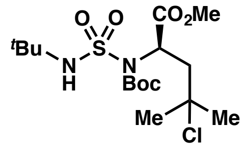

Prepared from chlorosulfamide **1f** (83 mg, 0.2 mmol) according to general procedure I. After irradiating for 1 hour, the product was isolated as a

colorless oil that solidified upon standing (67 mg, 81% yield) after purification on a Teledyne Isco CombiFlashR$_f$ system using a Redi Sep R$_f$ normal phase silica gel column (9:1 hexanes/EtOAc).

^{1}H NMR (400 MHz, CDCl$_3$) δ 5.33 (br s, 1H), 5.09 (t, *J* = 4.4 Hz, 1H), 3.76 (s, 3H), 2.97 (dd, *J* = 15.3, 5.4 Hz, 1H), 2.17 (dd, 15.3, 3.7 Hz, 1H), 1.67 (s, 3H), 1.62 (s, 3H), 1.49 (s, 9H), 1.37 (s, 9H).
^{13}C NMR (101 MHz, CDCl$_3$) δ 170.7, 151.4, 84.7, 69.1, 56.9, 56.1, 52.6, 47.6, 32.7, 32.6, 29.5, 28.0.
IR (neat) ν 3307 (br), 2977 (w), 2360 (w), 2340 (w), 1750 (m), 1718 (m), 1652 (w), 1558 (w), 1540 (w), 1506 (w), 1473 (w), 1456 (w), 1436 (w), 1395 (w), 1370 (m), 1352 (m), 1314 (w), 1249 (w), 1147 (m), 1115 (w), 998 (w), 844 (w), 774 (w), 711 (w), 667 (w), 614 (w), 576 (w) cm^{-1}.
TLC R$_f$ = 0.30 in 8:2 hexanes:EtOAc.
HRMS (ESI) m/z: [M + Na]$^+$ Calcd for $C_{16}H_{31}ClN_2O_6S{\bullet}Na$ 437.1489; Found 437.1492.

***N-tert*-Butoxycarbonyl-*N'*-*tert*-butyl-*N*-((*S*)-2-(1,3-dioxoisoindolin-2-yl)-3-chloro-3-methylbutyl)sulfamide (4g)**

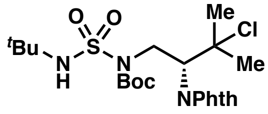

Prepared from chlorosulfamide **1g** (100 mg, 0.2 mmol) according to general procedure I. After irradiating for 15 minutes, the product was isolated a white solid (85 mg, 85% yield) following purification by silica gel column chromatography (Gradient: 100% hexanes → 9:1 hexanes/EtOAc).

^{1}H NMR (400 MHz, CDCl$_3$) δ 7.88–7.83 (m, 2H), 7.71–7.69 (m, 2H), 5.26 (br s, 1H), 4.92 (dd, *J* = 2.2, 10.8 Hz, 1H), 4.78 (dd, *J* = 10.8, 14.5 Hz, 1H), 4.19 (dd, *J* = 2.2, 14.5 Hz, 1H), 1.85 (s, 3H), 1.67 (s, 3H), 1.56 (s, 9H), 1.23 (s, 9H).
^{13}C NMR (126 MHz, CDCl$_3$) δ 168.5, 168.2, 151.7, 134.1, 133.8, 132.0, 131.4, 123.5, 123.3, 84.7, 69.8, 59.1, 55.1, 44.3, 31.3, 30.4, 29.3, 28.1.
IR (neat) ν 3308 (br), 2977 (w), 2935 (w), 1780 (w), 1717 (s), 1468 (w), 1428 (w), 1393 (m), 1356 (m), 1334 (m), 1249 (w), 1164 (m), 1140 (m), 1074 (w), 1042 (w), 991 (w), 957 (w), 876 (w), 848 (w), 794 (w), 774 (w), 721 (m), 710 (m), 604 (w), 577 (w), 531 (w) cm^{-1}.
TLC R$_f$ = 0.16 in 4:1 hexanes:EtOAc.
HRMS (ESI) m/z: [M + Na]$^+$ Calcd for $C_{22}H_{32}ClN_3O_6S{\bullet}Na$ 524.1593; Found 524.1591.

***N-tert*-Butoxycarbonyl-*N'*-*tert*-butyl-*N*-(3-chloro-3,7-dimethyloct-1-yl)sulfamide (4h)**

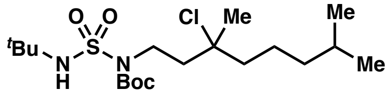

Prepared from chlorosulfamide **1h** (85 mg, 0.2 mmol) according to general procedure I. After irradiating for 15 minutes, the product was isolated as a colorless oil (79 mg, 92% yield) after purification on a Teledyne Isco CombiFlashR$_f$ system using a Redi Sep R$_f$ normal phase silica gel column (9:1 hexanes/EtOAc).

The product was obtained following purification in 95% yield (81 mg) after irradiating for 2 h in ^{i}PrOAc (2.0 mL, 0.1 M) in front of two 26W CFL bulbs (1600 lumens).

^{1}H NMR (400 MHz, $CDCl_3$) δ 5.32 (br s, 1H), 3.90–3.86 (m, 2H), 2.20–2.13 (m, 1H), 2.10–2.03 (m, 1H), 1.76–1.68 (m, 2H), 1.55 (s, 3H), 1.54 (s, 9H), 1.48–1.42 (m, 2H), 1.31 (s, 9H), 1.21–1.15 (m, 3H), 0.88 (d, J = 6.6 Hz, 6H).
^{13}C NMR (126 MHz, $CDCl_3$) δ 152.0, 83.7, 72.2, 55.1, 44.6, 44.0, 42.6, 38.9, 29.6, 29.4, 28.1, 27.7, 22.5, 22.5, 22.2.
IR (neat) ν 3340 (br), 2953 (w), 1709 (m), 1425 (w), 1392 (w), 1351 (m), 1294 (m), 1245 (w), 1139 (s), 986 (w), 912 (w), 848 (w), 802 (w), 773 (w), 713 (m), 606 (m), 576 (m) cm^{-1}.
TLC R_f = 0.49 in 8:2 hexanes/EtOAc.
HRMS (ESI) m/z: $[M - H]^-$ Calcd for $C_{19}H_{38}ClN_2O_4S$ 425.2240; Found 425.2245.

***N-tert*-Butoxycarbonyl-*N'-tert*-butyl-*N*-(3-chloropent-1-yl)sulfamide (4i)**

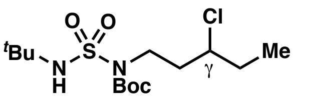

Prepared from chlorosulfamide **1i** (71 mg, 0.2 mmol) according to general procedure I. After irradiating for 15 minutes, the product was isolated as a colorless oil (53 mg, 75% yield) after purification on a Teledyne Isco CombiFlashR$_f$ system using a Redi Sep R$_f$ normal phase silica gel column (Gradient: 100% hexanes → 8:2 hexanes/Et_2O).

In order to determine the ratio of γ-chlorinated:δ-chlorinated isomers, duplicate reactions of chlorosulfamide **1i** (11 mg, 0.03 mmol) were subjected to general procedure I. After irradiating for 15 minutes, each sample was concentrated under reduced pressure and 1,2,4,5-tetrachloro-3-nitrobenzene (1.0 equiv) was added as an internal standard. ^{1}H NMR analysis of the crude reaction mixtures revealed a 8.8:1.0 mixture of γ-chlorinated:δ-chlorinated isomers as an average of 2 runs.
Sample 1: 88% yield γ-chlorinated **4i**, 8% yield δ-chlorinated **7i**, 7% yield protodehalogenated **S8i**.
Sample 2: 85% yield γ-chlorinated **4i**, 9% yield δ-chlorinated **7i**, 5% yield protodehalogenated **S8i**.

^{1}H NMR (400 MHz, $CDCl_3$) δ 5.32 (br s, 1H), 3.95–3.75 (m, 3H), 2.20–2.12 (m, 1H), 2.08–1.98 (m, 1H), 1.85–1.69 (m, 2H), 1.54 (s, 9H), 1.30 (s, 9H), 1.03 (t, J = 7.3 Hz, 3H).
^{13}C NMR (126 MHz, $CDCl_3$) δ 152.1, 83.9, 62.4, 55.1, 45.1, 37.7, 31.6, 29.4, 28.1, 10.8.
IR (neat) ν 3318 (br), 2973 (w), 1707 (m), 1392 (m), 1351 (m), 1286 (m), 1252 (m), 1138 (s), 986 (m), 911 (w), 847 (m), 793 (w), 773 (w), 712 (s), 603 (m), 577 (m) cm^{-1}.
TLC R_f = 0.54 in 8:2 hexanes/EtOAc.
HRMS (ESI) m/z: $[M + Na]^+$ Calcd for $C_{14}H_{29}ClN_2O_4S{\bullet}Na$ 379.1434; Found 379.1428.

***N-tert*-Butoxycarbonyl-*N'-tert*-butyl-*N*-(3-chlorohex-1-yl)sulfamide (4j)**

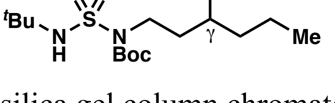

Prepared from chlorosulfamide **1j** (74 mg, 0.2 mmol) according to general procedure I. After irradiating for 15 minutes, the product was isolated as a white solid (62 mg, 84% yield) following purification by silica gel column chromatography (Gradient: 100% hexanes → 20:1 hexanes/EtOAc).

In order to determine the ratio of γ-chlorinated:δ-chlorinated isomers, duplicate reactions of chlorosulfamide **1j** (11 mg, 0.03 mmol) were subjected to general procedure I. After irradiating for 15 minutes, each sample was concentrated under reduced pressure and 1,2,4,5-tetrachloro-3-nitrobenzene (1.0 equiv) was added as an internal standard. ^{1}H NMR analysis of the crude

reaction mixtures revealed a 14.0:1.0 mixture of γ-chlorinated:δ-chlorinated isomers as an average of 2 runs.
Sample 1: 84% yield γ-chlorinated **4j**, 6% yield δ-chlorinated **7j**, 9% yield protodehalogenated **S8j**.
Sample 2: 84% yield γ-chlorinated **4j**, 6% yield δ-chlorinated **7j**, 8% yield protodehalogenated **S8j**.

^{1}H NMR (400 MHz, $CDCl_3$) δ 5.32 (br s, 1H), 3.95–3.88 (m, 2H), 3.83–3.75 (m, 1H), 2.20–2.12 (m, 1H), 2.07–1.98 (m, 1H), 1.75–1.69 (m, 2H), 1.54 (s, 9H), 1.49–1.40 (m, 2H), 1.30 (s, 9H), 0.92 (t, J = 7.3 Hz, 3H).
^{13}C NMR (101 MHz, $CDCl_3$) δ 152.0, 83.8, 60.6, 55.1, 45.0, 40.5, 38.0, 29.4, 28.1, 19.5, 13.4.
IR (neat) ν 3316 (br w), 2969 (w), 2874 (w), 1708 (m), 1426 (w), 1393 (m), 1352 (s), 1291 (m), 1256 (m), 1139 (s), 1042 (w), 986 (m), 848 (m), 810 (w), 772 (m), 713 (s), 604 (m), 578 (s) cm^{-1}.
TLC R_f = 0.22 in 9:1 hexanes:EtOAc.
HRMS (ESI) m/z: $[M – H]^-$ Calcd for $C_{15}H_{31}ClN_2O_4S$ 369.1620; Found 369.1617.

***N-tert*-Butyoxycarbonyl-*N'*-*tert*-butyl-*N*-(methyl 4-chlorohexano-6-yl)sulfamide (4k)**

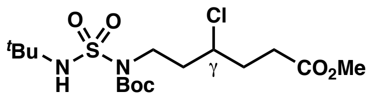

Prepared from chlorosulfamide **1k** (83 mg, 0.2 mmol) according to general procedure I. After irradiating for 15 minutes, the product was isolated as a colorless oil (69 mg, 83% yield) following purification by silica gel column chromatography (Gradient: 100% hexanes → 9:1 hexanes/EtOAc).

^{1}H NMR (400 MHz, $CDCl_3$) δ 5.31 (br s, 1H), 3.99–3.87 (m, 2H), 3.83–3.76 (m, 1H), 3.68 (s, 3H), 2.61–2.49 (m, 2H), 2.21–1.95 (m, 4H), 1.53 (s, 9H), 1.30 (s, 9H).
^{13}C NMR (126 MHz, $CDCl_3$) δ 173.1, 152.0, 84.0, 59.7, 55.2, 51.7, 44.8, 38.1, 33.4, 30.8, 29.4, 28.1.
IR (neat) ν 3315 (br), 2976 (w), 1711 (m), 1436 (w), 1393 (w), 1352 (m), 1290 (m), 1255 (m), 1139 (s), 989 (m), 916 (w), 848 (m), 792 (w), 773 (w), 712 (m), 604 (m), 576 (m) cm^{-1}.
TLC R_f = 0.29 in 4:1 hexanes:EtOAc.
HRMS (ESI) m/z: $[M + NH_4]^+$ Calcd for $C_{16}H_{31}ClN_2O_6S•NH_4$ 432.1930; Found 432.1930.

***N*-Acetyl-*N'*-*tert*-butyl-*N*-(3-chlorohex-1-yl)sulfamide (4l)**

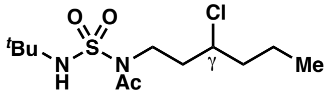

Prepared from chlorosulfamide **1l** (63 mg, 0.2 mmol) according to general procedure I. After irradiating for 15 minutes, the product was isolated as a colorless oil (55 mg, 87% yield) following purification by silica gel column chromatography (Gradient: 100% hexanes → 20:1 hexanes/EtOAc).

In order to determine the ratio of γ-chlorinated:δ-chlorinated isomers, duplicate reactions of chlorosulfamide **1l** (9 mg, 0.03 mmol) were subjected to general procedure I. After irradiating for 15 minutes, each sample was concentrated under reduced pressure and 1,2,4,5-tetrachloro-3-nitrobenzene (1.0 equiv) was added as an internal standard. ^{1}H NMR analysis of the crude reaction mixtures revealed a 22.5:1.0 mixture of γ-chlorinated:δ-chlorinated isomers as an average of 2 runs.
Sample 1: 89% yield γ-chlorinated **4l**, 4% yield δ-chlorinated **7l**.
Sample 2: 93% yield γ-chlorinated **4l**, 4% yield δ-chlorinated **7l**.

^{1}H NMR (400 MHz, $CDCl_3$) δ 5.42 (br s, 1H), 3.99–3.92 (m, 2H), 3.90–3.82 (m, 1H), 2.33 (s, 3H), 2.30–2.23 (m, 1H), 2.06–1.97 (m, 1H), 1.77–1.71 (m, 2H), 1.55–1.42 (m, 2H), 1.31 (s, 9H), 0.98 (t, J = 7.4 Hz, 3H).
^{13}C NMR (126 MHz, $CDCl_3$) δ 171.1, 60.7, 55.5, 45.5, 40.5, 38.2, 29.5, 23.9, 19.5, 13.4.
IR (neat) ν 3290 (br), 2962 (w), 2874 (w), 1673 (m), 1426 (m), 1394 (m), 1348 (s), 1265 (m), 1232 (m), 1154 (s), 1098 (w), 1039 (w), 1001 (m), 927 (m), 853 (m), 771 (m), 736 (s), 702 (m), 663 (m), 605 (s), 546 (s) cm^{-1}.
TLC R_f = 0.22 in 4:1 hexanes:EtOAc.
HRMS (ESI) m/z: $[M – H]^-$ Calcd for $C_{12}H_{25}ClN_2O_3S$ 311.1202; Found 311.1195.

***N-tert*-Butyl-*N'*-(3-chlorohex-1-yl)-*N'*-(2,2,2-trifluoroethyl)sulfamide (4m)**

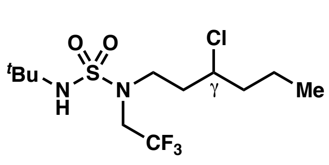

Prepared from chlorosulfamide **1m** (71 mg, 0.2 mmol) according to general procedure I. After irradiating for 15 minutes, the product was isolated as a colorless oil (69 mg, 97% yield) following purification by silica gel column chromatography (Gradient: 100% hexanes → 20:1 hexanes/EtOAc).

^{1}H NMR (400 MHz, $CDCl_3$) δ 4.00 (br s, 1H), 3.94–3.88 (m, 1H), 3.81 (qd, J = 8.8, 2.7 Hz, 2H), 3.59–3.42 (m, 2H), 2.21–2.14 (m, 1H), 2.04–1.95 (m, 1H), 1.74–1.69 (m, 2H), 1.59–1.42 (m, 2H), 1.35 (s, 9H), 0.93 (t, J = 7.4 Hz, 3H).
^{13}C NMR (126 MHz, $CDCl_3$) δ 124.4 (q, J = 281.1 Hz), 60.7, 55.1, 49.0 (q, J = 34.2 Hz), 47.4, 36.1, 29.6, 19.5, 13.4.
^{19}F NMR (376 MHz, $CDCl_3$) δ –69.71 (t, J = 8.9 Hz).
IR (neat) ν 3288 (br), 2963 (w), 2876 (w), 1465 (w), 1429 (w), 1393 (w), 1369 (w), 1317 (m), 1272 (m), 1231 (w), 1140 (s), 1081 (m), 1042 (m), 981 (m), 929 (w), 908 (w), 870 (m), 832 (w), 740 (m), 665 (m), 604 (m), 566 (m) cm^{-1}.
TLC R_f = 0.20 in 9:1 hexanes:EtOAc.
HRMS (ESI) m/z: $[M – H]^-$ Calcd for $C_{12}H_{24}ClF_3N_2O_2S$ 351.1126; Found 351.1118.

***N'-tert*-Butyl-*N*-(3-chloropent-1-yl)-*N*-trifluoroacetylsulfamide (4n)**

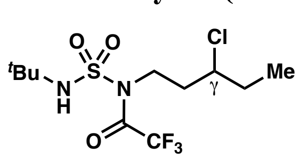

Prepared from chlorosulfamide **1n** (71 mg, 0.2 mmol) according to general procedure I at 0.013 M. After irradiating for 15 minutes, the product was isolated as a colorless oil (45 mg, 63% yield) after purification on a Teledyne Isco CombiFlashR$_f$ system using a Redi Sep R$_f$ normal phase silica gel column (95:5 hexanes/EtOAc).

^{1}H NMR (400 MHz, $CDCl_3$) δ 5.57 (br s, 1H), 4.15 (ddd, J = 15.9, 11.1, 5.3 Hz, 1H), 3.91–3.82 (m, 2H), 2.29–2.11 (m, 2H), 1.86–1.73 (m, 2H), 1.32 (s, 9H), 1.05 (t, J = 7.3 Hz, 3H).
^{13}C NMR (126 MHz, $CDCl_3$) δ 156.2 (q, J = 38.6 Hz), 115.0 (q, J = 274.6 Hz), 61.6, 56.1, 45.4, 37.7, 31.3, 29.1, 10.5.
^{19}F NMR (376 MHz, $CDCl_3$) δ –70.75.
IR (neat) ν 3311 (br), 2975 (w), 1705 (m), 1431 (w), 1396 (m), 1364 (m), 1320 (w), 1227 (m), 1210 (m), 1167 (s), 1081 (m), 1059 (m), 997 (m), 862 (w), 791 (w), 758 (m), 691 (m), 612 (s), 580 (m), 529 (m) cm^{-1}.
TLC R_f = 0.49 in 8:2 hexanes/EtOAc.

HRMS (ESI) m/z: $[M - H]^-$ Calcd for $C_{11}H_{19}ClF_3N_2O_3S$ 351.0757; Found 351.0765.

***N-tert*-Butoxycarbonyl-*N*-(3-chlorohex-1-yl)-*N'*-(2,2,2-trfiluoroethyl)sulfamide (4o)**

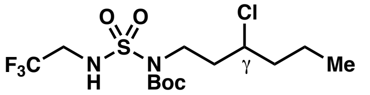

Prepared from chlorosulfamide **1o** (74 mg, 0.19 mmol) according to general procedure I. After irradiating for 15 minutes, the product was isolated as a colorless oil (58 mg, 79% yield) following purification by silica gel column chromatography (Gradient: 100% hexanes → 20:1 hexanes/EtOAc).

In order to determine the ratio of γ-chlorinated:δ-chlorinated isomers, duplicate reactions of chlorosulfamide **1o** (12 mg, 0.03 mmol) were subjected to general procedure I. After irradiating for 15 minutes, each sample was concentrated under reduced pressure and 1,2,4,5-tetrachloro-3-nitrobenzene (1.0 equiv) was added as an internal standard. ^{1}H NMR analysis of the crude reaction mixtures revealed a 10.5:1.0 mixture of γ-chlorinated:δ-chlorinated isomers as an average of 2 runs.
Sample 1: 77% yield γ-chlorinated **4o**, 7% yield δ-chlorinated **7o**, 14% protodehalogenated **S8o**.
Sample 2: 79% yield γ-chlorinated **4o**, 8% yield δ-chlorinated **7o**, 20% protodehalogenated **S8o**.

^{1}H NMR (400 MHz, $CDCl_3$) δ 5.82 (t, J = 6.9 Hz, 1H), 3.93–3.86 (m, 2H), 3.80–3.74 (m, 1H), 3.70–3.64 (m, 2H), 2.14–2.05 (m, 1H), 2.05–1.94 (m, 1H), 1.74–1.67 (m, 2H), 1.52 (s, 9H), 1.47–1.39 (m, 2H), 0.91 (t, J = 7.3 Hz, 3H).
^{13}C NMR (126 MHz, $CDCl_3$) δ 151.6, 123.4 (q, J = 278.3 Hz), 84.9, 60.2, 45.7, 45.2 (q, J = 36.0 Hz), 40.5, 38.1, 27.9, 19.5, 13.4.
^{19}F NMR (376 MHz, $CDCl_3$) δ –71.93 (t, J = 9.0 Hz).
IR (neat) ν 3309 (br), 2964 (w), 1712 (m), 1456 (w), 1371 (m), 1265 (m), 1144 (s), 963 (w), 832 (w), 734 (s), 704 (s), 664 (m), 583 (m), 550 (m) cm^{-1}.
TLC R_f = 0.21 in 9:1 hexanes/EtOAc.
HRMS (ESI) m/z: $[M - H]^-$ Calcd for $C_{13}H_{24}ClF_3N_2O_4S$ 395.1025; Found 395.1015.

***N-tert*-Butyl-*N'*-(3-chloro-4-methylpentyl)-*N'*-(2,2,2-trifluoroethyl)sulfamide (4p)**

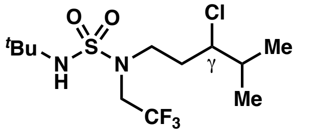

Prepared from chlorosulfamide **1p** (71 mg, 0.2 mmol) according to general procedure I. After irradiating for 15 minutes, the product was isolated as a colorless oil (50 mg, 70% yield) following purification by silica gel column chromatography (Gradient: 100% hexanes → 50:1 hexanes/EtOAc).

In order to determine the ratio of γ-chlorinated:δ-chlorinated isomers, duplicate reactions of chlorosulfamide **1p** (11 mg, 0.03 mmol) were subjected to general procedure I. After irradiating for 15 minutes, each sample was concentrated under reduced pressure and 2-bromo-5-(trifluoromethyl)pyridine (1.0 equiv) was added as an internal standard. ^{19}F NMR analysis of the crude reaction mixtures revealed a 13:1.0 mixture of γ-chlorinated:δ-chlorinated isomers as an average of 2 runs.
Sample 1: 64% yield γ-chlorinated **4p**, 6% yield δ-chlorinated **7p**, 7% yield of β-chlorinated **S11p**, and 14% yield of protodehalogenated **S8p**.
Sample 2: 66% yield γ-chlorinated **4p**, 5% yield δ-chlorinated **7p**, 7% yield of β-chlorinated **S11p**, and 10% yield of protodehalogenated **S8p**.

^{1}H NMR (400 MHz, $CDCl_3$) δ 4.11 (br s, 1H), 3.84–3.78 (m, 3H), 3.56 (ddd, *J* = 14.8, 9.9, 4.9 Hz, 1H), 3.44 (ddd, *J* = 15.4, 9.9, 6.1 Hz, 1H), 2.17–2.09 (m, 1H), 2.04–1.91 (m, 2H), 1.34 (s, 9H), 1.02 (d, *J* = 6.7 Hz, 3H), 0.99 (d, *J* = 6.7 Hz, 3H).
^{13}C NMR (126 MHz, $CDCl_3$) δ 124.4 (q, *J* = 280.7 Hz), 67.2, 55.1, 49.0 (q, *J* = 33.8 Hz), 34.6, 33.6, 29.7, 19.7, 17.4.
^{19}F NMR (376 MHz, $CDCl_3$) δ –69.70 (t, *J* = 9.0 Hz).
IR (neat) ν 3289 (br), 2969 (w), 1464 (w), 1426 (w), 1392 (m), 1369 (w), 1323 (m), 1272 (m), 1229 (w), 1141 (s), 1084 (m), 1043 (m), 981 (m), 928 (w), 906 (w), 870 (w), 823 (w), 738 (s), 704 (w), 664 (m), 604 (m), 561 (m) cm^{-1}.
TLC R_f = 0.32 in 4:1 hexanes:EtOAc.
HRMS (ESI) m/z: $[M – H]^-$ Calcd for $C_{12}H_{24}ClF_3N_2O_2S$ 351.1126; Found 351.1118.

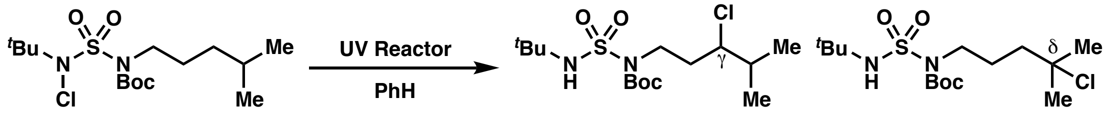

1q **4q** **7q**

Chlorosulfamide **1q** (74 mg, 0.2 mmol) was subjected to general procedure I. After irradiating for 15 minutes, products **4q** and **5q** (73 mg, 98% yield) were isolated as a mixture following purification by silica gel column chromatography (Gradient: 100% hexanes → 9:1 hexanes/EtOAc).

In order to determine the ratio of **4q**:**7q**, duplicate reactions of chlorosulfamide **1q** (11 mg, 0.03 mmol) were subjected to general procedure I. After irradiating for 1 hour, each sample was concentrated under reduced pressure and 1,2,4,5-tetrachloro-3-nitrobenzene (1.0 equiv) was added as an internal standard. ^{1}H NMR analysis of the crude reaction mixtures revealed a 2.0:1.0 mixture of **4q**:**7q** as an average of 2 runs.
Sample 1: 68% yield **4q**, 35% yield **7q**.
Sample 2: 69% yield **4q**, 35% yield **7q**.

***N-tert*-Butoxycarbonyl-*N'-tert*-butyl-*N*-(3-chloro-4-methylpentyl)sulfamide (4q)**

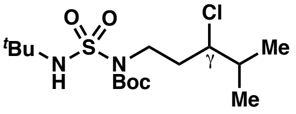

Isolated for characterization by silica gel column chromatography (Gradient: 100% hexanes → 20:1 hexanes/EtOAc) from a mixture of chlorinated isomers (**4q** and **7q**).

^{1}H NMR (400 MHz, $CDCl_3$) δ 5.33 (br s, 1H), 3.93 (ddd, *J* = 14.6, 9.2, 5.0 Hz, 1H), 3.86–3.73 (m, 2H), 2.15–2.00 (m, 2H), 1.99–1.91 (m, 1H), 1.54 (s, 9H), 1.30 (s, 9H), 1.02 (d, *J* = 6.7 Hz, 3H), 0.98 (d, *J* = 6.7 Hz, 3H).
^{13}C NMR (126 MHz, $CDCl_3$) δ 152.1, 83.9, 67.2, 55.1, 45.5, 35.6, 34.5, 29.4, 28.1, 19.7, 17.4.
IR (neat) ν 3313 (br), 2971 (w), 1708 (m), 1426 (w), 1393 (m), 1352 (s), 1292 (m), 1250 (m), 1138 (s), 1042 (w), 986 (m), 848 (m), 772 (m), 713 (s), 631 (m), 604 (m), 578 (m) cm^{-1}.
TLC R_f = 0.47 in 4:1 hexanes:EtOAc.
HRMS (ESI) m/z: $[M – H]^-$ Calcd for $C_{15}H_{31}ClN_2O_4S$ 369.1620; Found 369.1614.

***N-tert*-Butoxycarbonyl-*N'-tert*-butyl-*N*-(4-chloro-4-methylpentyl)sulfamide (7q)**

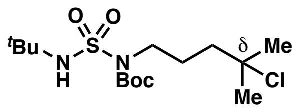

Isolated for characterization by silica gel column chromatography (Gradient: 100% hexanes → 50:1 hexanes/EtOAc) from a mixture of chlorinated isomers (**4q** and **7q**).

^{1}H NMR (400 MHz, $CDCl_3$) δ 5.32 (br s, 1H), 3.68 (t, J = 7.3 Hz, 2H), 1.94–1.86 (m, 2H), 1.78–1.74 (m, 2H), 1.57 (s, 6H), 1.54 (s, 9H), 1.30 (s, 9H).
^{13}C NMR (126 MHz, $CDCl_3$) δ 152.2, 83.6, 70.3, 55.1, 47.3, 42.7, 32.4, 29.4, 28.1, 25.4.
IR (neat) ν 3318 (br), 2974 (w), 2932 (w), 1707 (m), 1457 (w), 1425 (w), 1393 (m), 1368 (m), 1353 (s), 1292 (m), 1257 (m), 1138 (s), 1040 (w), 984 (m), 847 (m), 790 (w), 774 (m), 713 (s), 580 (s) cm^{-1}.
TLC R_f = 0.43 in 4:1 hexanes:EtOAc.
HRMS (ESI) m/z: [M – H]$^-$ Calcd for $C_{15}H_{31}ClN_2O_4S$ 369.1520; Found 369.1615.

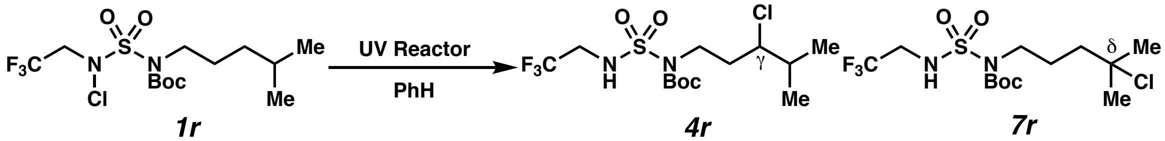

Chlorosulfamide **1r** (79 mg, 0.2 mmol) was subjected to general procedure I. After irradiating for 1 hour, products **4r** and **7r** (66 mg, 84% yield) were isolated as a mixture following purification by silica gel column chromatography (Gradient: 100% hexanes → 50:1 hexanes/acetone).

In order to determine the ratio of **4r**:**7r**, duplicate reactions of chlorosulfamide **1r** (12 mg, 0.03 mmol) were subjected to general procedure I. After irradiating for 1 hour, each sample was concentrated under reduced pressure and 1,2,4,5-tetrachloro-3-nitrobenzene (1.0 equiv) was added as an internal standard. ^{1}H NMR analysis of the crude reaction mixtures revealed a 1.0:1.9 mixture of **4r**:**7r** and 13% protodehalogenated material as an average of two runs.
Sample 1: 30% yield **4r**, 57% yield **7r**, 13% yield protodehalogenated **S8r**.
Sample 2: 30% yield **4r**, 56% yield **7r**, 13% yield protodehalogenated **S8r**.

***N-tert*-Butoxycarbonyl-*N*-(3-chloro-4-methylpentyl)-*N'*-(2,2,2-trifluoroethyl)sulfamide (4r)**

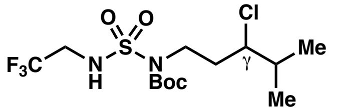

Isolated for characterization by silica gel column chromatography (Gradient: 100% hexanes → 50:1 hexanes/EtOAc) from a mixture of chlorinated isomers (**4r** and **7r**) that had already been purified by the column conditions used to isolate the mixture (Gradient: 100% hexanes → 50:1 hexanes/acetone).

^{1}H NMR (400 MHz, $CDCl_3$) δ 5.86 (t, J = 6.9 Hz, 1H), 3.93 (ddd, J = 14.5, 9.4, 5.1 Hz, 1H), 3.81–3.66 (m, 4H), 2.11–1.99 (m, 2H), 1.98–1.91 (m, 1H), 1.54 (s, 9H), 1.02 (d, J = 6.7 Hz, 3H), 0.99 (d, J = 6.7 Hz, 3H).
^{13}C NMR (126 MHz, $CDCl_3$) δ 151.7, 123.4 (q, J = 278.1 Hz), 85.0, 66.8, 46.2, 45.3 (q, J = 35.6 Hz), 35.6, 34.5, 27.9, 19.7, 17.4.
^{19}F NMR (376 MHz, $CDCl_3$) δ –71.93 (t, J = 8.8 Hz).
IR (neat) ν 3302 (br), 2971 (w), 1706 (m), 1456 (w), 1369 (m), 1342 (m), 1292 (m), 1276 (m), 1140 (s), 962 (m), 831 (m), 772 (m), 718 (s), 664 (m), 625 (m), 582 (m), 549 (m) cm^{-1}.

TLC R_f = 0.41 in 4:1 hexanes:EtOAc.
HRMS (ESI) m/z: $[M - H]^-$ Calcd for $C_{13}H_{24}ClF_3N_2O_4S$ 395.1025; Found 395.1015.

***N-tert*-Butoxycarbonyl-*N*-(4-chloro-4-methylpentyl)-*N'*-(2,2,2-trifluoroethyl)sulfamide (7r)**

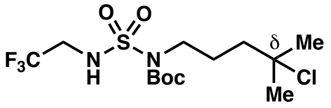

Isolated for characterization by silica gel column chromatography (Gradient: 100% hexanes → 50:1 hexanes/EtOAc) from a mixture of chlorinated isomers (**4r** and **7r**) that had already been purified by the column conditions used to isolate the mixture (Gradient: 100% hexanes → 50:1 hexanes/acetone).

^{1}H NMR (400 MHz, $CDCl_3$) δ 5.84 (t, *J* = 7.0 Hz, 1H), 3.73–3.65 (m, 4H), 1.91–1.83 (m, 2H), 1.76–1.72 (m, 2H), 1.58 (s, 6H), 1.53 (s, 9H).
^{13}C NMR (101 MHz, $CDCl_3$) δ 151.7, 123.4 (q, *J* = 278.8 Hz), 84.7, 70.1, 47.9, 45.2 (q, *J* = 35.4 Hz), 42.5, 32.4, 27.9, 25.5.
^{19}F NMR (376 MHz, $CDCl_3$) δ –71.93 (t, *J* = 8.9 Hz).
IR (neat) ν 3300 (br), 2978 (w), 1707 (m), 1457 (w), 1369 (m), 1340 (m), 1291 (m), 1276 (m), 1141 (s), 1008 (w), 963 (w), 832 (m), 793 (w), 774 (w), 719 (s), 664 (m), 630 (m), 584 (m), 549 (m) cm^{-1}.
TLC R_f = 0.38 in 4:1 hexanes:EtOAc.
HRMS (ESI) m/z: $[M - H]^-$ Calcd for $C_{13}H_{24}ClF_3N_2O_4S$ 395.1025; Found 395.1014.

***N-tert*-Butoxycarbonyl-*N'*-*tert*-butyl-*N*-(*tert*-butyl 2-chlorobutano-4-yl)sulfamide (S10t)**

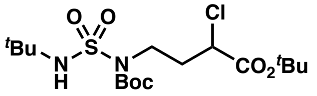

Prepared from chlorosulfamide **S9t** (86 mg, 0.2 mmol) according to general procedure I. After irradiating for 1 hour, the product was isolated as a colorless oil (65 mg, 76% yield) following purification by silica gel column chromatography (Gradient: 100% hexanes → 4:1 CH_2Cl_2/hexanes → 100% CH_2Cl_2).

^{1}H NMR (400 MHz, $CDCl_3$) δ 5.30 (br s, 1H), 4.24 (dd, *J* = 8.5, 4.8 Hz, 1H), 3.84 (t, *J* = 7.4 Hz, 2H), 2.42–2.33 (m, 1H), 2.29–2.22 (m, 1H), 1.54 (s, 9H), 1.49 (s, 9H), 1.30 (s, 9H).
^{13}C NMR (126 MHz, $CDCl_3$) δ 167.8, 151.9, 84.1, 83.0, 55.9, 55.2, 44.1, 34.4, 29.4, 28.1, 27.7.
IR (neat) ν 3318 (br), 2977 (w), 2935 (w), 1710 (m), 1476 (w), 1425 (w), 1393 (w), 1368 (m), 1353 (m), 1293 (m), 1254 (m), 1139 (s), 1040 (w), 988 (w), 912 (w), 845 (m), 773 (w), 711 (m), 604 (m), 576 (m) cm^{-1}.
TLC R_f = 0.17 in 6:4 CH_2Cl_2/hexanes.
HRMS (ESI) m/z: $[M - H]^-$ Calcd for $C_{17}H_{32}ClN_2O_6S$ 427.1669; Found 427.1664.

***N-tert*-Butoxycarbonyl-*N'*-*tert*-butyl-*N*-(3-chloro-3-((4-trifluoromethyl)phenyl)prop-1-yl) sulfamide (S10u)**

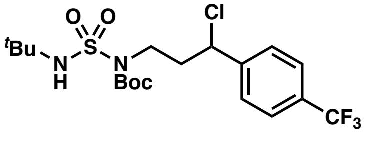

Prepared from chlorosulfamide **S9u** (94 mg, 0.2 mmol) according to general procedure I. After irradiating for 2 hours, the product was isolated as a white solid (46 mg, 49% yield) after two purifications by silica gel column chromatography (100% PhH).

^{1}H NMR (400 MHz, $CDCl_3$) δ 7.63 (d, *J* = 8.3 Hz, 2H), 7.53 (d, *J* = 8.3 Hz, 2H), 5.31 (br s, 1H), 4.94 (dd, *J* = 7.5 Hz, 6.6 Hz, 1H), 3.76–3.92 (m, 2H), 2.41–2.46 (m, 2H), 1.53 (s, 9H), 1.30 (s, 9H).
^{13}C NMR (126 MHz, $CDCl_3$, ^{1}H and ^{19}F decoupled) δ 151.9, 144.8, 130.6, 127.3, 125.8, 123.8, 84.2, 59.5, 55.3, 44.9, 39.3, 29.4, 28.1.
^{19}F NMR (376 MHz, $CDCl_3$) δ –62.73.
IR (neat) ν 3325 (br w), 2977 (w), 1709 (m), 1620 (w), 1422 (w), 1394 (m), 1369 (m), 1353 (m), 1323 (s), 1294 (m), 1257 (m), 1160 (s), 1125 (s), 1092 (w), 1068 (s), 1042 (w), 1017 (w), 990 (w), 845 (m), 774 (w), 714 (m), 626 (w), 603 (m), 579 (w) cm^{-1}.
TLC R_f = 0.24 in 9:1 hexanes:EtOAc.
HRMS (ESI) m/z: $[M + Na]^+$ Calcd for $C_{19}H_{28}ClF_3N_2O_4S$•Na 495.1303; Found 495.1298.

N-tert-Butoxycarbonyl-_N_-(3-chloropent-1-yl)-_N'_-(2,2,2-trfiluoroethyl)sulfamide (S10v)

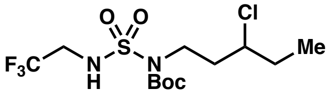

Prepared from chlorosulfamide **S9v** (77 mg, 0.2 mmol) according to general procedure I. After irradiating for 30 minutes, the product was isolated as a colorless oil (50 mg, 65% yield) after purification on a Teledyne Isco CombiFlashR_f system using a Redi Sep R_f normal phase silica gel column (9:1 hexanes/EtOAc).

^{1}H NMR (400 MHz, $CDCl_3$) δ 5.92 (t, *J* = 7.1 Hz, 1H), 3.94–3.65 (m, 5H), 2.15–1.95 (m, 2H), 1.84–1.69 (m, 2H), 1.53 (s, 9H), 1.03 (t, *J* = 7.3 Hz, 3H).
^{13}C NMR (126 MHz, $CDCl_3$) δ 151.6, 123.4 (q, *J* = 278.7 Hz), 84.9, 62.1, 45.2 (q, *J* = 35.1 Hz), 37.7, 31.6, 27.9, 10.8.
^{19}F NMR (376 MHz, $CDCl_3$) δ –71.95 (t, *J* = 8.9 Hz).
IR (neat) ν 3302 (br), 2976 (w), 1707 (m), 1368 (m), 1277 (m), 1140 (s), 1045 (w), 831 (m), 795 (m), 773 (m), 718 (s), 663 (m) cm^{-1}.
TLC R_f = 0.36 in 8:2 hexanes/EtOAc.
HRMS (ESI) m/z: $[M + NH_4]^+$ Calcd for $C_{12}H_{22}ClF_3N_2O_4S$•NH_4 400.1284; Found 400.1269.

N-tert-Butoxycarbonyl-_N_-(3-chloropent-1-yl)-_N'_-ethylsulfamide (S10w)

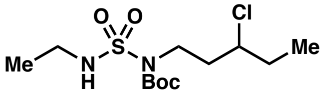

Prepared from chlorosulfamide **S9w** (66 mg, 0.2 mmol) according to general procedure I. After irradiating for 45 minutes, the product was isolated as a colorless oil (29 mg, 44% yield) after purification on a Teledyne Isco CombiFlashR_f system using a Redi Sep R_f normal phase silica gel column (9:1 hexanes/EtOAc).

^{1}H NMR (400 MHz, $CDCl_3$) δ 5.24 (t, *J* = 6.2 Hz, 1H), 3.95–3.74 (m, 3H), 3.03 (qd, *J* = 7.2, 5.9 Hz, 2H), 2.16–1.96 (m, 2H), 1.85–1.68 (m, 2H), 1.53 (s, 9H), 1.19 (t, *J* = 7.3 Hz, 3H), 1.03 (t, *J* = 7.3 Hz, 3H).
^{13}C NMR (126 MHz, $CDCl_3$) δ 151.6, 83.9, 62.0, 45.1, 38.7, 37.6, 31.3, 27.8, 14.1, 10.6.
IR (neat) ν 3310 (br), 2975 (w), 2937 (w), 1705 (m), 1345 (m), 1286 (m), 1251 (m), 1137 (s), 1063 (m), 846 (m), 794 (m), 773 (m), 714 (s) cm^{-1}.
TLC R_f = 0.33 in 8:2 hexanes/EtOAc.
HRMS (ESI) m/z: $[M + Na]^+$ Calcd for $C_{12}H_{25}ClN_2O_4S$•Na 351.1121; Found 351.1110.

VIII. Preparation and characterization of non-γ-chlorinated sulfamides.

***N-tert*-Butoxycarbonyl-*N'*-*tert*-butyl-*N*-(4-chloropent-1-yl)sulfamide (7i)**

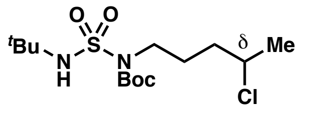

Prepared from sulfamide **S5a** (268 mg, 1.1 mmol) and 4-chloropentanol[6] (100 mg, 0.82 mmol) following general procedure F. The product was obtained as a colorless oil (216 mg, 44% yield) following purification on a Teledyne Isco CombiFlashR$_f$ system using a Redi Sep R$_f$ normal phase silica gel column (Gradient: 100% hexanes → 9:1 hexanes/EtOAc).

^{1}H NMR (400 MHz, $CDCl_3$) δ 5.30 (br s, 1H), 4.09–4.01 (m, 1H), 3.68 (t, *J* = 3.7 Hz, 2H), 1.94–1.89 (m, 1H), 1.85–1.71 (m, 3H), 1.53 (s, 9H), 1.52 (d, *J* = 10.5 Hz, 3H), 1.30 (s, 9H).
^{13}C NMR (101 MHz, $CDCl_3$) δ 152.0, 83.5, 57.9, 54.9, 46.6, 37.0, 29.3, 28.0, 26.6, 25.2.
IR (neat) ν 3293 (br), 2975 (m), 2933 (w), 2872 (w), 1699 (s), 1396 (m), 1369 (m), 1346 (s), 1307 (m), 1287 (m), 1254 (m), 1203 (m), 1164 (s), 1135 (s), 1072 (m), 1043 (m), 1001 (m), 869 (w), 843 (m), 794 (m), 769 (m), 711 (s), 677 (m), 642 (s) cm^{-1}.
TLC R$_f$ = 0.51 in 8:2 hexanes:EtOAc.
HRMS (ESI) m/z: [M – H]$^-$ Calcd for $C_{14}H_{28}ClN_2O_4S$ 355.1458; Found 355.1457.

***N-tert*-Butyl-*N'*-(4-chlorohex-1-yl)-*N'*-(2,2,2-trifluoroethyl)sulfamide (7m)**

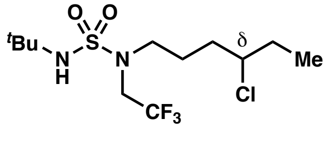

Prepared from *N*-(*tert*-butyl)-*N*'-(2,2,2-trifluoroethyl)sulfamide[11] (152 mg, 0.65 mmol) and **S1** (68 mg, 0.5 mmol, following general procedure F. The product was obtained as a colorless oil (100 mg, 57% yield) after purification by silica gel column chromatography (Gradient: 100% hexanes → 20:1 hexanes/EtOAc).

^{1}H NMR (400 MHz, $CDCl_3$) δ 4.02 (br s, 1H), 3.88–3.78 (m, 3H), 3.32 (t, *J* = 7.5 Hz, 2H), 1.99–1.89 (m, 1H), 1.83–1.66 (m, 5H), 1.34 (s, 9H), 1.03 (t, *J* = 7.3 Hz, 3H).
^{13}C NMR (126 MHz, $CDCl_3$) δ 124.4 (q, *J* = 280.6 Hz), 64.9, 54.9, 48.7, 48.1 (q, *J* = 34.5 Hz), 34.9, 31.5, 29.7, 24.1, 10.9.
^{19}F NMR (376 MHz, $CDCl_3$) δ –69.41 (t, *J* = 8.9 Hz).
IR (neat) ν 3288 (br), 2972 (w), 1464 (w), 1429 (w), 1393 (w), 1369 (w), 1315 (m), 1273 (m), 1234 (w), 1141 (s), 1082 (w), 1042 (m), 992 (s), 909 (w), 871 (m), 831 (w), 771 (m), 733 (m), 662 (m), 603 (m), 565 (m) cm^{-1}.
TLC R$_f$ = 0.43 in 4:1 hexanes:EtOAc.
HRMS (ESI) m/z: [M – H]$^-$ Calcd for $C_{12}H_{24}ClF_3N_2O_2S$ 351.1126; Found 351.1118.

***N-tert*-Butoxycarbonyl-*N*-(4-chlorohex-1-yl)-*N'*-(2,2,2-trifluoroethyl)sulfamide (7o)**

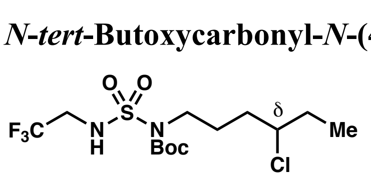

Prepared from sulfamide **S5b** (181 mg, 0.65 mmol) and **S1** (68 mg, 0.5 mmol) following general procedure F. The product was obtained as a colorless oil (133 mg, 67% yield) after purification by silica gel column chromatography (20:1 hexanes:EtOAc).

^{1}H NMR (400 MHz, $CDCl_3$) δ 5.84 (t, *J* = 6.6 Hz, 1H), 3.88–3.82 (m, 1H), 3.72–3.64 (m, 4H), 1.95–1.87 (m, 1H), 1.81–1.69 (m, 5H), 1.53 (s. 9H), 1.03 (t, *J* = 7.3 Hz, 3H).
^{13}C NMR (126 MHz, $CDCl_3$) δ 151.7, 123.4 (q, *J* = 279.1 Hz), 84.7, 64.8, 47.5, 45.2 (q, *J* = 35.5 Hz), 34.8, 31.5, 27.9, 26.7, 10.8.

^{19}F NMR (376 MHz, $CDCl_3$) δ –71.95 (t, *J* = 9.0 Hz).
IR (neat) ν 3300 (br), 2975 (w), 1706 (m), 1456 (w), 1369 (m), 1276 (m), 1140 (s), 993 (w), 963 (m), 912 (w), 832 (m), 774 (m), 718 (s), 664 (m), 630 (m), 582 (m), 549 (m) cm^{-1}.
TLC R_f = 0.18 in 9:1 hexanes:EtOAc.
HRMS (ESI) m/z: $[M – H]^-$ Calcd for $C_{13}H_{24}ClF_3N_2O_4S$ 395.1025; Found 395.1017.

***N*-*tert*-Butyl-*N*'-(4-chloro-4-methylpentyl)-*N*'-(2,2,2-trifluoroethyl)sulfamide** (**7p**).

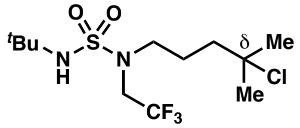

Isolated for characterization by silica gel column chromatography (Gradient: 100% hexanes → 50:1 hexanes:EtOAc) from a mixture of chlorinated isomers (**4p**, **7p** and **S11p**).

^{1}H NMR (400 MHz, $CDCl_3$) δ 3.97 (br s, 1H), 3.83 (q, J = 3.9 Hz, 2H), 3.33 (dd, J = 7.7, 7.7 Hz, 2H), 1.92–1.84 (m, 2H), 1.74–1.70 (m, 2H), 1.59 (s, 6H), 1.35 (s, 9H).
^{13}C NMR (126 MHz, $CDCl_3$) δ 124.5 (q, *J* = 278.3 Hz), 70.2, 55.0, 49.1, 48.1, (q, *J* = 33.7 Hz), 42.6, 32.4, 29.8, 22.9.
^{19}F NMR(376 MHz, $CDCl_3$) δ –69.41 (t, *J* = 9.0 Hz).
IR (neat) ν 3290 (br), 2974 (w), 2922 (w), 1428 (w), 1393 (w), 1370 (w), 1320 (m), 1273 (m), 1227 (w), 1141 (s), 1068 (m), 990 (m), 907 (w), 870 (w), 831 (w), 770 (w), 734 (w), 664 (m), 604 (m), 570 (m), 518 (w) cm^{-1}.
TLC R_f = 0.42 in 4:1 hexanes:EtOAc.
HRMS (ESI) m/z: $[M + H]^+$ Calcd for $C_{12}H_{24}ClF_3N_2O_2S$ 353.1272; Found 353.1274.

***N*-*tert*-Butyl-*N*'-(2-chloro-4-methylpentyl)-*N*'-(2,2,2-trifluoroethyl)sulfamide** (**S11p**).

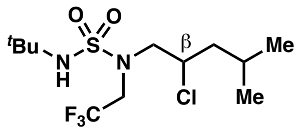

Prepared from *N*-(*tert*-butyl)-*N*'-(2,2,2-trifluoroethyl)sulfamide (111 mg, 0.48 mmol) and 2-chloro-4-methylpentanol (50 mg, 0.37 mmol, following general procedure F. The product was obtained as a colorless oil (70 mg, 53% yield) after purification by silica gel column chromatography (Gradient: 100% hexanes → 50:1 hexanes:EtOAc).

^{1}H NMR (400 MHz, $CDCl_3$) δ 4.40–4.35 (m, 1H), 4.10–3.97 (m, 3H), 3.70 (dd, J = 15.6, 4.2 Hz, 1H), 3.40 (dd, J = 15.6, 9.0 Hz, 1H), 1.95–1.87 (m, 1H), 1.65–1.59 (m, 1H), 1.52–1.47 (m, 1H), 1.36 (s, 9H), 0.96 (d, *J* = 6.6 Hz, 3H), 0.91 (d, *J* = 6.6 Hz, 3H).
^{13}C NMR (126 MHz, $CDCl_3$) δ 124.4 (q, *J* = 282.3 Hz), 59.5, 55.4, 55.2, 49.9 (q, *J* = 33.6 Hz), 44.7, 29.8, 25.0, 23.0, 21.1.
^{19}F NMR(376 MHz, $CDCl_3$) δ –69.27 (t, *J* = 7.9 Hz).
IR (neat) ν 3287 (br), 2962 (w), 1717 (w), 1471 (w), 1429 (w), 1394 (w), 1369 (w), 1328 (m), 1273 (m), 1204 (w), 1143 (s), 1078 (w), 1036 (m), 989 (m), 940 (m), 907 (w), 870 (w), 823 (w), 767 (w), 739 (w), 663 (m), 613 (m), 560 (m), 520 (w), 486 (w) cm^{-1}.
TLC R_f = 0.57 in 4:1 hexanes:EtOAc.
HRMS (ESI) m/z: $[M + H]^+$ Calcd for $C_{12}H_{24}ClF_3N_2O_2S$ 353.1272; Found 353.1273.

***N-tert*-Butoxycarbonyl-*N*-(4-chloropent-1-yl)-*N'*-(2,2,2-trfiluoroethyl)sulfamide (S12v)**

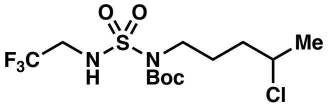

Prepared from sulfamide **S5b** (295 mg, 1.1 mmol) and 4-chloropentanol[6] (100 mg, 0.82 mmol) following general procedure F. The product was obtained as a colorless oil (217 mg, 70% yield) following purification on a Teledyne Isco CombiFlashR$_f$ system using a Redi Sep R$_f$ normal phase silica gel column (4:1 hexanes/EtOAc).

^{1}H NMR (400 MHz, CDCl$_3$) δ 5.78 (br s, 1H), 4.08–4.00 (m, 1H), 3.72–3.66 (m, 4H), 1.92–1.87 (m, 1H), 1.80–1.69 (m, 3H), 1.53 (s, 9H), 1.52 (d, *J* = 6.4 Hz, 3H).
^{13}C NMR (126 MHz, CDCl$_3$) δ 151.6, 123.4 (q, *J* = 279.0 Hz), 84.7, 57.9, 47.5, 45.1 (q, *J* = 35.3 Hz), 36.9, 27.8, 26.7, 25.2.
^{19}F NMR (376 MHz, CDCl$_3$) δ –71.93 (t, *J* = 8.9 Hz).
IR (neat) ν 3299 (br), 2978 (w), 1705 (m), 1368 (m), 1278 (m), 1139 (s), 1010 (w), 910 (w), 832 (m), 796 (w), 773 (w), 718 (s), 664 (m), 611 (m) cm^{-1}.
TLC R$_f$ = 0.45 in 8:2 hexanes:EtOAc.
HRMS (ESI) m/z: [M – H]$^-$ Calcd for $C_{12}H_{21}ClF_3N_2O_4S$ 381.0862; Found 381.0862.

***N-tert*-Butoxycarbonyl-*N*-(4-chloropent-1-yl)-*N'*-ethylsulfamide (S12w)**

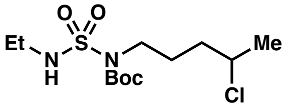

Prepared from sulfamide **S5c** (238 mg, 1.1 mmol) and 4-chloropentanol[6] (100 mg, 0.82 mmol, following general procedure F. The product was obtained as a colorless oil (268 mg, ≥ 98% yield) following purification on a Teledyne Isco CombiFlashR$_f$ system using a Redi Sep R$_f$ normal phase silica gel column (9:1 hexanes/EtOAc).

^{1}H NMR (400 MHz, CDCl$_3$) δ 5.20 (t, *J* = 5.0 Hz, 1H), 4.09–4.01 (m, 1H), 3.68 (t, *J* = 7.0 Hz, 2H), 3.04 (qd, *J* = 7.2, 6.3 Hz, 2H), 1.94–1.87 (m, 1H), 1.79–1.70 (m, 3H), 1.53 (m, 9H), 1.52 (d, *J* = 7.8 Hz, 3H), 1.20 (t, *J* = 7.3 Hz, 3H).
^{13}C NMR (101 MHz, CDCl$_3$) δ 151.7, 83.7, 57.8, 46.8, 38.7, 36.9, 27.8, 26.7, 25.1, 14.2.
IR (neat) ν 3311 (br), 2976 (w), 1703 (m), 1357 (m), 1286 (m), 1256 (m), 1135 (s), 1062 (m), 1009 (w), 844 (m), 794 (m), 773 (m), 714 (s) cm^{-1}.
TLC R$_f$ = 0.36 in 8:2 hexanes:EtOAc.
HRMS (ESI) m/z: [M – H]$^-$ Calcd for $C_{12}H_{24}ClN_2O_4S$ 327.1145; Found 327.1145.

IX. Preparation and characterization of sulfamate ester derivatives.

4-Methylpentyl *tert*-butylsulfamate (S13a)

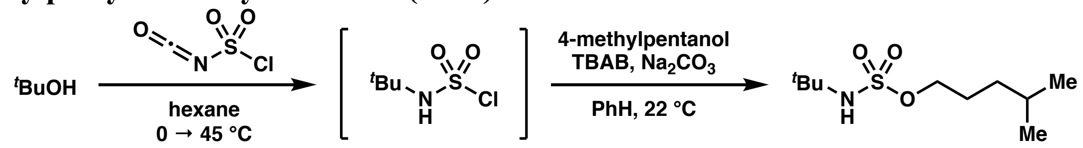

To a flame-dried round bottom flask equipped with magnetic stir bar and fitted with a rubber septum and nitrogen inlet was added hexanes (50 mL, 0.6 M). The reaction flask was then cooled at 0 °C in an ice water bath. *Tert*-butanol (4.3 mL, 30 mmol, 1.5 equiv) and chlorosulfonyl isocyanate (3.9 mL, 45 mmol, 1.5 equiv) were then sequentially added dropwise via syringe. Upon complete addition, the ice water bath was removed and replaced with an oil bath. The oil bath was heated to 45 °C and the reaction was stirred for 3 h. After 3 h, the reaction was removed from heat and allowed to cool to room temperature. The suspension was then concentrated under reduced pressure to remove all volatile materials. The crude material was then taken up in anhydrous benzene (50 mL, 0.60 M) and *tetra*-butylammonium bromide (TBAB, 0.97 g, 3.0 mmol, 0.1 equiv), 4-methylpentanol (3.06 g, 30 mmol, 1.0 equiv), and sodium carbonate (9.54 g, 90 mmol, 3.0 equiv) were added sequentially. Following addition, the reaction was left to stir at 22 °C for 18 h.

After 18 h, the reaction was quenched by dropwise addition of 1 M HCl until the aqueous phase reached a pH = 1.0. The biphasic solution was then transferred to a separatory funnel rinsing the flask with EtOAc (~50 mL) to achieve quantitative transfer. The organic phase was separated and the aqueous phase was extracted twice more with EtOAc (2 x 50 mL). The combined organic layers were washed once with brine (~50 mL), dried with $MgSO_4$, filtered, and concentrated under reduced pressure. The product was obtained as a colorless oil (3.06 g, 43% yield) following silica gel column chromatography (9:1 hexanes:EtOAc).

^{1}H NMR (400 MHz, $CDCl_3$) δ 4.27 (br s, 1H), 4.09 (t, J = 6.7 Hz, 2H), 1.76–1.69 (m, 2H), 1.61–1.54 (m, 1H), 1.36 (s, 9H), 1.31–1.25 (m, 2H), 0.90 (d, J = 6.6 Hz, 6H).
^{13}C NMR (126 MHz, $CDCl_3$) δ 70.7, 54.6, 34.6, 29.7, 27.6, 26.7, 22.4.
IR (neat) ν 3299 (br), 2957 (w), 2872 (w), 1469 (w), 1431 (w), 1394 (w), 1345 (m), 1231 (w), 1161 (s), 1041 (w), 981 (m), 917 (w), 875 (w), 802 (w), 731 (w), 618 (w), 587 (w), 514 (w), 463 (w) cm^{-1}.
TLC R_f = 0.50 in hexanes:EtOAc 4:1.
HRMS (ESI) m/z: $[M + NH_4]^+$ Calcd for $C_{10}H_{23}NO_3S{\bullet}NH_4$ 255.1737; Found 255.1742.

4-Methypentyl (2,2,2-trifluoroethyl)sulfamate (S13b)

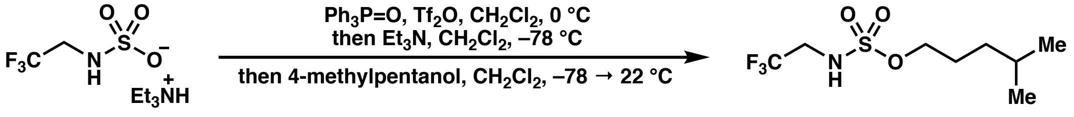

Following a literature procedure,[11] a flame-dried round bottom flask equipped with magnetic stir bar was charged with triphenylphosphine oxide (2.3 g, 8.25 mmol, 1.65 equiv) and fitted with a rubber septum. The flask was evacuated and backfilled with N_2. Anhydrous CH_2Cl_2 (25 mL) was added and the flask was cooled at 0 °C in an ice water bath. Trifluoromethanesulfonic anhydride

(1.25 mL) that had been freshly removed from the glovebox was then added to the cooled solution dropwise via syringe. The reaction was allowed to stir at 0 °C in an ice water bath for 15 minutes. A solution of sulfamic acid salt (2.1 g, 7.5 mmol, 1.5 equiv) in CH_2Cl_2 (4.0 mL) was added to the activated Ph_3PO via cannula transfer. The flask was rinsed with additional CH_2Cl_2 (1 mL) to achieve quantitative transfer. The resulting pale yellow solution was stirred for 15 minutes at 0 °C. During this time, a clean, flame-dried round bottom flask equipped with stir bar was fitted with a rubber septum and subsequently evacuated and backfilled with N_2. This process was repeated two more times. The flask was then charged with Et_3N (2.1 mL, 15.0 mmol, 3.0 equiv) and CH_2Cl_2 (20 mL) and the mixture was cooled at –78 °C in an ^{i}PrOH/dry ice bath. The activated sulfamic acid salt solution was transferred dropwise to the Et_3N solution via cannula (during which time an intense red color developed), rinsing the flask with additional CH_2Cl_2 (5 mL) to achieve quantitative transfer. The resultant solution was stirred at –78 °C for 15 minutes. 4-Methylpentanol (511 mg, 5.0 mmol, 1.0 equiv) was then added as a solution in CH_2Cl_2 (5 mL) to the Et_3N solution via canula. The alcohol-containing flask was rinsed with additional CH_2Cl_2 (1 mL) to achieve quantitative transfer. Without removing the cooling bath, the reaction was then stirred for 18 h, during which time no additional dry ice was added and the mixture warmed to room temperature.

After 18 h, the reaction was diluted with 1 M HCl (~20 mL) and H_2O (~30 mL). The biphasic mixture was transferred to a separatory funnel, rinsing the flask with CH_2Cl_2 to achieve quantitative transfer. The organic phase was separated and the aqueous phase was extracted twice more with CH_2Cl_2 (2 x ~40 mL). The combined organic phases were dried with $MgSO_4$, filtered, and concentrated under reduced pressure. The product was obtained as a white powder (958 mg, 73% yield) following silica gel column chromatography (8:1 hexanes:EtOAc).

^{1}H NMR (400 MHz, $CDCl_3$) δ 5.04 (br s, 1H), 4.16 (t, J = 6.6 Hz, 2H), 3.78–3.70 (m, 2H), 1.77–1.70 (m, 2H), 1.60–1.54 (m, 1H), 1.29–1.24 (m, 2H), 0.89 (d, J = 6.6 Hz, 6H).
^{13}C NMR (126 MHz, $CDCl_3$) δ 123.4 (q, J = 278.3), 72.3, 45.0 (q, J = 36.0), 34.3, 27.6, 26.5, 22.3.
^{19}F NMR (376 MHz, $CDCl_3$) δ –72.73 (t, J = 8.6 Hz).
IR (neat) ν 3296 (br), 2962 (w), 1465 (w), 1409 (w), 1348 (m), 1276 (m), 1154 (s), 1112 (m), 1061 (w), 961 (s), 924 (m), 871 (m), 835 (m), 804 (w), 737 (w), 664 (m), 561 (s), 524 (m), 469 (m) cm^{-1}.
TLC R_f = 0.39 in 4:1 hexanes:EtOAc.
HRMS (ESI) m/z: $[M + NH_4]^+$ Calcd for $C_8H_{16}F_3NO_3S$ 281.1141; Found 281.1147.

General Procedure J. Preparation of *N*-chlorosulfamate esters (9).

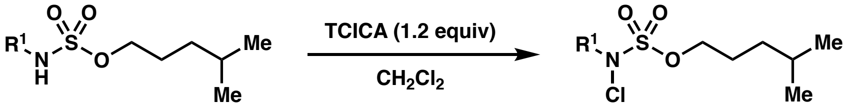

Following a literature procedure,[6b] a round bottom flask equipped with magnetic stir bar was charged with trichloroisocyanuric acid (TCICA, 1.2 equiv) and the flask was evacuated and backfilled with N_2. Anhydrous CH_2Cl_2 (0.2 M) was then added to create a suspension. Sulfamate ester **S13** (1.0 equiv) was then added to the suspension via syringe (if oil) or in a single portion

by brief opening of the flask (if solid). The suspension was stirred at 22 °C and monitored by TLC until complete consumption of starting material was observed.

Upon complete consumption of starting material as judged by TLC, the reaction was diluted with H_2O (~0.2 M) and the biphasic suspension was transferred to a separatory funnel. The reaction flask was rinsed with CH_2Cl_2 (5 mL) to ensure quantitative transfer. The organic phase was separated and the aqueous phase was extracted twice more with CH_2Cl_2. The combined organic phases were dried with $MgSO_4$, filtered, and concentrated under reduced pressure. The crude reaction mixtures were then purified by flash column chromatography on silica gel by loading samples as a solid and eluting with a hexanes:EtOAc solvent system as noted below.

4-Methylpentyl *tert*-butylchlorosulfamate ester (9a).

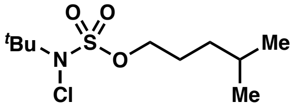

Prepared from sulfamate ester **S13a** (356 mg, 1.5 mmol) and TCICA (418 mg, 1.8 mmol) following general procedure J. The product was obtained as a colorless oil (354 mg, 87% yield) after silica gel flash column chromatography using hexanes:EtOAc (20:1).

^{1}H NMR (400 MHz, $CDCl_3$) δ 4.27 (t, J = 6.7 Hz, 2H), 1.78–1.73 (m, 2H), 1.58–1.55 (m, 1H), 1.49 (s, 9H), 1.31–1.23 (m, 2H), 0.88 (d, J = 6.8 Hz, 6H).
^{13}C NMR (126 MHz, $CDCl_3$) δ 74.5, 67.3, 34.3, 28.4, 27.4, 26.6, 22.3.
IR (neat) ν 2957 (w), 2872 (w), 1468 (w), 1366 (m), 1241 (w), 1191 (m), 1167 (m), 1040 (w), 953 (m), 886 (m), 807 (w), 734 (w), 614 (s), 591 (w), 517 (w), 469 (w), 433 (w) cm^{-1}.
TLC R_f = 0.57 in 10:1 hexanes:EtOAc.
HRMS (ESI) m/z: $[M + NH_4]^+$ Calcd for $C_{10}H_{22}ClNO_3S{\bullet}NH_4$ 289.1347; Found 289.1345.

4-Methylpentyl (2,2,2-trifluoroethyl)chlorosulfamate ester (9b).

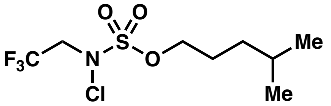

Prepared from sulfamate ester **S13b** (479 mg, 1.8 mmol) and TCICA (508 mg, 2.2 mmol) following general procedure J. The product was obtained as a colorless oil (489 mg, 90% yield) after silica gel flash column chromatography using hexanes:EtOAc (20:1).

^{1}H NMR (400 MHz, $CDCl_3$) δ 4.39 (t, J = 6.5 Hz, 2H), 4.08 (q, J = 8.1 Hz, 2H), 1.84–1.77 (m, 2H), 1.63–158 (m, 1H), 1.34–1.28 (m, 2H), 0.91 (d, J = 6.6 Hz, 6H).
^{13}C NMR (126 MHz, $CDCl_3$) δ 122.9 (q, J = 279.8 Hz), 76.7, 57.2 (q, J = 35.6 Hz), 34.2, 27.5, 26.7, 22.3.
^{19}F NMR (376 MHz, $CDCl_3$) δ –70.51 (t, J = 7.8 Hz).
IR (neat) ν 2960 (w), 2874 (w), 1470 (w), 1382 (m), 1310 (m), 1272 (m), 1159 (s), 1072 (w), 963 (s), 930 (s), 844 (m), 736 (w), 689 (m), 648 (m), 574 (s), 520 (w), 471 (m) cm^{-1}.
TLC R_f = 0.65 in 8:1 hexanes:EtOAc.
HRMS (ESI) m/z: $[M + NH_4]^+$ Calcd for $C_8H_{15}ClF_3NO_3S{\bullet}NH_4$ 315.0752; Found 315.0746.

General Procedure K. Sulfamate ester-guided chlorine-transfer reactions.

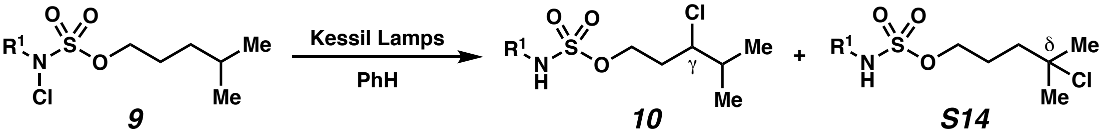

9 ***10*** ***S14***

The reactions were performed as described for previously reported sulfamate ester guided chlorination processes.[6b] In a flame-dried microwave vial equipped with magnetic stir bar and sealed with a crimp cap, *N*-chlorosulfamate ester **7** (0.2 mmol, 1.0 equiv) was dissolved in anhydrous PhH (3.0 mL, 0.07 M) under an inert atmosphere. The vial was then placed in between two Kessil lamps (ca. 5 cm away from each light source) and irradiated for 15 minutes. After 15 minutes, the vial was removed from the light, and the reaction was analyzed by TLC. If starting material remained, the reaction was irradiated between two Kessil lamps in 15 minute increments until all starting material had been consumed as judged by TLC.

Upon consumption of starting material as judged by TLC, the reaction solution was transferred to a flask rinsing the microwave vial with CH_2Cl_2 (ca. 3 mL) to make transfer quantitative. Volatiles were removed under reduced pressure and the crude material was purified by flash column chromatography on silica gel or Florisil by loading the samples as liquids in column eluent and eluting with a hexanes:EtOAc solvent system as noted below.

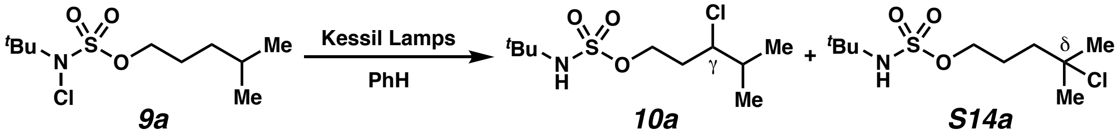

9a ***10a*** ***S14a***

Chlorosulfamate **9a** (54 mg, 0.2 mmol) was subjected to the general procedure. After irradiating for 1 hour, products **10a** and **S14a** (31 mg, 57% yield) were isolated as a mixture following purification by silica gel column chromatography (Gradient: 100% hexanes → 10:1 hexanes/acetone).

In order to determine the ratio of **10a**:**S14a**, duplicate reactions of chlorosulfamide **9a** (8 mg, 0.03 mmol) were subjected to general procedure K. After irradiating for 1 hour, each sample was concentrated under reduced pressure and 1,2,4,5-tetrachloro-3-nitrobenzene (1.0 equiv) was added as an internal standard. ^1H NMR analysis of the crude reaction mixtures revealed a 3.2:1.0 mixture of **10a**:**S14a** and 10% protodehalogenated material as an average of two runs.
Sample 1: 57% yield **10a**, 18% yield **S14a**, 7% yield protodehalogenated **S13a**.
Sample 2: 56% yield **10a**, 18% yield **S14a**, 12% yield protodehalogenated **S13a**.

3-Chloro-4-methylpentyl *tert*-butylsulfamate (10a).

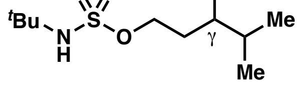

Isolated for characterization by silica gel column chromatography (Gradient: 100% hexanes → 25:1 hexanes:EtOAc) from a mixture of chlorinated isomers (**10a** and **S14a**).

^{1}H NMR (400 MHz, $CDCl_3$) δ 4.41 (br s, 1H), 4.32–4.29 (m, 2H), 3.99 (ddd, *J* = 6.7, 2.6, 2.6 Hz, 1H), 2.21–2.13 (m, 1H), 2.03–1.94 (m, 2H), 1.37 (s, 9H), 1.04 (d, *J* = 6.7 Hz, 3H), 0.99 (d, *J* = 6.7 Hz).
^{13}C NMR (126 MHz, $CDCl_3$) δ 67.5, 65.4, 54.7, 35.0, 34.4, 29.5, 19.7, 17.1.
IR (neat) ν 3298 (w), 2969 (w), 1467 (w), 1430 (w), 1394 (m), 1369 (m), 1345 (m), 1232 (w), 1206 (w), 1159 (s), 1084 (w), 1043 (w), 986 (s), 914 (m), 884 (m), 832 (w), 781 (m), 616 (m), 591 (w), 513 (m), 471 (w) cm^{-1}.
TLC R_f = 0.47 in 4:1 hexanes:EtOAc.
HRMS (ESI) m/z: $[M – H]^-$ Calcd for $C_{10}H_{22}ClNO_3S$ 270.0936; Found 270.0932.

4-Chloro-4-methylpentyl *tert*-butylsulfamate (S14a).

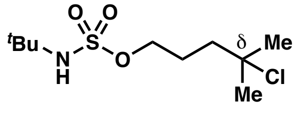

Isolated for characterization by silica gel column chromatography (Gradient: 100% hexanes → 25:1 hexanes:EtOAc) from a mixture of chlorinated isomers (**10a** and **S14a**).

^{1}H NMR (400 MHz, $CDCl_3$) δ 4.32 (br s, 1H), 4.15 (t, *J* = 6.1 Hz, 2H), 1.99–1.94 (m, 2H), 1.87–1.85 (m, 2H), 1.59 (s, 6H), 1.36 (s, 9h).
^{13}C NMR (126 MHz, $CDCl_3$) δ 70.2, 70.0, 54.7, 42.0, 32.4, 29.7, 24.9.
IR (neat) ν 3297 (br), 2974 (w), 1471 (w), 1430 (w), 1394 (m), 1370 (m), 1343 (m), 1233 (w), 1210 (w), 1159 (s), 981 (m), 924 (m), 874 (m), 804 (m), 733 (m), 615 (m), 587 (m), 512 (m), 425 (w) cm^{-1}.
TLC R_f = 0.35 in 4:1 hexanes:EtOAc.
HRMS (ESI) m/z: $[M – H]^-$ Calcd for $C_{10}H_{22}ClNO_3S$ 270.0936; Found 270.0931.

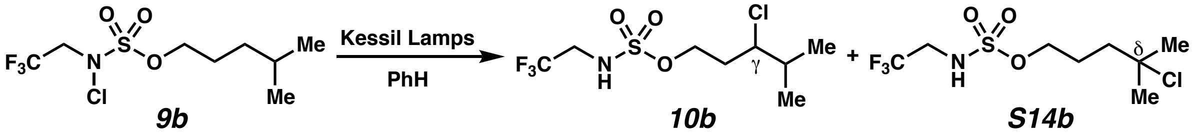

Chlorosulfamate **9b** (60 mg, 0.2 mmol) was subjected to the general procedure. After irradiating for 1 hour, products **10b** and **S14b** (40 mg, 67% yield) were isolated as a mixture following purification by silica gel column chromatography (Gradient: 100% hexanes → 10:1 hexanes/acetone).

In order to determine the ratio of **10b**:**S14b**, duplicate reactions of chlorosulfamide **9b** (8 mg, 0.03 mmol) were subjected to general procedure K. After irradiating for 1 hour, each sample was concentrated under reduced pressure and 1,2,4,5-tetrachloro-3-nitrobenzene (1.0 equiv) was added as an internal standard. ^{1}H NMR analysis of the crude reaction mixtures revealed a 4:1 mixture of **10b**:**S14b** and 5% protodehalogenated material as an average of two runs.
Sample 1: 56% yield **10b**, 14% yield **S14b**, 5% yield protodehalogenated **S13b**.
Sample 2: 53% yield **10b**,13% yield **S14b**, 4% yield protodehalogenated **S13b**.

3-Chloro-4-methylpentyl (2,2,2-trifluoroethyl)sulfamate (10b).

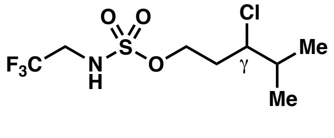

Isolated for characterization by silica gel column chromatography (Gradient: 100% hexanes → 25:1 hexanes:EtOAc) from a mixture of chlorinated isomers (**10b** and **S14b**).

^{1}H NMR (400 MHz, $CDCl_3$) δ 5.13 (t, *J* = 6.5 Hz, 1H), 4.40–4.37 (m, 2H), 3.97 (ddd, *J* = 6.7, 4.0, 4.0 Hz, 1H), 3.80–3.72 (m, 2H), 2.22–2.14 (m, 1H), 2.04–1.94 (m, 2H), 1.04 (d, *J* = 6.7 Hz, 3H), 0.99 (d, *J* = 6.7 Hz, 3H).
^{13}C NMR (126 MHz, $CDCl_3$) δ 123.3 (q, *J* = 278.3 Hz), 69.1, 65.0, 45.2 (q, *J* = 35.8 Hz), 34.7, 34.4, 19.6, 17.2.
^{19}F NMR (376 MHz, $CDCl_3$) δ–72.67 (t, *J* = 8.6 Hz).
IR (neat) ν 3307 (br), 2970 (w), 1465 (w), 1359 (m), 1275 (m), 1152 (s), 1044 (w), 963 (m), 918 (m), 868 (m), 816 (w), 777 (m), 664 (m), 561 (s), 512 (m), 471 (w), 439 (w) cm^{-1}.
TLC R_f = 0.41 in 4:1 hexanes:EtOAc.
HRMS (ESI) m/z: $[M – H]^-$ Calcd for $C_8H_{15}ClF_3NO_3S$ 296.0341; Found 296.0334.

4-Chloro-4-methylpentyl (2,2,2-trifluoroethyl)sulfamate (S14b).

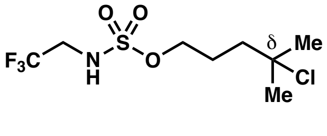

Isolated for characterization by silica gel column chromatography (Gradient: 100% hexanes → 25:1 hexanes:EtOAc) from a mixture of chlorinated isomers (**10b** and **S14b**).

^{1}H NMR (400 MHz, $CDCl_3$) δ 4.90 (t, *J* = 6.3 Hz, 1H), 4.24 (t, *J* = 6.0 Hz, 2H), 3.20–3.72 (m, 2H), 2.02–1.95 (m, 2H), 1.86–1.82 (m, 2H), 1.59 (s, 6H).
^{13}C NMR (126 MHz, $CDCl_3$) δ 123.3 (q, *J* = 277.1 Hz), 71.7, 69.8, 45.2 (q, *J* = 35.7 Hz), 41.7, 32.4, 24.7.
^{19}F NMR (376 MHz, $CDCl_3$) δ –72.67 (t, *J* = 8.8 Hz).
IR (neat) ν 3304 (br), 2923 (w), 2850 (w), 1457 (w), 1361 (m), 1276 (m), 1156 (s), 966 (m), 929 (m), 861 (w) 739 (w), 665 (w), 563 (m), 512 (w) cm^{-1}.
TLC R_f = 0.26 in 4:1 hexanes:EtOAc.
HRMS (ESI) m/z: $[M – H]^-$ Calcd for $C_8H_{15}ClF_3NO_3S$ 296.0341; Found 296.0333.

X. Sulfamide cleavage to access 3-chloroalkylamines.

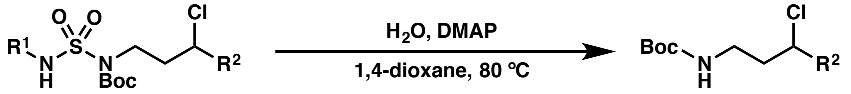

A clean microwave vial equipped with a stir bar was charged with sulfamide (1 equiv) and DMAP (1.5 equiv) and sealed with an aluminum seal cap. The vial was evacuated and backfilled with N_2 and the process was repeated two more times. 1,4-dioxane (0.13 M) and H_2O (2 equiv) were then added via syringe and the resulting solution was heated in an oil bath at 80 ºC overnight.

After stirring overnight, the suspension was allowed to cool to 21 ºC and then concentrated under reduced pressure. The crude mixture was then purified by silica gel flash chromatography as indicated below.

Methyl 6-((*tert*-butoxycarbonyl)amino)-4-chlorohexanoate (S15k)

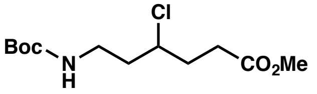

Prepared from sulfamide **4k** (150 mg, 0.36 mmol) according to the general procedure. The product was obtained as a colorless oil (85 mg, 85% yield) following purification on a Teledyne Isco CombiFlashR$_f$ system using a Redi Sep R$_f$ normal phase silica gel column (8:2 hexanes/EtOAc).

^{1}H NMR (400 MHz, $CDCl_3$) δ 4.67 (br s, 1H), 4.02–3.95 (m, 1H), 3.69 (s, 3H), 3.40–3.33 (m, 1H), 3.30–3.23 (m, 1H), 2.61–2.47 (m, 2H), 2.19–2.10 (m, 1H), 2.07–1.91 (m, 2H), 1.87–1.78 (m, 1H), 1.44 (s, 9H).
^{13}C NMR (101 MHz, $CDCl_3$) δ173.2, 155.9, 79.3, 60.1, 51.7, 38.4, 37.9, 33.4, 30.8, 28.3.
IR (neat) ν 3364 (br), 2975 (w), 1689 (m), 1514 (m), 1437 (m), 1391 (w), 1365 (w), 1247 (m), 1162 (s), 994 (w), 838 (w), 780 (w), 606 (w) cm^{-1}.
TLC R_f = 0.54 in 6:4 hexanes/EtOAc.
HRMS (ESI) m/z: $[M + Na]^+$ Calcd for $C_{12}H_{22}ClNO_4$•Na 302.1135; Found 302.1134.

XI. Preparation and characterization of compounds related to the radical clock experiment

N-tert-butyl-_N'_-(3-(2,2-dimethylcyclopropyl)propan-1-yl)-_N'_-(2,2,2-trifluoro)ethylsulfamide (S8s)

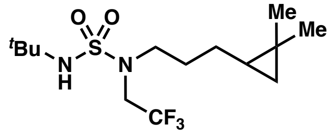

Prepared from *N*-(*tert*-butyl)-*N'*-(2,2,2-trifluoroethyl)sulfamide (513 mg, 2.2 mmol) and 3-(2,2-dimethylcylcopropyl)propanon-1-ol[12] (216 g, 1.7 mmol) following general procedure F. The product was obtained as a colorless oil (319 mg, 55% yield) after purification by silica gel column chromatography (Gradient: 100% hexanes → 20:1 hexanes:EtOAc).

^{1}H NMR (400 MHz, $CDCl_3$) δ 4.19 (br s, 1H), 3.80 (q, *J* = 8.9 Hz, 2H), 3.36–3.24 (m, 2H), 1.77–1.69 (m, 2H), 1.33 (s, 9H), 1.28–1.21 (m, 2H), 1.01 (s, 6H), 0.48–0.37 (m, 2H), –0.11 – –0.13 (m, 1H).
^{13}C NMR (126 MHz, $CDCl_3$) δ 124.5 (q, *J* = 280.4 Hz), 54.8, 49.1, 48.0 (q, *J* = 33.9 Hz), 29.7, 27.5, 27.4, 26.8, 24.0, 19.9, 19.7, 15.4.
^{19}F NMR(376 MHz, $CDCl_3$) δ –69.49 (t, *J* = 8.9 Hz)
IR (neat) ν 3286 (br), 2941(w), 1429 (w), 1393 (w), 1369 (w), 1323 (m), 1274 (m), 1229 (w), 1141 (s), 1085 (w), 995 (m), 908 (w), 871 (w), 831 (w), 770 (w), 734 (m), 665 (m), 609 (m), 564 (m), 519 (w) cm^{-1}.
TLC R_f = 0.26 in 9:1 hexanes:EtOAc.
HRMS (ESI) m/z: $[M + H]^+$ Calcd for $C_{14}H_{27}F_3N_2O_2S$ 345.1818; Found 345.1815.

N-tert-butyl-_N_-chloro-_N'_-(3-(2,2-dimethylcyclopropyl)propan-1-yl)-_N'_-(2,2,2-trifluoro)ethylsulfamide (1s)

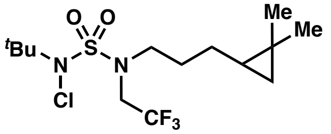

Prepared from sulfamide **S8s** (245 mg, 0.71 mmol) and TCICA (198 mg, 0.85 mmol) in CH_2Cl_2 (4 mL) following general procedure G. The product was obtained as a colorless oil (220 mg, 82% yield) after silica gel column chromatography (Gradient: 100% hexanes → 20:1 hexanes/EtOAc).

^{1}H NMR (400 MHz, $CDCl_3$) δ 3.94 (q, *J* = 8.8 Hz, 2H), 3.45–3.40 (m, 2H), 1.82–1.75 (m, 2H), 1.50 (s, 9H), 1.38–1.25 (m, 2H), 1.03 (s, 6H), 0.50–0.38 (m, 2H), –0.09 – –0.11 (m, 1H).
^{13}C NMR (126 MHz, $CDCl_3$) δ 123.8 (q, *J* = 280.8 Hz), 67.7, 50.5 50.4 (q, *J* = 35.2 Hz), 28.9, 27.5, 27.4, 26.7, 23.9, 19.9, 19.7, 15.4.
^{19}F NMR(376 MHz, $CDCl_3$) δ –70.13 (t, *J* = 8.8 Hz).
IR (neat) ν 2941 (w), 1463 (w), 1400 (w), 1360 (m), 1313 (w), 1276 (m), 1233 (w), 1153 (s), 1109 (w), 1085 (w), 1026 (m), 1006 (m), 873 (m), 820 (m), 787 (w), 736 (w), 663 (m), 606 (m), 566 (m), 519 (w), 487 (m) cm^{-1}.
TLC R_f = 0.53 in 9:1 hexanes:EtOAc.
HRMS (ESI) m/z: $[M + NH_4]^+$ Calcd for $C_{14}H_{26}ClF_3N_2O_2S{\bullet}NH_4$ 396.1699; Found 397.1701.

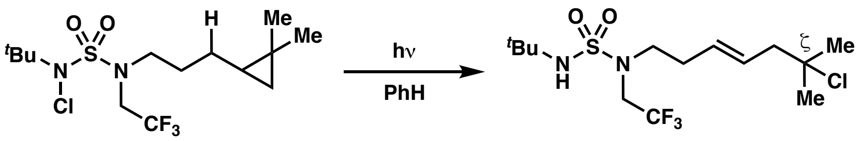

1s **12s**

In order to determine the yield of the ring-opened isomers, duplicate reactions of chlorosulfamide **1s** (10 mg, 0.027 mmol) were subjected to general procedure I. After irradiating for 15 minutes, each sample was concentrated under reduced pressure and 2-bromo-5-(trifluoromethyl)pyridine (0.03 mmol, 1.11 equiv) was added as an internal standard. ^{19}F NMR analysis of the crude reaction mixtures revealed an 80% yield of ζ-chlorinated ring-opened isomer **12s** as an average of 2 runs.
Sample 1: 78% yield **12s**
Sample 2: 82% **12s**

***N-tert*-butyl-*N'*-(6-chloro-6-methylhept-3-en-1-yl)-*N'*-(2,2,2-trifluoro)ethylsulfamide (12s)**

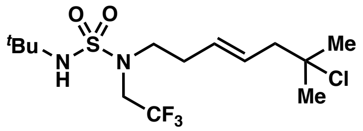

Chlorosulfamide **1s** (76 mg, 0.2 mmol) was subjected to general procedure I. After irradiating for 15 minutes, product **12s** was isolated for characterization by silica gel column chromatography (Gradient: 100% hexanes → 4:1 hexanes:Et_2O).

^{1}H NMR (400 MHz, $CDCl_3$) δ 5.66 (ddd, J = 14.4, 7.5, 7.5 Hz, 1H), 5.47 (dt, J = 14.4, 7.2, 7.2 Hz, 1H), 4.02 (br s, 1H), 3.83 (q, J = 8.8 Hz, 2H), 3.25 (dd, J = 7.4 Hz, 2H), 2.45–2.40 (m, 4H), 1.54 (s, 6H), 1.34 (s, 9H).
^{13}C NMR (126 MHz, $CDCl_3$) δ 130.0, 128.5, 124.4 (q, J = 281.5 Hz), 69.8, 54.9, 48.9, 48.9, 48.3 (q, J = 34.3 Hz), 32.1, 30.7, 29.7.
^{19}F NMR(376 MHz, $CDCl_3$) δ –69.48 (t, J = 9.0 Hz).
IR (neat) ν 3290 (br), 2974 (w), 1426 (w), 1393 (w), 1370 (w), 1324 (m), 1273 (m), 1232 (w), 1141 (s), 1085 (m), 1040 (m), 975 (m), 907 (w), 871 (w), 833 (w), 767 (w), 738 (w), 665 (m), 609 (w), 568 (m), 520 (w), 406 (w) cm^{-1}.
TLC R_f = 0.41 in 4:1 hexanes:EtOAc.
HRMS (ESI) m/z: $[M + H]^+$ Calcd for $C_{14}H_{26}ClF_3N_2O_2S$ 379.1430; Found 379.14284.

XII. Description of quantum yield experiments.

Determination of Light Intensity.

The light intensity was measured to determine the photon flux of the fluorimeter.[20] All solutions were stored in the dark and used the same day as prepared. Manipulations were performed in the dark and care was taken such that samples were protected from ambient light.

Preparation of stock solutions.
A 0.15 M solution of ferrioxalate was prepared by dissolving potassium ferrioxalate trihydrate ($K_3[Fe(C_2O_4)_3]\bullet 3H_2O$, 1.84 g, 3.75 mmol) in 0.05 M H_2SO_4 and diluting to 25 mL total volume with 0.05 M H_2SO_4 in a 25 mL volumetric flask.

A buffered 1,10-phenanthroline solution was prepared by dissolving phenanthroline (50 mg, 0.277 mmol) and sodium acetate (11.25 g) in 0.5 M H_2SO_4 and diluting to 50 mL total volume with 0.5 M H_2SO_4 in a 50 mL volumetric flask.

Determination of background Fe^{2+} concentration.
2.00 mL of the ferrioxalate solution was added to a quartz cuvette. Next 0.35 mL of the phenanthroline solution was added and the mixture was stored in the dark for 1 h. The UV-vis spectrum was subsequently obtained and the absorbance value recorded at 510 nm. The process was repeated 2 additional times for 3 total trials.

Trial 1: 0.2440203428
Trial 2: 0.2468722761
Trial 3: 0.1206529737
Average: 0.2038485309

Determination of photon flux.
2.00 mL of the ferrioxalate solution was added to a quartz cuvette. The cuvette was immediately irradiated with ultraviolet light (313 nm ± 5 nm) in a fluorimeter for 90 seconds. The cuvette was then removed from the fluorimeter and 0.35 mL of the phenanthroline solution was added to the ferrioxalate solution. The resulting solution was stored in the dark for 1 h and the UV-vis spectrum was obtained. The absorbance value at 510 nm was recorded and the process was repeated 2 additional times for 3 total trials.

Trial 1: 1.293553352
Trial 2: 1.417163253
Trial 3: 1.422570229
Average: 1.377762278

Calculations.
The amount of Fe^{2+} formed after irradiation was calculated according to the Beer-Lambert law:

$$mol\ Fe(II) = \frac{V \cdot \Delta A}{l \cdot \varepsilon}$$

where V is the volume of the sample analyzed (2.35 mL), ΔA is the difference in average absorbances (between the irradiated and unirradiated ferrioxalate solutions) at 510 nm, l is the path length of the cuvette, and ε is the molar absorptivity at 510 nm.

$$mol\ Fe(II) = \frac{(0.00235\ L) \cdot (1.173913747)}{(1.00\ cm) \cdot \left(11,100 \frac{L}{mol \cdot cm}\right)} = 2.48531 \cdot 10^{-7}\ mol$$

The fraction of light absorbed by the ferrioxalate actinometer was calculated by the following equation:

$$f = 1 - 10^{-A}$$

where A is the absorbance at 313 nm of the ferrioxalate actinometer solution prior to irradiation and addition of phenanthroline.

$$f = 1 - 10^{-10} = 0.999 \approx 1.000$$

The photon flux was calculated using the following equation:

$$photon\ flux = \frac{mol\ Fe(II)}{\phi \cdot t \cdot f}$$

where ϕ is the quantum yield of the ferrioxalate actinometer at 313 nm,[21] t is the time and f is the fraction of light absorbed by the ferrioxalate actinometer solution.

$$photon\ flux = \frac{2.48531 \cdot 10^{-7}\ mol}{(1.24\ einstein^{-1}) \cdot (90\ s) \cdot (1.000)} = 2.22698 \cdot 10^{-9}\ einstein \cdot s^{-1}$$

Determination of quantum yield.

In a nitrogen-filled glovebox, a quartz cuvette was charged with sulfamide **1h** (42.7 mg, 0.10 mmol, 1 equiv) and PhH (2.5 mL, 0.04 M). The cuvette was fitted with a Teflon cap and sealed with parafilm. The vial was then placed in a fluorimeter and irradiated with ultraviolet light (313 nm ± 5 nm) for 60 minutes. After 60 minutes, the vial was removed from the fluorimeter and the reaction mixture was transferred to a scintillation vial and concentrated under reduced pressure. The residue was purified as detailed above to provide the desired product (25.7 mg, 60% yield, 0.064 mmol). A duplicate experiment afforded 24.0 mg of the desired product (56% yield). The quantum yield was calculated as an average of the two runs.

The minimum quantum yield (ϕ) was calculated using the following equation:

$$\phi = \frac{mol\ product}{flux \cdot t \cdot f}$$

Where t is the reaction time and f is the fraction of light absorbed by sulfamide **1h**.

$$\phi = \frac{0.000058\,mol}{(2.22698 \cdot 10^{-9} einstein \cdot s^{-1}) \cdot (3600\ s) \cdot (1.000)} = 7.25$$

XIII. UV-Vis Spectra.

N-*tert*-Butoxycarbonyl-*N'*-*tert*-butyl-*N'*-chloro-*N*-(3,7-dimethyloct-1-yl)sulfamide **1h** in PhH (0.04 M).

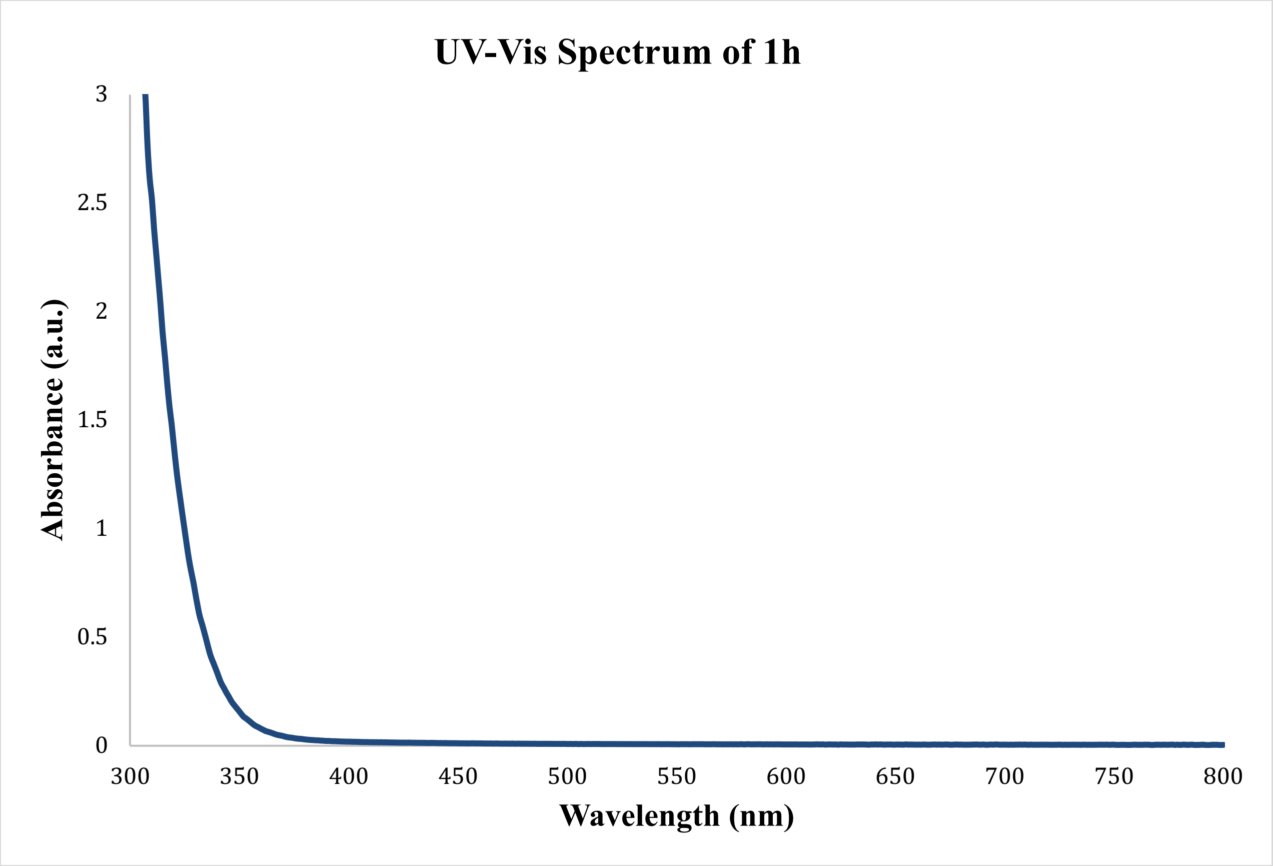

Absorbance at 313 nm: 2.142662287
Fraction of light absorbed at 313 nm: 0.99279991

N-*tert*-Butoxycarbonyl-*N'*-*tert*-butyl-*N*-(3-chloro-3,7-dimethyloct-1-yl)sulfamide **4h** in PhH (0.04 M).

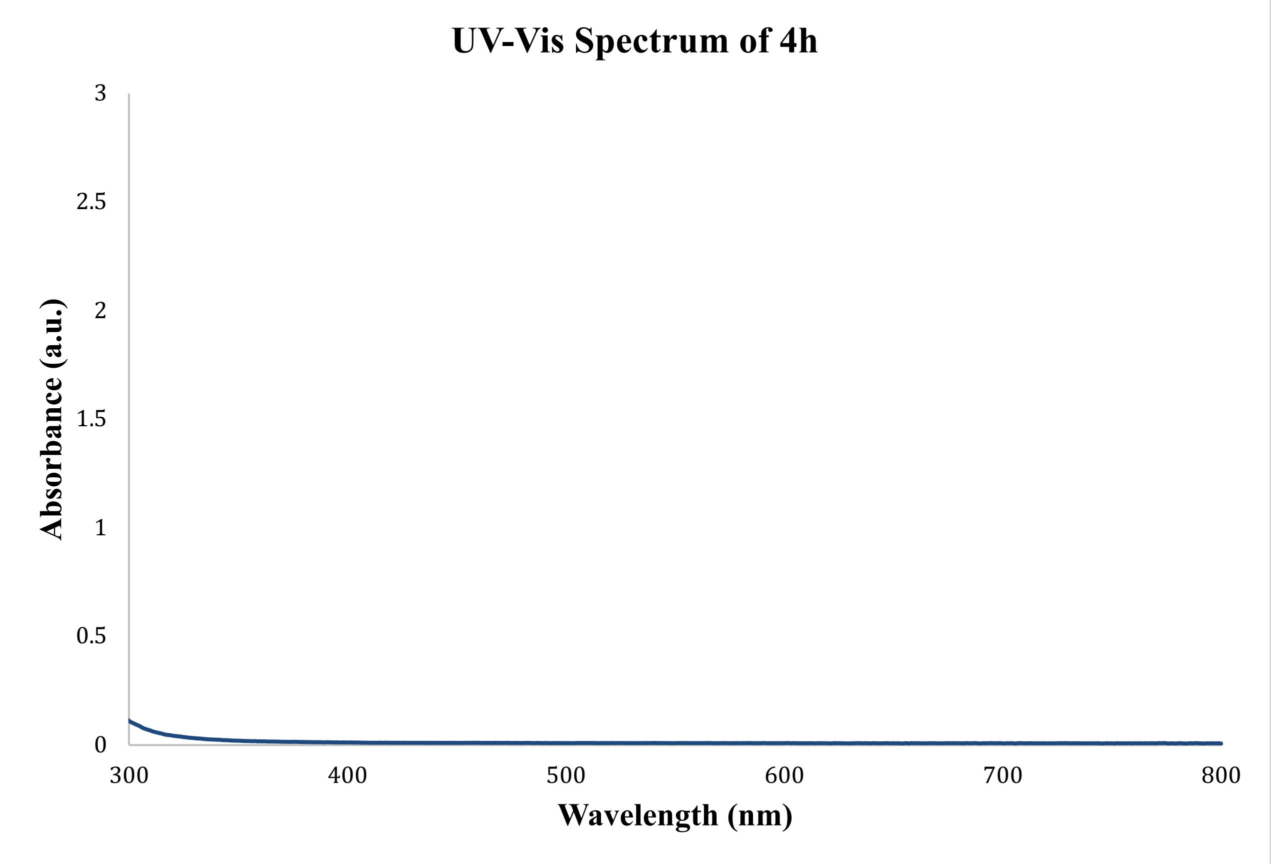

Absorbance at 313 nm: 0.05732247606
Fraction of light absorbed at 313 nm: 0.12365014

XIV. S–N bond lengths in related sulfamides

Structure	S–N(1) [Å]	S–N(2) [Å]	N(2)–S–N(1) °	Reference
$((Cl(CH_2)_2)N)SO_2(N((CH_2)_2Cl)Boc)_2$	1.6875 (10)	1.6147 (11)	108.16 (5)	A.Seridi, H.Akkari, J.-Y.Winum, P.Benard-Rocherulle, M.Abdaoui, *Acta Crystallographica Section E: Structure Reports Online*, 2009, 65, o2543, DOI: 10.1107/S1600536809038185
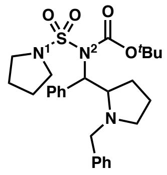	1.602(2)	1.689(2)	105.51(9)	Jorge Vargas-Caporali, Arie van der Lee, Georges Dewynter, Eusebio Juaristi, *Letters in Organic Chemistry*, 2018, 15, 352, DOI: 10.2174/1570178615666180110162202
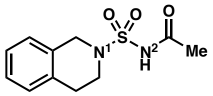	1.589 (3)	1.661 (3)	108.9 (1)	Chafika Bougheloum, Carole Barbey, Malika Berredjem, Abdelrani Messalhi, Nathalie Dupont, *Journal of Molecular Structure*, 2013, 1041, 6, DOI: 10.1016/j.molstruc.2013.02.018
$(^tBuNH)_2SO_2$	1.615	1.616	110.8 (1)	J.L.Atwood, A.H.Cowley, W.E.Hunter, S.K.Mehrotra, *Inorganic Chemistry*, 1982, 21, 435, DOI: 10.1021/ic00131a081
$((Me_2CHCH_2)NH)_2SO_2$	1.615 (5)	1.615 (5)	111.5 (3)	Bing Gong, Zhong Zheng, E.Skrzypczak-Jankun, Yinfa Yan, Jianhua Zhang, *Journal of the American Chemical Society*, 1998, 120, 1119, DOI: 10.1021/ja981707c

$((PhCH_2)NH)_2SO_2$	1.602 (2)	1.616 (2)	112.06 (13)	DOI: 10.1021/ja981707c
$((H_2NCOCH_2CH_2)NH)_2SO_2$	1.612 (2)	1.612 (2)	111.79 (12)	DOI: 10.1021/ja981707c
$((CH_3OC_6H_4CH_2)NH)_2SO_2$	1.615 (5)	1.618 (8)	112.9 (3)	DOI: 10.1021/ja981707c
$((PhCH(CH_3))NH)_2SO_2$	1.608 (2)	1.609 (2)	112.16 (12)	DOI: 10.1021/ja981707c
$(CyNH)_2SO_2$	1.6062(13)	1.6062(13)	102.33(11)	M.B.Hursthouse, T.Gelbrich, N.Bricklebank CCDC 223234: Experimental Crystal Structure Determination, 2014, DOI: 10.5517/cc7h933
$((H_2NCO(CH_2)_3)NH)_2SO_2$	1.618 (2)	1.618 (2)	111.8 (2)	Bing Gong, Chong Zheng, Jianhua Zhang, *Journal of Chemical Crystallography*, 1999, 29, 645, DOI: 10.1023/A:1009569417467
$((^iPrCH_2CH_2)NH)_2SO_2$	1.617 (5)	1.586 (6)	110.7 (3)	Bing Gong, Chong Zheng, E.Skrzypczak-Jankun, Jin Zhu, *Organic Letters*, 2000, 2, 3273, DOI: 10.1021/ol006343j
$((CyCH_2)NH)_2SO_2$	1.591 (12)	1.624 (12)	110.5 (10)	DOI: 10.1021/ol006343j
$((CH_3(CH_2)_4)NH)_2SO_2$	1.614 (2)	1.614 (2)	111.47 (18)	DOI: 10.1021/ol006343j
$((CH_3(CH_2)_5)NH)_2SO_2$	1.623 (3)	1.623 (3)	110.5 (2)	DOI: 10.1021/ol006343j
$((CH_3(CH_2)_7)NH)_2SO_2$	1.6235 (12)	1.6235 (12)	111.97 (9)	DOI: 10.1021/ol006343j

XV. Computational Calculations

Computational Methods

Density functional theory (DFT) calculations were performed using Gaussian 09[22] (revision D.01)[23] or Gaussian 16 (revision B.01).[24] GaussView[25] was used to generate input geometries and visualize output. Geometry minimizations and frequency calculations for 1,6- and 1,7-HAT pathways were initially performed using the uB3LYP[26] functional and the 6-31+G(d,p) basis set[27] with inspiration from related calculations by Leonori and co-workers.[28] The calculations were performed using the polarizable continuum model (PCM) to account for solvation effects in benzene and an ultrafine (99,590) integration grid to limit errors associated with low-frequency vibrational modes.[29]. These calculations were performed without any corrections for quantum tunneling.

Table S2. Investigations comparing experimental product ratios to computationally-calculated differences in transition state energies for 1,6- vs 1,7-HAT pathways.

entry	parent compound	experimental $\Delta\Delta G^{\ddagger}$ (kcal•mol^{-1})[a]	calculated $\Delta\Delta G^{\ddagger}$ (kcal•mol^{-1})[b] uB3LYP / 6-32+G(d,p)		
			RRHO Approximation	Quasi-RRHO Approximation[c]	Quasi-Harmonic Approximation[d]
1	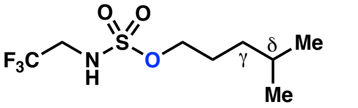	–0.82	–3.33	–2.94	–2.76
2	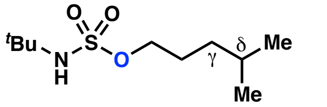	–0.69	–1.09	–0.63	–0.55
3	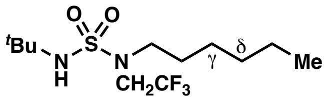	≤ –1.77	–3.74	–3.21	–2.90
4	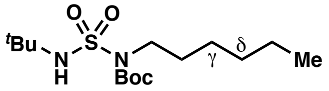	–1.56	–2.98	–2.66	–2.48
5	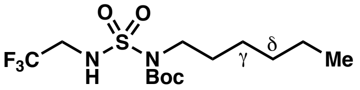	–1.39	–2.06	–1.77	–1.65
6	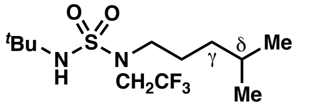	–1.52	+0.35	+0.84	+0.87
7	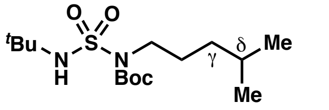	–0.41	+2.24	+2.59	+2.58
8	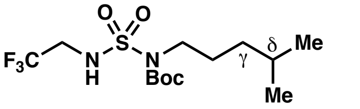	+0.38	+1.04	+1.42	+1.49

[a] $\Delta\Delta G^{\ddagger} = -RT$ ln(γ-chlorinated product / δ-chlorinated product) as determined by ^{1}H NMR of crude reaction mixture. [b] $\Delta\Delta G^{\ddagger}$ = (ΔG(1,6-HAT TS)–ΔG(1,7-HAT TS)) as determined from the

calculated Gibbs free energies using the functional/basis set combination reported at the top of each column. [c] Corrected using the quasi-RRHO approximation of Grimme.[30] [d] Corrected using the quasi-harmonic approximation of Cramer and Truhlar.[31]

The computed energies of the transition states were found to be highly dependent upon the molecule's orientation,[29] which we expect leads, at least in part, to the discrepancies in both qualitative and quantitative trends between the experimental and calculated values. These discrepancies appear to be most drastic for sulfamide substrates where there is a significant difference in BDE of the hydrogen atoms being engaged by the associated 1,6- and 1,7-HAT processes (entries 6–8). The observed trends hold even when quasi-harmonic corrections have been applied.[30,31] These corrections should limit error associated with contributions with the sensitive entropic component of small vibrational frequencies.

The calculations were evaluated for spin contamination by comparing the value of total spin (S**2) at the optimized energy to s(s+1) for the system (s = ½ times the number of unpaired electrons). The aforementioned calculations did not appear to suffer from any significant spin contamination using this approach.

Table S3. Computed Energies
DFT Method: uB3LYP / 6-31+G(d,p) / PCM – benzene [values in Hartrees]

entry	species	total electronic energy	sum of electronic and zero-point energies	sum of electronic and thermal enthalpies	Gibbs free energy
1	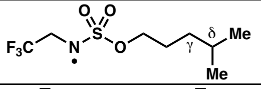	-1291.971313	-1291.731514	-1291.712045	-1291.782881
2	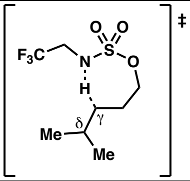	-1291.949419	-1291.713544	-1291.695571	-1291.760012
3	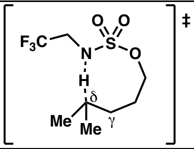	-1291.944541	-1291.708985	-1291.690935	-1291.754709
4	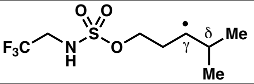	-1291.976256	-1291.737506	-1291.717239	-1291.791126
5	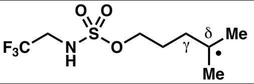	-1291.976256	-1291.737506	-1291.717239	-1291.791126
6	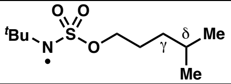	-1072.872454	-1072.553343	-1072.533194	-1072.603021
7	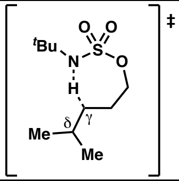	-1072.843678	-1072.528804	-1072.510182	-1072.573582
8	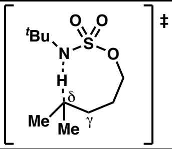	-1072.842968	-1072.528043	-1072.509493	-1072.571838
9	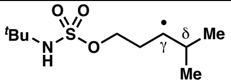	-1072.870243	-1072.552259	-1072.531837	-1072.603126
10	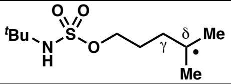	-1072.875201	-1072.557278	-1072.536536	-1072.608835
11	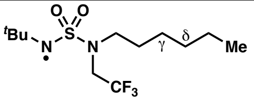	-1429.370182	-1429.005472	-1428.980228	-1429.063409

12	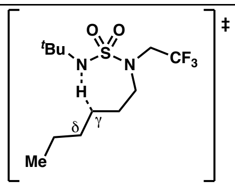	-1429.340594	-1428.98022	-1428.956321	-1429.03403
13	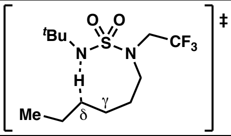	-1429.33668	-1428.975845	-1428.952239	-1429.028074
14	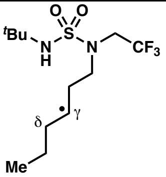	-1429.367828	-1429.004451	-1428.978892	-1429.063585
15	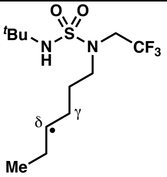	-1429.368048	-1429.004394	-1428.978925	-1429.06269
16	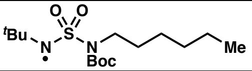	-1398.851453	-1398.393464	-1398.3647	-1398.4541
17	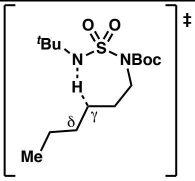	-1398.819166	-1398.365841	-1398.338331	-1398.423107
18	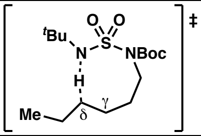	-1398.815491	-1398.361922	-1398.334535	-1398.418357
19	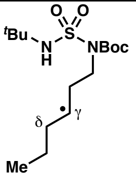	-1398.843427	-1398.386613	-1398.357576	-1398.44844
20	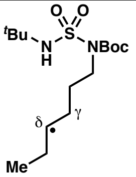	-1398.84364	-1398.386767	-1398.357735	-1398.447839
21	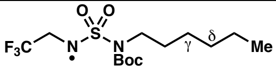	-1617.952624	-1617.637302	-1617.545982	-1617.637302

22	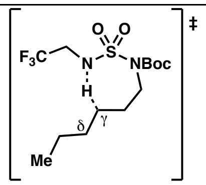	-1617.924212	-1617.549869	-1617.522918	-1617.60877
23	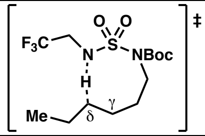	-1617.922067	-1617.54754	-1617.520787	-1617.605488
24	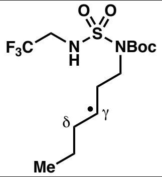	-1617.951581	-1617.573757	-1617.545288	-1617.637108
25	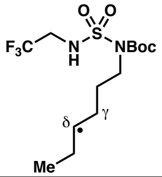	-1617.951698	-1617.573934	-1617.545422	-1617.637009
26	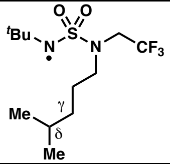	-1429.369513	-1429.005144	-1428.979957	-1429.062929
27	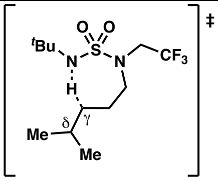	-1429.334723	-1428.974624	-1428.950768	-1429.027491
28	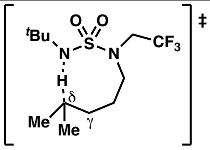	-1429.337386	-1428.976563	-1428.953032	-1429.028051
29	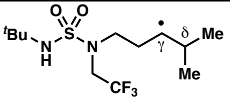	-1429.367669	-1429.004146	-1428.978771	-1429.06217
30	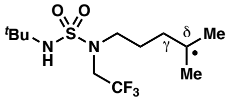	-1429.37336	-1429.010181	-1428.984453	-1429.06929
31	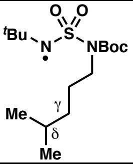	-1398.846083	-1398.38882	-1398.360001	-1398.44937

32	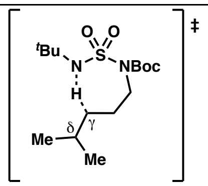	-1398.811272	-1398.358247	-1398.330722	-1398.414987
33	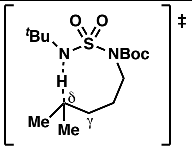	-1398.8158	-1398.362738	-1398.335325	-1398.418555
34	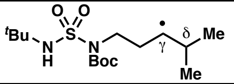	-1398.847203	-1398.390511	-1398.361581	-1398.451464
35	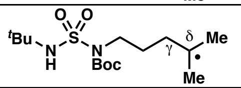	-1398.851937	-1398.395599	-1398.366286	-1398.457894
36	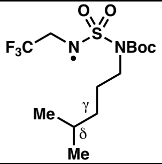	-1617.951968	-1617.573883	-1617.545747	-1617.636694
37	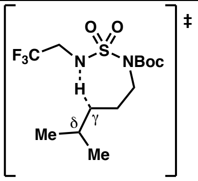	-1617.918224	-1617.544275	-1617.517293	-1617.602441
38	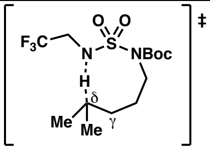	-1617.920971	-1617.547026	-1617.520237	-1617.604101
39	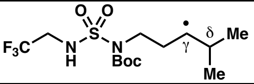	-1617.951346	-1617.573249	-1617.545000	-1617.635358
40	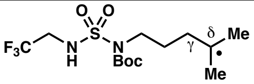	-1617.956073	-1617.578672	-1617.549928	-1617.642636

Table S4. Optimized Structures and Cartesian Coordinates
DFT Method: uB3LYP / 6-31+G(d,p) / PCM – benzene

entry	species	minimized structure
1	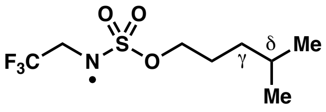	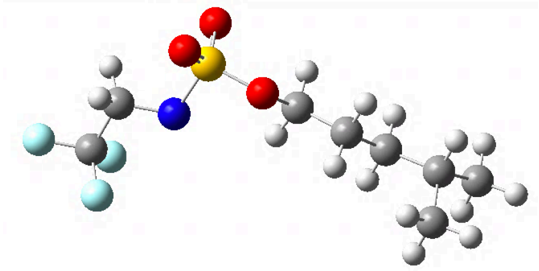

Atom	Coordinates (Angstroms) X	Y	Z
C	-1.855462	0.388382	1.932594
N	0.143206	0.554160	0.570399
S	1.489731	-0.192827	-0.146221
O	1.068573	-1.299588	-0.994717
O	2.462688	-0.388756	0.930078
C	2.551585	2.194799	-0.560902
H	1.789436	2.666323	0.065988
H	3.407110	1.902172	0.052157
C	2.953620	3.075539	-1.730314
H	3.677286	2.534432	-2.352049
H	2.069549	3.264569	-2.347785
C	3.567251	4.396183	-1.237146
H	2.819289	4.954066	-0.654812
H	4.390742	4.169824	-0.545865
C	4.109202	5.308812	-2.356760
H	4.836120	4.722619	-2.939105
C	4.852620	6.508894	-1.749531
H	5.669064	6.184568	-1.094295
H	5.282021	7.143877	-2.532280
H	4.171626	7.130116	-1.153915
C	3.006803	5.784994	-3.316180
H	2.514454	4.952946	-3.829549
H	2.236811	6.349529	-2.774525
H	3.421847	6.445012	-4.085905
C	-0.701970	-0.369002	1.288346
H	-0.146307	-0.892963	2.079948
H	-1.116685	-1.135518	0.618265
F	-2.652298	-0.477821	2.603207
F	-1.424322	1.314893	2.815886
F	-2.619137	1.022806	1.017350
O	1.964641	0.966874	-1.136087

2

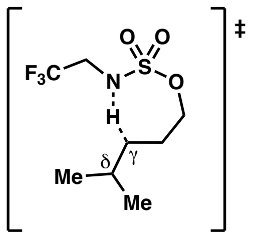

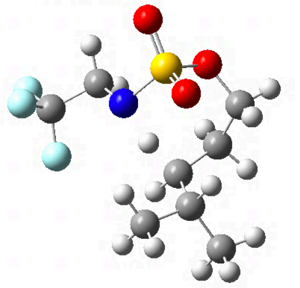

Atom	Coordinates (Angstroms) X	Y	Z
C	2.109103	1.111449	-0.000659
N	-0.123832	0.873014	-0.941745
S	-1.101009	0.149672	-2.100089
O	-0.475408	-1.089670	-2.543919
O	-2.461406	0.183310	-1.576164
C	-1.804816	2.373065	-3.400222
H	-2.806510	2.150945	-3.025363
H	-1.870793	2.627675	-4.459116
O	-1.021658	1.137295	-3.408037
C	-1.110489	3.493710	-2.622321
H	-1.642704	4.424505	-2.868004
H	-0.096438	3.613954	-3.019904
C	-1.063532	3.332718	-1.108204
H	-0.608371	2.154814	-0.892520
H	-0.259076	3.926091	-0.656783
C	-2.358322	3.418408	-0.303671
H	-3.102188	2.752728	-0.758487
C	-2.141616	2.966028	1.149785
H	-1.395932	3.597029	1.648853
H	-3.075552	3.039325	1.716184
H	-1.797567	1.929123	1.198001
C	-2.918285	4.859806	-0.341815
H	-3.146274	5.190847	-1.359667
H	-3.844820	4.910808	0.239943
H	-2.206104	5.568584	0.096977
C	1.299516	0.913876	-1.272969
H	1.565870	1.719026	-1.968904
H	1.608656	-0.036154	-1.719933
F	1.788758	2.269191	0.631756
F	3.430752	1.173715	-0.301661
F	1.940378	0.110446	0.885905

3	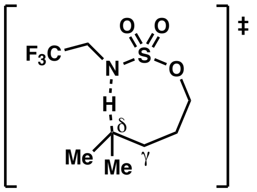	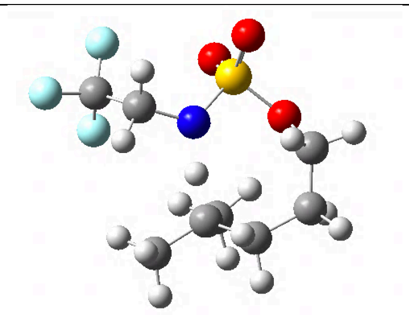

Atom	Coordinates (Angstroms) X	Y	Z
C	0.294770	0.502812	-0.115482
N	-2.242278	0.351530	0.279293
S	-2.774940	-0.729289	-0.888166
O	-2.889561	-2.025443	-0.210259
O	-2.113036	-0.653265	-2.185369
C	-5.169724	0.167854	-0.060299
H	-6.033853	-0.463758	-0.278114
H	-4.723197	-0.147618	0.885163
C	-5.550098	1.643297	-0.060603
H	-5.864379	1.922536	-1.072703
H	-6.444469	1.733071	0.569643
C	-4.496734	2.627352	0.481292
H	-4.964101	3.624282	0.518961
H	-4.268939	2.366853	1.523107
C	-3.171628	2.796905	-0.276100
H	-2.629294	1.696856	-0.131240
C	-3.282429	3.045128	-1.773064
H	-3.831353	2.254227	-2.287715
H	-2.292756	3.128045	-2.231535
H	-3.810566	3.994567	-1.947050
C	-2.238959	3.777213	0.427740
H	-2.113909	3.534262	1.488527
H	-2.661288	4.790916	0.372131
H	-1.253368	3.804955	-0.045020
C	-0.897357	0.246479	0.813059
H	-0.821158	0.973706	1.625953
H	-0.758553	-0.751059	1.249742
F	0.546063	-0.517404	-0.958517
F	1.411738	0.679234	0.641020
F	0.135847	1.621829	-0.861078
O	-4.225038	-0.075930	-1.157701

4	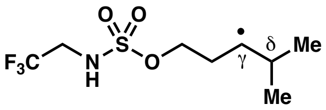	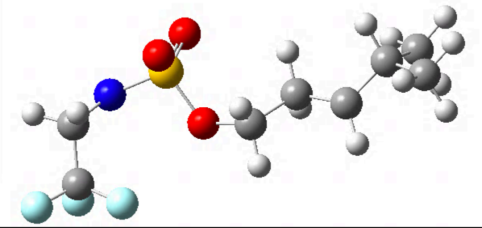

Atom Coordinates (Angstroms)

Atom	X	Y	Z
N	-0.909392	-0.611561	-0.600107
S	-2.309246	-0.051020	0.071614
O	-3.396478	-0.781778	-0.570444
O	-2.150968	-0.051763	1.524398
C	-3.157132	2.465617	0.286885
H	-3.005750	2.313940	1.358215
H	-2.712656	3.419783	-0.003807
C	-4.633934	2.397523	-0.104523
H	-4.687512	2.395920	-1.206704
H	-5.065481	1.445854	0.226651
C	-5.399852	3.546712	0.474422
H	-5.005564	4.551872	0.319640
H	-0.993245	-0.847253	-1.582992
C	-6.803499	3.405101	0.984423
H	-6.858477	2.472654	1.568425
C	-7.818013	3.262511	-0.180205
H	-7.828094	4.168639	-0.797393
H	-7.568853	2.414541	-0.826557
H	-8.830647	3.104500	0.209550
C	-7.194645	4.573343	1.904206
H	-8.213217	4.447051	2.287159
H	-6.516325	4.649043	2.760667
H	-7.158412	5.525320	1.360023
O	-2.334254	1.493215	-0.445568
C	0.408013	-0.359303	-0.051025
H	1.029430	-1.244785	-0.212107
H	0.325524	-0.195070	1.025022
C	1.148126	0.824435	-0.670838
F	0.612765	2.020384	-0.349437
F	2.436334	0.830024	-0.246234
F	1.168587	0.743892	-2.026051

5	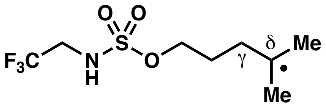	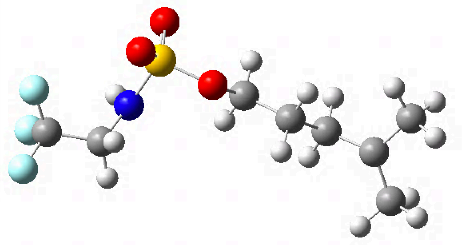

Atom	X	Y	Z
C	-0.741258	-1.232617	0.703845
N	-2.650424	0.220541	1.348003
S	-2.513145	1.879298	1.498449
O	-2.773900	2.162275	2.907129
O	-1.332652	2.363791	0.799796
C	-5.104411	2.203731	1.070259
H	-5.256918	1.123753	1.173569
H	-5.212372	2.681169	2.047934
C	-6.041995	2.807152	0.036945
H	-5.809330	3.871599	-0.074828
H	-5.867518	2.333143	-0.935310
C	-7.522841	2.624689	0.447114
H	-7.735218	1.551667	0.538828
H	-7.656437	3.054391	1.455661
C	-8.496270	3.256809	-0.511761
H	-2.738562	-0.241078	2.247433
C	-8.668495	4.745495	-0.493170
H	-7.742914	5.269455	-0.229928
H	-9.428332	5.054916	0.248762
H	-9.008942	5.127924	-1.463118
C	-9.563638	2.419619	-1.146384
H	-9.205430	1.411832	-1.387383
H	-9.942710	2.877099	-2.068260
H	-10.437895	2.292428	-0.480055
C	-2.009898	-0.507978	0.266054
H	-1.748733	0.181360	-0.538541
H	-2.692542	-1.264093	-0.133635
F	-1.001492	-2.086369	1.732471
F	-0.249282	-1.971371	-0.318639
F	0.232971	-0.401393	1.119607
O	-3.738815	2.432050	0.594010

6	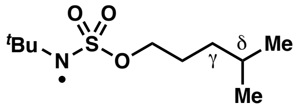	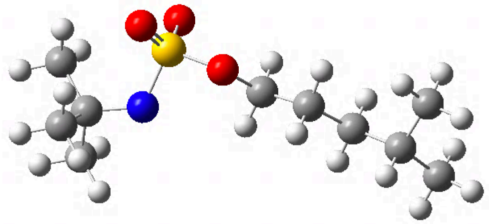

Atom	Coordinates (Angstroms) X	Y	Z
C	0.270275	-1.048418	0.522496
C	1.110647	-2.091926	1.285388
H	1.581342	-2.810937	0.610885
H	1.888595	-1.608080	1.881815
H	0.452204	-2.644608	1.962242
C	-0.785467	-1.756558	-0.376223
H	-1.453866	-2.331387	0.272973
H	-1.379950	-1.023871	-0.928577
H	-0.304672	-2.433821	-1.084756
C	-0.440887	-0.105030	1.512644
H	0.287727	0.414457	2.142100
H	-1.033400	0.642312	0.977647
H	-1.107214	-0.683368	2.160302
N	1.028708	-0.159470	-0.372499
S	2.397879	-0.734690	-1.165311
O	3.521303	-0.581652	-0.233936
O	2.175572	-2.001225	-1.857733
C	2.879159	1.736162	-1.984890
H	2.106543	2.125583	-1.315296
H	3.838833	1.711599	-1.461993
C	2.957585	2.524496	-3.281640
H	3.684695	2.039783	-3.941524
H	1.983601	2.482630	-3.784729
C	3.349343	3.987153	-3.018008
H	4.347464	4.022908	-2.556976
H	2.654434	4.414190	-2.281533
C	3.345979	4.890115	-4.268746
H	2.347673	4.818263	-4.726612
C	4.380508	4.450544	-5.317104
H	5.394375	4.475392	-4.896995
H	4.194255	3.436291	-5.684115
H	4.363940	5.121168	-6.183466
C	3.573538	6.355931	-3.867008
H	3.530566	7.016193	-4.740335
H	2.817537	6.696783	-3.150369
H	4.558754	6.484981	-3.400926
O	2.517665	0.358690	-2.343767

7	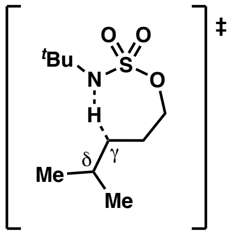	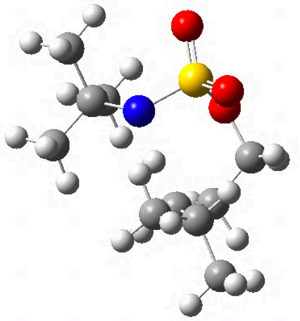

Atom	Coordinates (Angstroms) X	Y	Z
C	-1.728181	1.003267	-0.057322
C	-1.210227	1.166801	-1.497057
H	-1.470273	0.308433	-2.120442
H	-1.613235	2.069844	-1.964833
H	-0.118931	1.254945	-1.479270
C	-1.138728	-0.272256	0.589406
H	-0.048522	-0.175398	0.631578
H	-1.517527	-0.393176	1.608378
H	-1.390278	-1.163471	0.013424
C	-1.311980	2.218040	0.794045
H	-1.649437	3.158631	0.349376
H	-1.720575	2.142498	1.805844
H	-0.220771	2.254117	0.863346
N	-3.221613	0.921949	0.076999
S	-4.143051	-0.060190	-0.911664
O	-3.436215	-1.298489	-1.225751
O	-5.485468	-0.094781	-0.330249
C	-5.212238	1.806619	-2.494300
H	-6.154382	1.515755	-2.023597
H	-5.364862	1.881712	-3.572687
O	-4.262615	0.709245	-2.367066
C	-4.658282	3.122859	-1.943044
H	-5.348552	3.916081	-2.266835
H	-3.703460	3.330705	-2.438052
C	-4.483729	3.196193	-0.432278
H	-3.815951	2.123770	-0.092848
H	-3.769912	3.976936	-0.144817
C	-5.718680	3.202307	0.466905
H	-6.349594	2.344922	0.203915
C	-5.326477	3.063302	1.947025
H	-4.690115	3.899818	2.263070
H	-6.218139	3.064136	2.582499
H	-4.783186	2.131517	2.126305
C	-6.544186	4.491445	0.249391

H	-6.905382	4.585577	-0.779362
H	-7.418963	4.487155	0.908750
H	-5.950326	5.382785	0.485554

8

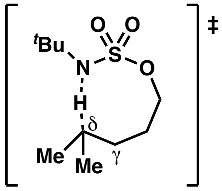

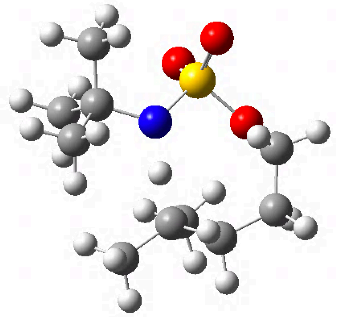

Atom	Coordinates (Angstroms) X	Y	Z
C	-0.620757	0.612072	0.010560
C	0.515080	1.199672	-0.851682
H	0.571577	0.701560	-1.821818
H	0.368798	2.270226	-1.020605
H	1.472305	1.069014	-0.335904
C	-0.349003	-0.887430	0.269036
H	0.573124	-0.969776	0.853223
H	-1.163479	-1.346862	0.833587
H	-0.208867	-1.448731	-0.657782
C	-0.699831	1.331831	1.368231
H	-0.889305	2.400112	1.248489
H	-1.497200	0.904318	1.983130
H	0.249669	1.218024	1.901280
N	-1.972759	0.798550	-0.622541
S	-2.390152	-0.204657	-1.906790
O	-1.420816	-0.265045	-3.003708
O	-2.896275	-1.462608	-1.345899
C	-4.816712	0.907075	-1.719552
H	-4.667507	0.526489	-0.706353
H	-5.630174	0.351620	-2.192734
C	-5.075365	2.405585	-1.740325
H	-5.120206	2.740910	-2.782943
H	-6.079256	2.563019	-1.325048
C	-4.087521	3.261471	-0.930674
H	-4.448658	4.302586	-0.961335
C	-2.611882	3.304166	-1.348972
H	-4.137879	2.963673	0.125035
H	-2.190606	2.094583	-1.028532
C	-1.828468	4.277150	-0.476449
H	-2.032232	4.136781	0.589122
H	-0.749498	4.206160	-0.641614
H	-2.126459	5.304915	-0.732051

C	-2.328020	3.499541	-2.830045
H	-1.251136	3.513619	-3.025056
H	-2.777827	2.724593	-3.451356
H	-2.731785	4.472066	-3.151590
O	-3.624501	0.641285	-2.527222

9	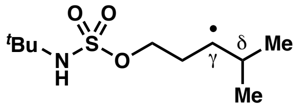	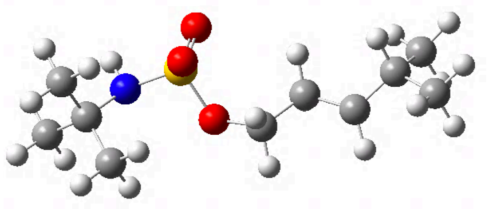

Atom	Coordinates (Angstroms) X	Y	Z
C	1.163916	-0.020978	0.211475
C	1.319786	-1.191045	1.200420
H	0.556724	-1.149552	1.980925
H	1.233122	-2.151712	0.680677
H	2.305799	-1.151924	1.675475
C	1.284264	1.329096	0.932770
H	2.283458	1.409448	1.371424
H	1.142479	2.158757	0.235090
H	0.555645	1.420938	1.742065
C	2.227616	-0.115891	-0.896301
H	2.144586	-1.059263	-1.448583
H	2.128010	0.711304	-1.604947
H	3.226820	-0.078982	-0.451948
N	-0.145188	-0.099251	-0.517151
S	-1.634553	0.175406	0.142222
O	-2.596195	-0.524979	-0.711975
O	-1.686772	-0.023707	1.594934
C	-2.674226	2.561369	0.671787
H	-2.581277	2.259331	1.717667
H	-2.301016	3.582131	0.560594
C	-4.114639	2.444580	0.167529
H	-4.098715	2.600819	-0.924728
H	-4.484355	1.425156	0.327445
C	-5.009344	3.435982	0.844468
H	-4.693896	4.480129	0.860356
H	-0.225622	-0.882966	-1.159480
C	-6.422790	3.115887	1.232064
H	-6.430280	2.110096	1.681441
C	-7.351909	3.056624	-0.008453
H	-7.405371	4.035922	-0.498687
H	-6.992136	2.328747	-0.743009
H	-8.367813	2.766782	0.285445

C	-6.966949	4.111190	2.269898
H	-7.991273	3.854167	2.561289
H	-6.348554	4.120420	3.173765
H	-6.981893	5.129274	1.861075
O	-1.729877	1.782650	-0.131831

10

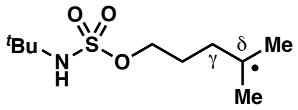

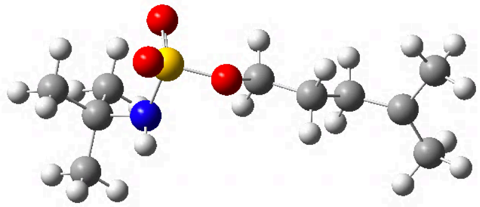

Atom	Coordinates (Angstroms) X	Y	Z
C	0.423863	-0.138292	0.436396
C	1.876777	0.285608	0.715897
H	2.308338	0.805924	-0.145060
H	1.941362	0.945008	1.585881
H	2.487005	-0.599875	0.921717
C	0.375871	-1.080594	-0.779027
H	0.943405	-1.989724	-0.560788
H	-0.654297	-1.366625	-1.013433
H	0.821739	-0.614103	-1.665367
C	-0.193591	-0.838298	1.656242
H	-0.194561	-0.187842	2.532329
H	-1.224192	-1.139336	1.443839
H	0.386567	-1.736357	1.891933
S	-0.444680	2.487092	0.862532
O	-0.451295	2.198681	2.295713
O	0.513160	3.440161	0.306045
C	-3.089478	2.449436	0.944497
H	-3.077669	1.391609	0.662913
H	-3.064008	2.534829	2.034408
C	-4.281387	3.180724	0.347735
H	-4.212010	4.243488	0.603918
H	-4.244501	3.107973	-0.744986
C	-5.616645	2.593606	0.863492
H	-5.600052	2.619055	1.967709
H	-5.669970	1.534257	0.581278
C	-6.834130	3.317982	0.354131
C	-7.176343	4.656565	0.935059
H	-7.794845	4.554828	1.846604
H	-6.285865	5.226147	1.223271
H	-7.756497	5.268542	0.233694
C	-7.903788	2.555987	-0.365846
H	-8.520195	3.214289	-0.990061

H	-7.486645	1.769922	-1.006262
H	-8.596793	2.055251	0.336851
O	-1.884610	3.081708	0.405302
N	-0.450022	1.026325	0.031674
H	-0.328937	1.251252	-0.955477

11	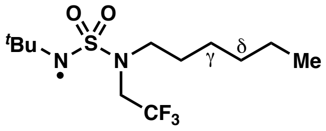	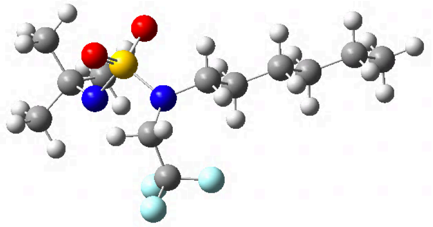

Atom	Coordinates (Angstroms) X	Y	Z
C	0.380783	-1.059623	0.000238
C	0.204910	0.483845	-0.029738
H	1.102274	0.990615	0.329712
H	-0.020243	0.827857	-1.043125
H	-0.636281	0.745901	0.620108
C	-0.909102	-1.740945	-0.493527
H	-1.149463	-1.422170	-1.511728
H	-0.797236	-2.829274	-0.489295
H	-1.744268	-1.476141	0.162516
C	0.722556	-1.536063	1.429663
H	0.901647	-2.614608	1.447372
H	1.600413	-1.025486	1.831304
H	-0.128198	-1.318059	2.082670
N	1.405289	-1.414975	-0.996931
S	3.039636	-1.240737	-0.647131
O	3.462045	-2.591121	-0.233077
O	3.404832	-0.094482	0.194335
N	3.593557	-0.878214	-2.190823
C	4.686239	0.125673	-2.313917
H	5.308174	0.100210	-1.414295
H	5.309938	-0.206309	-3.149270
C	4.178910	1.548007	-2.563805
H	3.542458	1.552234	-3.456501
H	3.552969	1.852907	-1.717959
C	5.333469	2.543194	-2.741681
H	5.973744	2.524016	-1.848108
H	5.966875	2.223236	-3.581963
C	4.856898	3.980654	-2.989716
H	4.214344	4.000635	-3.881840
H	4.224730	4.302155	-2.149473

C	6.003676	4.983814	-3.171064
H	6.646041	4.962737	-2.279797
H	6.635253	4.662303	-4.010877
C	5.519667	6.417560	-3.416903
H	4.913924	6.779922	-2.577721
H	6.361162	7.107603	-3.542376
H	4.902108	6.477193	-4.321095
C	3.506667	-1.953651	-3.171665
H	2.907484	-2.774849	-2.776855
H	4.497435	-2.351699	-3.421094
C	2.849315	-1.517660	-4.477715
F	2.761311	-2.583443	-5.314706
F	3.568549	-0.565007	-5.128102
F	1.605099	-1.027695	-4.311188

12

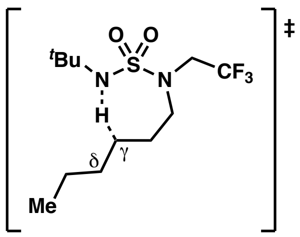

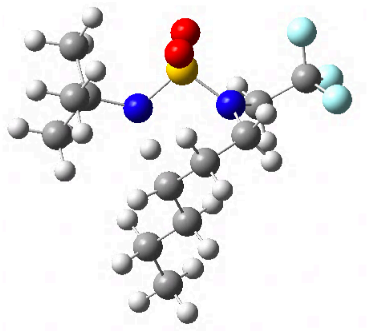

Atom	X	Y	Z
C	1.766391	2.594000	2.628364
C	0.503781	3.413013	2.302729
H	1.484648	1.684544	3.165429
H	2.360328	3.197014	3.332438
H	-0.138939	3.454628	3.187985
H	0.783234	4.440536	2.056857
S	-0.570296	1.299939	0.918501
O	-1.682676	1.172459	-0.030691
O	-0.698649	0.701465	2.256368
N	0.835130	0.821779	0.094429
C	1.188031	-0.635769	0.009602
C	2.368141	-0.695691	-0.976559
H	3.234273	-0.146520	-0.594784
H	2.671332	-1.736689	-1.127459
H	2.086843	-0.269885	-1.944045
C	1.602302	-1.253994	1.361068
H	0.788481	-1.213112	2.086790
H	1.882227	-2.301622	1.208310
H	2.469844	-0.735708	1.781648

Coordinates (Angstroms)

C	-0.005750	-1.417620	-0.580616
H	-0.346489	-0.967636	-1.516271
H	0.316248	-2.444690	-0.780995
H	-0.850642	-1.457122	0.111049
C	4.072646	2.910359	-0.582143
H	4.906137	2.341978	-0.147458
H	3.543059	2.222773	-1.252237
C	2.623117	2.227065	1.428974
H	1.812929	1.490076	0.689412
H	3.410289	1.513325	1.694092
C	3.125504	3.351627	0.542094
H	3.650848	4.076755	1.188454
H	2.280432	3.901772	0.108282
C	4.624763	4.088800	-1.391924
H	5.189786	4.779090	-0.754397
H	5.295263	3.744008	-2.185942
H	3.815968	4.658640	-1.864354
N	-0.268203	2.931969	1.131957
C	-0.780440	3.878005	0.159461
H	-0.005828	4.599506	-0.123647
H	-1.104763	3.348483	-0.737098
C	-1.980369	4.685539	0.656721
F	-3.018771	3.915812	1.033709
F	-1.664406	5.474527	1.720041
F	-2.415721	5.512149	-0.329689

13

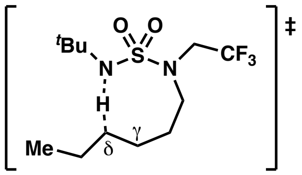

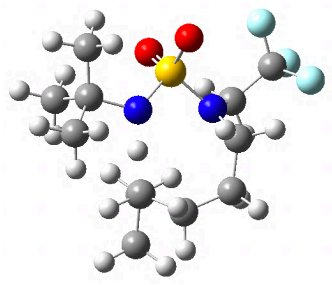

Atom	Coordinates (Angstroms) X	Y	Z
C	0.481685	4.661034	0.456634
C	1.896941	4.374843	-0.033620
H	0.479558	4.599069	1.552314
H	2.624463	4.990870	0.508494
H	0.248358	5.711488	0.221076
C	-0.592677	2.290551	0.253423
H	-0.375740	2.155534	1.314763
H	-1.561398	1.819248	0.064512
C	-0.661290	3.786870	-0.091930

H	-0.752818	3.912911	-1.178240
H	-1.602112	4.166590	0.325917
C	3.600460	2.027611	1.875363
C	3.590462	3.146642	2.933056
H	4.551131	3.167903	3.457621
H	3.436161	4.129453	2.478410
H	2.796649	2.976309	3.666008
C	4.740504	2.257649	0.861464
H	5.704818	2.229942	1.379762
H	4.739651	1.488335	0.086235
H	4.646397	3.236434	0.380412
C	3.801880	0.671999	2.586765
H	4.733134	0.722923	3.160181
H	2.978345	0.457619	3.271361
H	3.886090	-0.155381	1.878603
N	2.247820	2.091135	1.226058
H	2.150193	3.209276	0.491954
C	2.193951	4.357982	-1.522926
H	3.194692	3.939912	-1.684105
H	1.497260	3.698108	-2.047902
C	2.137097	5.764075	-2.155247
H	2.397755	5.714511	-3.217727
H	1.136900	6.202751	-2.076146
H	2.842830	6.445730	-1.667535
S	1.713690	0.802447	0.288775
O	1.212559	-0.209337	1.231866
O	2.641443	0.353675	-0.765854
N	0.449447	1.571956	-0.516350
C	-0.705002	0.007081	-2.133050
C	0.203394	1.217385	-1.900932
H	1.149946	1.002981	-2.399850
H	-0.269047	2.062895	-2.409971
F	-0.231017	-1.124600	-1.576739
F	-1.962844	0.192306	-1.648902
F	-0.828816	-0.213474	-3.469156

14	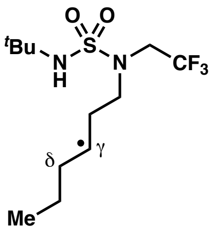	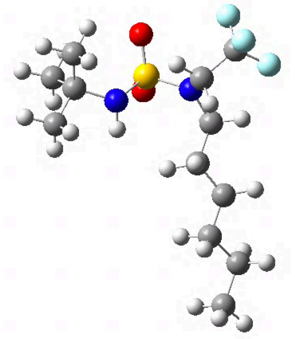

Atom Coordinates (Angstroms)
X Y Z

C	-0.176569	-0.929341	-0.000033
C	-0.191111	-2.206242	0.859046
H	0.221031	-2.022805	1.854793
H	-1.208213	-2.594781	0.972426
H	0.419073	-2.979444	0.380699
C	1.247351	-0.368894	-0.133655
H	1.889093	-1.114613	-0.614220
H	1.246453	0.533716	-0.753130
H	1.672074	-0.123253	0.840841
C	-0.743482	-1.232627	-1.398524
H	-1.756323	-1.648754	-1.338522
H	-0.770804	-0.328844	-2.015588
H	-0.110608	-1.970669	-1.899684
S	-1.134836	0.553516	2.188213
O	-2.105001	-0.269132	2.920966
O	0.257365	0.633079	2.629349
N	-1.775278	2.104914	2.180526
C	-3.233992	2.299799	2.339044
H	-3.567946	1.647905	3.146420
H	-3.362215	3.329558	2.682488
C	-4.075693	2.058566	1.072898
H	-3.605704	2.582481	0.218928
H	-4.059827	0.990415	0.814458
N	-1.089337	0.148551	0.541361
H	-2.052588	-0.029907	0.258895
C	-5.492373	2.507707	1.240690
H	-5.676528	3.420014	1.807925
C	-6.603035	1.943925	0.415259
H	-6.514254	0.846290	0.377326
H	-6.494651	2.271701	-0.637521
C	-8.008023	2.329803	0.904441
H	-8.087354	3.424821	0.935452
H	-8.134156	1.982066	1.937845
C	-9.127569	1.759375	0.027607
H	-9.092236	0.663688	0.002735
H	-10.114113	2.051427	0.402815
H	-9.044372	2.117724	-1.005481
C	-0.999344	3.133699	1.505097
H	-0.119083	2.700248	1.027317
H	-1.589712	3.627821	0.725879
C	-0.501064	4.221261	2.454685
F	0.285600	3.749451	3.438650
F	0.221071	5.136319	1.754146
F	-1.521453	4.897060	3.047865

15	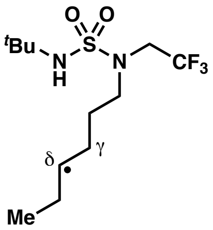	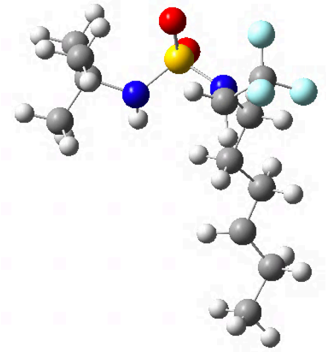

Atom Coordinates (Angstroms)

Atom	X	Y	Z
C	1.643351	-2.590077	-0.825143
C	1.642303	-4.031383	-0.285351
H	1.976653	-4.069251	0.754830
H	0.642836	-4.474278	-0.342685
H	2.324561	-4.648121	-0.879630
C	3.040793	-1.961237	-0.718071
H	3.749837	-2.547257	-1.311938
H	3.030799	-0.937897	-1.106728
H	3.390114	-1.938396	0.315216
C	1.187147	-2.580669	-2.295119
H	0.195484	-3.034341	-2.410035
H	1.154078	-1.560064	-2.689467
H	1.889497	-3.159909	-2.901407
S	0.471810	-1.688086	1.565381
O	-0.470473	-2.714005	2.029604
O	1.827747	-1.626046	2.110073
C	-1.760940	-0.162624	1.975857
H	-2.066329	-0.995128	2.611569
H	-1.978193	0.756606	2.526687
C	-2.535657	-0.183703	0.652283
H	-2.147208	0.583212	-0.029791
H	-2.395528	-1.154491	0.159264
N	0.639360	-1.704129	-0.122145
H	-0.293467	-1.851989	-0.506104
N	-0.285932	-0.219127	1.852925
C	0.434371	0.982983	1.462004
H	1.361264	0.725049	0.946833
H	-0.162467	1.598213	0.779999
C	0.814669	1.862830	2.651264
C	-4.849466	-0.026925	-0.387081
H	-4.506106	-0.689127	-1.182267
C	-6.257701	0.472241	-0.440566
H	-6.316343	1.460696	0.040534
H	-6.908220	-0.178434	0.174928

C	-6.837727	0.549216	-1.859588
H	-6.823947	-0.434400	-2.343073
H	-7.875127	0.899034	-1.844190
H	-6.257913	1.237216	-2.484682
F	1.489751	2.956424	2.206357
F	-0.274530	2.320913	3.324900
F	1.599662	1.237890	3.547417
C	-4.040587	0.047734	0.868753
H	-4.197823	1.023954	1.352788
H	-4.409452	-0.696709	1.600743

16

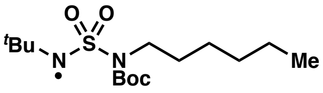

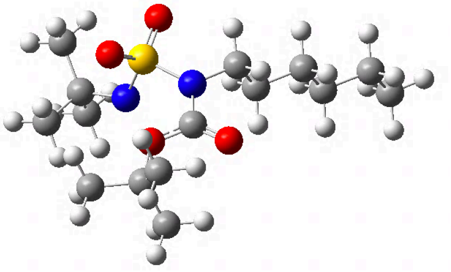

Atom	Coordinates (Angstroms) X	Y	Z
N	-0.006407	1.320439	-0.972133
S	1.481679	0.888931	-0.299540
O	1.494816	-0.507797	0.137390
O	1.858338	1.944276	0.649378
N	2.533728	1.092298	-1.626757
C	3.423073	2.284577	-1.658636
H	3.699130	2.514300	-0.629929
H	4.320103	1.964387	-2.190986
C	2.790737	3.499181	-2.342495
H	2.477619	3.217248	-3.355153
H	1.887135	3.795059	-1.795660
C	3.764118	4.683424	-2.413095
H	4.085513	4.953450	-1.396866
H	4.671573	4.376788	-2.952728
C	4.128629	7.107965	-3.175542
H	5.036178	6.801382	-3.713861
H	4.451781	7.377847	-2.160551
C	1.427299	-1.718163	-3.735947
C	0.268301	-2.560167	-3.201197
H	-0.645691	-1.964055	-3.131648
H	0.082875	-3.399043	-3.878646
H	0.503161	-2.957102	-2.209747
C	2.716455	-2.541846	-3.791066
H	3.553123	-1.954776	-4.171806
H	2.968856	-2.923340	-2.796810

H	2.562712	-3.398671	-4.455033
C	1.069355	-1.083439	-5.081959
H	0.183231	-0.448804	-4.981298
H	1.892554	-0.485172	-5.474365
H	0.839341	-1.876564	-5.801003
C	2.513215	0.269692	-2.765838
O	3.303105	0.436786	-3.679302
O	1.555653	-0.654670	-2.698512
C	3.162840	5.918233	-3.097622
H	2.254390	6.224882	-2.559013
H	2.839959	5.647585	-4.113368
C	3.520166	8.337124	-3.860676
H	2.629945	8.688423	-3.325209
H	4.233873	9.167291	-3.900394
H	3.218406	8.107660	-4.889693
C	-1.237307	1.139240	-0.179181
C	-2.183092	2.257762	-0.664355
H	-2.293935	2.227405	-1.751718
H	-3.168878	2.129995	-0.205927
H	-1.795297	3.240944	-0.381912
C	-1.086091	1.196076	1.353435
H	-0.461005	0.384718	1.733543
H	-0.655416	2.148024	1.673018
H	-2.078171	1.097467	1.804538
C	-1.803291	-0.244427	-0.608393
H	-1.933187	-0.291309	-1.692409
H	-1.138066	-1.050664	-0.292934
H	-2.780116	-0.376330	-0.131739

17

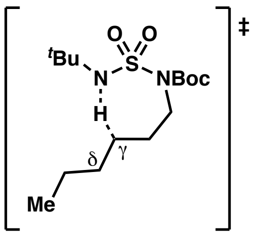

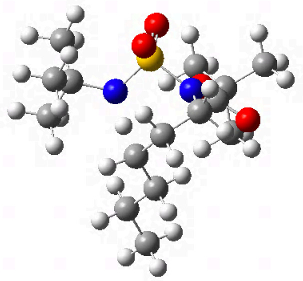

Atom	Coordinates (Angstroms) X	Y	Z
C	1.440738	0.501914	-0.000226
C	0.116094	1.259357	-0.245403
H	1.223895	-0.488972	0.405236
H	1.982898	1.050063	0.785866
H	-0.602372	1.004933	0.536912
H	0.268140	2.337202	-0.225825
S	-0.759206	-0.645303	-1.999835
O	-1.889197	-0.773804	-2.917031

O	-0.853123	-1.340130	-0.706047
N	0.678483	-0.925696	-2.833472
C	3.768283	1.390361	-3.089948
H	4.593496	0.729753	-2.790134
H	3.215621	0.861098	-3.875270
C	2.344238	0.355127	-1.213435
H	1.595640	-0.296769	-2.092986
H	3.147074	-0.368983	-1.035578
C	2.843004	1.620204	-1.887139
H	3.391549	2.196995	-1.121316
H	2.003575	2.259426	-2.184642
C	4.339218	2.695246	-3.656653
H	4.930390	3.229895	-2.903783
H	4.989566	2.503045	-4.516537
H	3.538381	3.366811	-3.987173
N	-0.496495	0.998447	-1.574943
C	-0.730561	2.095266	-2.426402
O	-0.653713	3.243902	-2.017547
O	-1.007647	1.716090	-3.668769
C	-1.469181	2.682718	-4.701913
C	-0.352050	3.682469	-5.012517
H	-0.650807	4.298899	-5.866857
H	-0.152764	4.336645	-4.162814
H	0.568207	3.154259	-5.281686
C	-2.757821	3.362476	-4.232733
H	-2.579956	4.012597	-3.374671
H	-3.159097	3.969914	-5.050439
H	-3.509018	2.612932	-3.965404
C	-1.736226	1.770891	-5.900712
H	-2.088092	2.369728	-6.746307
H	-0.822604	1.249104	-6.199923
H	-2.497335	1.024859	-5.657502
C	1.111921	-2.334195	-3.123919
C	-0.000327	-3.057383	-3.914560
H	-0.309380	-2.469729	-4.782464
H	0.389862	-4.019683	-4.261846
H	-0.881697	-3.250983	-3.298645
C	1.473354	-3.145359	-1.862730
C	2.351763	-2.181901	-4.022809
H	2.107838	-1.615889	-4.926322
H	3.162276	-1.666146	-3.499230
H	2.717429	-3.170739	-4.317224
H	0.612801	-3.269379	-1.203019
H	2.273801	-2.658120	-1.297239
H	1.830376	-4.136779	-2.160718

18

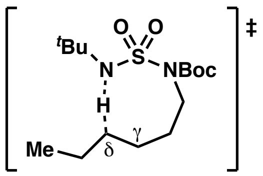

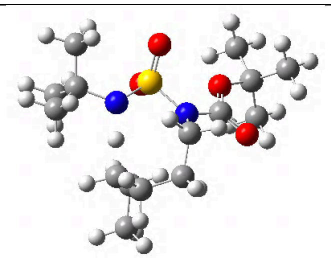

Atom	X	Y	Z
C	-2.221695	0.984409	0.000364
C	-0.795483	0.945940	-0.530879
H	-2.200187	0.734382	1.068963
H	-0.112133	1.505725	0.119015
H	-2.578245	2.025506	-0.051664
C	-2.975107	-1.422997	-0.673804
H	-2.619939	-1.736860	0.307315
H	-3.898973	-1.961508	-0.892932
C	-3.253037	0.087905	-0.706310
H	-3.398374	0.395410	-1.746431
H	-4.219771	0.240680	-0.209900
N	-0.237135	-1.543116	0.166843
H	-0.440305	-0.274305	-0.249937
C	-0.531695	1.234142	-1.998382
H	0.495348	0.940479	-2.243334
H	-1.184942	0.626154	-2.630852
C	-0.720773	2.725444	-2.345936
H	-0.482158	2.901213	-3.400138
H	-1.752388	3.052009	-2.178207
H	-0.063147	3.360747	-1.741717
S	-0.419923	-2.486989	-1.207909
O	-0.560044	-3.891397	-0.797806
O	0.541871	-2.162929	-2.267107
N	-1.976933	-1.874109	-1.687865
C	-2.512563	-2.213865	-2.941582
O	-3.620221	-1.823028	-3.277916
O	-1.693097	-2.986346	-3.649916
C	-2.001625	-3.408422	-5.041847
C	-0.762811	-4.226094	-5.412137
H	-0.865730	-4.607145	-6.432799
H	0.138379	-3.609615	-5.355918
H	-0.643801	-5.074727	-4.732609
C	-2.134535	-2.174763	-5.938094
H	-2.231200	-2.498387	-6.979651
H	-3.012688	-1.582111	-5.676873
H	-1.241096	-1.547596	-5.858727

(Coordinates in Angstroms)

C	-3.258234	-4.282876	-5.048469
H	-4.146849	-3.709185	-4.781606
H	-3.398416	-4.699424	-6.051391
H	-3.146494	-5.115632	-4.347045
C	1.059216	-1.590347	0.931229
C	0.779193	-0.783678	2.213422
H	1.671890	-0.779618	2.846885
H	0.524251	0.255956	1.988757
H	-0.045639	-1.228978	2.777084
C	2.239821	-0.966927	0.159109
H	3.132472	-0.976133	0.793340
H	2.457720	-1.520338	-0.756275
H	2.029529	0.072587	-0.110761
C	1.399914	-3.043527	1.326339
H	2.245463	-3.019059	2.021500
H	0.553569	-3.525724	1.821425
H	1.682981	-3.650308	0.464300

19

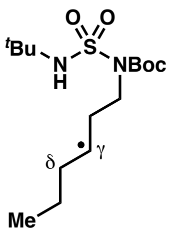

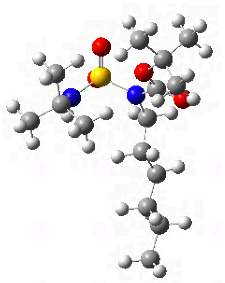

Atom	Coordinates (Angstroms) X	Y	Z
S	-2.109665	1.505576	0.000000
O	-2.888668	1.119569	1.179354
O	-0.707164	1.873920	0.113968
N	-2.979101	2.878723	-0.636134
C	-4.419855	3.001225	-0.291698
H	-4.602443	2.386066	0.586869
H	-4.587856	4.043930	-0.016461
C	-5.356583	2.603675	-1.442667
H	-5.056387	3.163469	-2.346423
H	-5.220328	1.540844	-1.689449
N	-2.258795	0.199270	-1.012735
H	-3.183878	-0.202584	-0.887891
C	-6.791996	2.870283	-1.120576
H	-7.026880	3.747279	-0.517455
C	-7.901526	2.211089	-1.874428
H	-7.683426	1.137321	-1.991531
H	-7.939237	2.605374	-2.909242

	Atom	X	Y	Z
	C	-9.288383	2.387469	-1.235611
	H	-9.496561	3.460240	-1.125851
	H	-9.269558	1.971978	-0.219738
	C	-10.411328	1.727509	-2.042654
	H	-10.246088	0.647992	-2.142735
	H	-11.384651	1.871243	-1.561449
	H	-10.473760	2.148527	-3.053325
	C	-1.653298	-0.066868	-2.358169
	C	-2.025867	-1.533991	-2.644824
	C	-2.425568	3.950593	-1.354269
	O	-3.066499	4.973629	-1.541743
	O	-1.198119	3.701968	-1.812957
	C	-0.361806	4.782241	-2.410213
	C	0.966628	4.066903	-2.662519
	H	1.683473	4.769391	-3.098167
	H	1.381072	3.678549	-1.728363
	H	0.834885	3.234307	-3.359701
	C	-0.984980	5.257459	-3.725683
	H	-1.149994	4.410852	-4.399927
	H	-1.933539	5.768359	-3.557348
	H	-0.294822	5.950849	-4.217737
	C	-0.182344	5.916168	-1.397557
	H	0.542254	6.635142	-1.793318
	H	-1.120362	6.440012	-1.208745
	H	0.207483	5.525820	-0.452448
	H	-1.622088	-2.197169	-1.874347
	H	-3.113207	-1.669490	-2.688954
	H	-1.614889	-1.833996	-3.613162
	C	-0.125705	0.071364	-2.292876
	H	0.287493	-0.574719	-1.513314
	H	0.298512	-0.229325	-3.256261
	H	0.179053	1.096940	-2.083485
	C	-2.244783	0.842233	-3.449956
	H	-1.857320	0.543410	-4.429822
	H	-3.336450	0.761450	-3.479832
	H	-1.974514	1.886880	-3.285566

20

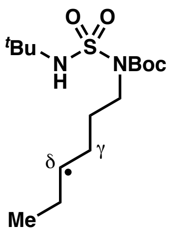

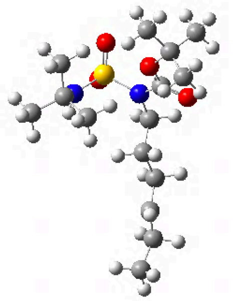

	Atom	Coordinates (Angstroms) X	Y	Z

	S 0.771961 -0.603724 -0.013539
	O 0.058792 -0.991491 1.206590
	O 2.184338 -0.257518 0.020898
	N -0.112671 0.790063 -0.579785
	C -1.524813 0.936148 -0.136147
	H -1.645656 0.337100 0.765020
	H -1.657110 1.985084 0.135776
	C -2.543267 0.530844 -1.205773
	H -2.368044 1.116362 -2.115806
	H -2.406276 -0.525034 -1.470551
	N 0.542657 -1.897436 -1.026971
	H -0.377132 -2.290154 -0.846284
	C -5.021161 0.380461 -1.743433
	H -4.765967 0.486963 -2.798168
	C -6.463446 0.237677 -1.376029
	H -6.885846 1.230397 -1.127516
	H -6.550387 -0.343674 -0.445277
	C -7.325508 -0.403787 -2.472280
	H -6.980134 -1.418106 -2.700951
	H -8.374911 -0.464957 -2.165537
	H -7.281433 0.180937 -3.398380
	C 1.058778 -2.161135 -2.409642
	C 0.661272 -3.625463 -2.676659
	C 0.408130 1.852705 -1.334525
	O -0.228147 2.885272 -1.481625
	O 1.600262 1.586658 -1.870972
	C 2.411544 2.656770 -2.519324
	C 3.709235 1.923395 -2.863362
	H 4.406033 2.618604 -3.341209
	H 4.179261 1.522285 -1.961424
	H 3.519104 1.098209 -3.555798
	C 1.709964 3.148491 -3.788481
	H 1.485259 2.307794 -4.452792
	H 0.784062 3.675109 -3.555383
	H 2.377957 3.831696 -4.323466
	C 2.675750 3.782390 -1.516027
	H 3.381989 4.493387 -1.956829
	H 1.760286 4.318213 -1.261443
	H 3.123190 3.381242 -0.601457
	C -3.987650 0.749238 -0.727156
	H -4.104800 1.811878 -0.438505
	H -4.163029 0.182255 0.200489
	H 1.110973 -4.293353 -1.936273
	H -0.427226 -3.755619 -2.650832
	H 1.007339 -3.923498 -3.670654

C	2.587947	-2.030663	-2.443566
H	3.047860	-2.679998	-1.693456
H	2.946577	-2.332081	-3.433018
H	2.911397	-1.007104	-2.253712
C	0.401504	-1.245487	-3.457525
H	0.724487	-1.541549	-4.461353
H	-0.690074	-1.322694	-3.417883
H	0.685378	-0.202371	-3.306911

21	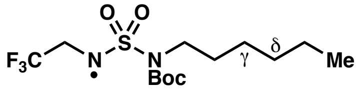	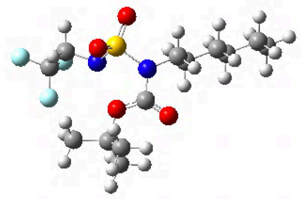

Atom	Coordinates (Angstroms)		
	X	Y	Z
C	0.419216	0.204566	-0.000359
N	2.841017	0.297796	-0.087539
S	4.340188	-0.061405	0.645318
O	4.352985	-1.436449	1.138992
O	4.596634	1.047114	1.569474
N	5.412955	0.131313	-0.642561
C	6.349220	1.290266	-0.644141
H	6.607436	1.504802	0.392922
H	7.246098	0.932662	-1.152674
C	5.780278	2.528367	-1.339427
H	5.490206	2.263062	-2.363317
H	4.871067	2.851942	-0.818519
C	6.796221	3.678342	-1.370302
H	7.095265	3.929046	-0.342437
H	7.708457	3.344909	-1.885287
C	7.265079	6.094976	-2.097282
H	8.177720	5.763022	-2.611409
H	7.565987	6.343939	-1.070183
C	1.719624	-0.113135	0.724972
H	1.712172	0.416324	1.689615
H	1.749902	-1.189095	0.943699
C	4.144253	-2.599638	-2.773615
C	2.908077	-3.329985	-2.248479
H	2.056843	-2.647404	-2.172534
H	2.641840	-4.137931	-2.936286
H	3.103201	-3.762916	-1.263243
C	5.356676	-3.531338	-2.822813
H	6.244975	-3.016401	-3.191725
H	5.565538	-3.941115	-1.829839

H	5.135834	-4.366266	-3.495510
C	3.857212	-1.920614	-4.113966
H	3.027425	-1.214024	-4.015464
H	4.733448	-1.392529	-4.492356
H	3.568508	-2.683731	-4.843979
C	5.380948	-0.700884	-1.782138
O	6.202672	-0.584301	-2.670849
O	4.364304	-1.557009	-1.724096
F	-0.634573	-0.174850	0.762926
F	0.284473	1.523058	-0.255738
F	0.323525	-0.450232	-1.179497
C	6.257560	4.938299	-2.061143
H	5.343811	5.270370	-1.547530
H	5.957528	4.687580	-3.088829
C	6.719115	7.350517	-2.787139
H	5.825329	7.726339	-2.274939
H	7.461209	8.156263	-2.796237
H	6.441390	7.141476	-3.827156

22

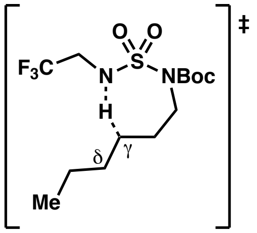

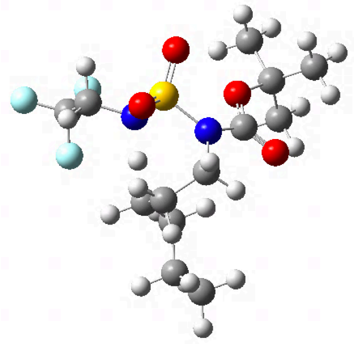

Atom	Coordinates (Angstroms) X	Y	Z
C	0.358489	-1.635747	-0.506803
C	-0.863549	-0.687880	-0.538029
H	0.118389	-2.517227	0.091510
H	1.156230	-1.104542	0.034194
H	-1.382791	-0.732952	0.421554
H	-0.560409	0.343545	-0.708583
S	-2.418632	-2.554632	-1.778275
O	-3.753675	-2.616797	-2.362495
O	-2.213666	-3.161705	-0.456600
N	-1.301759	-3.124232	-2.915104
C	2.796753	-0.509576	-2.463156
H	2.745314	-0.033581	-1.475937
H	3.499894	-1.347411	-2.370001
C	0.889332	-2.089034	-1.856956
H	-0.131486	-2.639313	-2.445448

H	1.559630	-2.950997	-1.762814
C	1.402458	-1.058642	-2.846770
H	0.701537	-0.220676	-2.933658
H	1.472668	-1.517571	-3.841081
C	3.326250	0.498042	-3.489936
H	2.655482	1.359795	-3.581208
H	4.313915	0.871504	-3.199017
H	3.419965	0.040401	-4.481441
N	-1.842304	-0.957643	-1.629320
C	-2.092252	0.070502	-2.570162
O	-1.685698	1.205248	-2.384273
O	-2.778107	-0.371413	-3.612942
C	-3.263467	0.539528	-4.690281
C	-2.066585	1.154882	-5.418517
H	-2.427671	1.713332	-6.288291
H	-1.511876	1.837474	-4.773243
H	-1.392298	0.370331	-5.775723
C	-4.198811	1.590726	-4.089353
H	-3.662978	2.280257	-3.435163
H	-4.654856	2.166339	-4.901273
H	-5.001473	1.108709	-3.522747
C	-4.027542	-0.421479	-5.601935
H	-4.433523	0.130020	-6.455298
H	-3.365586	-1.207074	-5.976792
H	-4.854208	-0.892189	-5.063179
C	-1.258292	-4.584528	-2.990803
H	-0.761941	-5.055314	-2.133245
H	-2.273193	-4.992711	-3.058573
C	-0.520446	-5.004636	-4.251867
F	0.751940	-4.530645	-4.277990
F	-1.127029	-4.580494	-5.379789
F	-0.444070	-6.357512	-4.315137

23

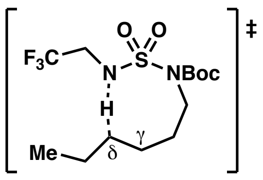

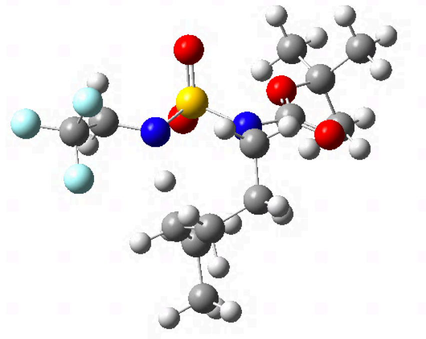

Atom	Coordinates (Angstroms) X	Y	Z
C	-0.852979	-0.102643	0.000349
C	0.547290	0.020208	-0.585228

H	-0.772857	-0.560897	0.993933
H	1.254313	0.431382	0.145619
H	-1.235852	0.915351	0.174778
C	-1.616048	-2.360776	-1.083191
H	-1.193601	-2.842453	-0.201920
H	-2.550549	-2.865357	-1.333192
C	-1.898850	-0.870689	-0.828137
H	-2.085206	-0.379193	-1.788093
H	-2.847949	-0.824910	-0.279641
N	1.153049	-2.546926	-0.471967
H	0.926316	-1.196001	-0.596128
C	0.737853	0.623883	-1.965202
H	1.757625	0.418371	-2.310342
H	0.065715	0.151429	-2.688006
C	0.508479	2.150180	-1.969426
H	0.696780	2.556382	-2.968586
H	-0.519845	2.404500	-1.692734
H	1.182196	2.654513	-1.267814
S	0.909455	-3.191398	-2.009420
O	0.938810	-4.652882	-1.871899
O	1.784697	-2.563585	-3.003035
N	-0.697913	-2.615838	-2.235071
C	-1.315841	-2.747477	-3.494186
O	-2.455552	-2.354412	-3.677317
O	-0.517481	-3.339861	-4.376046
C	-0.896411	-3.511692	-5.807725
C	0.349353	-4.178814	-6.392099
H	0.196821	-4.367895	-7.458976
H	1.225549	-3.536027	-6.271452
H	0.547314	-5.131926	-5.893717
C	-1.137422	-2.140517	-6.442654
H	-1.287606	-2.268599	-7.519505
H	-2.021035	-1.654253	-6.026147
H	-0.267342	-1.493004	-6.296074
C	-2.113932	-4.433624	-5.905250
H	-3.005477	-3.966002	-5.484937
H	-2.303844	-4.663631	-6.958766
H	-1.922619	-5.375126	-5.381111
C	2.517274	-2.774953	0.004366
H	3.241283	-2.052218	-0.390974
H	2.859783	-3.779486	-0.267638
C	2.544523	-2.690255	1.522819
F	3.811543	-2.867839	1.972687
F	1.770002	-3.626046	2.110501
F	2.125295	-1.481298	1.976832

24	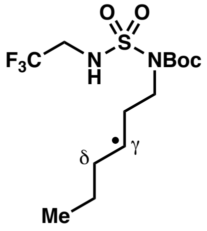	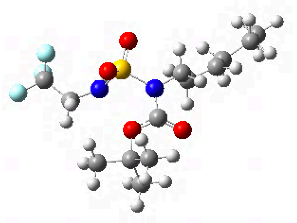

Atom	X	Y	Z
S	-0.102612	0.557737	-0.000026
O	-1.163759	-0.232201	0.621884
O	1.207168	0.701572	0.623722
N	-0.809773	2.102195	-0.225439
C	-2.191614	2.340414	0.267059
H	-2.358457	1.691073	1.124643
H	-2.211530	3.376852	0.606859
C	-3.264295	2.108841	-0.806737
H	-2.983772	2.690450	-1.703502
H	-3.261025	1.053280	-1.110169
N	0.134204	-0.013928	-1.555412
H	-0.673584	-0.528506	-1.893776
C	-4.628324	2.500353	-0.335689
H	-4.719924	3.400052	0.273112
C	-5.867116	1.927030	-0.944139
H	-5.751892	0.837565	-1.059474
H	-5.990823	2.308897	-1.976952
C	-7.152312	2.228615	-0.156858
H	-7.257108	3.316473	-0.047278
H	-7.051959	1.824702	0.858874
C	-8.410853	1.656607	-0.817754
H	-8.347483	0.566044	-0.913371
H	-9.307495	1.887256	-0.232578
H	-8.553621	2.069979	-1.823445
C	1.437040	-0.446918	-2.023051
H	2.210961	0.090519	-1.475520
H	1.536107	-0.216900	-3.087973
C	1.679652	-1.943767	-1.858321
F	0.729807	-2.671389	-2.506840
F	2.879066	-2.283890	-2.389522
F	1.671268	-2.343395	-0.570645
C	-0.145300	3.175180	-0.839821
O	-0.662259	4.276393	-0.919624
O	1.055768	2.823754	-1.304568
C	1.995840	3.840145	-1.864558
C	3.234001	3.002489	-2.187393

H	4.018258	3.650108	-2.589980
H	3.618093	2.513157	-1.287852
H	3.005589	2.239243	-2.937220
C	1.401405	4.450180	-3.136159
H	1.126467	3.663701	-3.846304
H	0.521110	5.055153	-2.916798
H	2.154477	5.087190	-3.611498
C	2.314064	4.887480	-0.794776
H	3.102078	5.549265	-1.168247
H	1.440151	5.493616	-0.552808
H	2.679322	4.404204	0.116613

25	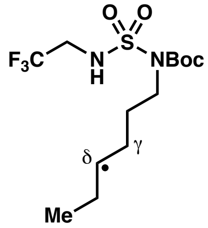	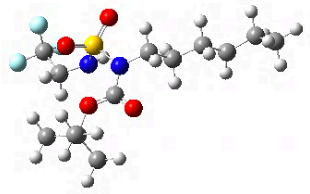

Center Number | Atomic Number | Atomic Type | Coordinates (Angstroms) X Y Z

Atom	X	Y	Z
S	0.548313	0.204289	-0.000145
O	-0.465194	-0.583495	0.699656
O	1.899173	0.348926	0.529220
N	-0.175010	1.746813	-0.180720
C	-1.510504	1.991644	0.426278
H	-1.600564	1.345326	1.298788
H	-1.495652	3.028677	0.765702
C	-2.667562	1.759072	-0.547539
H	-2.522756	2.386981	-1.434838
H	-2.664356	0.714002	-0.879206
N	0.677242	-0.369938	-1.567330
H	-0.157204	-0.873576	-1.853625
C	-5.185866	1.907098	-0.833561
H	-5.024371	2.081486	-1.897608
C	-6.592251	1.858274	-0.328739
H	-6.886548	2.853565	0.056678
H	-6.645752	1.192897	0.546689
C	-7.617050	1.414781	-1.381954
H	-7.398210	0.403300	-1.741533
H	-8.632197	1.415561	-0.971415
H	-7.605968	2.087076	-2.247661
C	1.942203	-0.825079	-2.112314
H	2.756387	-0.295656	-1.617862
H	1.977660	-0.602598	-3.182863
C	2.174112	-2.324228	-1.952752
F	1.178398	-3.042711	-2.539299

F	3.335616	-2.683584	-2.551237
F	2.236494	-2.716135	-0.664027
C	0.441736	2.818124	-0.845811
O	-0.079900	3.919130	-0.889910
O	1.604803	2.465383	-1.397760
C	2.494312	3.478074	-2.040566
C	3.703370	2.638529	-2.455519
H	4.451807	3.282833	-2.925887
H	4.159472	2.157139	-1.585785
H	3.415378	1.868665	-3.177533
C	1.797791	4.076420	-3.265097
H	1.465204	3.283416	-3.942576
H	0.938731	4.684690	-2.980399
H	2.509545	4.707827	-3.806881
C	2.896211	4.534977	-1.009028
H	3.649930	5.194761	-1.450683
H	2.043402	5.141724	-0.701959
H	3.335315	4.060071	-0.126260
C	-4.026272	2.082928	0.094509
H	-3.999352	3.124186	0.471050
H	-4.172035	1.459164	0.989991

26

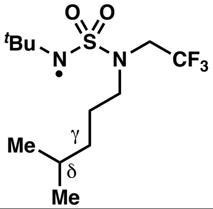

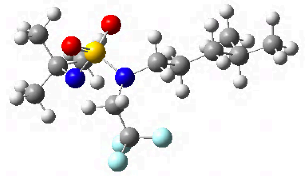

Atom	Coordinates (Angstroms)		
	X	Y	Z
C	1.105909	-0.538957	-0.000122
C	0.751193	0.964031	-0.172787
H	1.552946	1.603948	0.200041
H	0.563424	1.198870	-1.224257
H	-0.161349	1.168459	0.396415
C	-0.053994	-1.410914	-0.516053
H	-0.254254	-1.203335	-1.570992
H	0.185254	-2.473420	-0.411960
H	-0.961062	-1.202374	0.059982
C	1.394655	-0.857642	1.483696
H	1.698714	-1.900866	1.605318
H	2.173447	-0.212584	1.896076
H	0.477965	-0.697057	2.059475
N	2.235736	-0.837317	-0.897396
S	3.807451	-0.444209	-0.452200
O	4.342496	-1.694554	0.117253
O	3.973617	0.804870	0.300985

N	4.439110	-0.151314	-1.981347
C	5.451876	0.933519	-2.108001
H	5.989804	1.043631	-1.161714
H	6.175275	0.589112	-2.853167
C	4.849495	2.275964	-2.530909
H	4.309121	2.145752	-3.476201
H	4.117696	2.578715	-1.776311
C	5.933592	3.350795	-2.694868
H	6.437585	3.514025	-1.730362
H	6.703720	2.975170	-3.384094
C	5.427827	4.706974	-3.228155
H	4.902178	4.514736	-4.175978
C	4.529842	-1.307283	-2.866088
H	3.964413	-2.142535	-2.451485
H	5.567839	-1.635867	-2.996270
C	3.958830	-1.038404	-4.255206
F	4.027842	-2.173322	-4.997582
F	4.656127	-0.085195	-4.928257
F	2.670270	-0.644002	-4.234399
C	6.611913	5.638971	-3.530038
H	7.305560	5.185309	-4.247276
H	6.269155	6.591148	-3.950380
H	7.175684	5.863120	-2.615377
C	4.438135	5.386094	-2.267811
H	3.543931	4.778342	-2.097204
H	4.907905	5.572269	-1.293317
H	4.108656	6.351631	-2.667946

27

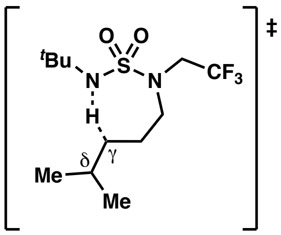

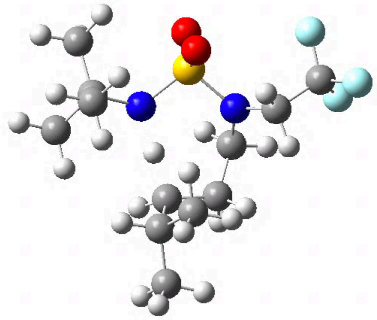

Atom	Coordinates (Angstroms) X	Y	Z
C	-0.297607	0.452434	0.000672
C	-0.266281	-0.583320	1.148745
H	0.633675	0.366632	-0.569364
H	-0.281375	1.447053	0.466853
H	0.584358	-0.383135	1.805419
H	-1.167580	-0.515907	1.761055
S	-1.515029	-2.950357	0.550328

O	-1.955160	-3.346759	1.895083
O	-1.163397	-4.031098	-0.390017
N	-2.641551	-1.835267	0.001847
C	-3.860958	-2.300172	-0.735449
C	-4.782234	-1.069286	-0.814526
H	-4.329183	-0.265060	-1.402079
H	-5.723070	-1.345681	-1.300760
H	-5.005296	-0.687131	0.185741
C	-3.546492	-2.817540	-2.153766
H	-2.883423	-3.684103	-2.122080
H	-4.477306	-3.108265	-2.652359
H	-3.072472	-2.037845	-2.757948
C	-4.566158	-3.402895	0.084978
H	-4.762597	-3.067256	1.105943
H	-5.519713	-3.638012	-0.398907
H	-3.974915	-4.320698	0.128731
H	-2.034556	-0.835092	-0.634379
N	-0.153456	-1.961861	0.648705
C	1.106540	-2.442773	0.119183
H	0.929634	-3.230629	-0.615077
C	2.038358	-3.018154	1.184799
F	1.506282	-4.074444	1.830656
F	3.197183	-3.431095	0.607017
F	2.374423	-2.099953	2.130440
H	1.646277	-1.628295	-0.373432
C	-1.315790	0.490027	-2.428832
H	-2.293152	0.299365	-2.893105
C	-0.934107	1.948606	-2.773833
H	-0.876338	2.078031	-3.860320
H	-1.673465	2.658054	-2.387211
H	0.042676	2.212811	-2.352979
C	-0.310754	-0.499332	-3.040507
H	0.710492	-0.311590	-2.690719
H	-0.566644	-1.536412	-2.801861
H	-0.299560	-0.399939	-4.130961
C	-1.504745	0.338901	-0.921239
H	-2.346237	0.939988	-0.559087

28

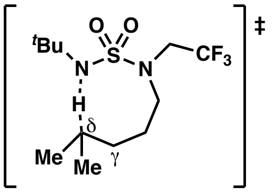

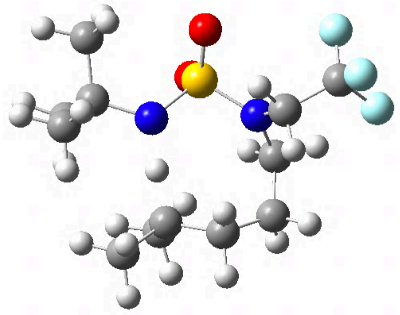

Atom	Coordinates (Angstroms) X	Y	Z
C	-1.475525	-0.392402	0.137640
H	-0.504352	-0.695839	0.541132
H	-2.221531	-0.736406	0.852138
S	-3.120629	-2.054357	-1.272732
O	-3.527771	-2.466263	0.080494
O	-2.824924	-3.078194	-2.283074
N	-4.216657	-0.969730	-1.981674
C	-5.638641	-1.440345	-2.206131
C	-6.189291	-0.538518	-3.326244
H	-5.605908	-0.669830	-4.242379
H	-7.230402	-0.805259	-3.536802
H	-6.161430	0.516510	-3.050268
C	-5.699740	-2.903805	-2.697522
H	-5.405122	-3.615939	-1.923697
H	-6.737549	-3.116940	-2.973544
H	-5.065161	-3.062567	-3.571032
C	-6.494254	-1.311715	-0.929441
H	-6.581052	-0.270516	-0.609545
H	-7.505128	-1.682859	-1.128106
H	-6.065659	-1.894479	-0.110530
N	-1.723002	-1.146390	-1.103938
C	-0.870510	-0.967154	-2.264683
H	-1.366558	-1.356536	-3.154249
C	0.471978	-1.690050	-2.155900
F	0.353778	-3.010486	-1.928953
F	1.167253	-1.530954	-3.313315
F	1.251090	-1.182609	-1.160299
H	-0.645209	0.091099	-2.433806
C	-1.512323	1.136928	-0.013913
H	-1.569924	1.553424	0.999201
H	-0.556789	1.490975	-0.419956
C	-2.631146	1.720047	-0.891827
H	-2.562136	2.816910	-0.815735
H	-2.433661	1.496041	-1.947937
C	-4.085201	1.340908	-0.596717
H	-4.207067	0.159631	-1.207657
C	-5.042932	2.248579	-1.359308
H	-6.084993	1.933384	-1.257244
H	-4.971517	3.267613	-0.951138
H	-4.794131	2.304424	-2.423589
C	-4.461832	1.198831	0.870039
H	-3.904949	0.415406	1.387591
H	-4.259240	2.147229	1.390542

H	-5.527251	0.984900	0.990089

29	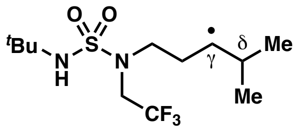	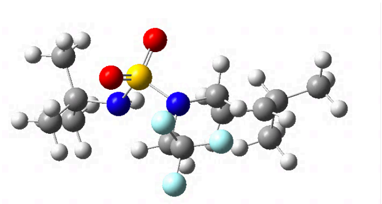

Atom	Coordinates (Angstroms) X	Y	Z
C	2.191326	-2.091351	-2.370091
S	0.356033	-1.207191	1.063491
O	-0.730625	-2.033854	1.589589
O	1.628062	-1.088104	1.766838
N	-0.340671	0.338771	0.866615
C	0.363866	1.452609	0.375611
O	-0.166391	2.547109	0.300336
O	1.611994	1.144534	0.023058
C	2.589996	2.209819	-0.361225
C	2.755638	3.192752	0.800216
H	3.566787	3.888372	0.562463
H	1.846407	3.769716	0.973407
H	3.023352	2.659731	1.717826
C	2.132541	2.886198	-1.655234
H	1.220786	3.464899	-1.503675
H	2.922119	3.561158	-2.001630
H	1.957637	2.139598	-2.435759
C	3.876964	1.414600	-0.584062
H	3.760376	0.705514	-1.408045
H	4.688509	2.101536	-0.841309
H	4.159944	0.870486	0.321714
C	-1.788496	0.517138	1.156983
H	-2.057153	-0.174465	1.953196
H	-1.888200	1.535737	1.533645
C	-2.686861	0.310928	-0.073757
H	-2.288625	0.936013	-0.891652
H	-2.615328	-0.730496	-0.412925
C	-5.435627	0.346879	-1.911236
H	-5.678926	1.412577	-1.995269
H	-4.520501	0.163165	-2.483927
H	-6.247033	-0.225516	-2.376685
C	-6.574279	0.165511	0.350953
H	-7.403694	-0.381098	-0.111297
H	-6.482919	-0.168923	1.389754
H	-6.840638	1.229737	0.361838
N	0.665091	-1.755999	-0.500830

H	-0.044758	-2.443476	-0.742681
C	-4.112977	0.658839	0.214292
H	-4.312133	1.604978	0.719164
C	-5.265273	-0.059358	-0.423963
H	-5.039851	-1.137837	-0.413104
C	2.026046	-2.142045	-0.859489
H	2.289392	-3.154836	-0.533664
H	2.728198	-1.439184	-0.412737
F	2.019975	-0.854988	-2.884016
F	3.433847	-2.507470	-2.714862
F	1.304007	-2.908979	-2.996482

30	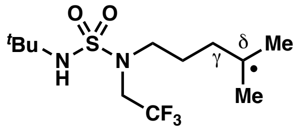	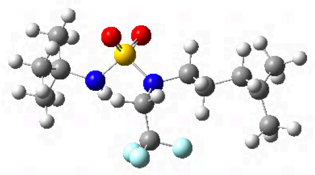

Atom	Coordinates (Angstroms) X	Y	Z
C	-0.083891	-0.278995	0.000068
C	-0.599742	-1.691471	0.328531
H	-0.860148	-1.787788	1.386194
H	-1.483729	-1.937254	-0.268029
H	0.179606	-2.427960	0.107111
C	1.135429	0.077511	0.864689
H	1.939417	-0.640849	0.674116
H	1.502169	1.078204	0.615244
H	0.891447	0.050326	1.927944
C	0.295440	-0.191901	-1.488616
H	-0.551031	-0.457186	-2.132778
H	0.633607	0.815839	-1.749702
H	1.108173	-0.892250	-1.701119
S	-2.158187	0.835529	1.505263
O	-3.337969	-0.032481	1.395470
O	-1.305860	0.704241	2.695688
C	-4.138637	2.657230	1.771287
H	-4.399155	1.944173	2.559750
H	-4.144269	3.653046	2.224558
C	-5.163760	2.589365	0.637547
H	-4.886975	3.308662	-0.141112
H	-5.135549	1.592001	0.187735
C	-7.635286	2.893114	0.058072
C	-7.742710	4.094452	-0.831614
H	-8.406502	4.864478	-0.394811
H	-6.772298	4.576049	-0.997115

H	-8.166097	3.840717	-1.811320
C	-8.791396	1.943550	0.125910
H	-9.243222	1.782451	-0.860592
H	-8.496097	0.968169	0.530084
H	-9.599385	2.323814	0.779851
N	-1.145890	0.780200	0.172467
H	-1.726416	0.882189	-0.658385
C	-6.594259	2.884600	1.145137
H	-6.582543	3.862590	1.659318
H	-6.857244	2.139273	1.907164
N	-2.742200	2.412279	1.307296
C	-1.756494	3.464790	1.534554
H	-1.914358	3.977769	2.490520
H	-0.750607	3.043133	1.542409
C	-1.774577	4.521046	0.435960
F	-0.814252	5.449828	0.678183
F	-1.539335	4.008279	-0.791939
F	-2.957472	5.184886	0.367530

31

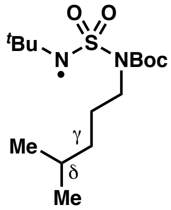

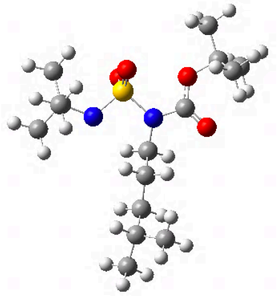

Atom	Coordinates (Angstroms) X	Y	Z
C	0.548287	-0.409099	-0.000149
C	0.611002	-1.564893	1.016869
H	0.935796	-2.500177	0.555593
H	1.287623	-1.328144	1.841587
H	-0.391281	-1.720611	1.427284
C	-0.379132	-0.789936	-1.190981
H	-1.383210	-0.974215	-0.795201
H	-0.433727	0.023704	-1.919047
H	-0.018707	-1.691926	-1.689582
C	0.015226	0.869906	0.679256
H	0.663426	1.165190	1.509602
H	-0.036017	1.696681	-0.034398
H	-0.988606	0.683489	1.073631
N	1.823383	-0.000896	-0.618049
S	3.056586	-1.128344	-0.780443
O	3.701100	-1.291449	0.530249
O	2.583273	-2.307347	-1.509015

N	4.075101	-0.204810	-1.814382
C	5.151940	-0.806096	-2.490873
O	5.879384	-0.152909	-3.219531
O	5.251962	-2.102763	-2.209840
C	6.238734	-2.990017	-2.885525
C	7.658178	-2.545720	-2.524316
H	8.371938	-3.279814	-2.912377
H	7.893886	-1.570382	-2.952351
H	7.777726	-2.497357	-1.437440
C	5.982340	-2.988659	-4.394213
H	6.203423	-2.016855	-4.838518
H	6.624472	-3.739785	-4.865616
H	4.940976	-3.251013	-4.605204
C	5.916394	-4.353859	-2.272952
H	4.883534	-4.640535	-2.488706
H	6.586097	-5.111491	-2.691016
H	6.050104	-4.330894	-1.187657
C	4.110809	1.280399	-1.694782
H	3.697856	1.551488	-0.722391
H	5.165822	1.563598	-1.703182
C	3.357261	1.986216	-2.822250
H	3.771907	1.650943	-3.778004
H	2.305461	1.676967	-2.791885
C	3.458071	3.512675	-2.692773
H	4.513391	3.817582	-2.758128
H	3.115836	3.805929	-1.689714
C	2.645874	4.309237	-3.734560
H	1.602624	3.962118	-3.677522
C	3.142565	4.078488	-5.170888
H	4.191747	4.384729	-5.272601
H	3.071183	3.027994	-5.469675
H	2.554106	4.665491	-5.885195
C	2.659863	5.807772	-3.393652
H	2.052373	6.381384	-4.102818
H	2.266969	5.993115	-2.387267
H	3.681404	6.207335	-3.432111

32	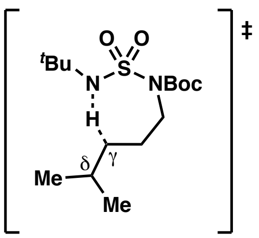	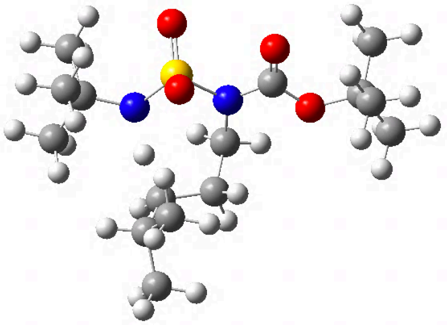

Atom Coordinates (Angstroms)

	X	Y	Z
C	-1.667078	0.751226	-0.282387
C	-1.669479	-0.425352	0.715184
H	-0.984704	0.508482	-1.102368
H	-1.243425	1.623185	0.237101
H	-0.746622	-0.427408	1.290852
H	-2.505436	-0.349817	1.412076
S	-3.174633	-2.355313	-0.714339
O	-3.571422	-3.621203	-0.090913
O	-2.943490	-2.329852	-2.163804
N	-4.206176	-1.152512	-0.182590
C	-5.681623	-1.278495	-0.445363
C	-6.266295	0.117625	-0.158109
H	-5.936368	0.853779	-0.896887
H	-7.358079	0.065917	-0.203197
H	-5.979782	0.464449	0.839313
C	-6.038255	-1.707305	-1.880317
H	-5.695442	-2.721627	-2.099279
H	-7.125920	-1.684368	-2.005134
H	-5.593018	-1.031941	-2.616650
C	-6.268751	-2.273921	0.581670
H	-6.005478	-1.972744	1.599742
H	-7.360119	-2.273322	0.488420
H	-5.900745	-3.286587	0.414381
H	-3.711116	-0.008411	-0.694744
H	-1.729626	-1.753455	0.063756
C	-3.216408	1.753872	-2.173668
H	-4.296054	1.805120	-2.373143
C	-2.699276	3.211998	-2.140143
H	-2.891462	3.701542	-3.101328
H	-3.193032	3.798003	-1.357236
H	-1.618788	3.243133	-1.959435
C	-2.566582	0.966484	-3.322507
H	-1.473086	0.978103	-3.252418
H	-2.888934	-0.078025	-3.331741
H	-2.833876	1.420081	-4.282981
C	-3.052815	1.116158	-0.797410
H	-3.627811	1.654317	-0.033611
C	-0.586875	-2.515037	-0.203625
O	-0.626504	-3.591619	-0.769792
O	0.518226	-1.901006	0.257079
C	1.857202	-2.543736	0.161535
C	2.237601	-2.732276	-1.309342
H	1.592982	-3.463163	-1.798630
H	2.170093	-1.781055	-1.847042

H	3.273337	-3.082659	-1.368743
C	1.862046	-3.857983	0.946613
H	1.527331	-3.690985	1.975486
H	1.219206	-4.605420	0.480493
H	2.884235	-4.248976	0.981761
C	2.761177	-1.505821	0.830298
H	3.796460	-1.859479	0.823024
H	2.718550	-0.551608	0.296430
H	2.460344	-1.339610	1.869170

33	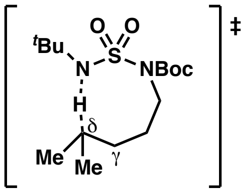	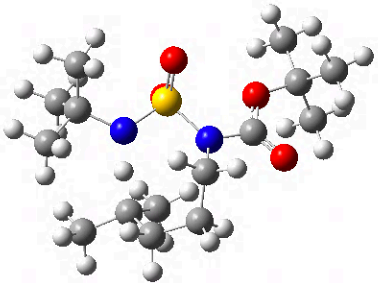

Atom	Coordinates (Angstroms) X	Y	Z
C	0.397665	4.696510	0.894806
C	1.818723	4.756122	0.329636
H	0.460800	4.430610	1.958511
H	0.004659	5.726757	0.870781
C	-0.324383	2.281253	0.199330
H	0.084510	1.956196	1.155124
H	-1.245773	1.727583	0.011042
C	-0.631948	3.785551	0.207737
H	-0.822524	4.111894	-0.819456
H	-1.582455	3.909892	0.742204
C	3.698248	2.012128	1.731287
C	3.464032	2.795362	3.036143
H	4.325171	2.663611	3.698704
H	3.345323	3.863970	2.854598
H	2.572013	2.426552	3.550956
C	4.955943	2.531244	1.007657
H	5.823024	2.437821	1.670075
H	5.156807	1.965197	0.095363
H	4.849500	3.584996	0.735380
C	3.887129	0.523608	2.106656
H	4.690932	0.463523	2.847897
H	2.975667	0.112473	2.547974
H	4.159442	-0.091730	1.249294
N	2.432949	2.204133	0.932571
H	2.243490	3.535433	0.608401

S	2.256064	1.370395	-0.511733
O	2.251778	-0.074018	-0.241693
O	3.130383	1.873478	-1.577183
N	0.628478	1.874150	-0.870673
C	2.695160	5.740171	1.094302
H	2.574831	5.657678	2.177773
H	3.755265	5.630117	0.846495
H	2.404478	6.763376	0.813675
C	1.941280	4.947277	-1.173821
H	2.987324	4.925439	-1.491997
H	1.398144	4.196012	-1.746239
H	1.532093	5.934158	-1.439749
C	0.045786	1.533544	-2.101055
O	-1.110557	1.834029	-2.358060
O	0.888009	0.869038	-2.889246
C	0.529112	0.462878	-4.273391
C	1.816831	-0.206715	-4.756777
H	1.682235	-0.564727	-5.782066
H	2.650661	0.500356	-4.738205
H	2.071398	-1.058325	-4.119657
C	0.207893	1.703263	-5.111024
H	0.075175	1.404059	-6.155920
H	-0.706916	2.191047	-4.771052
H	1.034768	2.418947	-5.065393
C	-0.628296	-0.538345	-4.230474
H	-1.549047	-0.070432	-3.879008
H	-0.798622	-0.932590	-5.237799
H	-0.380879	-1.378229	-3.573742

34

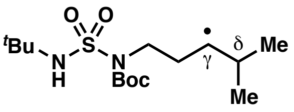

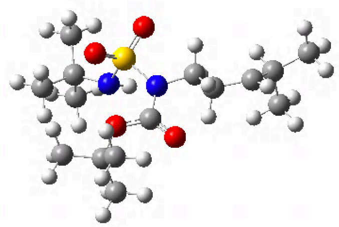

Atom	Coordinates (Angstroms) X	Y	Z
C	0.789932	0.074282	0.000101
C	0.498100	-1.216092	0.787602
H	0.694162	-1.089064	1.855758
H	-0.543560	-1.528524	0.665122
H	1.143638	-2.021617	0.422488
C	2.240887	0.529543	0.211375
H	2.920941	-0.264194	-0.114714
H	2.448275	1.427705	-0.376579

H	2.440906	0.750431	1.260975
C	0.533304	-0.158937	-1.499174
H	-0.491268	-0.504561	-1.681182
H	0.702426	0.757419	-2.073204
H	1.214624	-0.929008	-1.872210
S	-0.518294	1.564884	1.960261
O	-1.585755	0.700532	2.481366
O	0.726945	1.671121	2.713458
N	-1.280478	3.093910	1.777404
C	-0.620220	4.266702	1.366409
O	-1.231410	5.315233	1.234988
O	0.679811	4.074679	1.162535
C	1.584886	5.204789	0.811093
C	1.567207	6.247677	1.931935
H	2.334009	7.002071	1.727933
H	0.599943	6.746671	2.002432
H	1.799613	5.778405	2.892985
C	1.184582	5.786968	-0.546856
H	0.214467	6.283267	-0.500010
H	1.937346	6.519028	-0.857390
H	1.146745	4.997680	-1.304416
C	2.949570	4.519339	0.732027
H	2.957109	3.757632	-0.052133
H	3.718920	5.262194	0.500858
H	3.198245	4.041474	1.683528
C	-2.729092	3.207108	2.092440
H	-2.956320	2.495379	2.883975
H	-2.872212	4.216589	2.478992
C	-3.638403	2.972058	0.874741
H	-3.271014	3.609604	0.051741
H	-3.541283	1.931170	0.538034
C	-6.415168	2.906067	-0.923805
H	-6.693426	3.962895	-1.011379
H	-5.503196	2.747754	-1.509181
H	-7.214684	2.304873	-1.373355
C	-7.513373	2.705491	1.356309
H	-8.332012	2.129602	0.910623
H	-7.395760	2.381889	2.395849
H	-7.812944	3.760890	1.363868
N	-0.146967	1.205998	0.359707
H	-1.037258	1.108010	-0.127858
C	-5.071297	3.274947	1.179622
H	-5.293166	4.216077	1.684269
C	-6.209496	2.516259	0.563389
H	-5.950132	1.445464	0.578379

35	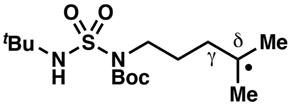	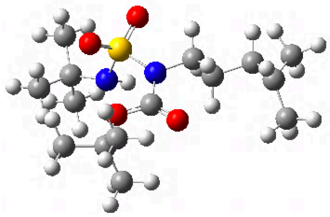

Atom	Coordinates (Angstroms)		
	X	Y	Z
C	0.956946	-0.037207	0.000126
C	0.755948	-1.280022	0.886193
H	1.033560	-1.083350	1.925338
H	-0.285456	-1.615508	0.866948
H	1.386704	-2.096728	0.519999
C	2.410241	0.453730	0.064709
H	3.078448	-0.346761	-0.269269
H	2.550241	1.318369	-0.589678
H	2.690057	0.740214	1.079672
C	0.586101	-0.368487	-1.456181
H	-0.442522	-0.740561	-1.532413
H	0.689721	0.512462	-2.097254
H	1.251062	-1.149965	-1.835033
S	-0.219612	1.557736	1.962771
O	-1.220592	0.711093	2.625914
O	1.080025	1.736381	2.601830
N	-1.027794	3.058722	1.747709
C	-0.424760	4.216983	1.224332
O	-1.061632	5.250189	1.093223
O	0.856057	4.029988	0.918823
C	1.713766	5.152736	0.447532
C	1.775332	6.240548	1.523202
H	2.511928	6.994174	1.226192
H	0.810317	6.731747	1.654276
H	2.093120	5.813407	2.479457
C	1.194965	5.674405	-0.894987
H	0.227382	6.166154	-0.787497
H	1.911749	6.397019	-1.298819
H	1.101013	4.853144	-1.612639
C	3.075694	4.476992	0.282440
H	3.028437	3.686566	-0.471426
H	3.815258	5.217158	-0.037515
H	3.405940	4.036588	1.227136
C	-2.460496	3.157112	2.134222
H	-2.623702	2.477303	2.969723
H	-2.603928	4.179460	2.486957

C	-3.420710	2.849462	0.982368
H	-3.184984	3.502786	0.135039
H	-3.281826	1.813199	0.649486
C	-5.884089	2.816366	0.291278
C	-6.039343	3.866620	-0.767131
H	-6.779009	4.633524	-0.469190
H	-5.102219	4.398036	-0.966681
H	-6.397723	3.442677	-1.713456
C	-6.983664	1.814914	0.469167
H	-7.386285	1.478195	-0.494112
H	-6.648150	0.932981	1.027346
H	-7.837680	2.237503	1.032232
N	0.027659	1.099307	0.363770
H	-0.897423	0.954214	-0.040387
C	-4.895343	3.045974	1.402835
H	-5.002127	4.073707	1.793487
H	-5.114535	2.373076	2.241929

36	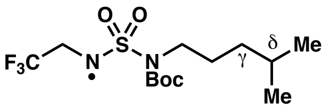	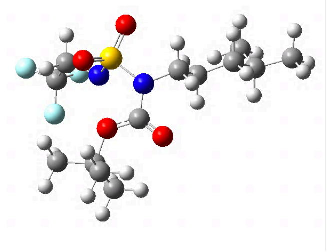

Atom	Coordinates (Angstroms) X	Y	Z
C	-2.537839	1.163760	-0.953438
N	-0.119220	1.305893	-1.058932
S	1.392378	0.970846	-0.340390
O	1.439932	-0.410998	0.131478
O	1.628538	2.070554	0.599538
N	2.452083	1.209080	-1.631539
C	3.361065	2.389507	-1.619646
H	3.622067	2.591845	-0.580797
H	4.262174	2.061640	-2.140552
C	2.757746	3.626508	-2.288334
H	2.477806	3.375290	-3.318882
H	1.839983	3.904917	-1.759890
C	3.752377	4.796443	-2.293115
H	4.001508	5.070037	-1.256738
H	4.690237	4.459265	-2.756617
C	3.267813	6.057236	-3.038192
H	2.992267	5.752706	-4.059380
C	4.405923	7.084256	-3.146639

H	5.281089	6.659817	-3.651813
H	4.088415	7.968645	-3.710388
H	4.724500	7.420999	-2.151727
C	2.029984	6.690562	-2.382726
H	1.175088	6.007242	-2.364885
H	2.246176	6.984934	-1.347499
H	1.720050	7.590400	-2.925951
C	-1.225436	0.860875	-0.243571
H	-1.234936	1.374865	0.729356
H	-1.172180	-0.217601	-0.042117
C	1.233906	-1.515743	-3.799708
C	0.026687	-2.291916	-3.272989
H	-0.842596	-1.635934	-3.171260
H	-0.226141	-3.093137	-3.973621
H	0.248312	-2.738757	-2.299662
C	2.471487	-2.411151	-3.884243
H	3.339386	-1.863943	-4.255120
H	2.706175	-2.833937	-2.902549
H	2.264749	-3.238943	-4.570104
C	0.908911	-0.818126	-5.121700
H	0.061214	-0.137359	-4.997187
H	1.764600	-0.257966	-5.501077
H	0.631117	-1.573996	-5.863377
C	2.430452	0.396575	-2.785467
O	3.239187	0.551380	-3.680264
O	1.438537	-0.488365	-2.732233
F	-3.577499	0.749696	-0.188861
F	-2.701690	2.483381	-1.185163
F	-2.629331	0.527293	-2.142892

37

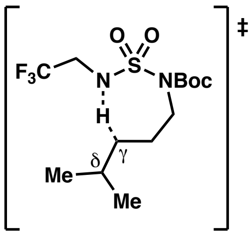

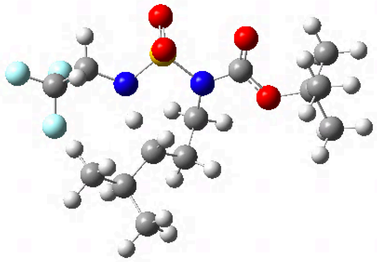

Atom	Coordinates (Angstroms) X	Y	Z
C	-2.147133	1.042188	-0.001262
C	-1.582397	-0.351611	0.341257
H	-1.321812	1.686407	-0.331525
H	-2.539536	1.469244	0.929642
H	-0.652127	-0.253848	0.898528
H	-2.287243	-0.908959	0.956617
S	-2.481702	-2.154887	-1.605210

O	-2.363132	-3.554570	-1.189306
O	-2.522960	-1.843967	-3.036394
N	-1.275498	-1.175807	-0.851578
C	0.010775	-1.302242	-1.394519
O	0.287755	-2.121267	-2.249613
O	0.837570	-0.405198	-0.838388
C	2.287547	-0.364306	-1.189187
C	2.945754	-1.696474	-0.822847
H	2.579596	-2.510857	-1.449064
H	4.028110	-1.609363	-0.962602
H	2.758656	-1.941336	0.227570
C	2.447041	-0.011160	-2.669701
H	3.508887	0.143236	-2.887241
H	2.071683	-0.807039	-3.313870
H	1.915174	0.917019	-2.901876
C	2.803004	0.766153	-0.296972
H	3.875093	0.906014	-0.463886
H	2.292772	1.706441	-0.526469
H	2.646213	0.530983	0.760037
N	-3.803547	-1.504685	-0.778295
C	-6.118815	-1.938738	-0.168849
C	-4.350092	2.060841	-0.970262
H	-4.838452	1.935494	0.006399
C	-5.402191	1.881268	-2.074809
H	-4.948966	1.988280	-3.067470
H	-5.881070	0.899236	-2.025159
H	-6.190116	2.635878	-1.982789
C	-3.764640	3.493218	-1.018816
H	-4.572692	4.227627	-0.932266
H	-3.056384	3.675163	-0.205128
H	-3.247677	3.670245	-1.969327
C	-3.228704	1.038327	-1.076074
H	-3.712944	-0.162479	-0.962047
H	-2.817270	0.967905	-2.090011
C	-5.073573	-2.037145	-1.269876
H	-4.976870	-3.097981	-1.525366
H	-5.453488	-1.512291	-2.153966
F	-6.291810	-0.661774	0.258925
F	-7.318376	-2.372708	-0.628738
F	-5.805823	-2.681339	0.913239

38	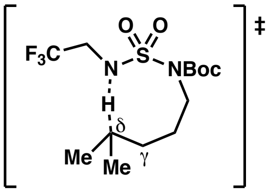	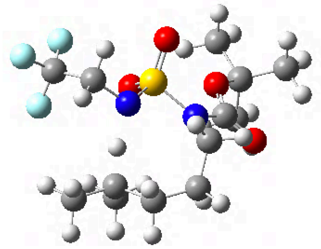

Atom	Coordinates (Angstroms) X	Y	Z
C	-5.492609	2.026106	0.000239
C	-4.185078	2.175925	-0.785543
H	-5.243438	1.808580	1.047503
H	-5.969619	3.019561	0.008387
C	-6.163516	-0.480318	-0.411855
H	-5.579532	-0.699601	0.481181
H	-7.076868	-1.076148	-0.377782
C	-6.540184	1.009234	-0.484998
H	-6.875793	1.238938	-1.500943
H	-7.423782	1.133944	0.153770
N	-3.343478	-0.312568	-0.260657
H	-3.671738	1.066722	-0.643577
S	-3.714213	-1.326237	-1.541500
O	-3.471833	-2.696681	-1.068402
O	-3.129896	-0.891671	-2.808844
N	-5.404876	-0.978987	-1.595245
C	-2.032575	-0.438872	0.354717
H	-2.015400	0.248505	1.204574
H	-1.919583	-1.456800	0.747303
C	-0.778555	-0.139689	-0.474024
F	-0.495881	-1.094551	-1.381850
F	0.289367	-0.056957	0.364710
F	-0.860188	1.039459	-1.133974
C	-6.184211	-1.472800	-2.658098
O	-7.372269	-1.207551	-2.742601
O	-5.473434	-2.239239	-3.479674
C	-6.036530	-2.788645	-4.745159
C	-6.484290	-1.638802	-5.650223
H	-6.774258	-2.044870	-6.624753
H	-7.338821	-1.105719	-5.230209
H	-5.662087	-0.933850	-5.807293
C	-7.170814	-3.762648	-4.417873
H	-8.029509	-3.246501	-3.986197
H	-7.489183	-4.262382	-5.338675
H	-6.824974	-4.529348	-3.717539

C	-4.834650	-3.522954	-5.340815
H	-4.484486	-4.305408	-4.661680
H	-5.122707	-3.987104	-6.288904
H	-4.009474	-2.830002	-5.525663
C	-4.330436	2.406934	-2.282458
H	-4.827884	3.373354	-2.452538
H	-3.353941	2.443232	-2.773456
H	-4.927416	1.634123	-2.770618
C	-3.224161	3.154702	-0.119933
H	-3.647532	4.168560	-0.165069
H	-3.063657	2.916646	0.937048
H	-2.255351	3.178261	-0.625297

39

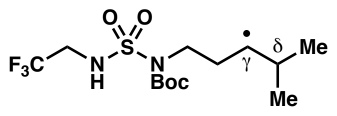

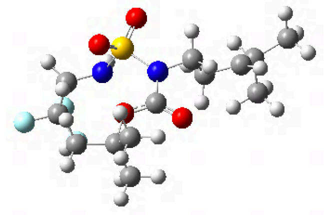

Atom	Coordinates (Angstroms) X	Y	Z
C	1.143479	-0.911028	0.000228
S	-0.692169	-0.026600	3.433551
O	-1.778846	-0.853247	3.959642
O	0.579793	0.092617	4.137000
N	-1.388917	1.519276	3.236376
C	-0.684316	2.633169	2.745560
O	-1.214540	3.727689	2.670369
O	0.563821	2.325107	2.393052
C	1.541805	3.390415	2.008794
C	1.707367	4.373384	3.170221
H	2.518553	5.068974	2.932513
H	0.798133	4.950369	3.343328
H	1.974997	3.840380	4.087865
C	1.084350	4.066733	0.714756
H	0.172517	4.645328	0.866260
H	1.873865	4.741791	0.368408
H	0.909585	3.320111	-0.065781
C	2.828812	2.595240	1.786030
H	2.712278	1.886082	0.962103
H	3.640320	3.282202	1.528738
H	3.111817	2.051212	2.691850
C	-2.836717	1.697693	3.526884
H	-3.105346	1.006102	4.323117
H	-2.936349	2.716302	3.903532
C	-3.735145	1.491491	2.296191

H	-3.336943	2.116591	1.478286
H	-3.663607	0.450074	1.957009
C	-6.484312	1.528227	0.458917
H	-6.727763	2.593935	0.375445
H	-5.569290	1.344900	-0.114065
H	-7.295752	0.955955	-0.006624
C	-7.622449	1.345625	2.721263
H	-8.451887	0.799127	2.258920
H	-7.530826	1.010703	3.759882
H	-7.888949	2.409810	2.732717
N	-0.382948	-0.575595	1.869315
H	-1.092779	-1.263104	1.627507
C	-5.161251	1.839390	2.584293
H	-5.360391	2.785540	3.089158
C	-6.313573	1.121305	1.945960
H	-6.087987	0.042859	1.956271
C	0.978035	-0.961664	1.510818
H	1.241329	-1.974444	1.836706
H	1.680151	-0.258791	1.957611
F	0.972261	0.325292	-0.513769
F	2.386061	-1.327217	-0.344355
F	0.256238	-1.728674	-0.626242

40

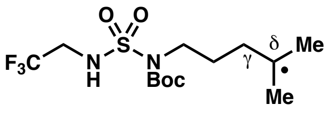

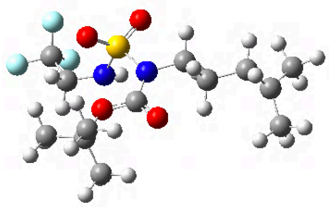

Atom	Coordinates (Angstroms) X	Y	Z
C	-5.476426	1.838953	0.001943
S	-7.425896	4.368807	1.609668
O	-8.507557	3.555986	2.163678
O	-6.177358	4.567403	2.335883
N	-8.164137	5.880087	1.287908
C	-7.490427	6.961382	0.697841
O	-8.042298	8.035903	0.532543
O	-6.238499	6.648340	0.358282
C	-5.295484	7.681878	-0.162325
C	-5.113173	8.782704	0.885346
H	-4.323456	9.463924	0.552545
H	-6.028796	9.358303	1.026916
H	-4.808449	8.351424	1.843874
C	-5.801344	8.215821	-1.504538

H	-6.720282	8.790872	-1.386561
H	-5.035416	8.865069	-1.941174
H	-5.982979	7.391111	-2.201177
C	-4.002173	6.886037	-0.343391
H	-4.136521	6.083728	-1.075064
H	-3.212642	7.549778	-0.707636
H	-3.677721	6.450228	0.605796
C	-9.592820	6.075455	1.652943
H	-9.800106	5.452743	2.522748
H	-9.680833	7.121835	1.949691
C	-10.557227	5.756029	0.508159
H	-10.289470	6.360370	-0.365807
H	-10.456132	4.702004	0.223971
C	-13.012311	5.790969	-0.202605
C	-13.149310	6.817678	-1.286015
H	-13.884697	7.597959	-1.013368
H	-12.205220	7.335902	-1.488477
H	-13.502232	6.375182	-2.225876
C	-14.113602	4.793117	-0.016503
H	-14.506613	4.437848	-0.977066
H	-13.783414	3.921873	0.561326
H	-14.973149	5.225735	0.530176
N	-7.036472	3.780533	0.090390
H	-7.806871	3.261712	-0.321133
C	-12.025112	6.033272	0.907588
H	-12.093194	7.081229	1.248856
H	-12.277146	5.408721	1.774530
F	-6.357522	1.093146	-0.718685
F	-4.230168	1.481612	-0.392266
F	-5.618905	1.478632	1.293627
C	-5.695036	3.330628	-0.230269
H	-4.977511	3.883129	0.376170
H	-5.488881	3.529406	-1.286094

XVI. References.

[1] Mintz, M.J.; Walling, C. *Org. Synth.* **1969**, *49*, 9.
[2] Johnson, R. C. J. Chem. Ed. 1970, 47, 702.
[3] Kanegusuku, A. L. G.; Castanheiro, T.; Ayer, S. K.; Roizen, J. L. *unpublished results.*
[4] Zhao, R.; Lu, W. *Org. Lett.* **2017**, *19*, 1768–1771.
[5] Govindarajan, S.R.; Xu, Y.; Swanson, J.P.; Jain, T.; Lu, Y.; Choi, J.-W.; Joy, A. *Macromolecules*. **2016**, *49*, 2429.
[6] a) Quinn, R.K.; Könst, Z.A.; Michalak, S.E.; Schmidt, Y.; Szklarski, A.R.; Flores, A.R.; Nam, S.; Horne, D.A.; Vanderwal, C.D.; Alexanian, E.J. *J. Am. Chem. Soc.* **2016**, *138*, 696.

b) Characterization data: Short, M. A.; Blackburn, J. M.; Roizen, J. L. *Angew. Chem. Int. Ed.* **2018**, *57*, 296.

[7] Mustafa, D.; Ma, D.; Zhou, W.; Meisenheimer, P.; Cali, J. J. *Bioconjugate Chem.* **2016**, *27*, 87–101.

[8] McCune, C. D.; Beio, M. L.; Sturdivant, J. M.; Salud-Bea, R. de la; Darnell, B. M.; Berkowitz, D. B. *J. Am. Chem. Soc.* **2017**, *139*, 14077–14089.

[9] Carocci, A.; Catalano, A.; Corbo, F.; Duranti, A.; Amoroso, R.; Franchini, C.; Lentini, G.; Tortorella, V. *Tetrahedron: Asymmetry*, **2000**, *11*, 3619.

[10] a) Koppenhoefer, B.; Schurig, V. *Org. Synth.* **1988**, *66*, 151; b) Koppenhoefer, B.; Schurig, V. *Org. Synth.* **1988**, *66*, 160.

[11] Blackburn, J. M.; Short, M. A.; Castanheiro, T.; Ayer, S. K.; Muellers, T. D.; Roizen, J. L. *Org. Lett.* **2017**, *19*, 6012.

[12] Skvorcova, M.; Jirgensons, A. *Org. Lett.* **2017**, *10*, 2478.

[13] Thottumkara, A. P.; Bowsher, M. S.; Vinod, T. K. *Org. Lett.* **2005**, *7*, 2933.

[14] Wong, M.-K.; Chung, N.-W.; He, L.; Yang, D. *J. Am. Chem. Soc.* **2003**, *125*, 158.

[15] Mukherjee, S.; Maji, B.; Tlahuext-Aca, A.; Glorius, F. *J. Am. Chem. Soc.* **2016**, *138*, 16200.

[16] Suthagar, K.; Watson, A. J. A.; Wilkinson, B. L.; Fairbanks, A. J. *Eur. J. Med. Chem.* **2015**, *102*, 153.

[17] Masui, T.; Kabaki, M.; Watanabe, H.; Kobayashi, T. Masui, Y. *Org. Process Res. Dev.* **2004**, *8*, 408.

[18] Kurokawa, T.; Kim, M.; Du Bois, J. *Angew. Chem. Int. Ed.* **2009**, *48*, 2777.

[19] Shehata, M. F.; Short, M. A.; Sanders, M. A.; Roizen, J. L.; *Tetrahedron,* **2019**, *75*, 3186.

[20] Cismesia, M. A., Yoon, T. P. *Chem. Sci.* **2015**, *6,* 5426.

[21] Hatchard, C.G.; Parker, C.A. *Proc. R. Soc. Lond. A*. **1956**, *235*, 518.

[22] R. G. Parr, W. Yang, *Density-Functional Theory of Atoms and Molecules* 1989, Oxford University Press, Oxford U.K.

[23] M. J. Frisch, G. W. Trucks, H. B. Schlegel, G. E. Scuseria, M. A. Robb, J. R. Cheeseman, G. Scalmani, V. Baron, E, B. Mennucci, G. A. Petersson, H. Nakatsuji, M. Caricato, X. Li, H. P. Hratchian, A. F. Izmaylov, J. Bloino, G. Zheng, J. L. Sonnenberg, M. Hada, M. Ehara, K. Toyota, R. Fukuda, J. Hasegawa, M. Ishida, T. Nakajima, Y. Honda, O. Kitao, H. Nakai, T. Vreven, J. A. Montgomery Jr., J. E. Peralta, F. Ogliaor, M. Bearpark, J. J. Heyd, E. Brothers, K. N. Kudin, V. N. Staroverov, T. Keith, R. Kobayashi, J. Normand, K. Raghavachari, A. Rendell, J. C. Burant, S. S. Iyengar, J. Tomasi, M. CossI, N. Rega, J. M. Millam, M. Klene, J. E. Knox, J. B. Cross, V. Bakken, C. Adamo, J. Jaramillo, R. Gomperts, R. E. Stratmann, O. Yazyev, A. J. Austin, R. Cammi, C. Pomelli, J. W. Ochterski, R. L. Martin, K. Morokuma, V. G. Zakrzewski, G. A. Voth, P. Salvador, J. J. Dannenberg, S. Dapprich, A. D. Daniels, O. Farkas, J. B. Foresman, J. V. Ortiz, J. Cioslowski, D. J. Fox, Gaussian 09, revision D.01; Gaussian, Inc. Wallingford, CT, 2013.

[24] M. J. Frisch, G. W. Trucks, H. B. Schlegel, G. E. Scuseria, M. A. Robb, J. R. Cheeseman, G. Scalmani, V. Barone, G. A. Petersson, H. Nakatsuji, X. Li, M. Caricato, A. V. Marenich, J. Bloino, B. G. Janesko, R. Gomperts, B. Mennucci, H. P. Hratchian, J. V. Ortiz, A. F. Izmaylov, J. L. Sonnenberg, D. Williams-Young, F. Ding, F. Lipparini, F. Egidi, J. Goings, B. Peng, A. Petrone, T. Henderson, D. Ranasinghe, V. G. Zakrzewski, J. Gao, N. Rega, G. Zheng, W. Liang, M. Hada, M. Ehara, K. Toyota, R. Fukuda, J. Hasegawa, M. Ishida, T. Nakajima, Y. Honda, O.

Kitao, H. Nakai, T. Vreven, K. Throssell, J. A. Montgomery, Jr., J. E. Peralta, F. Ogliaro, M. J. Bearpark, J. J. Heyd, E. N. Brothers, K. N. Kudin, V. N. Staroverov, T. A. Keith, R. Kobayashi, J. Normand, K. Raghavachari, A. P. Rendell, J. C. Burant, S. S. Iyengar, J. Tomasi, M. Cossi, J. M. Millam, M. Klene, C. Adamo, R. Cammi, J. W. Ochterski, R. L. Martin, K. Morokuma, O. Farkas, J. B. Foresman, and D. J. Fox, Gaussian 16, revision B.01, Gaussian, Inc., Wallingford CT, 2016.

[25] R. Denningtom, T. Keith, J. Millam, GaussView 2009, version 5; Semichem Inc.: Shawnee Mission, KS, 2009.

[26] a) Stephens, P. J.; Devlin, F. J.; Chabalowski, C. F.; Frisch, M. J. *J. Chem. Phys*. **1994**, *98*, 11623; b) Becke, A. D. *J. Chem. Phys*. **1993**, *98*, 1372; c) Becke, A. D. *J. Phys. Chem.* **1993**, *98*, 5648; d) Lee, C.; Yang, W.; Parr, R. G. *Phys. Rev. B* **1988**, *37*, 785.

[27] Svejstrup, T. D.; Zawodny, W.; Douglas, J. J.; Bidgeli, D.; Sheikh, N. S.; Leonori, D. *Chem. Commun*. **2016**, *52*, 12302.

[28] Morcillo, S. P.; Dauncey, E. M.; Kim, J. H.; Douglas, J. J.; Sheikh, N. S.; Leonori, D. *Angew. Chem. Int. Ed.* **2018**, *57*, 12945.

[29] Bootsma, A. N.; Wheeler, S. E. "Popular Integration Grids Can Result in Large Errors in DFT-Computed Free Energies," *ChemRxiv*. *Pre-print.* DOI:10.26434/chemrxiv.8864204.v5.

[30] Grimme, S. *Chem. Eur. J.* **2012**, *18*, 999955.

[31] Ribeiro, R. F.; Marenich, A. V.; Cramer, C. J.; Truhlar, D. G. *J. Phys. Chem. B*, **2011**, *115*, 14556.

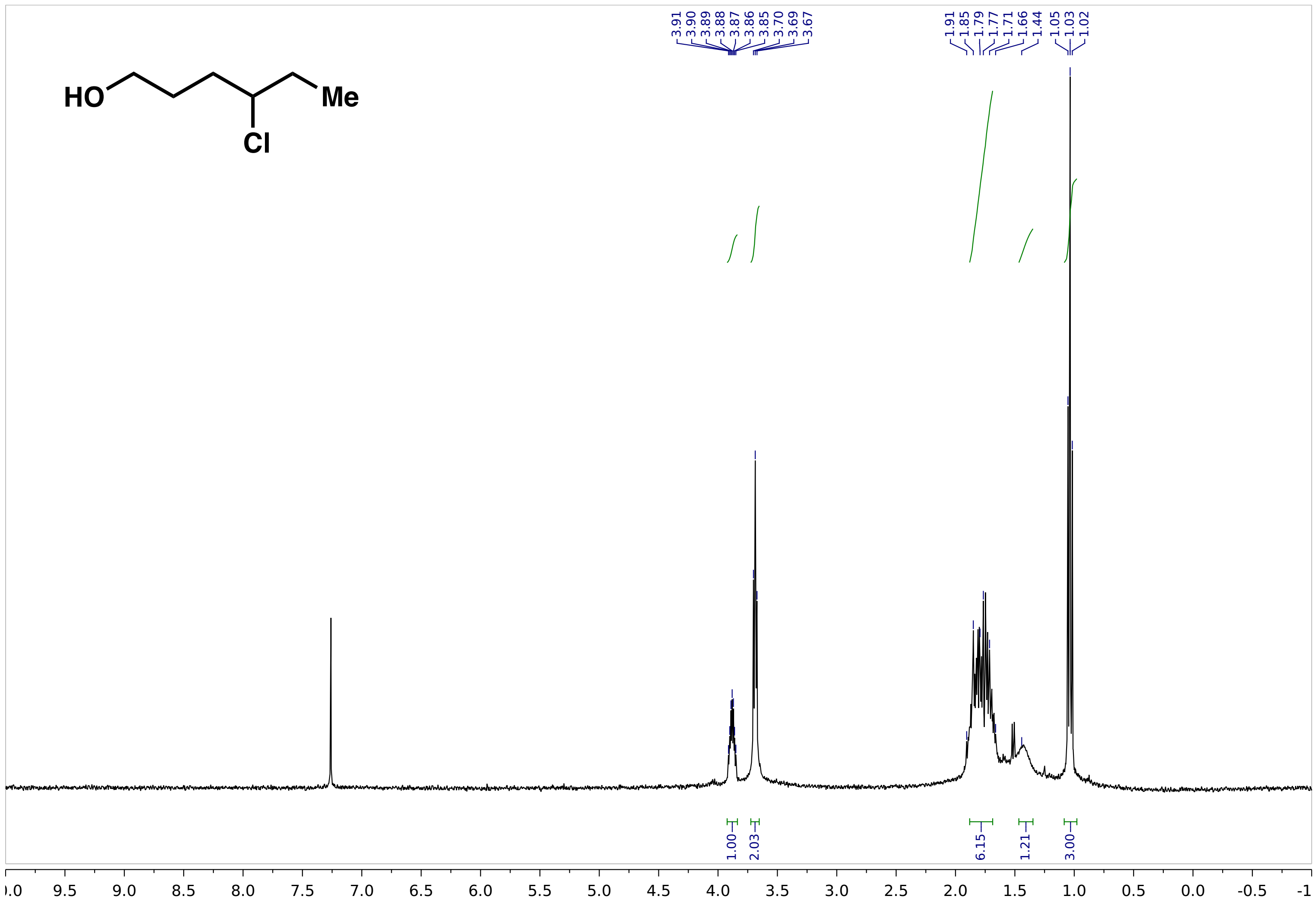

^{1}H NMR (400 MHz, $CDCl_3$) for 4-chlorohexanol (**S1**)

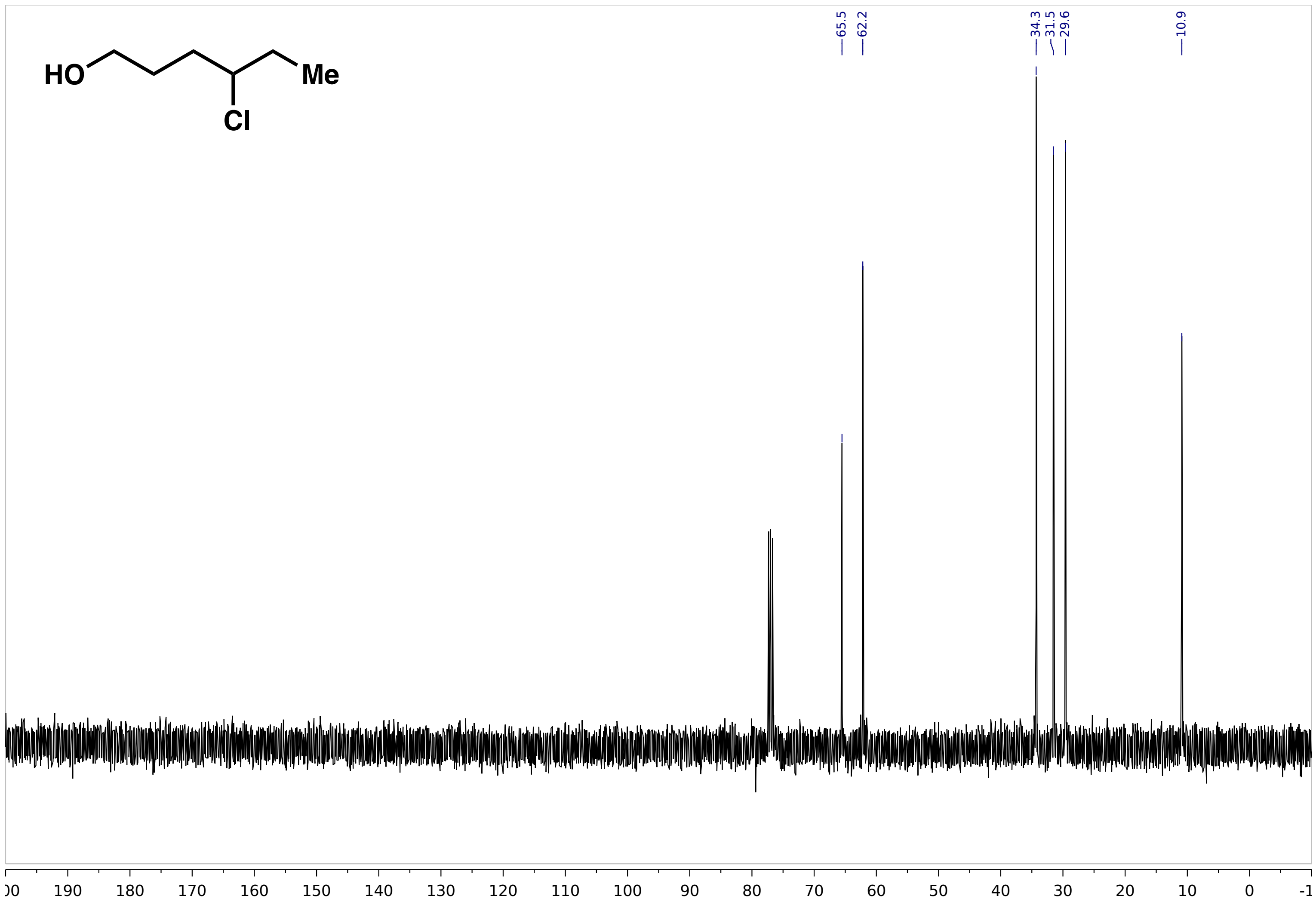

^{13}C NMR (101 MHz, $CDCl_3$) for 4-chlorohexanol (**S1**)

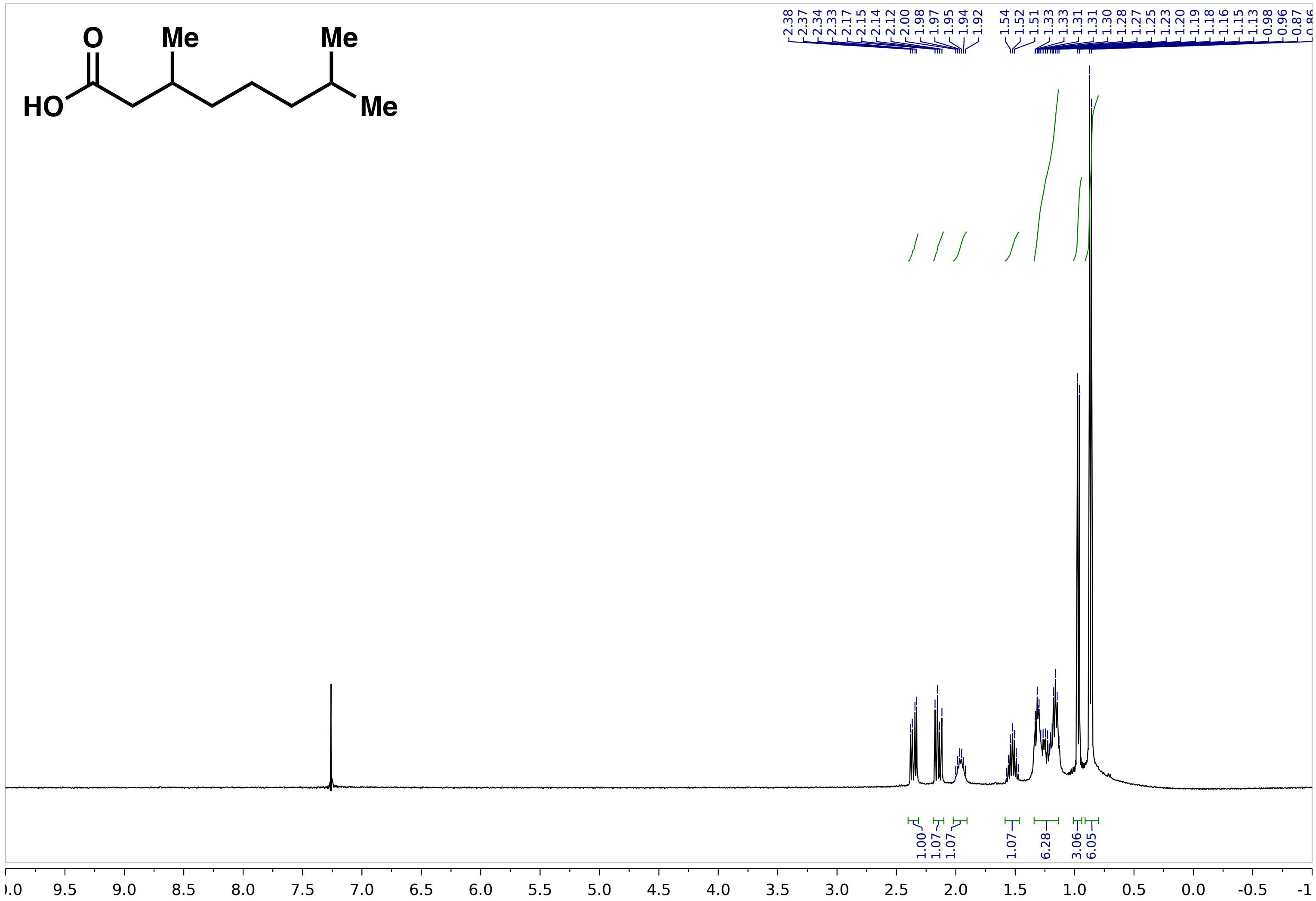

^{1}H NMR (400 MHz, $CDCl_3$) for 3,7-dimethyloctanoic acid (**S2h**)

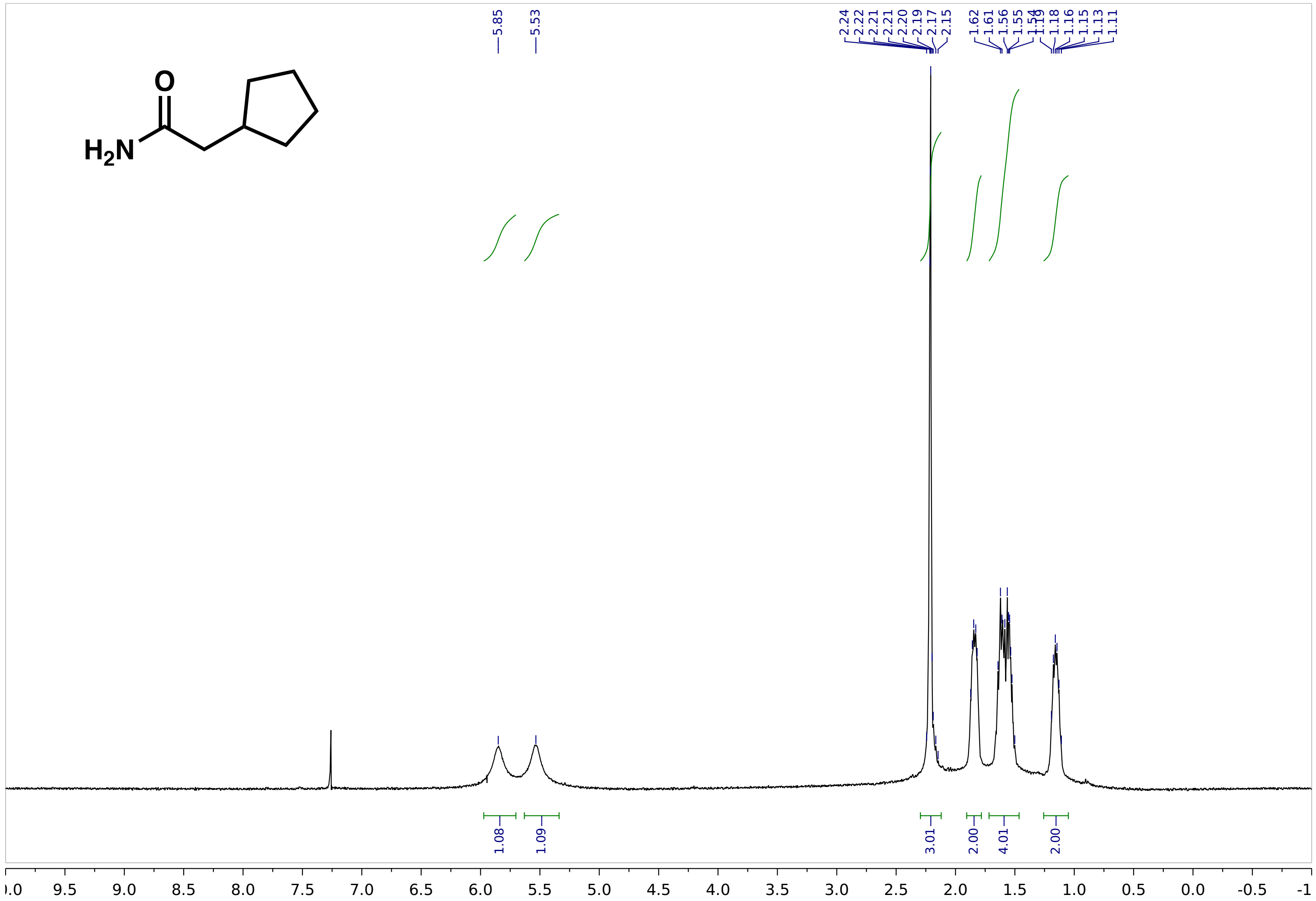

^{1}H NMR (400 MHz, $CDCl_3$) for 2-cyclopentylacetamide (**S3d**)

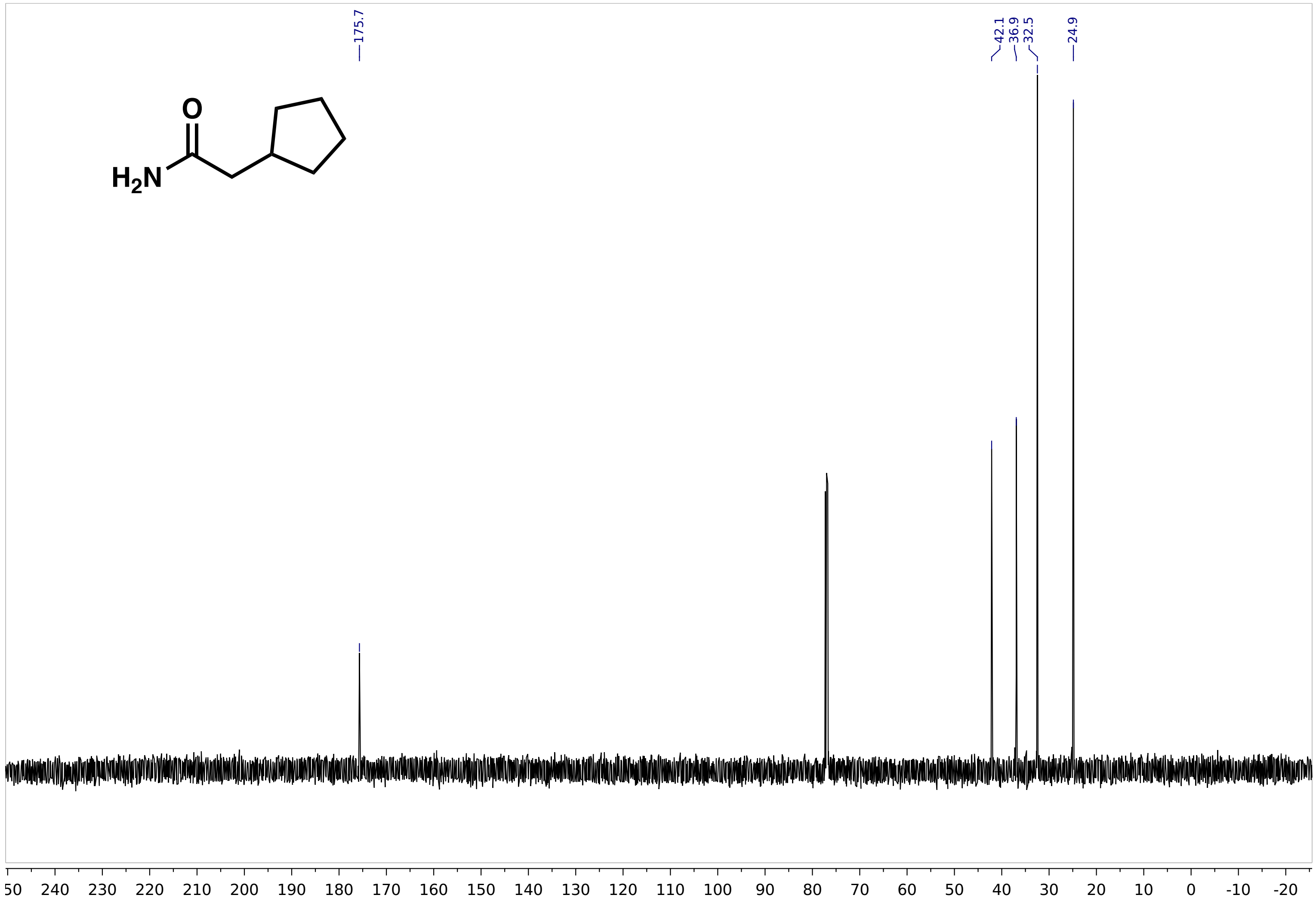

^{13}C NMR (126 MHz, $CDCl_3$) for 2-cyclopentylacetamide (**S3d**)

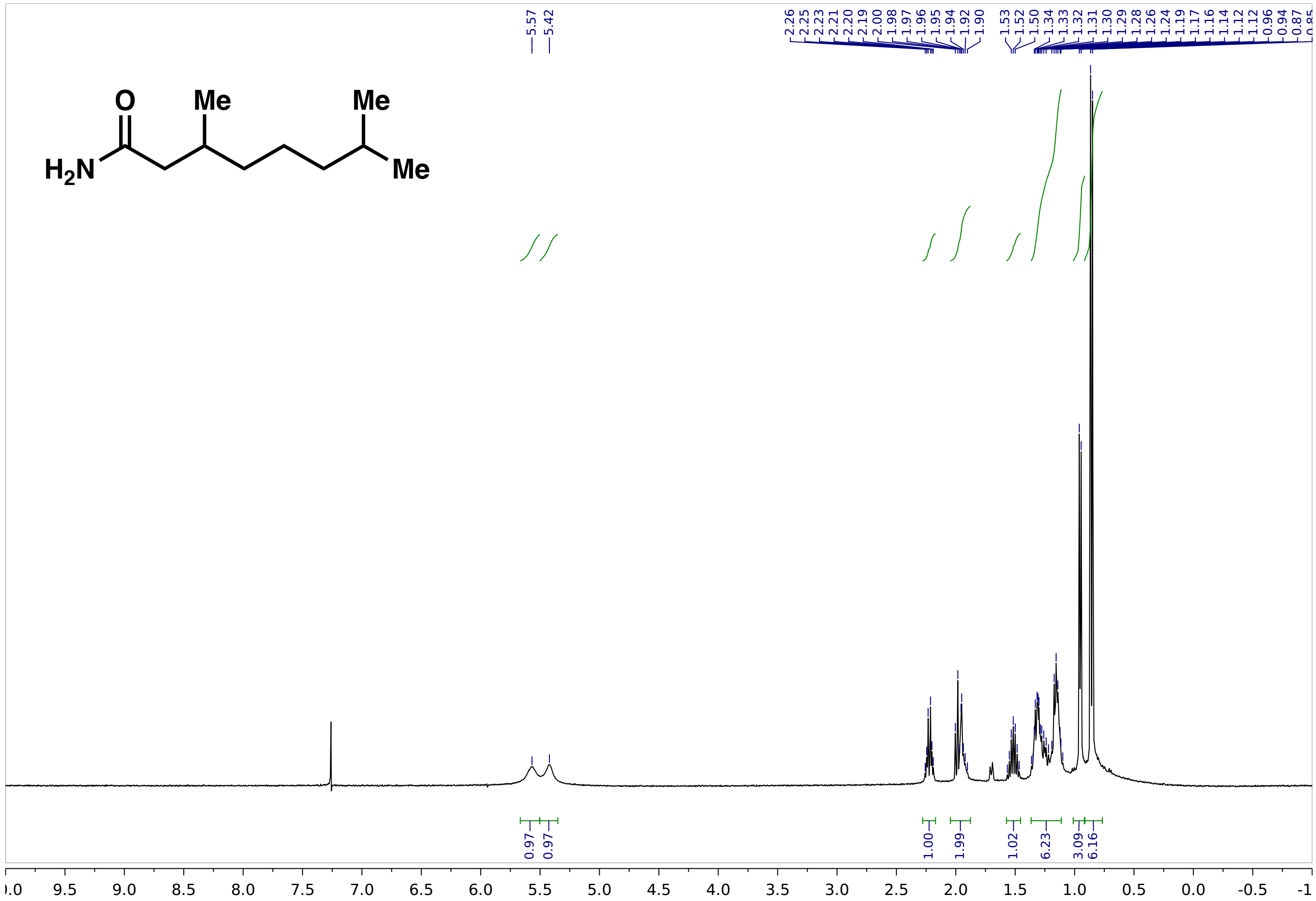

^{1}H NMR (400 MHz, $CDCl_3$) for 3,7-dimethyloctylamide (**S3h**)

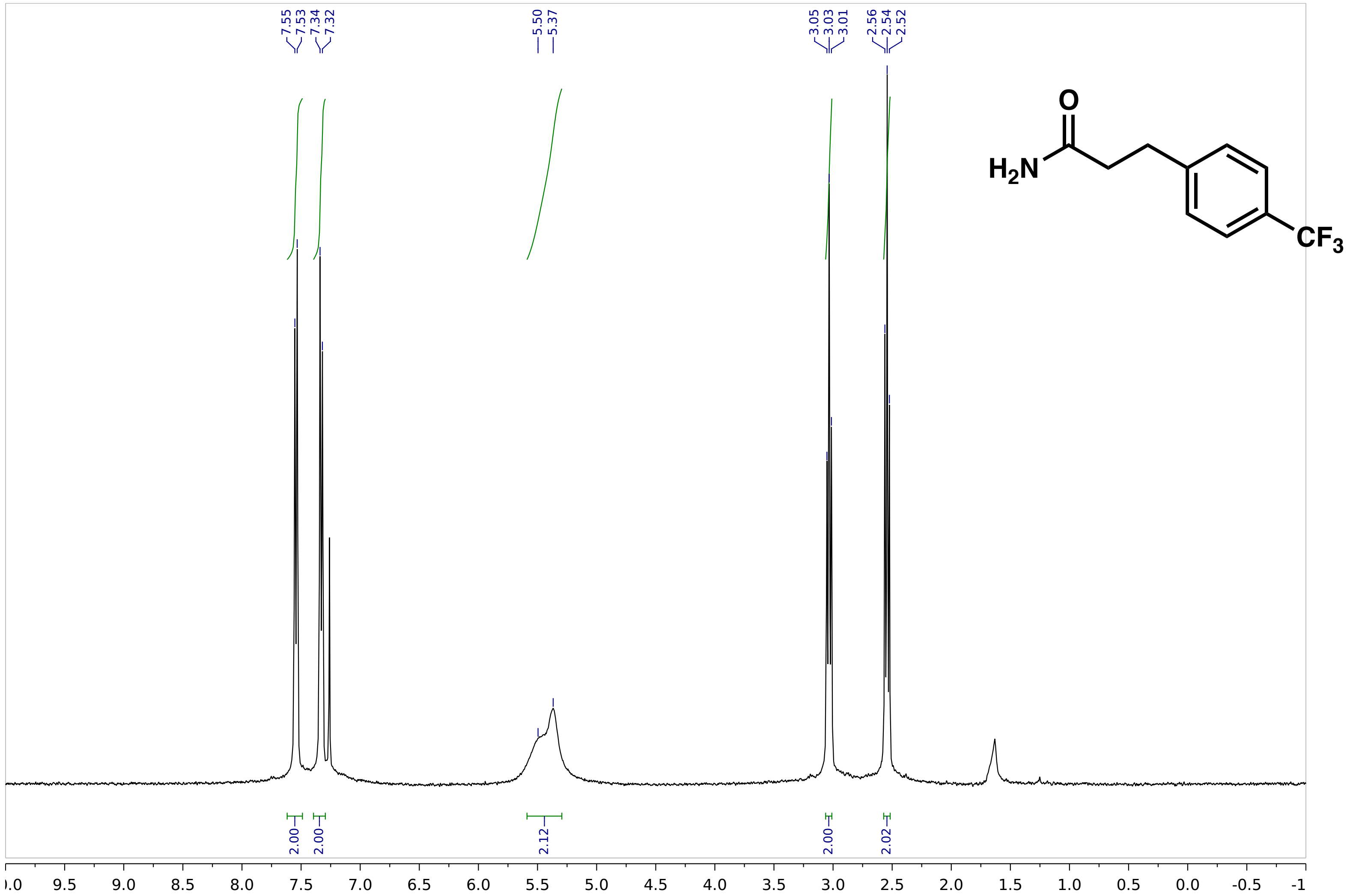

^{1}H NMR (400 MHz, $CDCl_3$) for 3-(4-trifluoromethylphenyl)propamide (**S3t**)

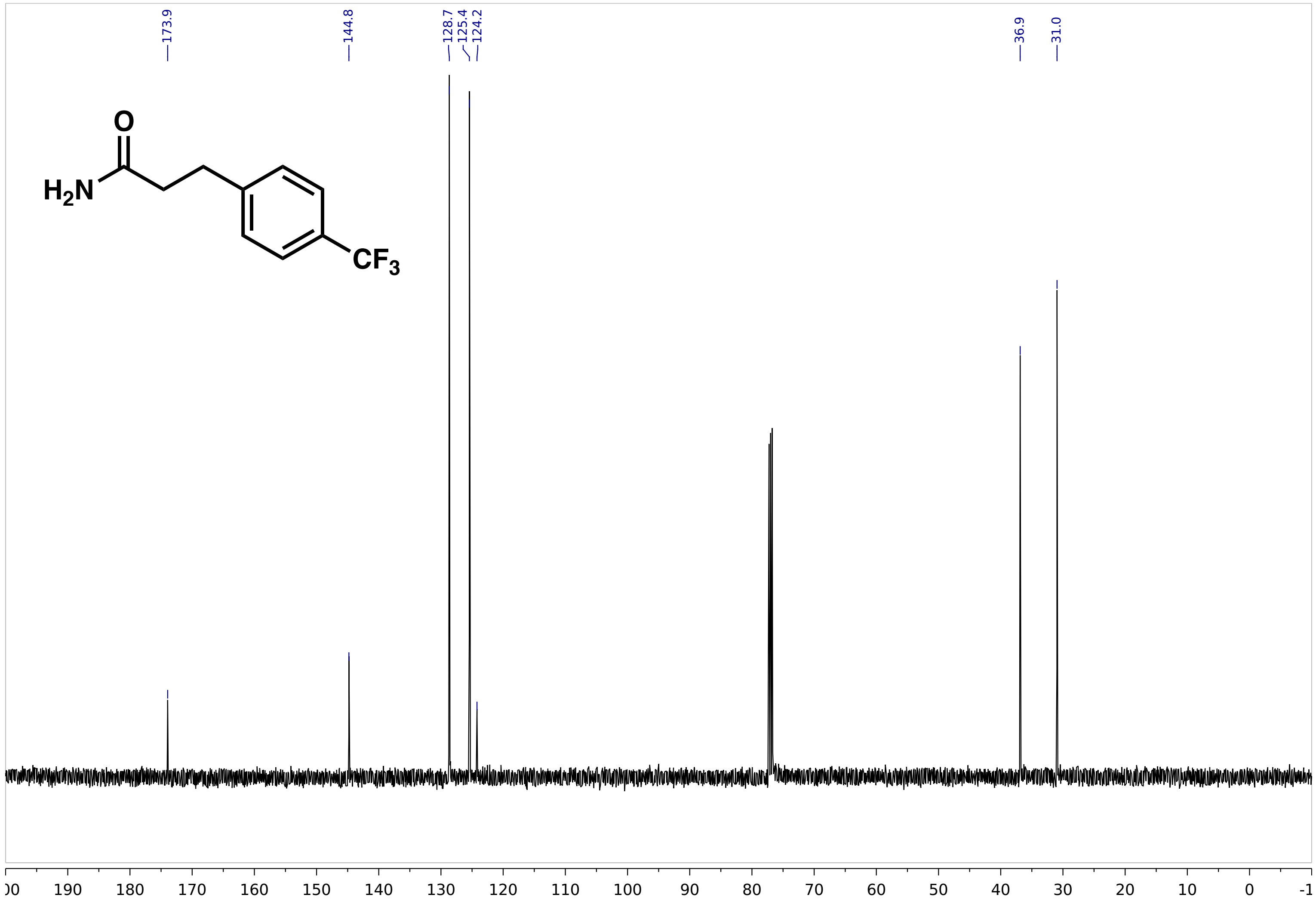

^{13}C NMR (126 MHz, $CDCl_3$, ^{1}H and ^{19}F decoupled) for 3-(4-trifluoromethylphenyl)propamide (**S3t**)

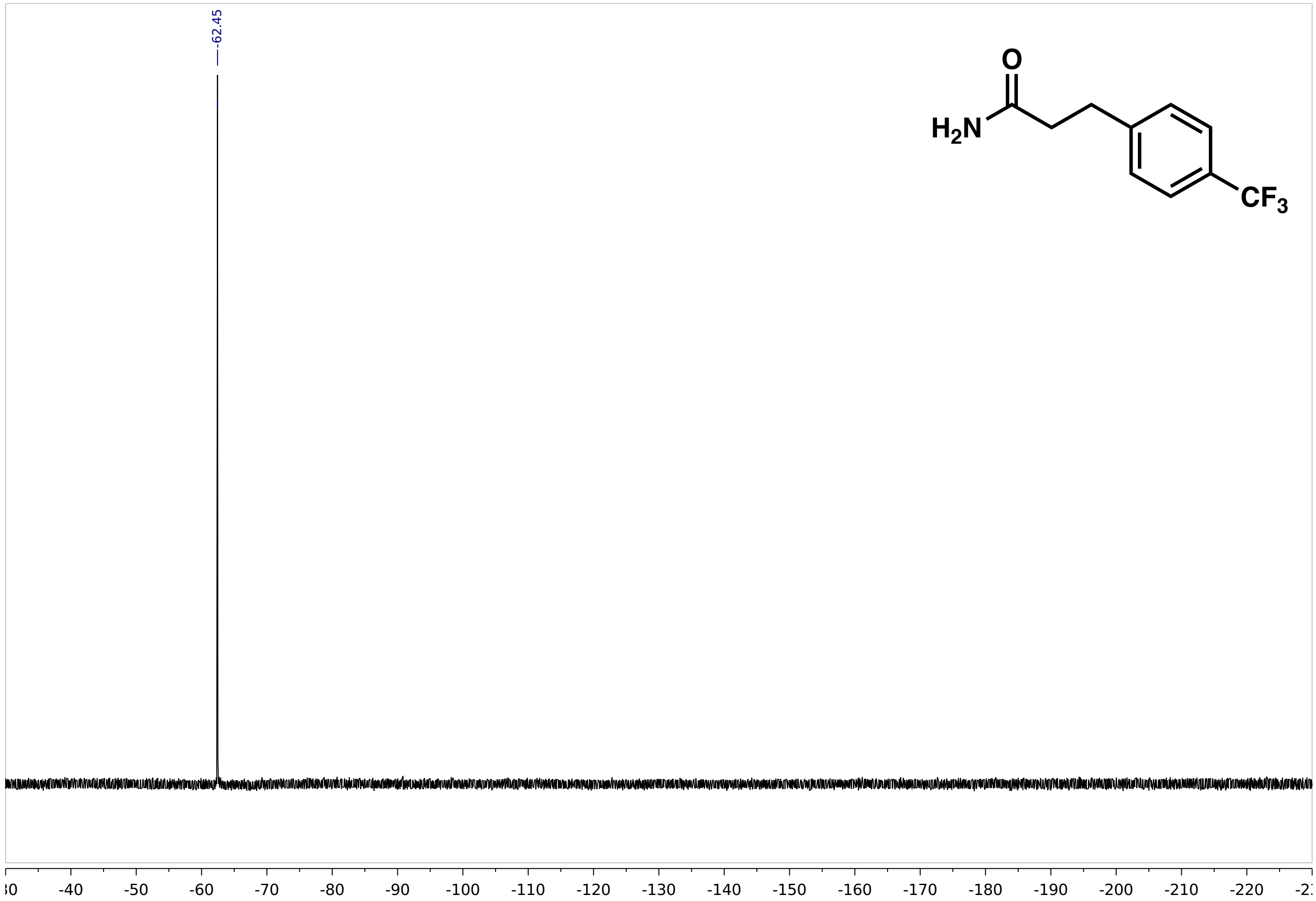

^{19}F NMR (376 MHz, $CDCl_3$) for 3-(4-trifluoromethylphenyl)propamide (**S3t**)

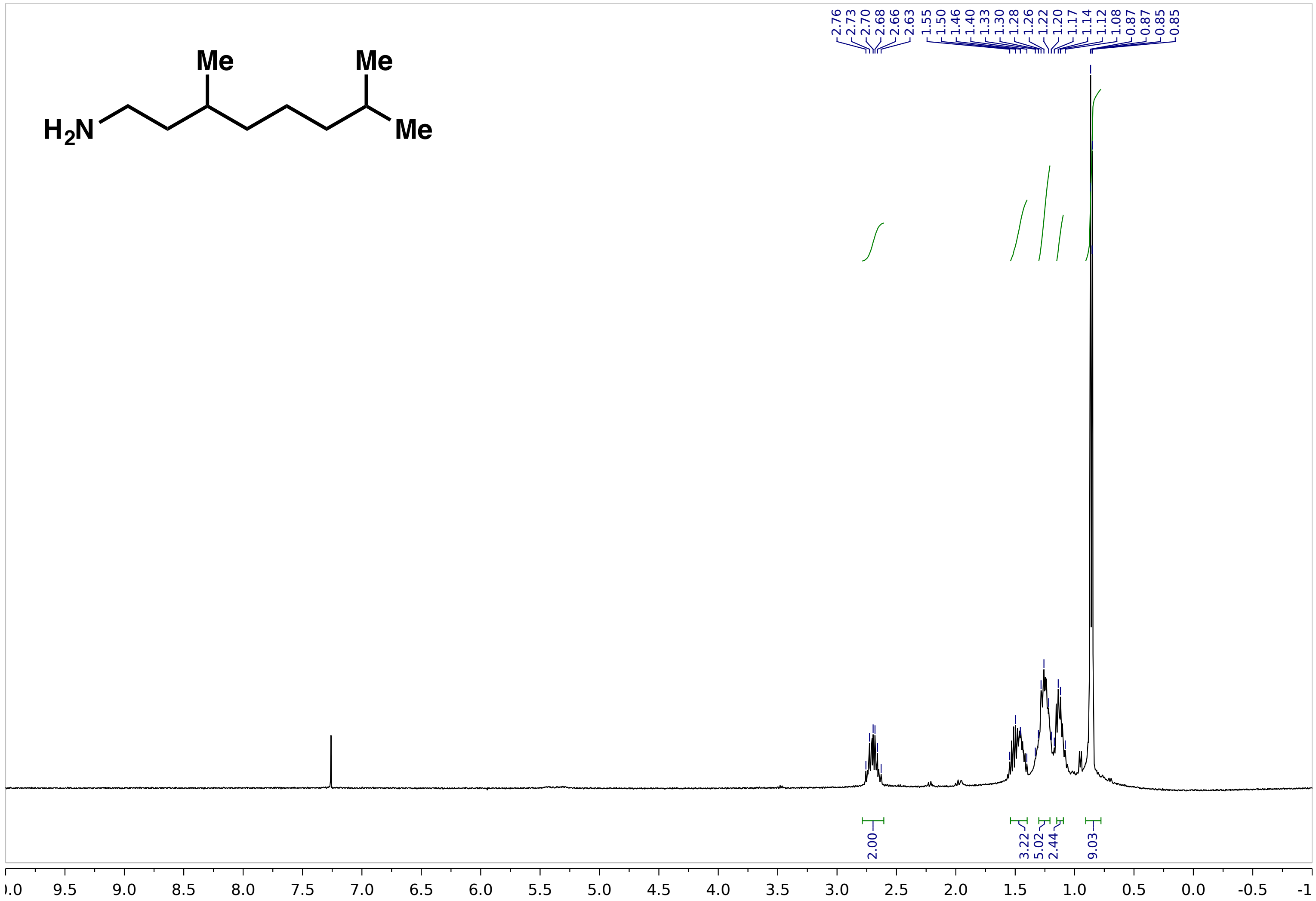

^{1}H NMR (400 MHz, $CDCl_3$) for 3,7-dimethyloctylamine (**S4h**)

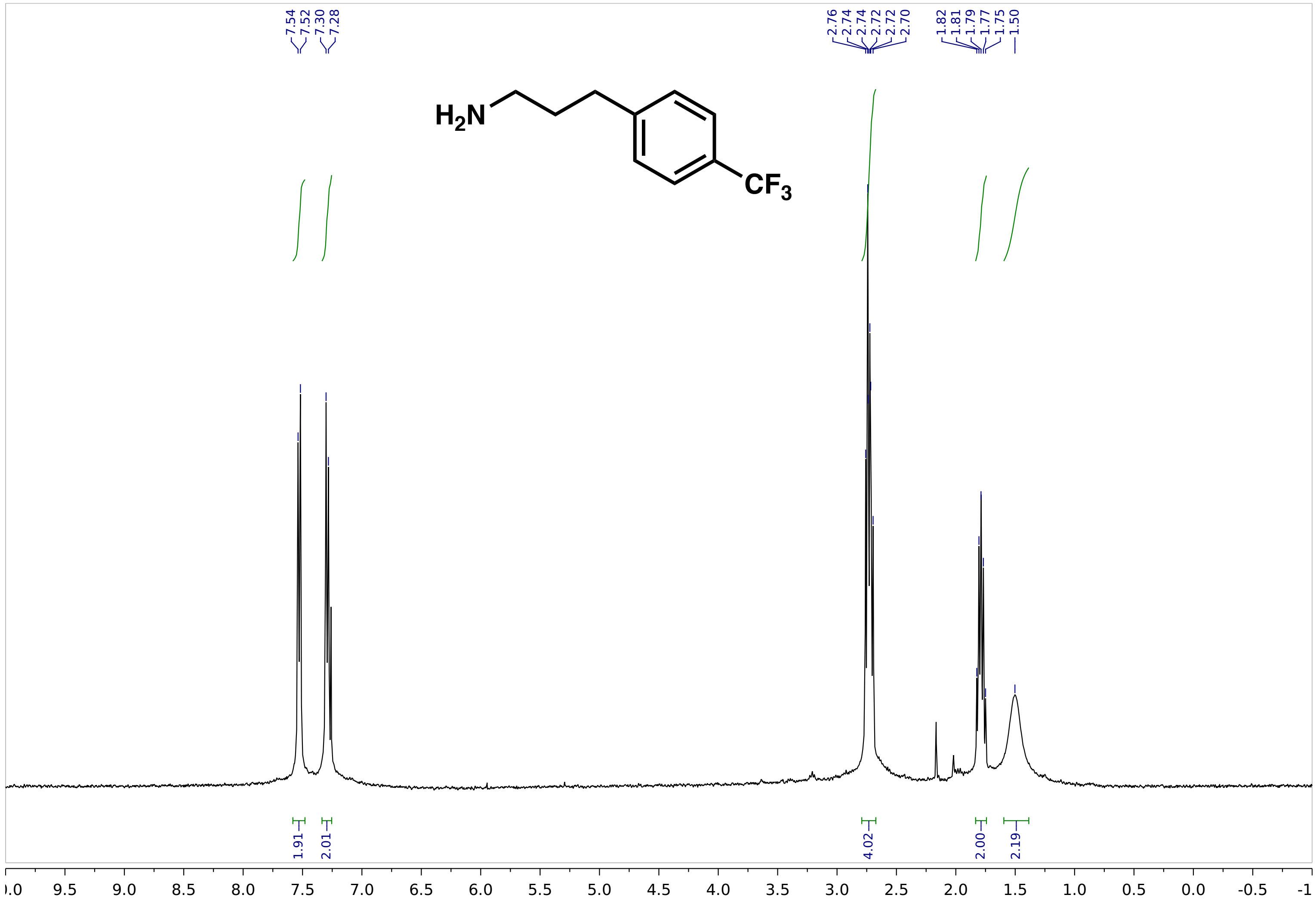

^{1}H NMR (400 MHz, $CDCl_3$) for 3-(4-(trifluoromethyl)phenyl)propylamine (**S4t**)

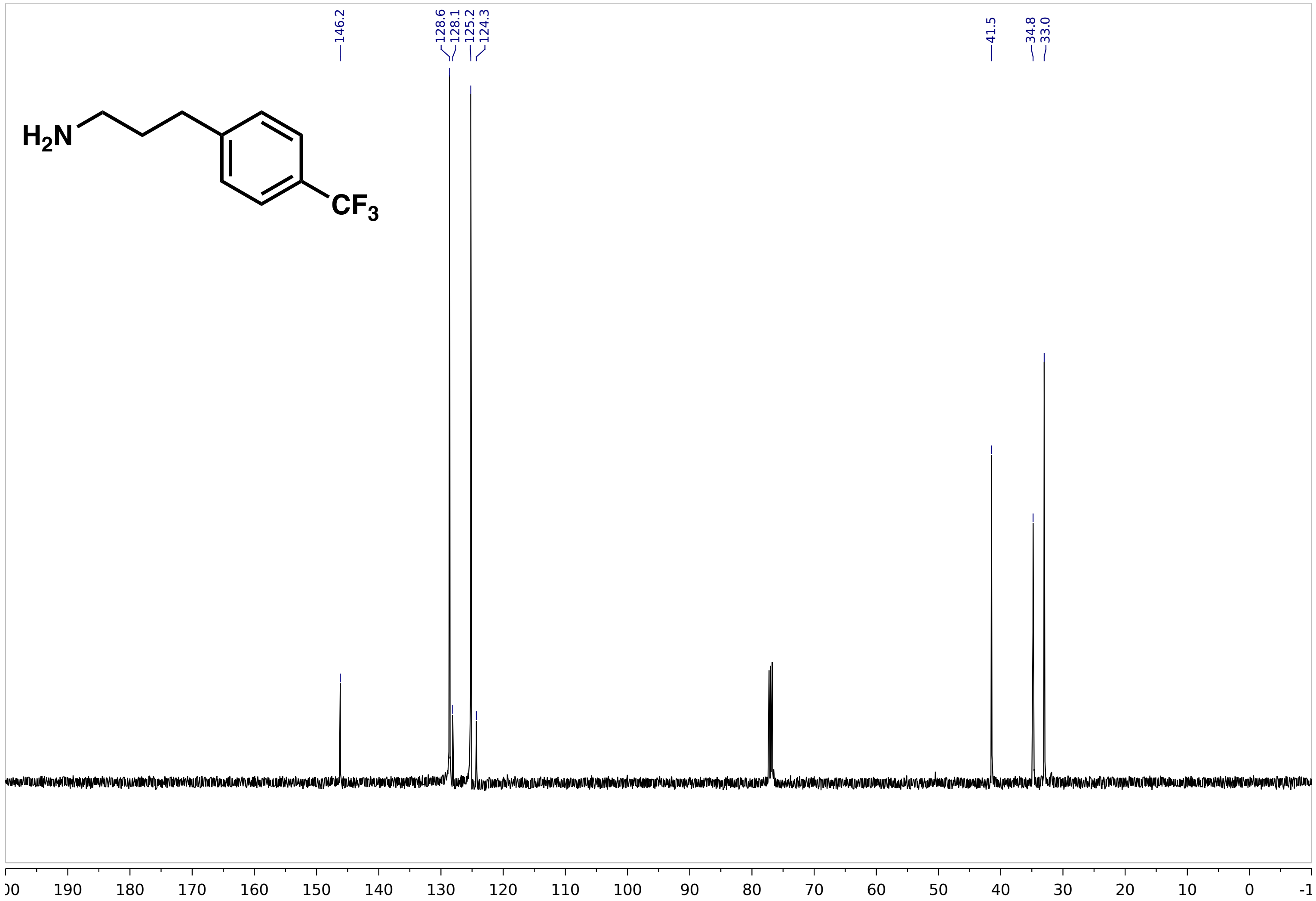

^{13}C NMR (126 MHz, $CDCl_3$, ^{1}H and ^{19}F decoupled) for 3-(4-(trifluoromethyl)phenyl)propylamine (**S4t**)

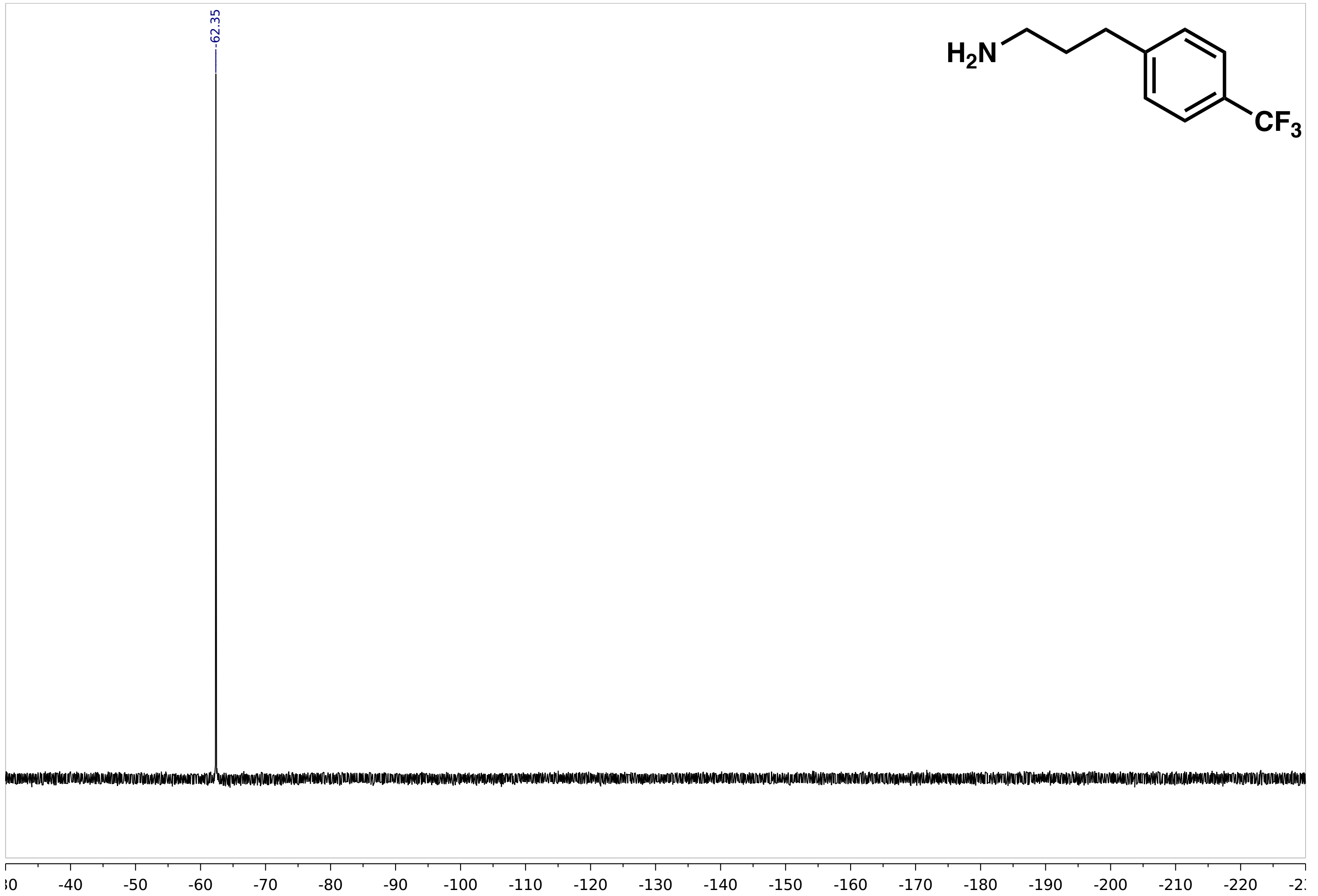

^{19}F NMR (376 MHz, $CDCl_3$) for 3-(4-(trifluoromethyl)phenyl)propylamine (**S4t**)

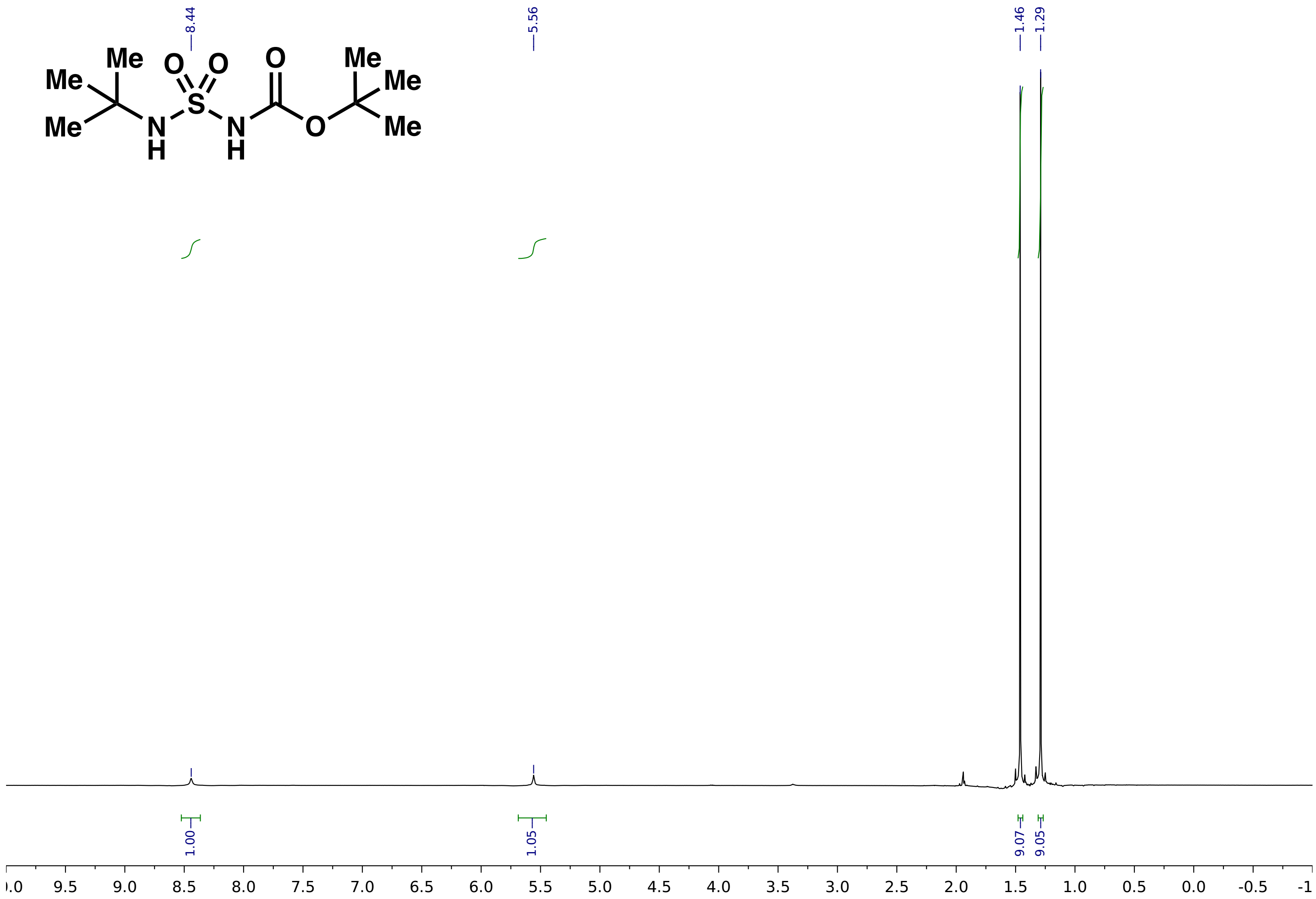

^{1}H NMR (400 MHz, CD_3CN) for *N*-*tert*-butoxycarbonyl-*N'*-*tert*-butylsulfamide (**S5a**)

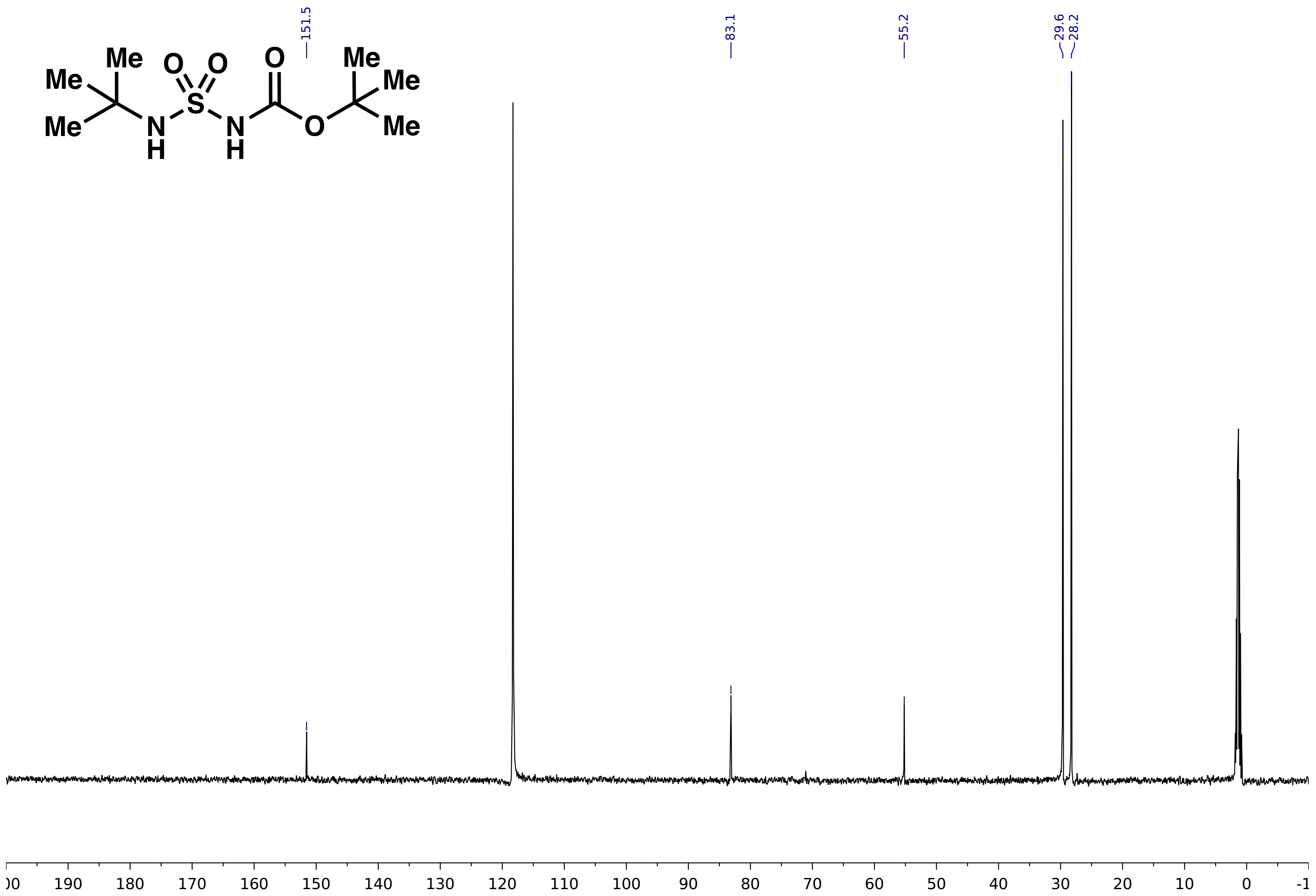

^{13}C NMR (126 MHz, CD_3CN) for *N*-*tert*-butoxycarbonyl-*N'*-*tert*-butylsulfamide (**S5a**)

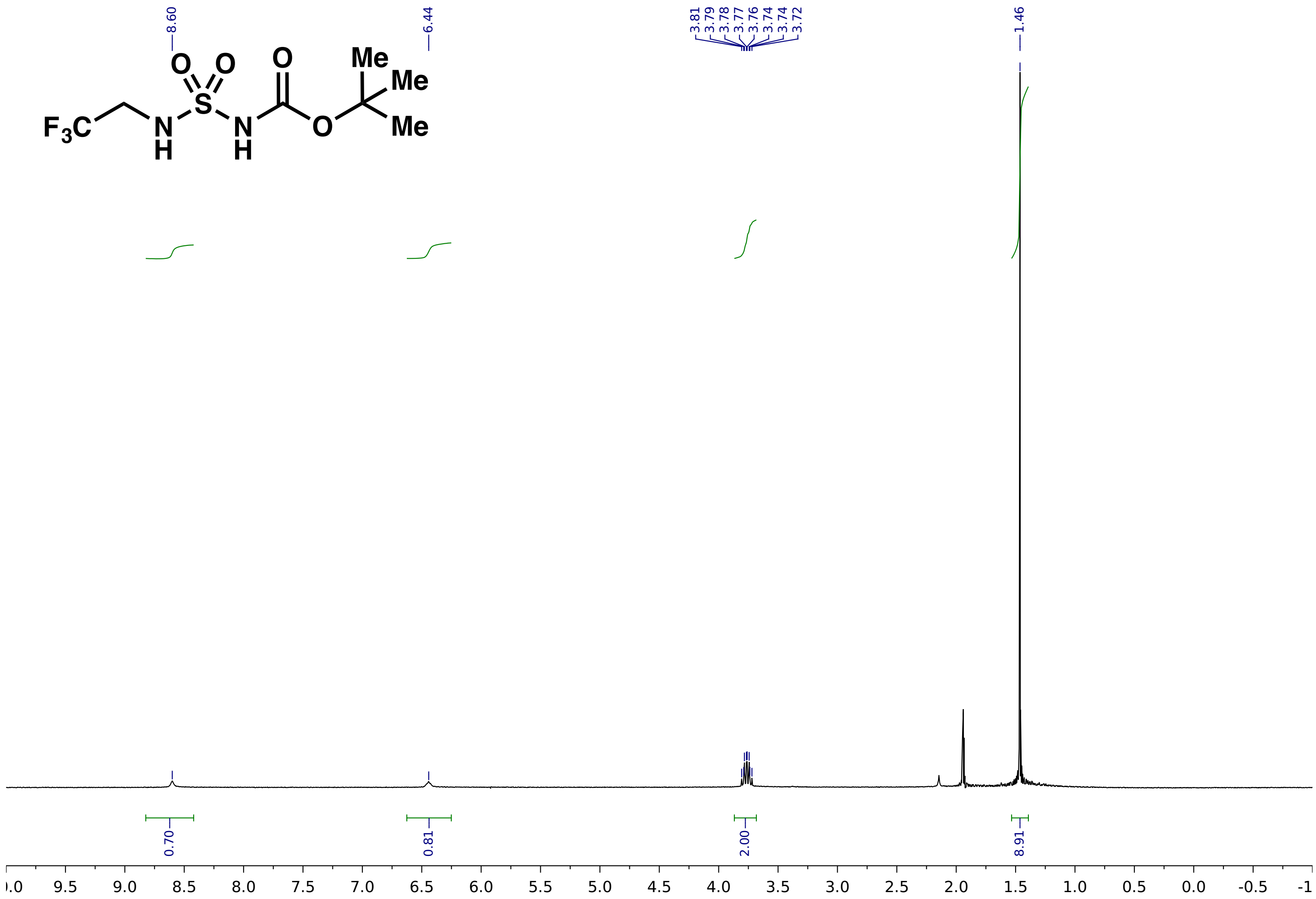

^{1}H NMR (400 MHz, CD_3CN) for *N*-*tert*-butoxycarbonyl-*N'*-(2,2,2-trifluoroethyl)sulfamide (**S5b**)

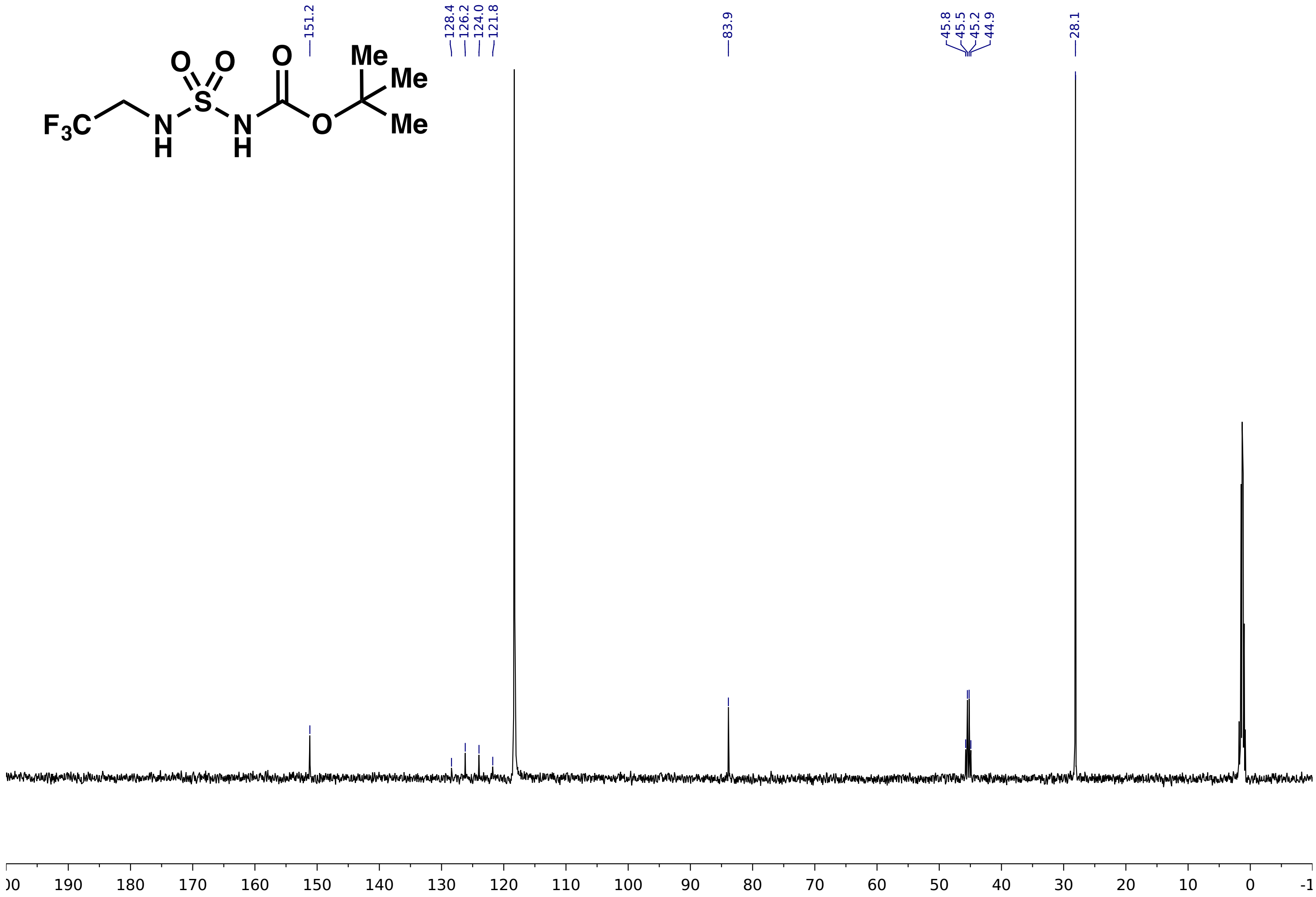

^{13}C NMR (126 MHz, CD_3CN) for *N*-*tert*-butoxycarbonyl-*N'*-(2,2,2-trifluoroethyl)sulfamide (**S5b**)

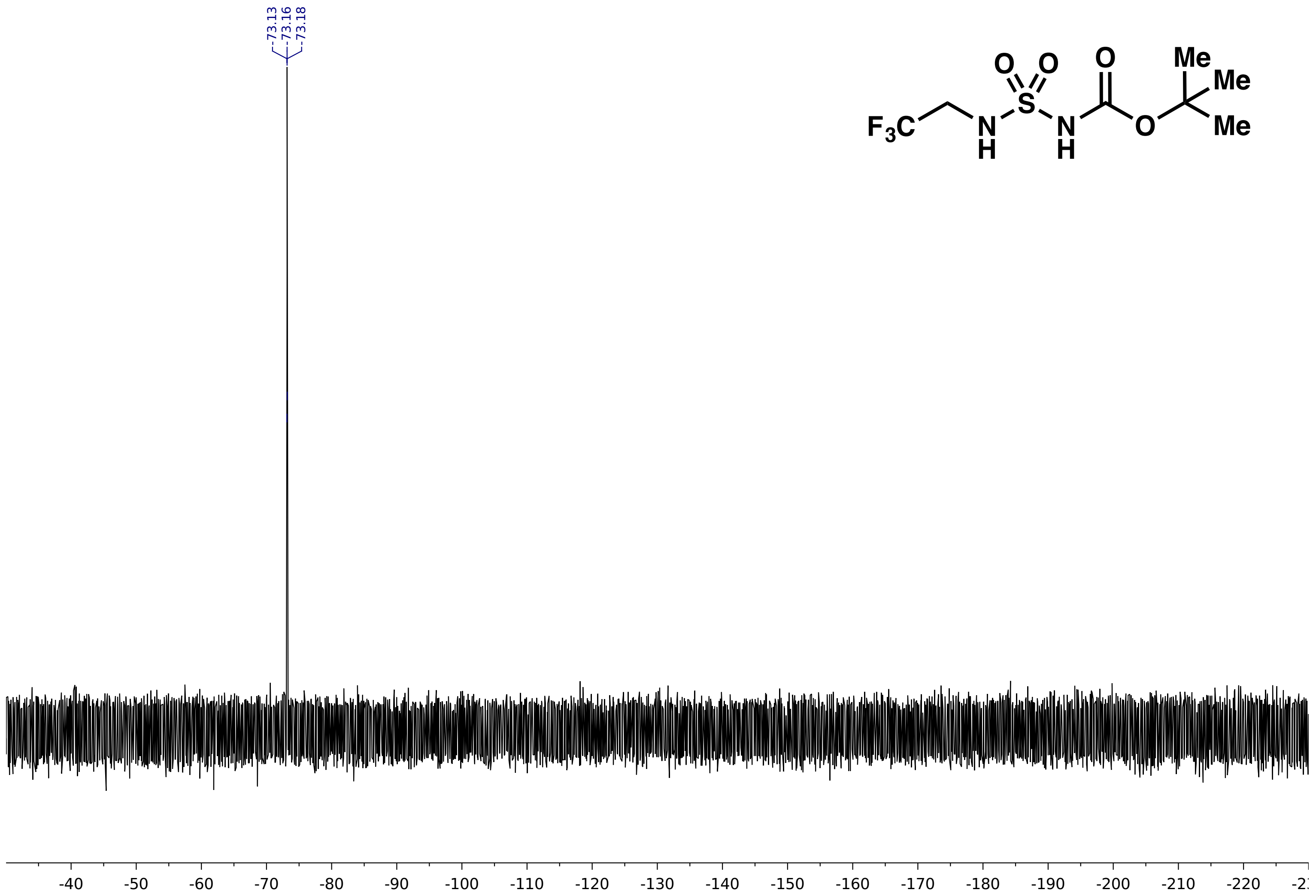

^{19}F NMR (376 MHz, CD_3CN) for *N*-*tert*-butoxycarbonyl-*N'*-(2,2,2-trifluoroethyl)sulfamide (**S5b**)

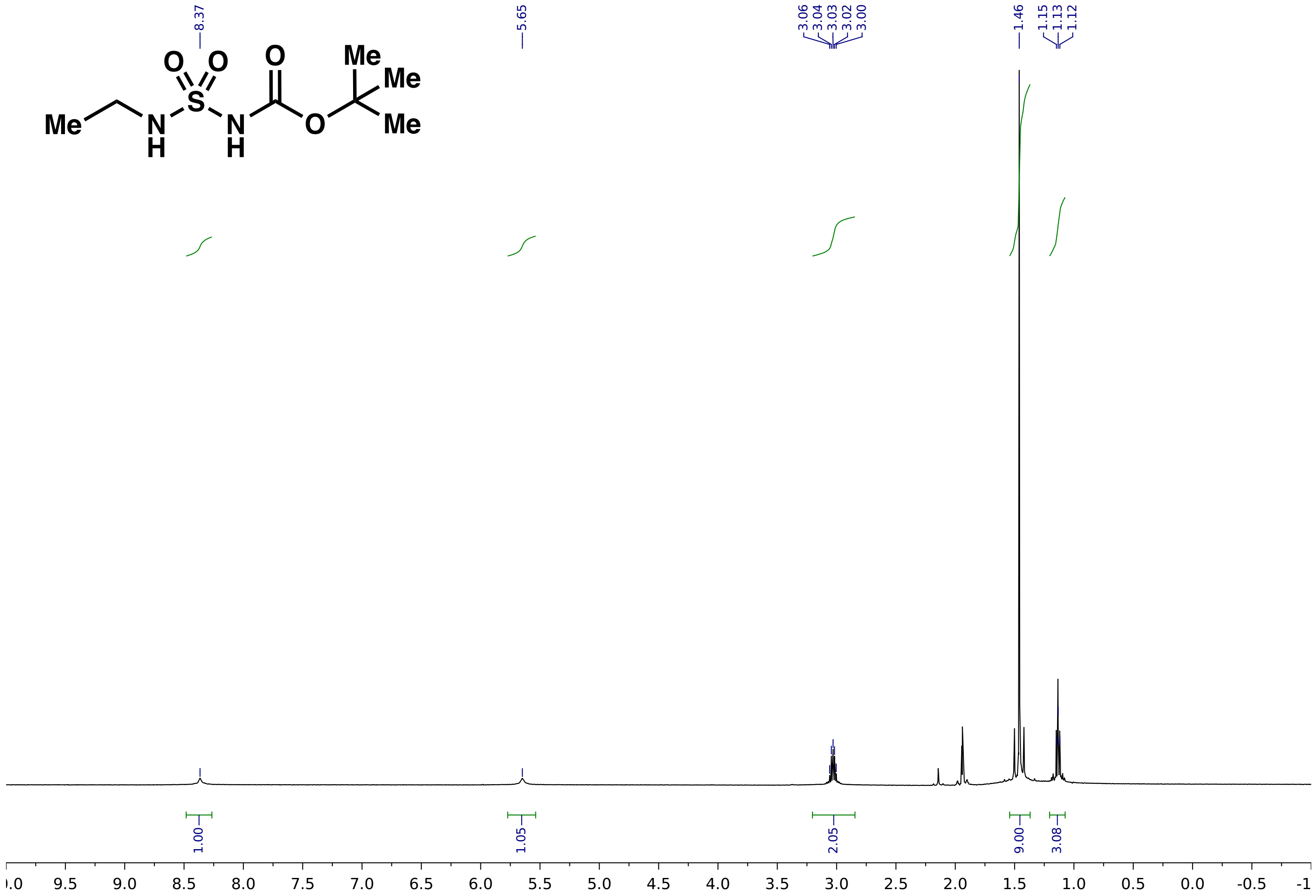

^{1}H NMR (500 MHz, CD_3CN) for *N*-*tert*-butoxycarbonyl-*N’*-ethylsulfamide (**S5c**)

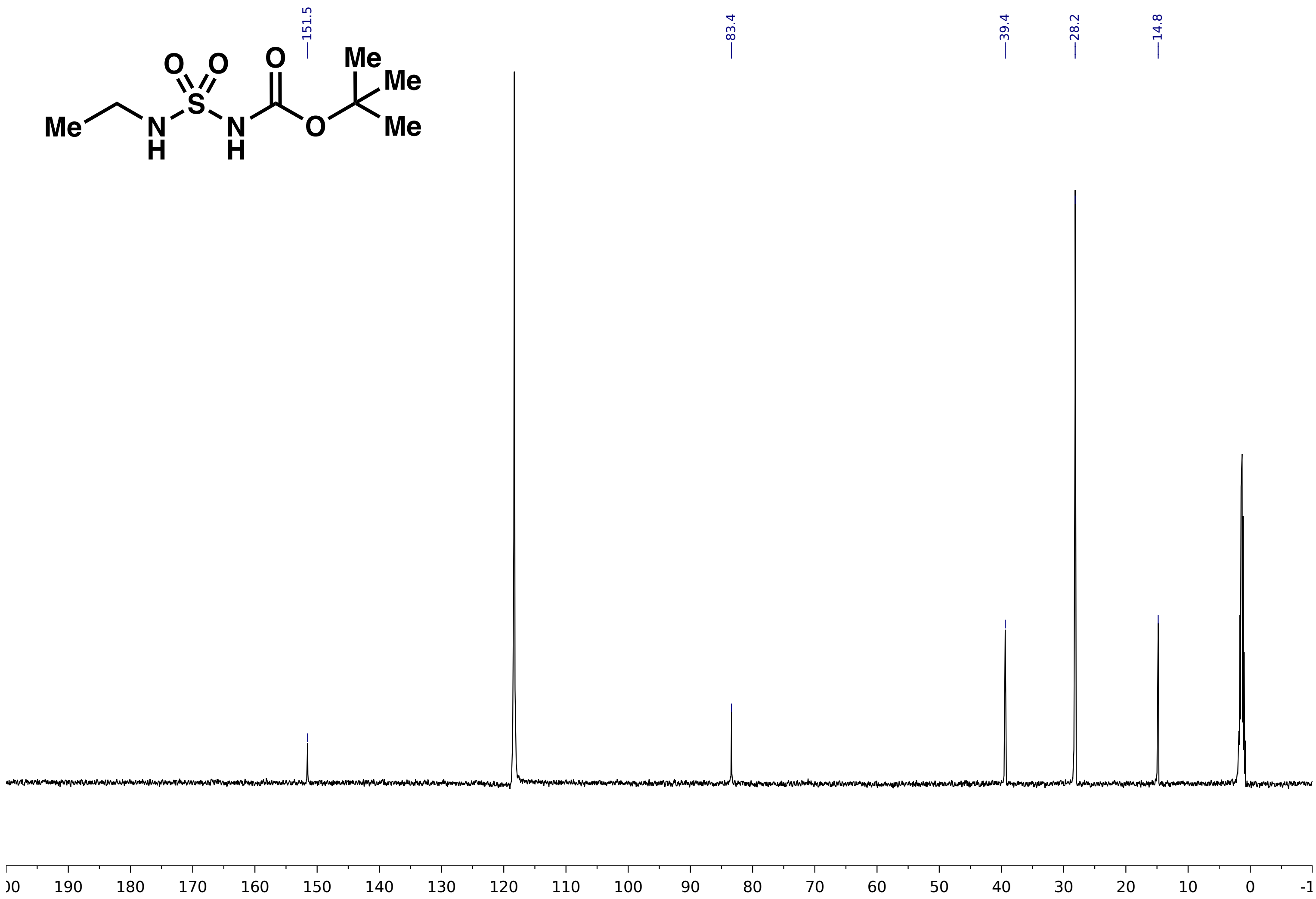

^{13}C NMR (126 MHz, CD_3CN) for *N*-*tert*-butoxycarbonyl-*N'*-ethylsulfamide (**S5c**)

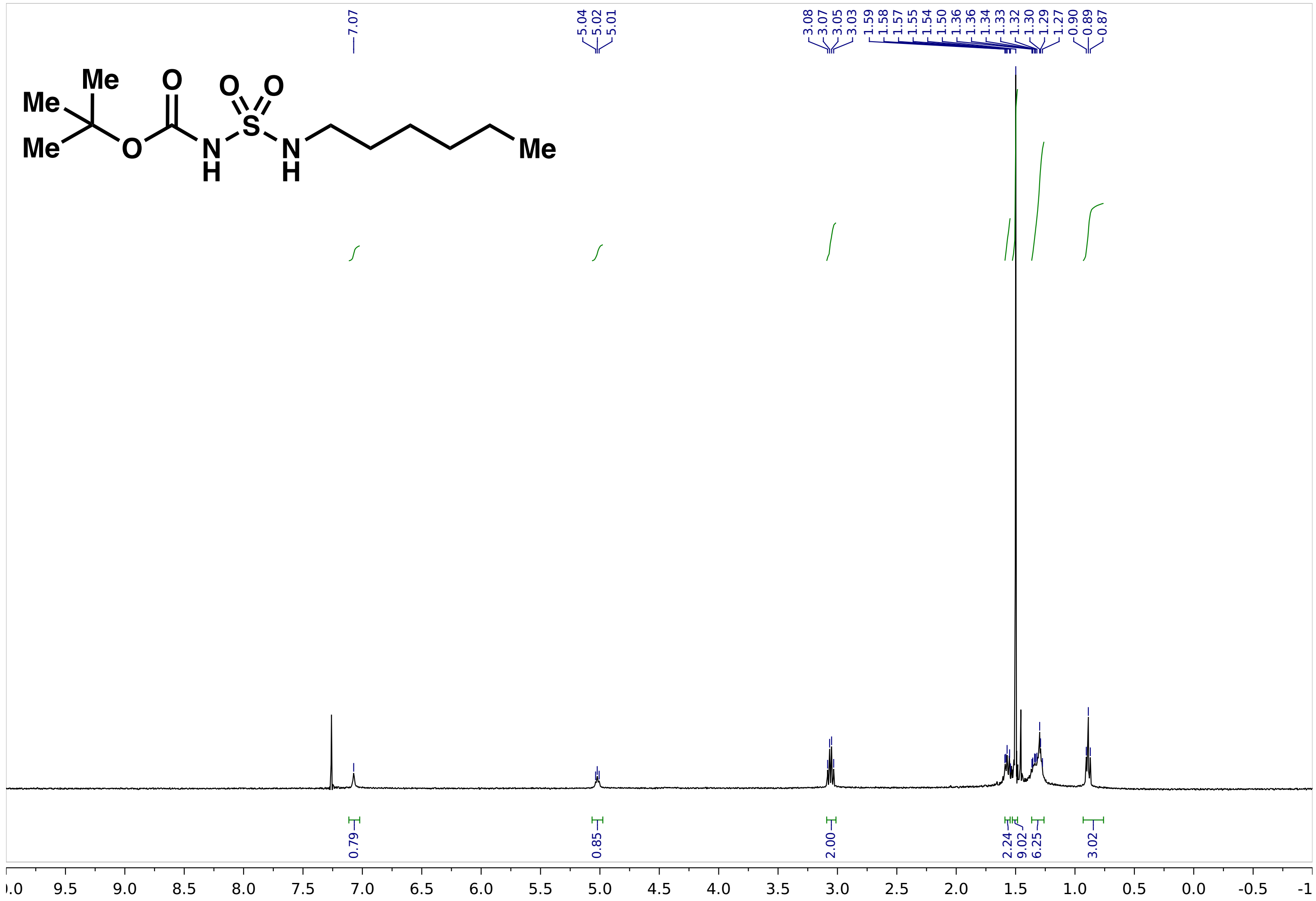

^1H NMR (400 MHz, $CDCl_3$) for *N*-*tert*-butoxycarbonyl-*N*'-hexyl sulfamide (**S5d**)

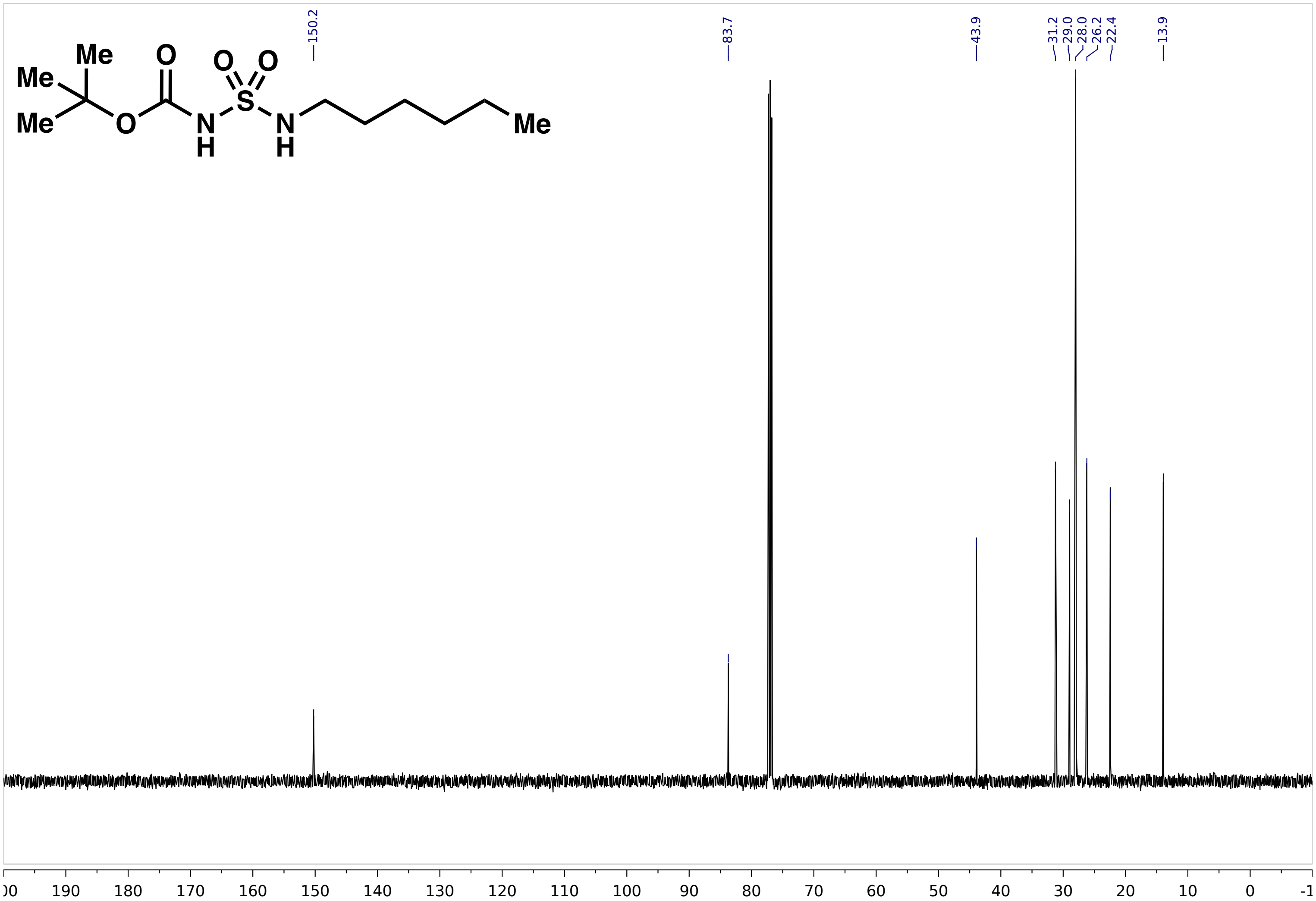

^{13}C NMR (126 MHz, $CDCl_3$) for *N*-*tert*-butoxycarbonyl-*N*'-hexyl sulfamide (**S5d**)

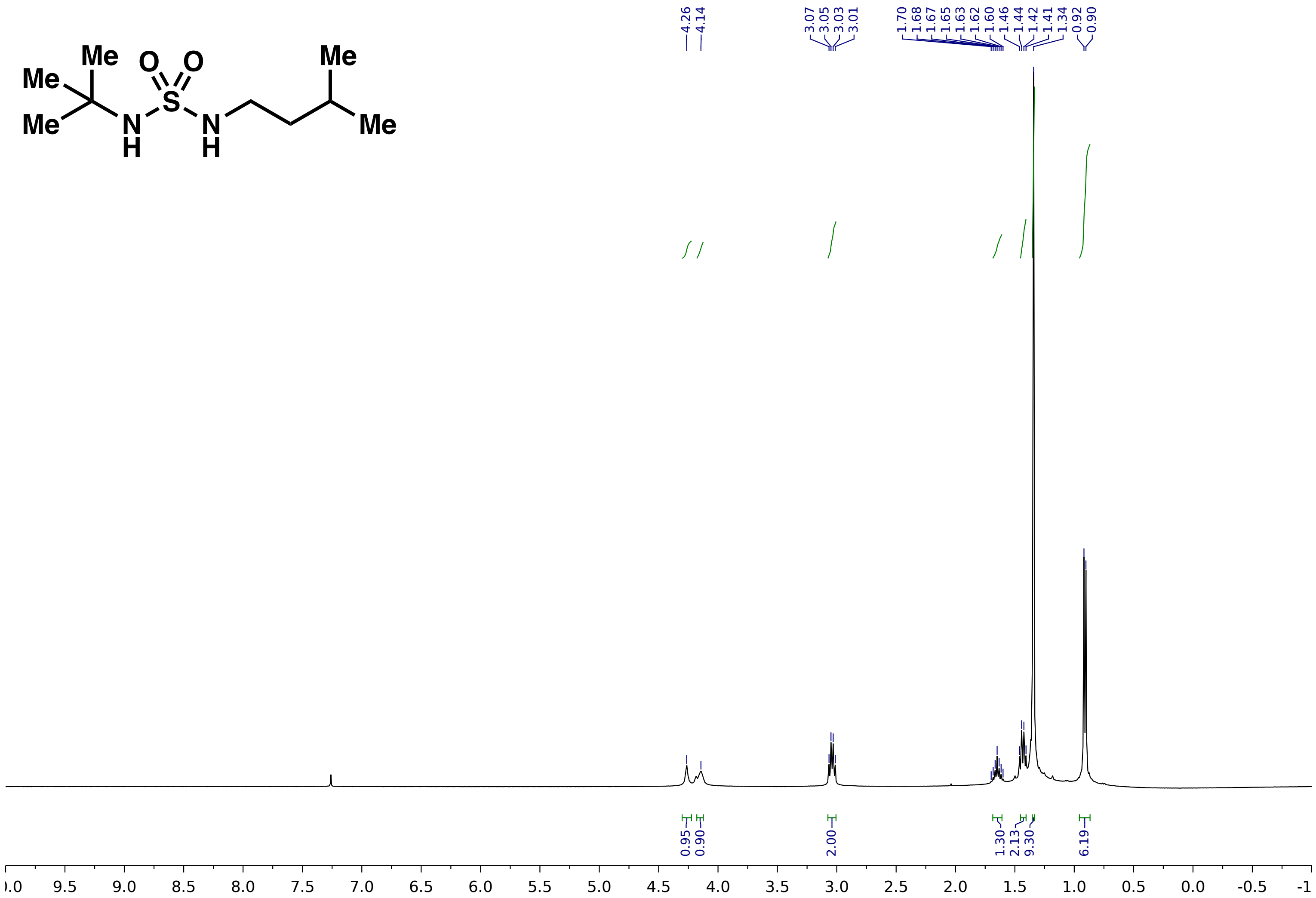

^{1}H NMR (400 MHz, $CDCl_3$) for *N*-*tert*-butyl-*N'*-isopentylsulfamide (**S6c**)

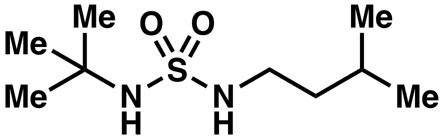

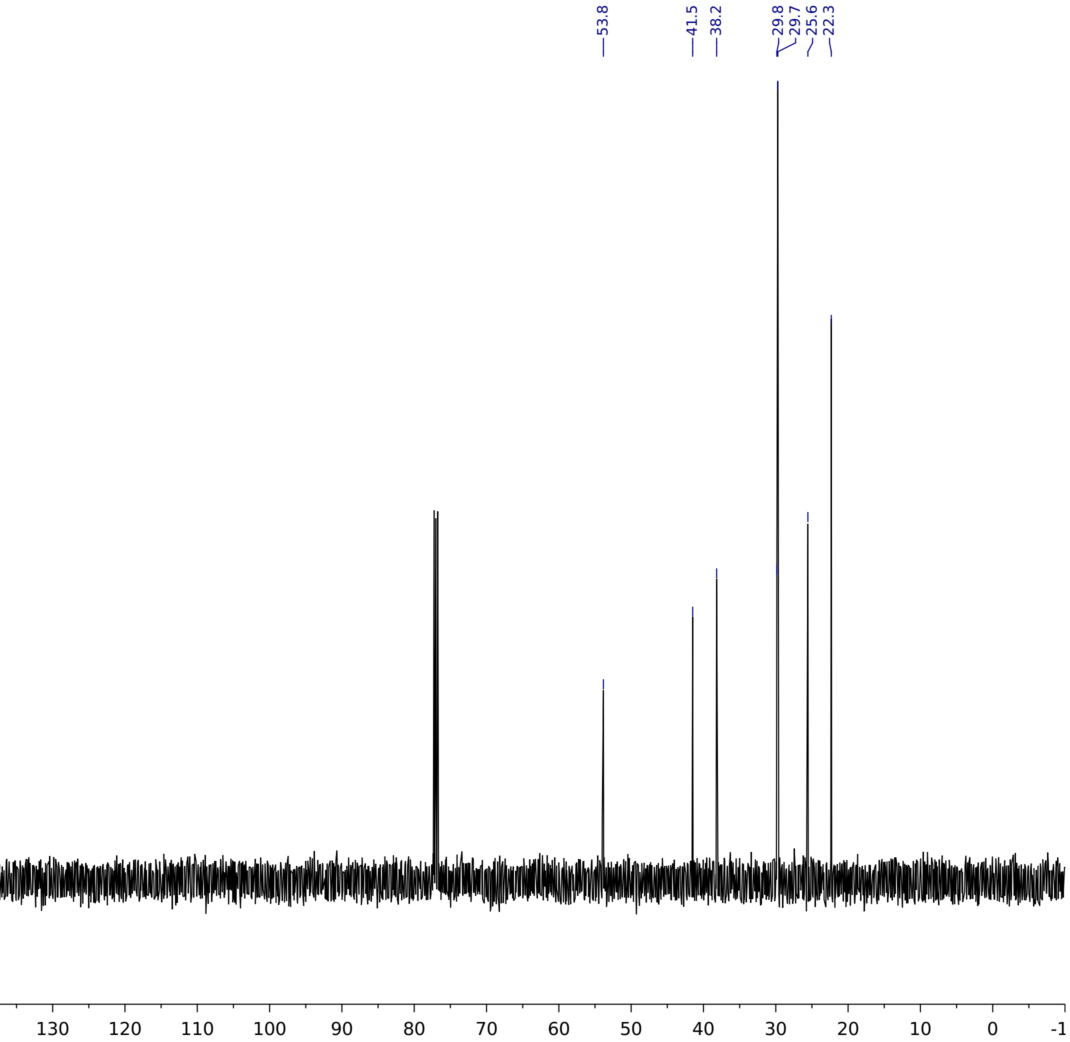

^{13}C NMR (126 MHz, $CDCl_3$) for *N*-*tert*-butyl-*N'*-isopentylsulfamide (**S6c**)

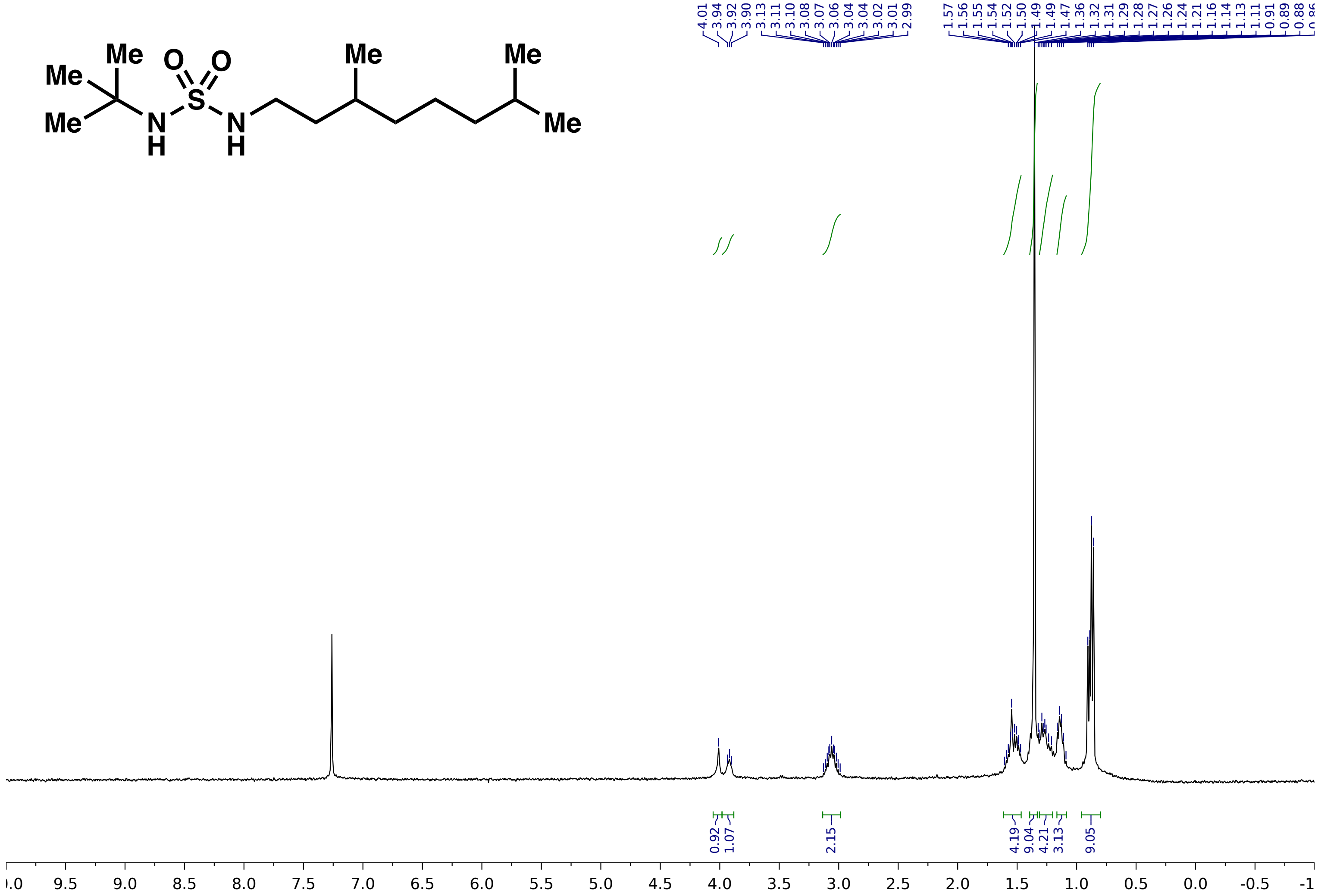

^{1}H NMR (400 MHz, $CDCl_3$) for *N*-*tert*-butyl-*N'*-(3,7-dimethyloct-1-yl)sulfamide (**S6h**)

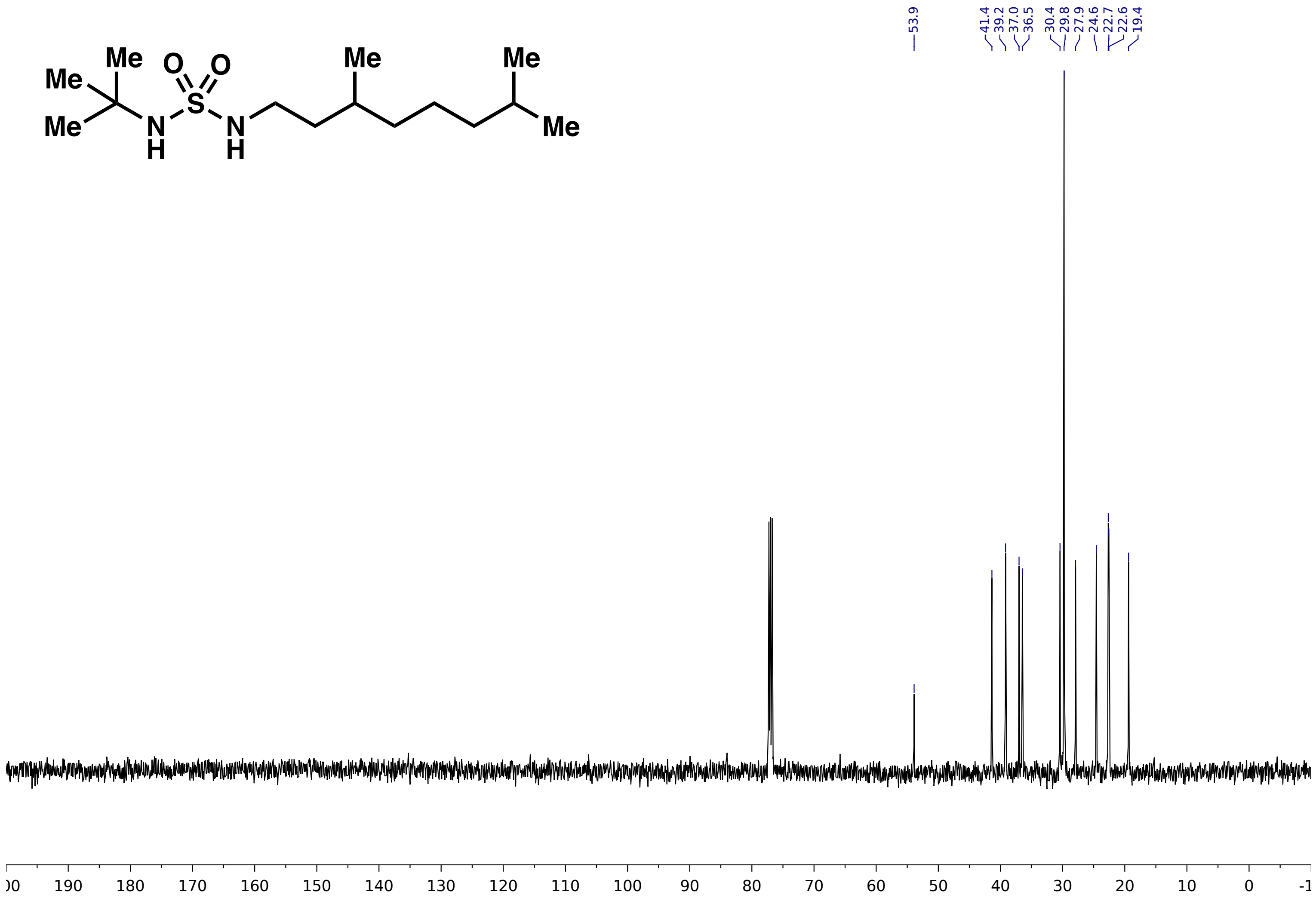

^{13}C NMR (126 MHz, $CDCl_3$) for *N*-*tert*-butyl-*N'*-(3,7-dimethyloct-1-yl)sulfamide (**S6h**)

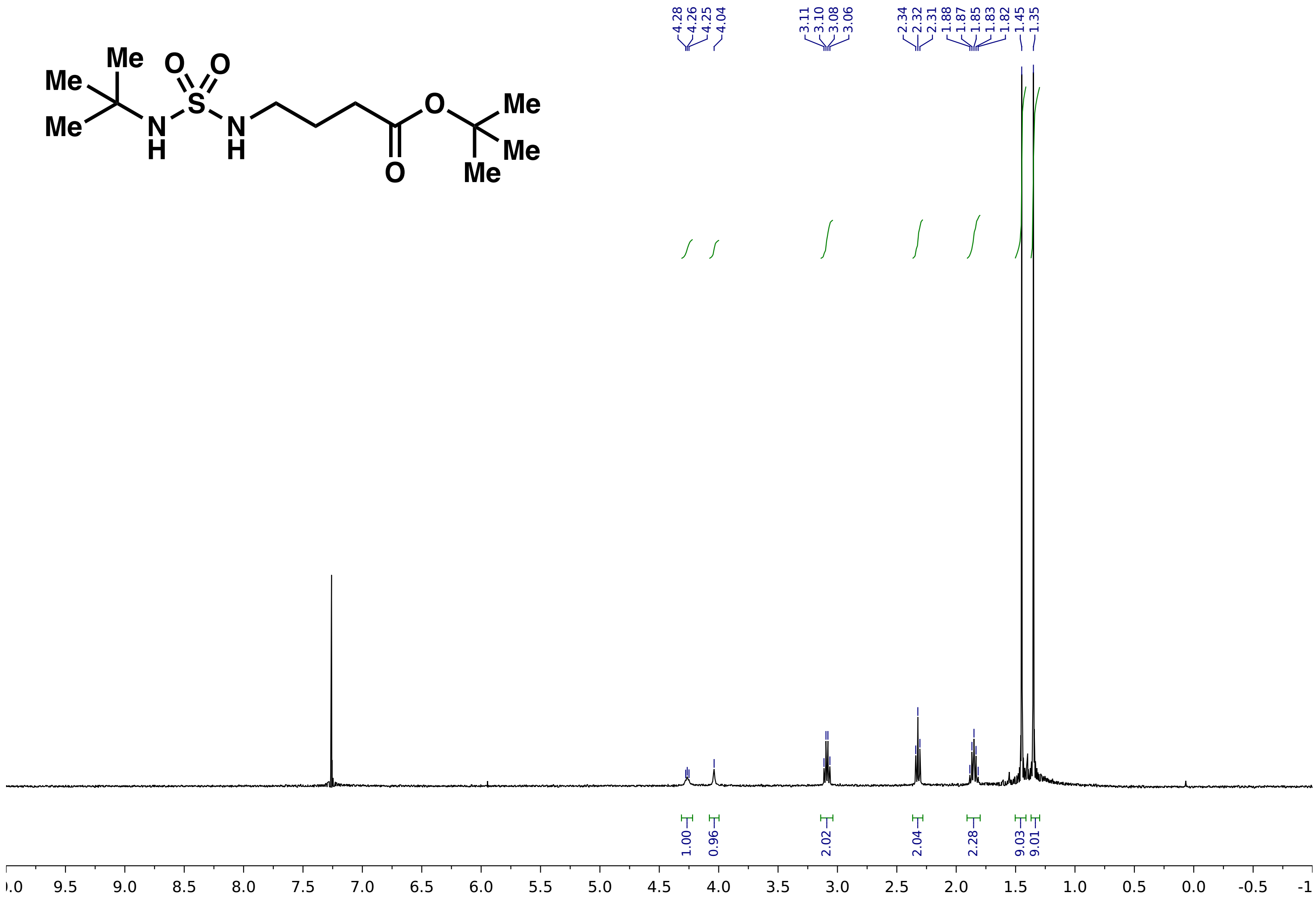

^{1}H NMR (400 MHz, $CDCl_3$) for *N*-(*tert*-butyl butano-4-yl)-*N'*-*tert*-butylsulfamide (**S6t**)

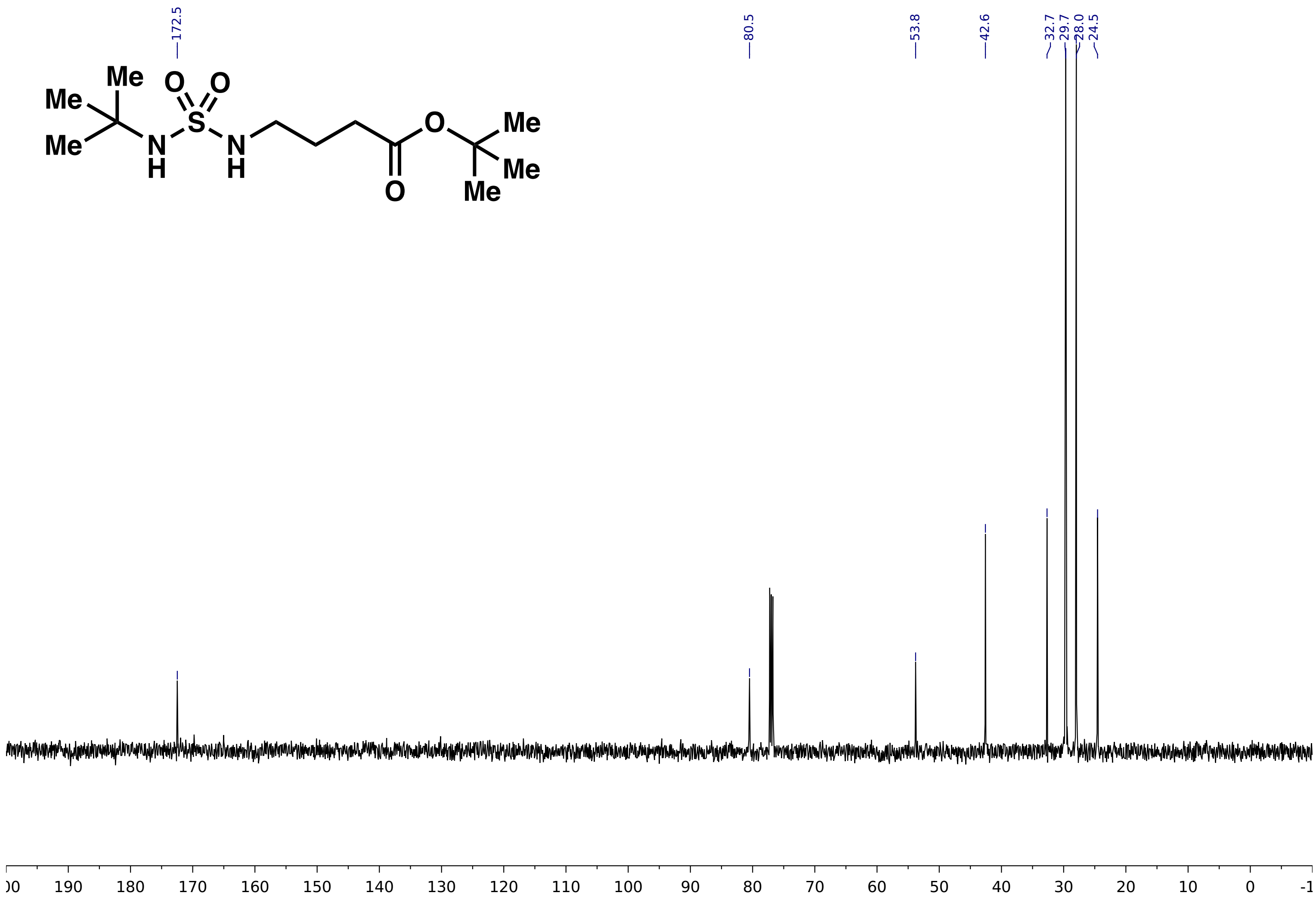

^{13}C NMR (126 MHz, $CDCl_3$) for *N*-(*tert*-butyl butano-4-yl)-*N'*-*tert*-butylsulfamide (**S6t**)

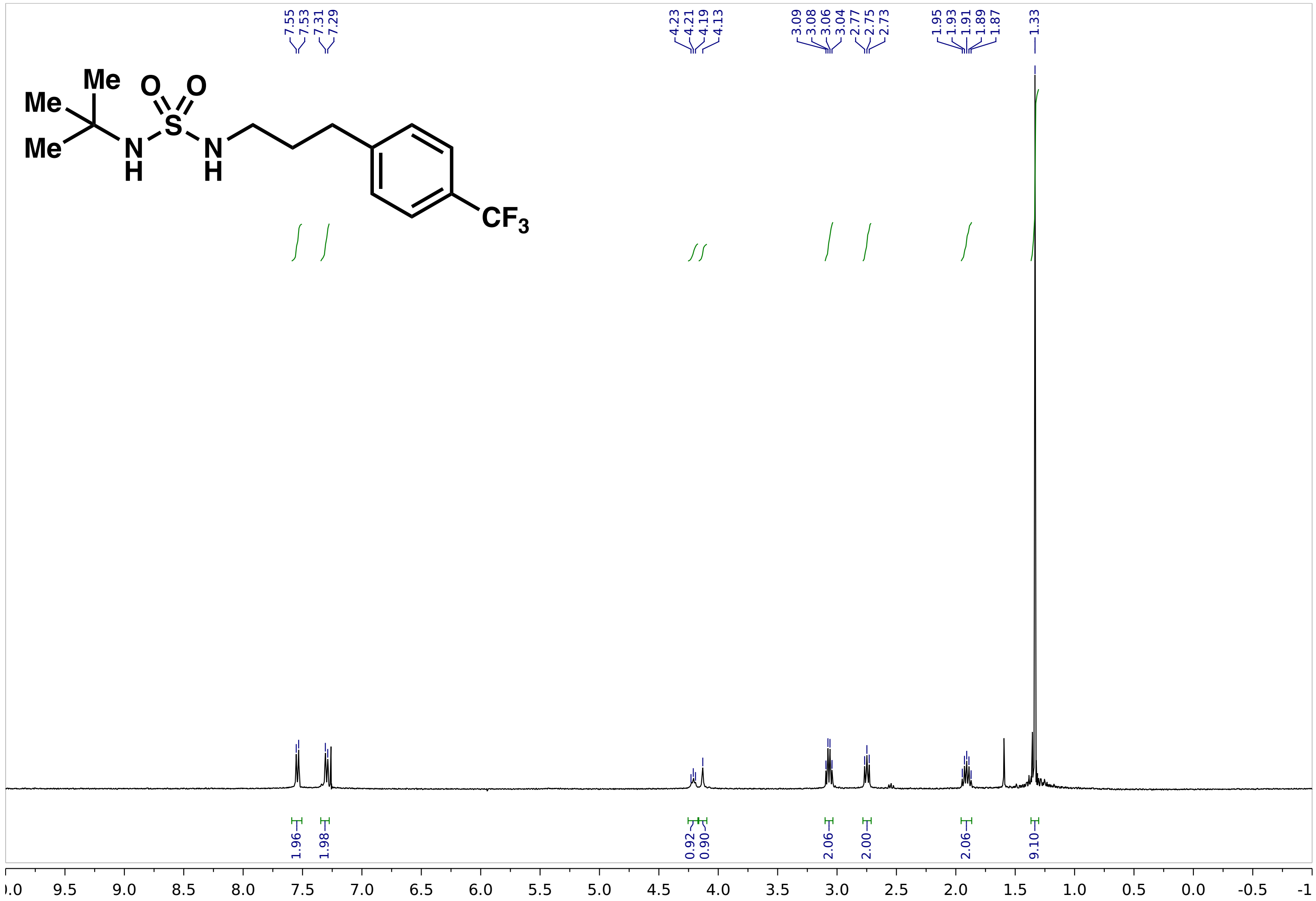

^{1}H NMR (400 MHz, $CDCl_3$) for *N*-*tert*-butyl-*N'*-(3-(4-trifluoromethyl)phenyl)prop-1-yl)sulfamide (**S6u**)

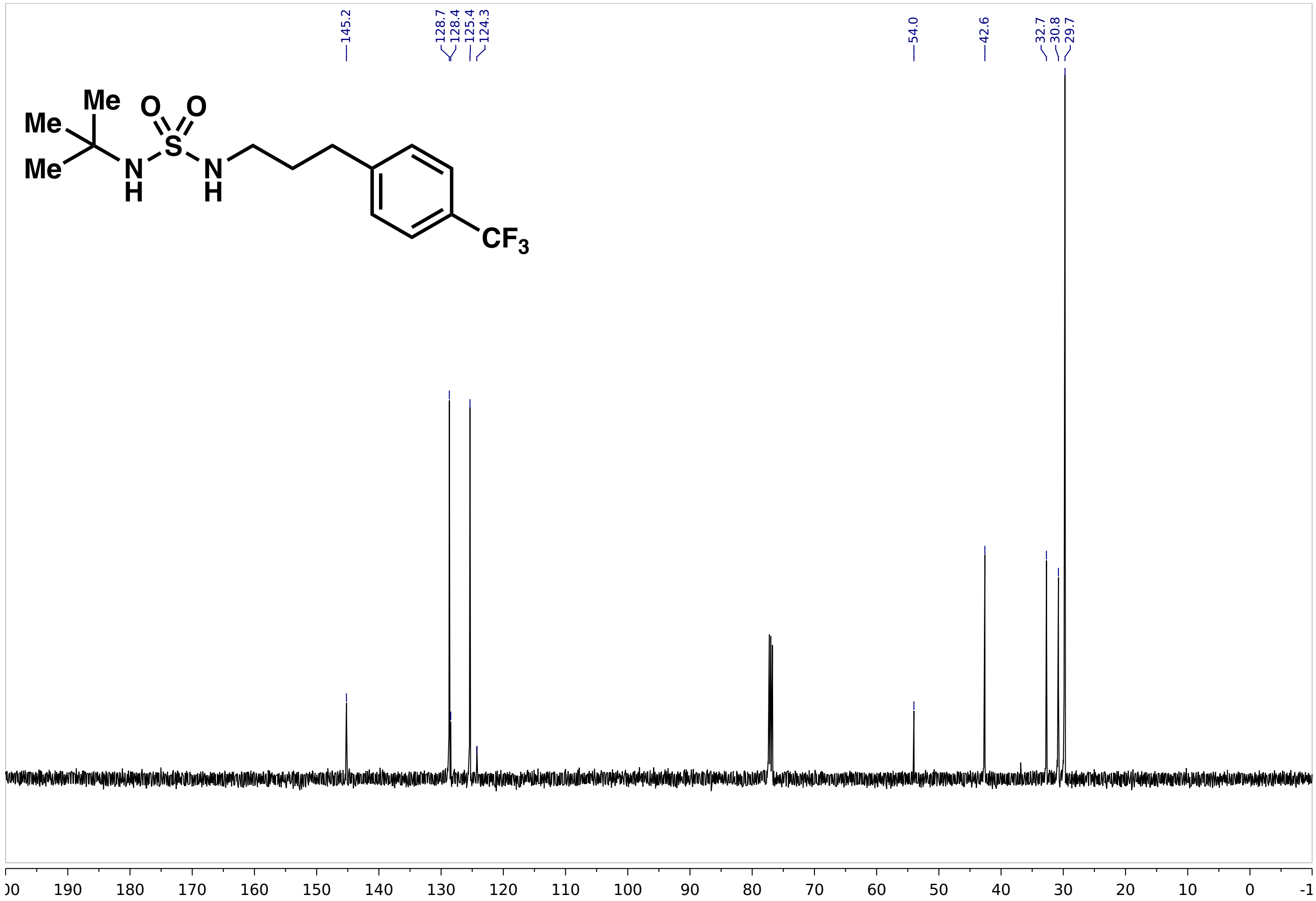

^{13}C NMR (126 MHz, $CDCl_3$, ^1H and ^{19}F decoupled) for *N-tert*-butyl-*N'*-(3-(4-trifluoromethyl)phenyl)prop-1-yl)sulfamide (**S6u**)

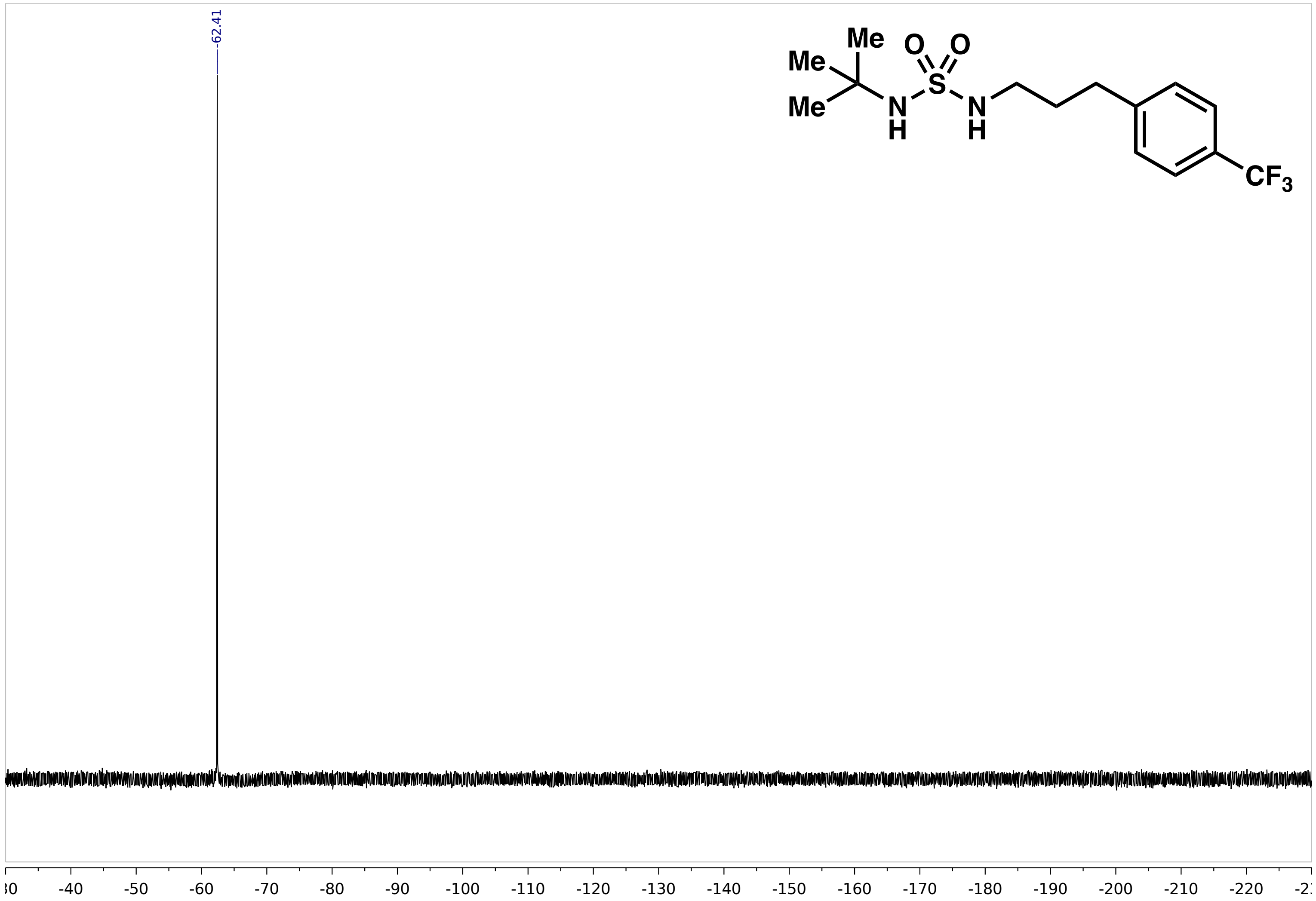

^{19}F NMR (376 MHz, $CDCl_3$) for *N*-*tert*-butyl-*N'*-(3-(4-trifluoromethyl)phenyl)prop-1-yl)sulfamide (**S6u**)

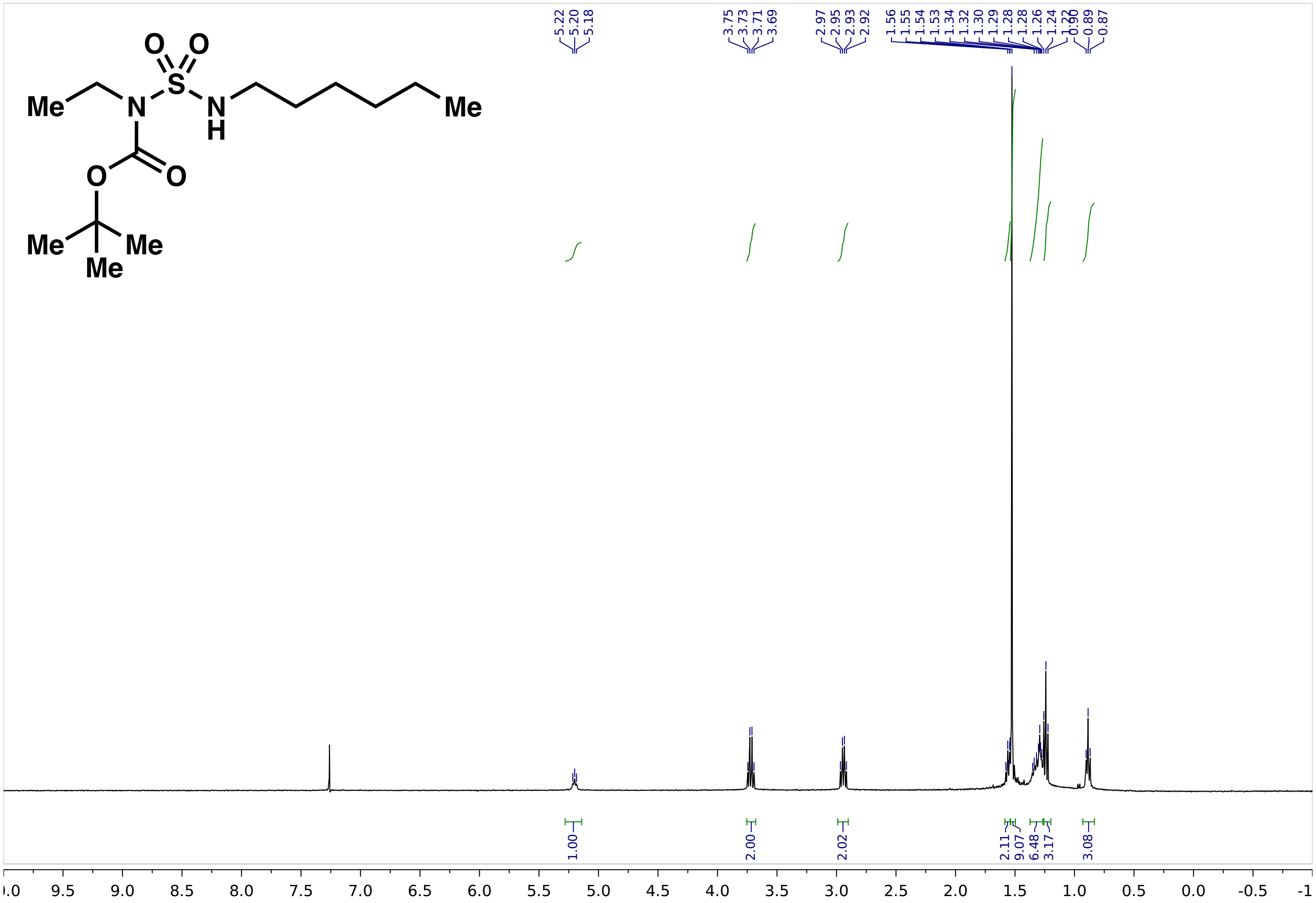

^{1}H NMR (400 MHz, $CDCl_3$) for *N*-*tert*-butoxycarbonyl-*N*-ethyl-*N*'-hexylsulfamide (**S7a**)

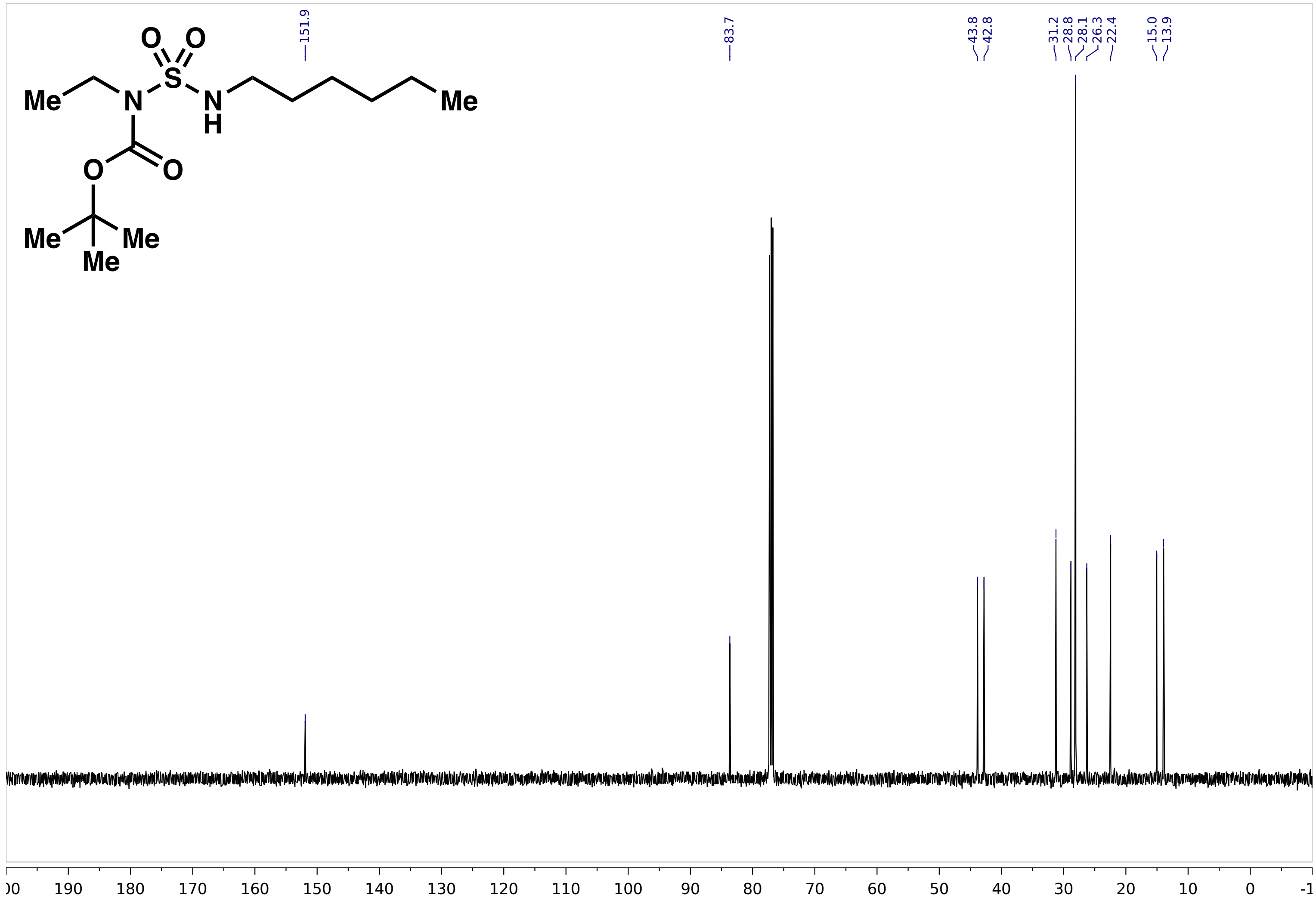

^{13}C NMR (126 MHz, $CDCl_3$) for *N*-*tert*-butoxycarbonyl-*N*-ethyl-*N*’-hexylsulfamide (**S7a**)

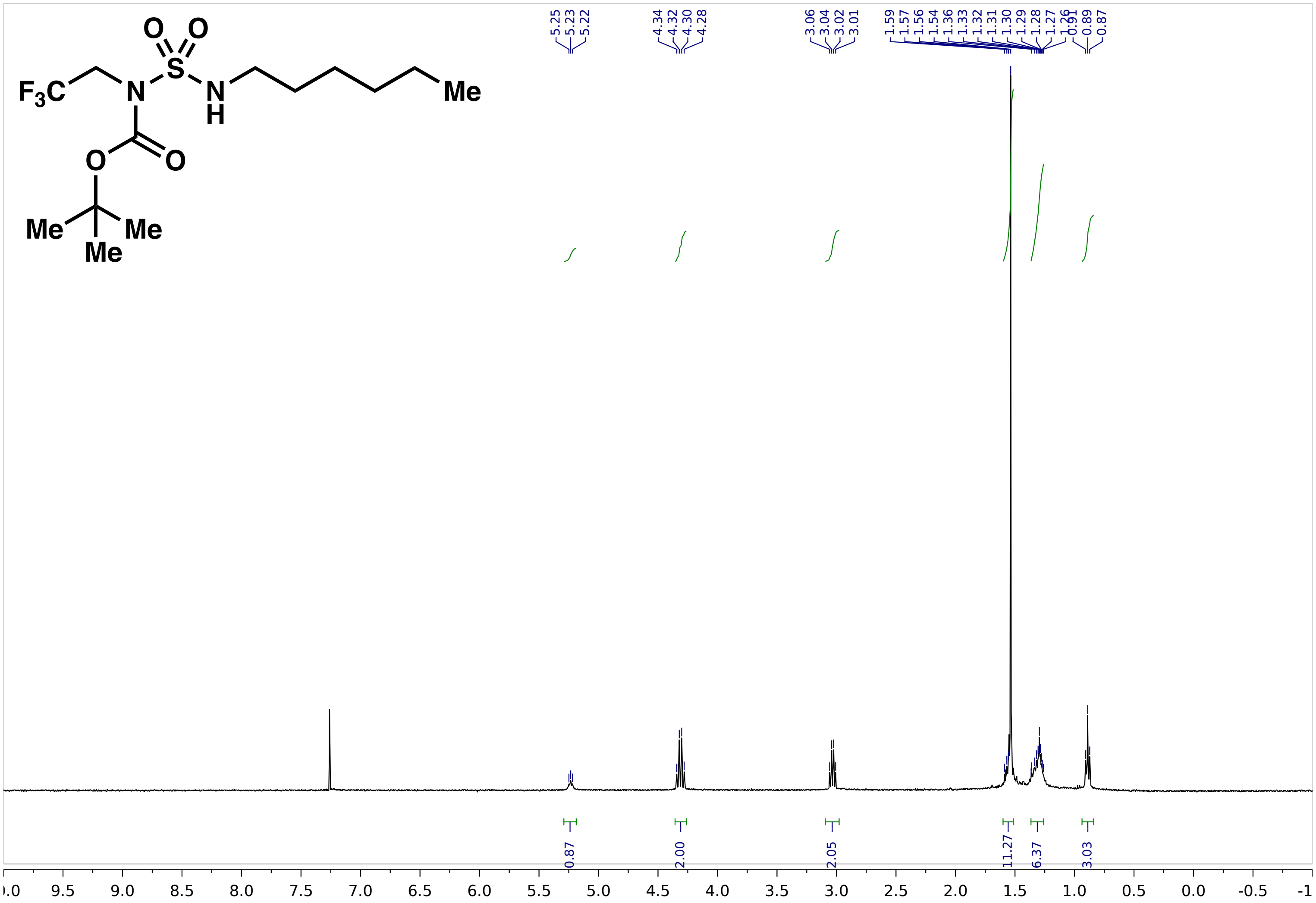

^{1}H NMR (400 MHz, CDCl₃) for *N*-*tert*-butoxycarbonyl-*N*'-hexyl-*N*-(2,2,2-trifluoro)ethylsulfamide (**S7b**)

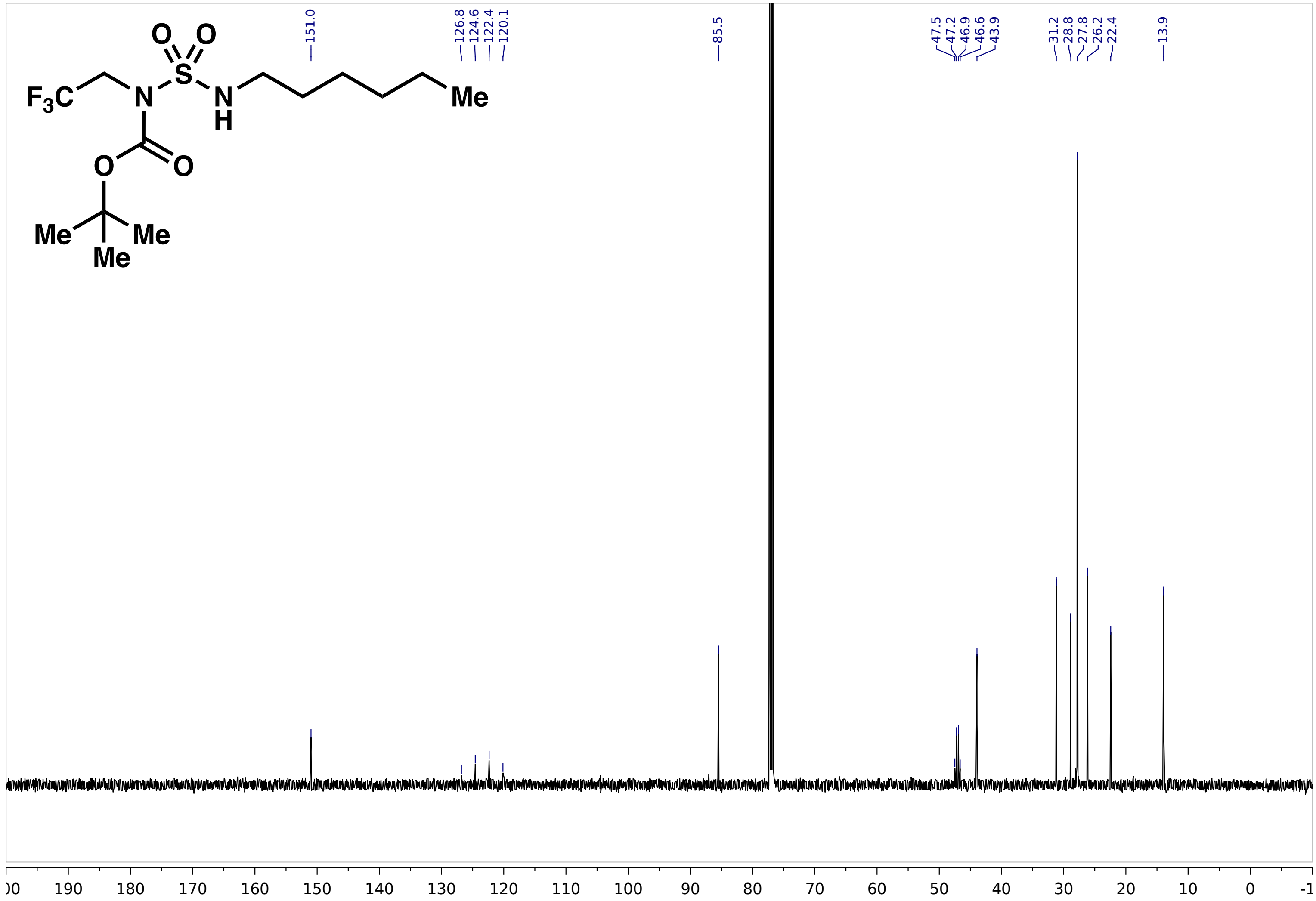

^{13}C NMR (126 MHz, $CDCl_3$) for *N*-*tert*-butoxycarbonyl-*N*'-hexyl-*N*-(2,2,2-trifluoro)ethylsulfamide (**S7b**)

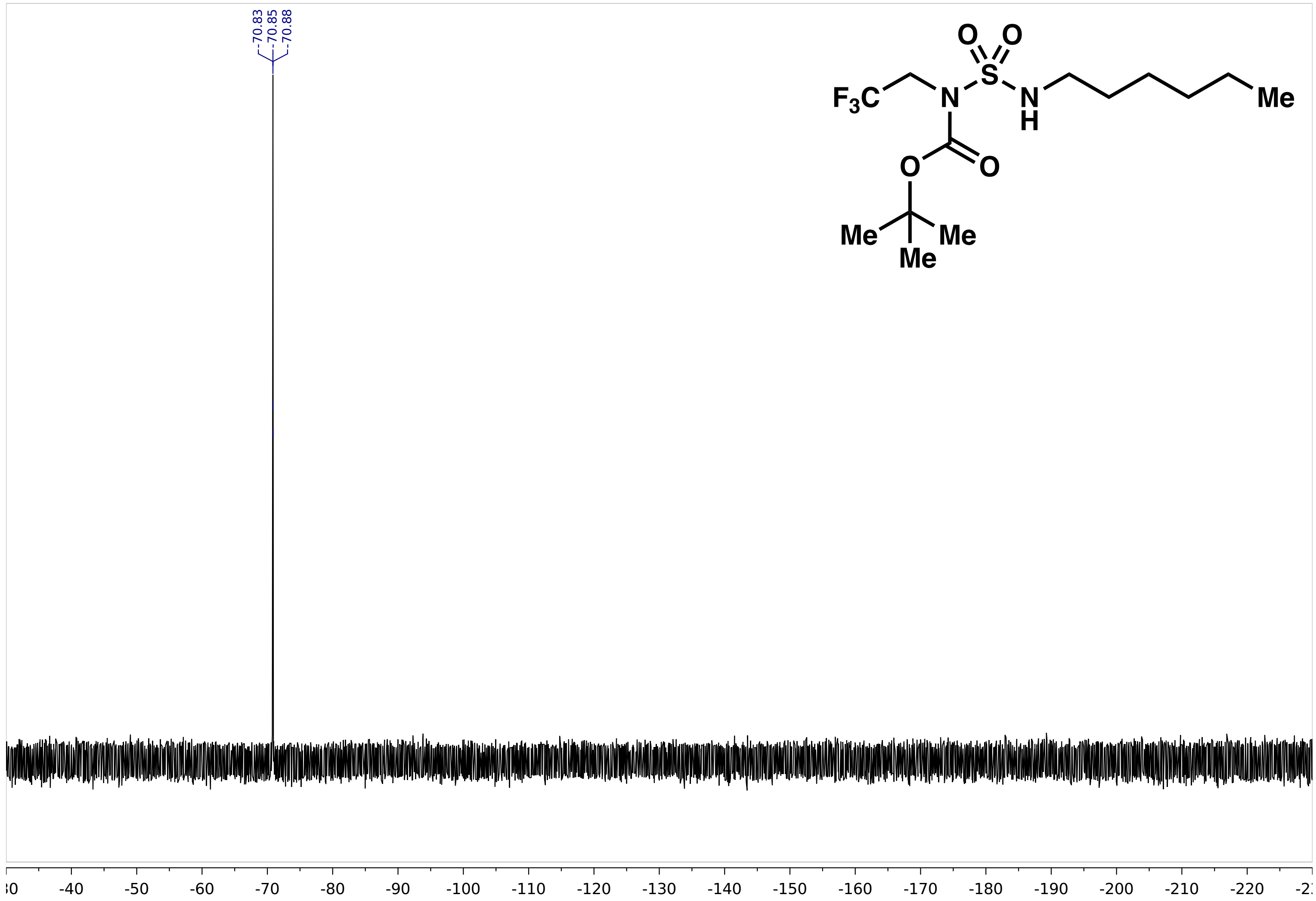

^{19}F NMR (376 MHz, $CDCl_3$) for *N*-*tert*-butoxycarbonyl-*N*'-hexyl-*N*-(2,2,2-trifluoro)ethylsulfamide (**S7b**)

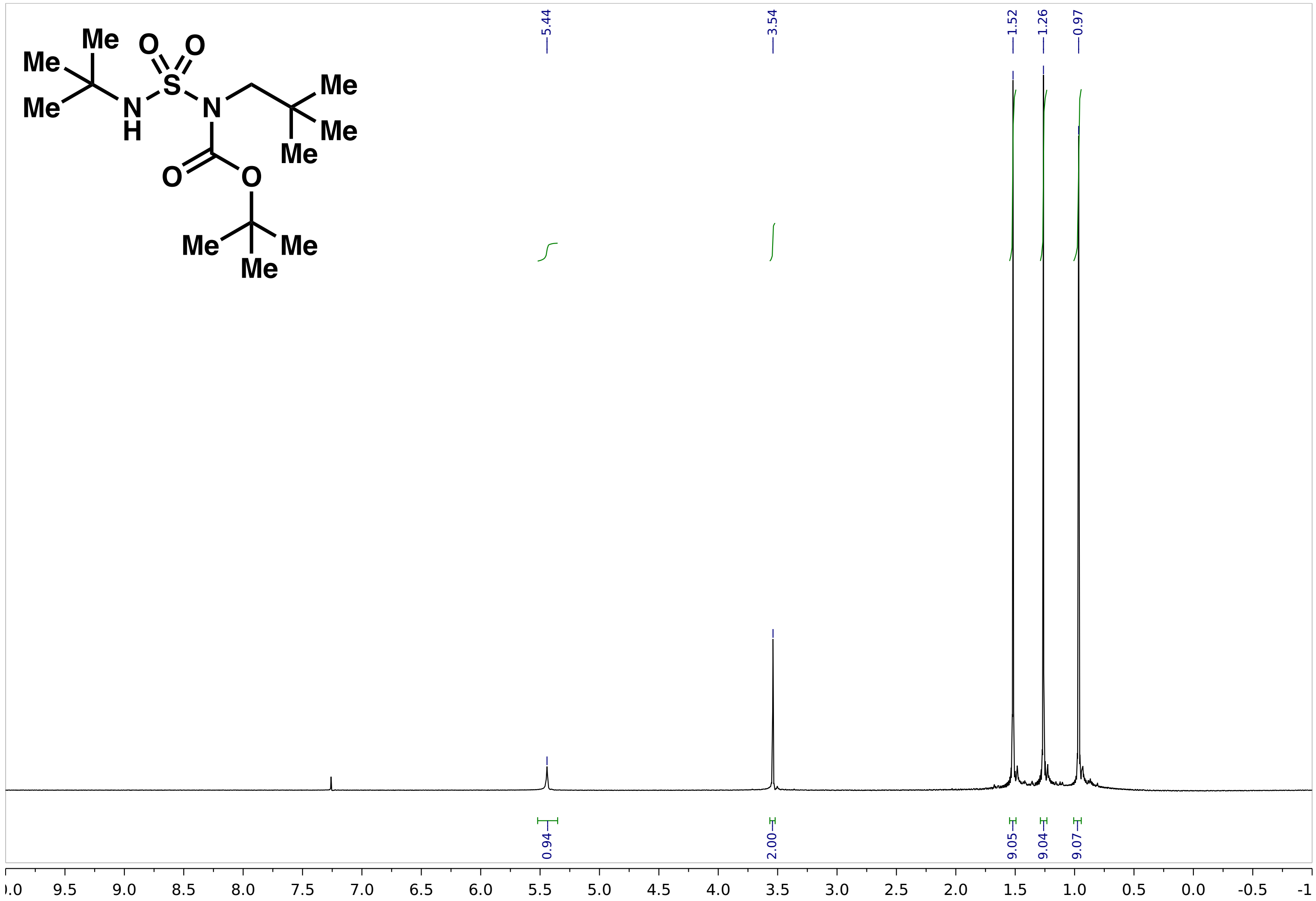

^{1}H NMR (400 MHz, $CDCl_3$) for *N*-*tert*-butoxycarbonyl-*N'*-*tert*-butyl-*N*-neopentylsulfamide (**S8a**)

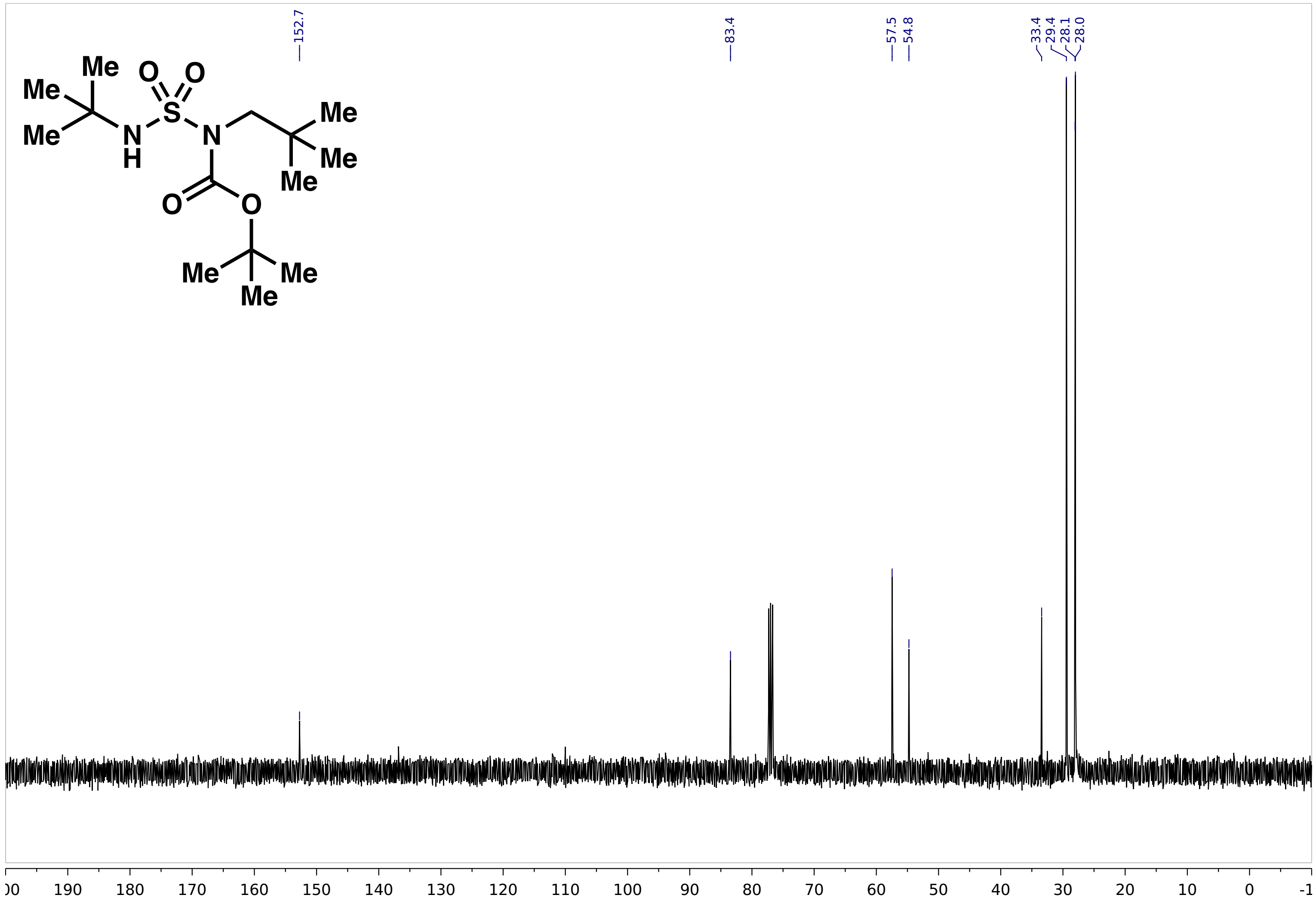

^{13}C NMR (101 MHz, $CDCl_3$) for *N*-*tert*-butoxycarbonyl-*N'*-*tert*-butyl-*N*-neopentylsulfamide (**S8a**)

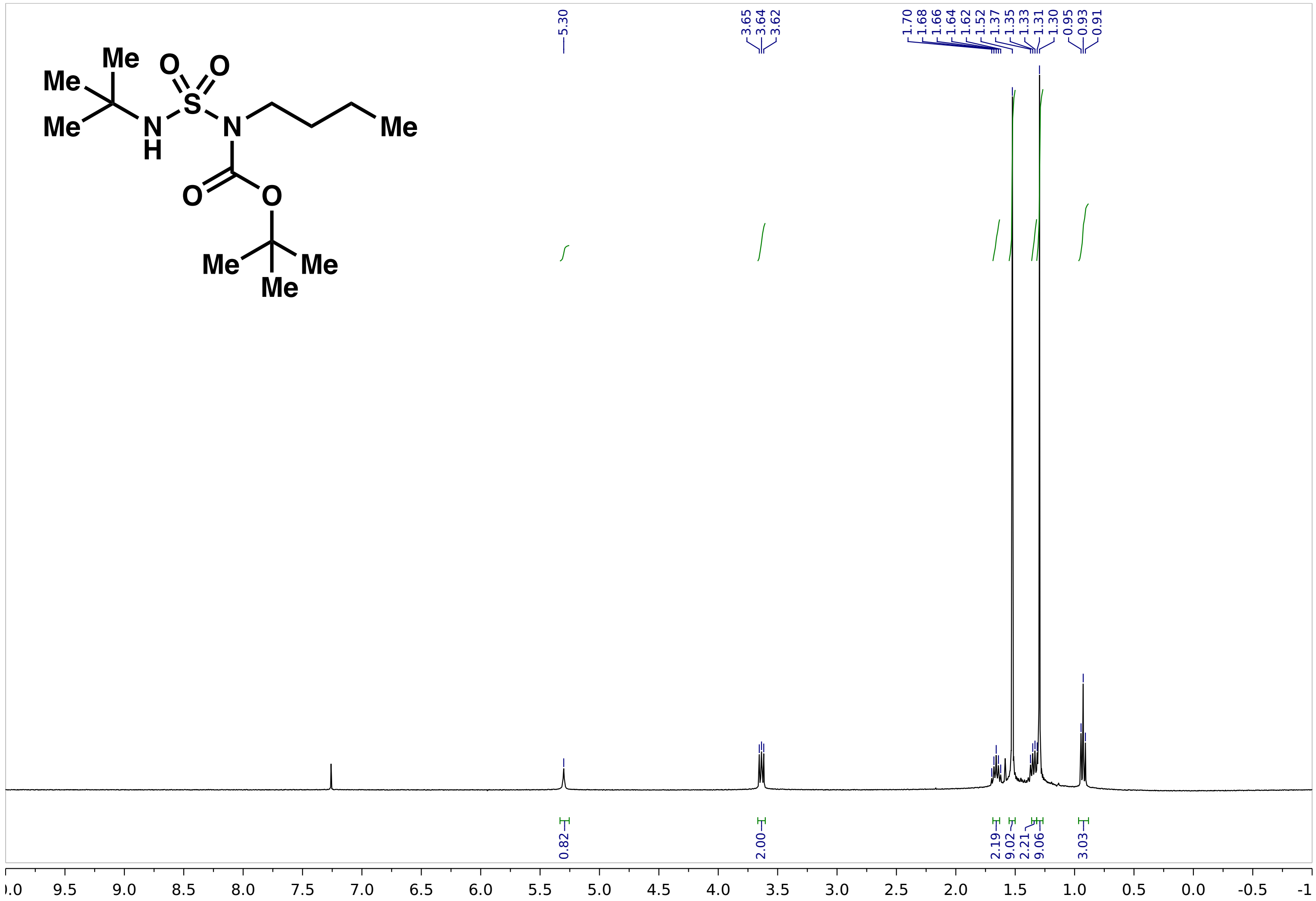

^{1}H NMR (400 MHz, $CDCl_3$) for *N*-*tert*-butoxycarbonyl-*N*-butyl-*N'*-*tert*-butylsulfamide (**S8b**)

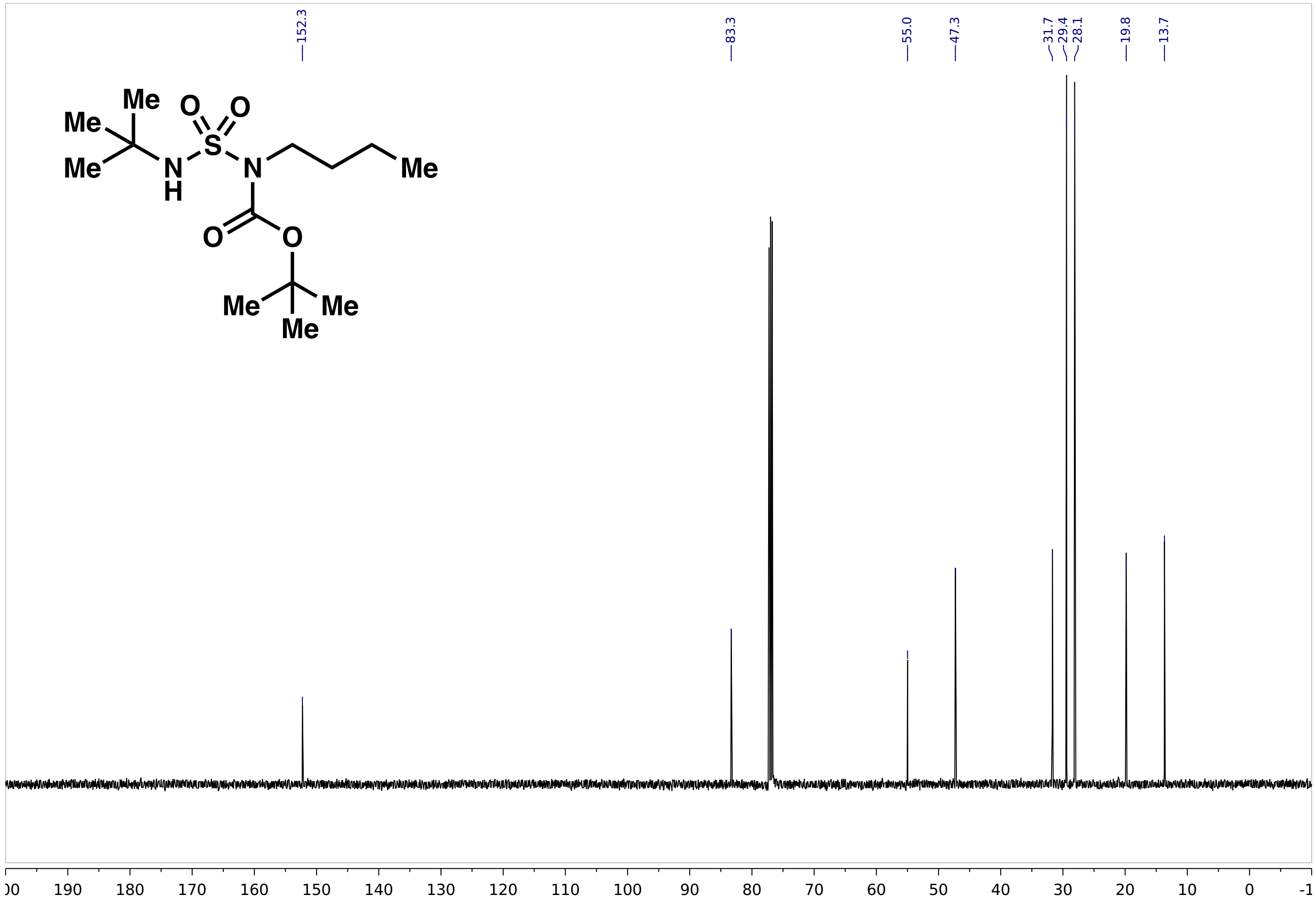

^{13}C NMR (126 MHz, $CDCl_3$) for *N*-*tert*-butoxycarbonyl-*N*-butyl-*N'*-*tert*-butylsulfamide (**S8b**)

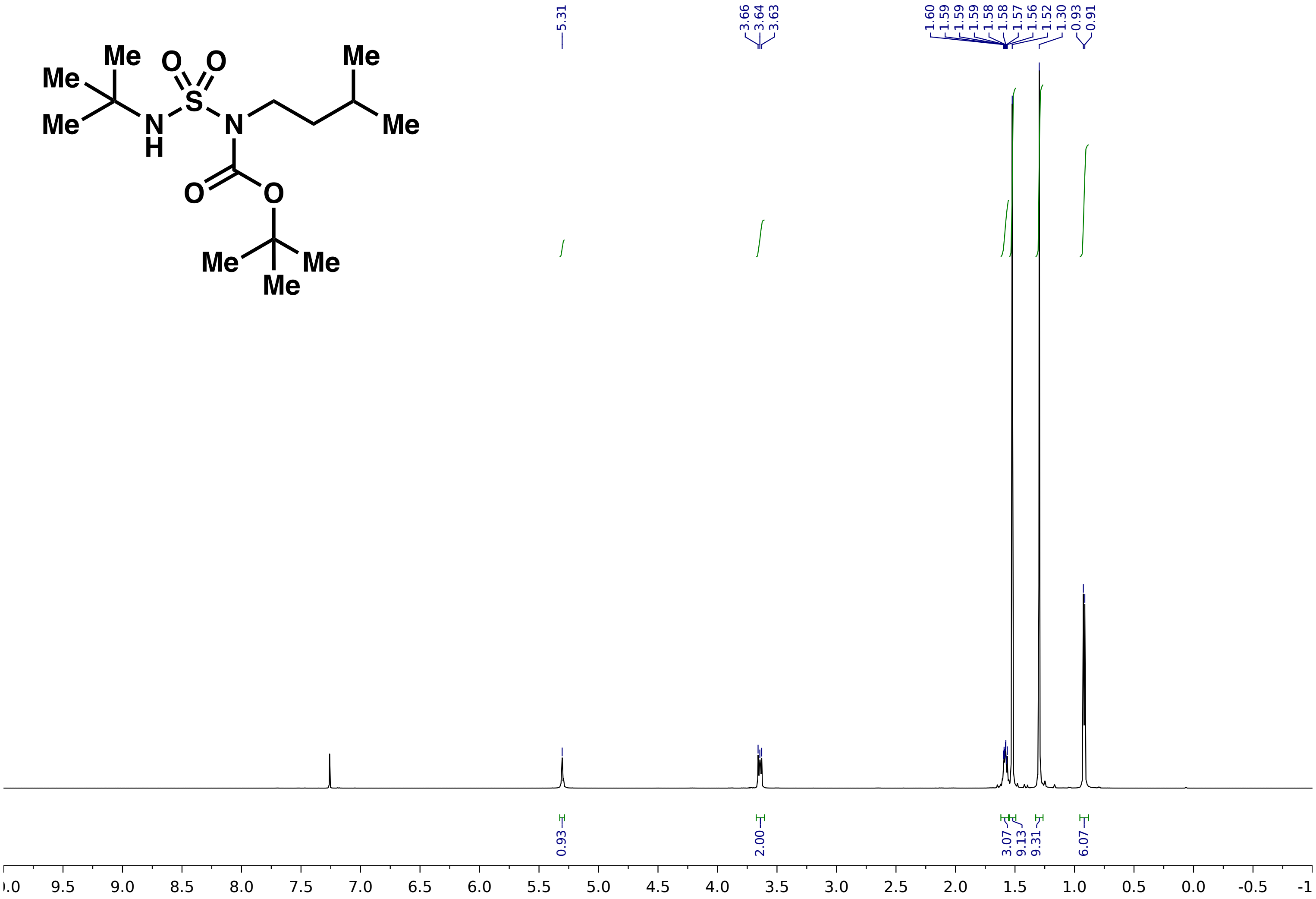

^{1}H NMR (400 MHz, C_6D_6) for *N*-*tert*-butoxycarbonyl-*N'*-*tert*-butyl-*N*-isopentylsulfamide (**S8c**)

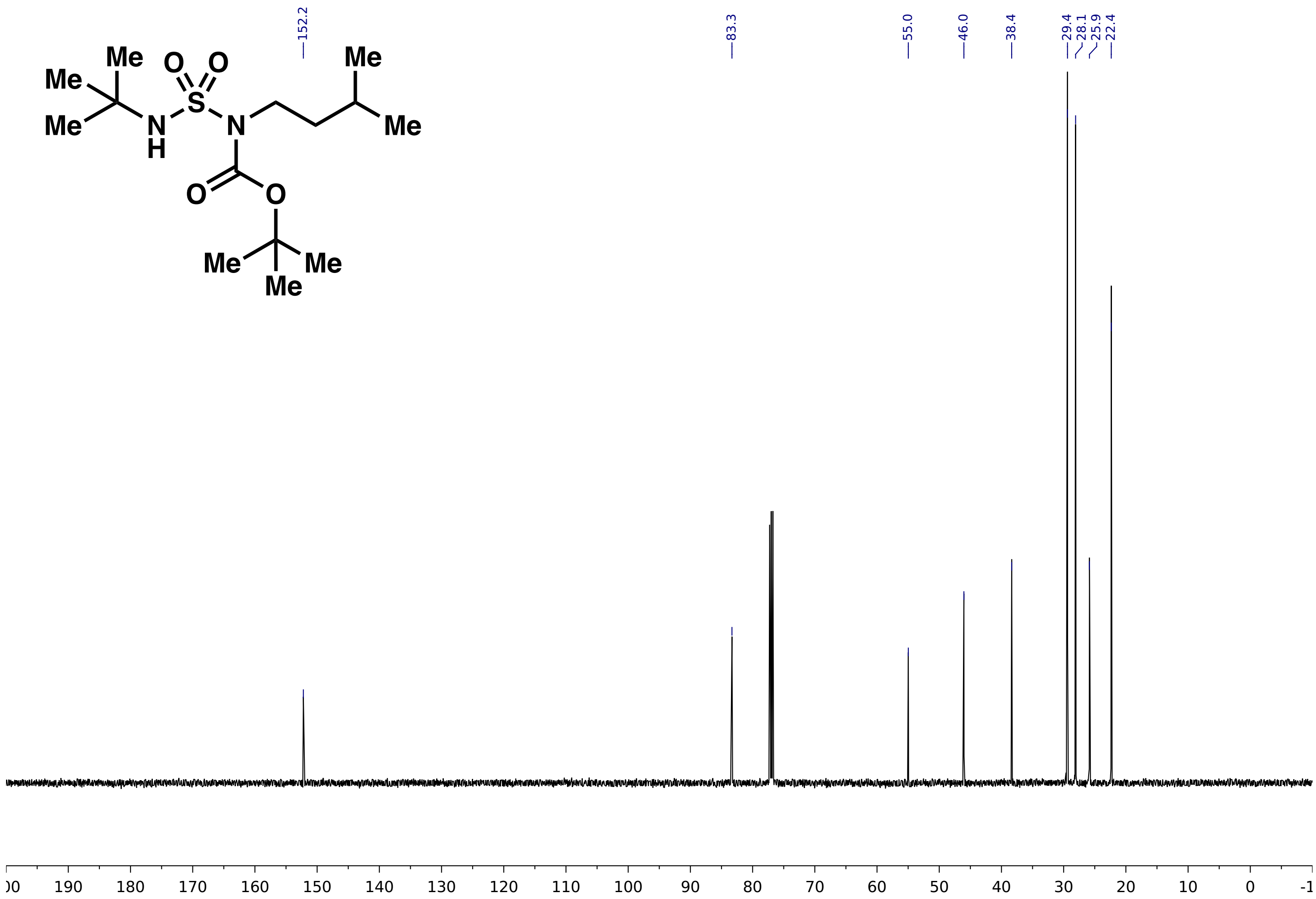

^{13}C NMR (126 MHz, $CDCl_3$) for *N*-*tert*-butoxycarbonyl-*N'*-*tert*-butyl-*N*-isopentylsulfamide (**S8c**)

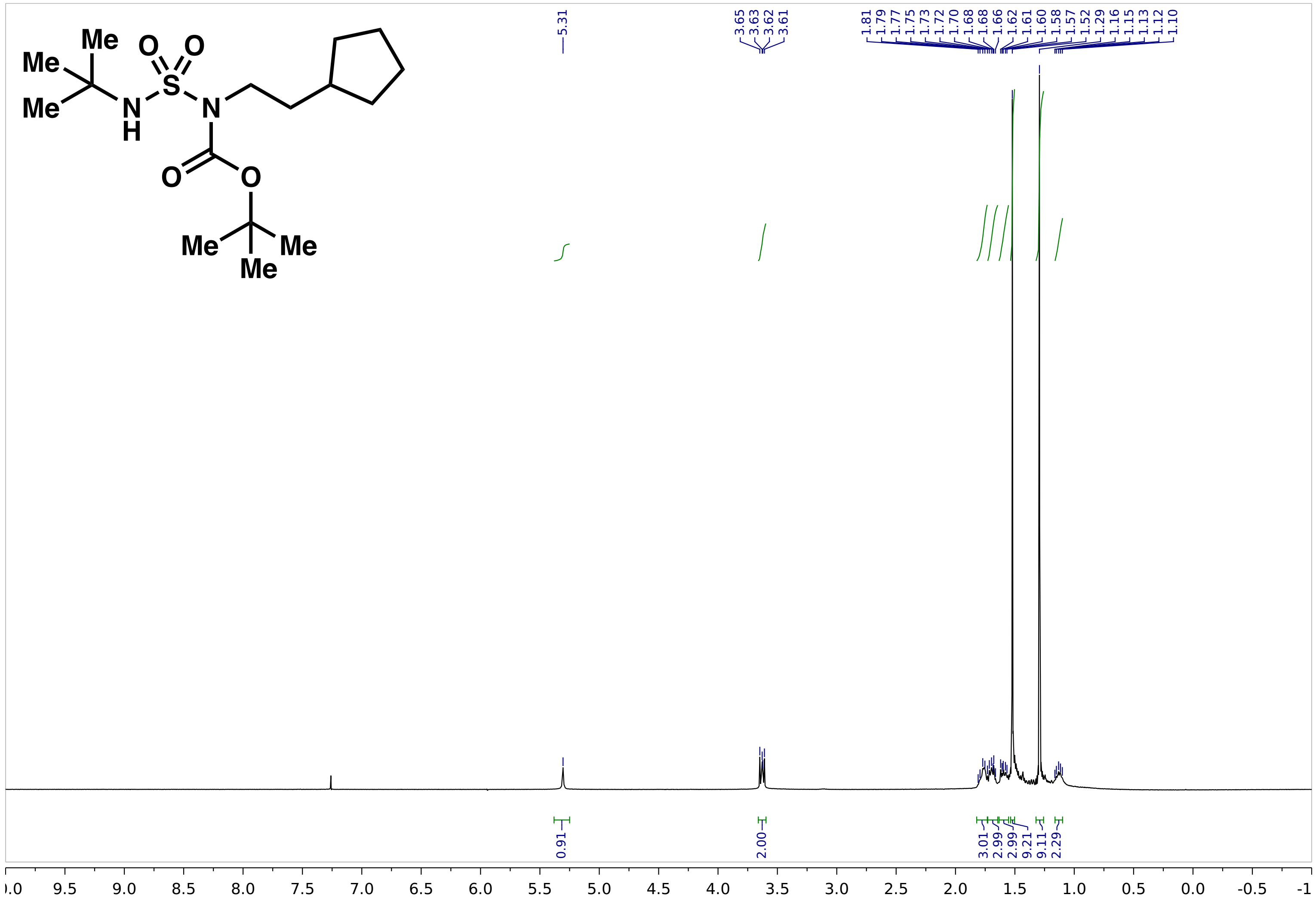

^{1}H NMR (400 MHz, $CDCl_3$) for *N*-*tert*-butoxycarbonyl-*N’*-*tert*-butyl-*N*-(2-(cyclopentyl)ethyl)sulfamide (**S8d**)

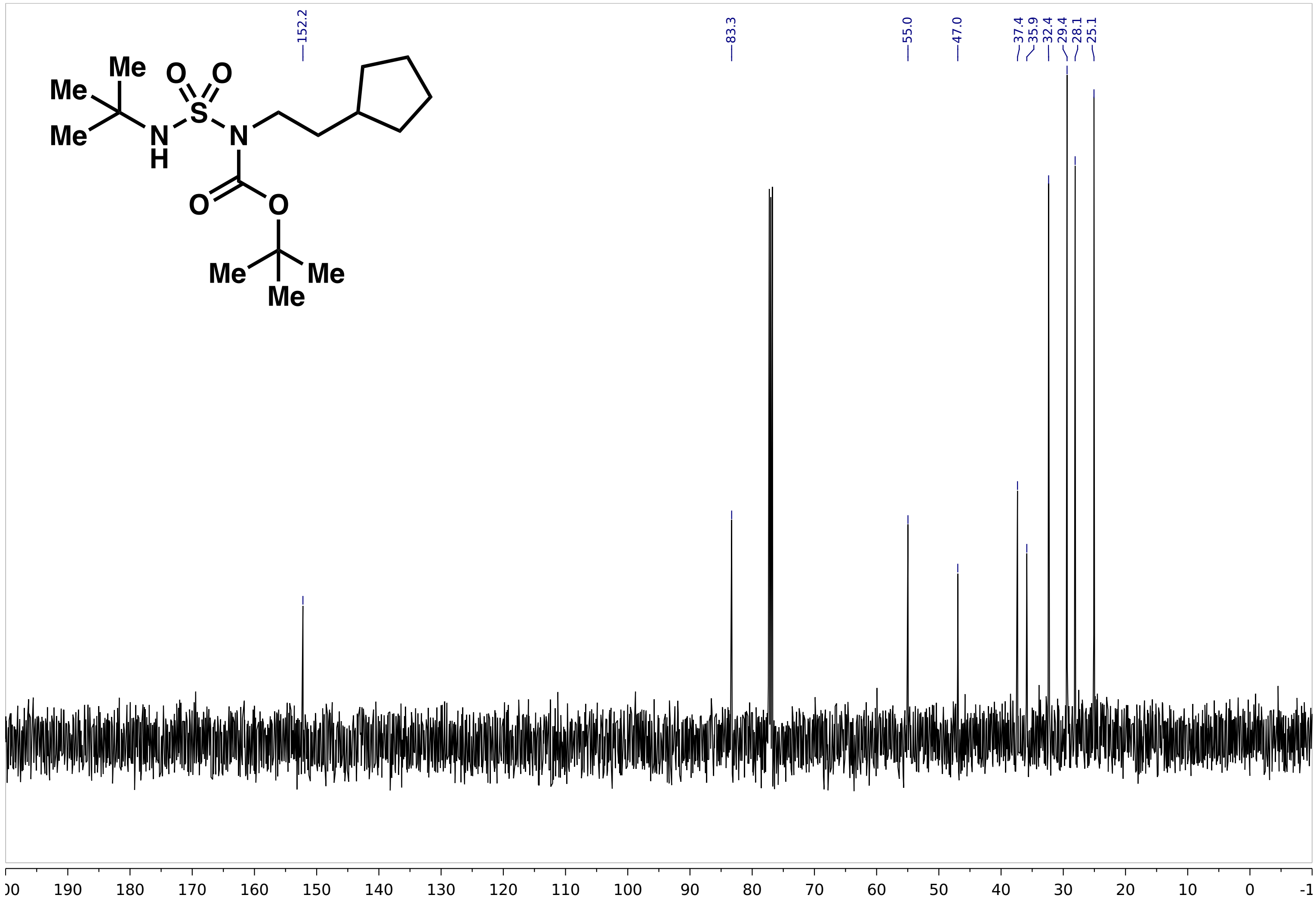

^{13}C NMR (126 MHz, $CDCl_3$) for *N*-*tert*-butoxycarbonyl-*N'*-*tert*-butyl-*N*-(2-(cyclopentyl)ethyl)sulfamide (**S8d**)

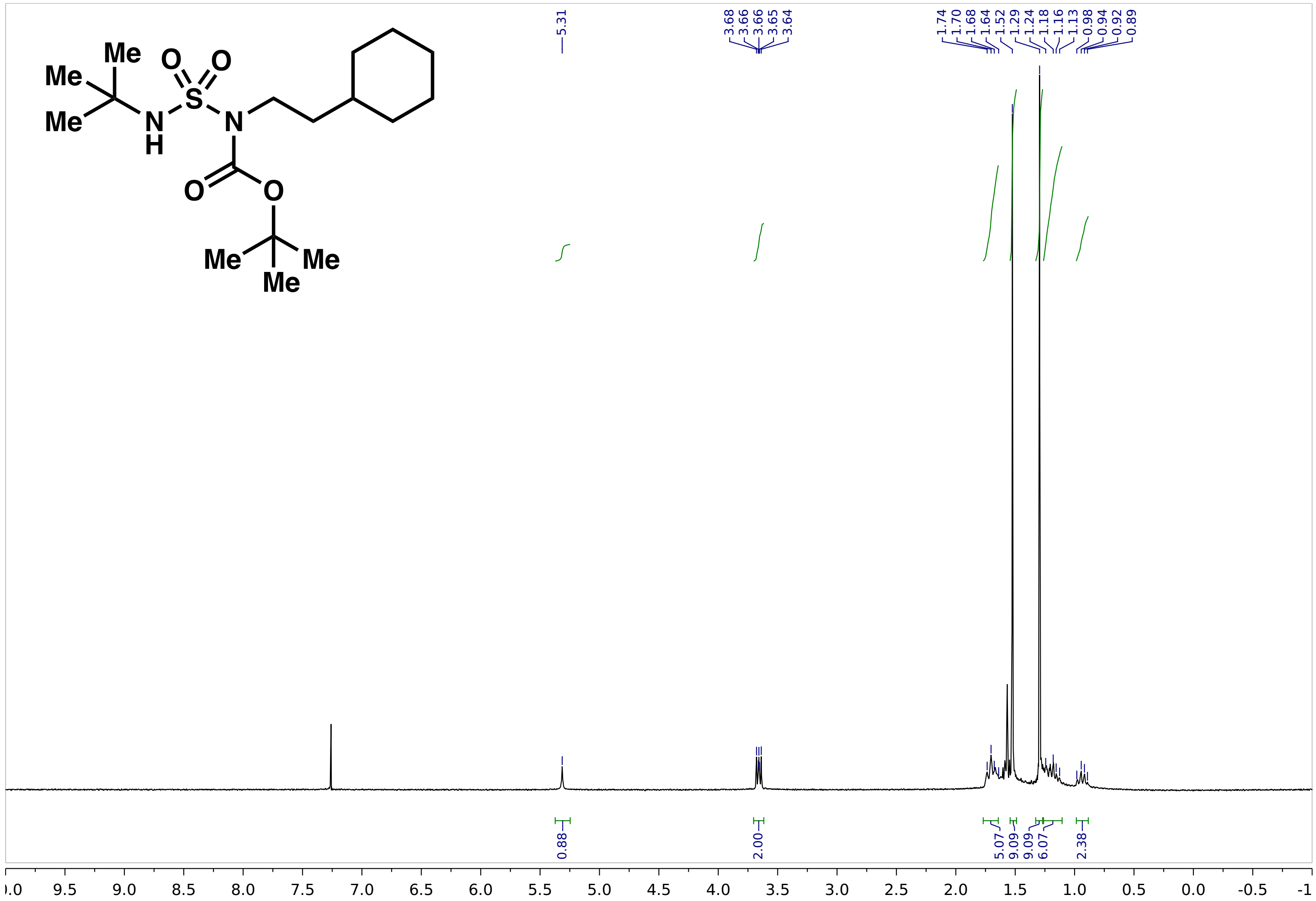

^{1}H NMR (400 MHz, CDCl3) for *N*-*tert*-butoxycarbonyl-*N'*-*tert*-butyl-*N*-(2-(cyclohexyl)ethyl)sulfamide (**S8e**)

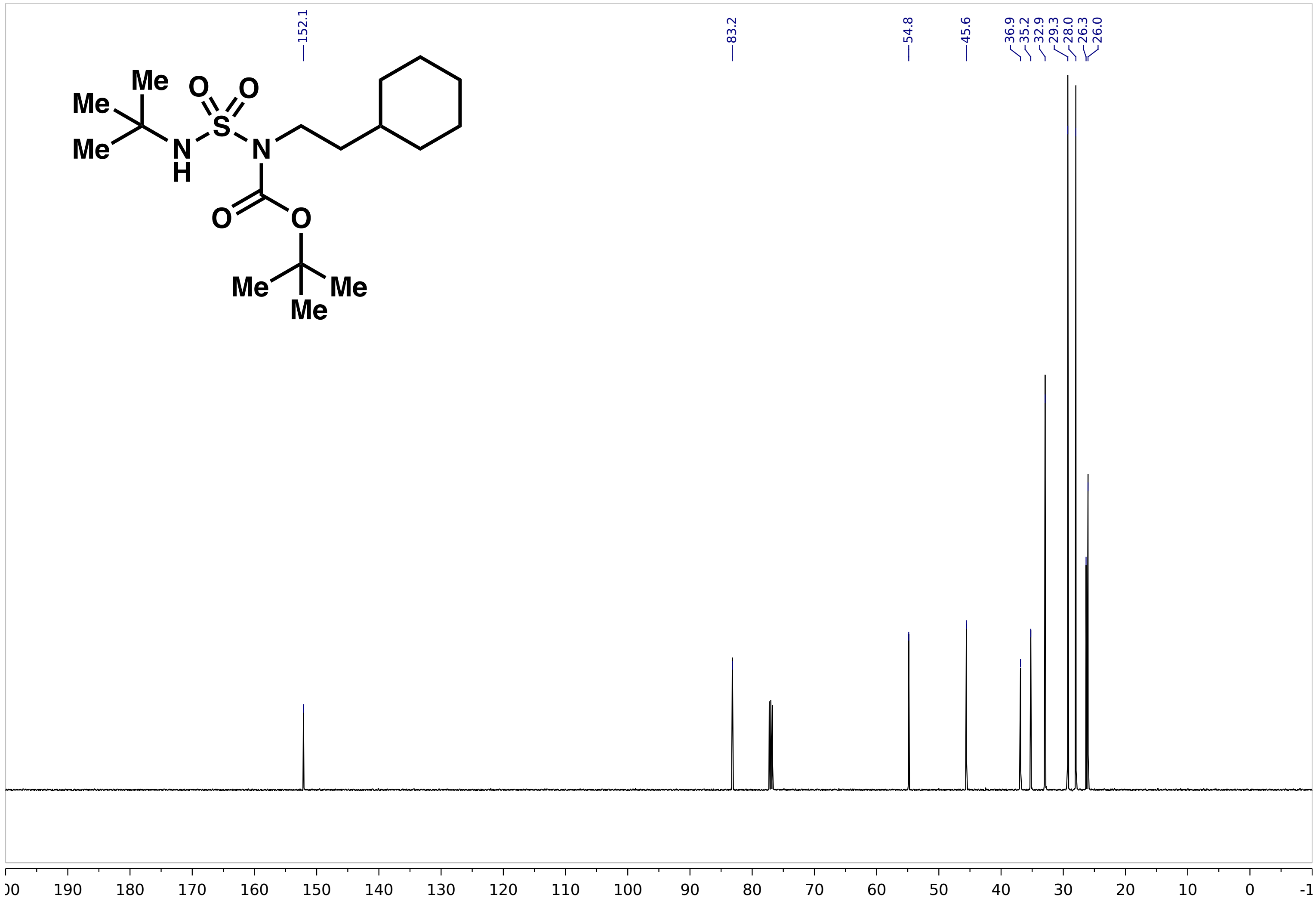

^{13}C NMR (126 MHz, $CDCl_3$) for *N*-*tert*-butoxycarbonyl-*N'*-*tert*-butyl-*N*-(2-(cyclohexyl)ethyl)sulfamide (**S8e**)

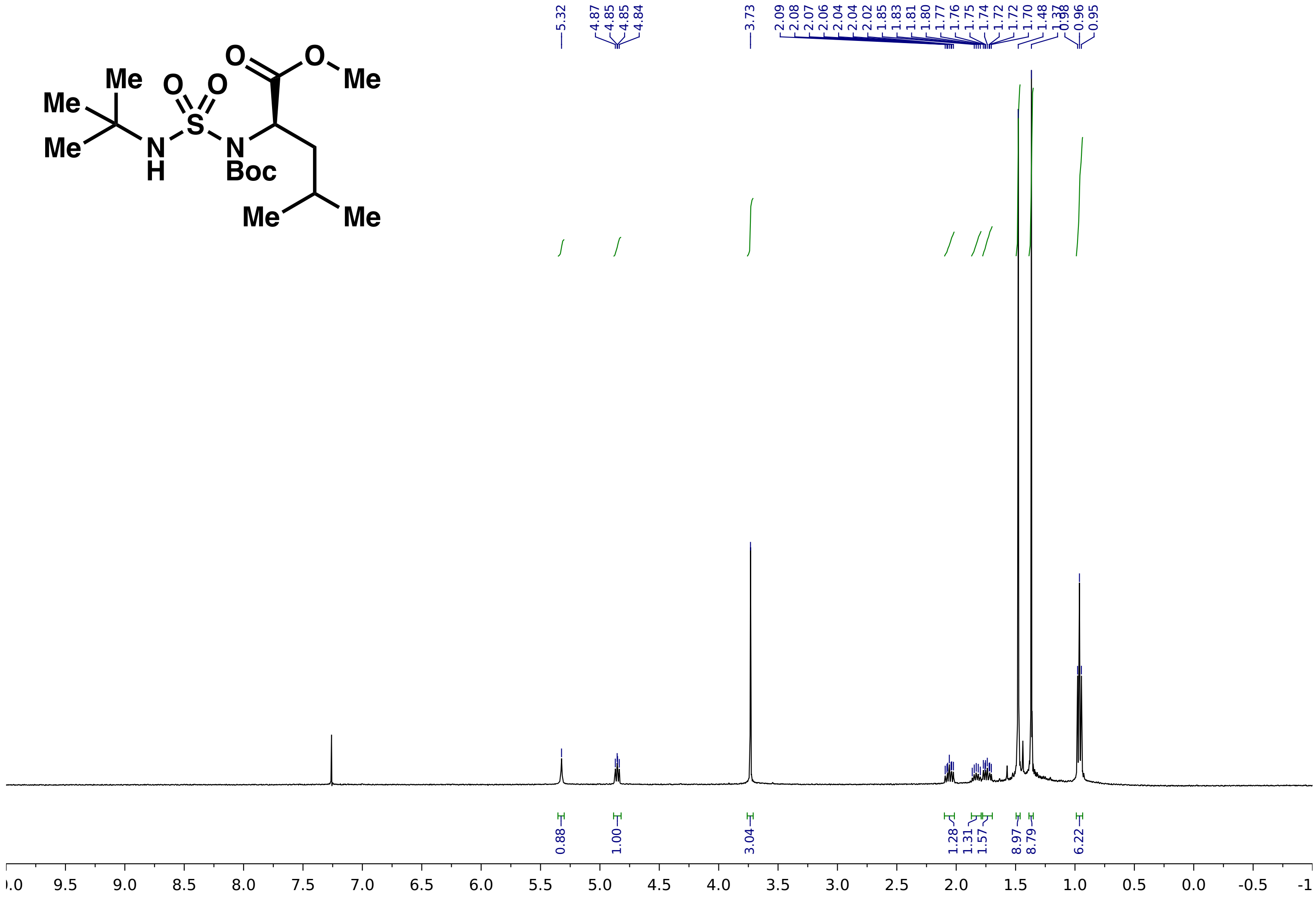

^{1}H NMR (400 MHz, $CDCl_3$) for *N*-*tert*-butoxycarbonyl-*N'*-*tert*-butyl-*N*-((2*R*)-methyl 4-methylpentano-2-yl)sulfamide (**S8f**)

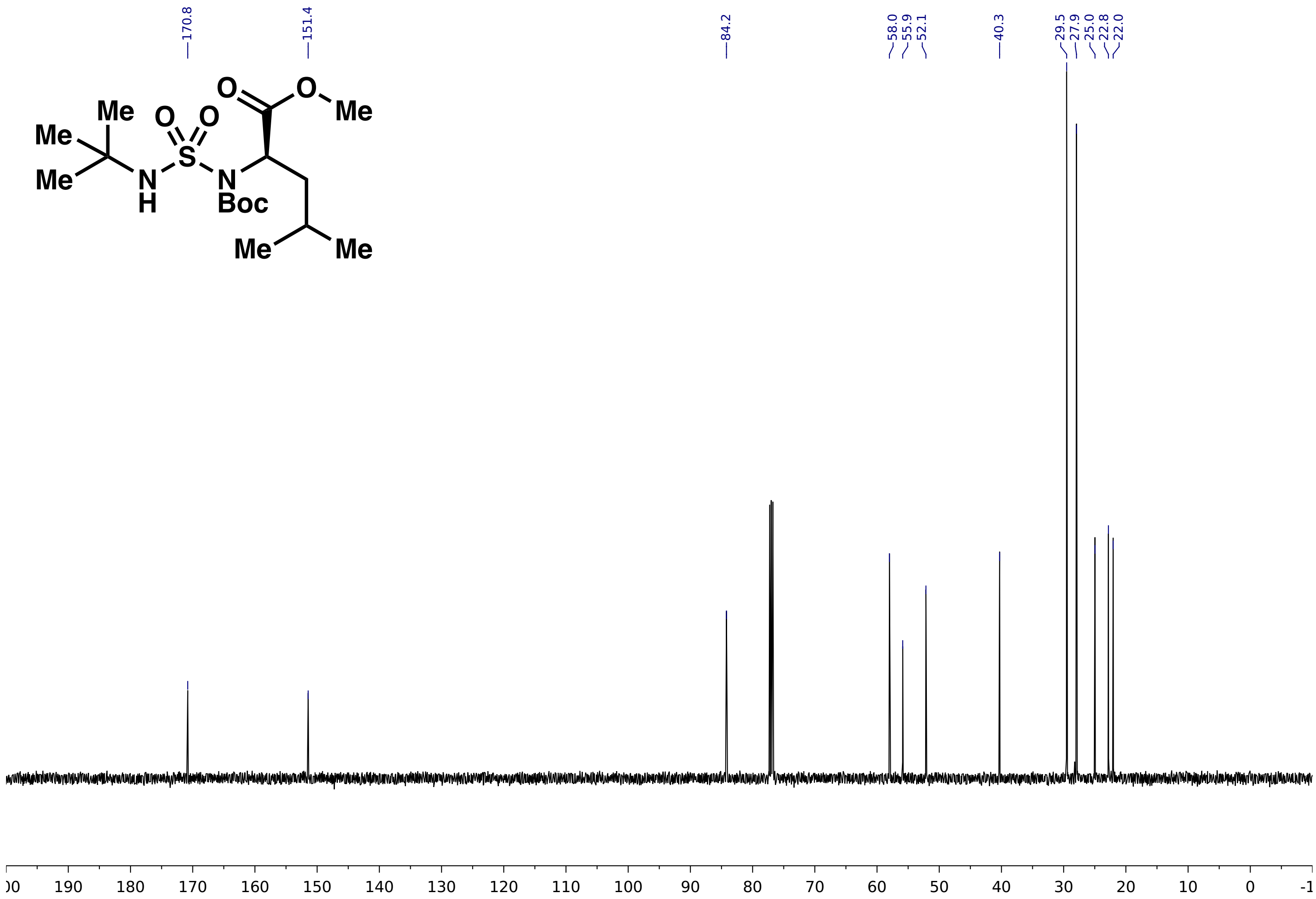

^{13}C NMR (126 MHz, $CDCl_3$) for *N*-*tert*-butoxycarbonyl-*N'*-*tert*-butyl-*N*-((*2R*)-methyl 4-methylpentano-2-yl)sulfamide (**S8f**)

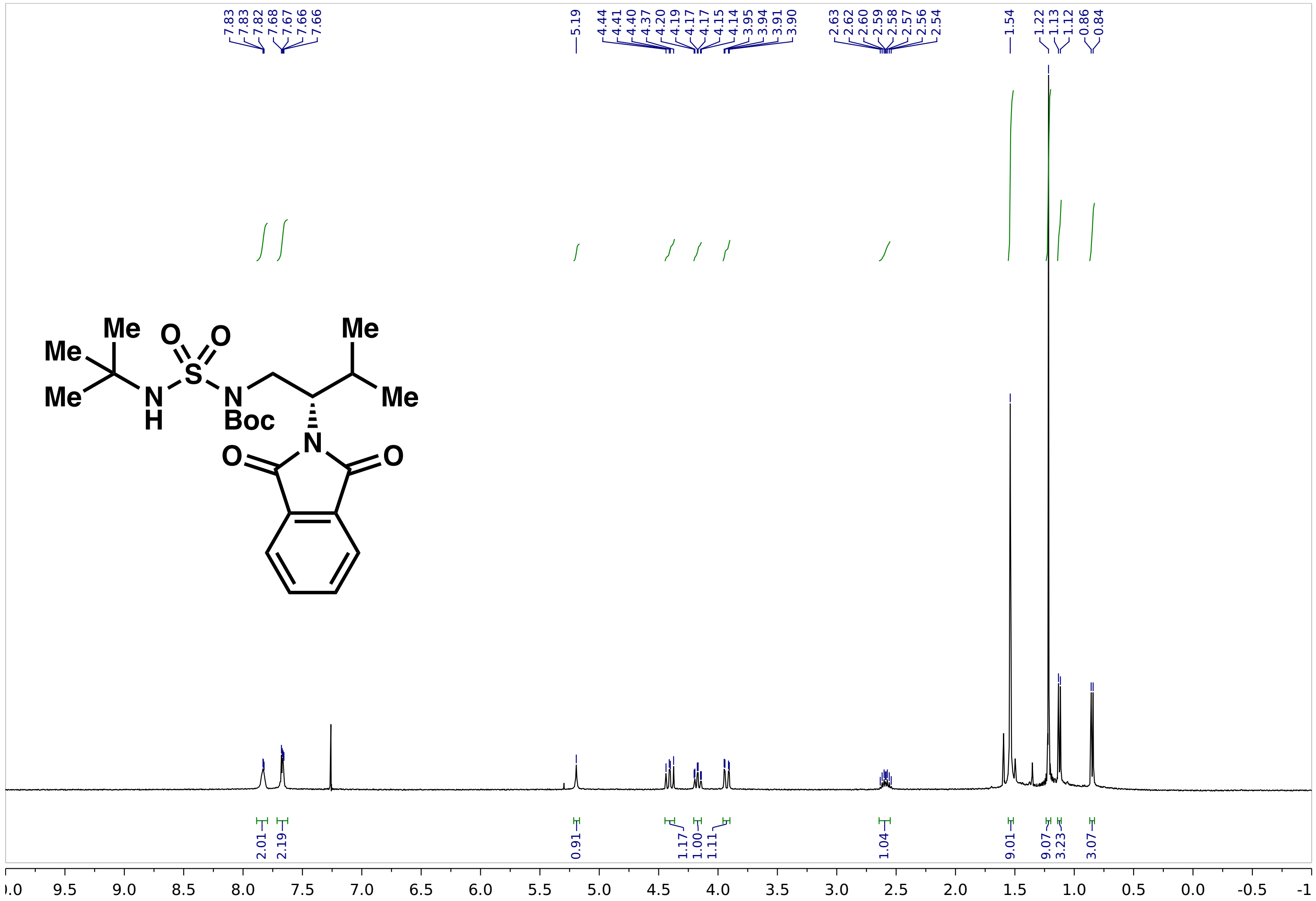

^{1}H NMR (400 MHz, $CDCl_3$) for *N-tert*-butoxycarbonyl-*N*'-*tert*-butyl-*N*-((*S*)-2-(1,3-dioxoisoindolin-2-yl)-3-methylbutyl)sulfamide (**S8g**)

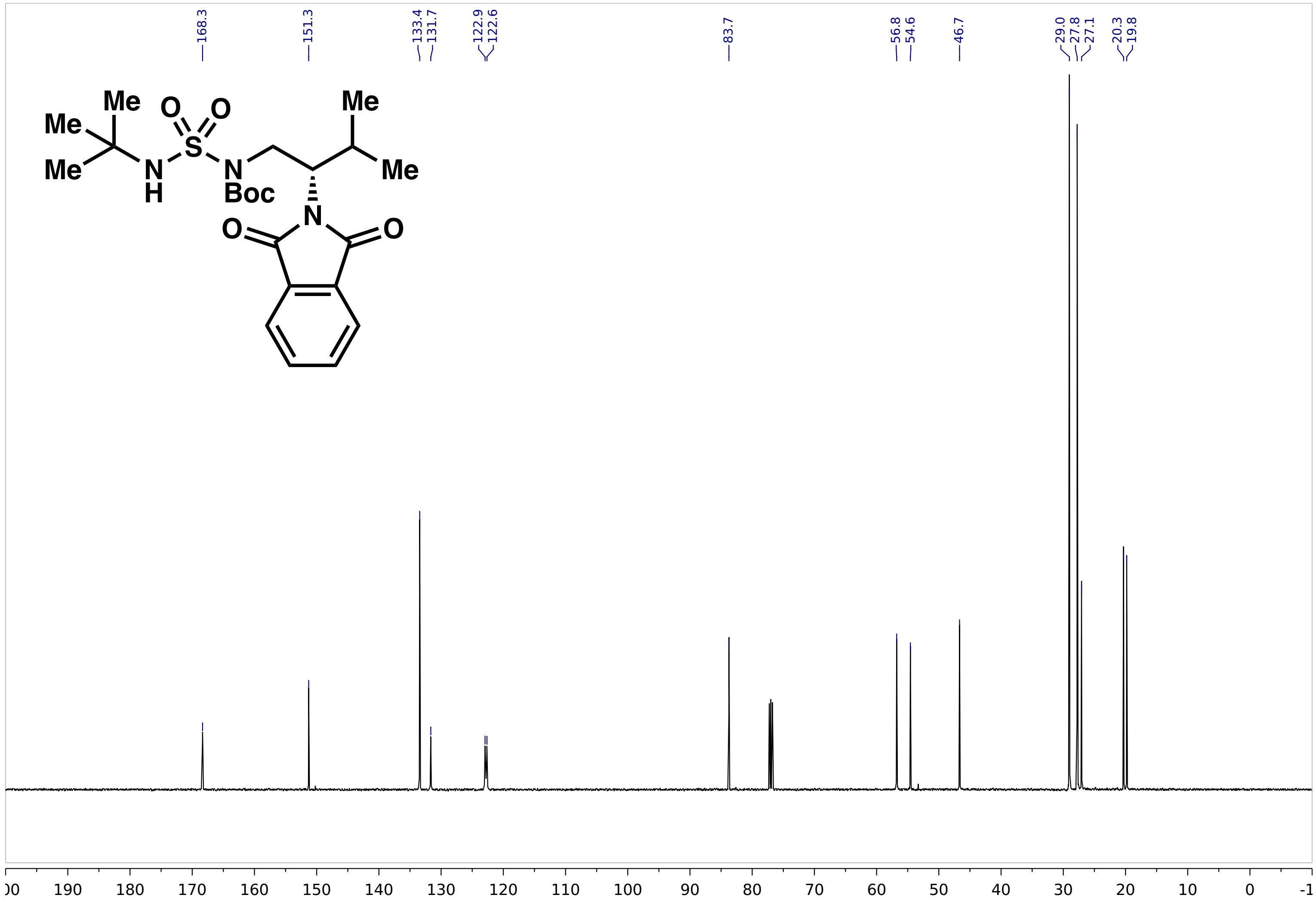

^{13}C NMR (126 MHz, $CDCl_3$) for *N*-*tert*-butoxycarbonyl-*N*'-*tert*-butyl-*N*-((*S*)-2-(1,3-dioxoisoindolin-2-yl)-3-methylbutyl)sulfamide (**S8g**)

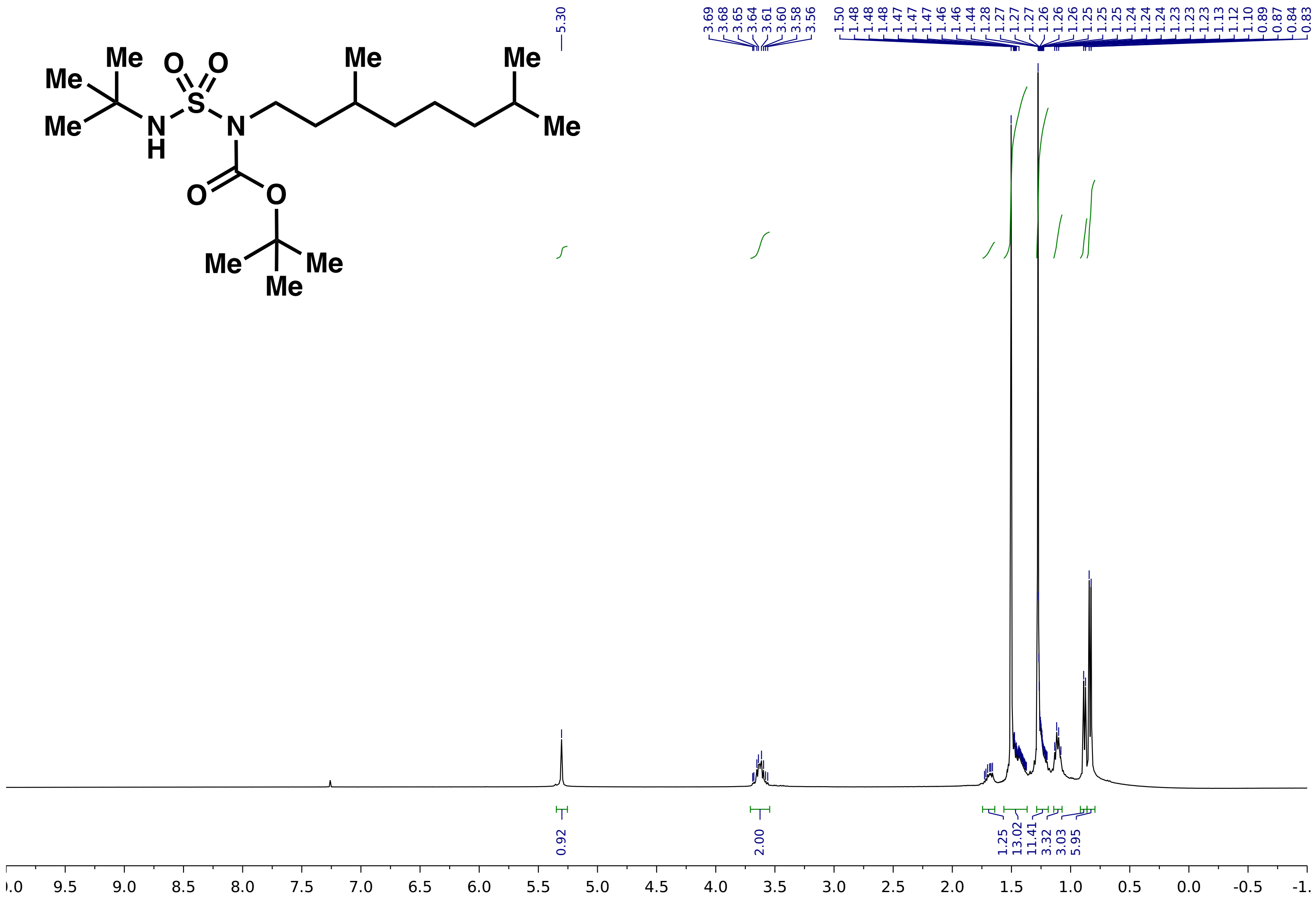

^{1}H NMR (400 MHz, $CDCl_3$) for *N*-*tert*-butoxycarbonyl-*N'*-*tert*-butyl-*N*-(3,7-dimethyloct-1-yl)sulfamide (**S8h**)

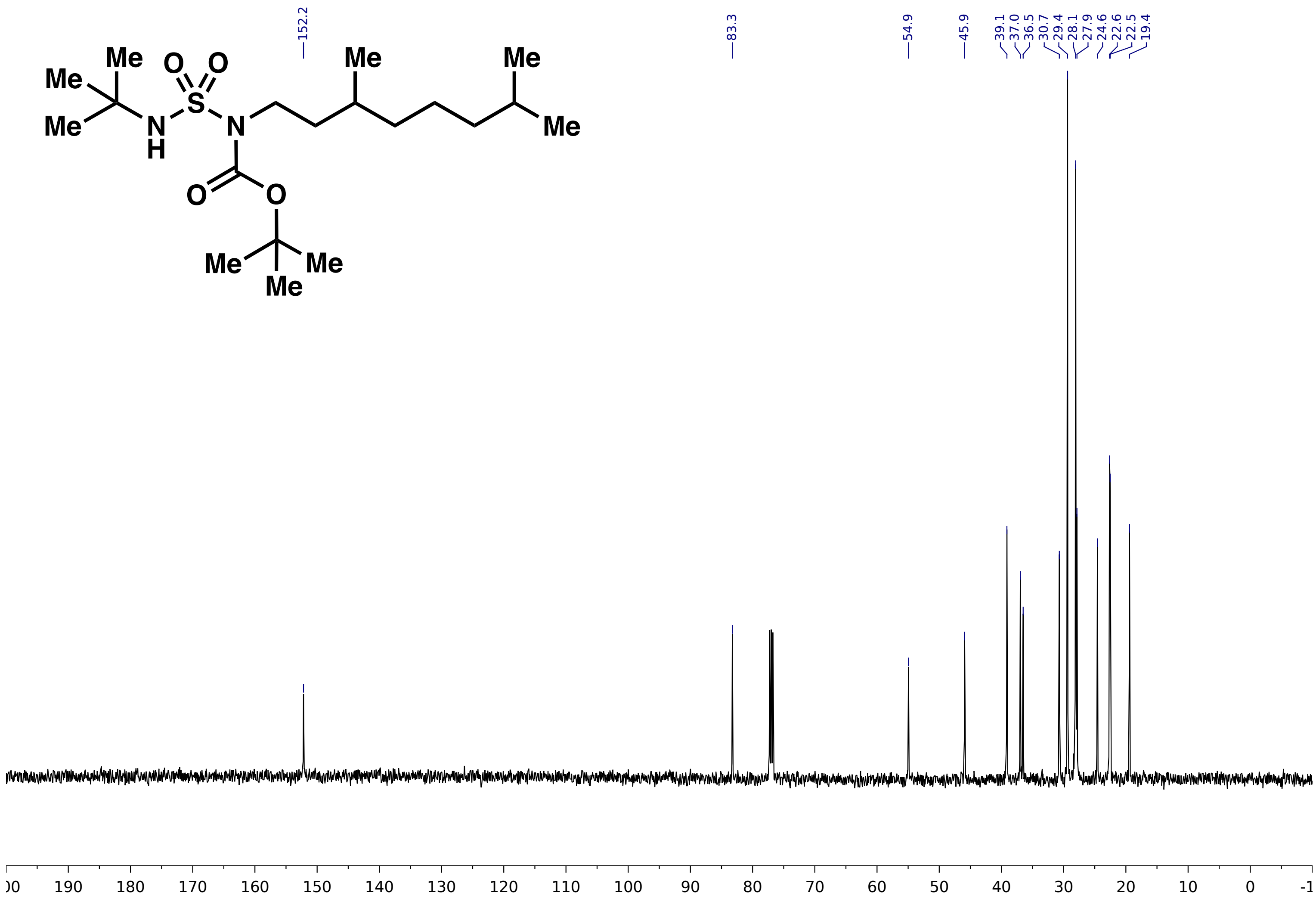

^{13}C NMR (126 MHz, $CDCl_3$) for *N*-*tert*-butoxycarbonyl-*N'*-*tert*-butyl-*N*-(3,7-dimethyloct-1-yl)sulfamide (**S8h**)

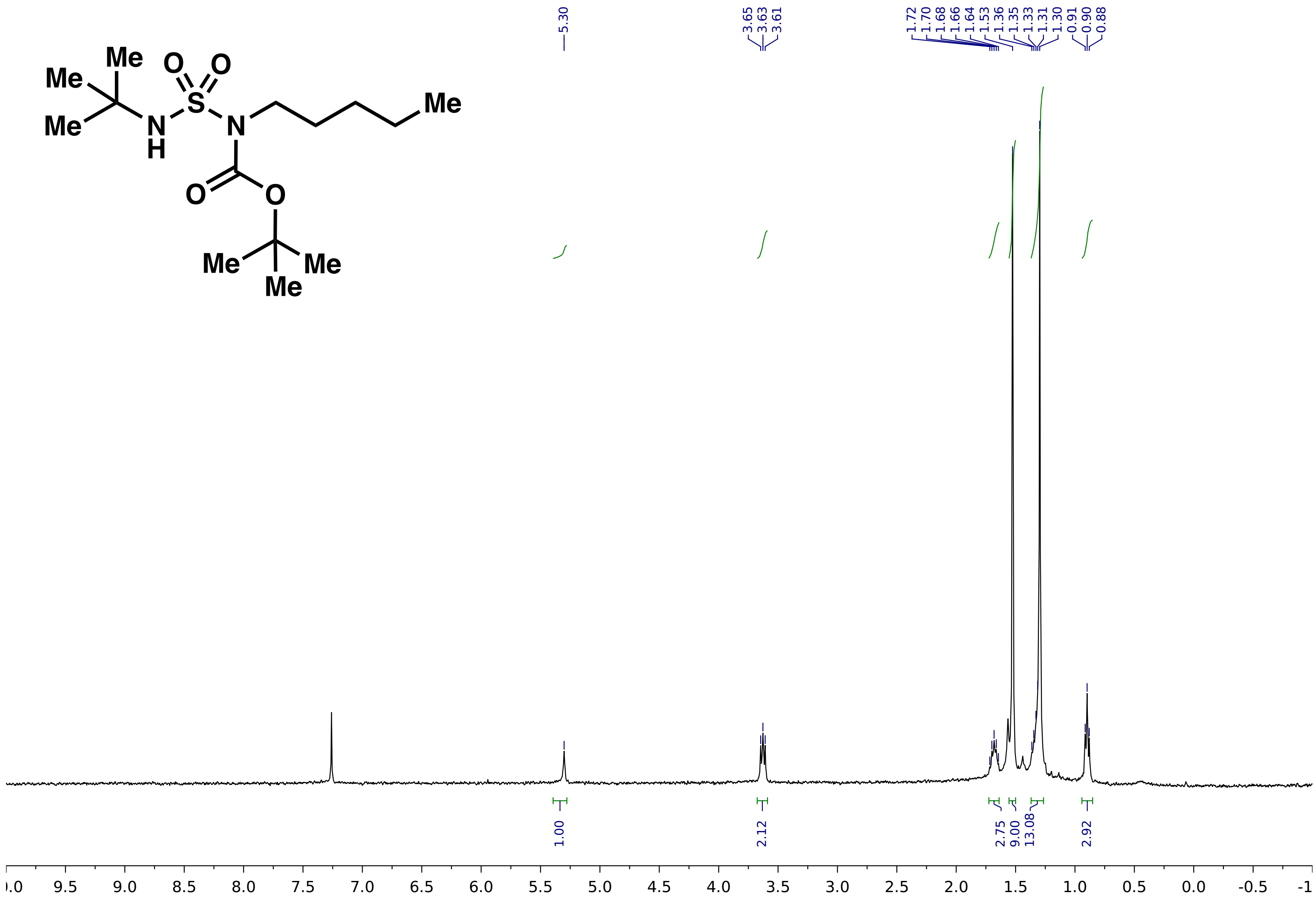

^{1}H NMR (400 MHz, $CDCl_3$) for *N*-*tert*-butoxycarbonyl-*N'*-*tert*-butyl-*N*-pentylsulfamide (**S8i**)

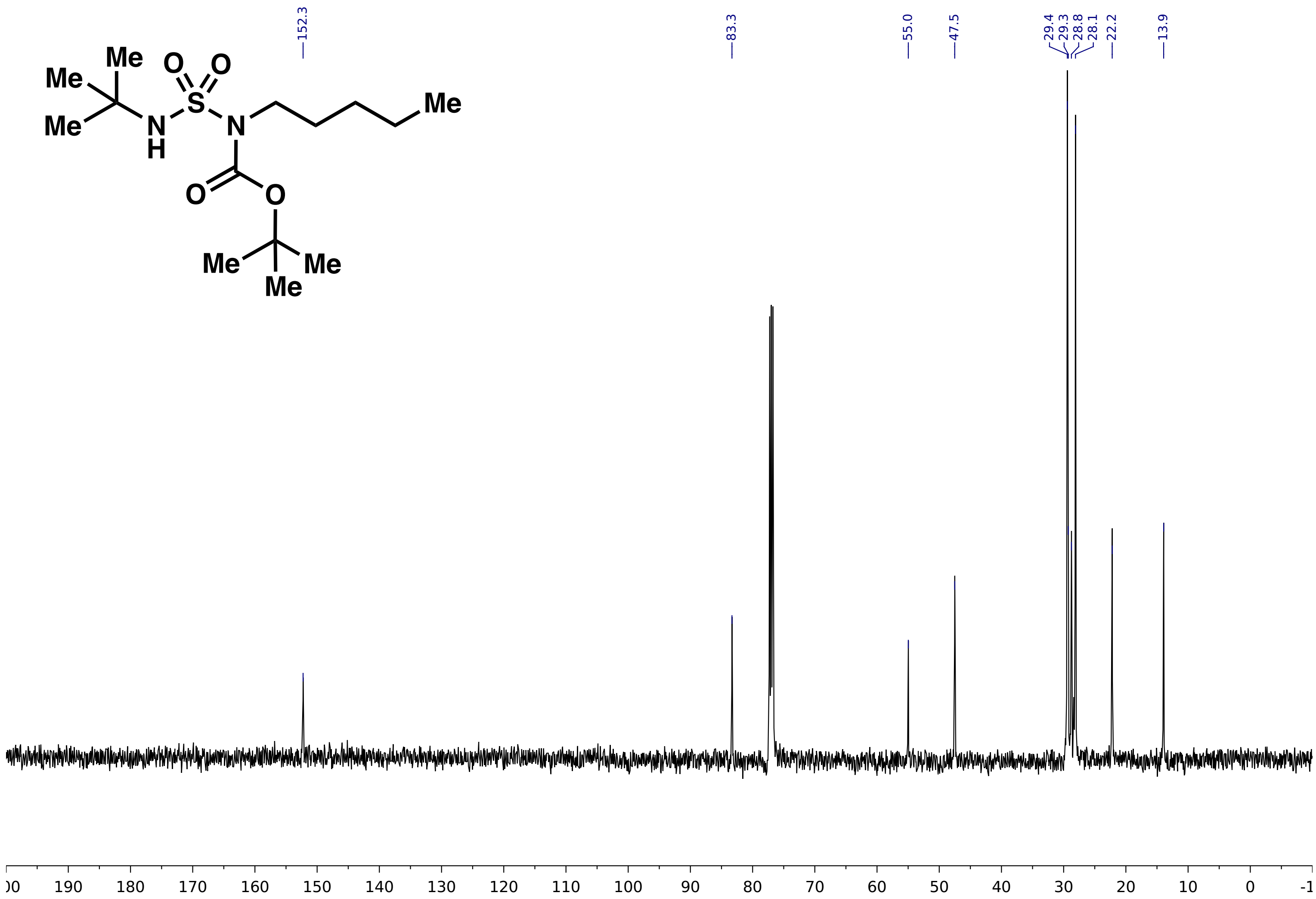

^{13}C NMR (126 MHz, $CDCl_3$) for *N*-*tert*-butoxycarbonyl-*N'*-*tert*-butyl-*N*-pentylsulfamide (**S8i**)

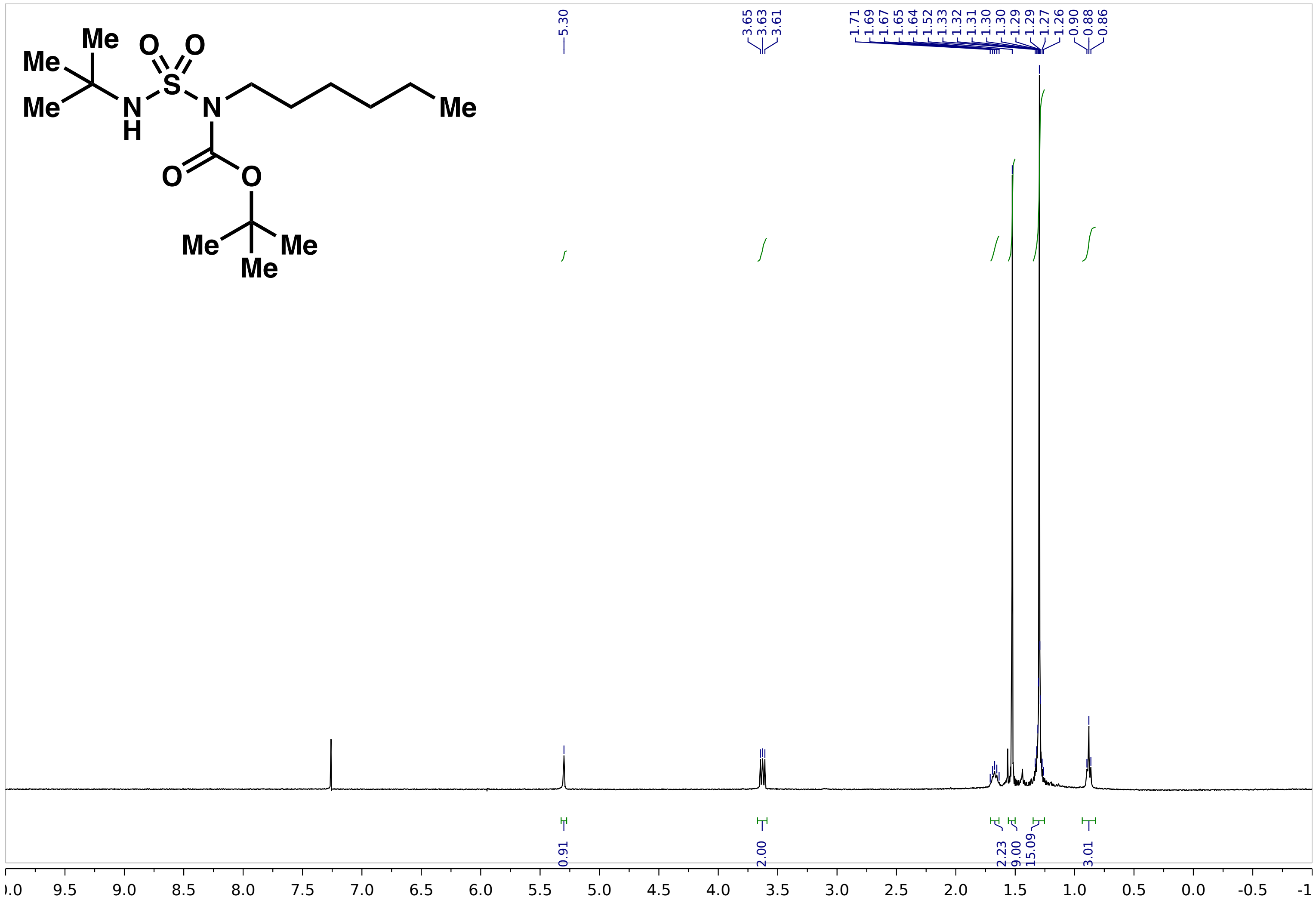

^{1}H NMR (400 MHz, $CDCl_3$) for *N*-*tert*-butoxycarbonyl-*N'*-*tert*-butyl-*N*-hexylsulfamide (**S8j**)

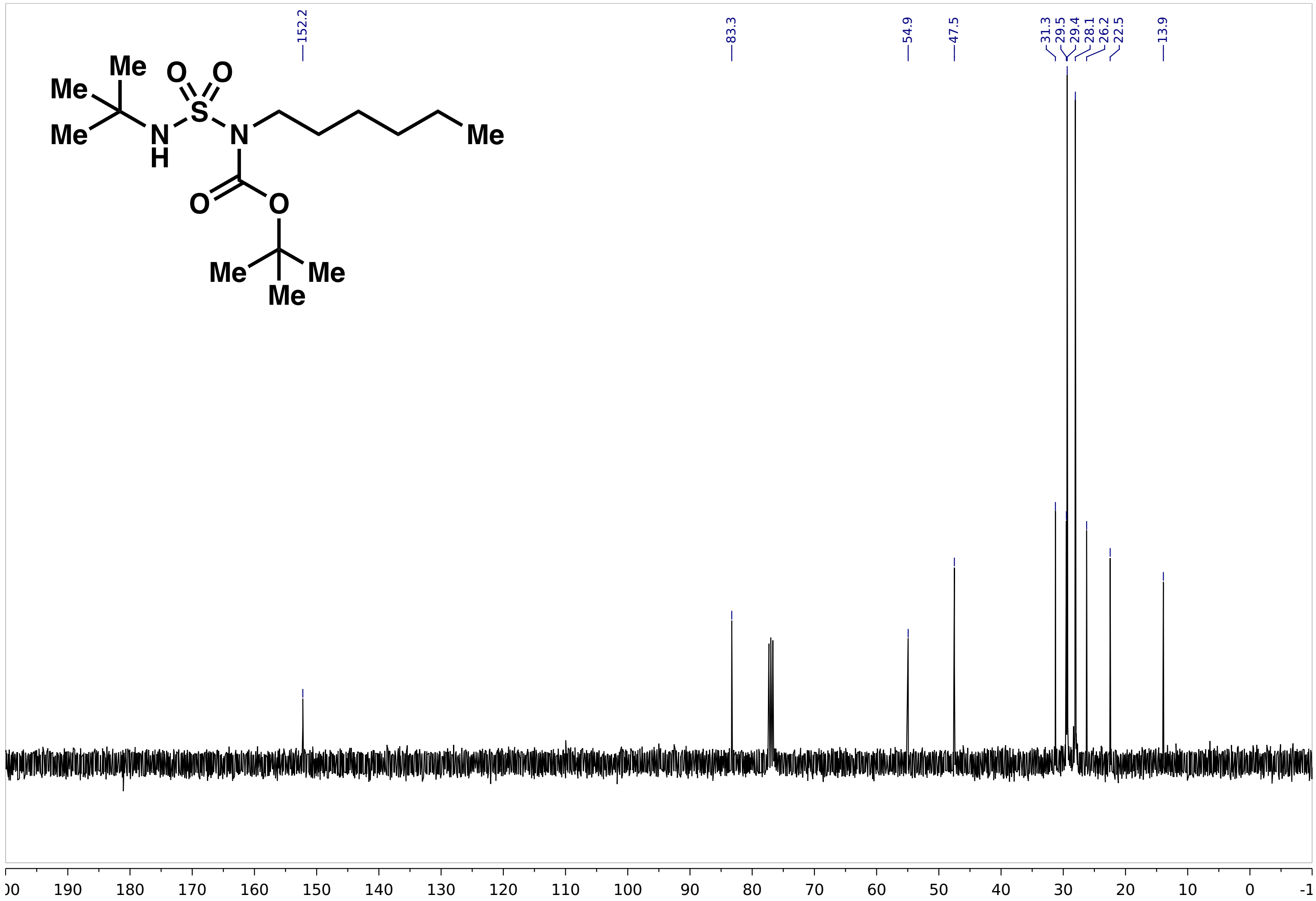

^{13}C NMR (101 MHz, $CDCl_3$) for *N*-*tert*-butoxycarbonyl-*N'*-*tert*-butyl-*N*-hexylsulfamide (**S8j**)

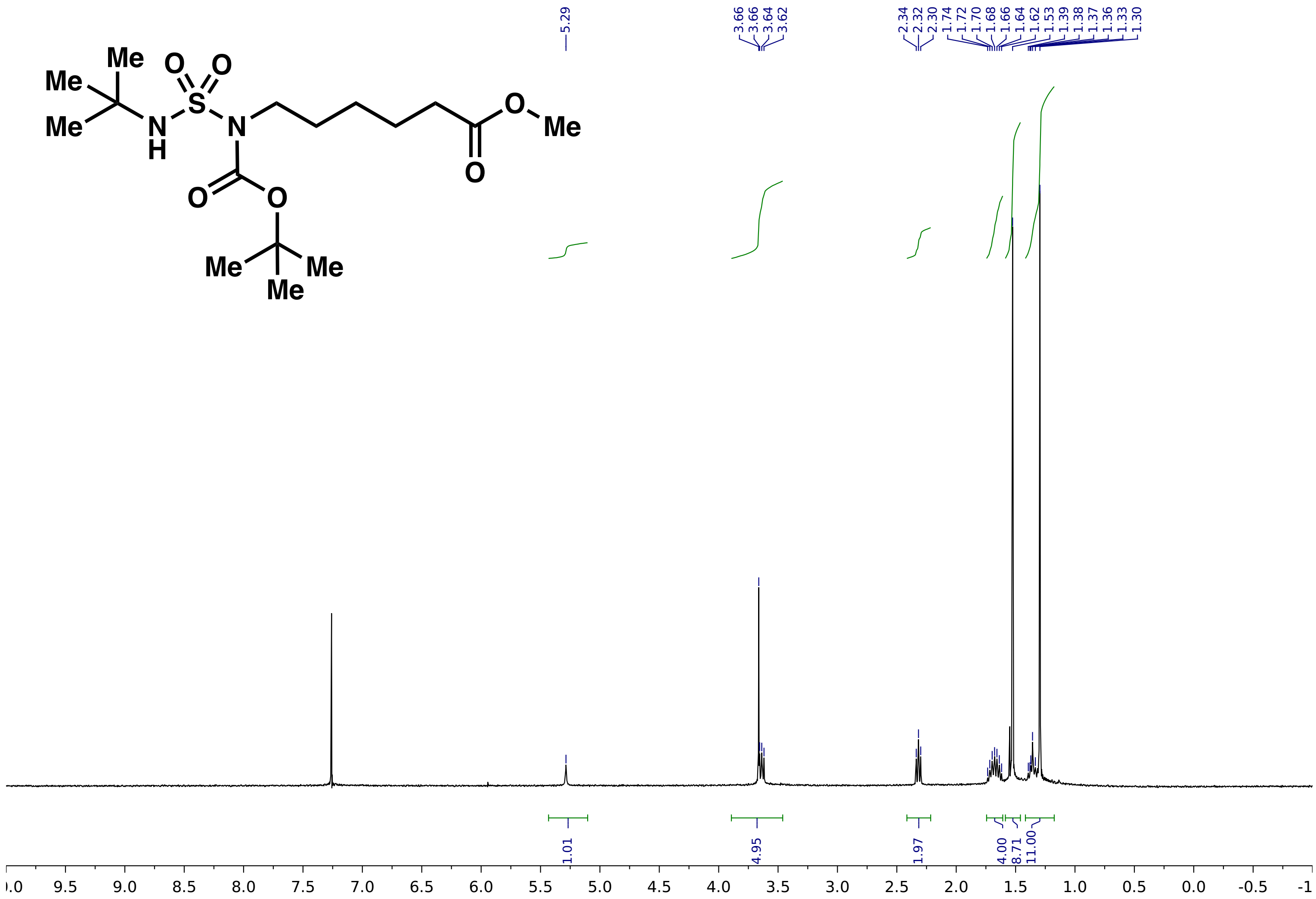

^{1}H NMR (400 MHz, $CDCl_3$) for *N*-*tert*-butyoxycarbonyl-*N'*-*tert*-butyl-*N*-(methyl hexano-6-yl)sulfamide (**S8k**)

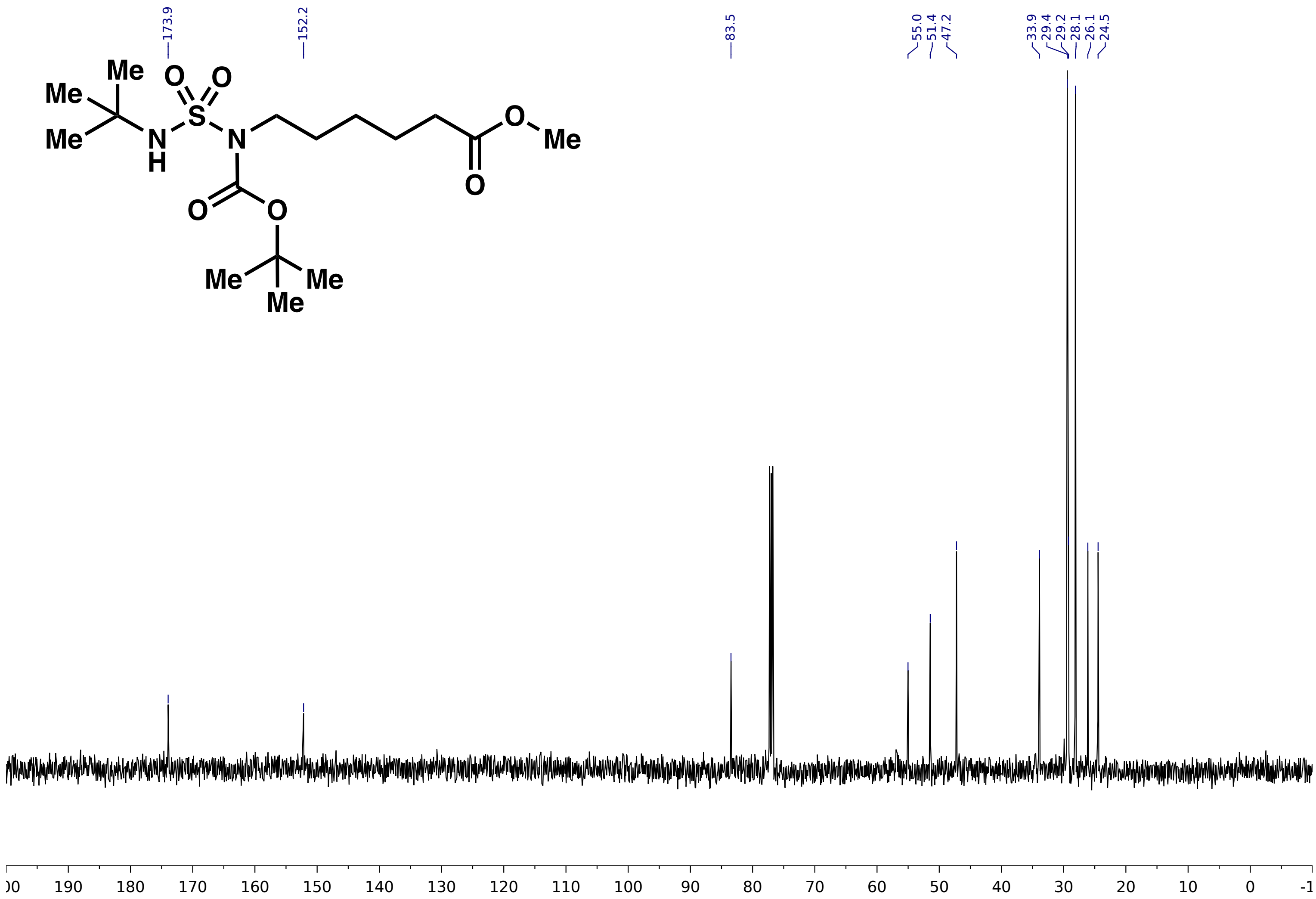

^{13}C NMR (126 MHz, $CDCl_3$) for *N*-*tert*-butyoxycarbonyl-*N'*-*tert*-butyl-*N*-(methyl hexano-6-yl)sulfamide (**S8k**)

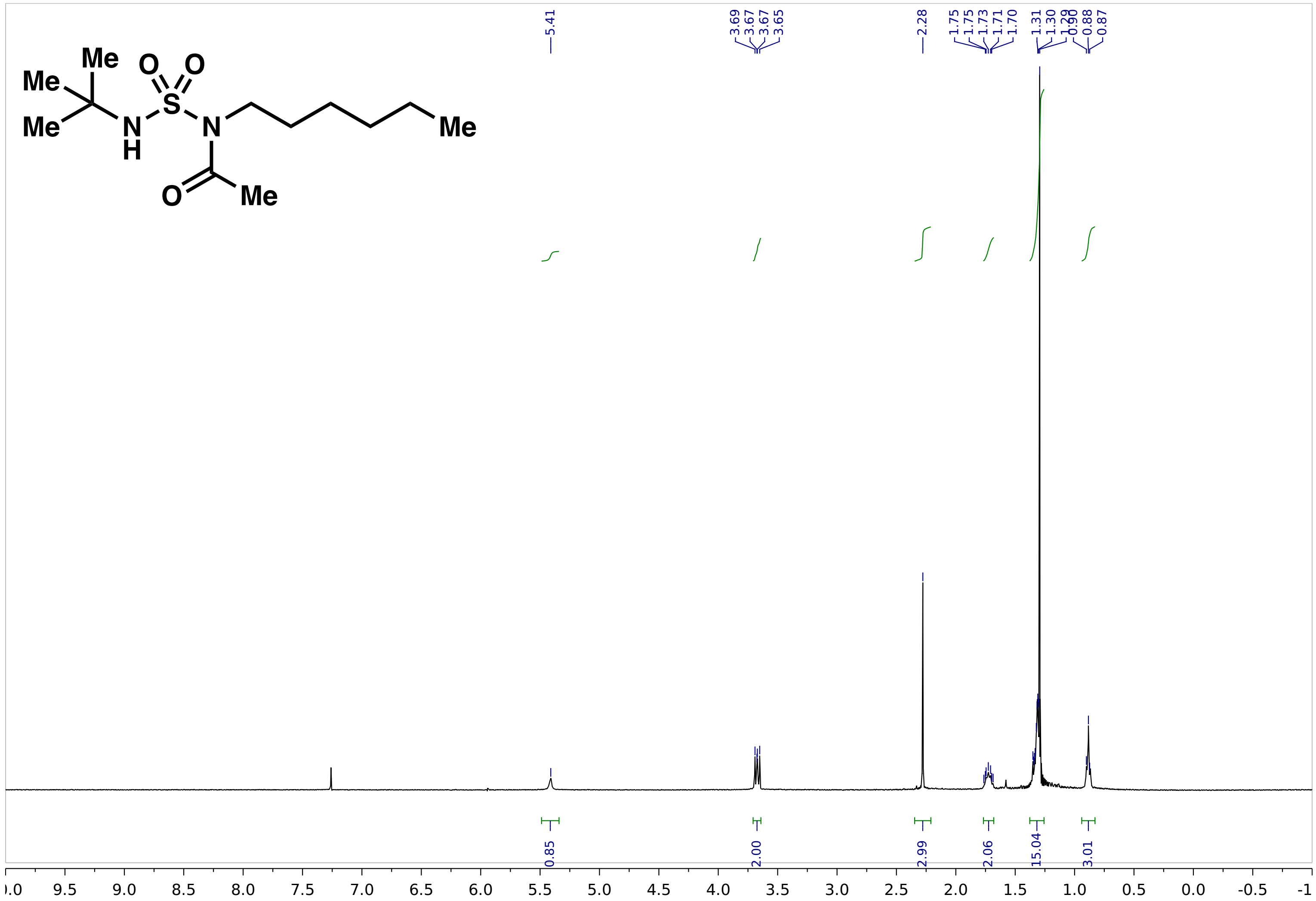

^{1}H NMR (400 MHz, $CDCl_3$) for *N*-acetyl-*N*’-*tert*-butyl-*N*-hexylsulfamide (**S8l**)

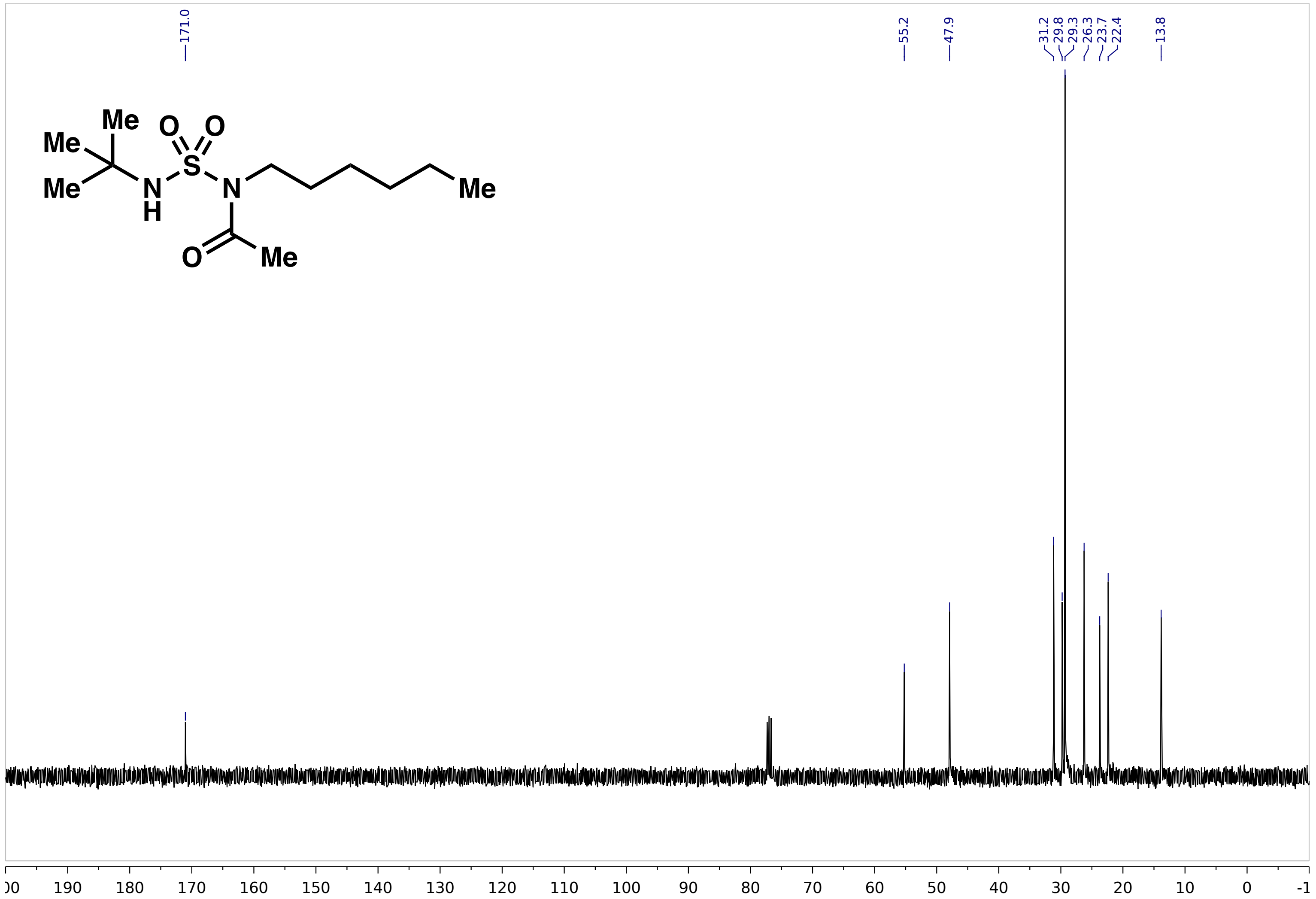

^{13}C NMR (101 MHz, $CDCl_3$) for *N*-acetyl-*N*'-*tert*-butyl-*N*-hexylsulfamide (**S8l**)

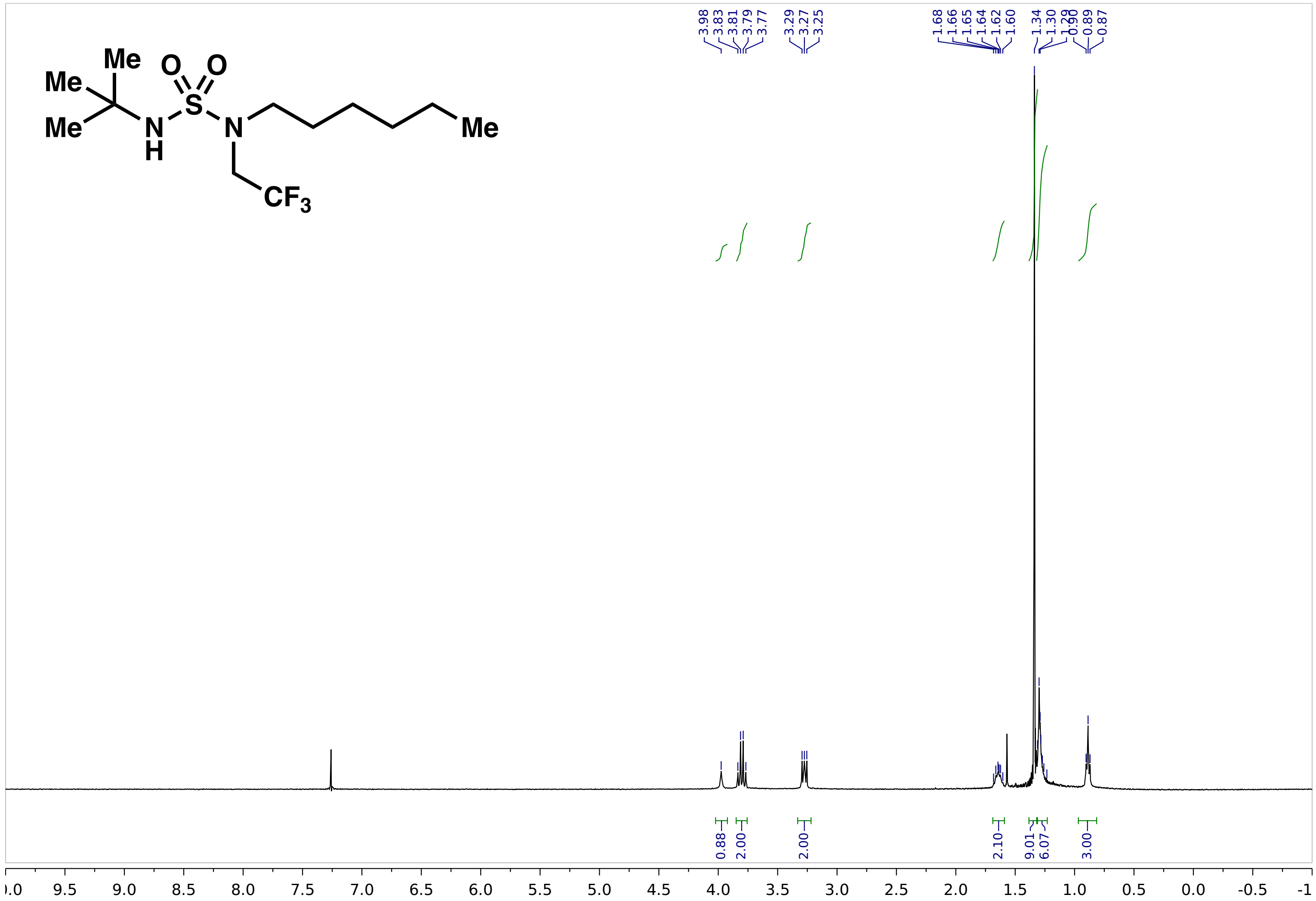

^{1}H NMR (400 MHz, $CDCl_3$) for *N*-*tert*-butyl-*N'*-hexyl-*N'*-(2,2,2-trifluoroethyl)sulfamide (**S8m**)

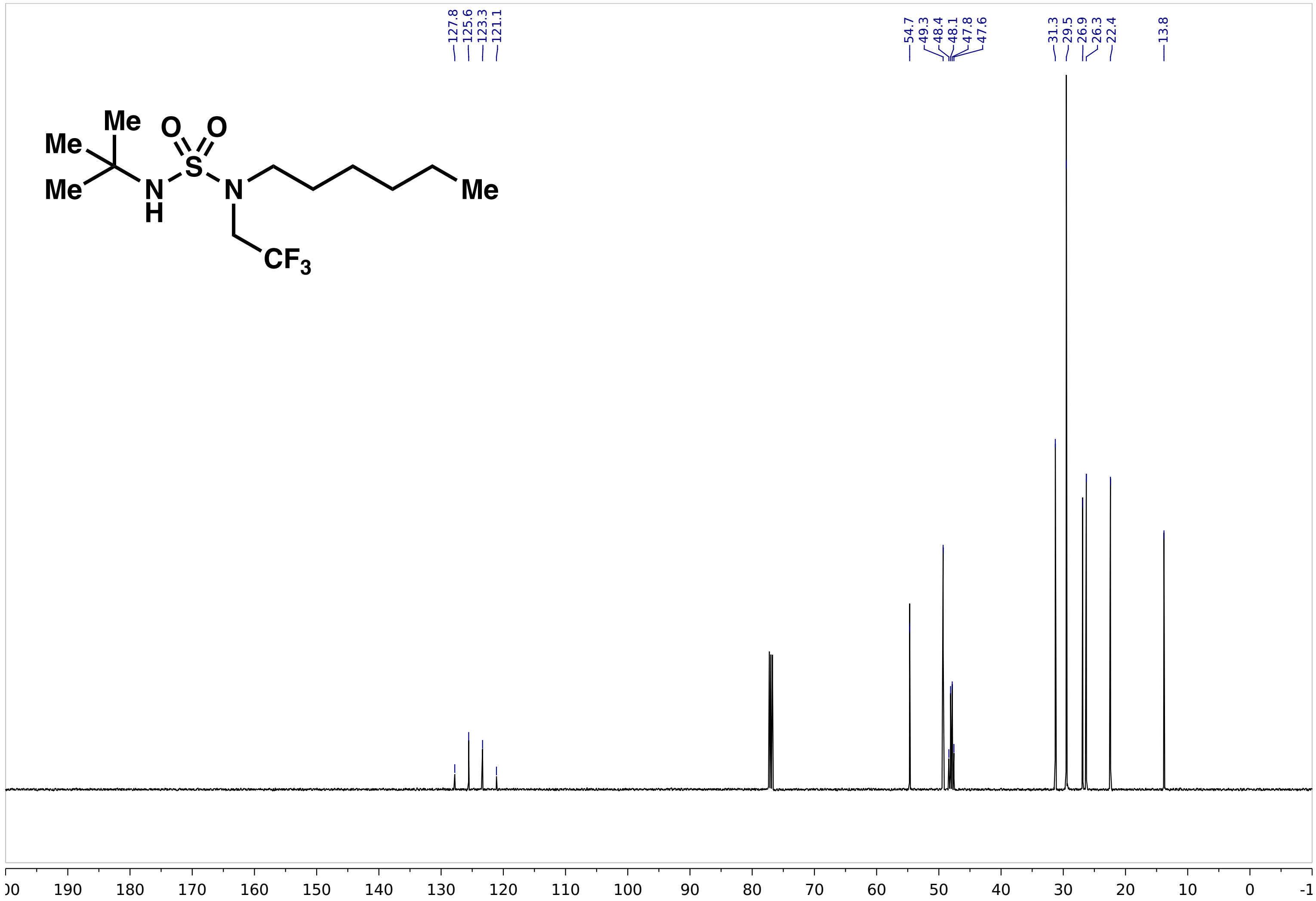

^{13}C NMR (126 MHz, $CDCl_3$) for *N*-*tert*-butyl-*N'*-hexyl-*N*'-(2,2,2-trifluoroethyl)sulfamide (**S8m**)

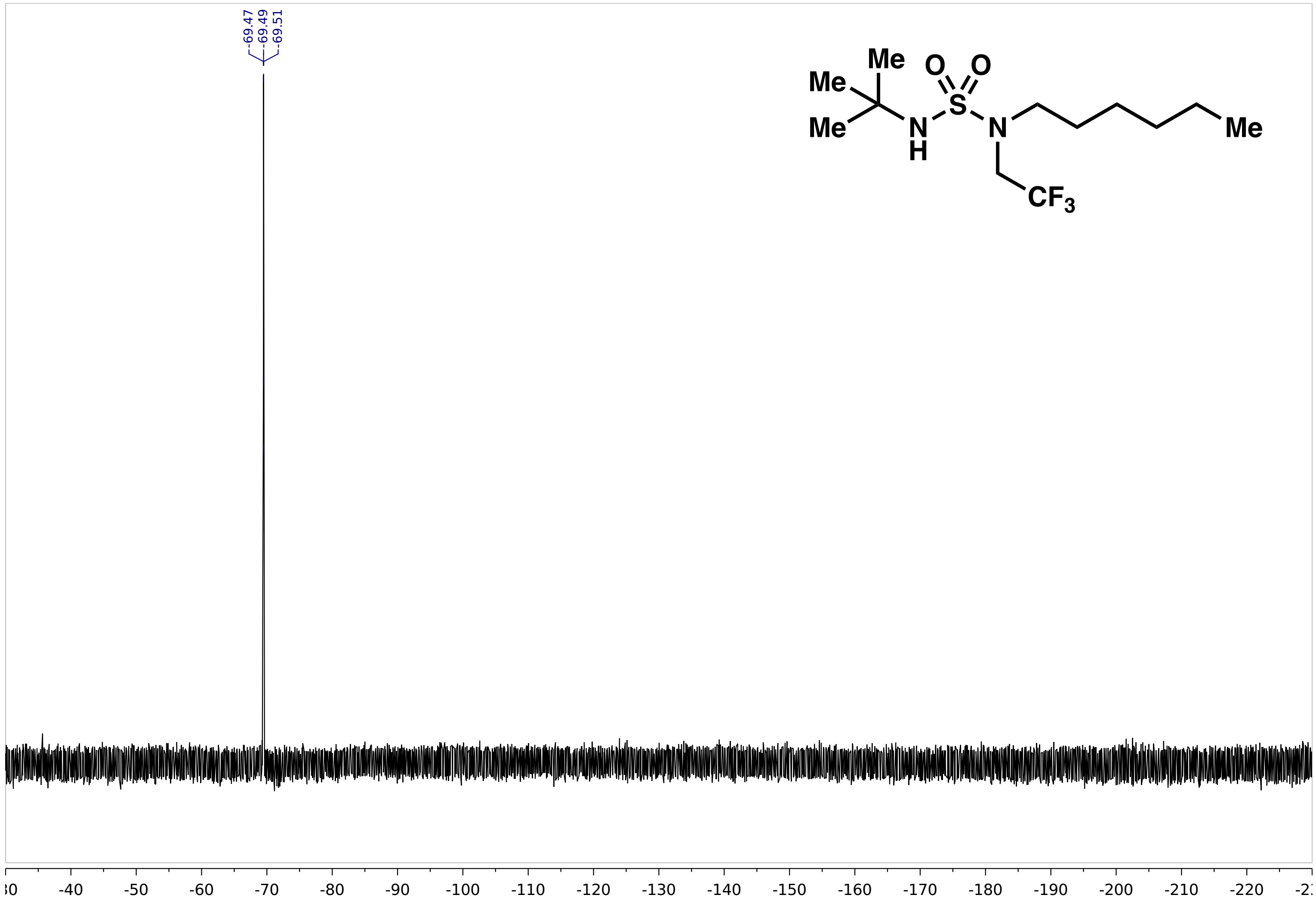

^{19}F NMR (376 MHz, $CDCl_3$) for *N*-*tert*-butyl-*N'*-hexyl-*N*'-(2,2,2-trifluoroethyl)sulfamide (**S8m**)

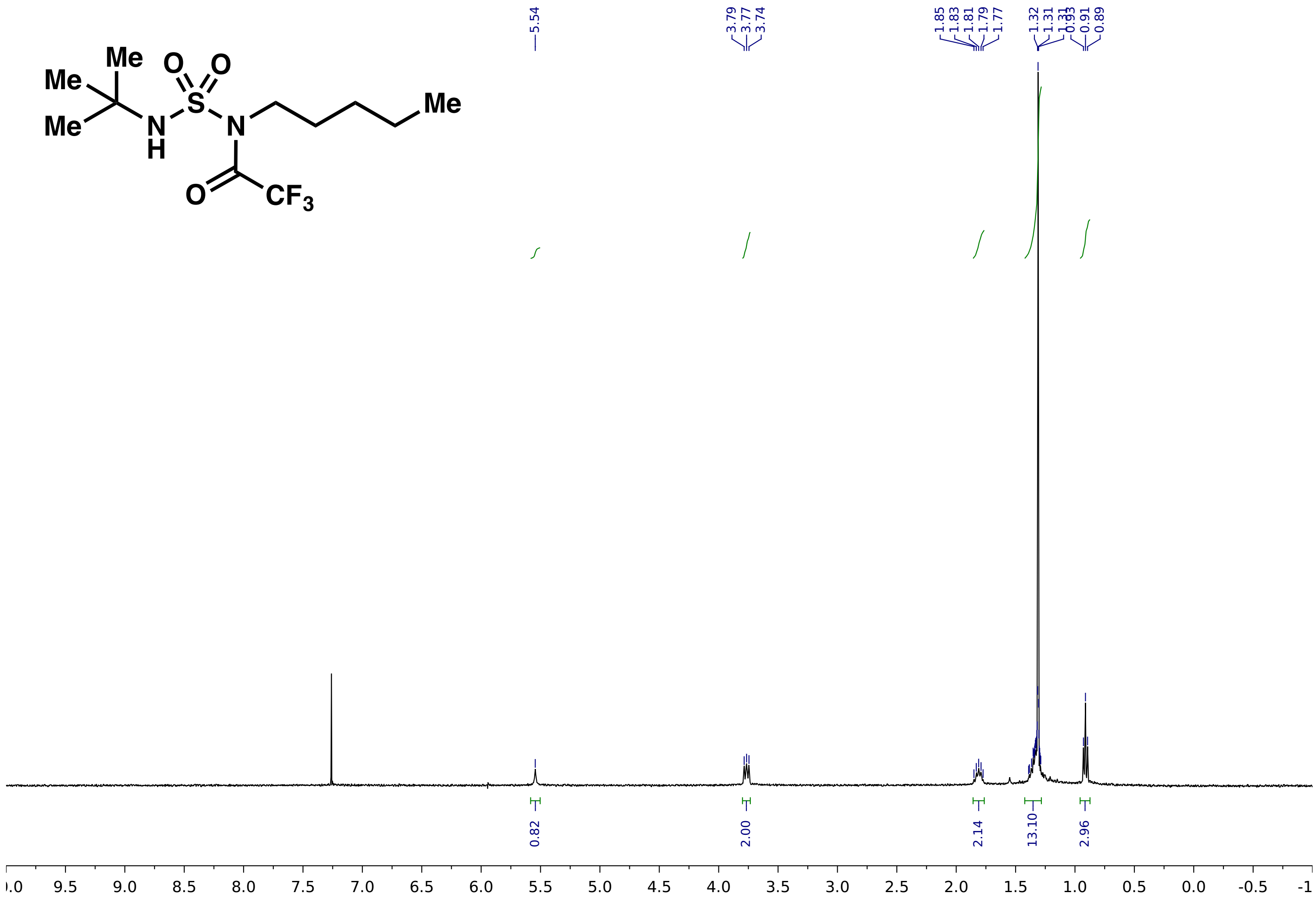

^{1}H NMR (400 MHz, $CDCl_3$) for *N'*-*tert*-butyl-*N*-trifluoroacetyl-*N*-pentylsulfamide (**S8n**)

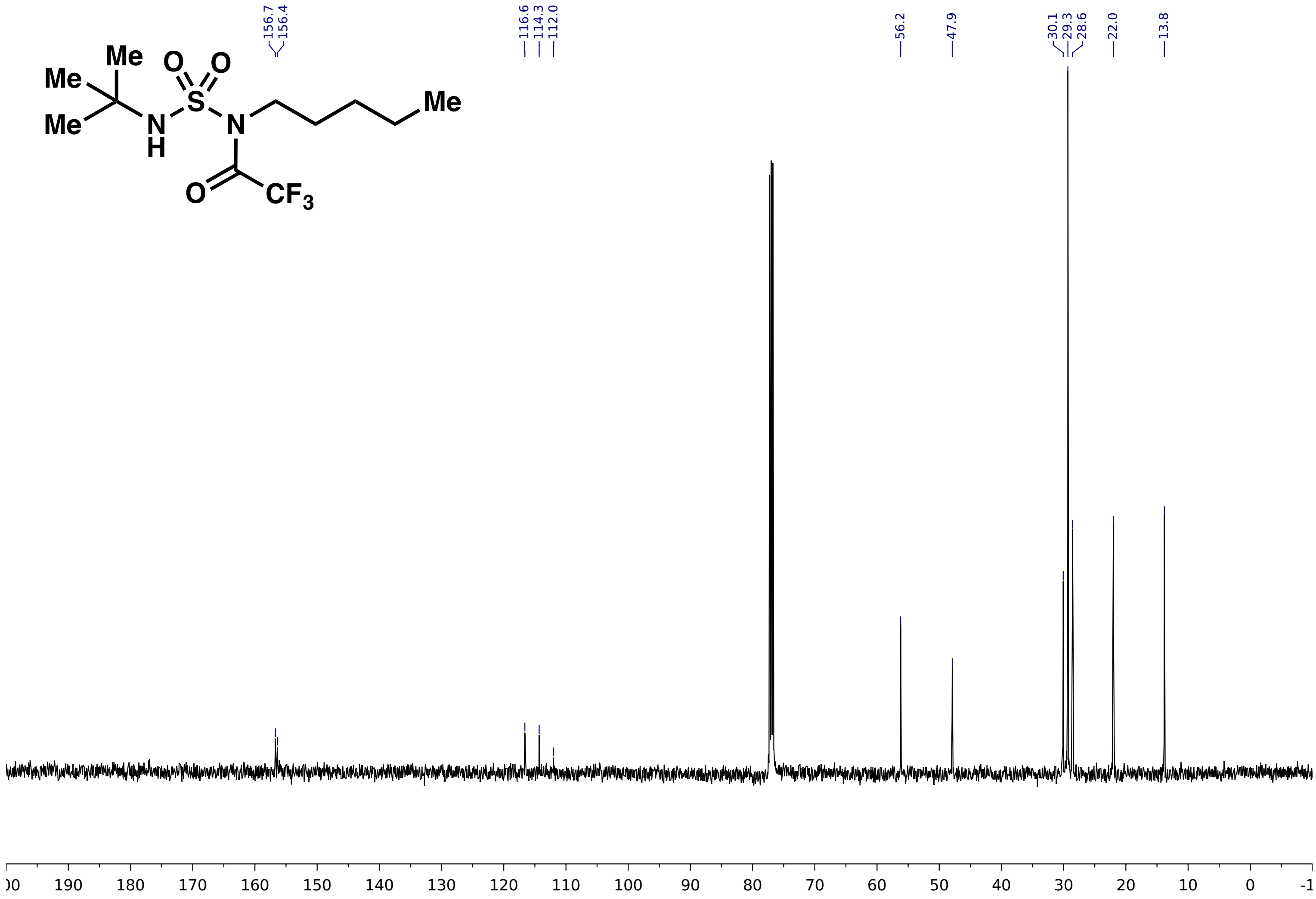

^{13}C NMR (126 MHz, $CDCl_3$) for *N'*-*tert*-butyl-*N*-trifluoroacetyl-*N*-pentylsulfamide (**S8n**)

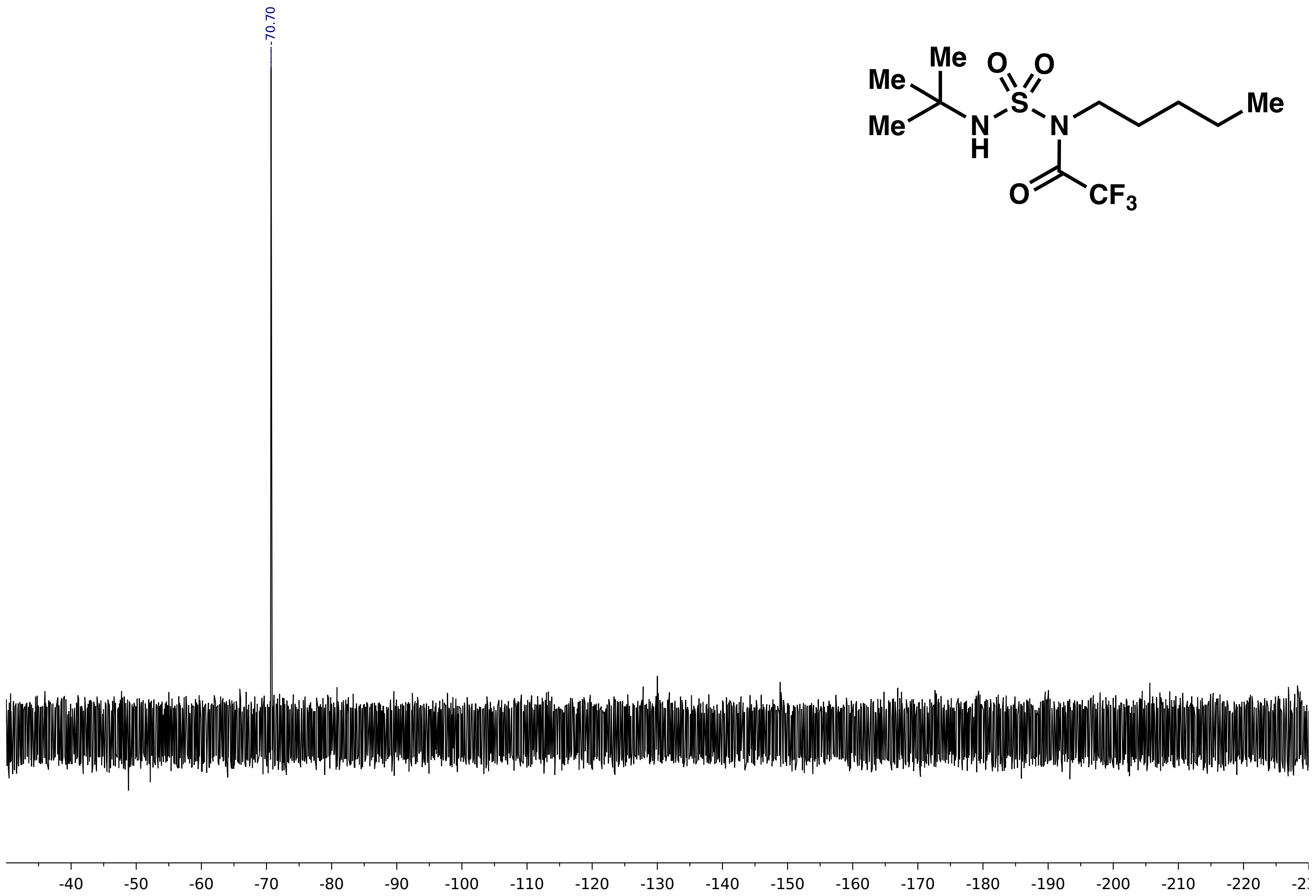

^{19}F NMR (376 MHz, $CDCl_3$) for *N'*-*tert*-butyl-*N*-trifluoroacetyl-*N*-pentylsulfamide (**S8n**)

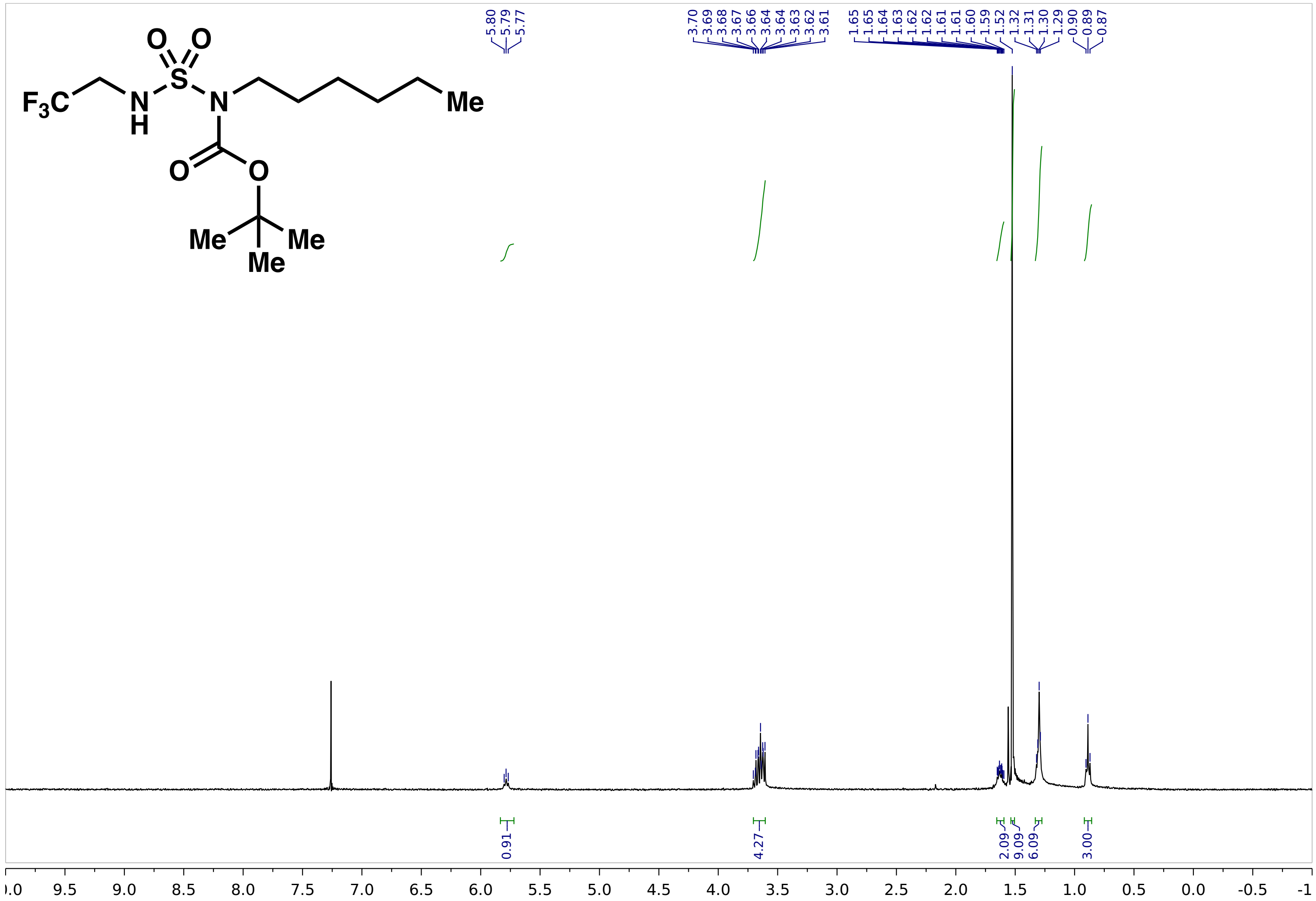

^{1}H NMR (400 MHz, $CDCl_3$) for *N*-*tert*-butoxycarbonyl-*N*-hexyl-*N'*-(2,2,2-trifluoroethyl)sulfamide (**S8o**)

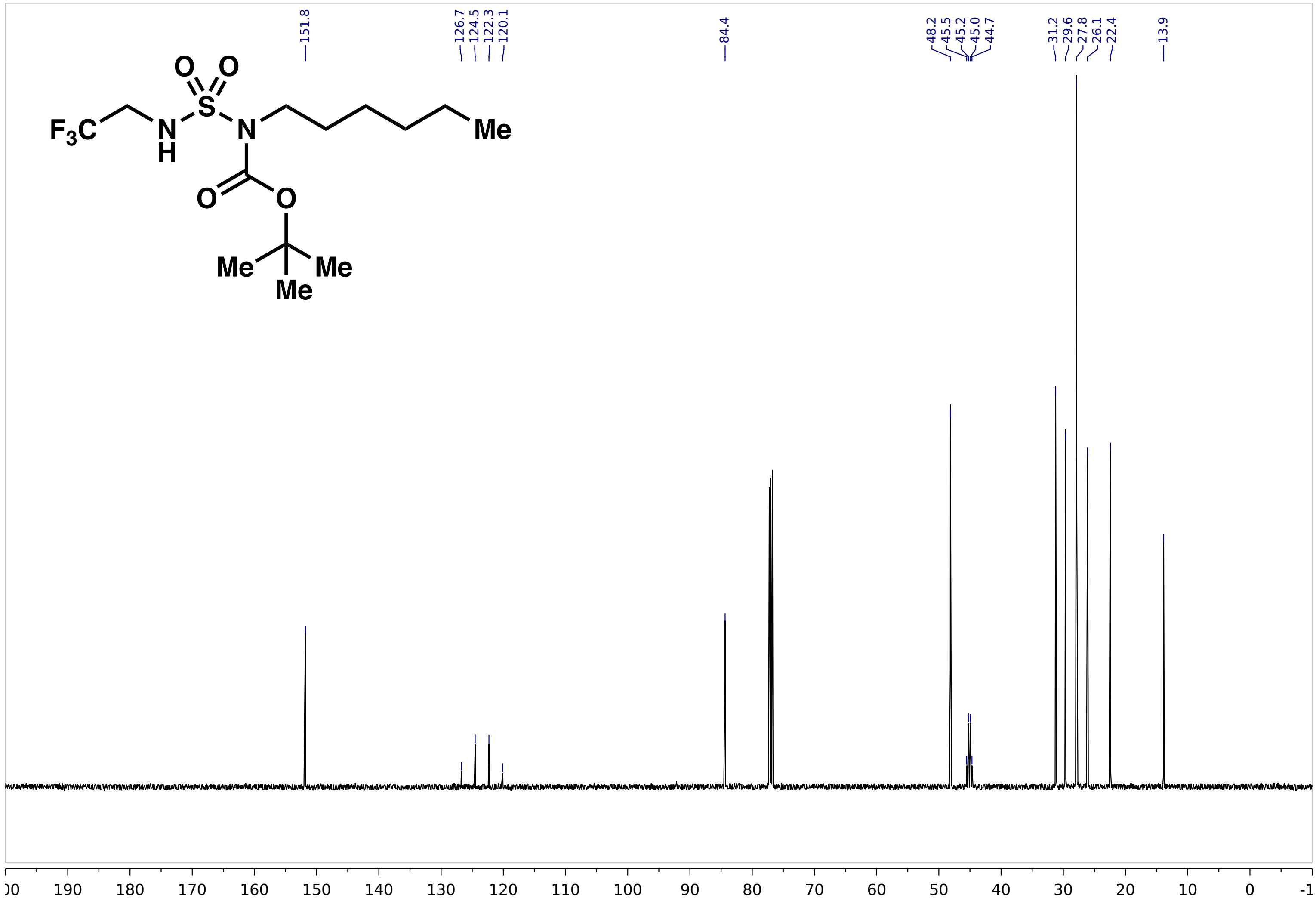

^{13}C NMR (126 MHz, $CDCl_3$) for *N*-*tert*-butoxycarbonyl-*N*-hexyl-*N'*-(2,2,2-trifluoroethyl)sulfamide (**S8o**)

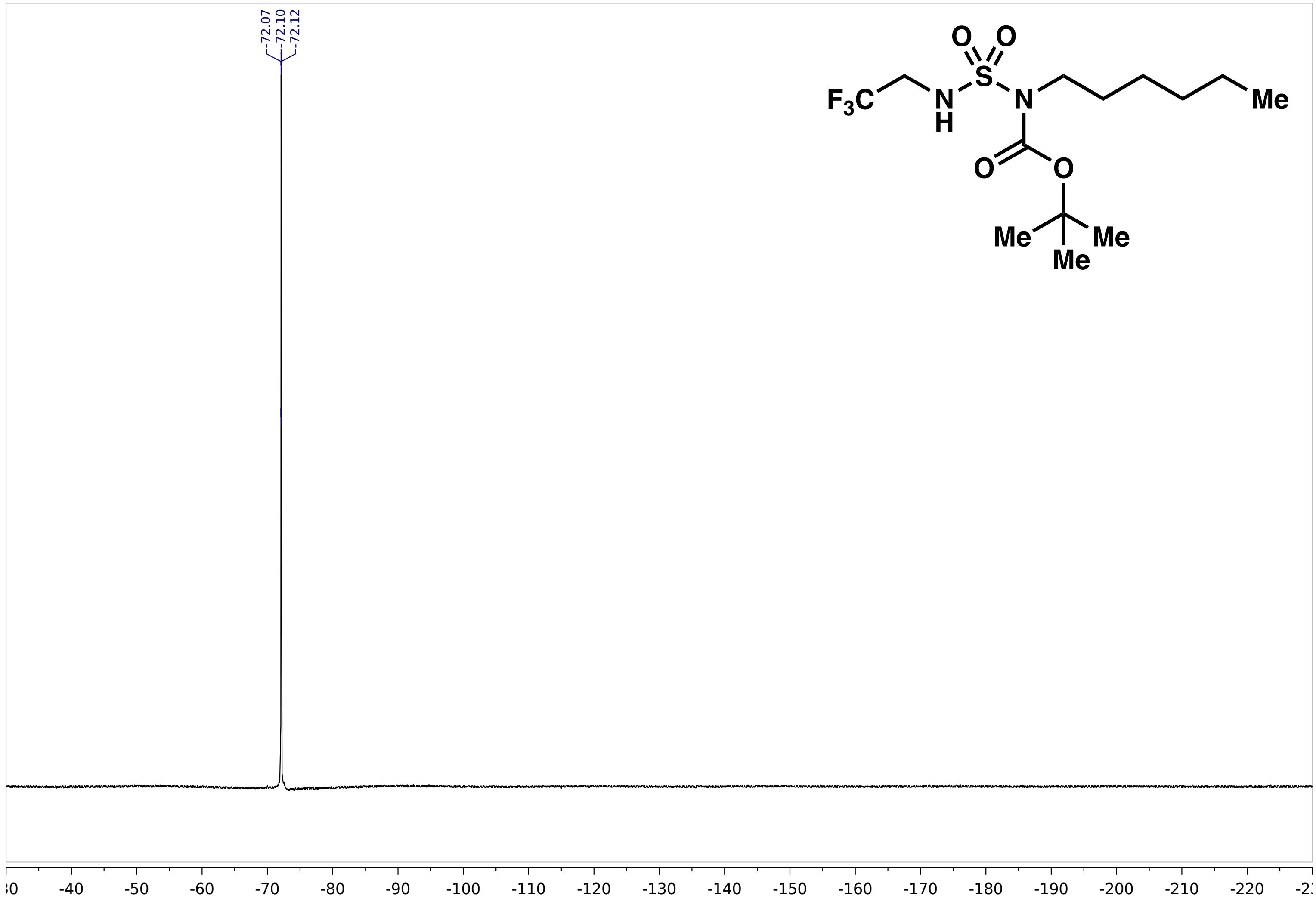

^{19}F NMR (376 MHz, $CDCl_3$) for *N*-*tert*-butoxycarbonyl-*N*-hexyl-*N'*-(2,2,2-trifluoroethyl)sulfamide (**S8o**)

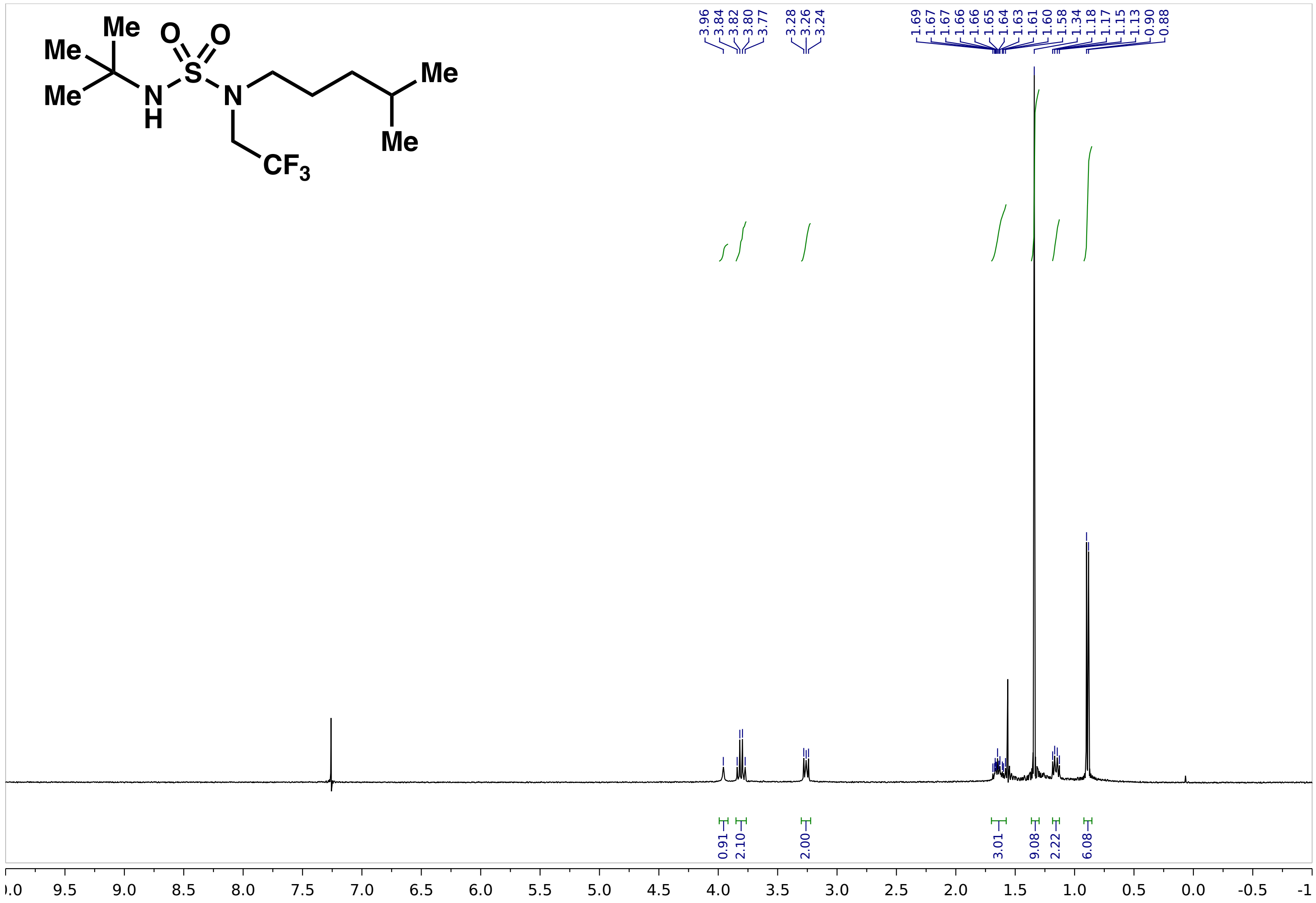

^{1}H NMR (400 MHz, CDCl₃) for *N*-*tert*-Butyl-*N'*-4-methylpentyl-*N'*-(2,2,2-trifluoroethyl)sulfamide (**S8p**)

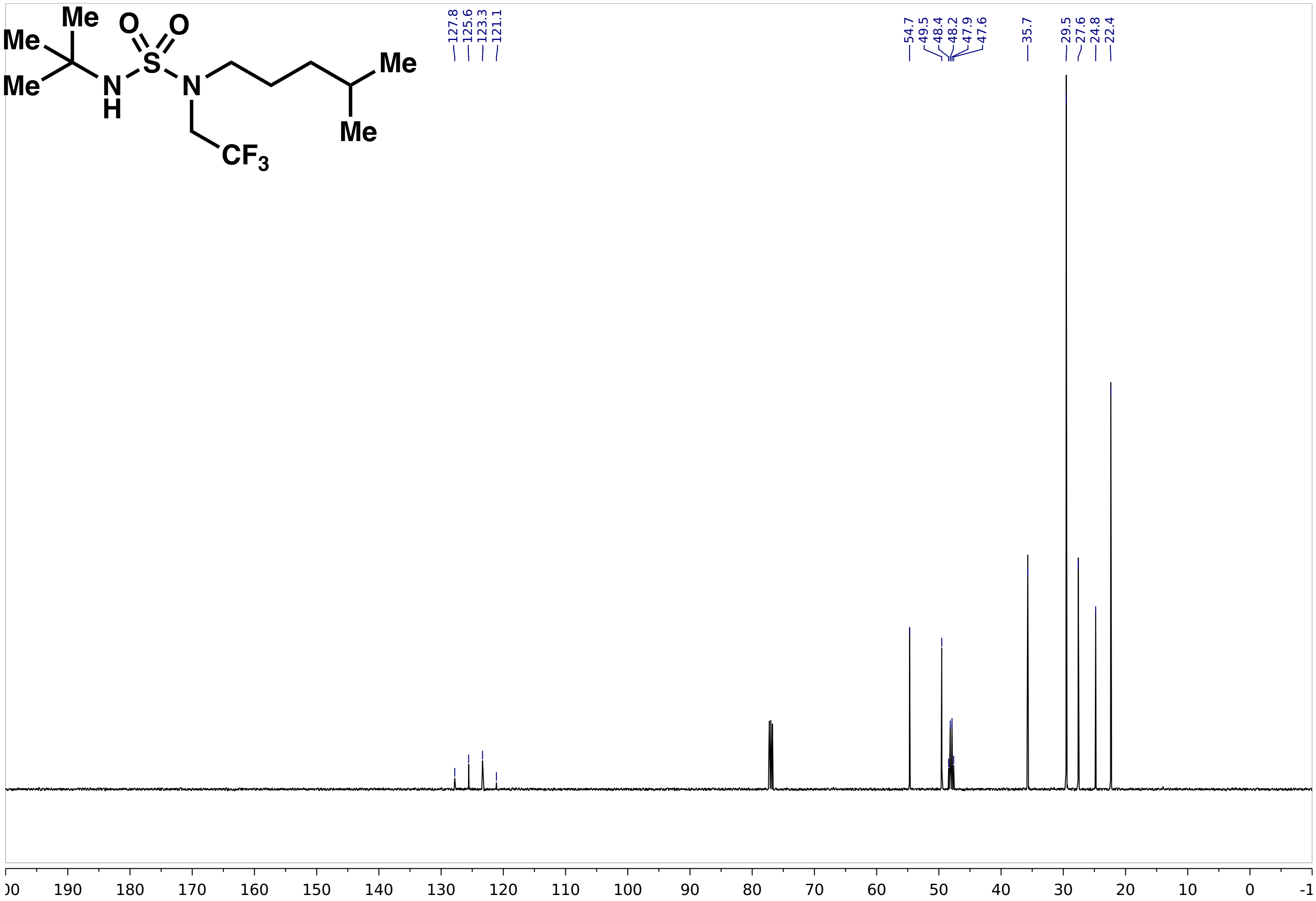

^{13}C NMR (126 MHz, $CDCl_3$) for *N*-*tert*-Butyl-*N'*-4-methylpentyl-*N*'-(2,2,2-trifluoroethyl)sulfamide (**S8p**)

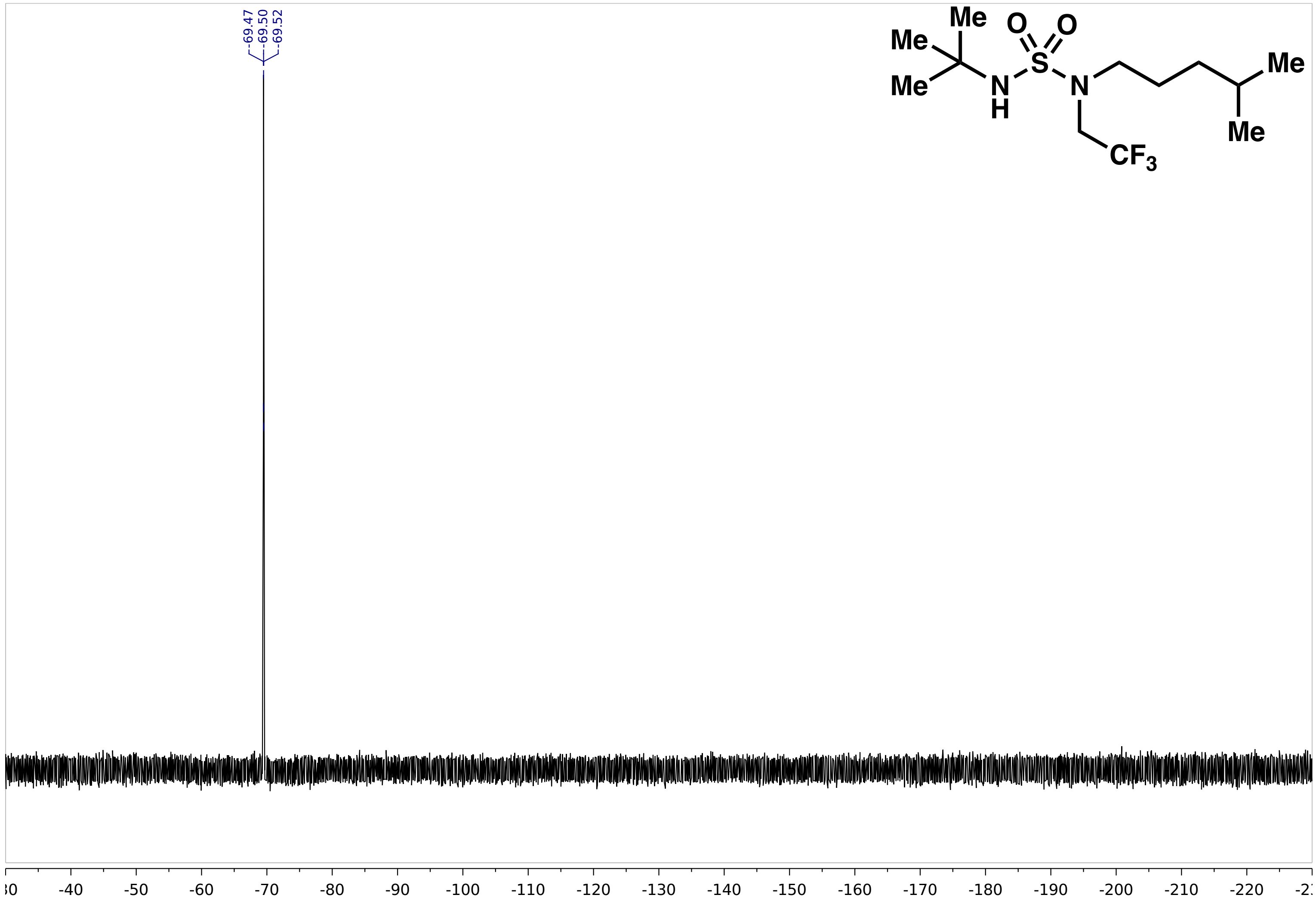

^{19}F NMR (376 MHz, $CDCl_3$) for *N-tert*-Butyl-*N'*-4-methylpentyl-*N*'-(2,2,2-trifluoroethyl)sulfamide (**S8p**)

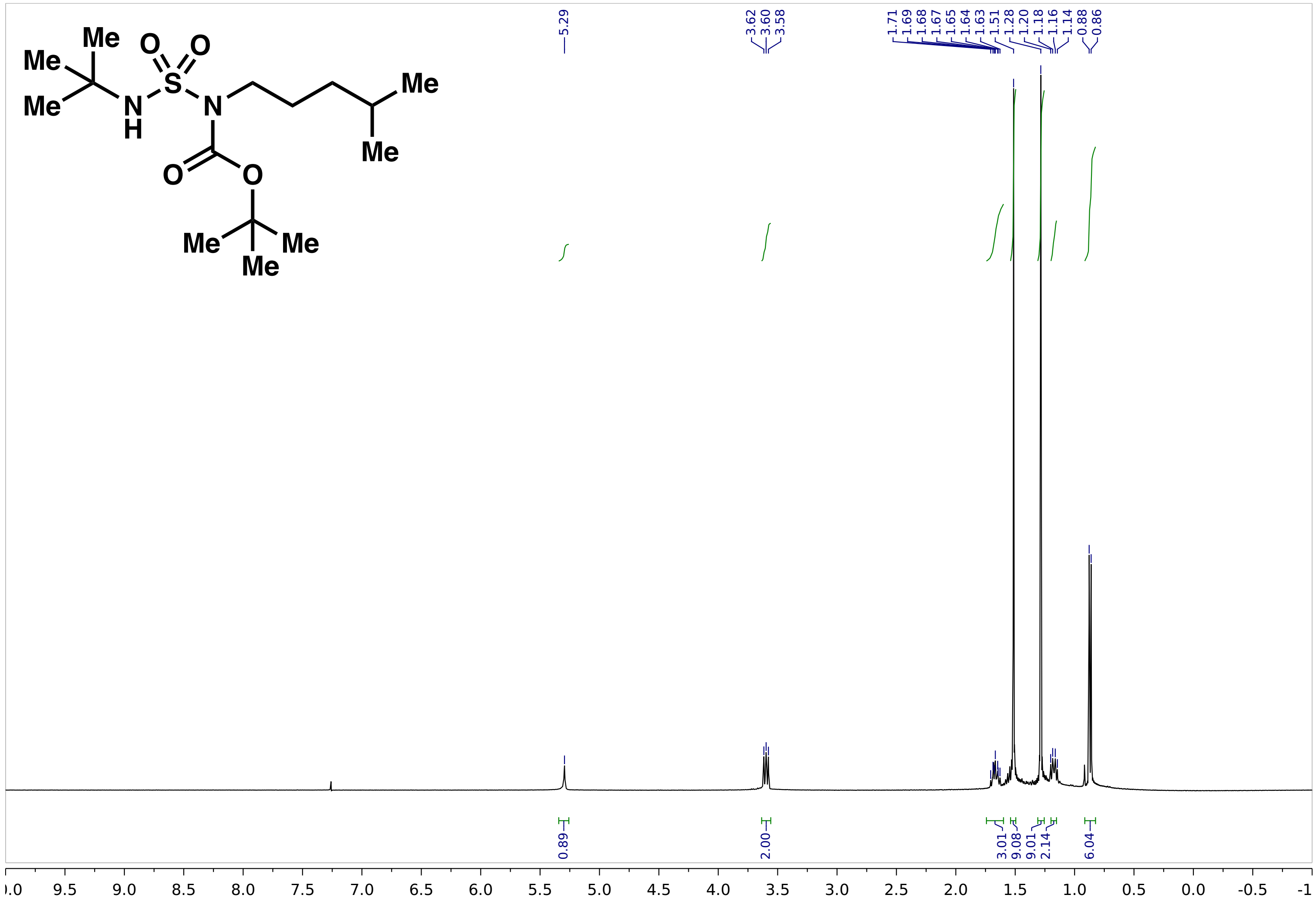

^{1}H NMR (400 MHz, $CDCl_3$,) for *N*-*tert*-butoxycarbonyl-*N'*-*tert*-butyl-*N*-4-methylpentylsulfamide (**S8q**)

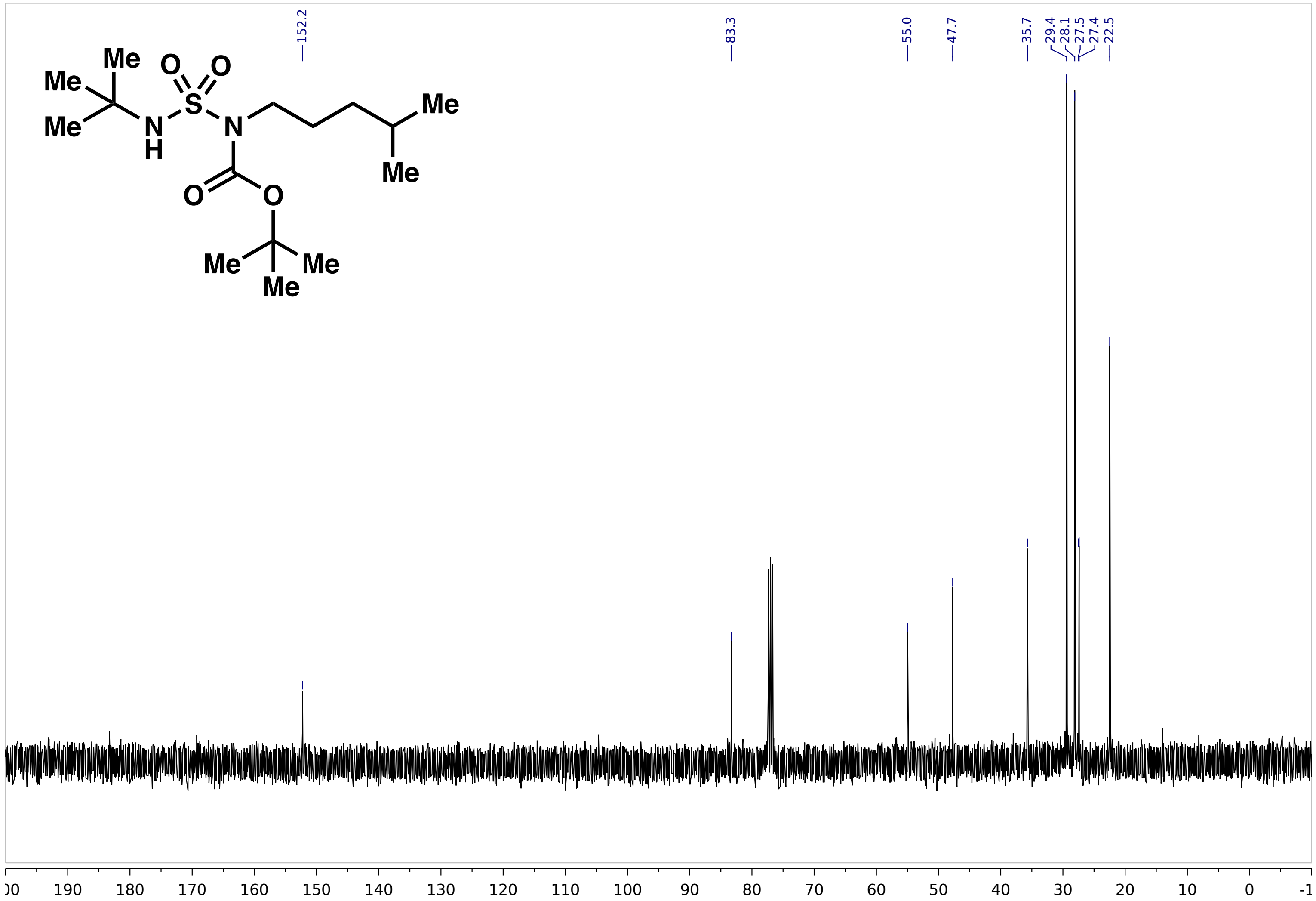

^{13}C NMR (101 MHz, $CDCl_3$) for *N*-*tert*-butoxycarbonyl-*N'*-*tert*-butyl-*N*-4-methylpentylsulfamide (**S8q**)

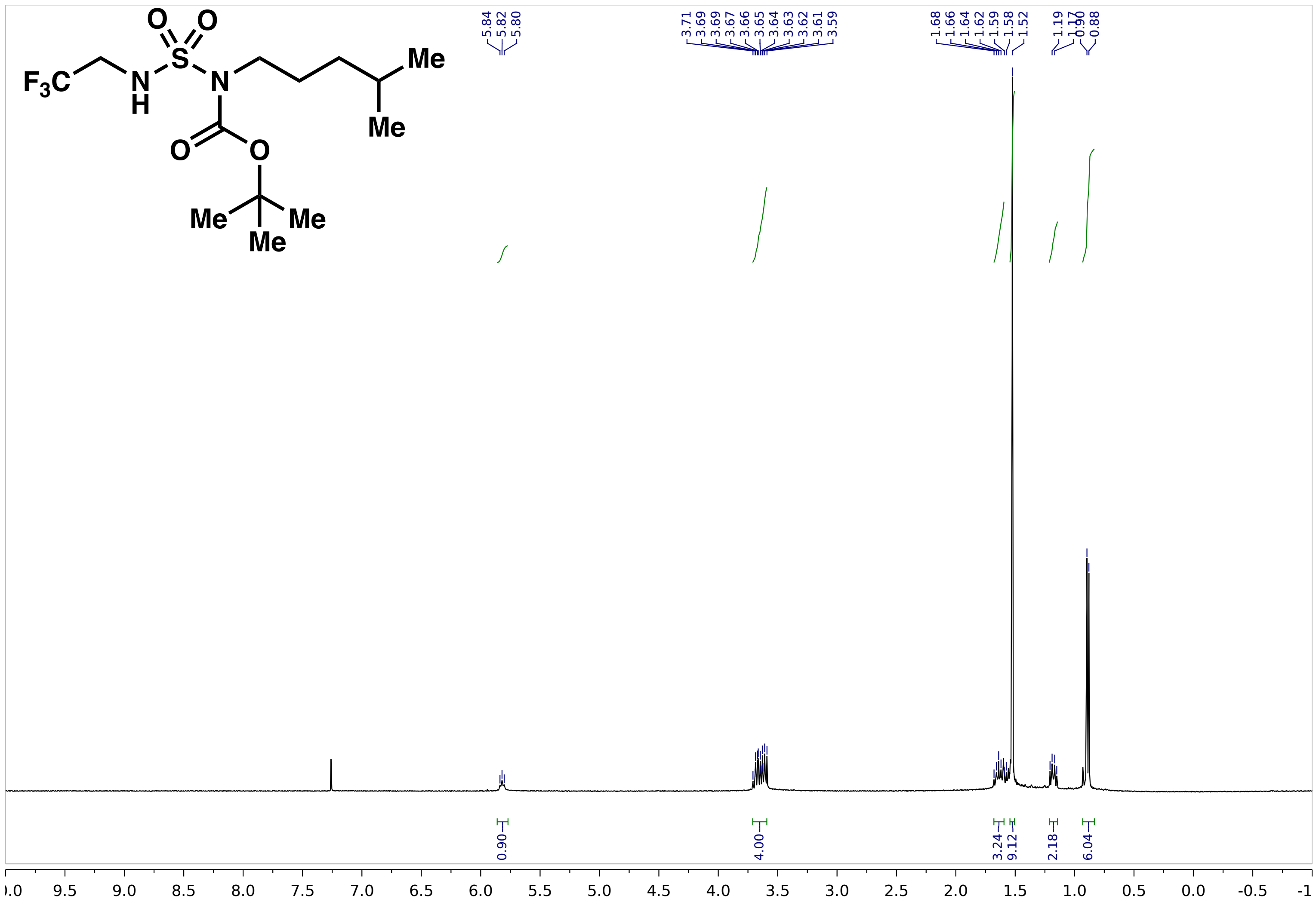

^{1}H NMR (400 MHz, $CDCl_3$) for *N*-*tert*-Butoxycarbonyl-*N*-4-methylpentyl-*N'*-(2,2,2-trifluoroethyl)sulfamide (**S8r**)

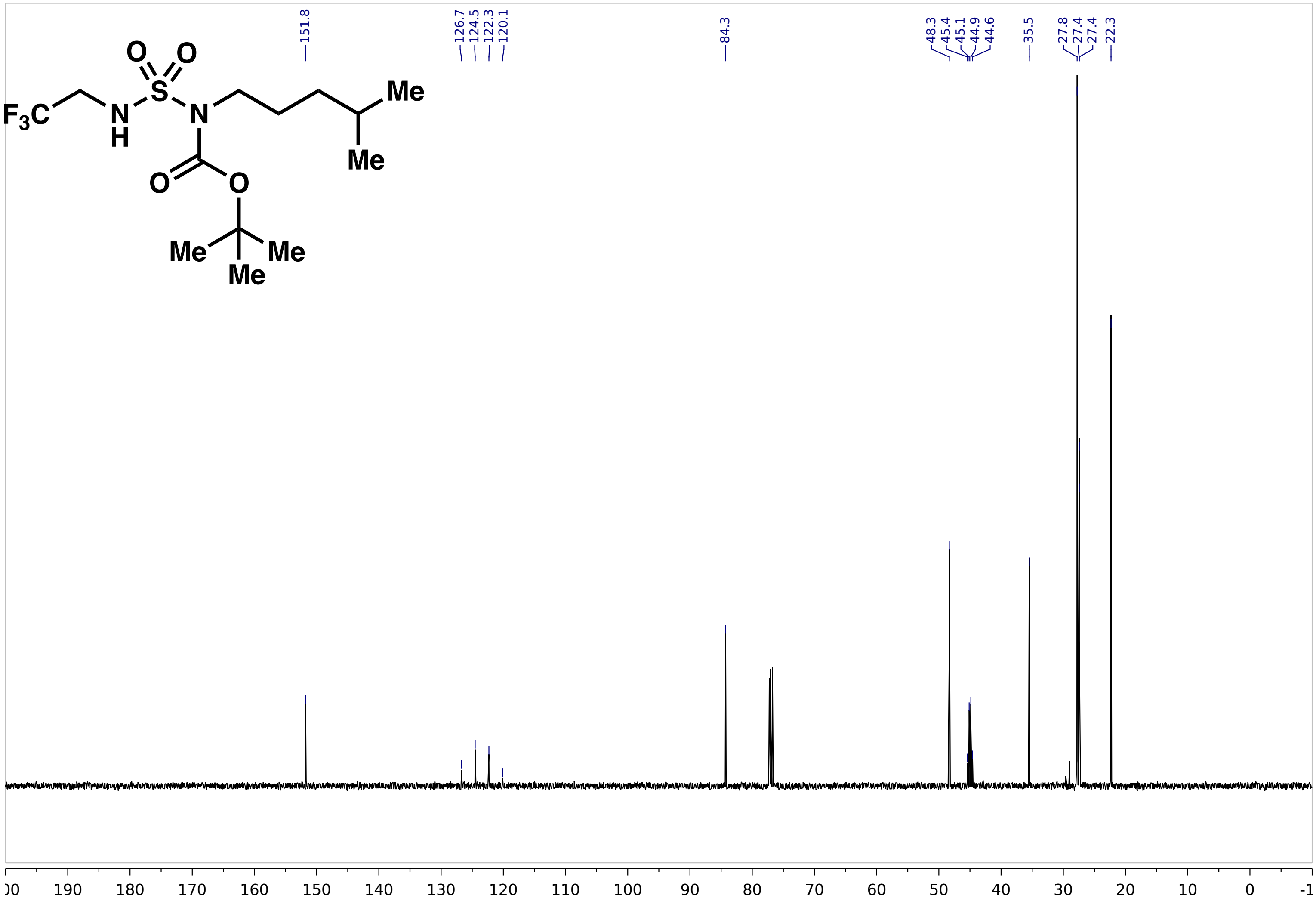

^{13}C NMR (126 MHz, $CDCl_3$) for *N*-*tert*-Butoxycarbonyl-*N*-4-methylpentyl-*N'*-(2,2,2-trifluoroethyl)sulfamide (**S8r**)

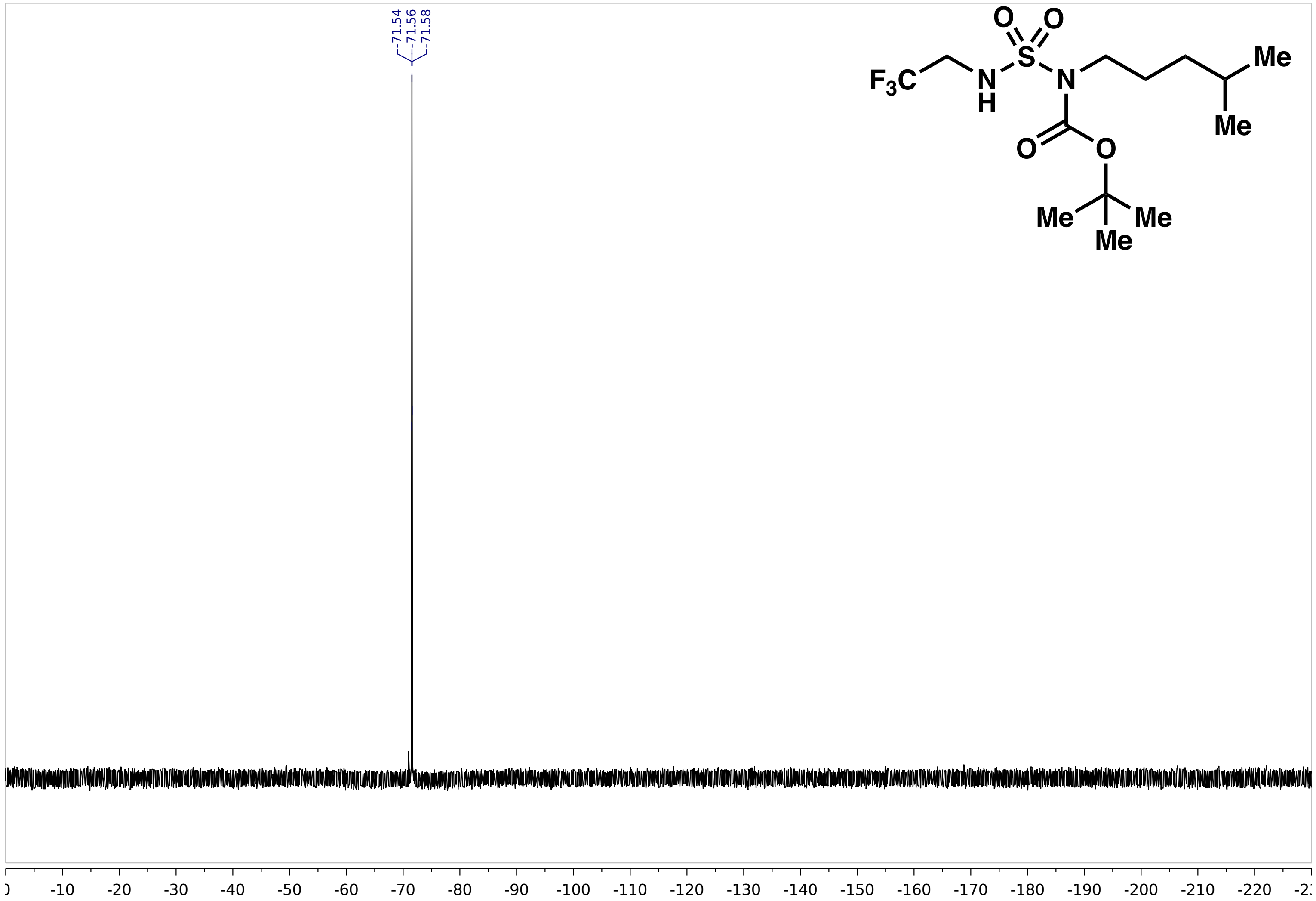

^{19}F NMR (376 MHz, $CDCl_3$) for *N*-*tert*-Butoxycarbonyl-*N*-4-methylpentyl-*N'*-(2,2,2-trifluoroethyl)sulfamide (**S8r**)

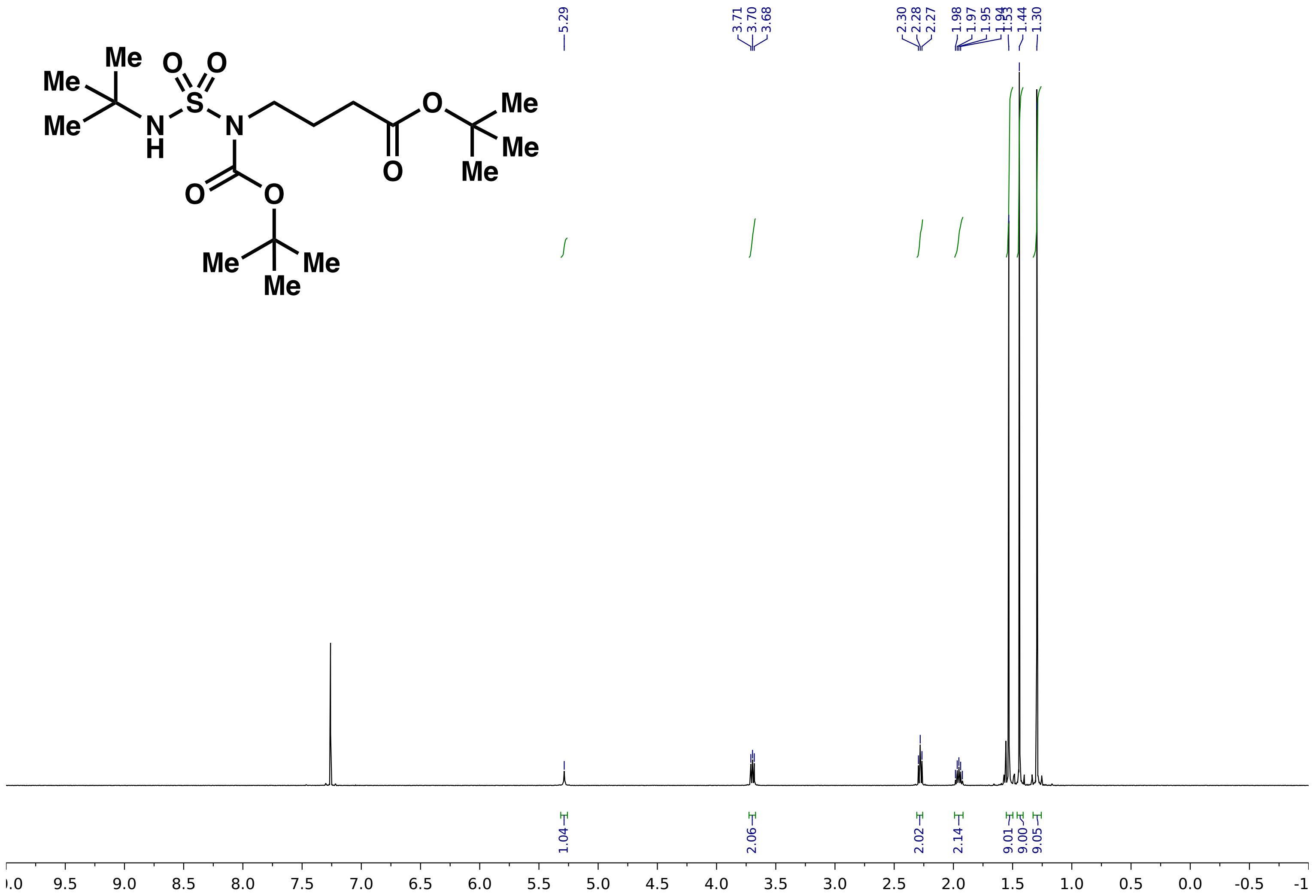

^{1}H NMR (400 MHz, $CDCl_3$) for *N*-*tert*-butoxycarbonyl-*N'*-*tert*-butyl-*N*-(*tert*-butyl butano-4-yl)sulfamide (**S8t**)

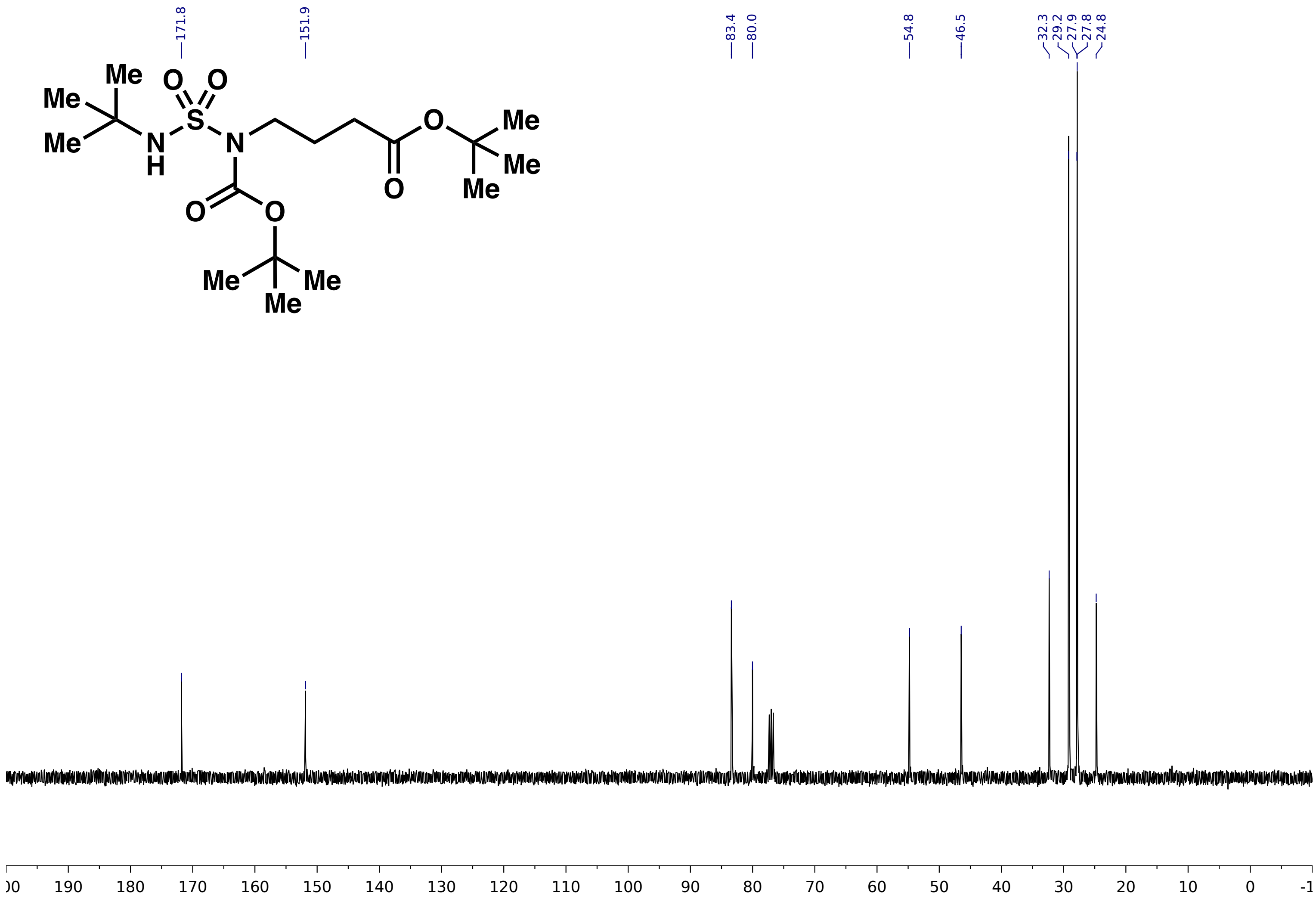

^{13}C NMR (101 MHz, $CDCl_3$) for *N*-*tert*-butoxycarbonyl-*N’*-*tert*-butyl-*N*-(*tert*-butyl butano-4-yl)sulfamide (**S8t**)

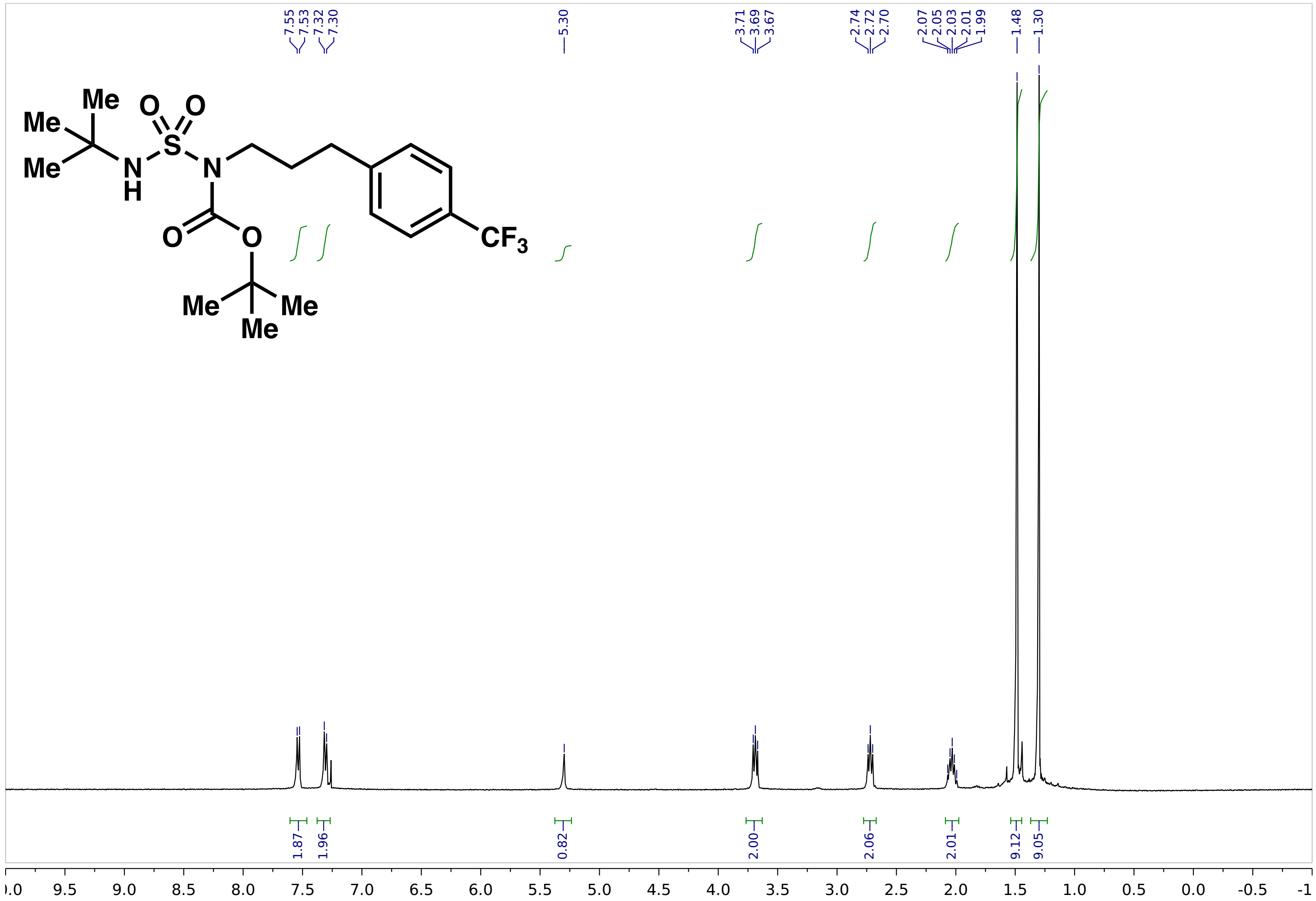

^{1}H NMR (400 MHz, $CDCl_3$) for *N*-*tert*-butoxycarbonyl-*N'*-*tert*-butyl-*N*-(3-((4-trifluoromethyl)phenyl)prop-1-yl)sulfamide (**S8u**)

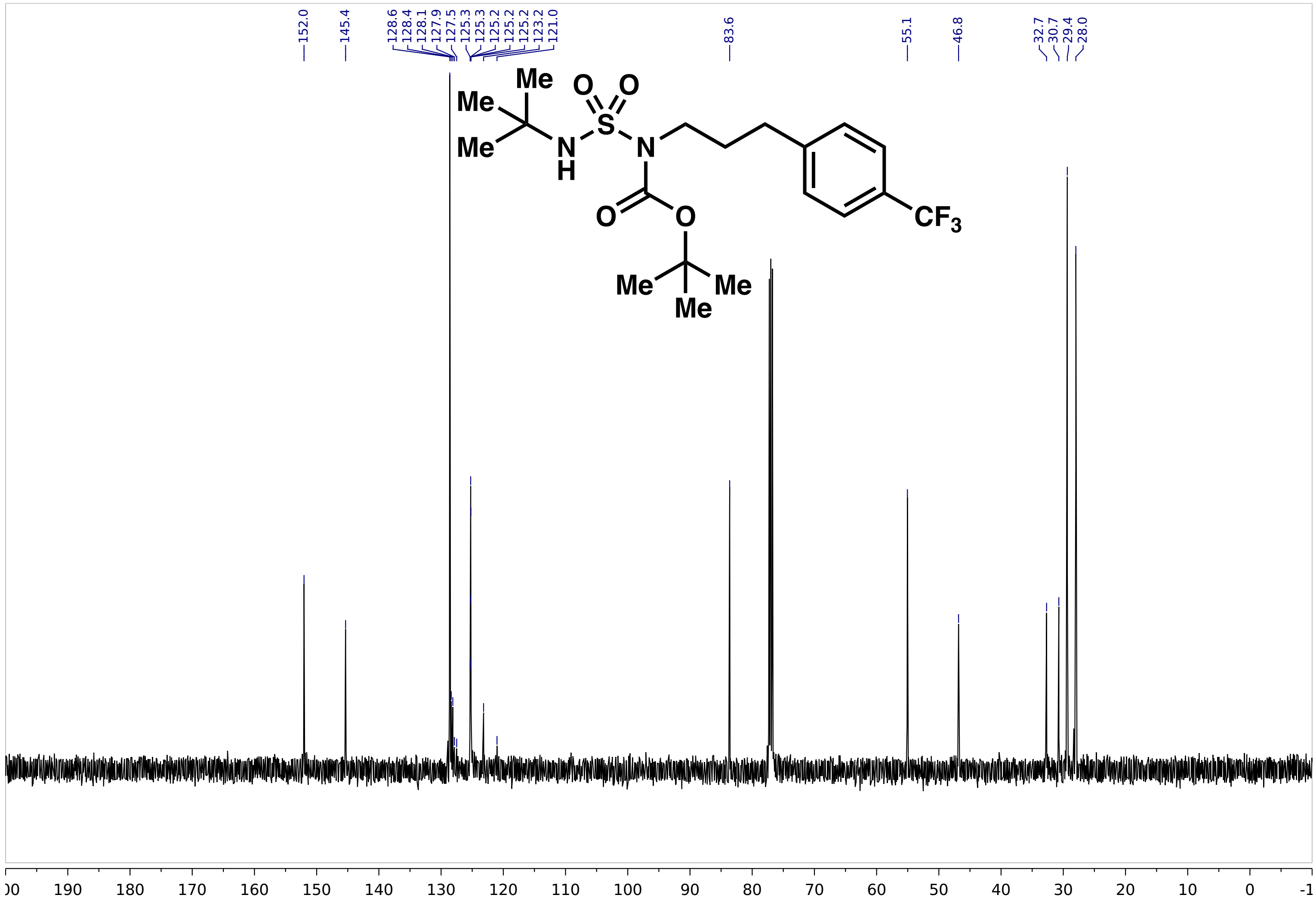

^{13}C NMR (126 MHz, $CDCl_3$) for *N*-*tert*-butoxycarbonyl-*N'*-*tert*-butyl-*N*-(3-((4-trifluoromethyl)phenyl)prop-1-yl)sulfamide (**S8u**)

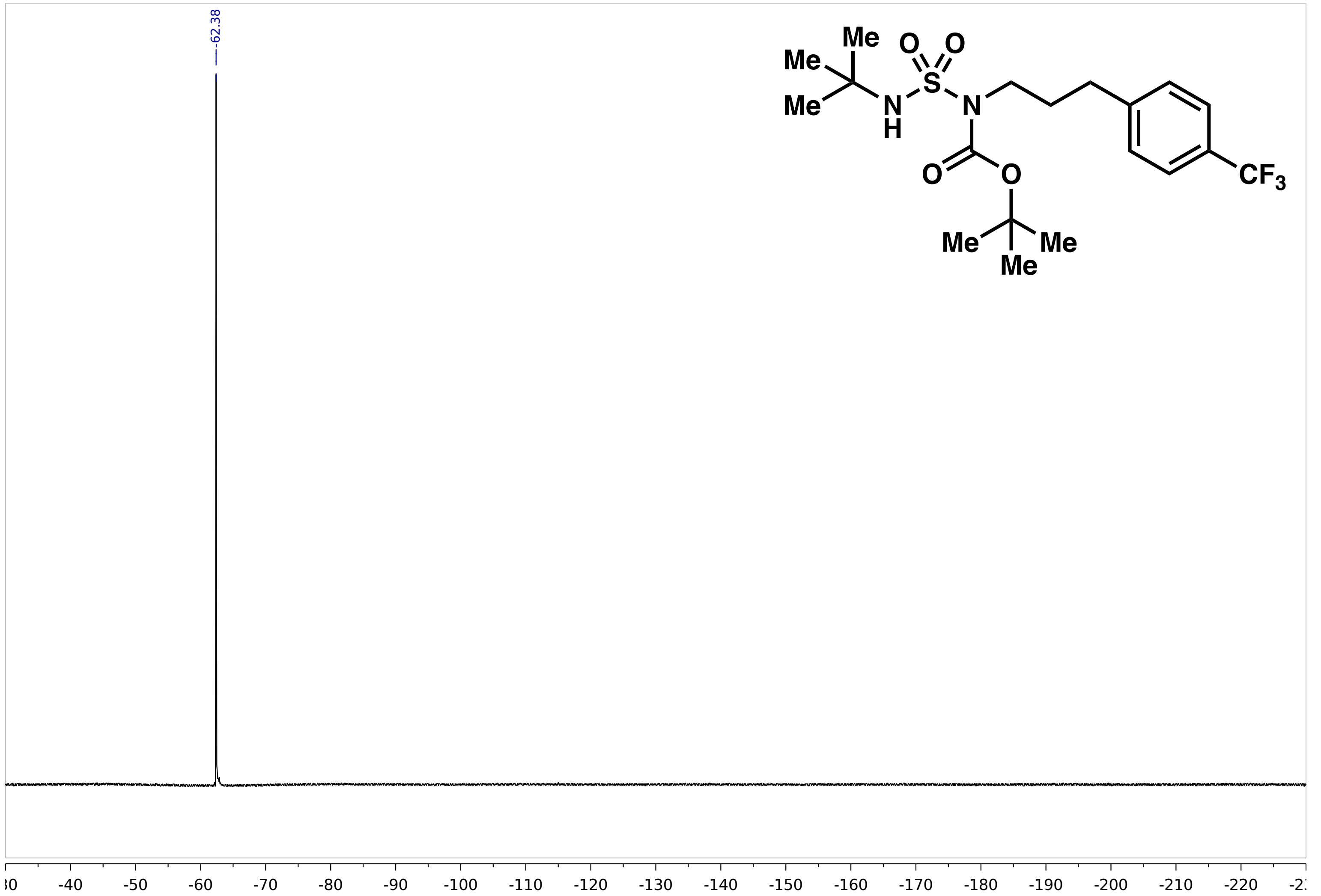

^{19}F NMR (376 MHz, $CDCl_3$) for *N*-*tert*-butoxycarbonyl-*N'*-*tert*-butyl-*N*-(3-((4-trifluoromethyl)phenyl)prop-1-yl)sulfamide (**S8u**)

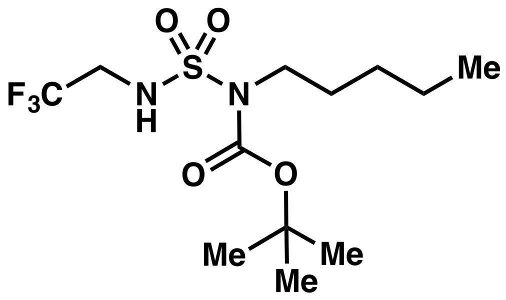

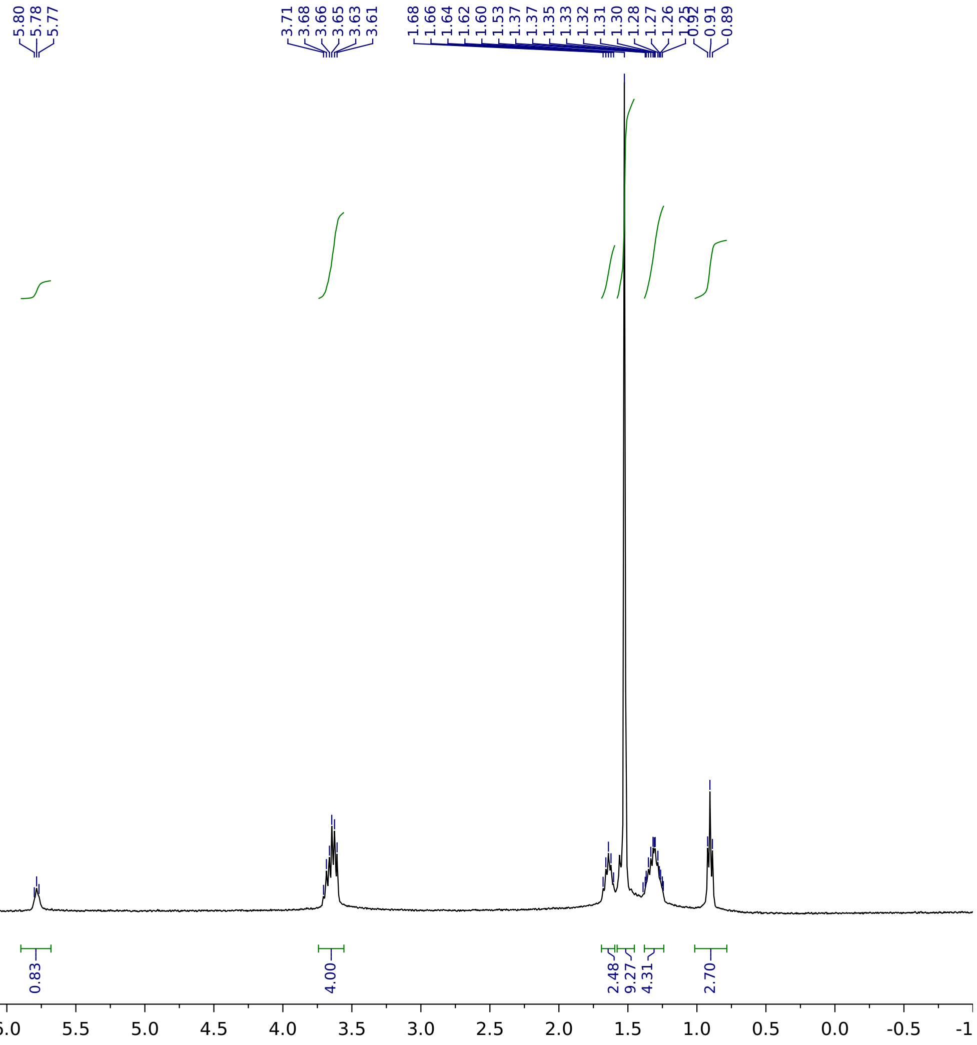

^{1}H NMR (400 MHz, $CDCl_3$) for *N*-*tert*-butoxycarbonyl-*N'*-(2,2,2-trfiluoroethyl)-*N*-pentylsulfamide (**S8v**)

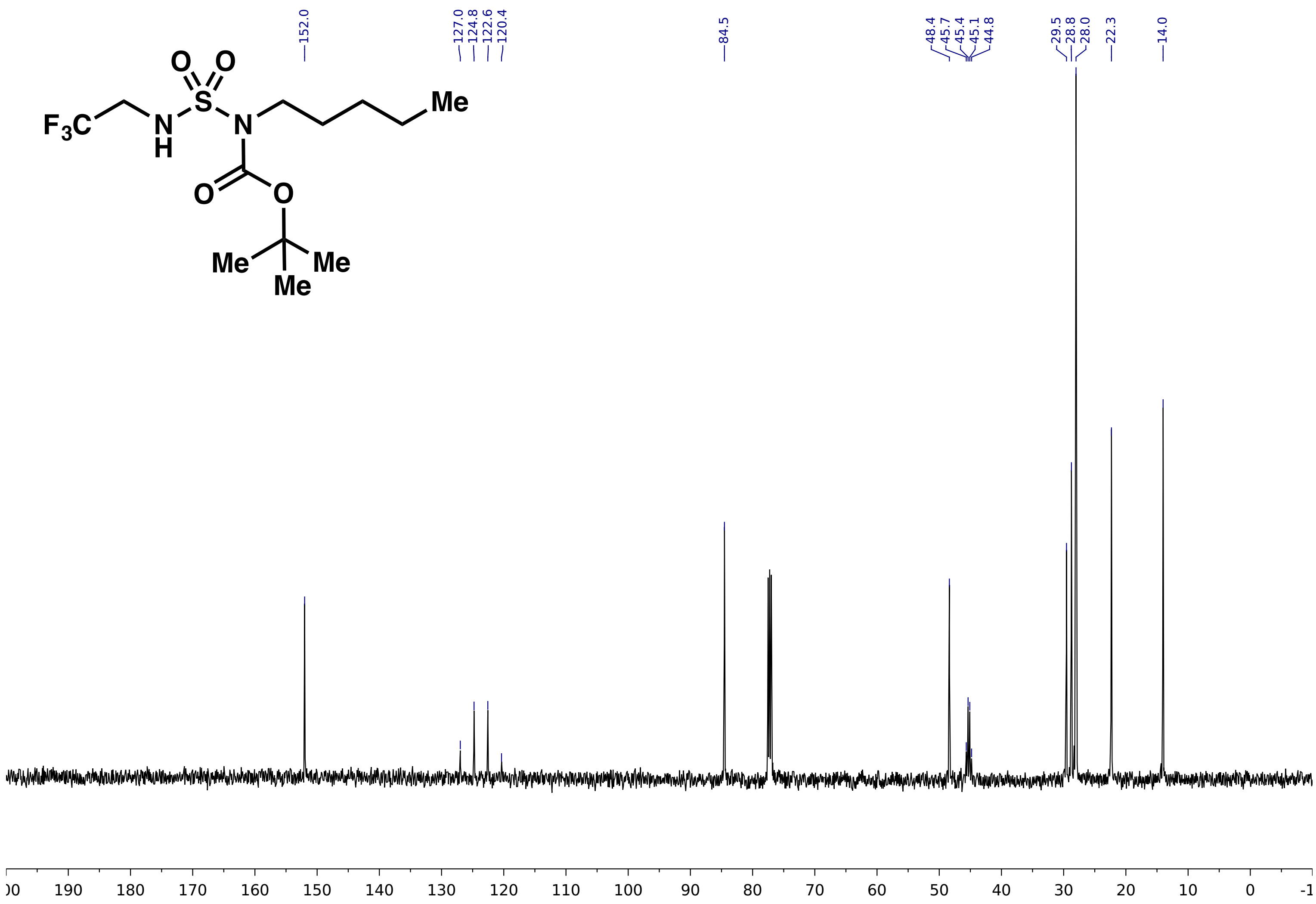

^{13}C NMR (126 MHz, $CDCl_3$) for *N*-*tert*-butoxycarbonyl-*N'*-(2,2,2-trfiluoroethyl)-*N*-pentylsulfamide (**S8v**)

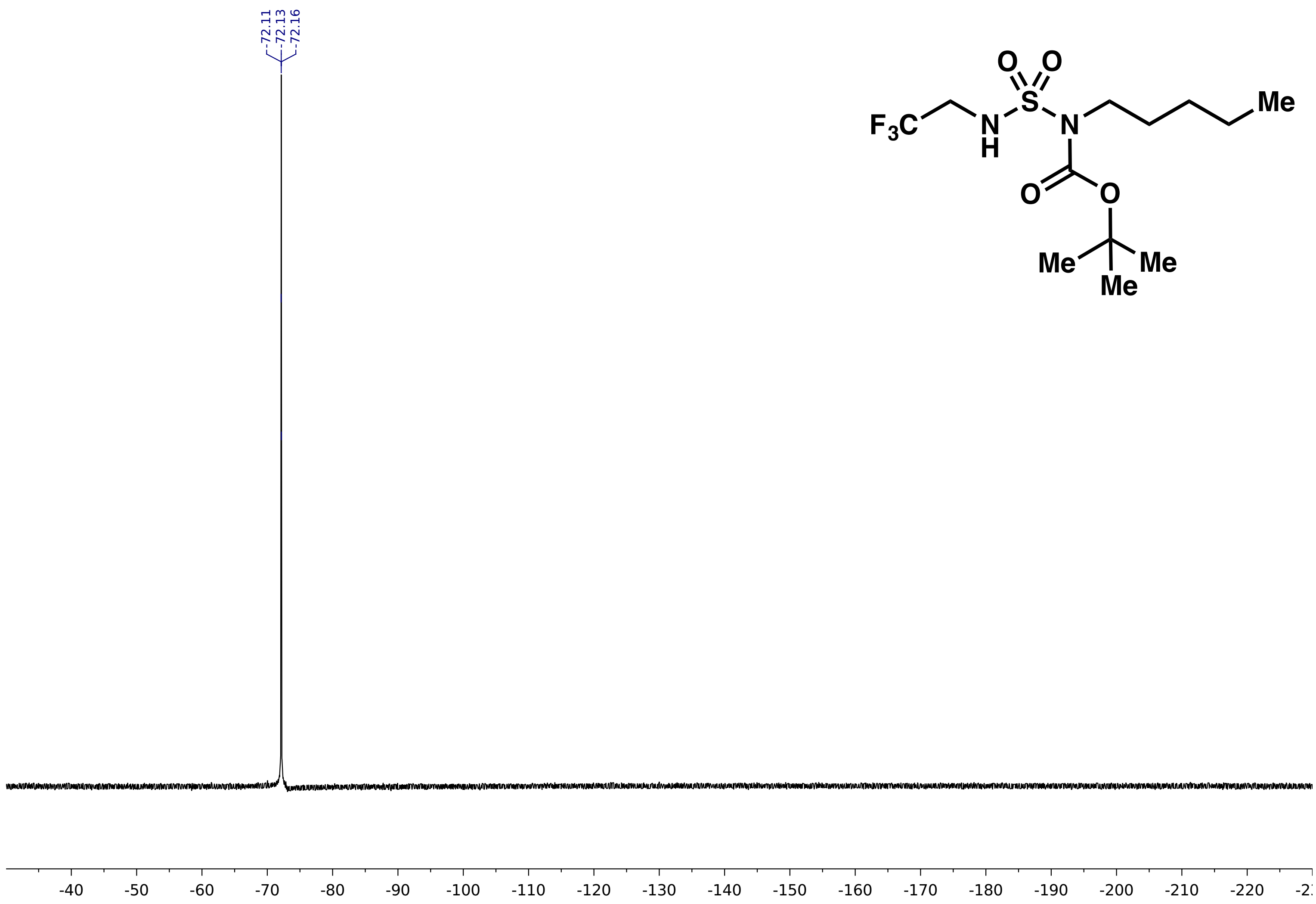

^{19}F NMR (376 MHz, $CDCl_3$) for *N*-*tert*-butoxycarbonyl-*N'*-(2,2,2-trfiluoroethyl)-*N*-pentylsulfamide (**S8v**)

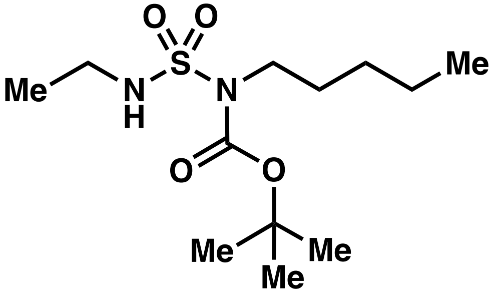

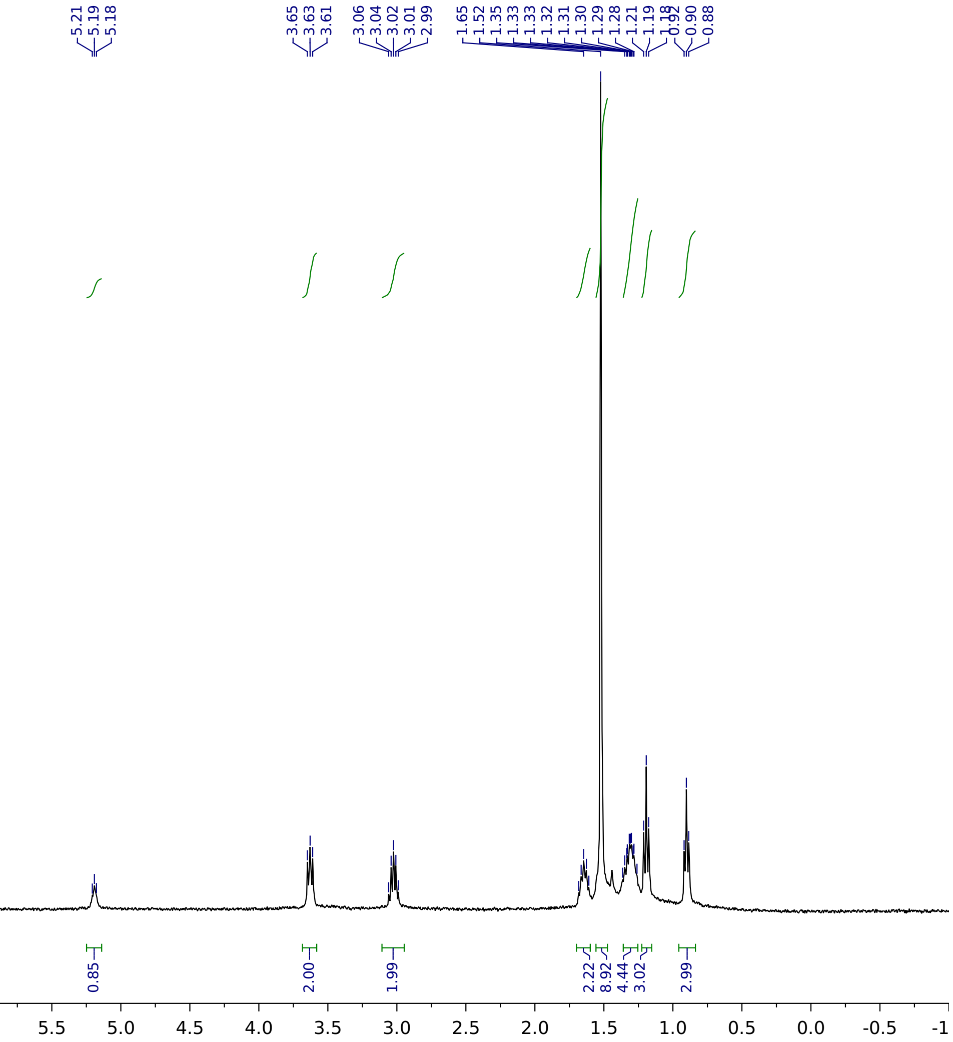

^{1}H NMR (400 MHz, $CDCl_3$) for *N*-*tert*-butoxycarbonyl-*N'*-ethyl-*N*-pentylsulfamide (**S8u**)

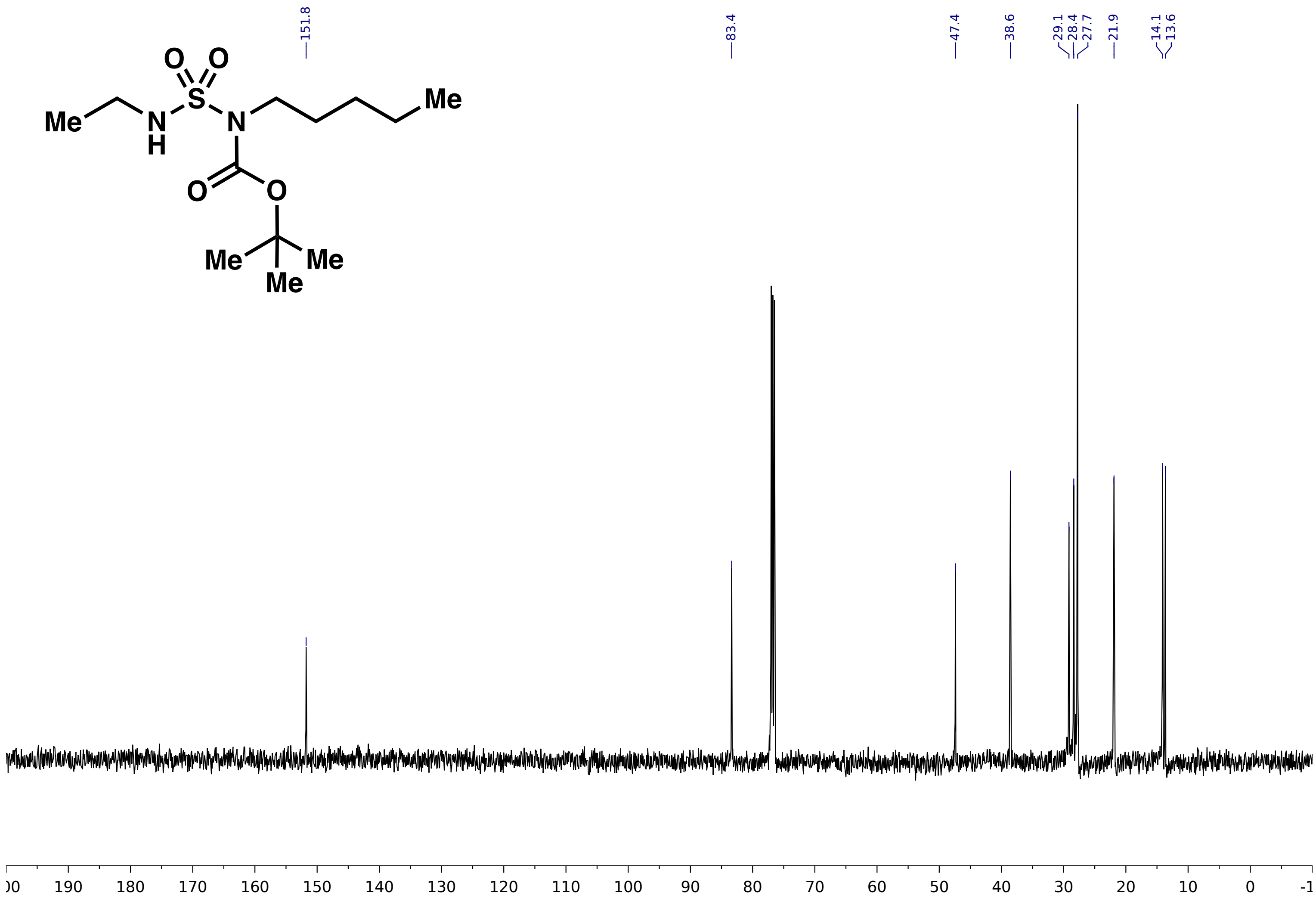

^{13}C NMR (126 MHz, $CDCl_3$) for *N*-*tert*-butoxycarbonyl-*N'*-ethyl-*N*-pentylsulfamide (**S8u**)

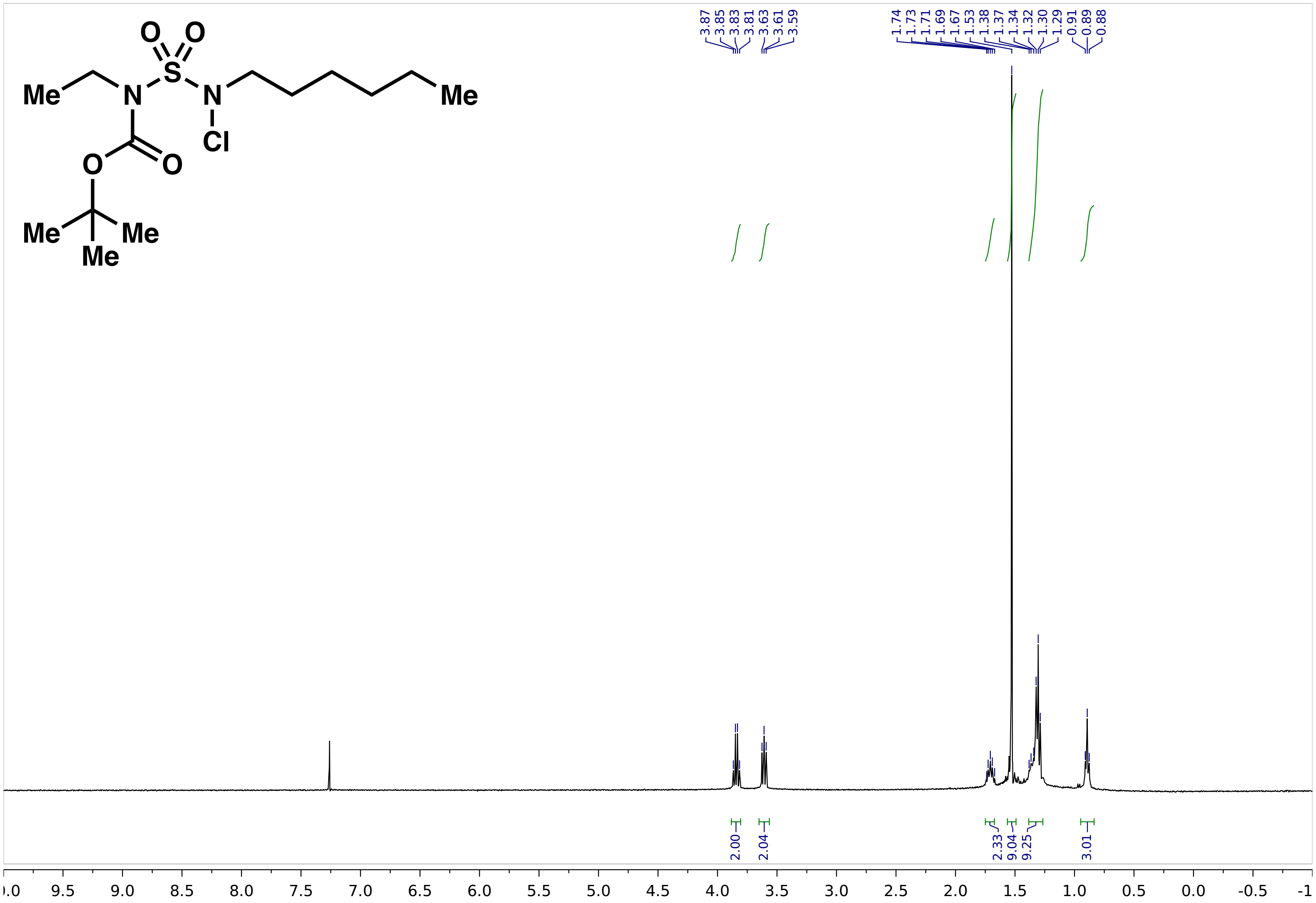

^{1}H NMR (400 MHz, $CDCl_3$) for *N*-*tert*-butoxycarbonyl-*N*-ethyl-*N*'-chloro-*N'*-hexylsulfamide (**5a**)

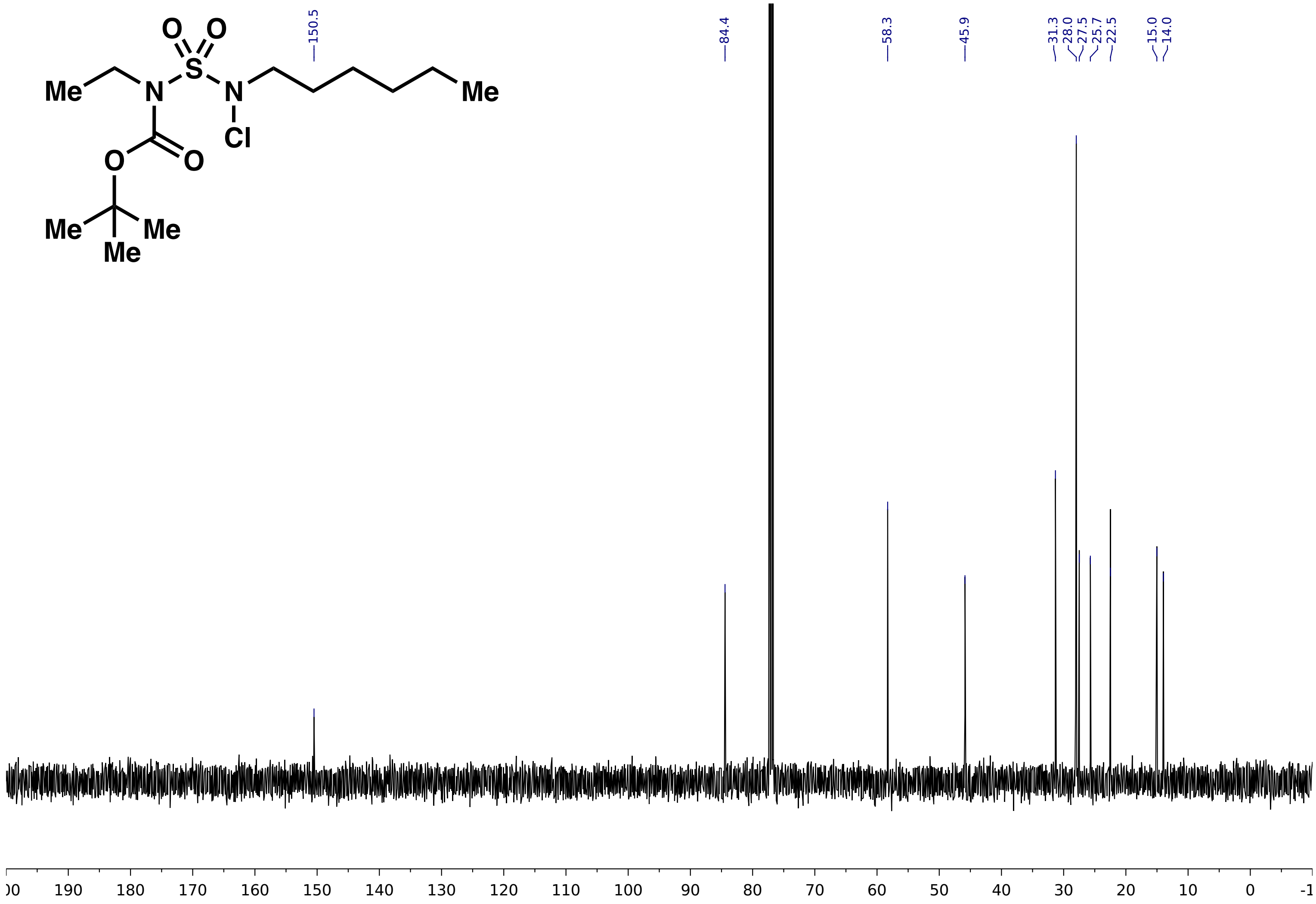

^{13}C NMR (126 MHz, $CDCl_3$) for *N*-*tert*-butoxycarbonyl-*N*-ethyl-*N*'-chloro-*N*'-hexylsulfamide (**5a**)

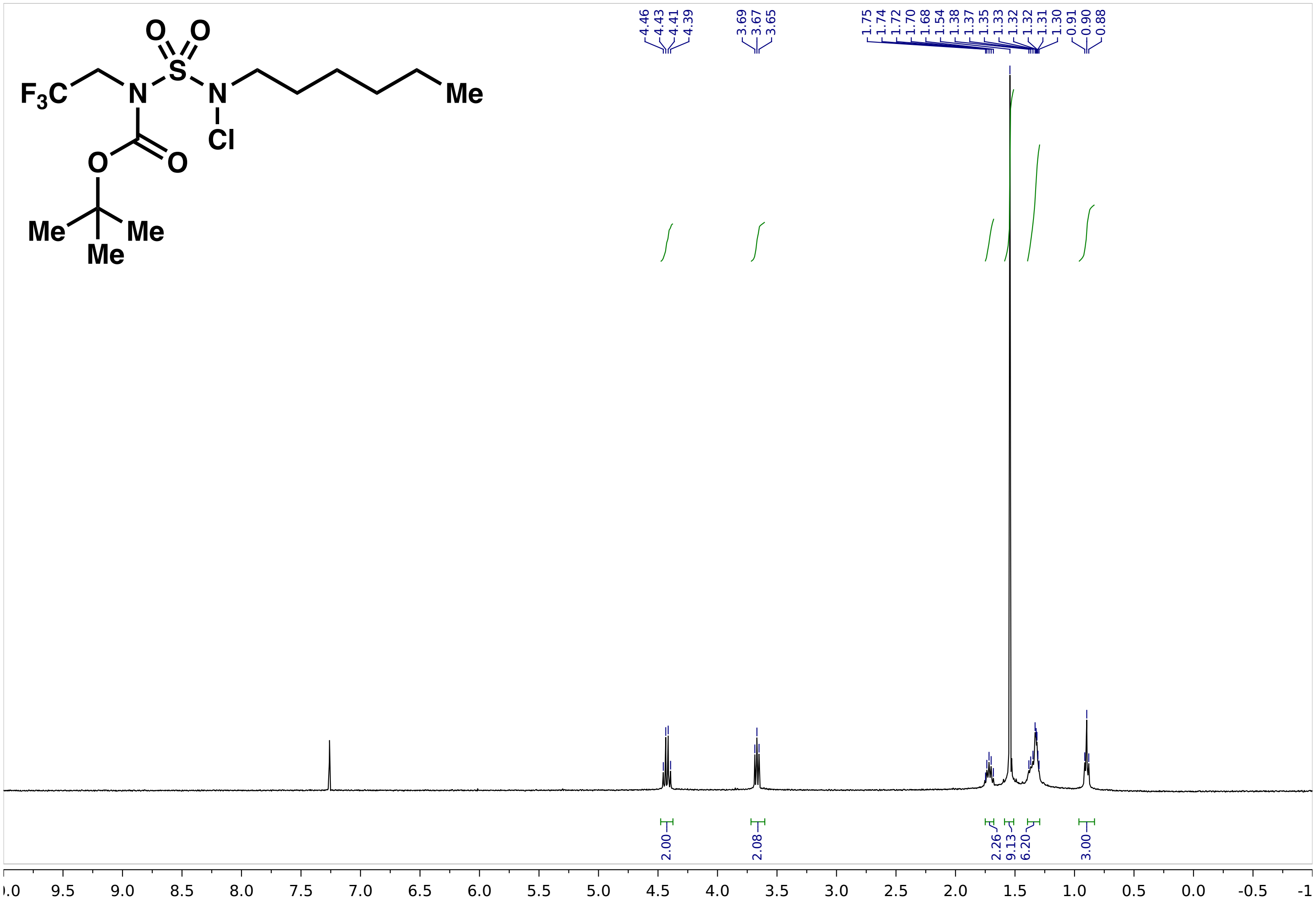

^{1}H NMR (400 MHz, $CDCl_3$) for *N*-*tert*-butoxycarbonyl-*N*’-chloro-*N’*-hexyl-*N*-(2,2,2-trifluoro)ethylsulfamide (**5b**)

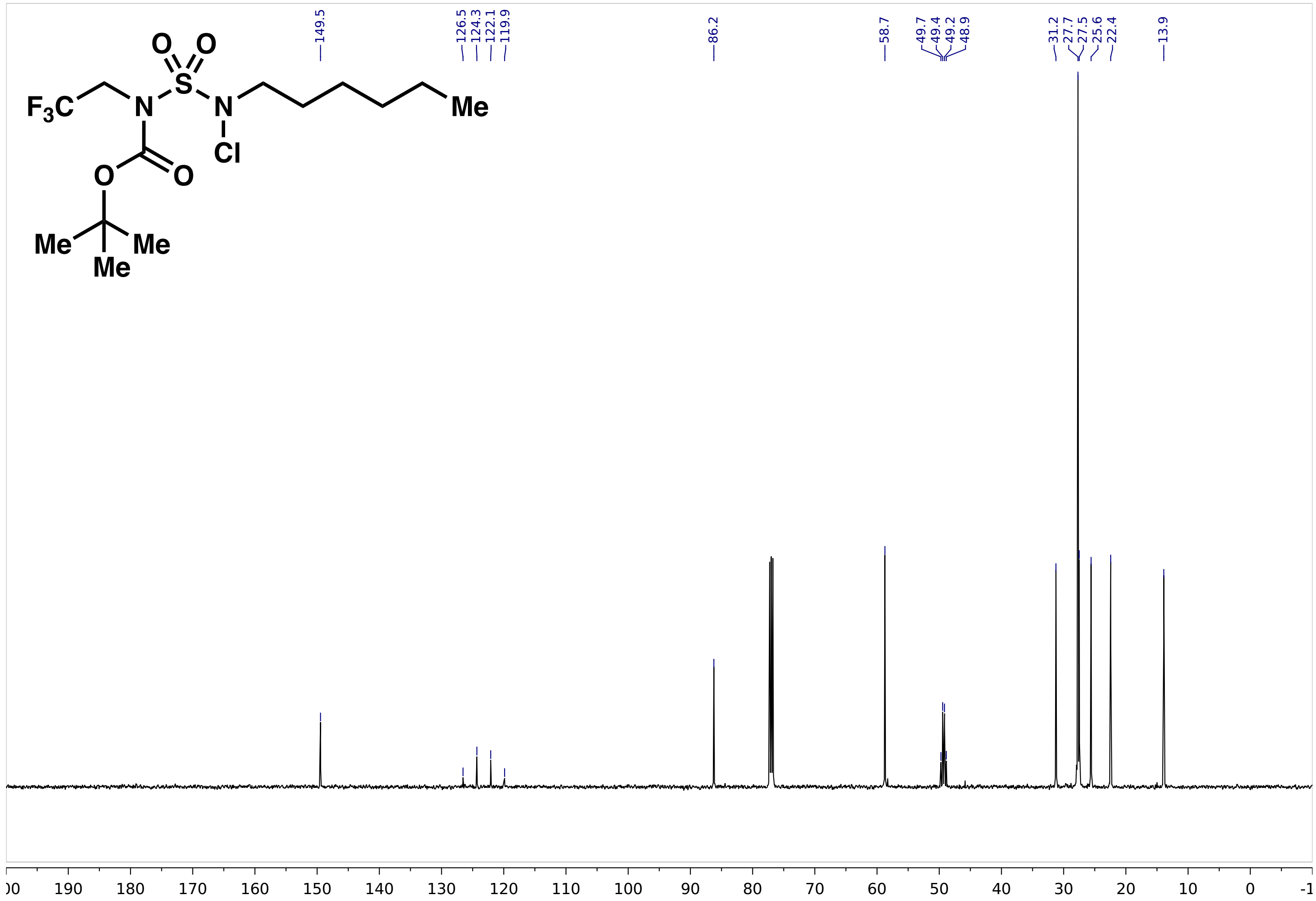

^{13}C NMR (126 MHz, $CDCl_3$) for *N*-*tert*-butoxycarbonyl-*N*'-chloro-*N'*-hexyl-*N*-(2,2,2-trifluoro)ethylsulfamide (**5b**)

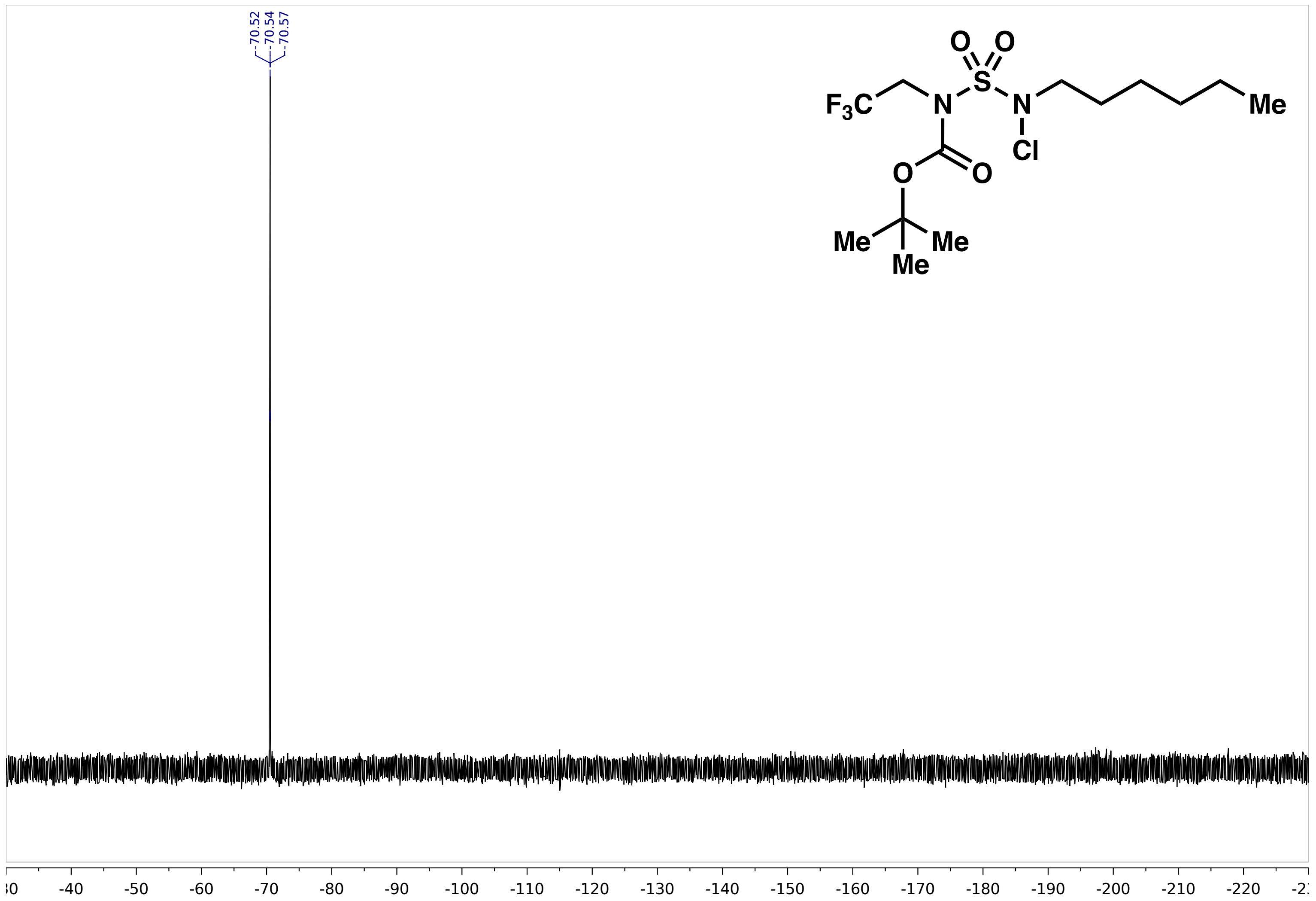

^{19}F NMR (376 MHz, $CDCl_3$) for *N*-*tert*-butoxycarbonyl-*N*'-chloro-*N'*-hexyl-*N*-(2,2,2-trifluoro)ethylsulfamide (**5b**)

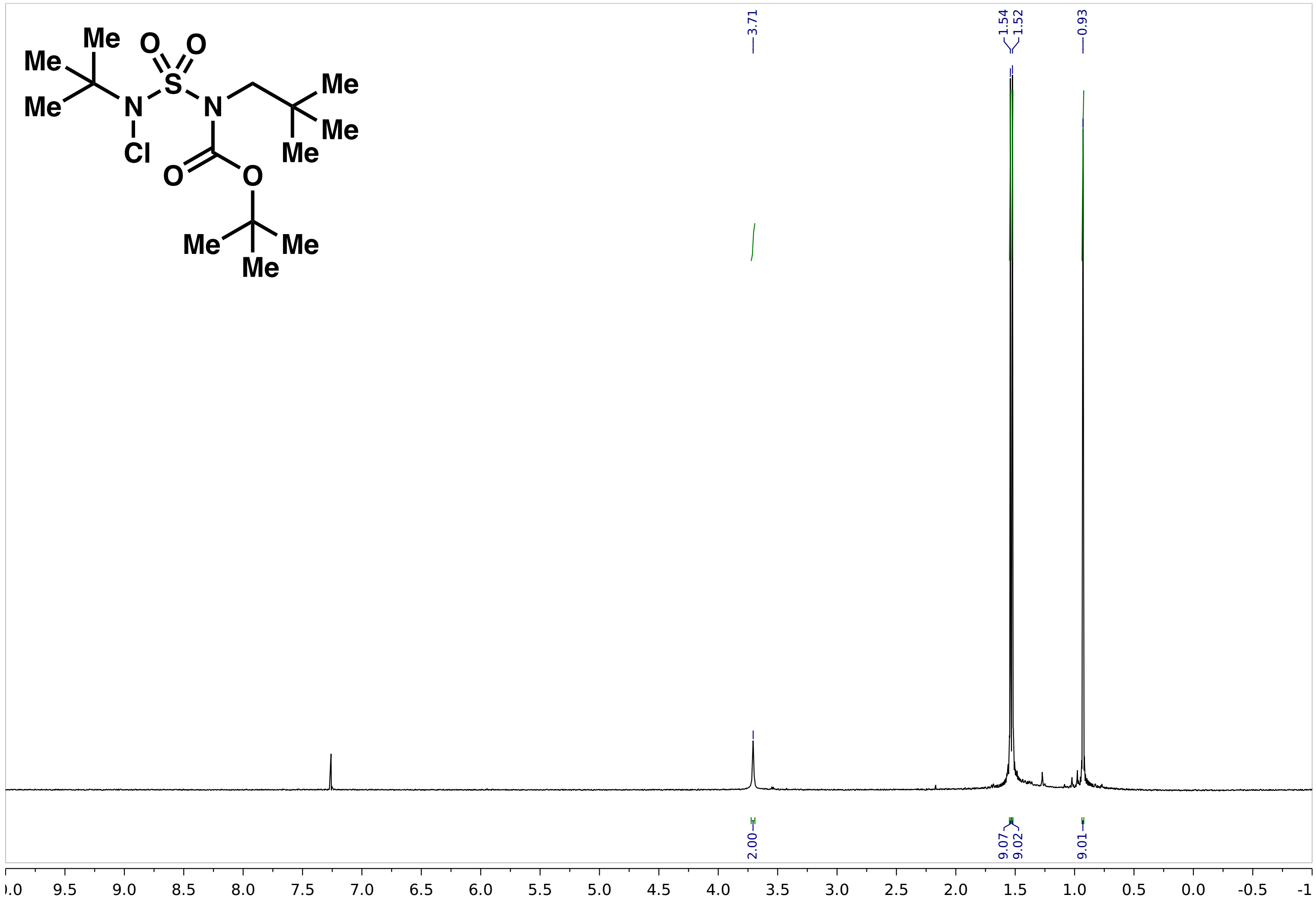

^{1}H NMR (400 MHz, $CDCl_3$) for *N*-*tert*-butoxycarbonyl-*N'*-*tert*-butyl-*N'*-chloro-*N*-neopentylsulfamide (**1a**)

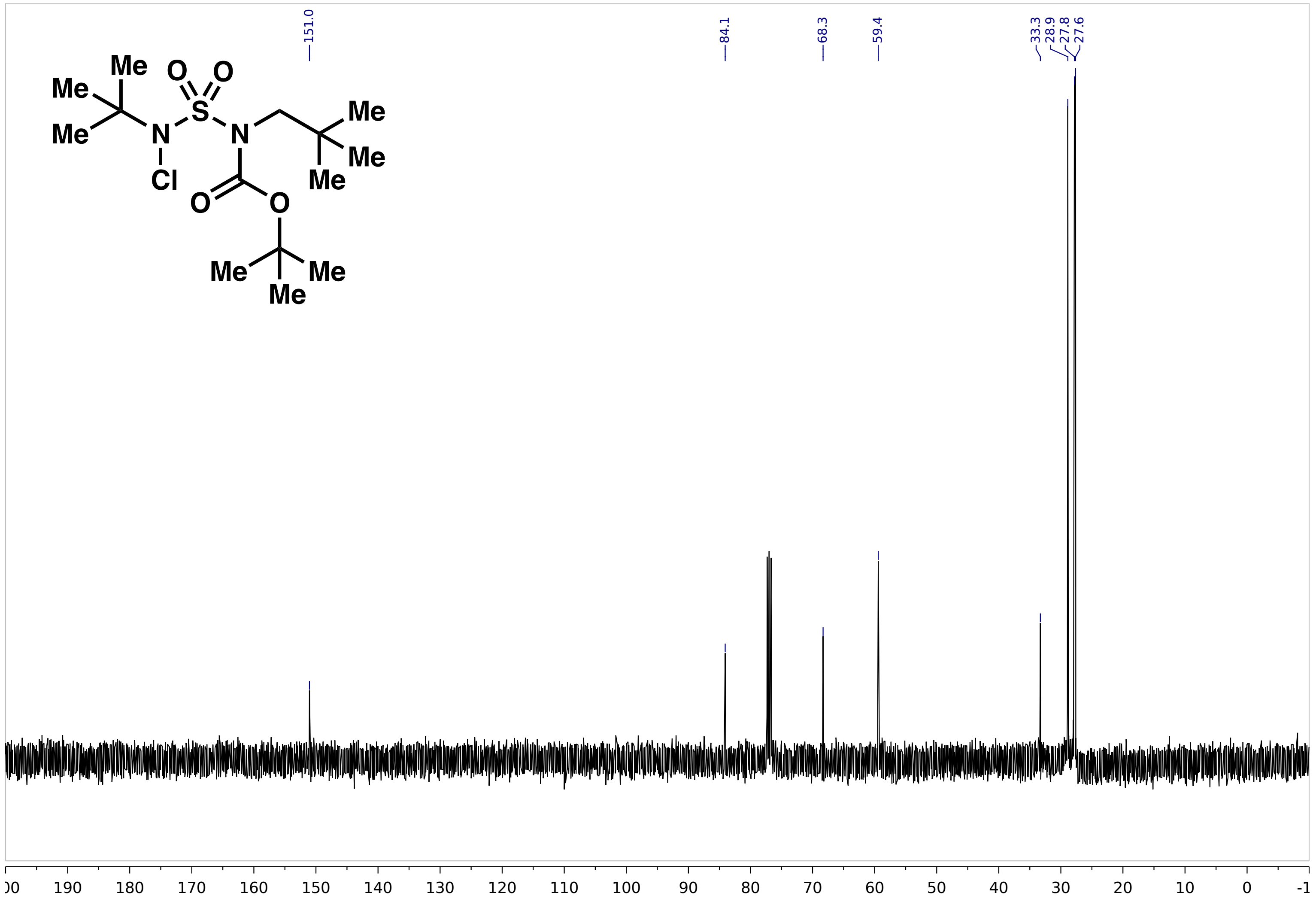

^{13}C NMR (101 MHz, $CDCl_3$) for *N*-*tert*-butoxycarbonyl-*N'*-*tert*-butyl-*N'*-chloro-*N*-neopentylsulfamide (**1a**)

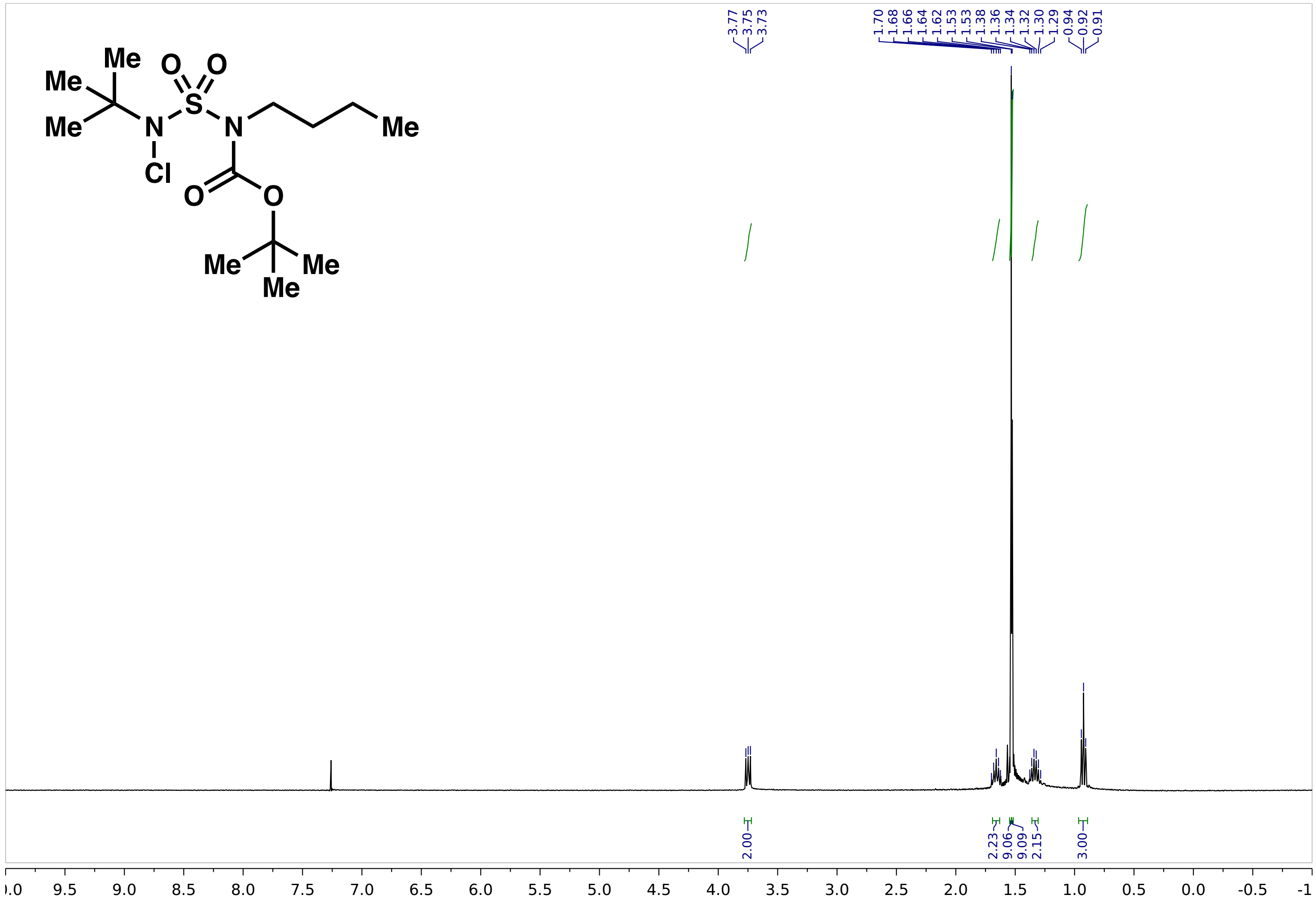

^{1}H NMR ($CDCl_3$, 400 MHz) for *N*-*tert*-butoxycarbonyl-*N*-butyl-*N'*-*tert*-butyl-*N'*-chlorosulfamide (**1b**)

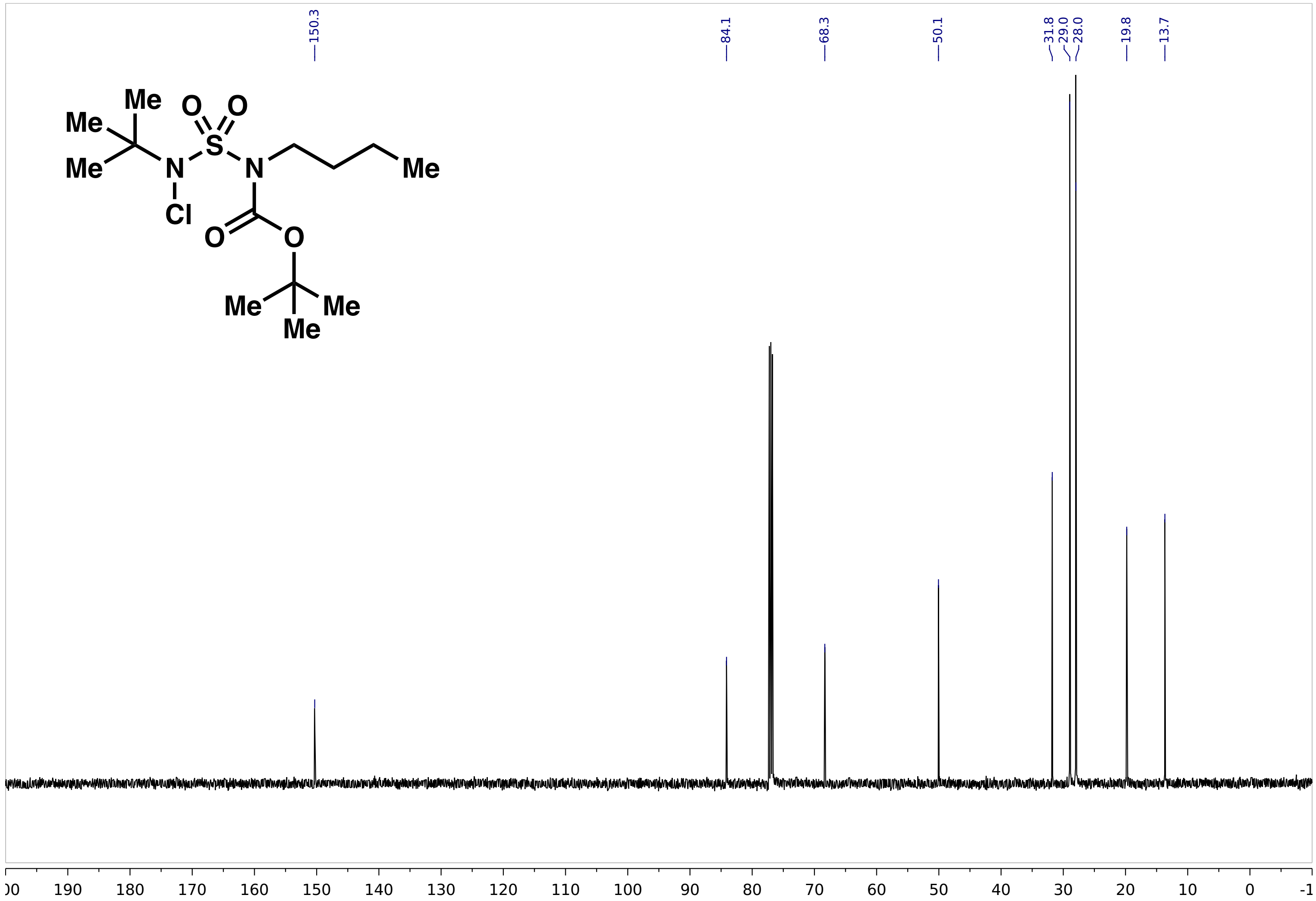

^{13}C NMR ($CDCl_3$, 126 MHz) for *N*-*tert*-butoxycarbonyl-*N*-butyl-*N'*-*tert*-butyl-*N*'-chlorosulfamide (**1b**)

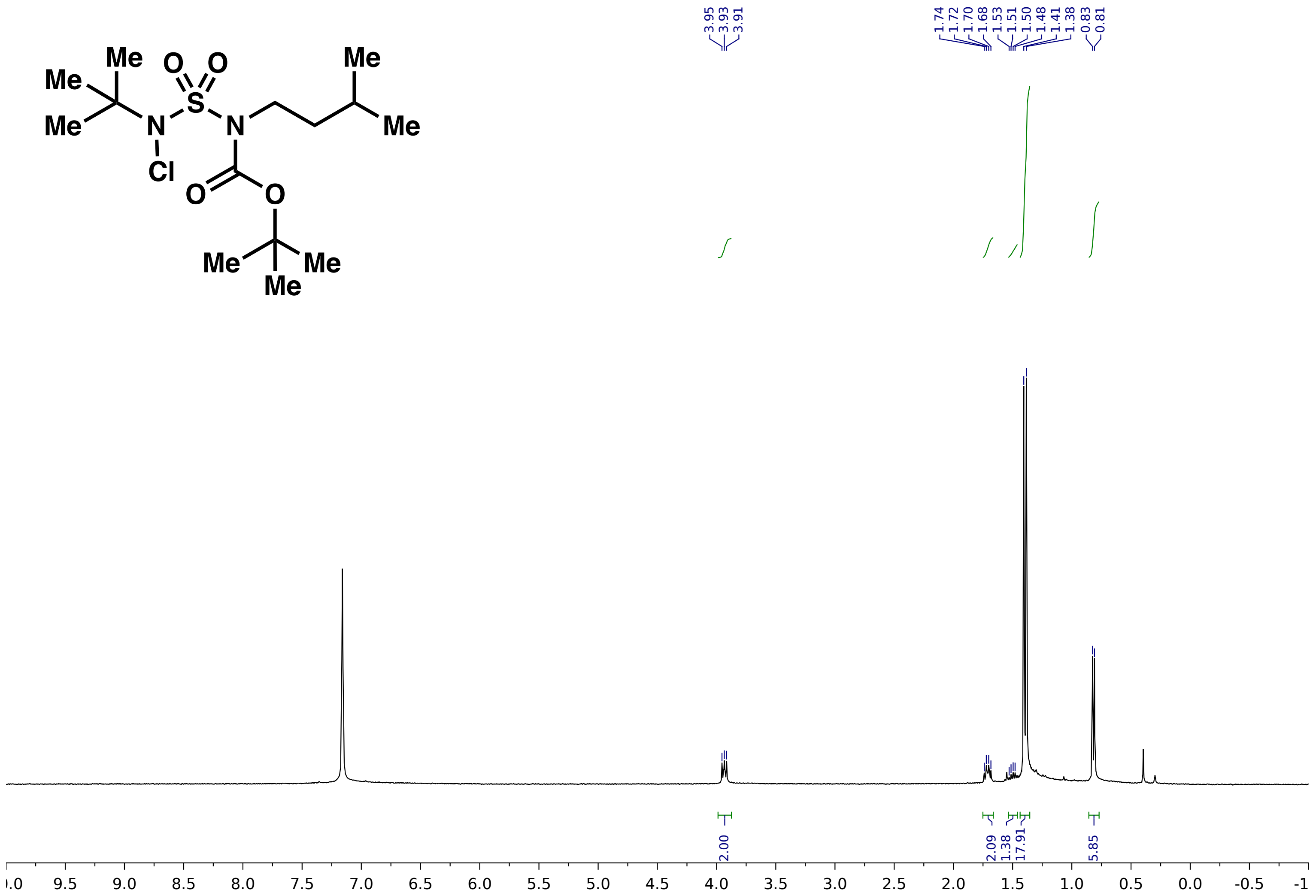

^{1}H NMR (C_6D_6, 400 MHz) for *N*-*tert*-butoxycarbonyl-*N'*-*tert*-butyl-*N'*-chloro-*N*-isopentylsulfamide (**1c**)

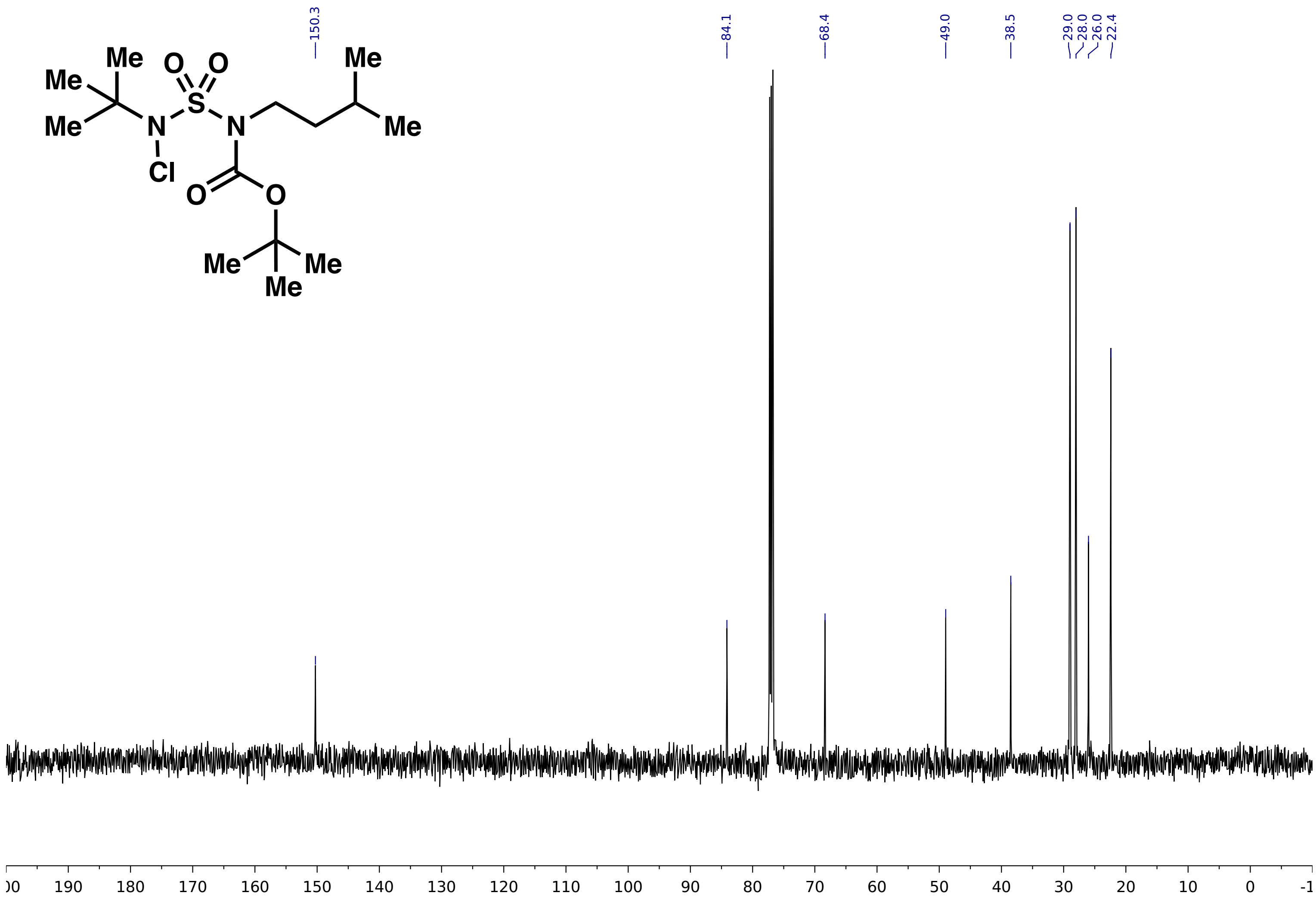

^{13}C NMR ($CDCl_3$, 126 MHz) for *N*-*tert*-butoxycarbonyl-*N'*-*tert*-butyl-*N'*-chloro-*N*-isopentylsulfamide (**1c**)

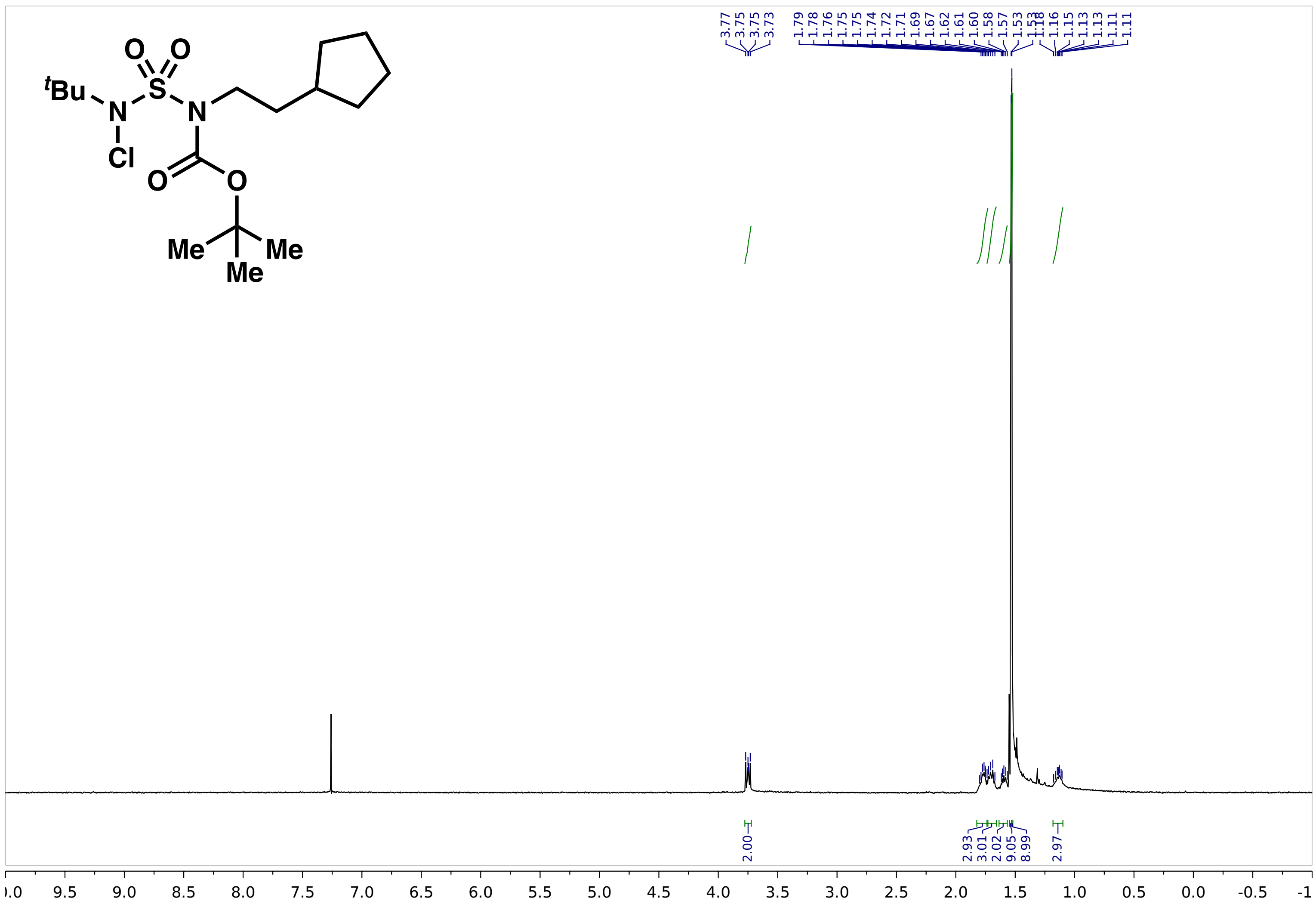

^{1}H NMR (400 MHz, $CDCl_3$) for *N*-*tert*-butoxycarbonyl-*N'*-*tert*-butyl-*N'*-chloro-*N*-(2-(cyclopentyl)ethyl)sulfamide (**1d**)

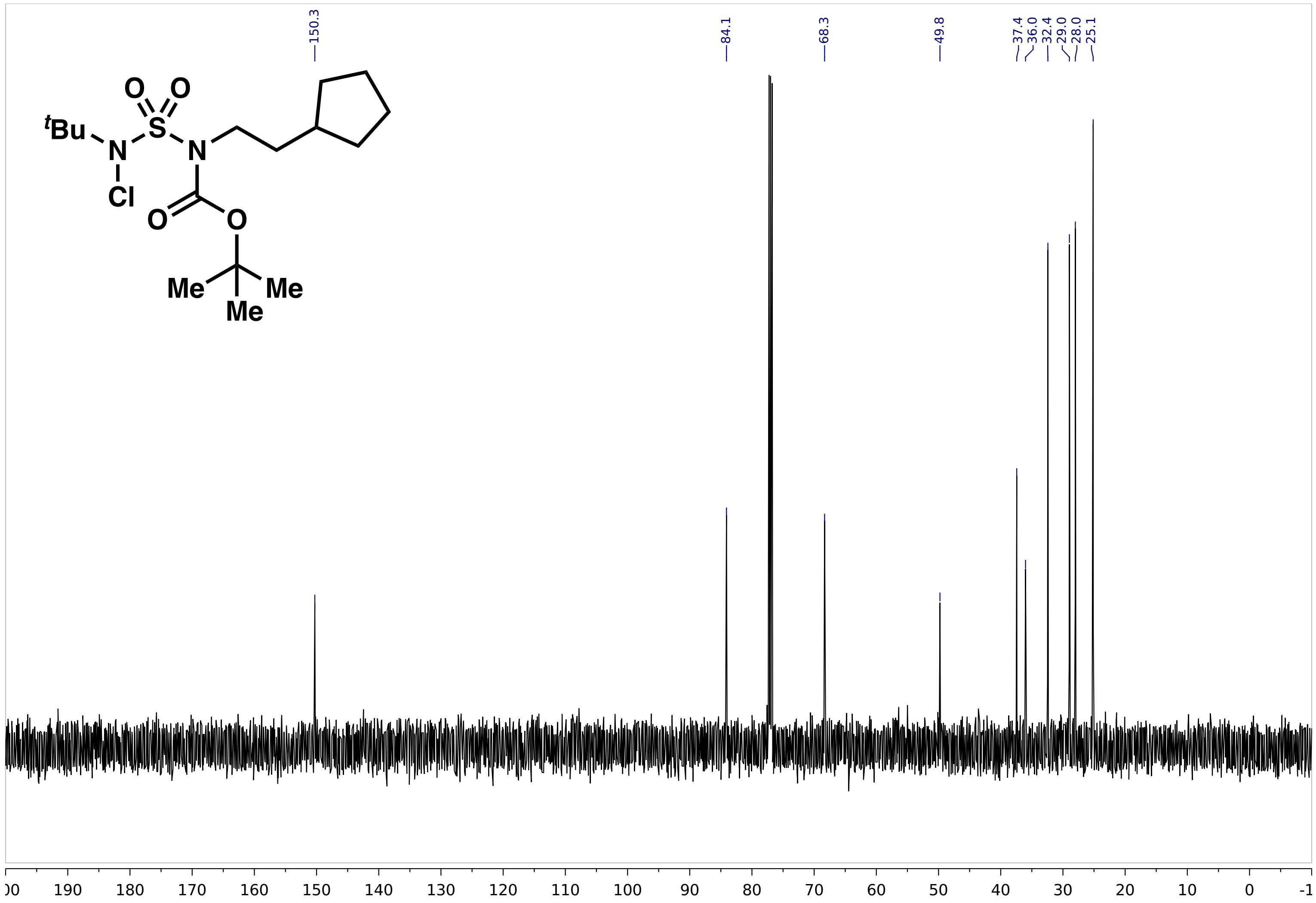

^{13}C NMR (126 MHz, $CDCl_3$) for *N*-*tert*-butoxycarbonyl-*N'*-*tert*-butyl-*N'*-chloro-*N*-(2-(cyclopentyl)ethyl)sulfamide (**1d**)

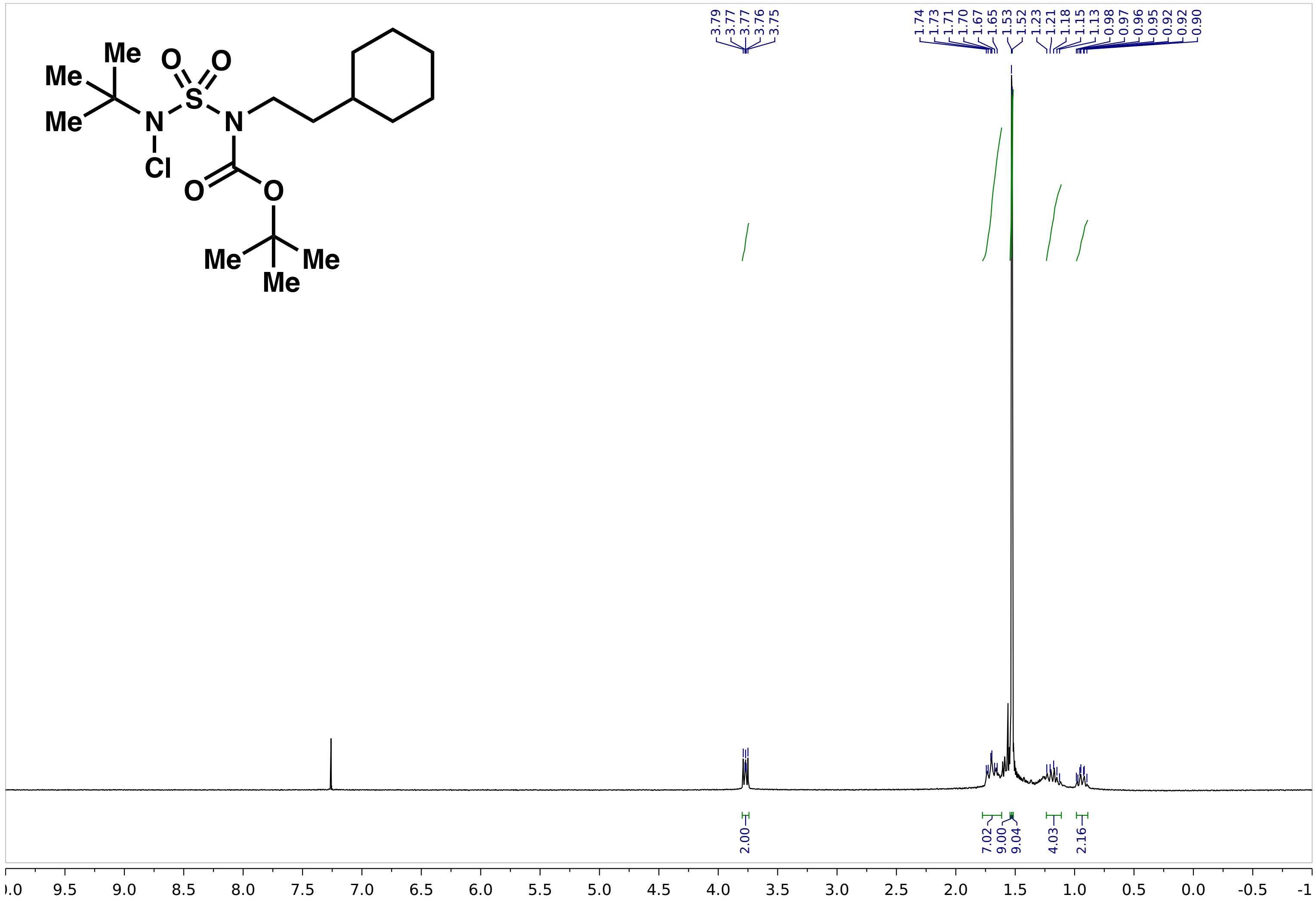

^{1}H NMR (400 MHz, $CDCl_3$) for *N*-*tert*-butoxycarbonyl-*N'*-*tert*-butyl-*N'*-chloro-*N*-(2-(cyclohexyl)ethyl)sulfamide (**1e**)

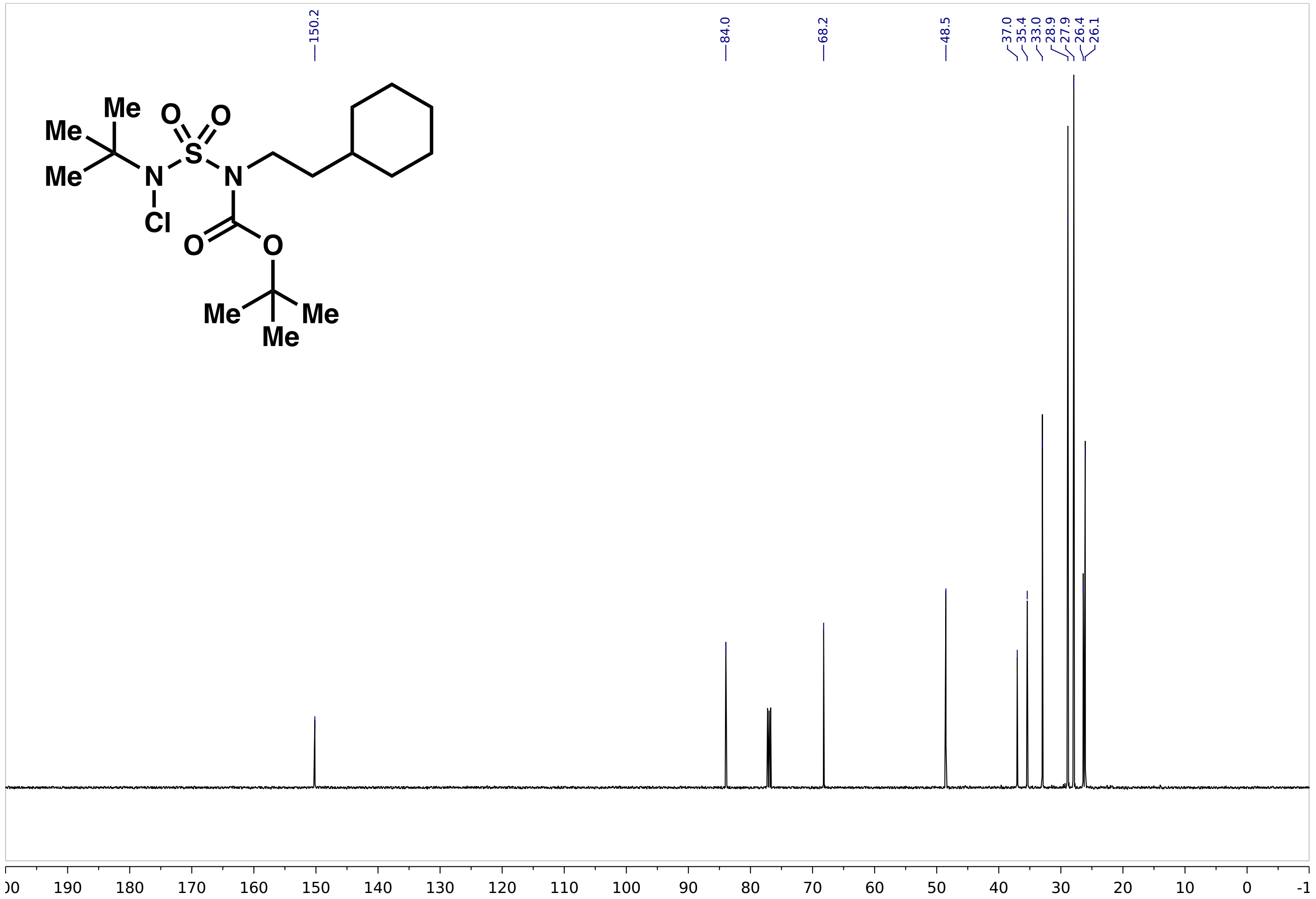

^{13}C NMR (126 MHz, $CDCl_3$) for *N*-*tert*-butoxycarbonyl-*N'*-*tert*-butyl-*N'*-chloro-*N*-(2-(cyclohexyl)ethyl)sulfamide (**1e**)

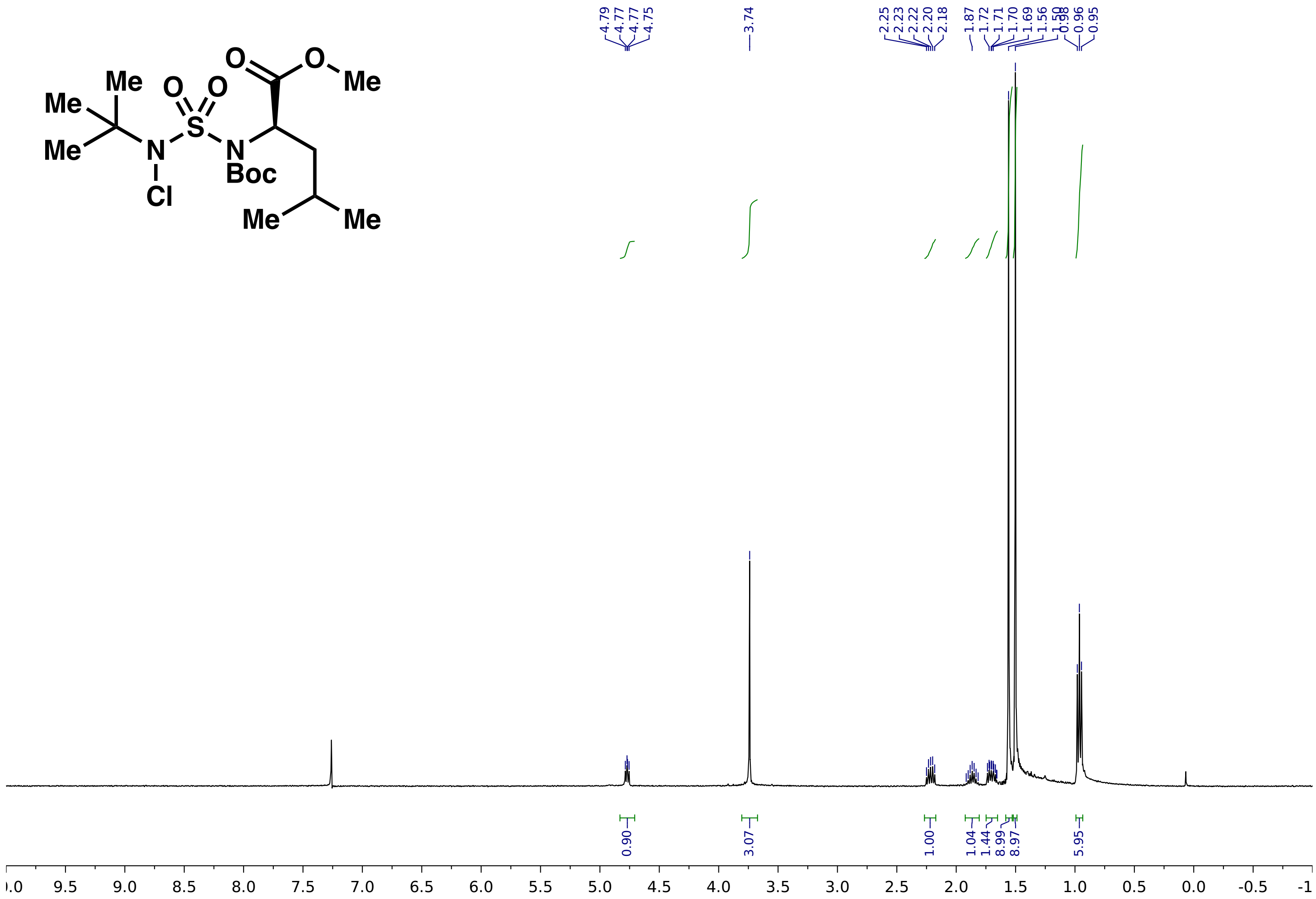

^{1}H NMR (400 MHz, $CDCl_3$) for *N*-*tert*-butoxycarbonyl-*N'*-*tert*-butyl-*N'*-chloro-*N*-((*2R*)-methyl 4-methylpentano-2-yl)sulfamide (**1f**)

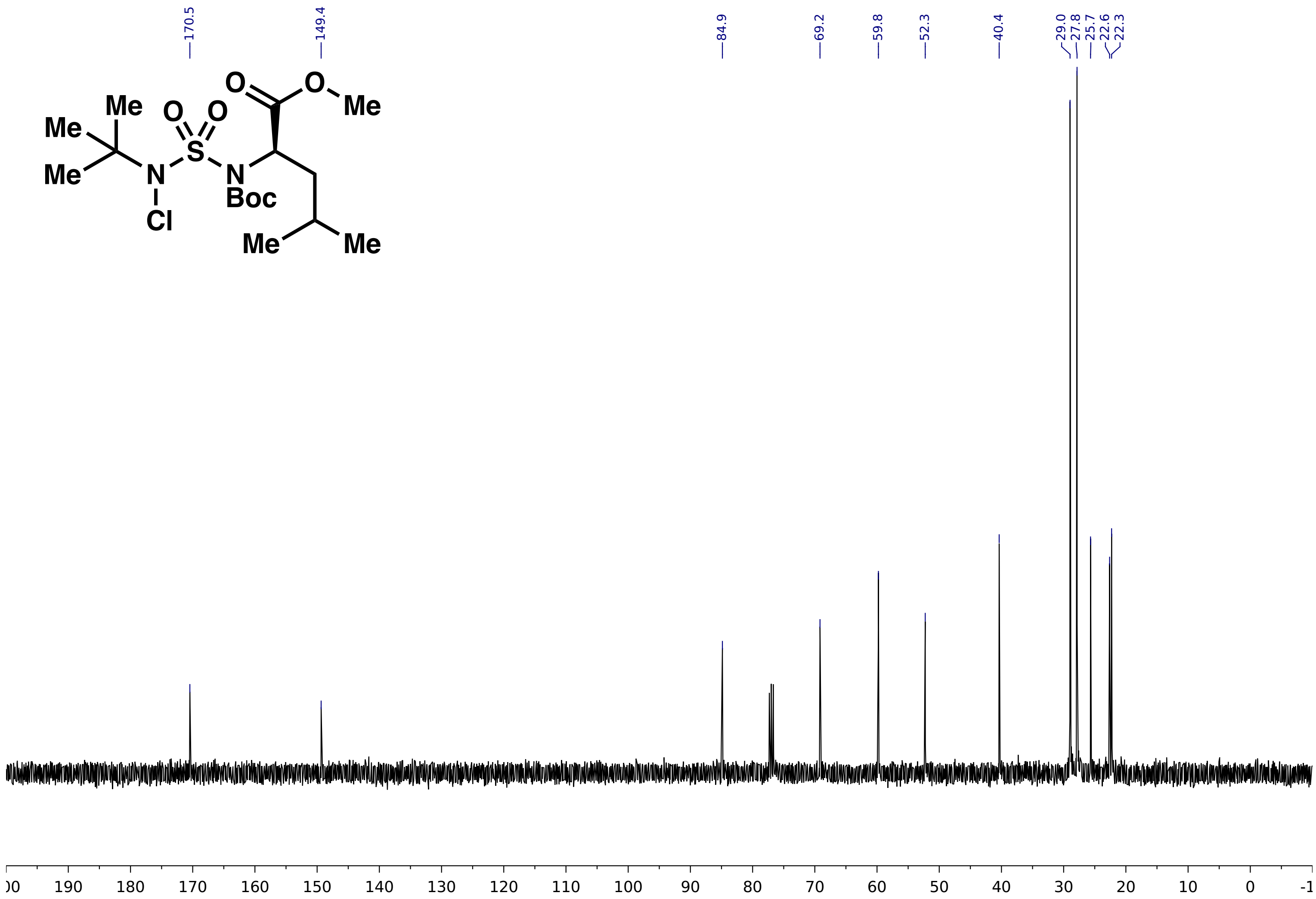

^{13}C NMR (101 MHz, $CDCl_3$) for *N*-*tert*-butoxycarbonyl-*N'*-*tert*-butyl-*N'*-chloro-*N*-((*2R*)-methyl 4-methylpentano-2-yl)sulfamide (**1f**)

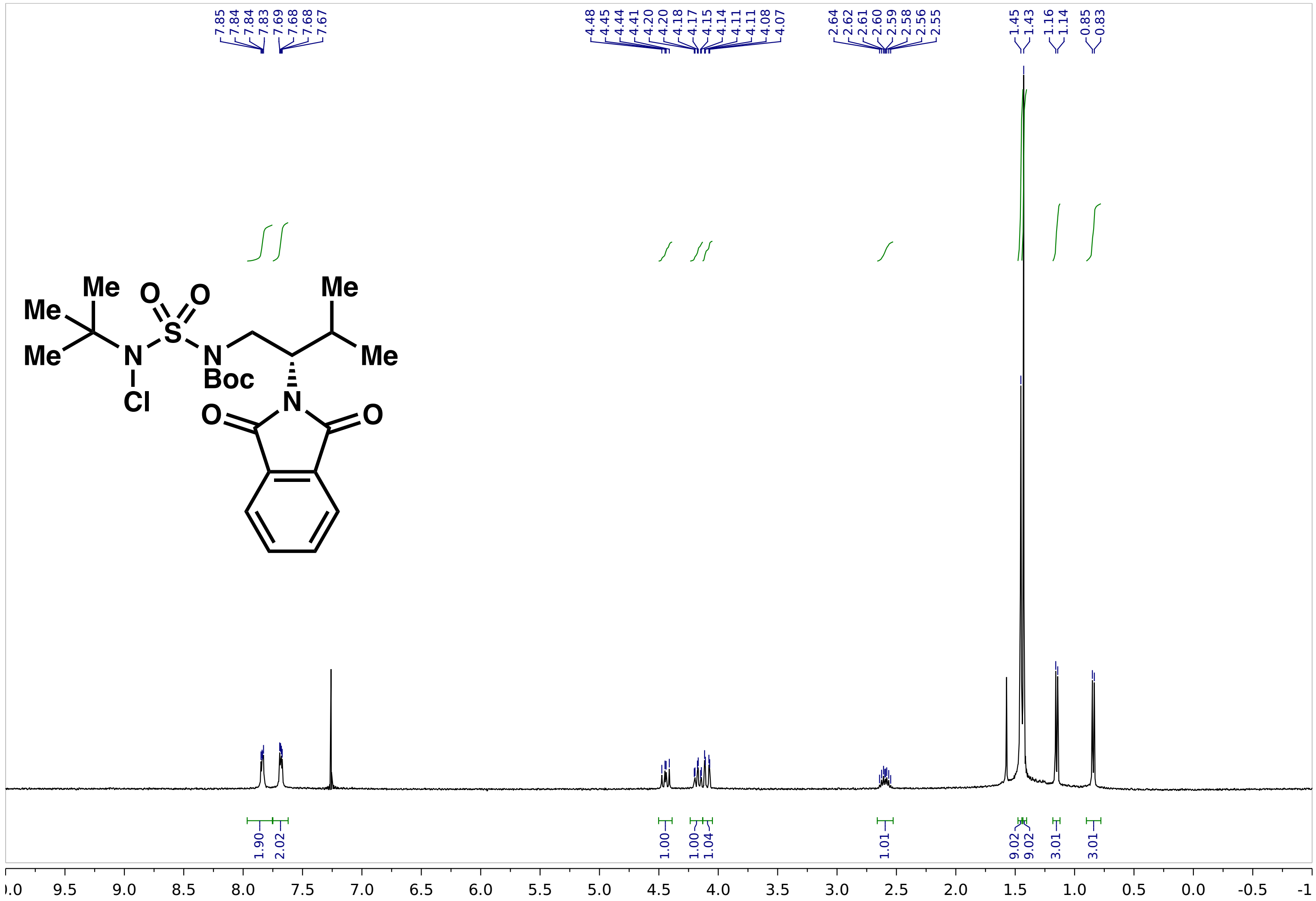

^{1}H NMR (400 MHz, $CDCl_3$) for *N*-*tert*-butoxycarbonyl-*N*'-*tert*-butyl-*N'*-chloro-*N*-((*S*)-2-(1,3-dioxoisoindolin-2-yl)-3-methylbutyl)sulfamide (**1g**)

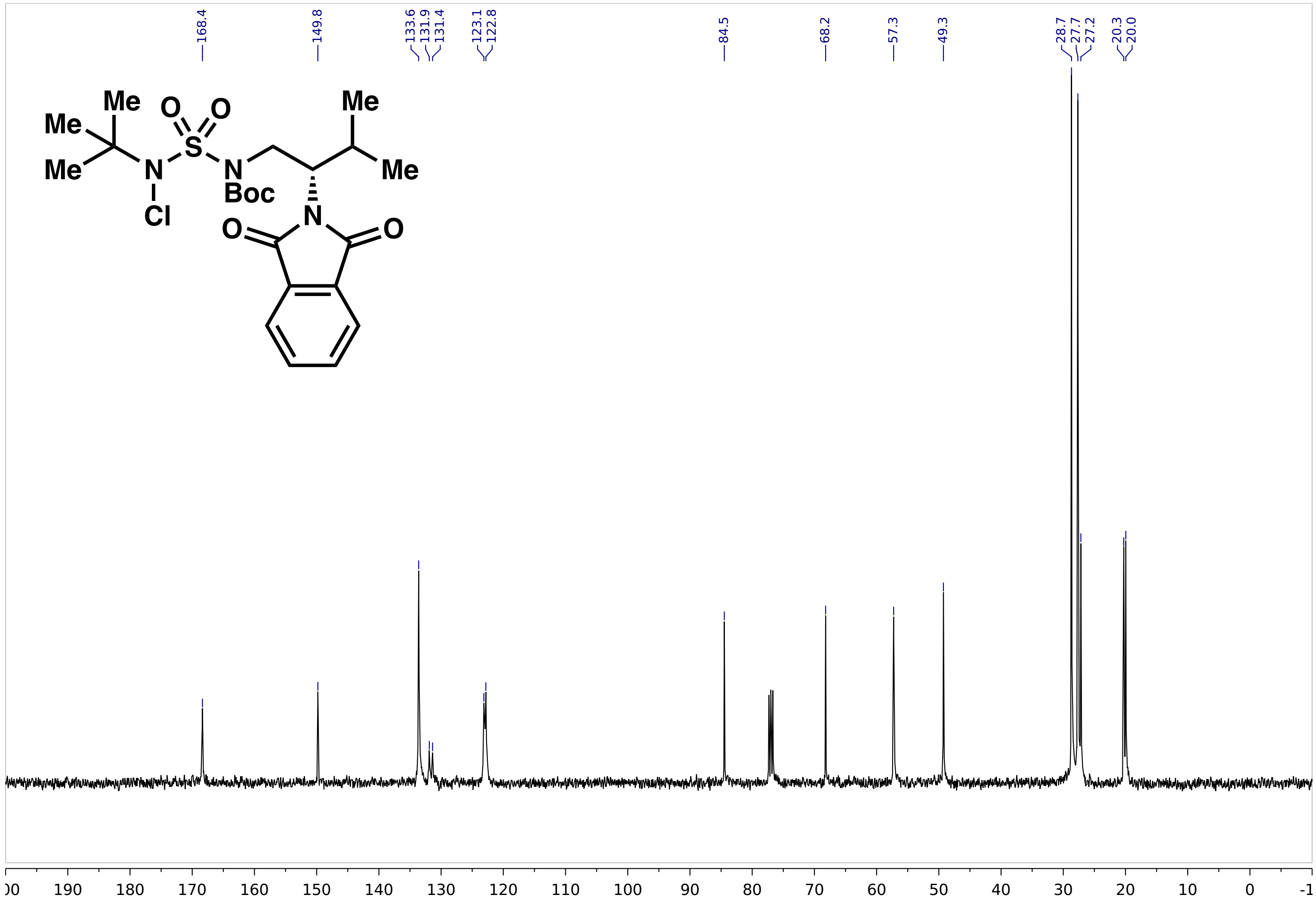

^{13}C NMR (101 MHz, $CDCl_3$) for *N*-*tert*-butoxycarbonyl-*N*'-*tert*-butyl-*N'*-chloro-*N*-((*S*)-2-(1,3-dioxoisoindolin-2-yl)-3-methylbutyl)sulfamide (**1g**)

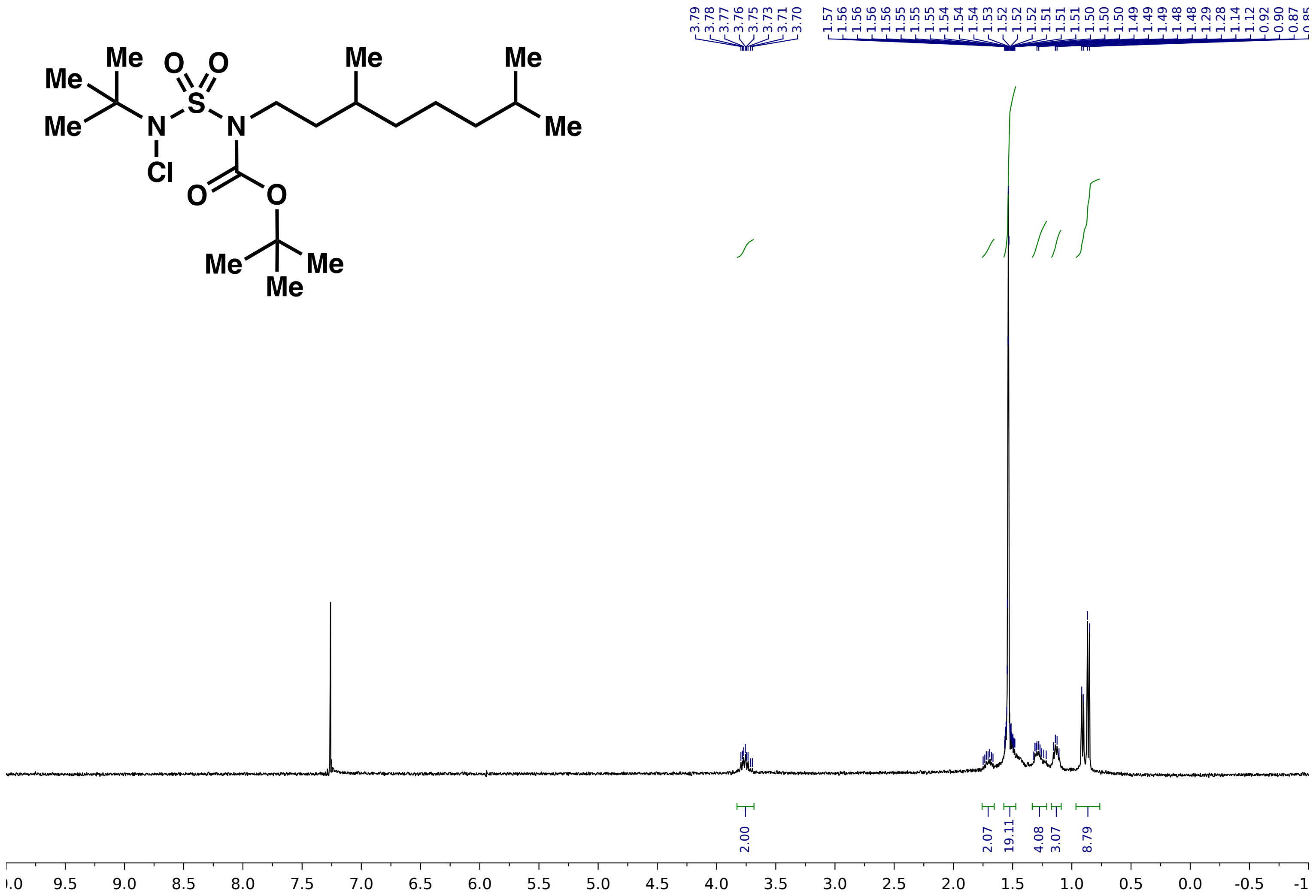

^{1}H NMR (400 MHz, $CDCl_3$) for *N*-*tert*-butoxycarbonyl-*N'*-*tert*-butyl-*N'*-chloro-*N*-(3,7-dimethyloct-1-yl)sulfamide (**1h**)

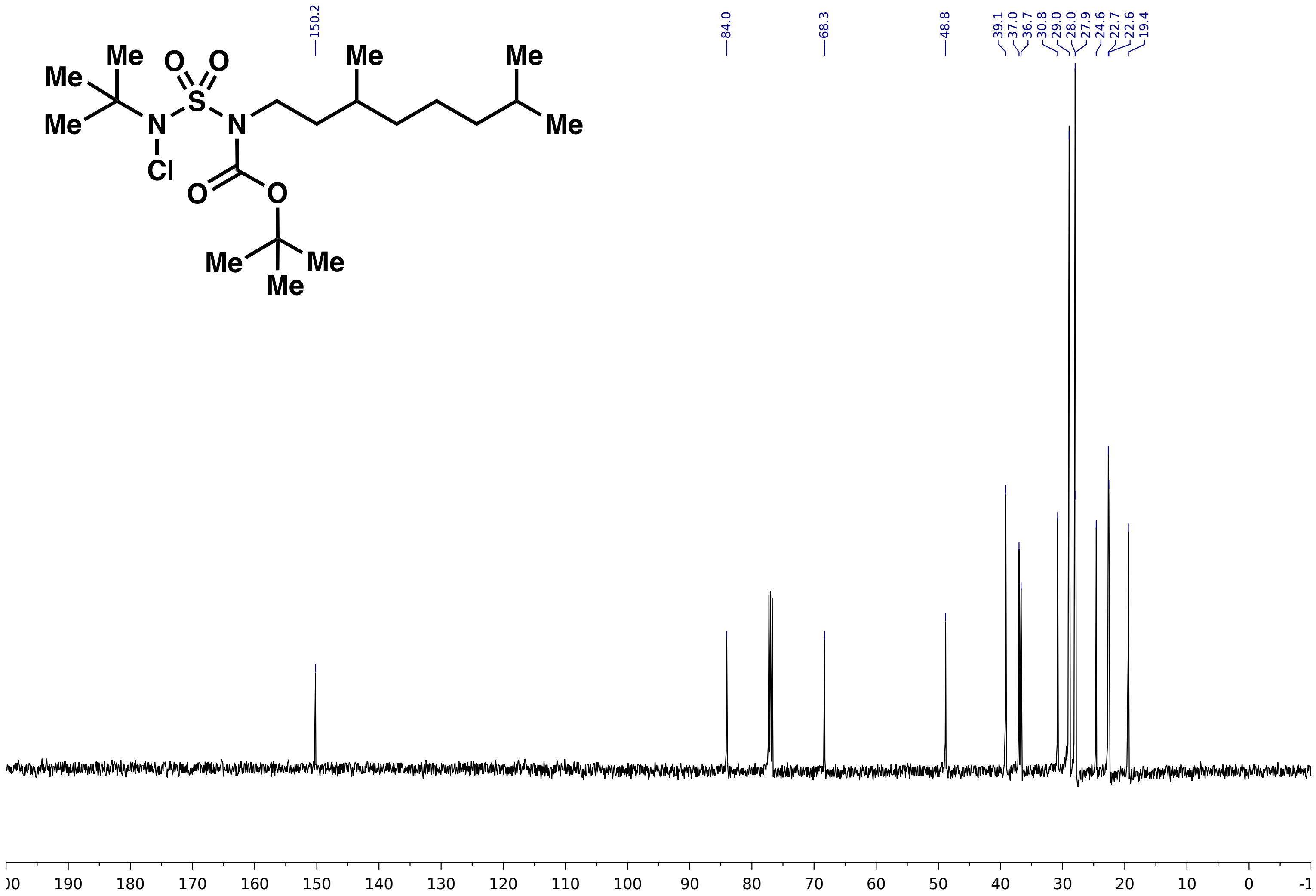

^{13}C NMR (126 MHz, $CDCl_3$) for *N*-*tert*-butoxycarbonyl-*N'*-*tert*-butyl-*N'*-chloro-*N*-(3,7-dimethyloct-1-yl)sulfamide (**1h**)

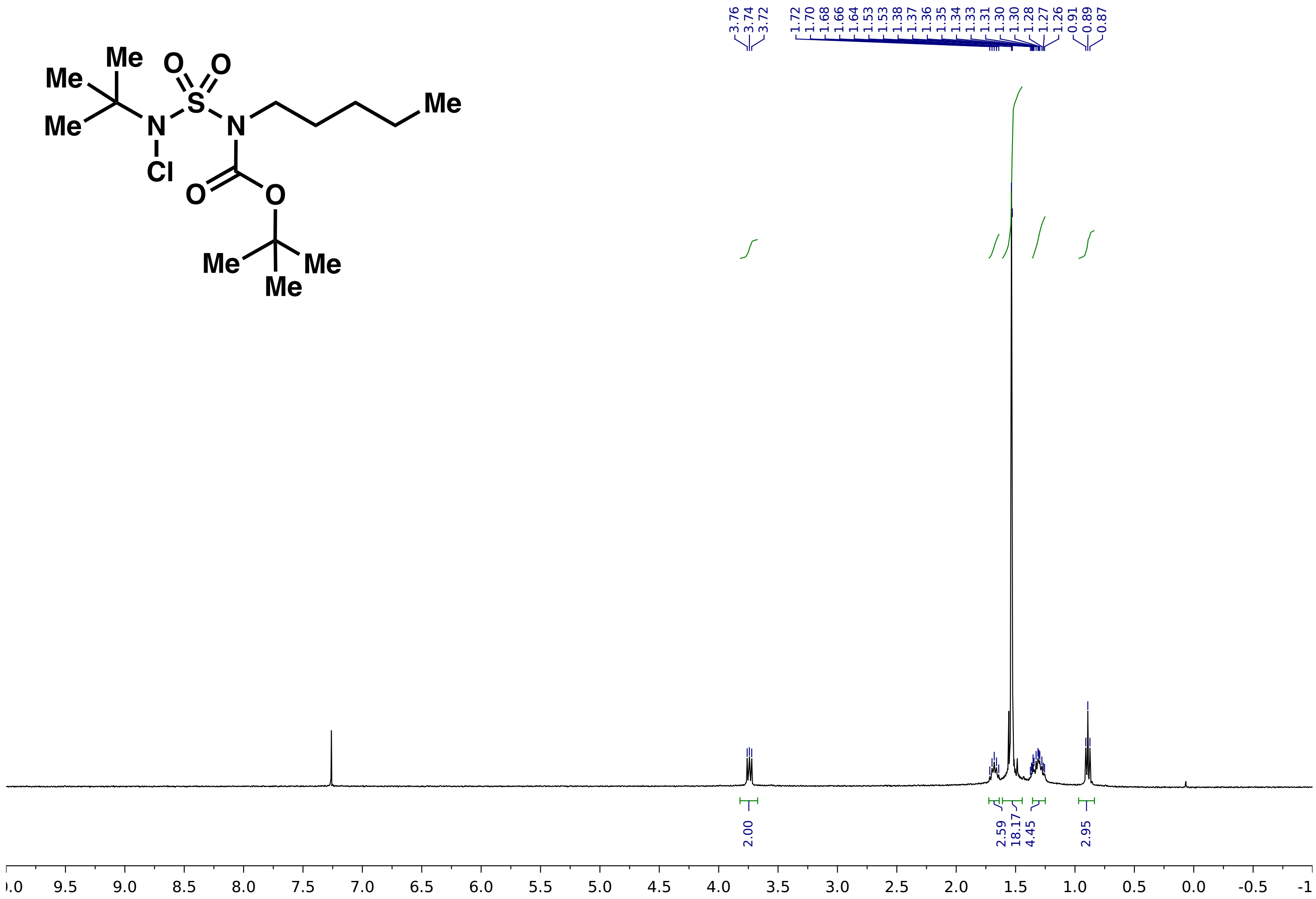

^{1}H NMR (400 MHz, $CDCl_3$) for *N*-*tert*-butoxycarbonyl-*N'*-*tert*-butyl-*N'*-chloro-*N*-pentylsulfamide (**1i**)

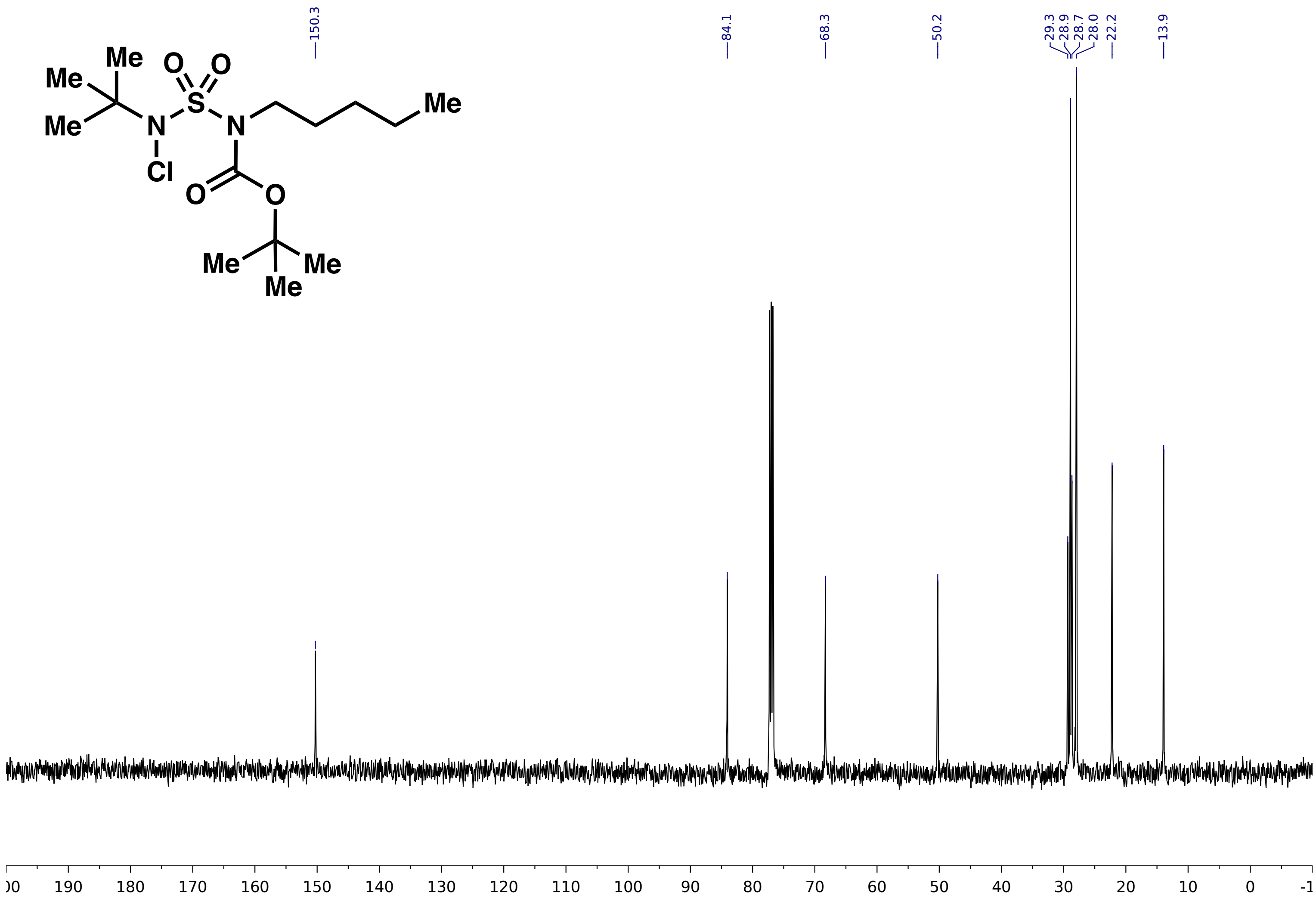

^{13}C NMR (126 MHz, CDCl$_3$) for *N*-*tert*-butoxycarbonyl-*N'*-*tert*-butyl-*N'*-chloro-*N*-pentylsulfamide (**1i**)

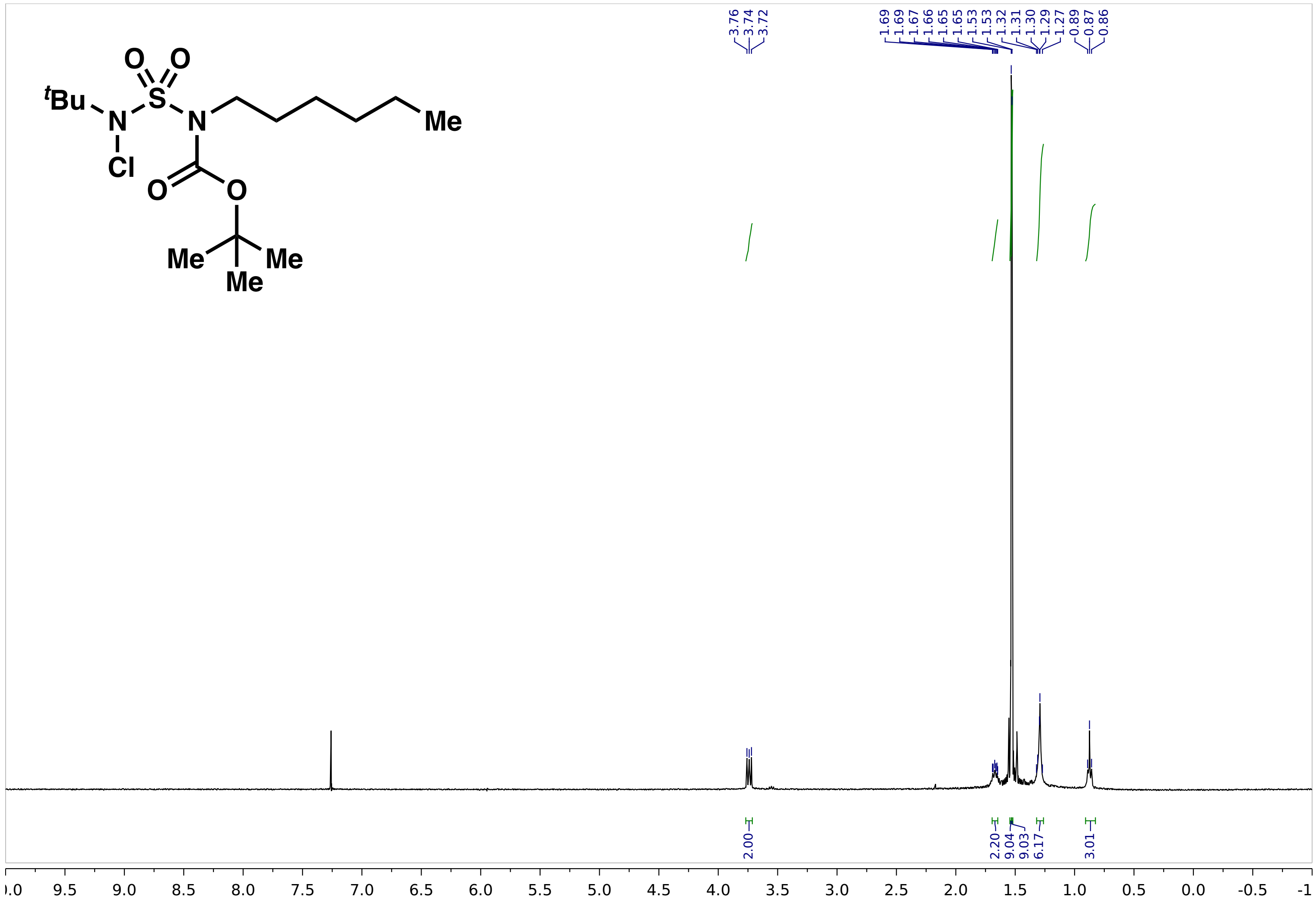

^{1}H NMR (400 MHz, $CDCl_3$) for *N*-*tert*-butoxycarbonyl-*N'*-*tert*-butyl-*N'*-chloro-*N*-hexylsulfamide (**1j**)

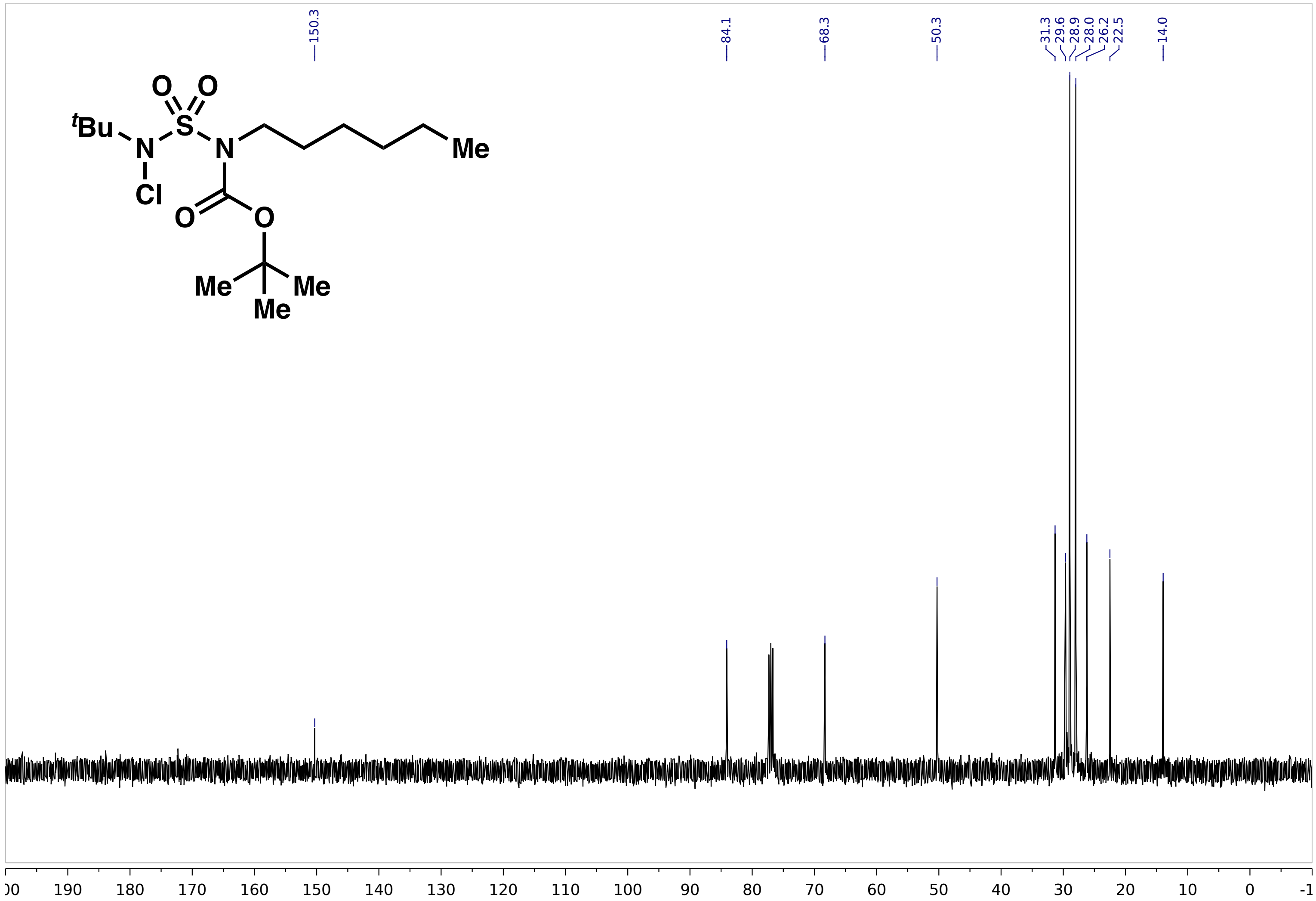

^{13}C NMR (101 MHz, $CDCl_3$) for *N*-*tert*-butoxycarbonyl-*N'*-*tert*-butyl-*N'*-chloro-*N*-hexylsulfamide (**1j**)

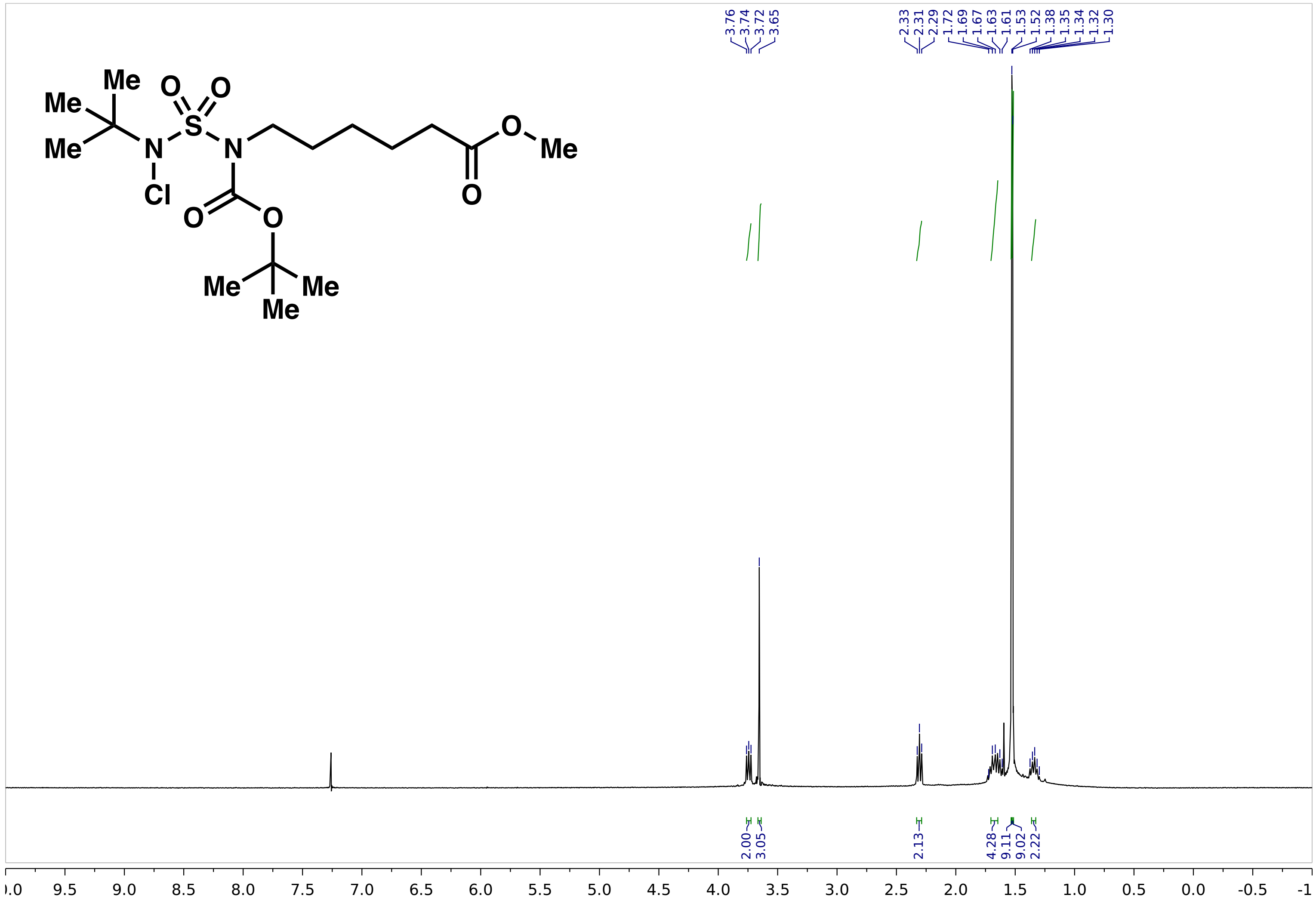

^{1}H NMR (400 MHz, $CDCl_3$) for *N*-*tert*-butyoxycarbonyl-*N'*-*tert*-butyl-*N'*-chloro-*N*-(methyl hexano-6-yl)sulfamide (**1k**)

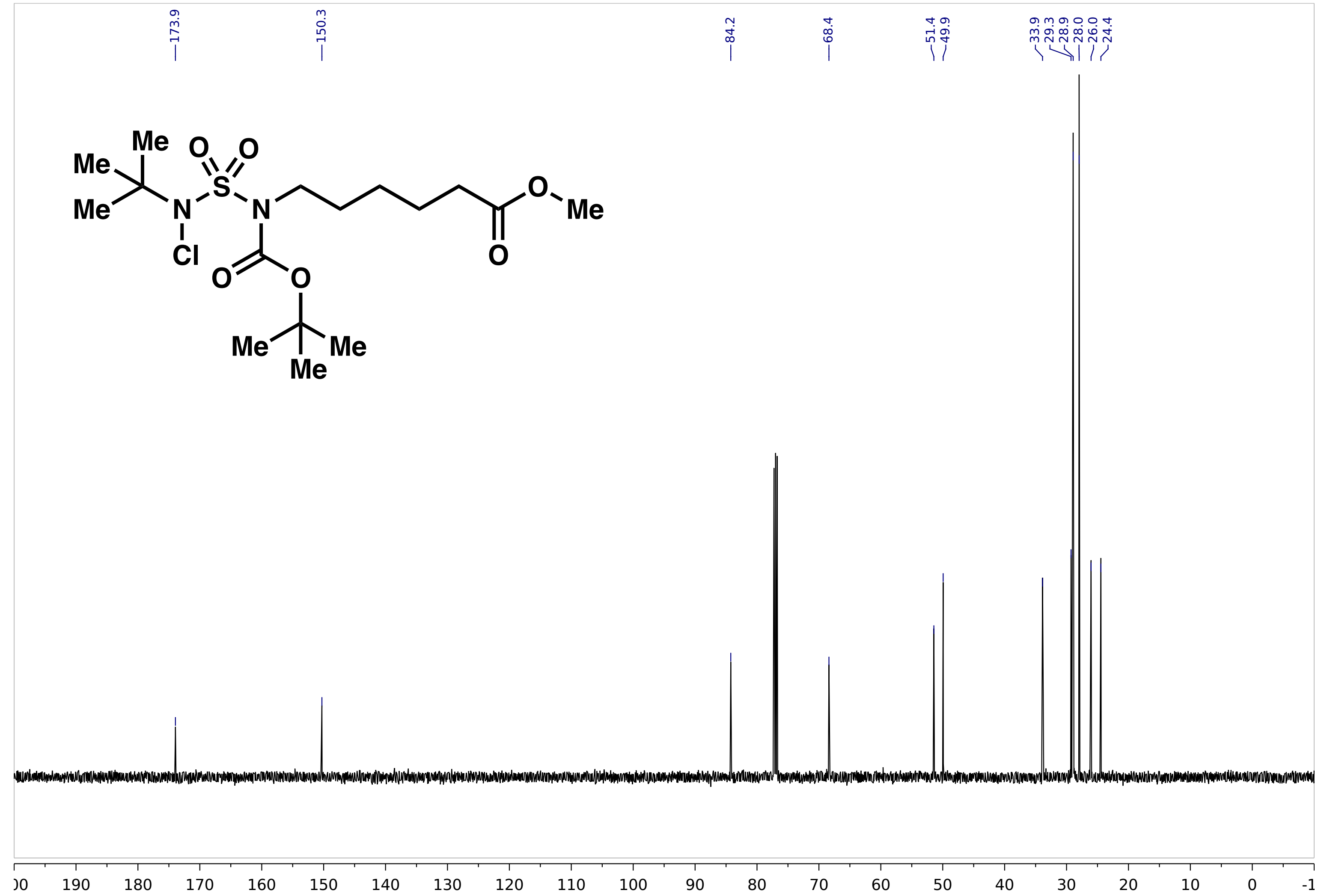

^{13}C NMR (126 MHz, $CDCl_3$) for *N*-*tert*-butyoxycarbonyl-*N'*-*tert*-butyl-*N'*-chloro-*N*-(methyl hexano-6-yl)sulfamide (**1k**)

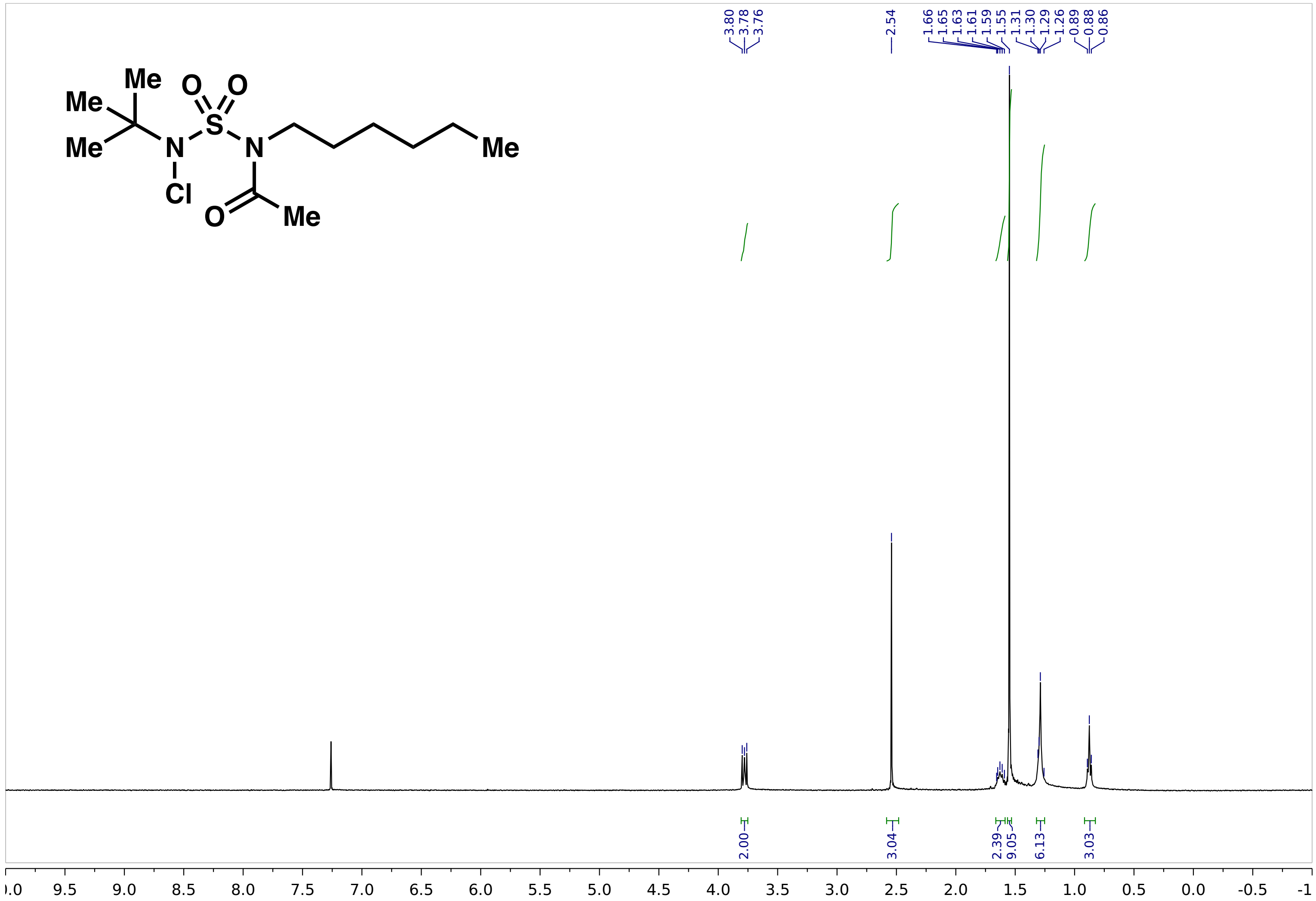

^{1}H NMR (400 MHz, $CDCl_3$) for *N*-acetyl-*N*'-*tert*-butyl-*N*'-chloro-*N*-hexylsulfamide (**1l**)

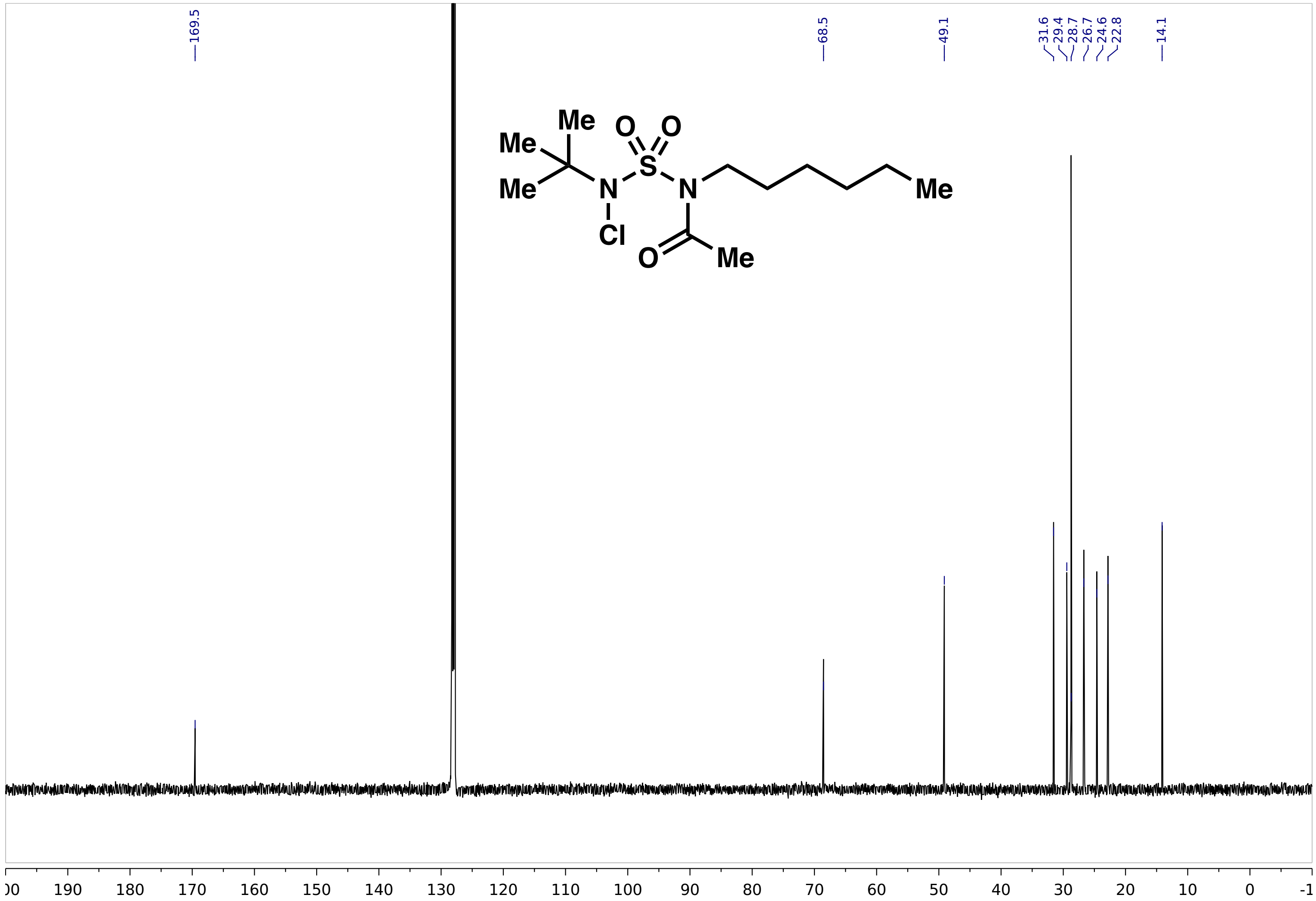

^{13}C NMR (126 MHz, C_6D_6) for *N*-acetyl-*N*'-*tert*-butyl-*N*'-chloro-*N*-hexylsulfamide (**1l**)

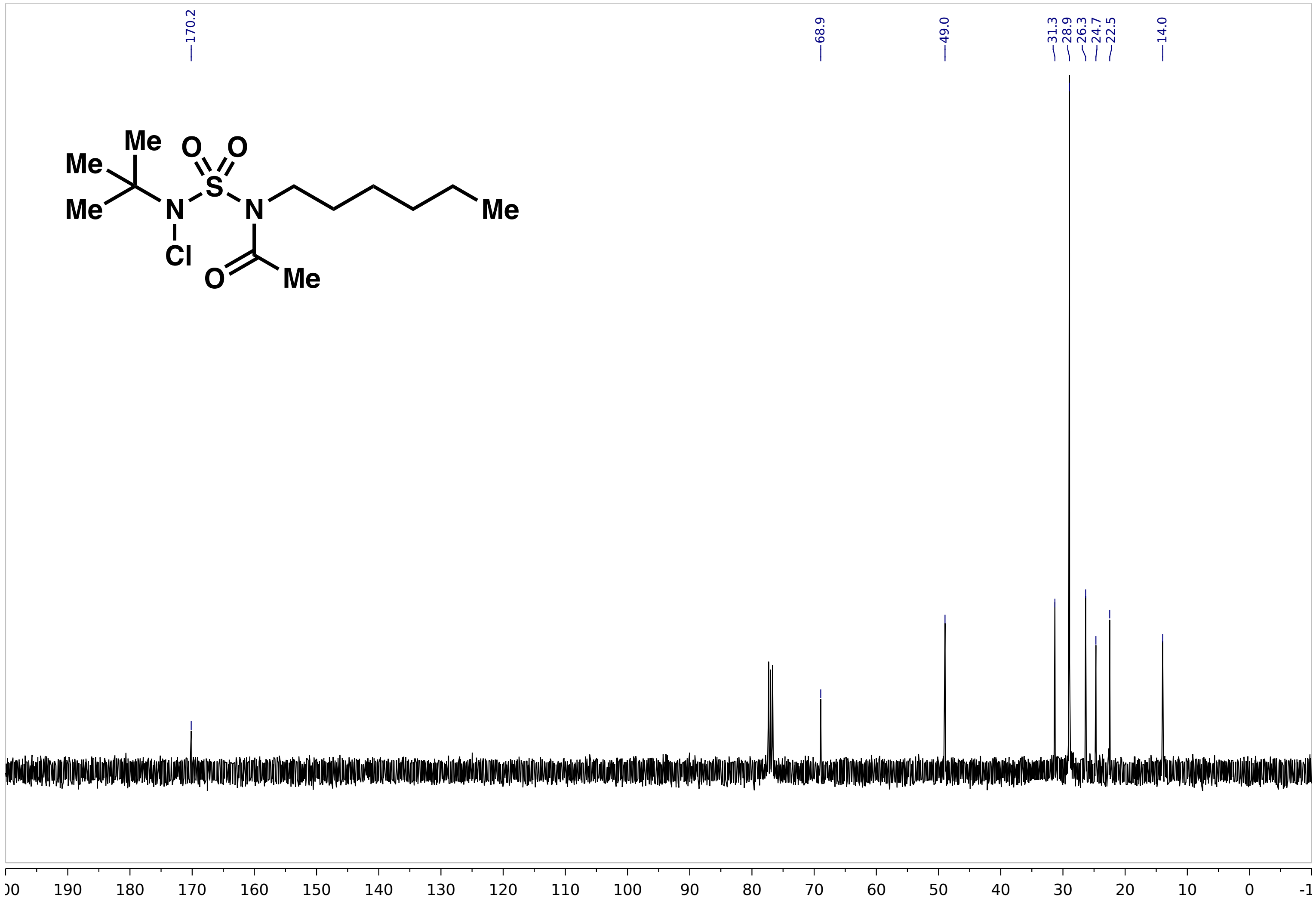

^{13}C NMR (101 MHz, $CDCl_3$) for *N*-acetyl-*N*'-*tert*-butyl-*N*'-chloro-*N*-hexylsulfamide (**1l**)

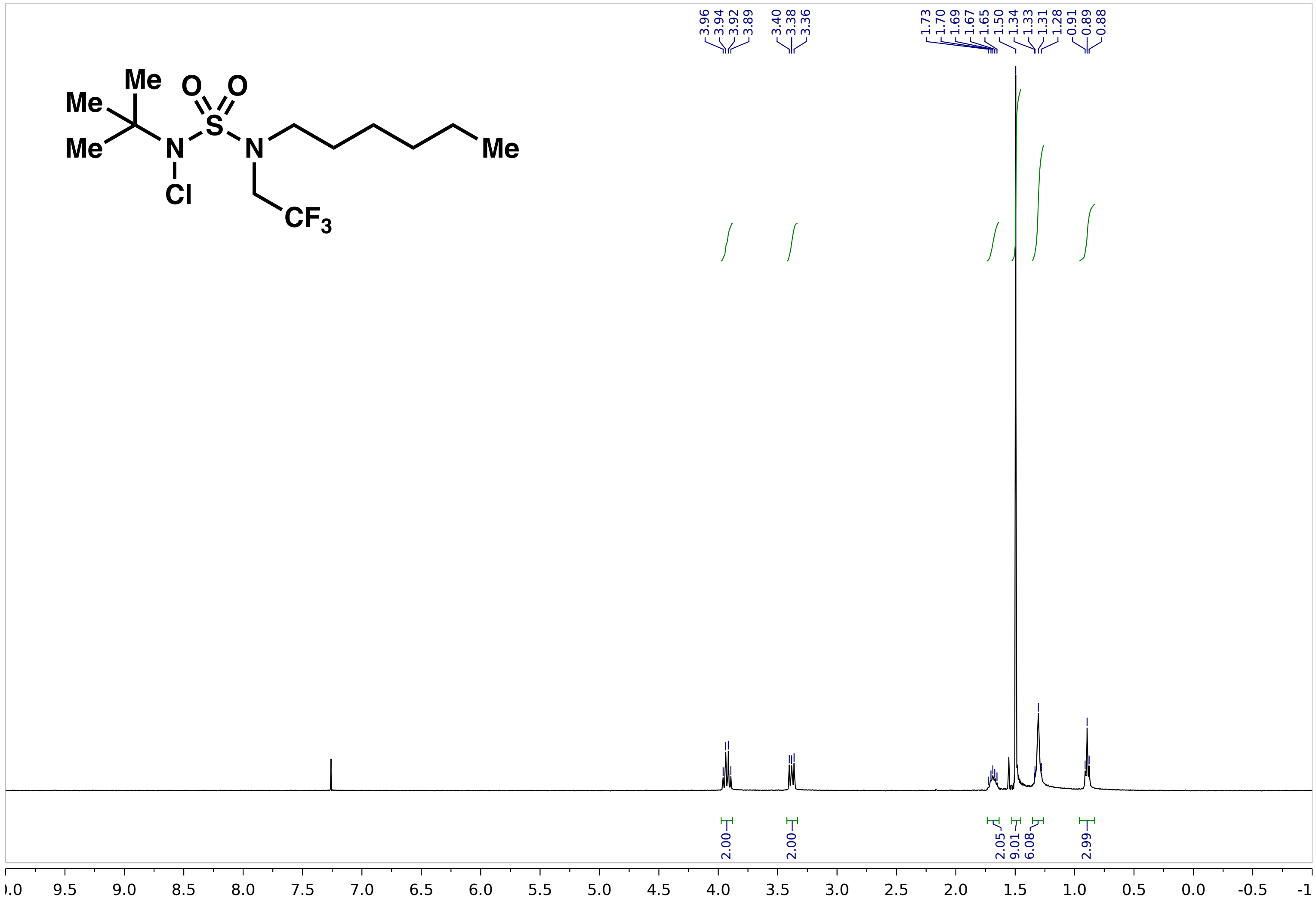

^{1}H NMR (400 MHz, $CDCl_3$) for *N*-*tert*-butyl-*N*-chloro-*N'*-hexyl-*N'*-(2,2,2-trifluoroethyl)sulfamide (**1m**)

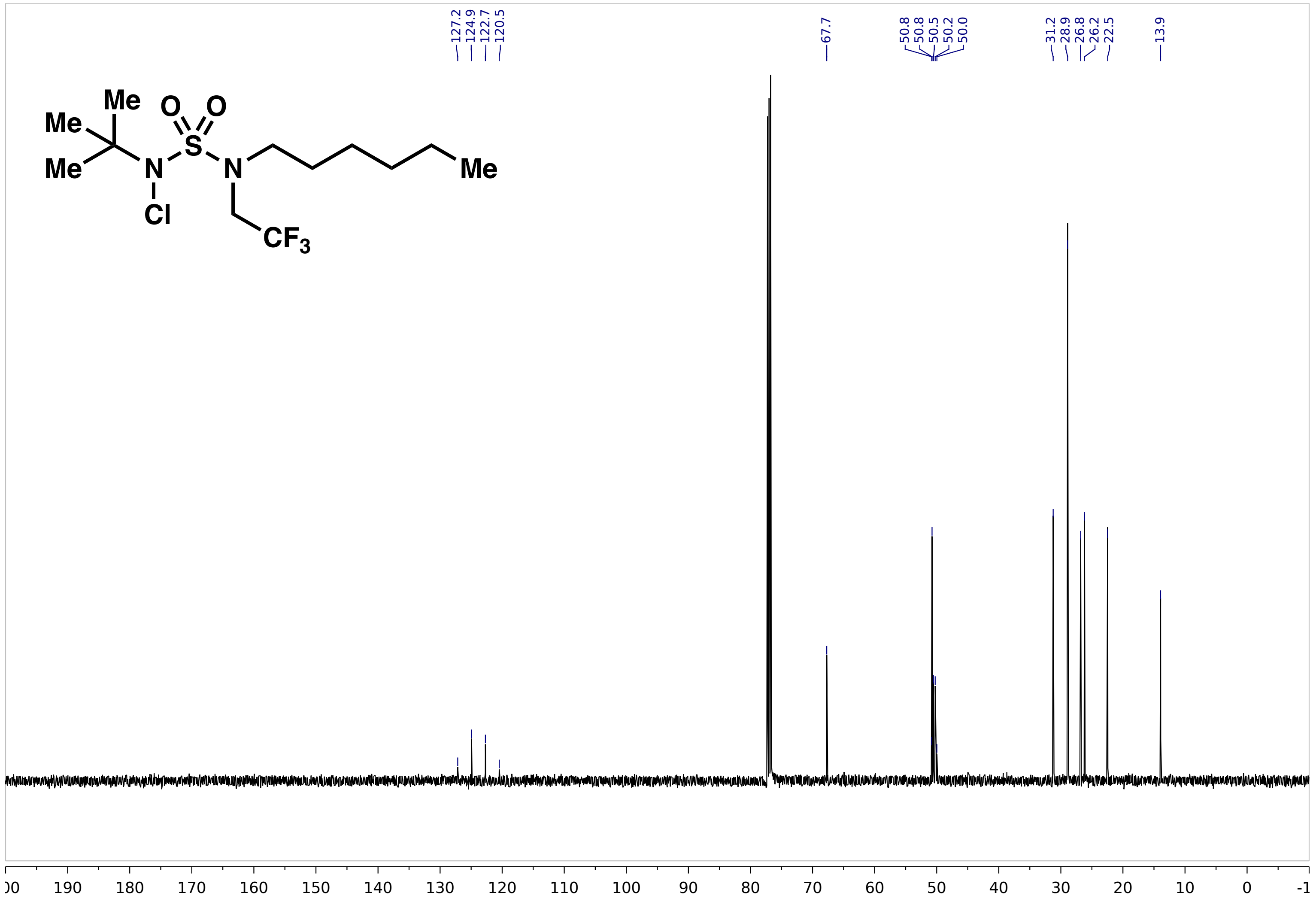

^{13}C NMR (126 MHz, $CDCl_3$) for *N*-*tert*-butyl-*N*-chloro-*N'*-hexyl-*N*'-(2,2,2-trifluoroethyl)sulfamide (**1m**)

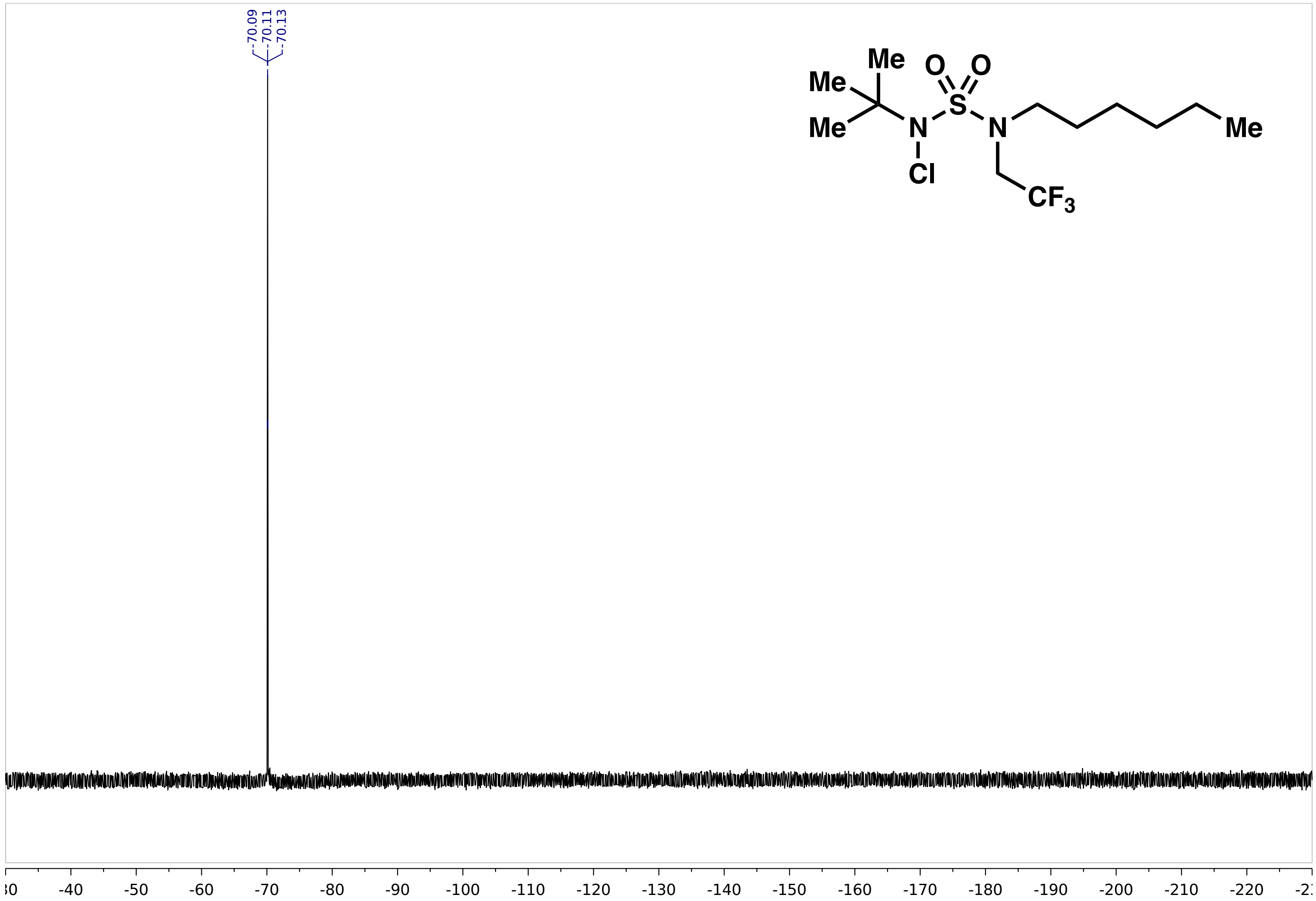

^{19}F NMR (376 MHz, $CDCl_3$) for *N*-*tert*-butyl-*N*-chloro-*N'*-hexyl-*N*'-(2,2,2-trifluoroethyl)sulfamide (**1m**)

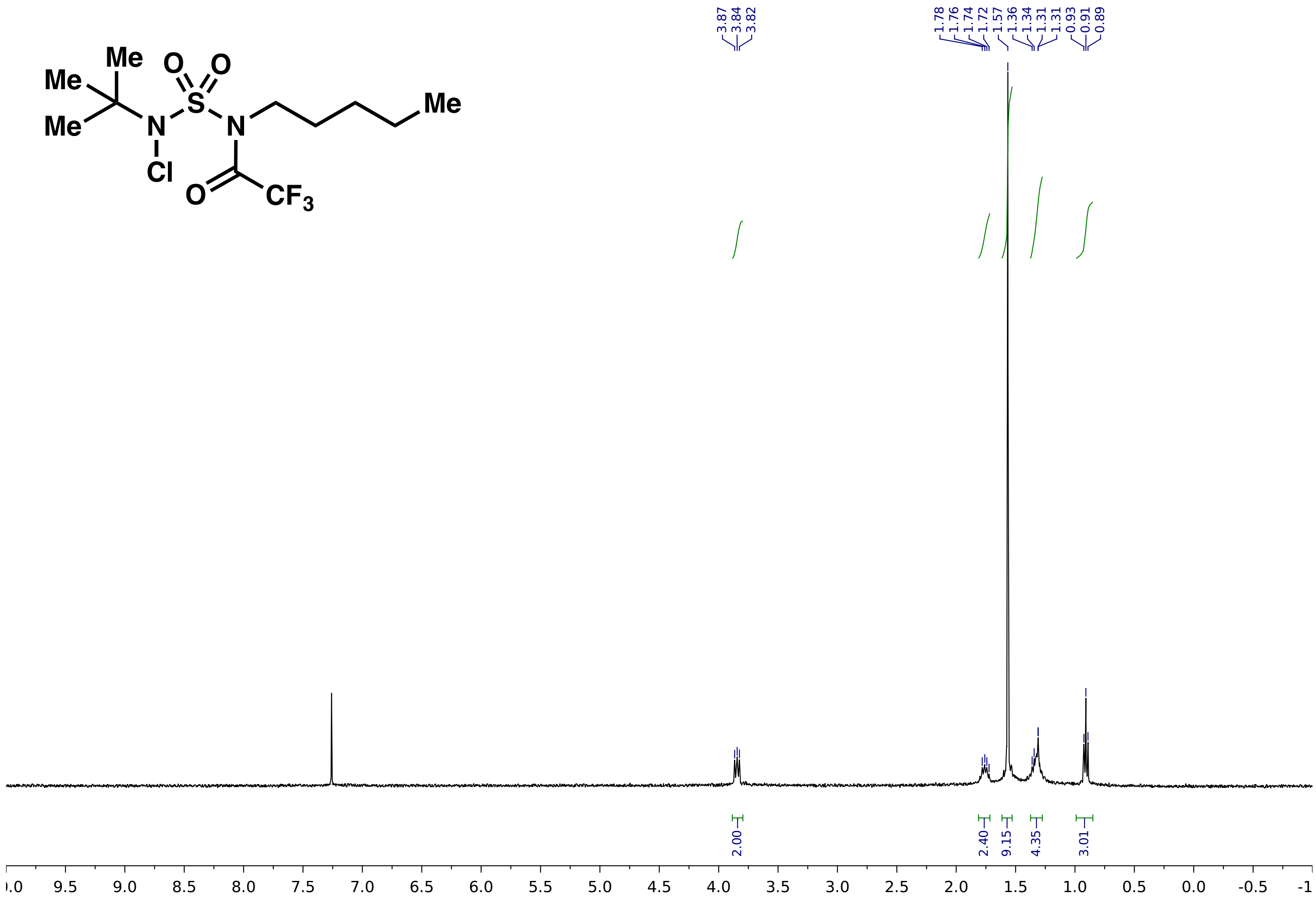

^{1}H NMR (400 MHz, $CDCl_3$) for *N'*-*tert*-butyl-*N'*-chloro-*N*-trifluoroacetyl-*N*-pentylsulfamide (**1n**)

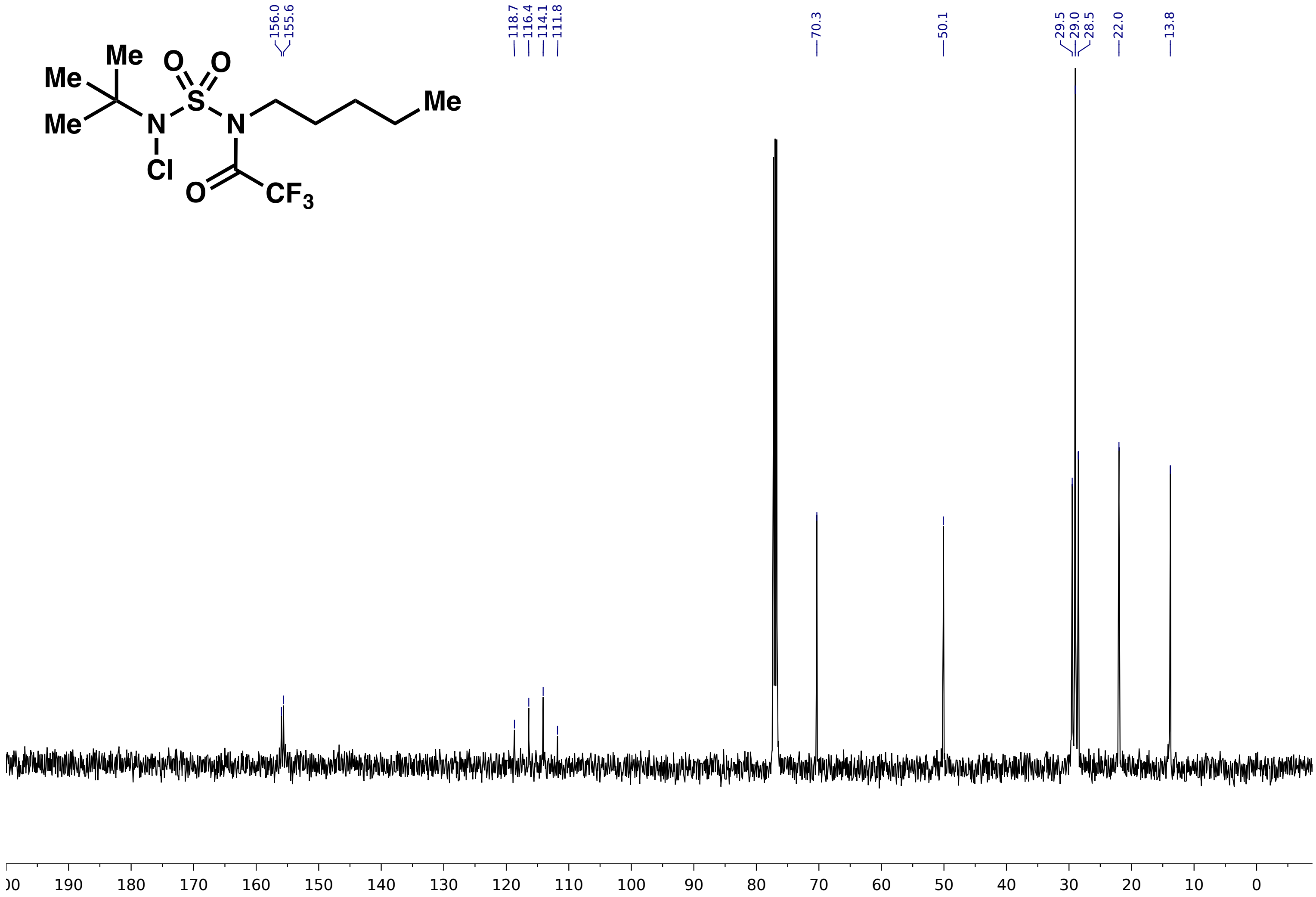

^{13}C NMR (400 MHz, $CDCl_3$) for *N'*-*tert*-butyl-*N'*-chloro-*N*-trifluoroacetyl-*N*-pentylsulfamide (**1n**)

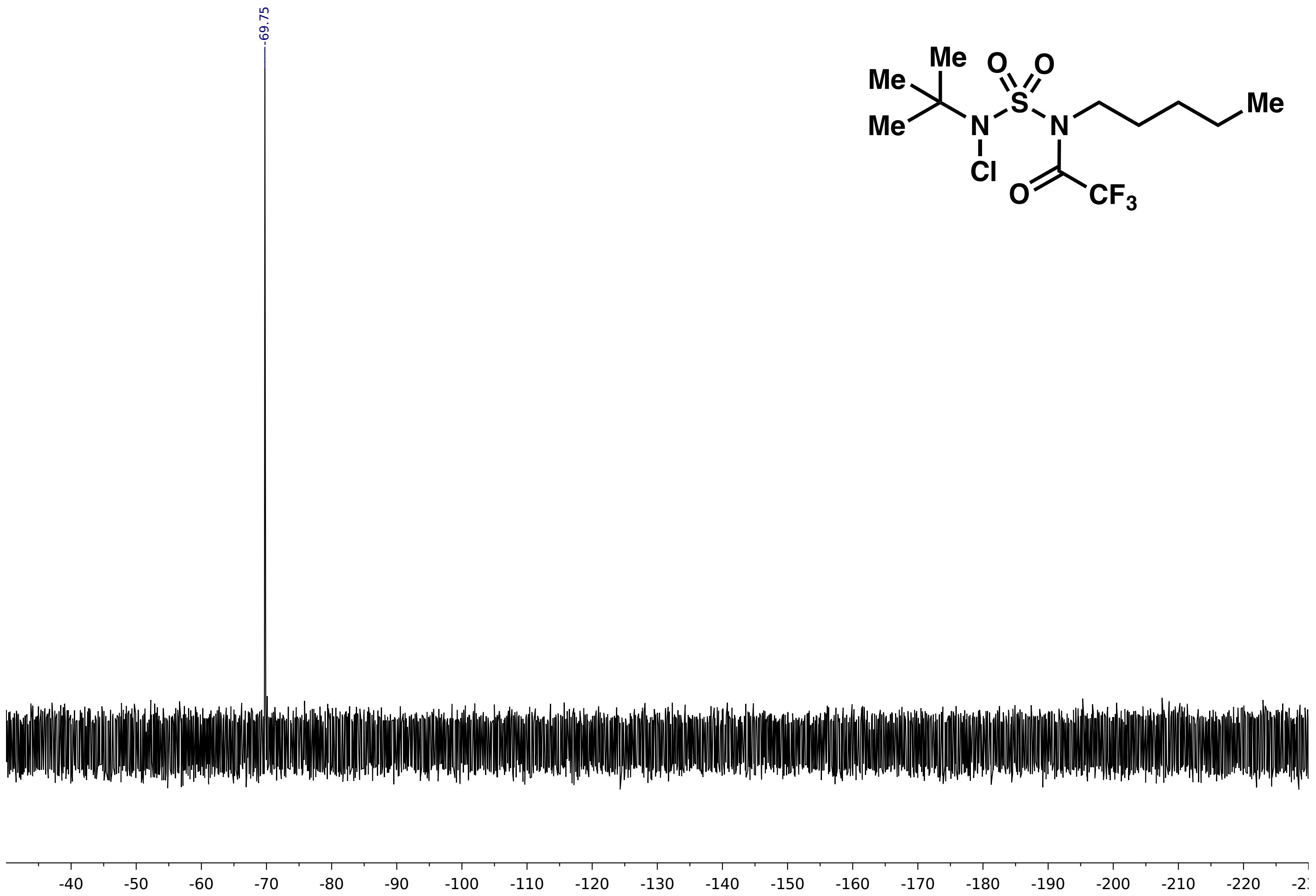

^{19}F NMR (376 MHz, $CDCl_3$) for *N'*-*tert*-butyl-*N'*-chloro-*N*-trifluoroacetyl-*N*-pentylsulfamide (**1n**)

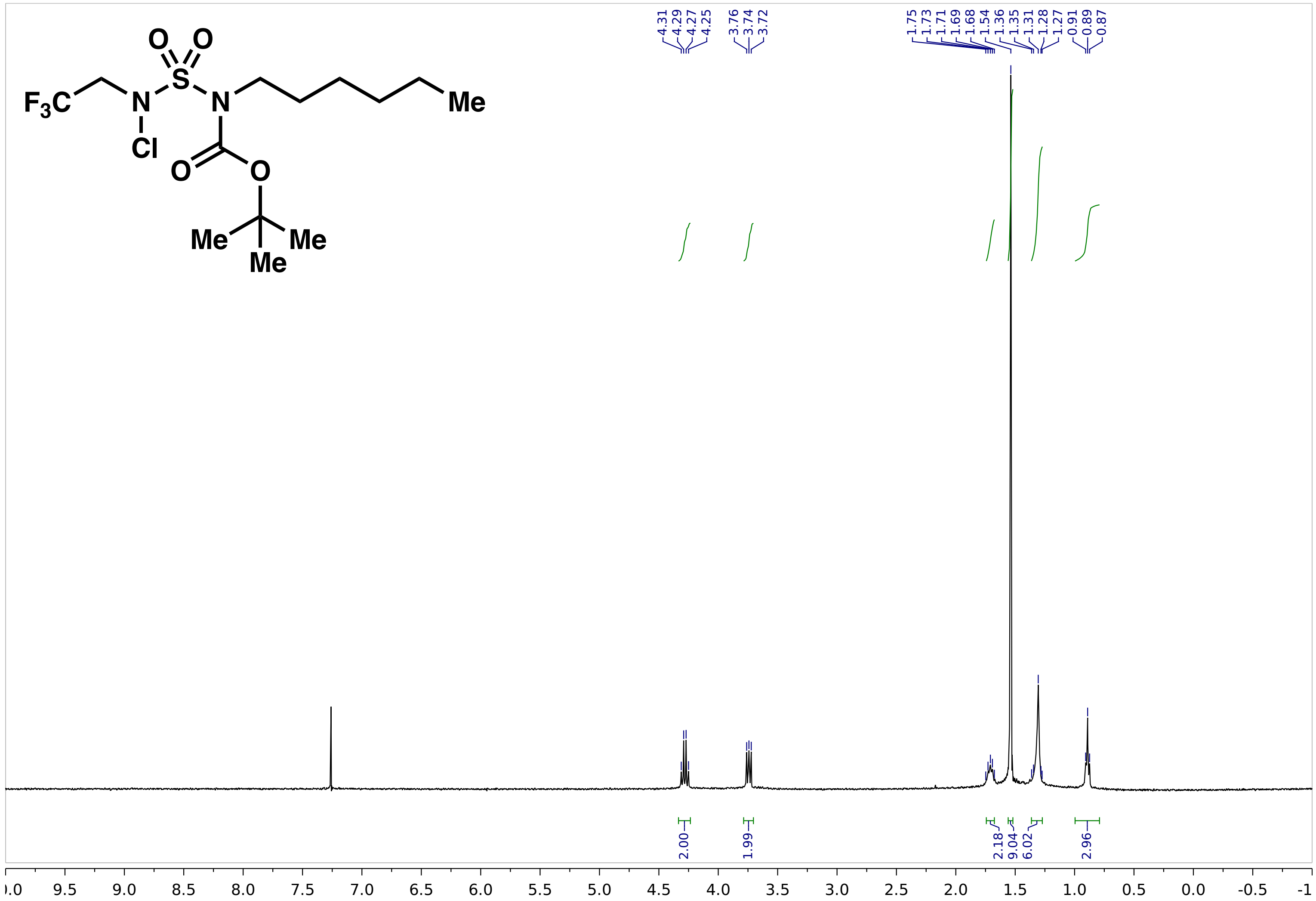

^{1}H NMR (400 MHz, $CDCl_3$) for *N*-*tert*-butoxycarbonyl-*N*-hexyl-*N'*-chloro-*N'*-(2,2,2-trifluoroethyl)sulfamide (**1o**)

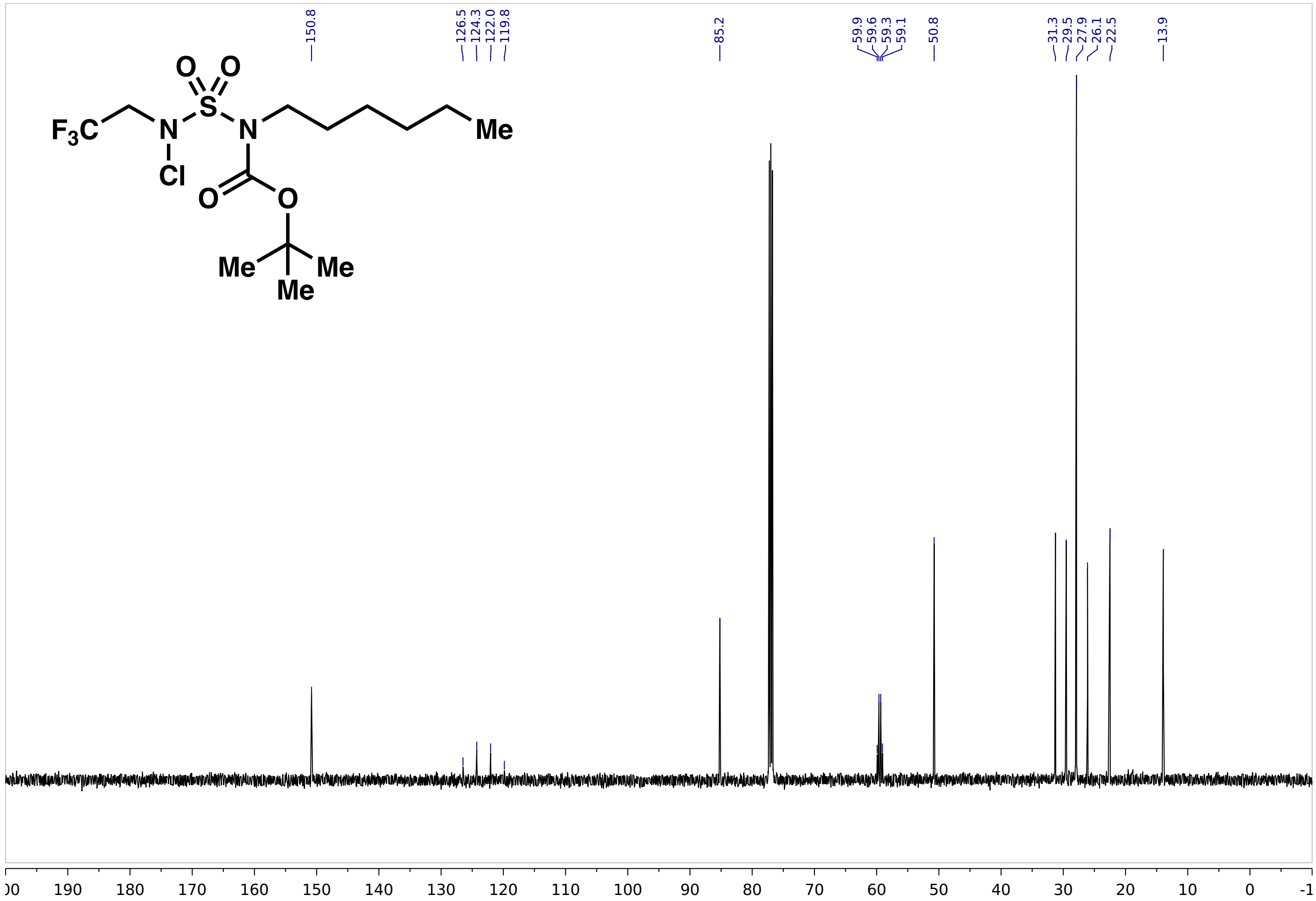

^{13}C NMR (126 MHz, $CDCl_3$) for *N*-*tert*-butoxycarbonyl-*N*-hexyl-*N'*-chloro-*N'*-(2,2,2-trifluoroethyl)sulfamide (**1o**)

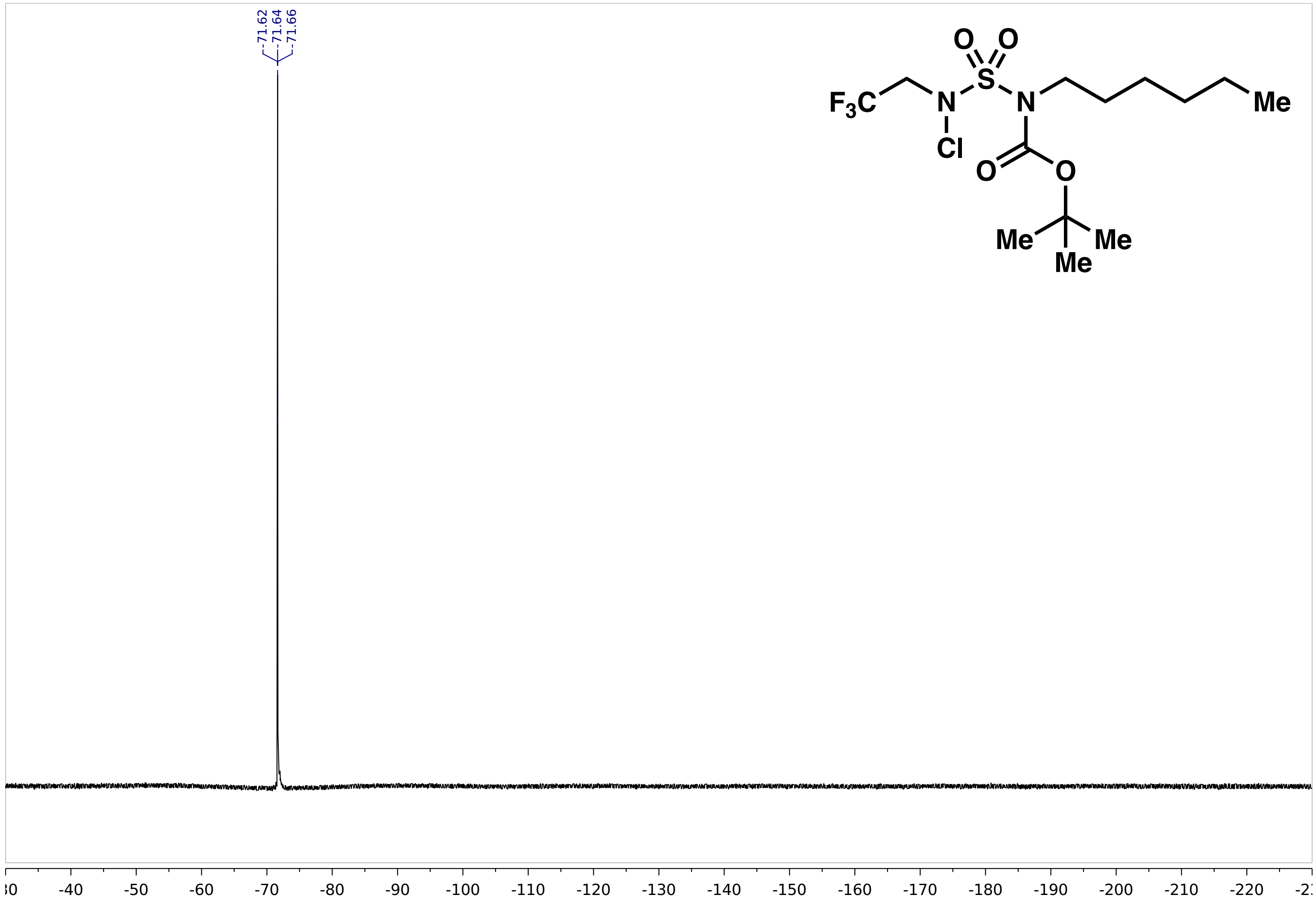

^{19}F NMR (126 MHz, $CDCl_3$) for *N*-*tert*-butoxycarbonyl-*N*-hexyl-*N'*-chloro-*N'*-(2,2,2-trifluoroethyl)sulfamide (**1o**)

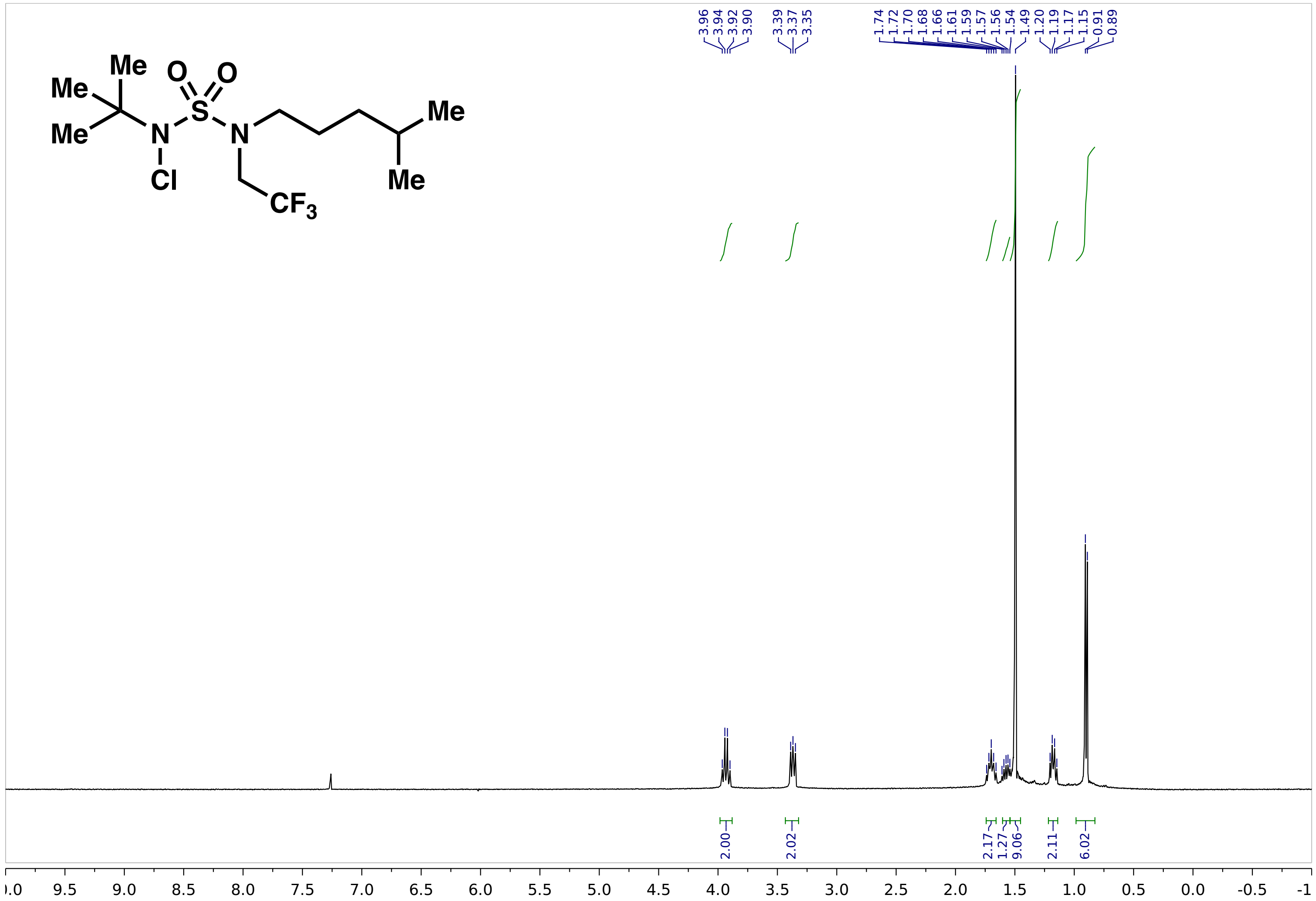

^{1}H NMR (400 MHz, $CDCl_3$) for *N*-*tert*-butyl-*N*-chloro-*N*'-4-methylpentyl-*N*'-(2,2,2-trifluoroethyl)sulfamide (**1p**)

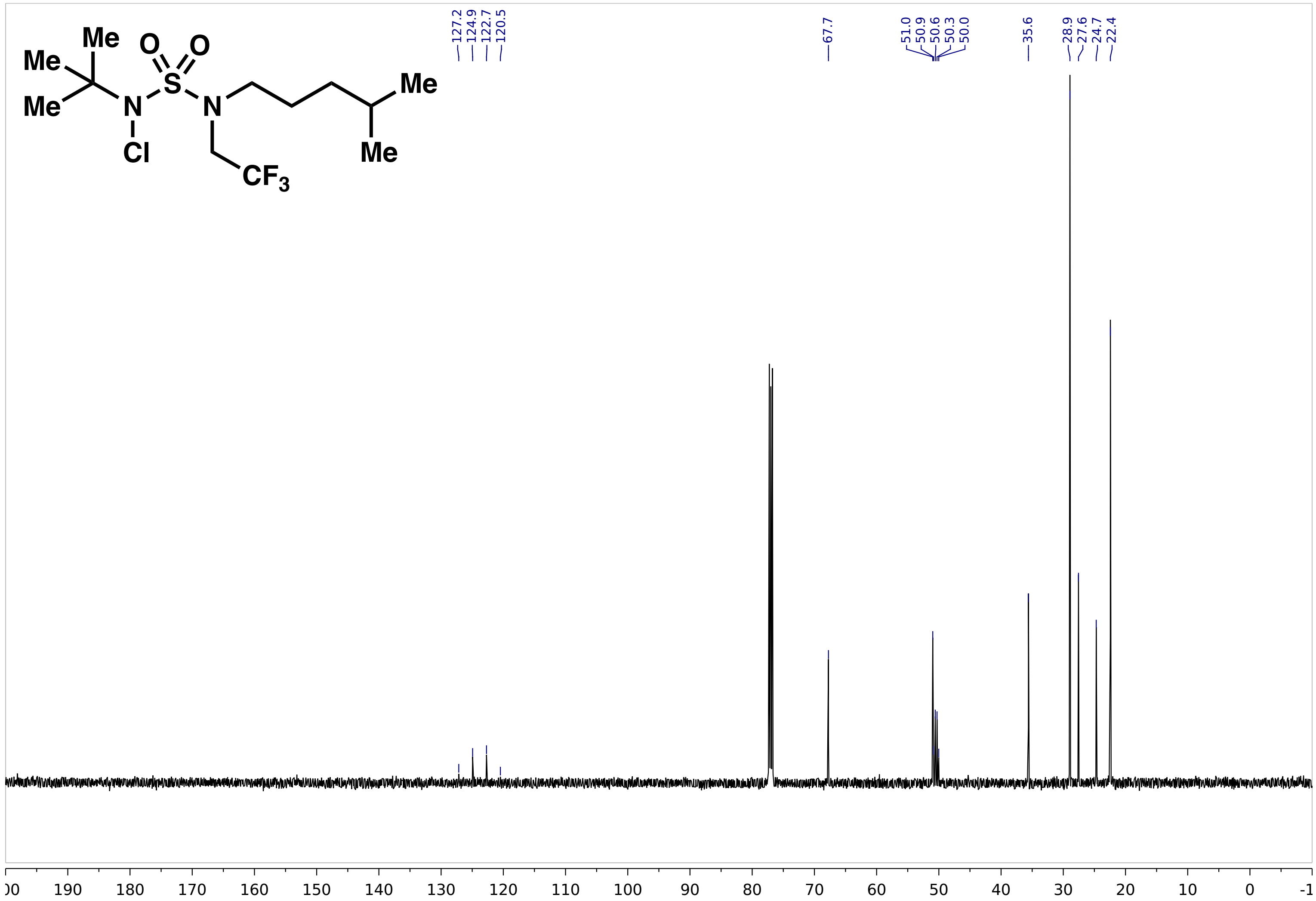

^{13}C NMR (126 MHz, $CDCl_3$) for *N*-*tert*-butyl-*N*-chloro-*N*'-4-methylpentyl-*N*'-(2,2,2-trifluoroethyl)sulfamide (**1p**)

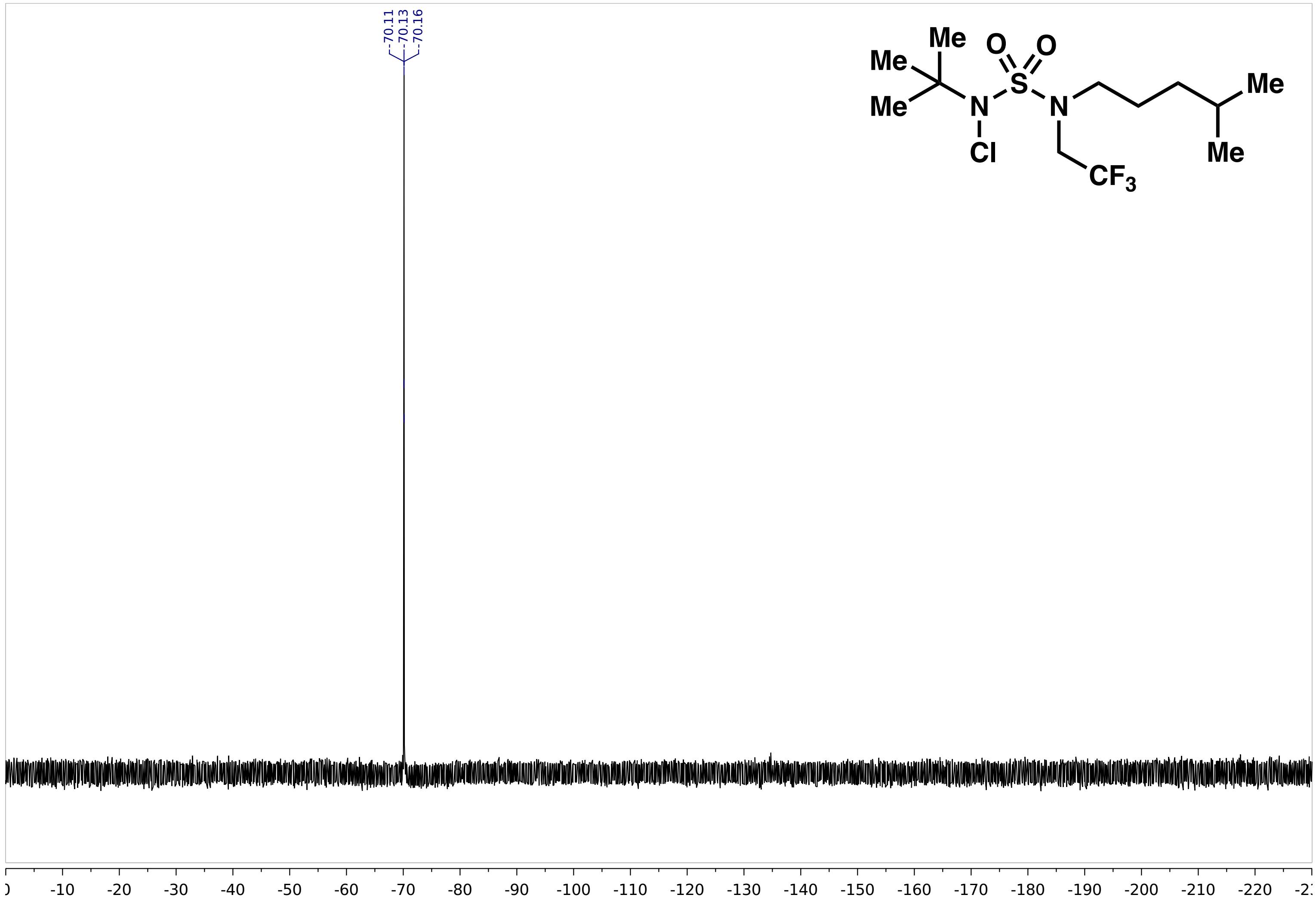

^{19}F NMR (376 MHz, $CDCl_3$) for *N*-*tert*-butyl-*N*-chloro-*N*'-4-methylpentyl-*N*'-(2,2,2-trifluoroethyl)sulfamide (**1p**)

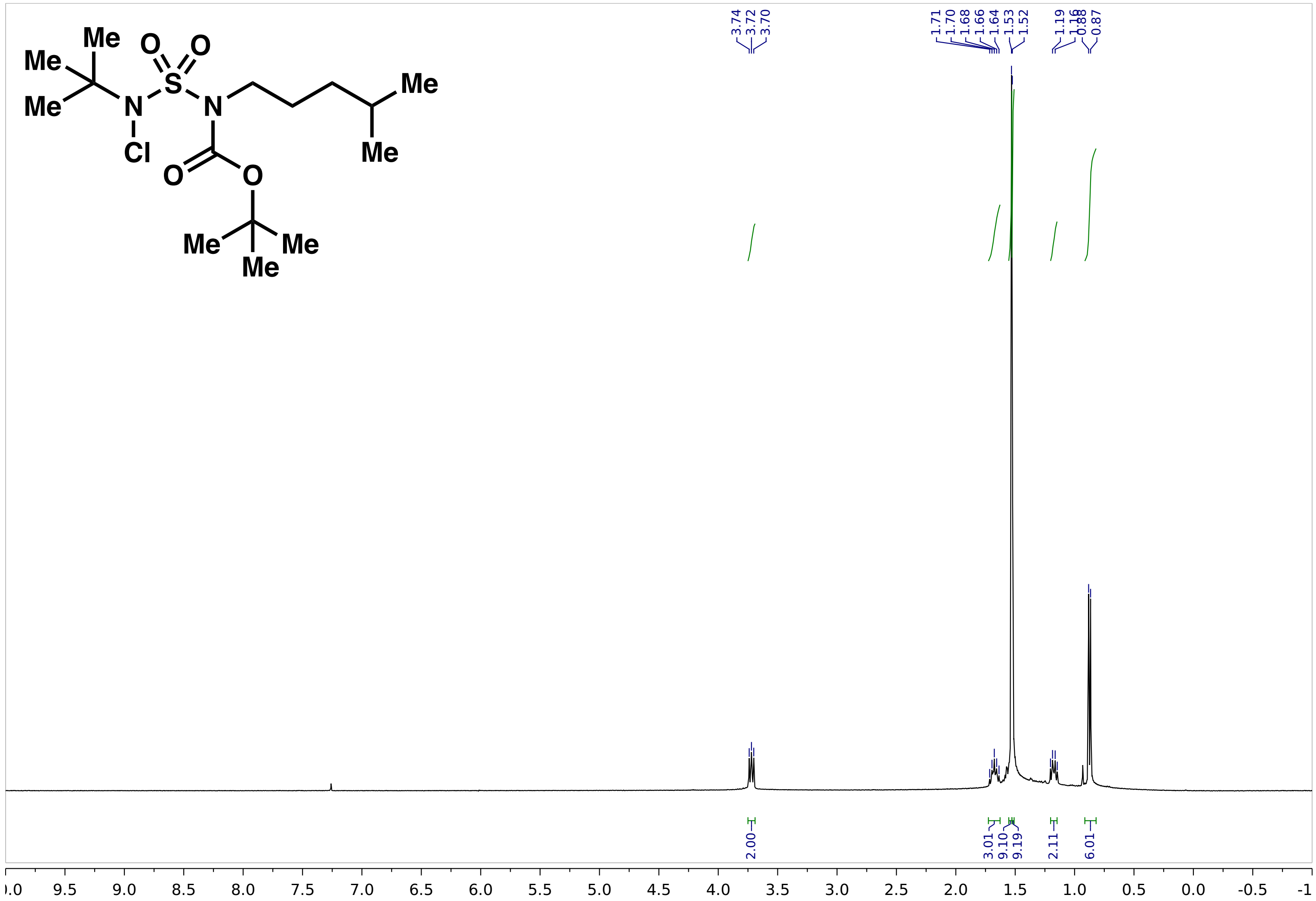

^{1}H NMR (400 MHz, $CDCl_3$) for *N*-*tert*-butoxycarbonyl-*N'*-*tert*-butyl-*N'*-chloro-*N*-4-methylpentylsulfamide (**1q**)

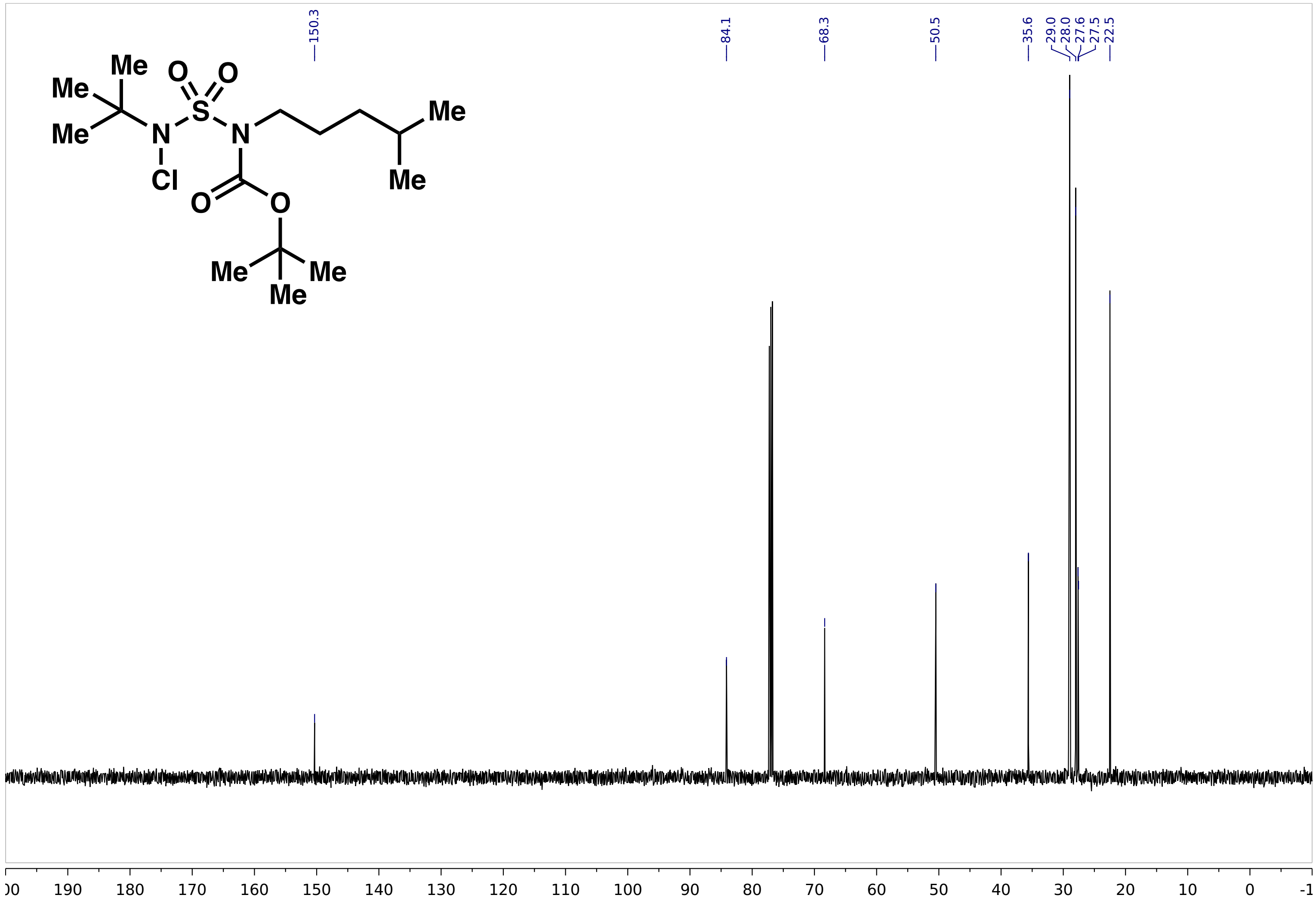

^{13}C NMR (126 MHz, $CDCl_3$) for *N*-*tert*-butoxycarbonyl-*N'*-*tert*-butyl-*N'*-chloro-*N*-4-methylpentylsulfamide (**1q**)

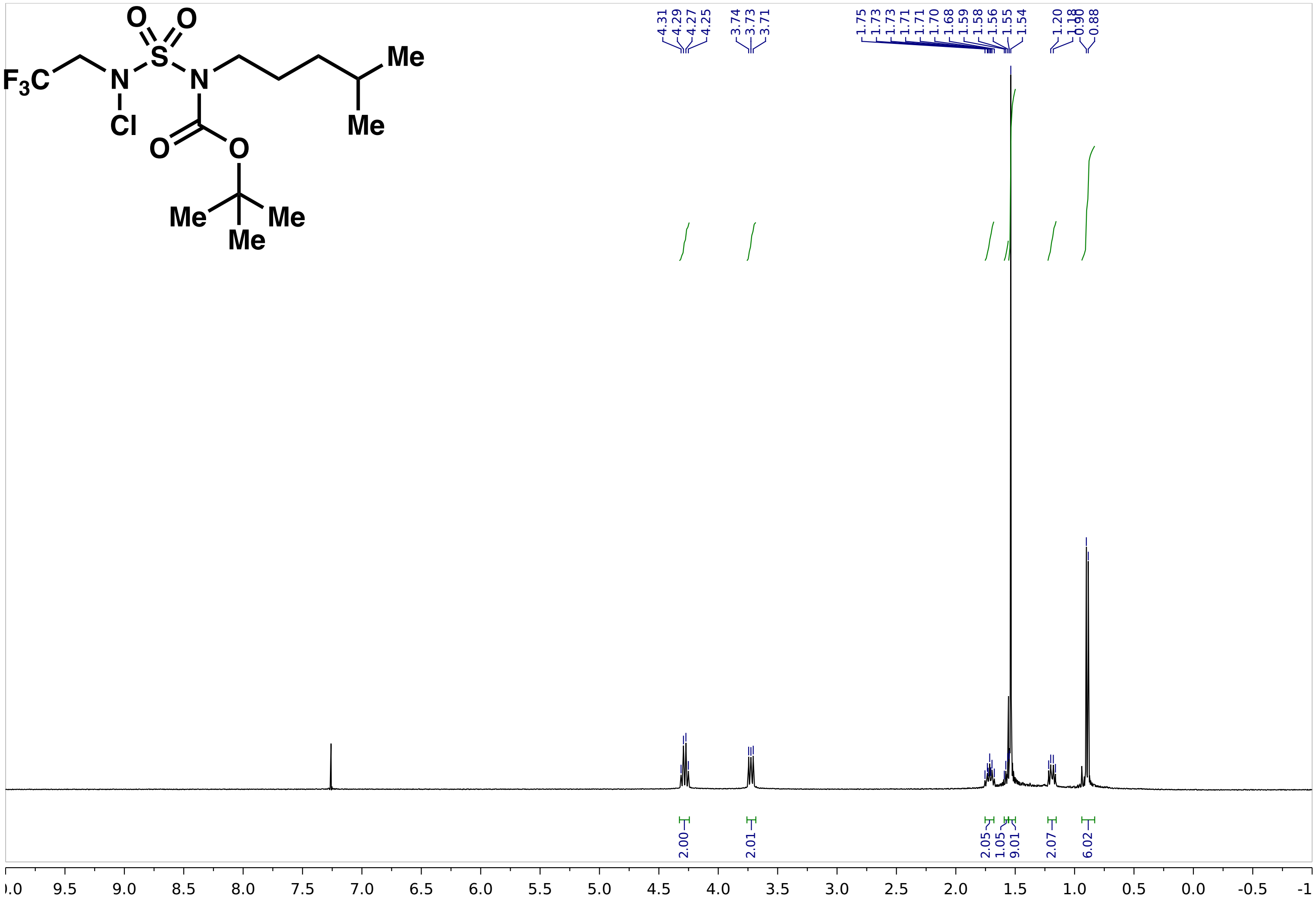

^{1}H NMR (400 MHz, $CDCl_3$) for *N*-*tert*-Butoxycarbonyl-*N*'-chloro-*N*-4-methylpentyl-*N'*-(2,2,2-trifluoroethyl)sulfamide (**1r**)

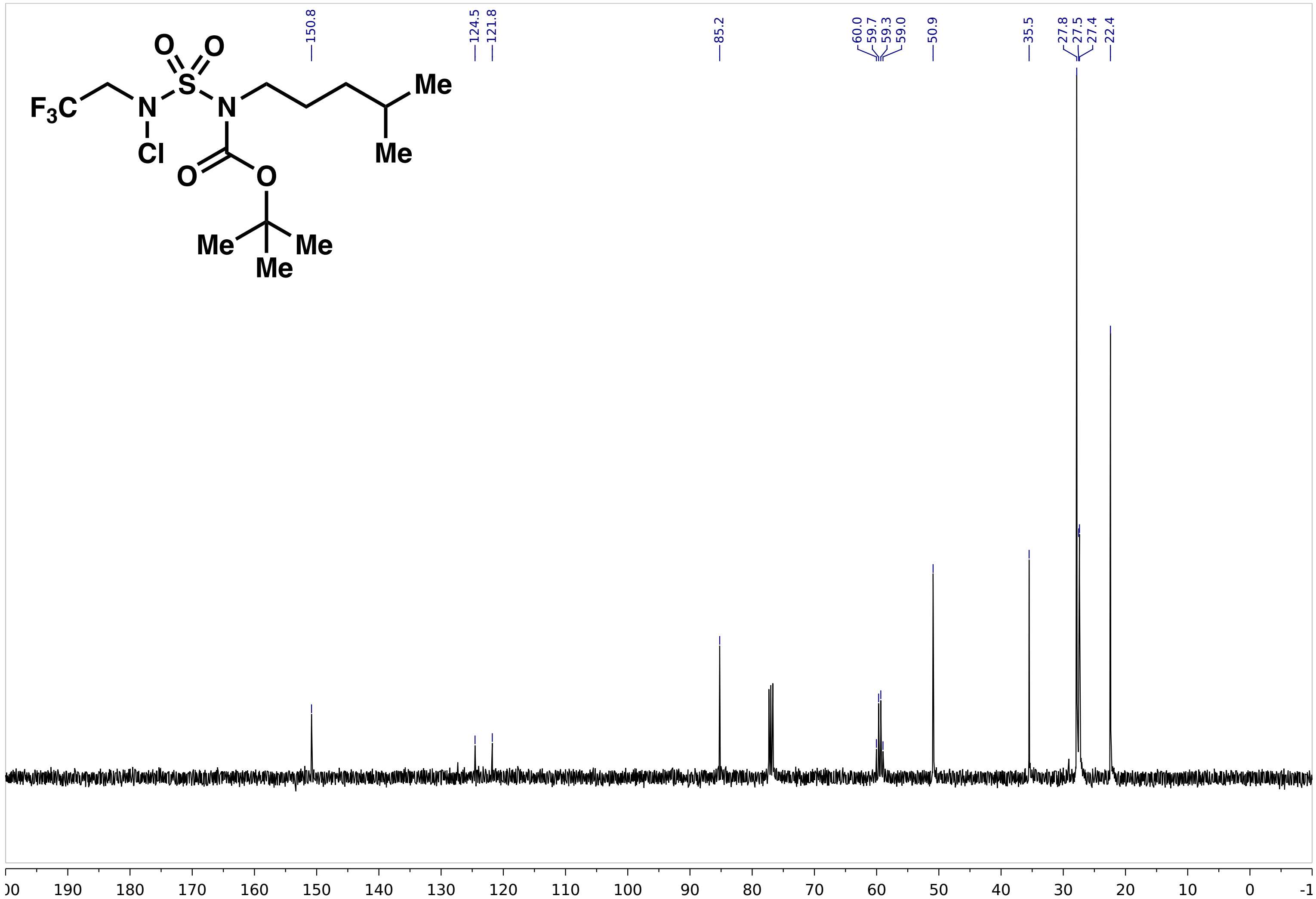

^{13}C NMR (101 MHz, $CDCl_3$) for *N*-*tert*-Butoxycarbonyl-*N*'-chloro-*N*-4-methylpentyl-*N'*-(2,2,2-trifluoroethyl)sulfamide (**1r**)

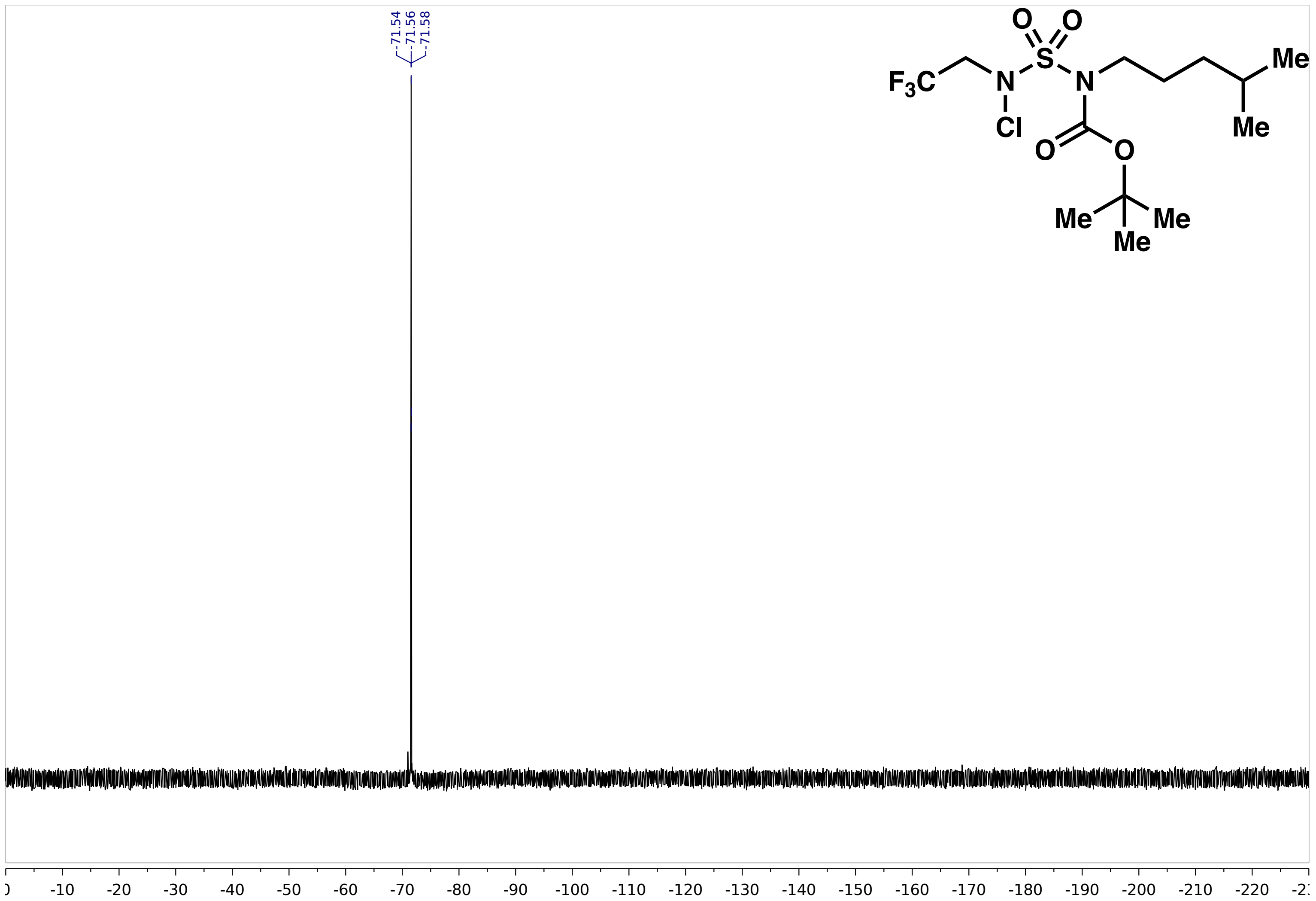

^{19}F NMR (376 MHz, $CDCl_3$) for *N*-*tert*-Butoxycarbonyl-*N*'-chloro-*N*-4-methylpentyl-*N*'-(2,2,2-trifluoroethyl)sulfamide (**1r**)

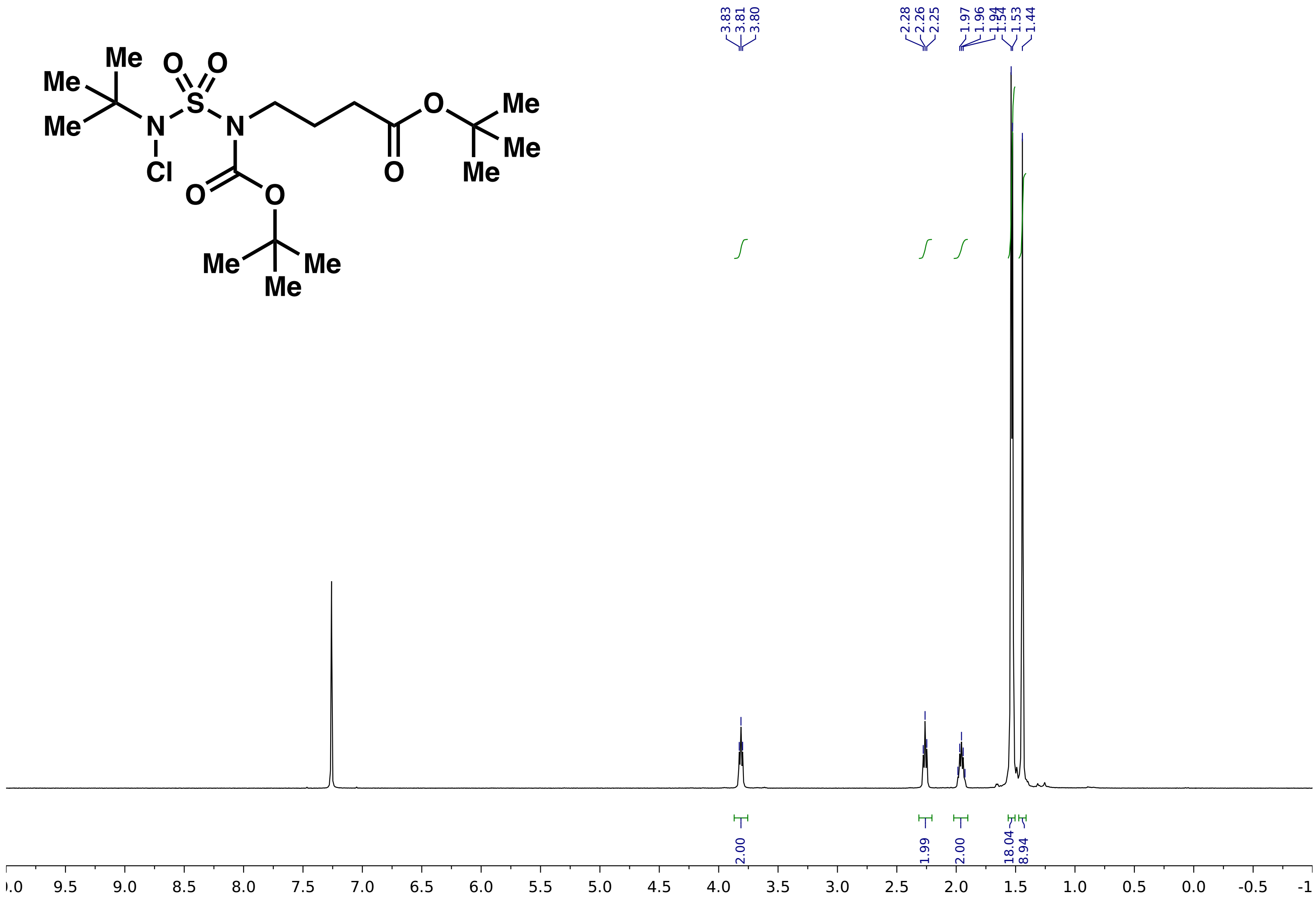

^{1}H NMR (400 MHz, $CDCl_3$) for *N*-*tert*-butoxycarbonyl-*N'*-tert-butyl-*N*-(*tert*-butyl butano-4-yl)-*N'*-chlorosulfamide (**S9t**)

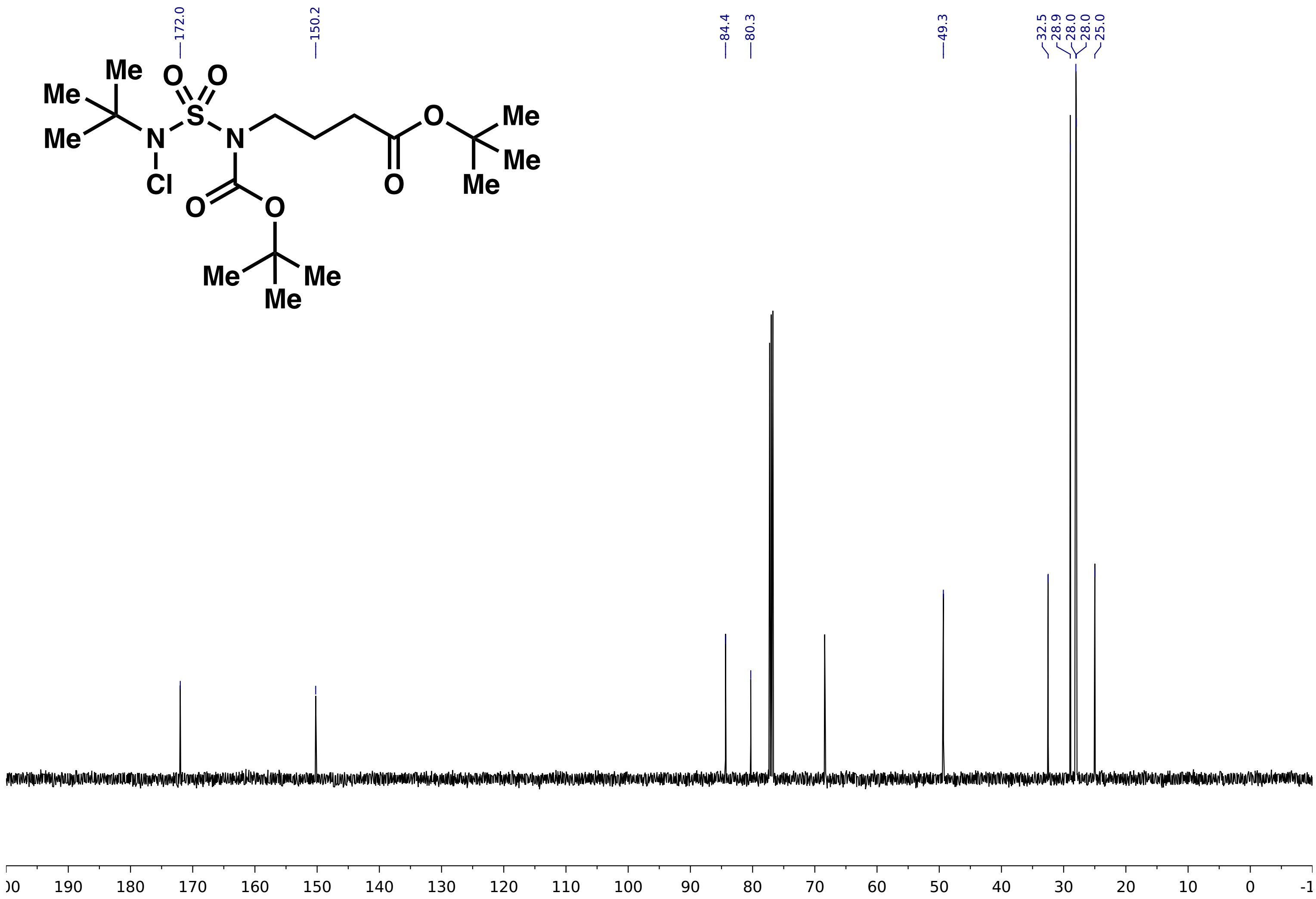

^{13}C NMR (126 MHz, $CDCl_3$) for *N*-*tert*-butoxycarbonyl-*N'*-tert-butyl-*N*-(*tert*-butyl butano-4-yl)-*N'*-chlorosulfamide (**S9t**)

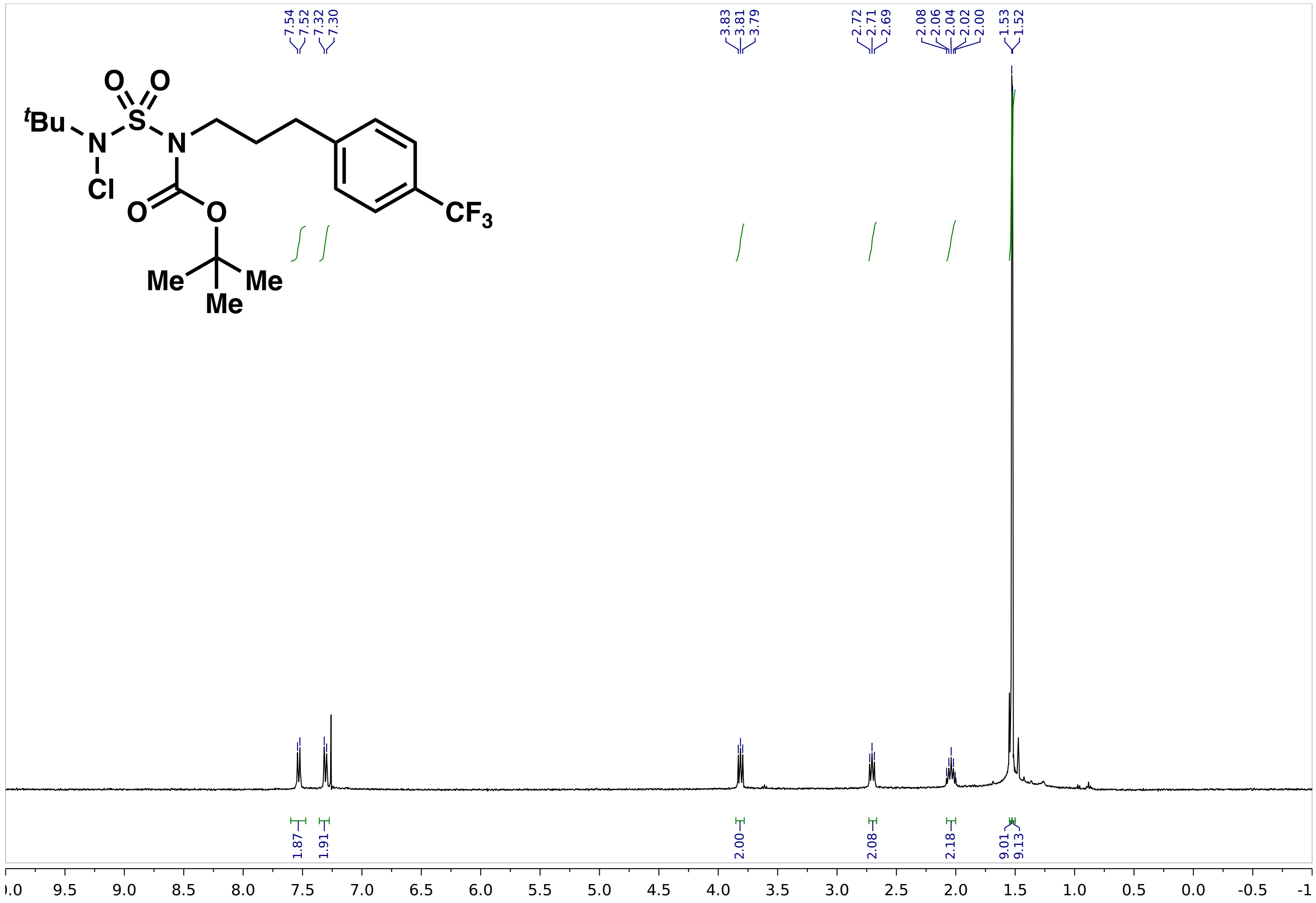

^{1}H NMR (400 MHz, $CDCl_3$) for *N*-*tert*-butoxycarbonyl-*N'*-*tert*-butyl-*N'*-chloro-*N*-(3-((4-trifluoromethyl)phenyl)prop-1-yl)sulfamide (**S9u**)

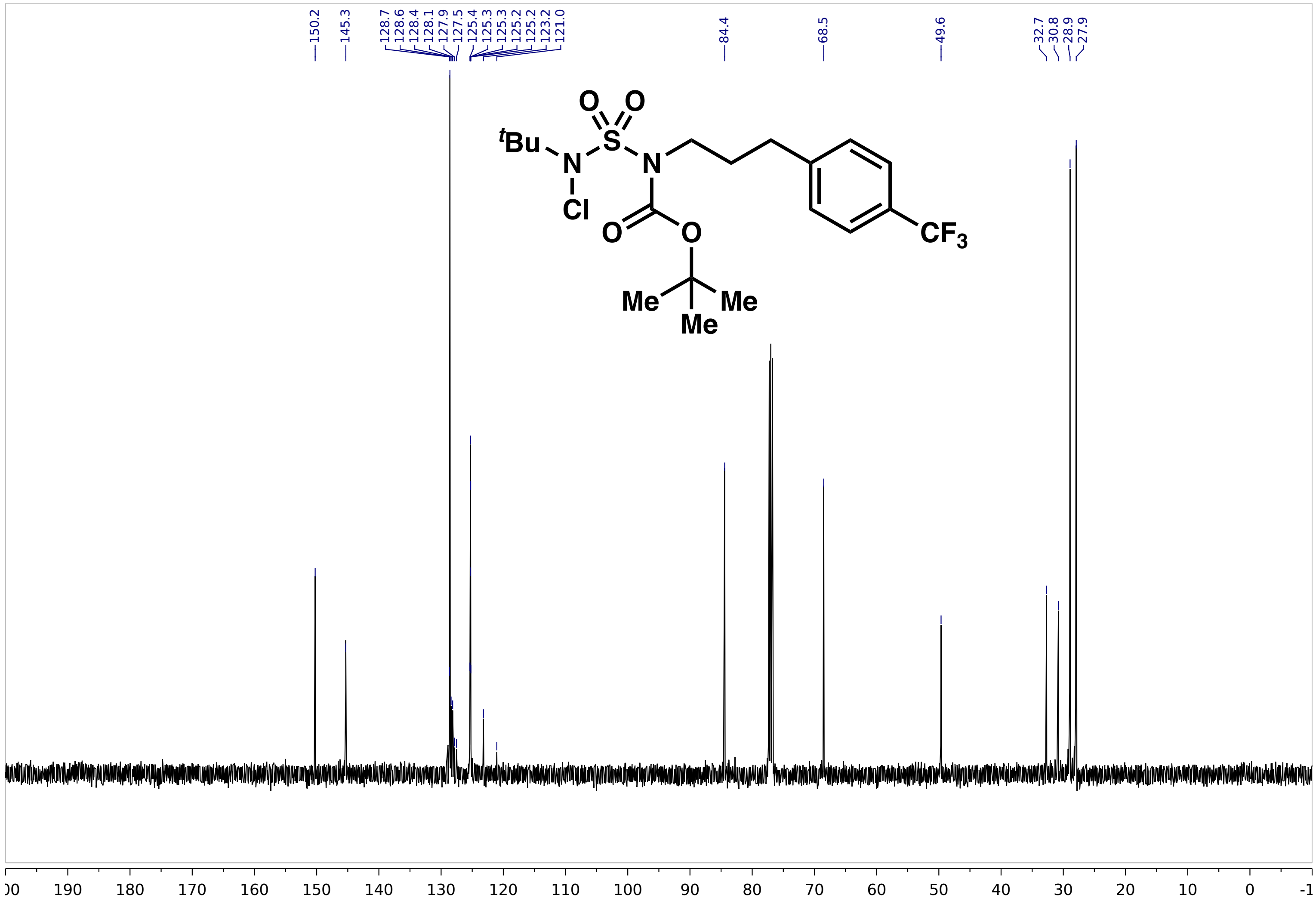

^{13}C NMR (126 MHz, $CDCl_3$) for *N*-*tert*-butoxycarbonyl-*N'*-*tert*-butyl-*N'*-chloro-*N*-(3-((4-trifluoromethyl)phenyl)prop-1-yl)sulfamide (**S9u**)

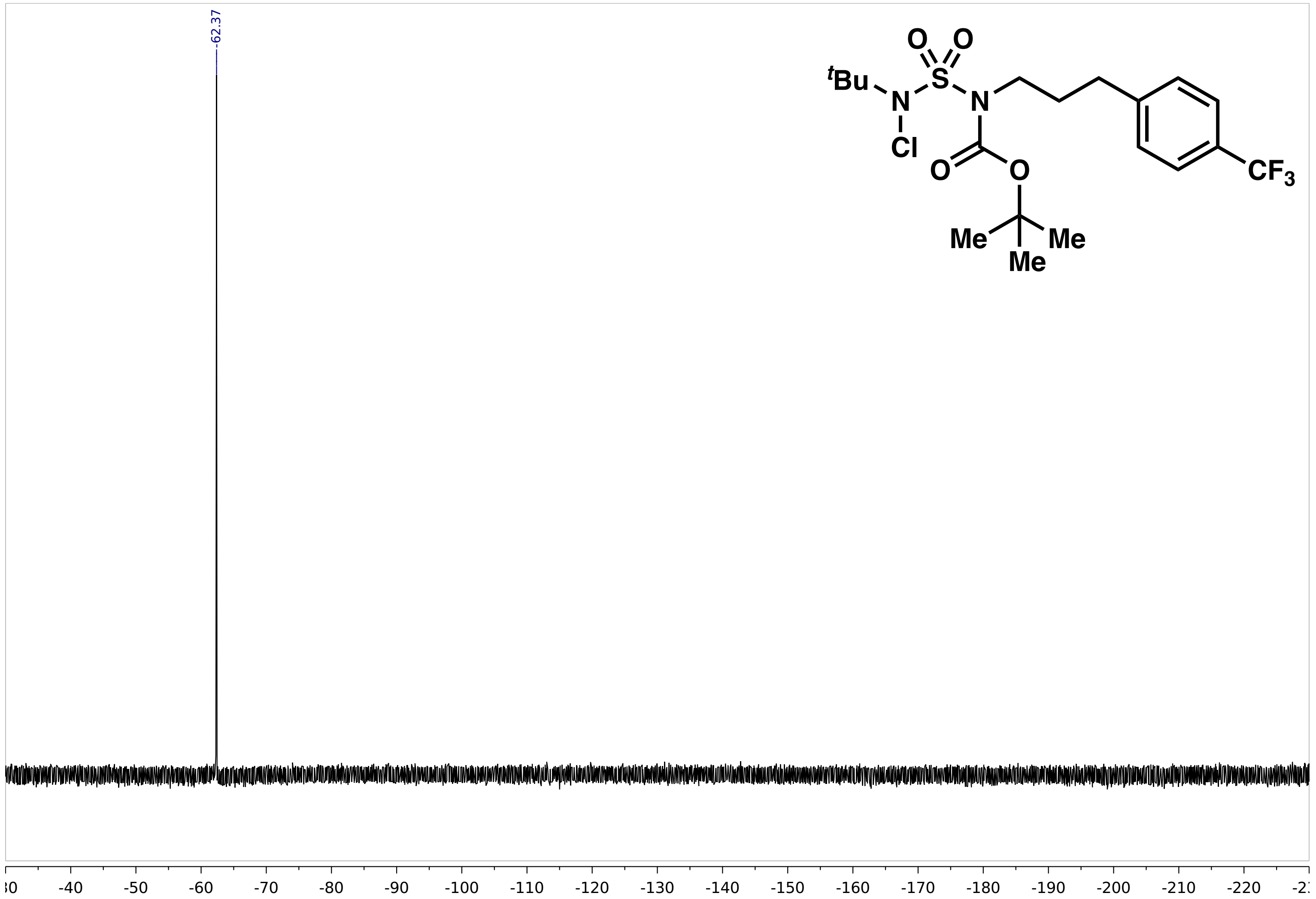

^{19}F NMR (376 MHz, $CDCl_3$) for *N*-*tert*-butoxycarbonyl-*N'*-*tert*-butyl-*N'*-chloro-*N*-(3-((4-trifluoromethyl)phenyl)prop-1-yl)sulfamide (**S9u**)

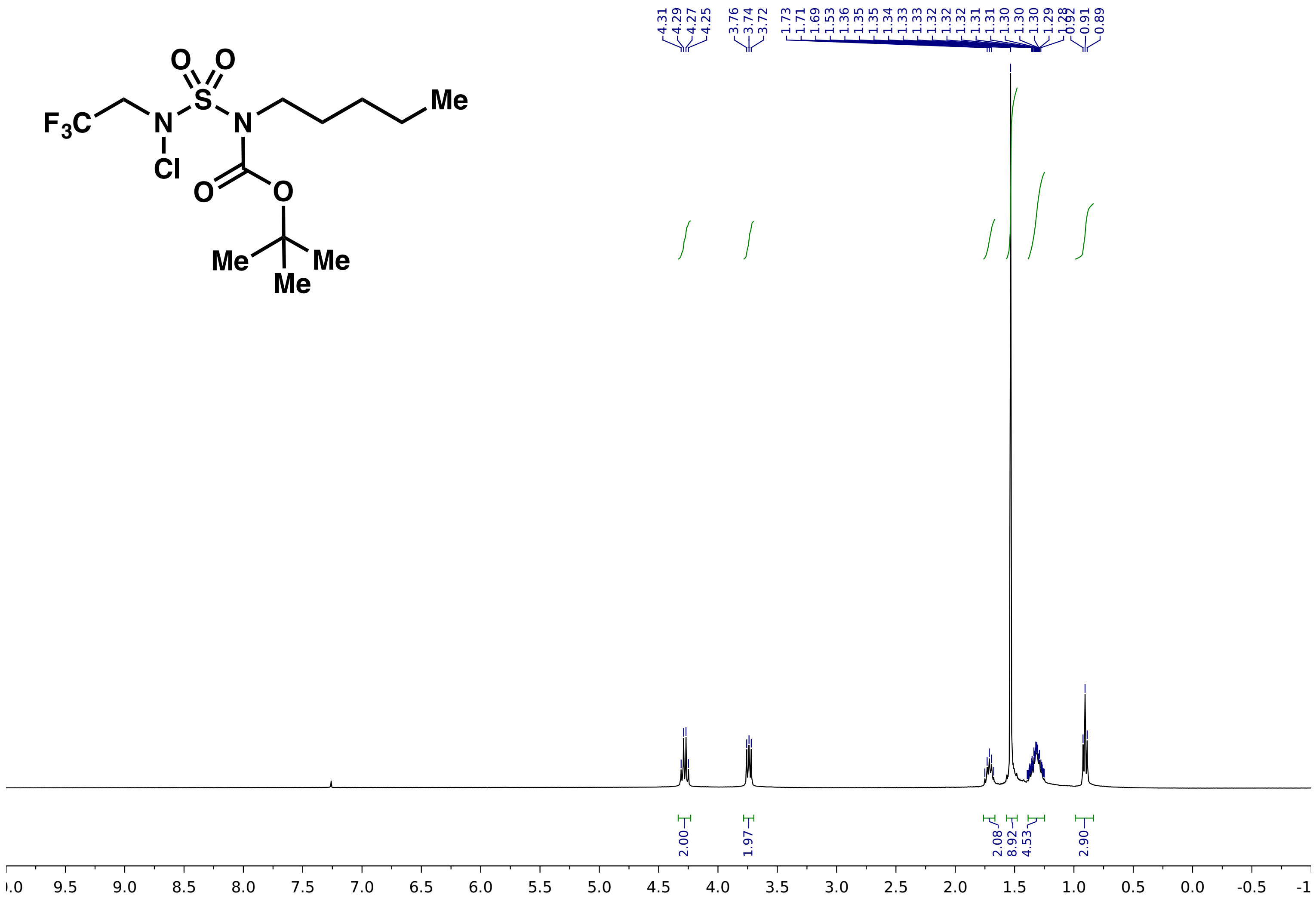

^{1}H NMR (400 MHz, $CDCl_3$) for *N*-*tert*-butoxycarbonyl-*N'*-chloro-*N'*-(2,2,2-trifluoroethyl)-*N*-pentylsulfamide (**S9v**)

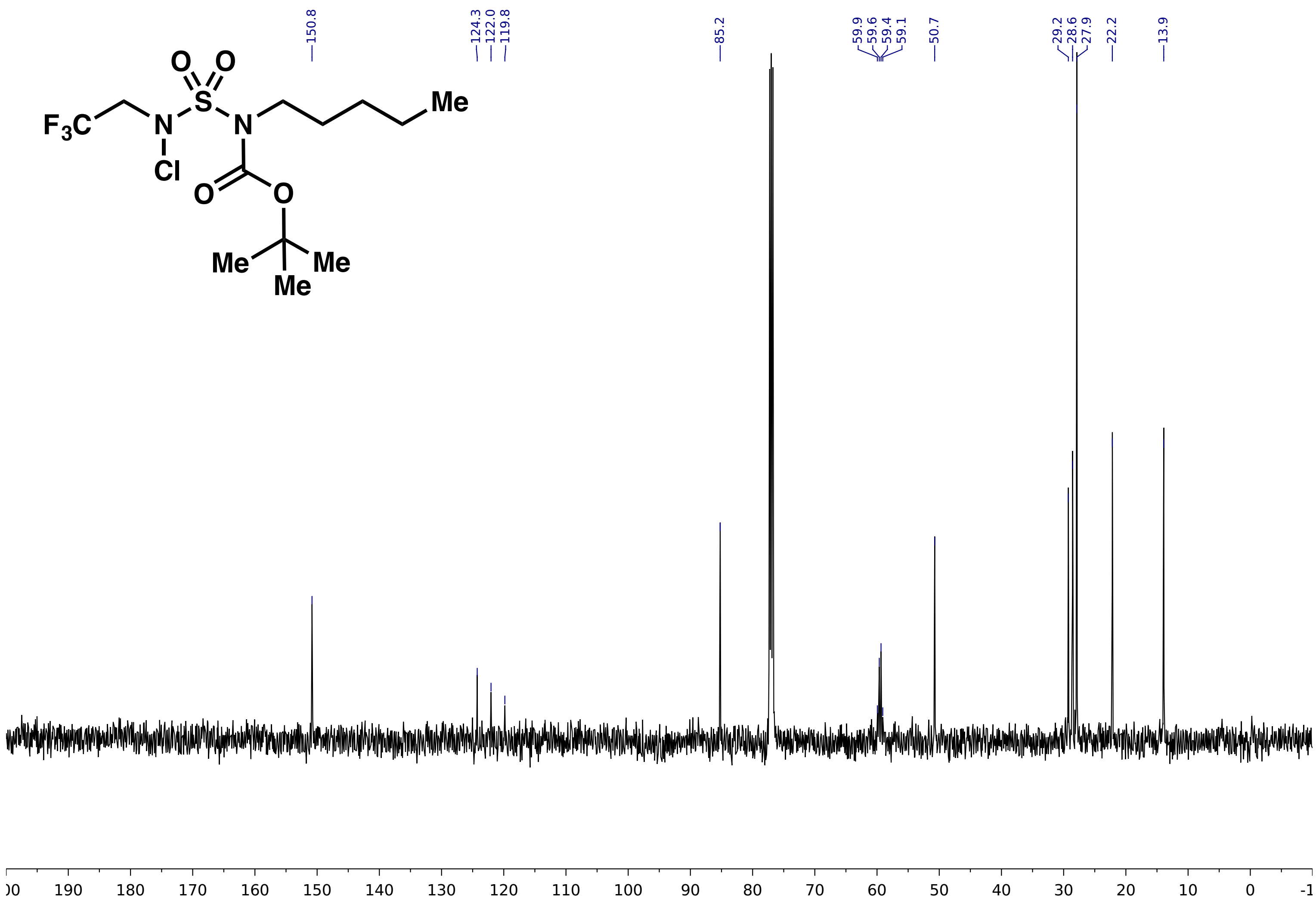

^{13}C NMR (126 MHz, $CDCl_3$) for *N*-*tert*-butoxycarbonyl-*N'*-chloro-*N'*-(2,2,2-trifluoroethyl)-*N*-pentylsulfamide (**S9v**)

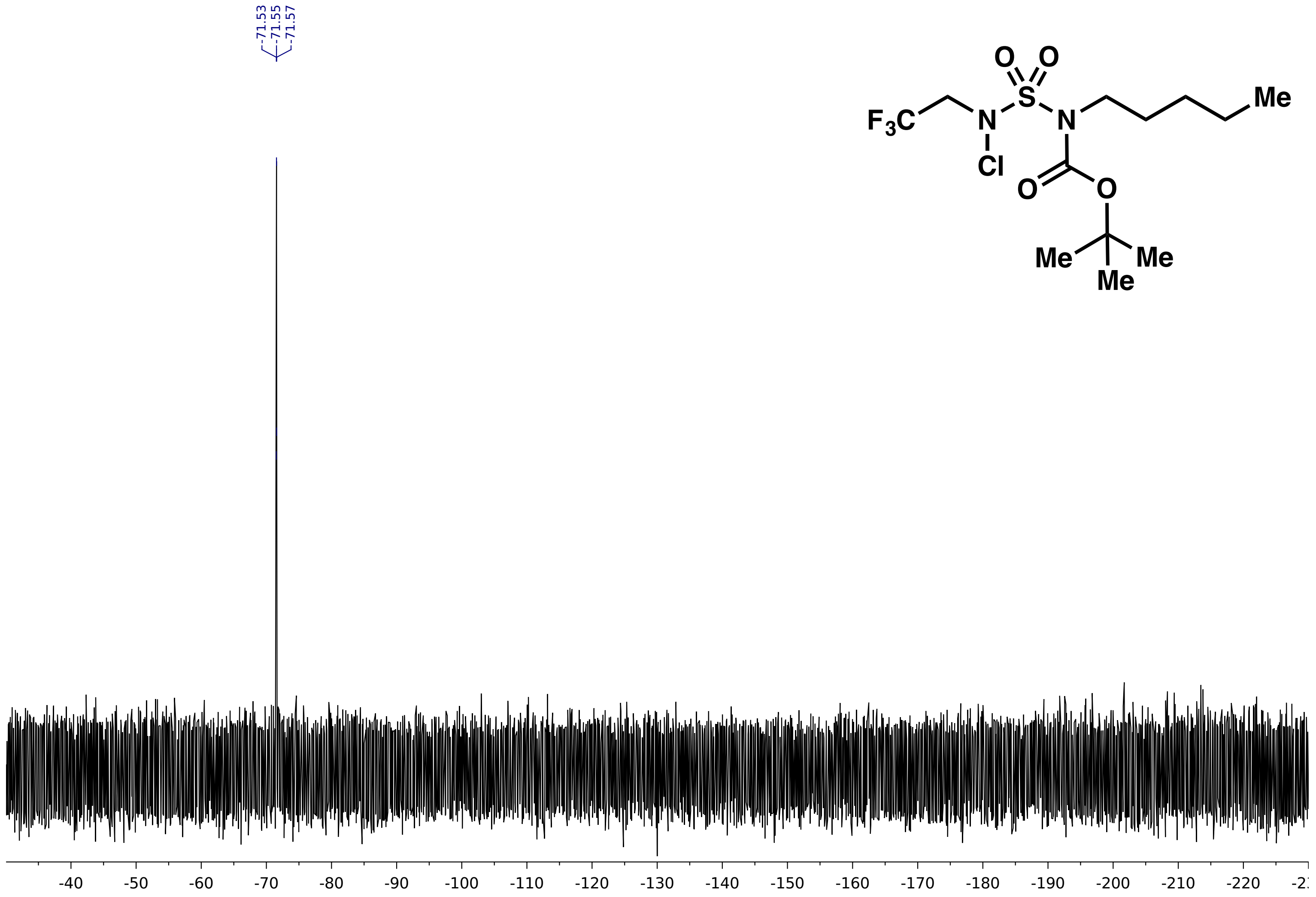

^{19}F NMR (376 MHz, $CDCl_3$) for *N*-*tert*-butoxycarbonyl-*N'*-chloro-*N'*-(2,2,2-trifluoroethyl)-*N*-pentylsulfamide (**S9v**)

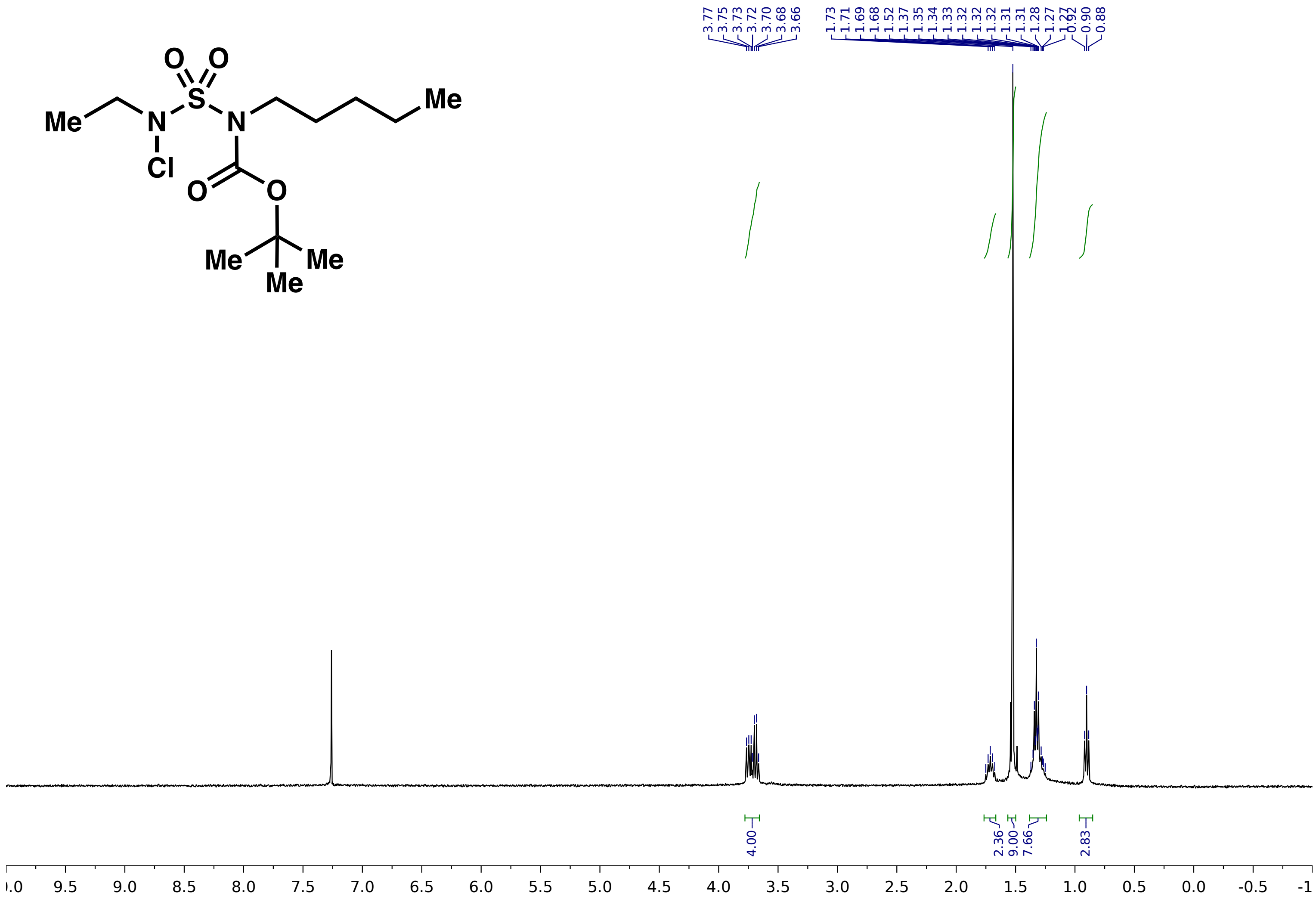

^{1}H NMR (400 MHz, $CDCl_3$) for *N*-*tert*-butoxycarbonyl-*N'*-chloro-*N'*-ethyl -*N*-pentylsulfamide (**S9w**)

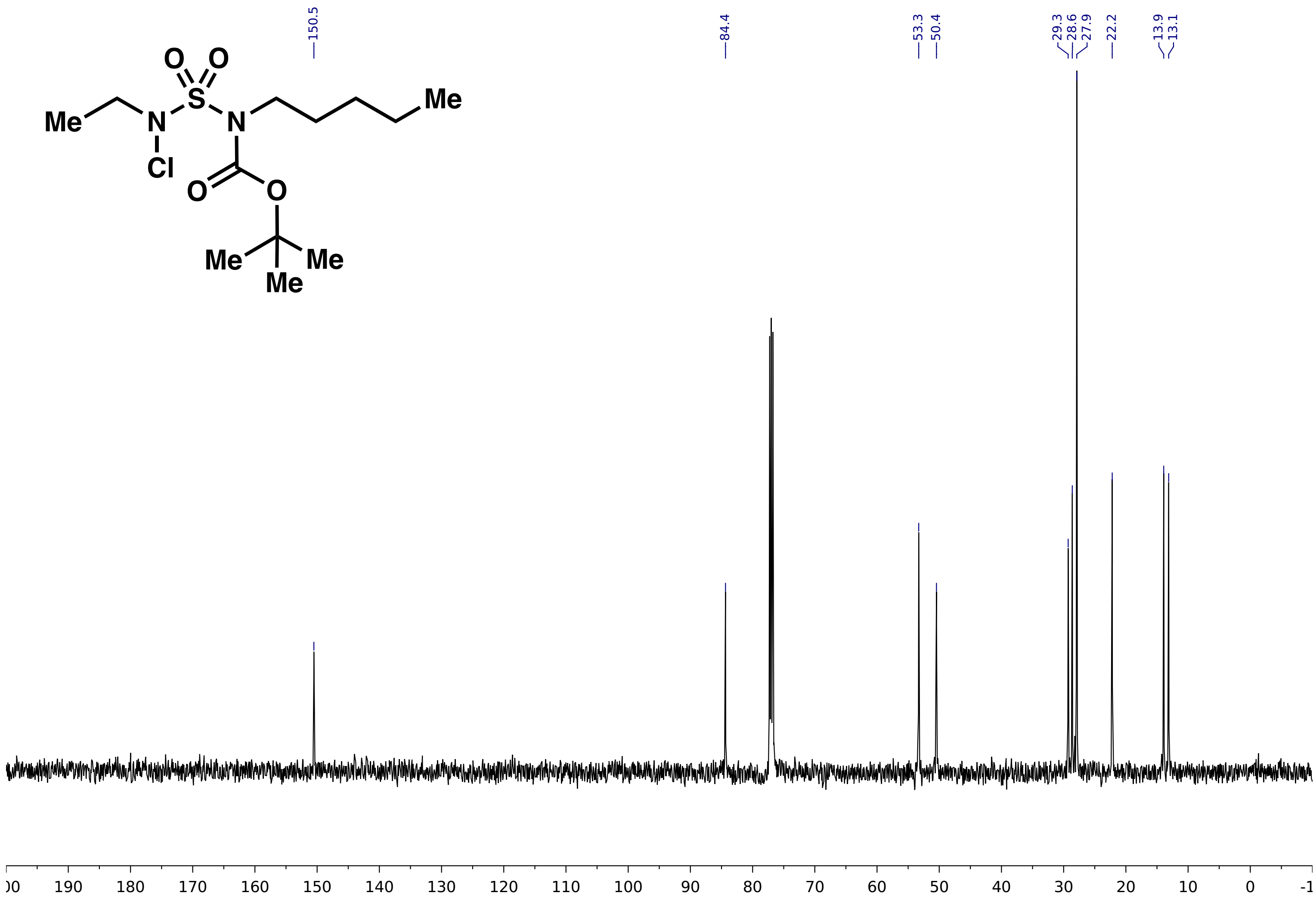

^{13}C NMR (126 MHz, $CDCl_3$) for *N*-*tert*-butoxycarbonyl-*N'*-chloro-*N'*-ethyl -*N*-pentylsulfamide (**S9w**)

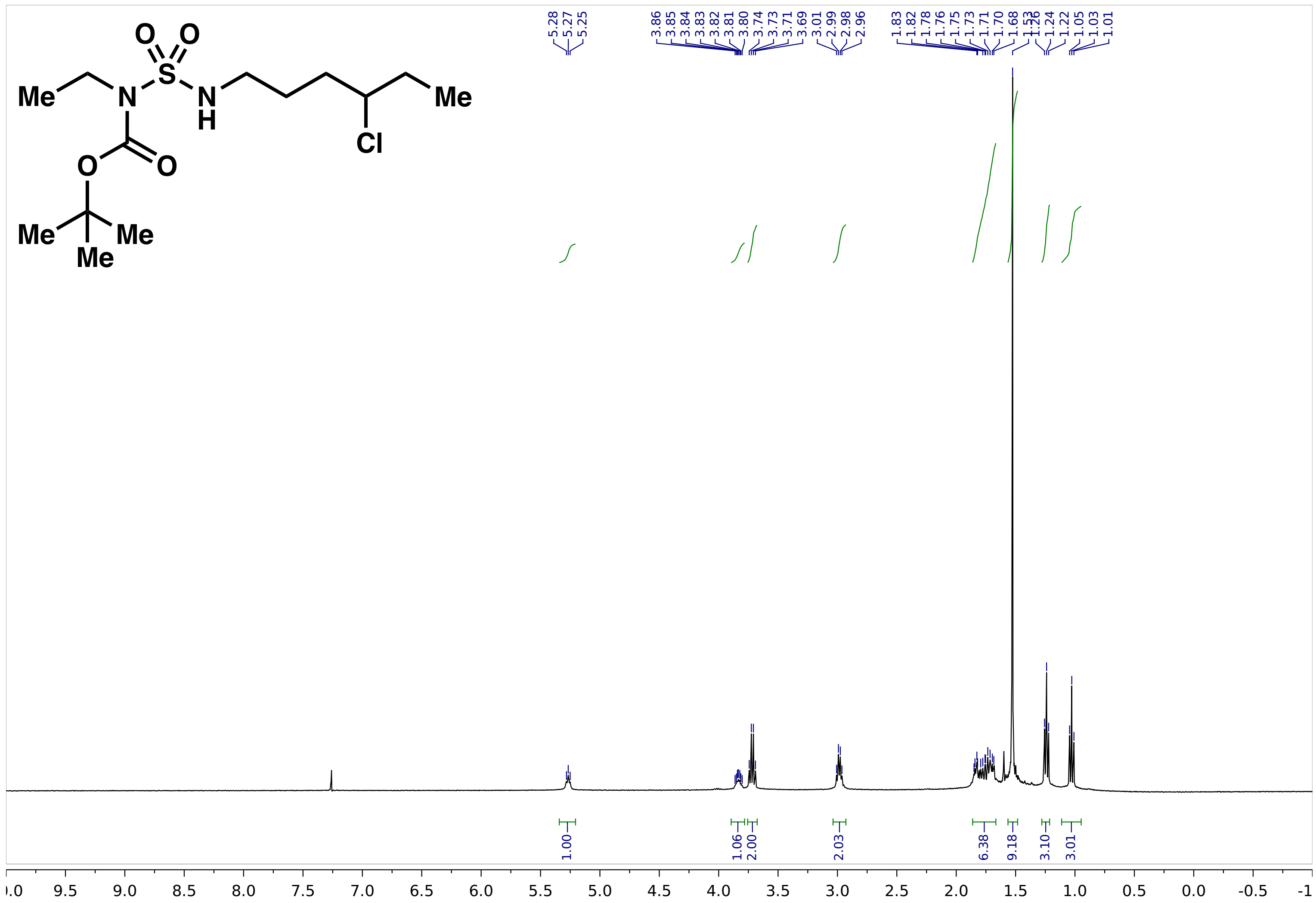

^{1}H NMR (400 MHz, $CDCl_3$) for *N*-*tert*-butoxycarbonyl-*N'*-(4-chloro)hexyl-*N*-ethylsulfamide (**6a**)

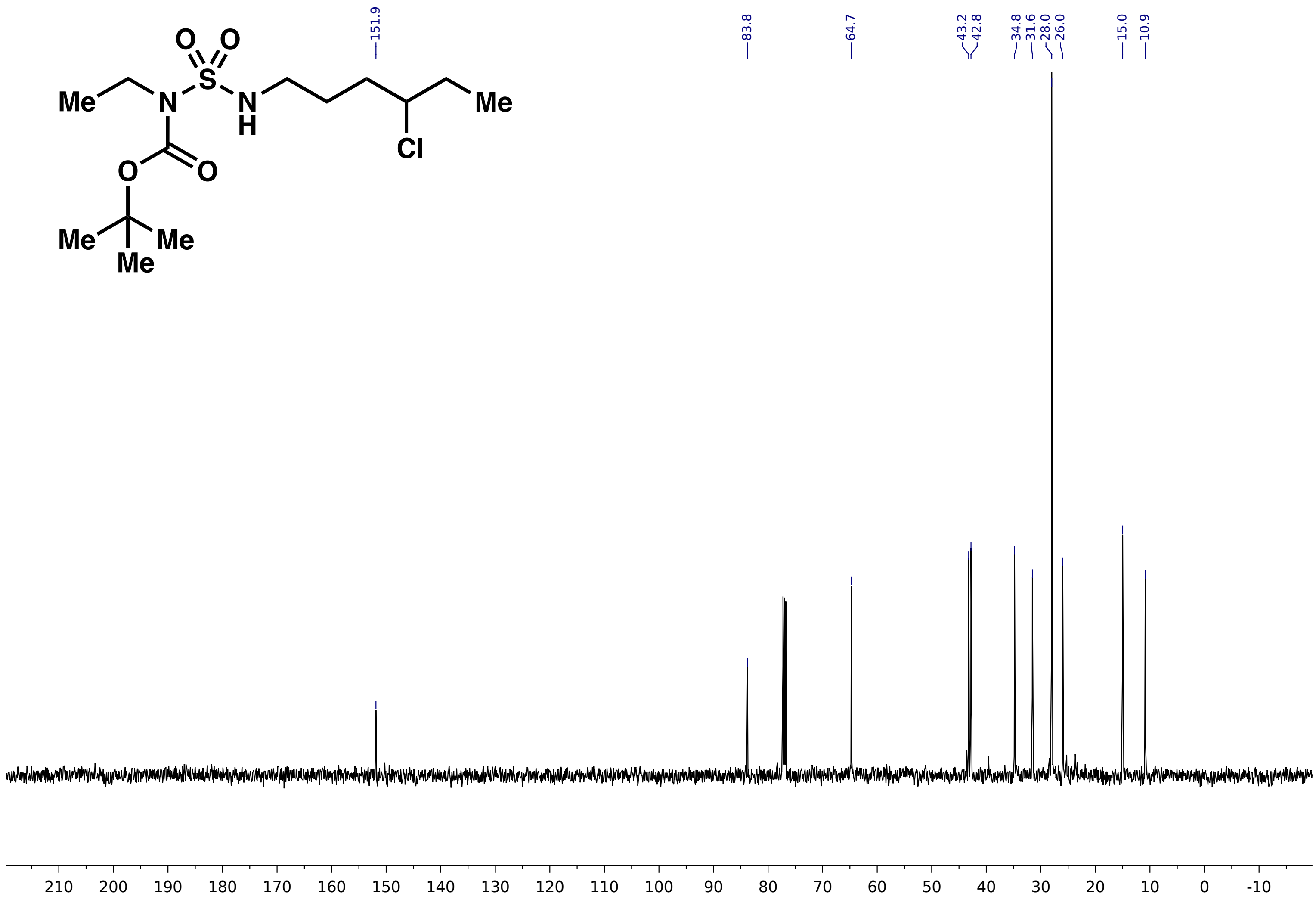

^{13}C NMR (126 MHz, $CDCl_3$) for *N*-*tert*-butoxycarbonyl-*N'*-(4-chloro)hexyl-*N*-ethylsulfamide (**6a**)

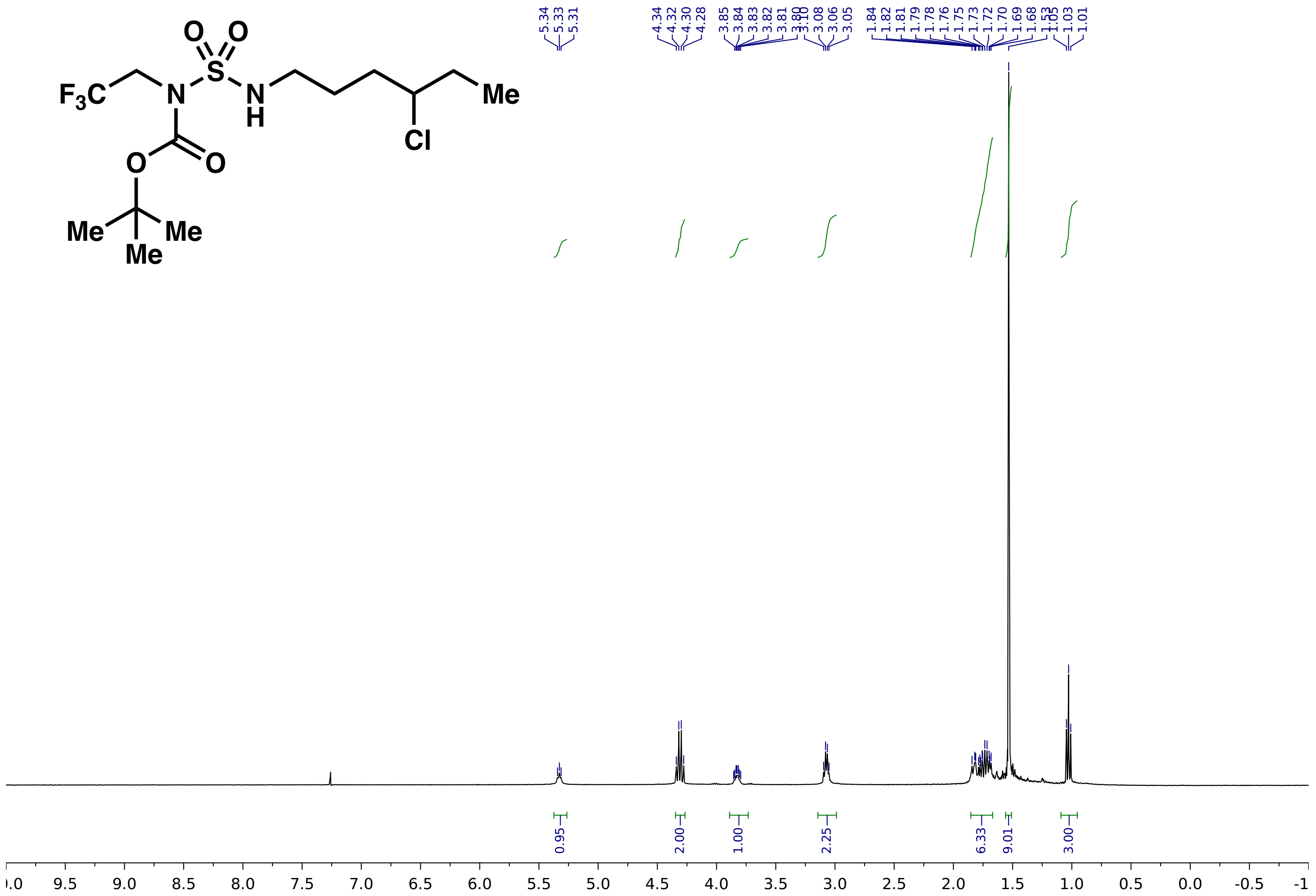

^{1}H NMR (400 MHz, $CDCl_3$) for *N*-*tert*-butoxycarbonyl-*N'*-(4-chloro)hexyl-*N*-(2,2,2-trifluoro)ethylsulfamide (**6b**)

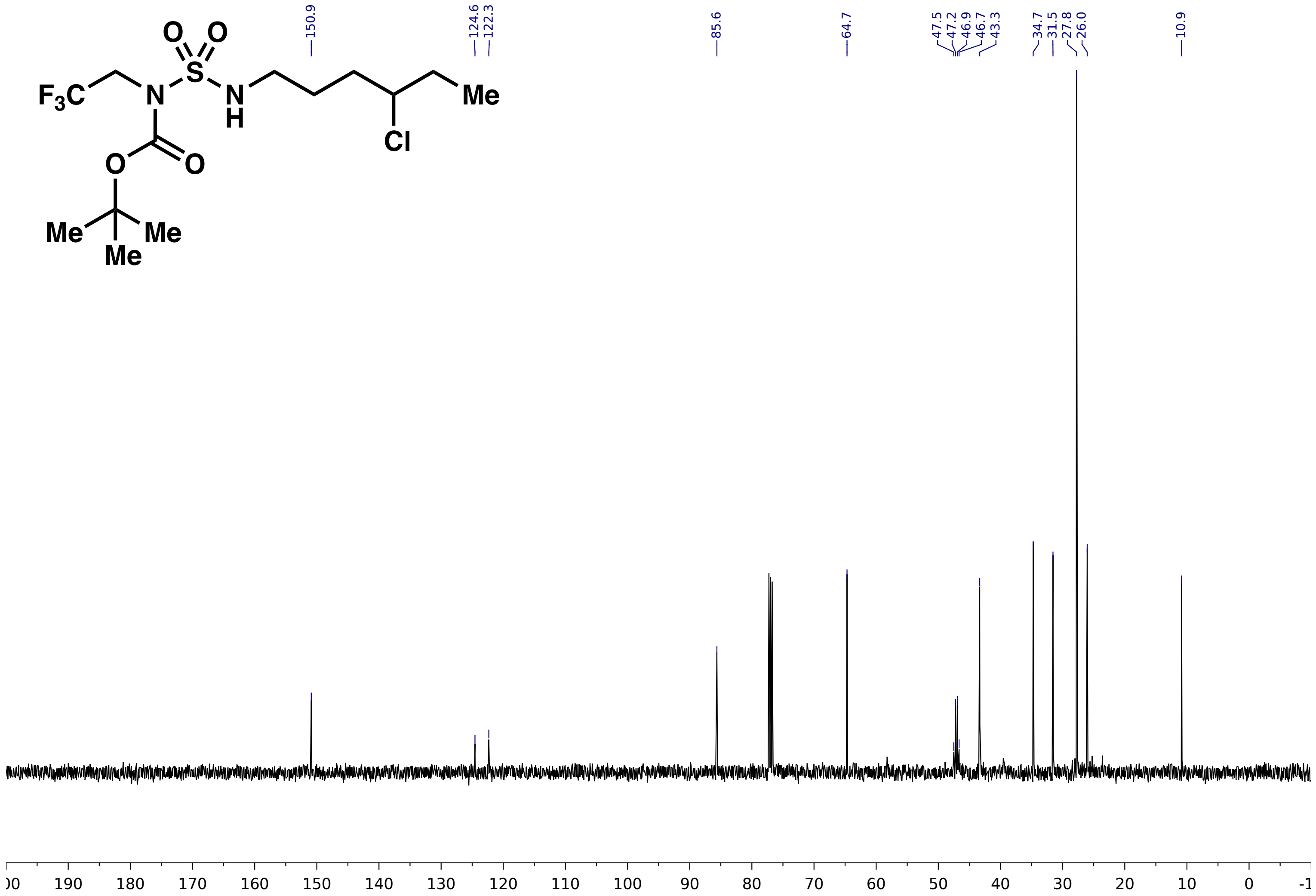

^{13}C NMR (126 MHz, $CDCl_3$) for *N*-*tert*-butoxycarbonyl-*N'*-(4-chloro)hexyl-*N*-(2,2,2-trifluoro)ethylsulfamide (**6b**)

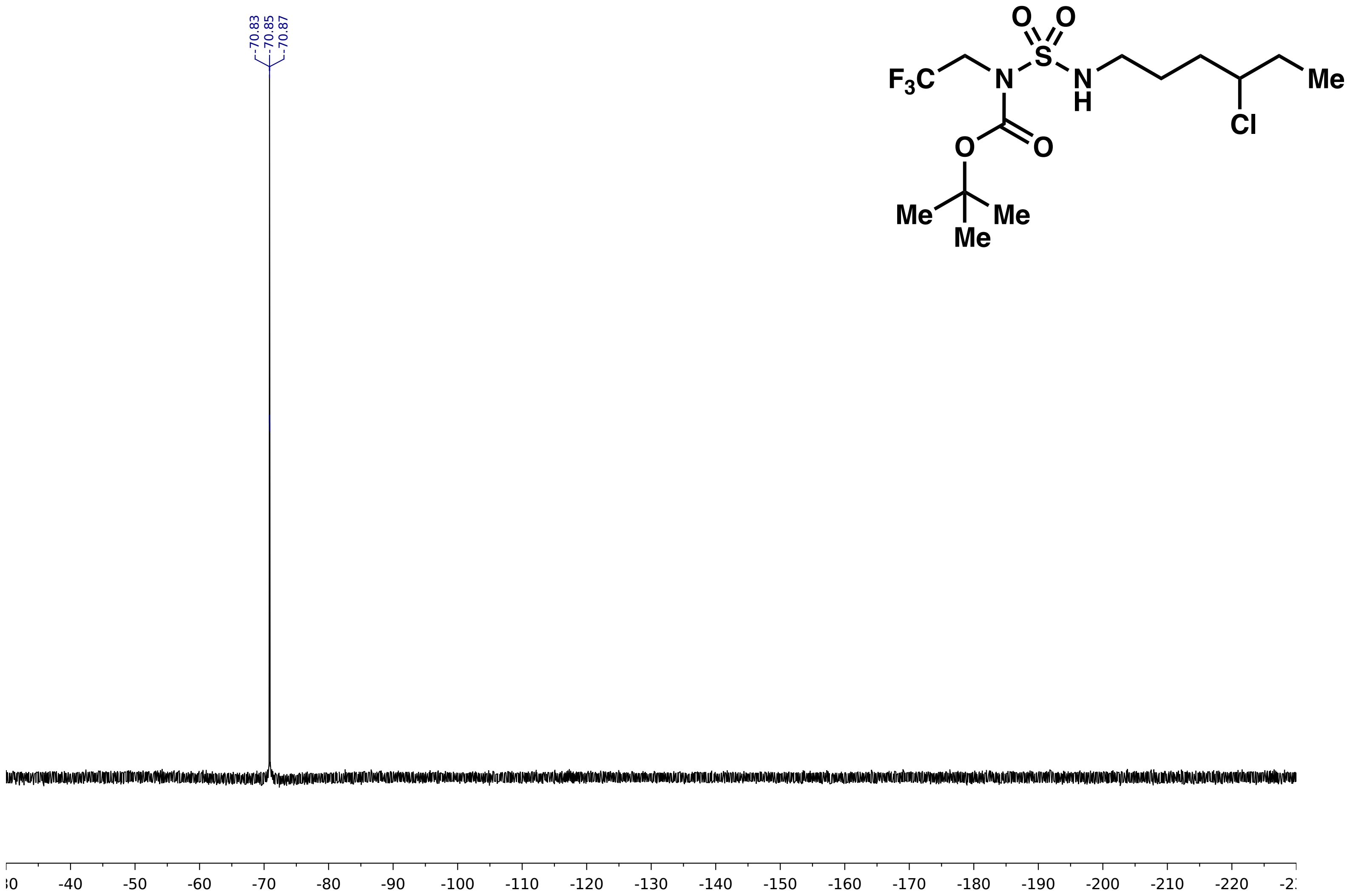

^{19}F NMR (376 MHz, $CDCl_3$) for *N*-*tert*-butoxycarbonyl-*N'*-(4-chloro)hexyl-*N*-(2,2,2-trifluoro)ethylsulfamide (**6b**)

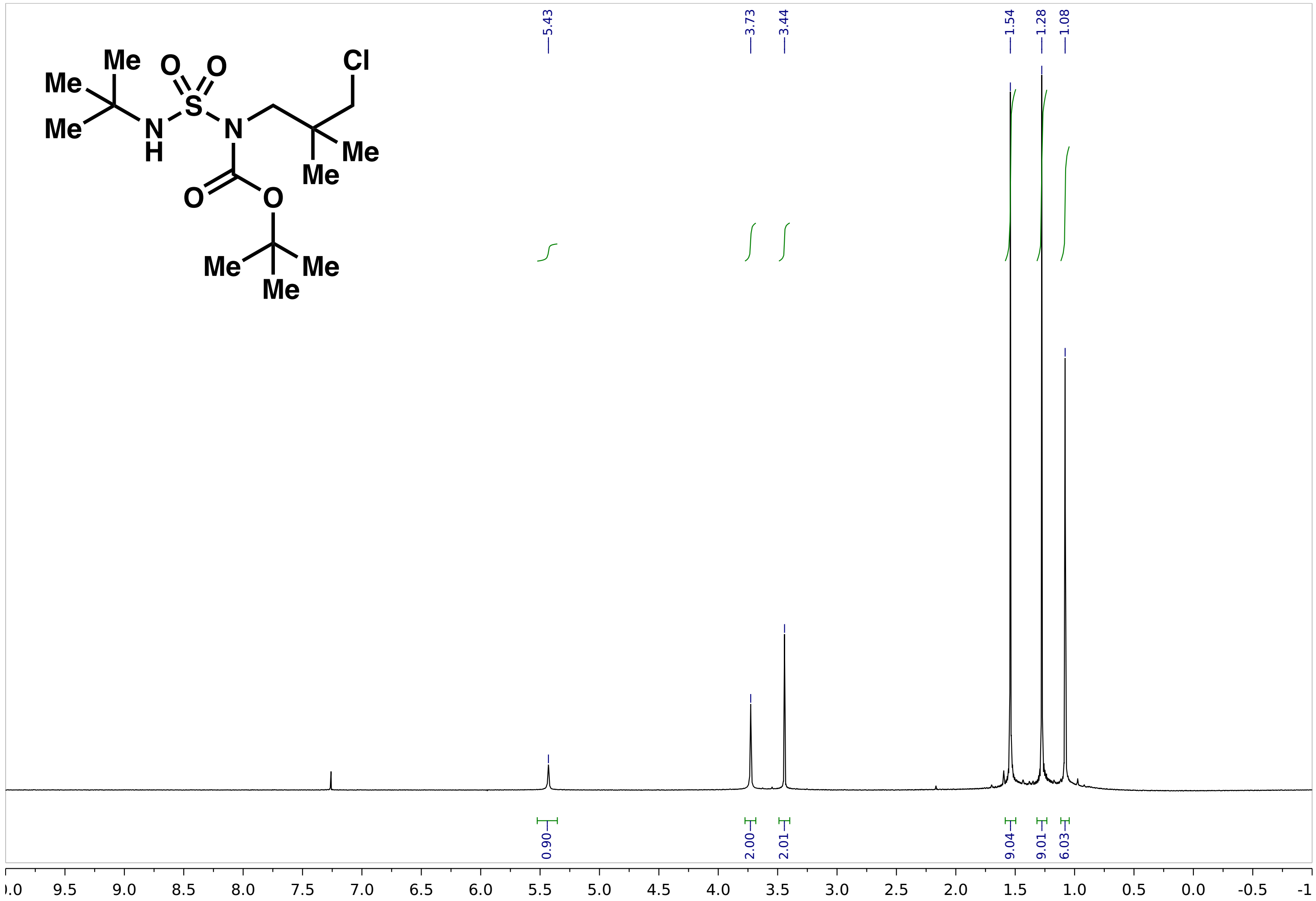

^{1}H NMR (400 MHz, $CDCl_3$) for *N*-*tert*-butoxycarbonyl-*N'*-*tert*-butyl-*N*-(3-chloro-2,2-dimethylprop-1-yl)sulfamide (**4a**)

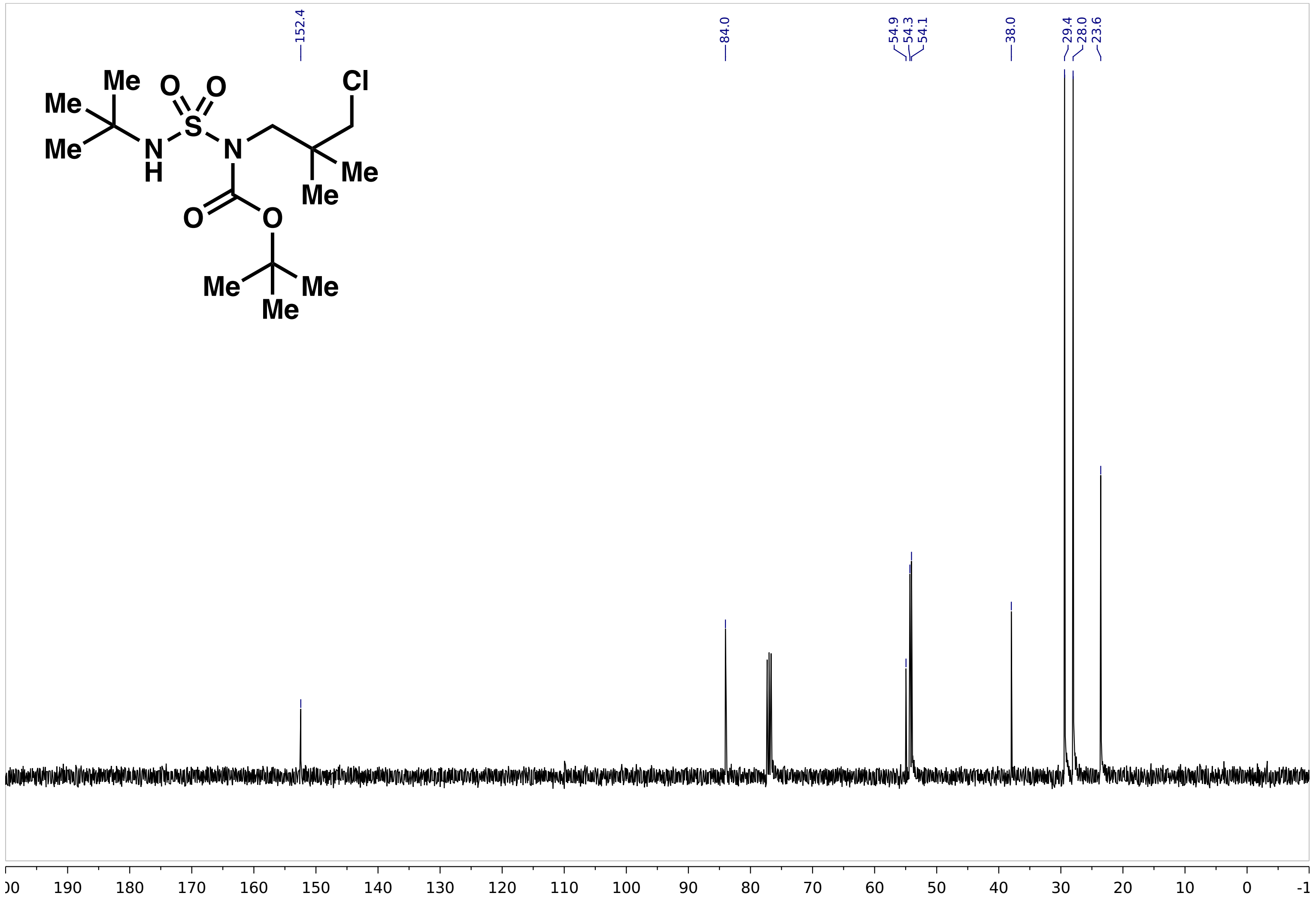

^{13}C NMR (101 MHz, $CDCl_3$) for *N*-*tert*-butoxycarbonyl-*N'*-*tert*-butyl-*N*-(3-chloro-2,2-dimethylprop-1-yl)sulfamide (**4a**)

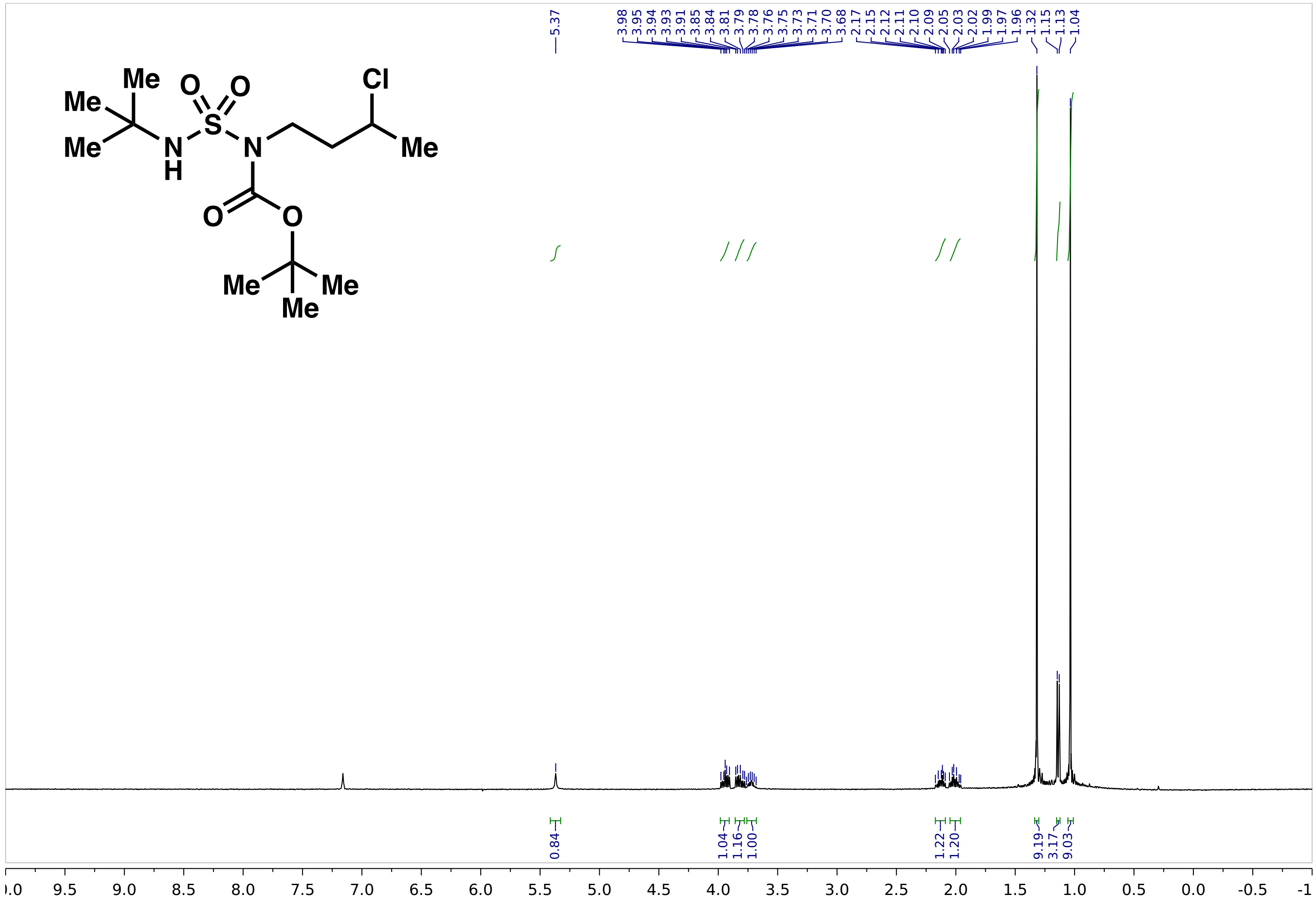

^{1}H NMR (400 MHz, C_6D_6) for *N*-*tert*-butoxycarbonyl-*N'*-*tert*-butyl-*N*-(3-chlorobut-1-yl)sulfamide (**4b**)

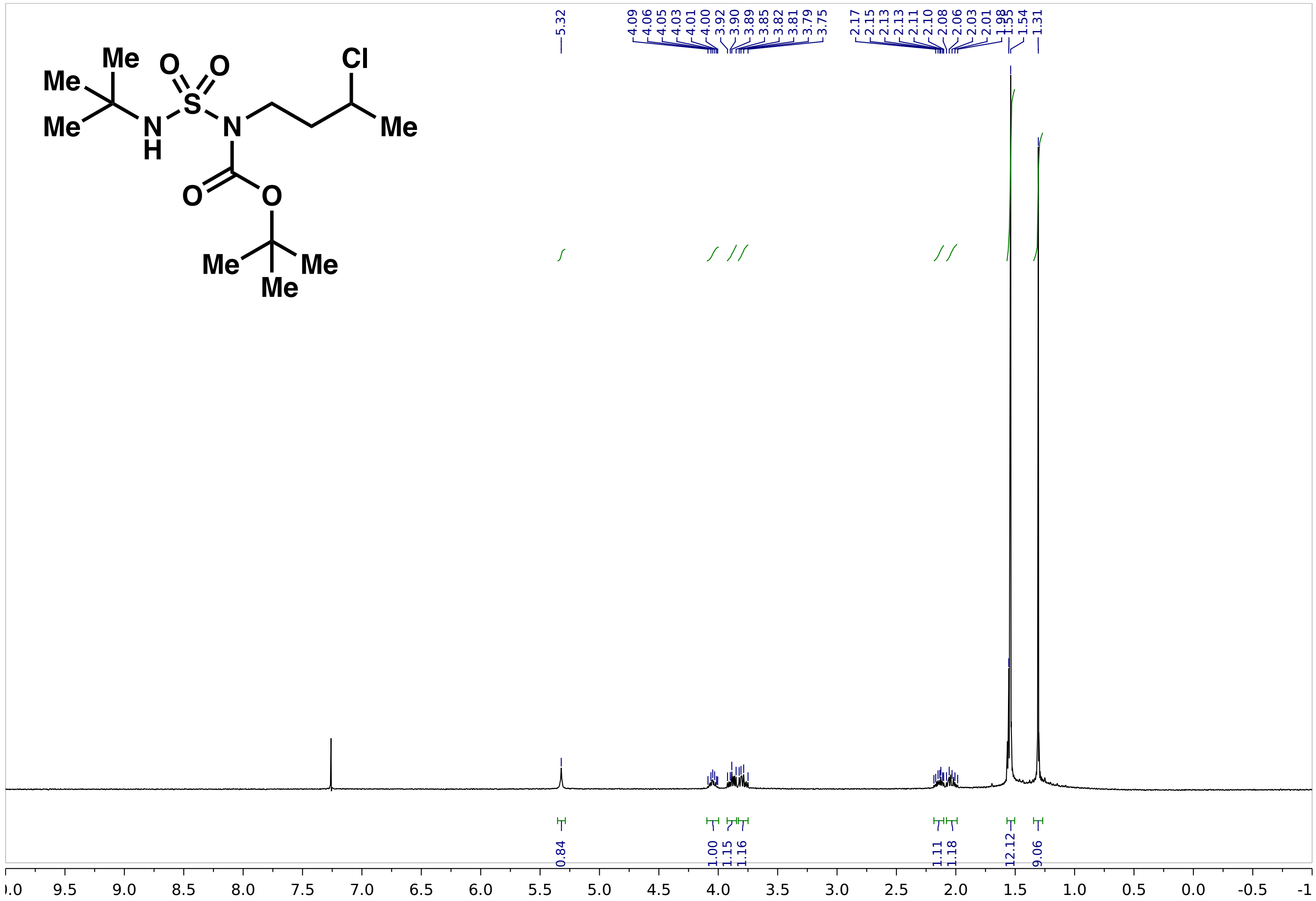

^{1}H NMR (400 MHz, CDCl$_3$) for *N*-*tert*-butoxycarbonyl-*N'*-*tert*-butyl-*N*-(3-chlorobut-1-yl)sulfamide (**4b**)

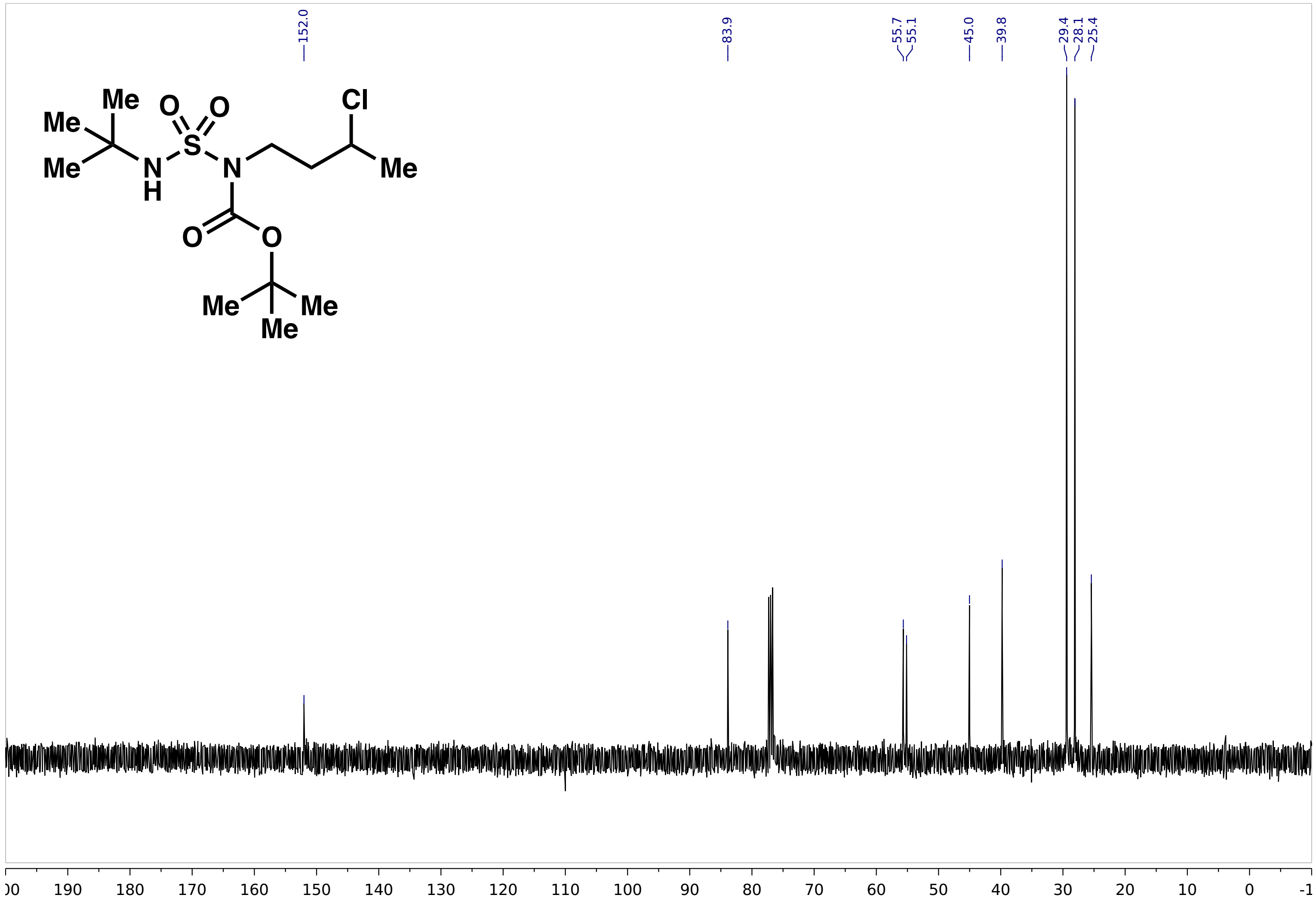

^{13}C NMR (101 MHz, $CDCl_3$) for *N*-*tert*-butoxycarbonyl-*N'*-*tert*-butyl-*N*-(3-chlorobut-1-yl)sulfamide (**4b**)

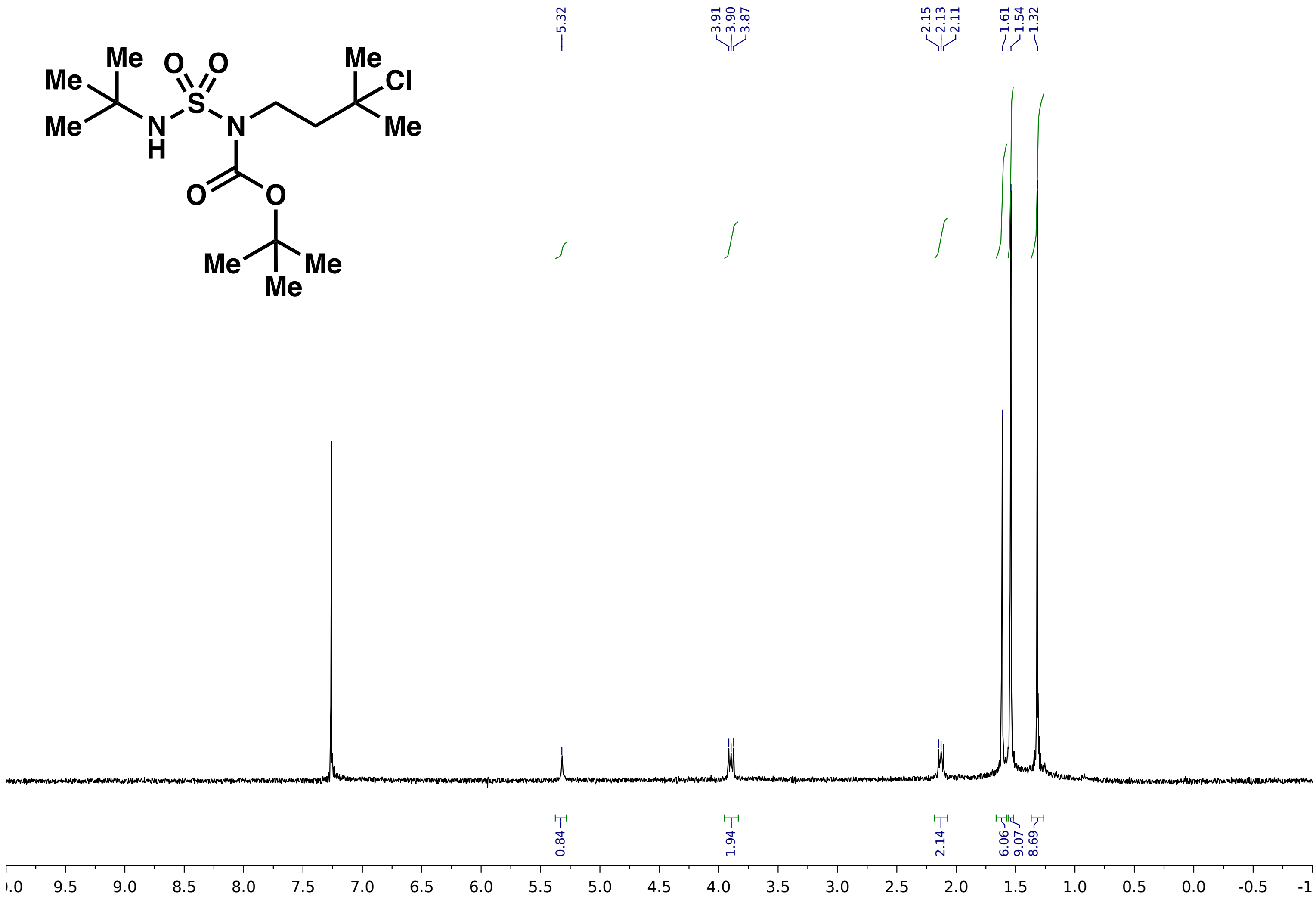

^{1}H NMR (400 MHz, $CDCl_3$) for *N*-*tert*-butoxycarbonyl-*N'*-*tert*-butyl-*N*-(3-chloro-3-methylbut-1-yl)sulfamide (**4c**)

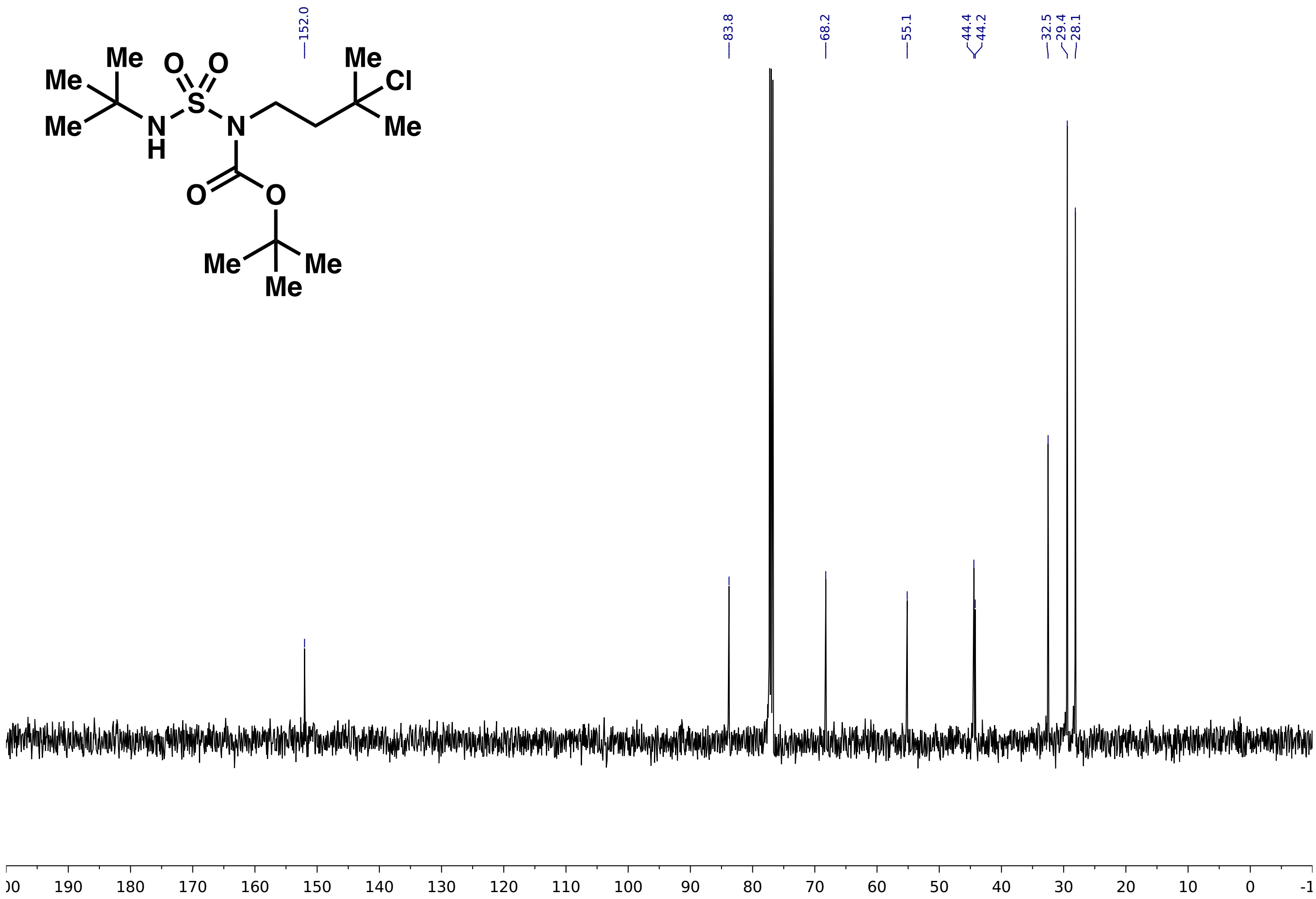

^{13}C NMR (126 MHz, $CDCl_3$) for *N*-*tert*-butoxycarbonyl-*N'*-*tert*-butyl-*N*-(3-chloro-3-methylbut-1-yl)sulfamide (**4c**)

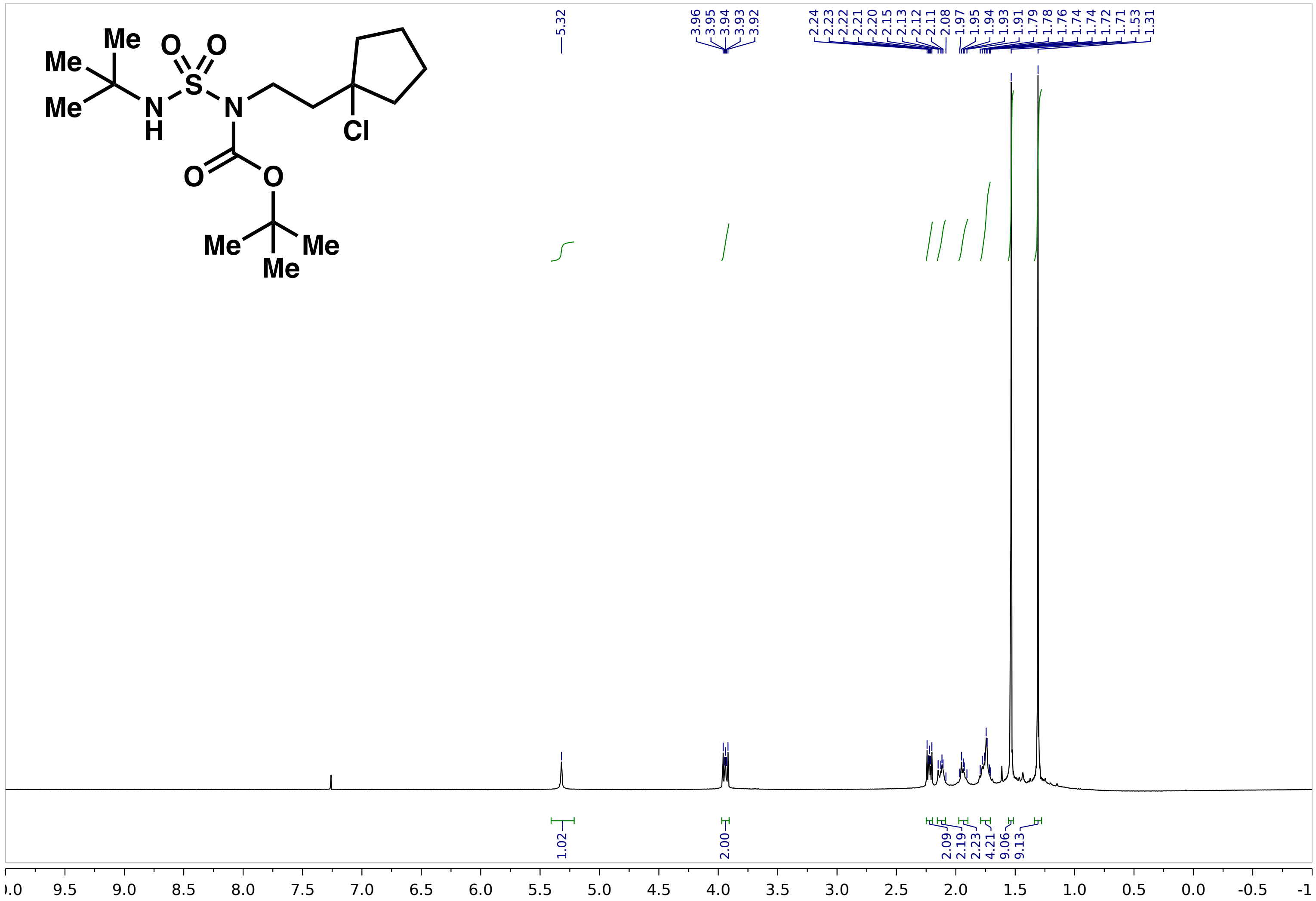

^{1}H NMR (400 MHz, $CDCl_3$) for *N*-*tert*-butoxycarbonyl-*N'*-*tert*-butyl-*N*-(2-(1-chlorocyclopent-1-yl)ethyl)sulfamide (**4d**)

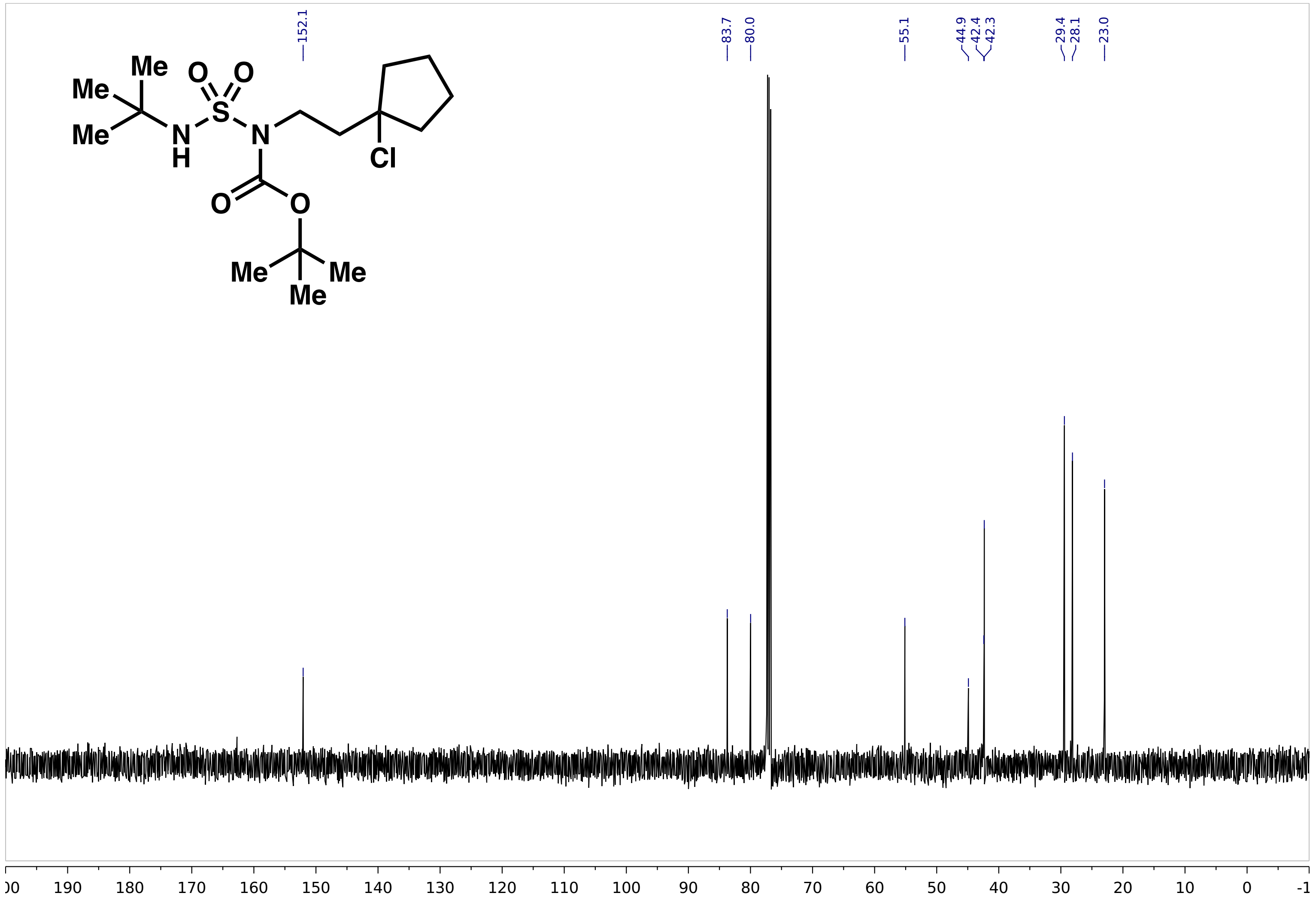

^{13}C NMR (126 MHz, $CDCl_3$) for *N*-*tert*-butoxycarbonyl-*N'*-*tert*-butyl-*N*-(2-(1-chlorocyclopent-1-yl)ethyl)sulfamide (**4d**)

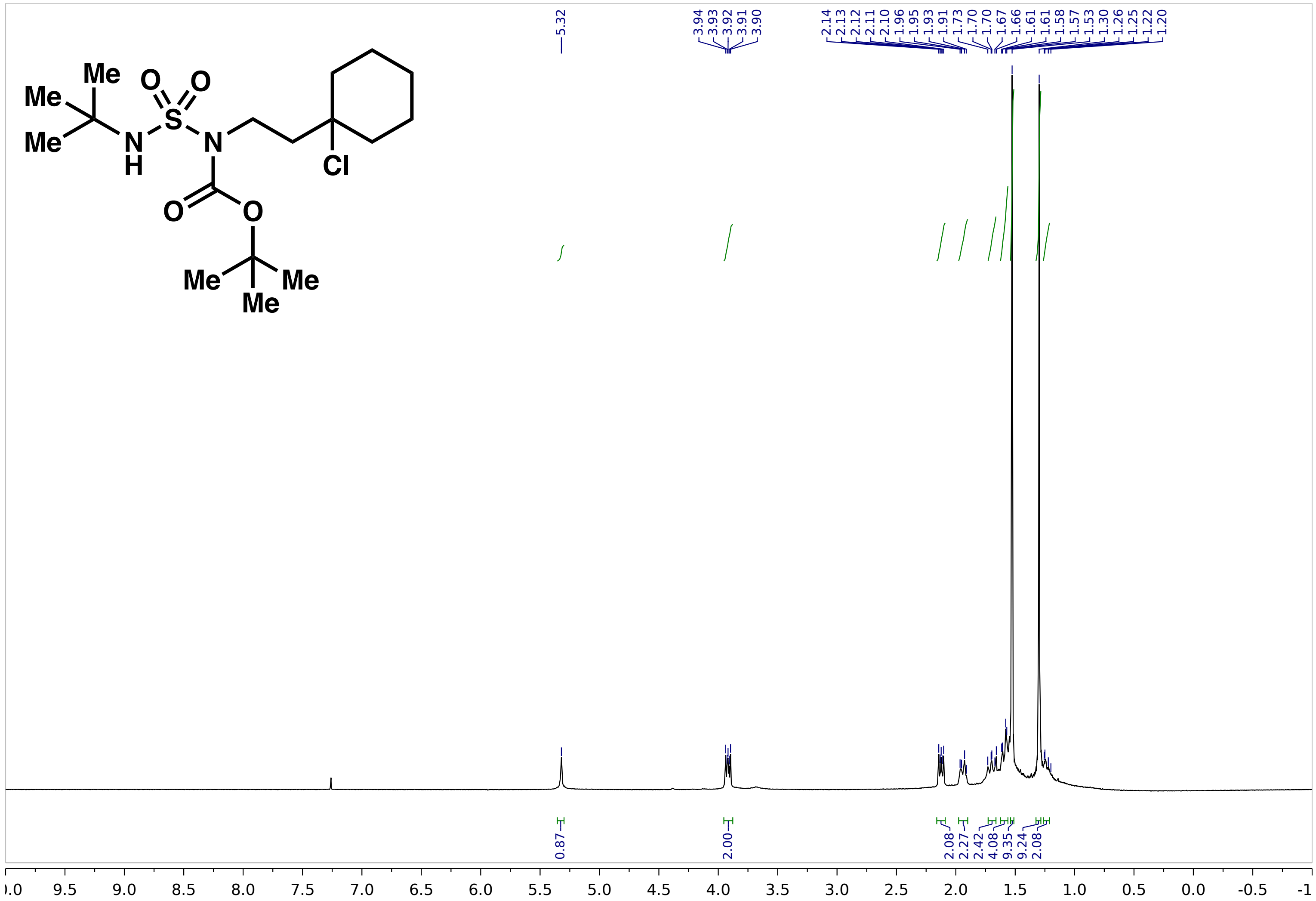

^{1}H NMR (400 MHz, $CDCl_3$) for *N*-*tert*-butoxycarbonyl-*N'*-*tert*-butyl-*N*-(2-(1-chlorocyclohex-1-yl)ethyl)sulfamide (**4e**)

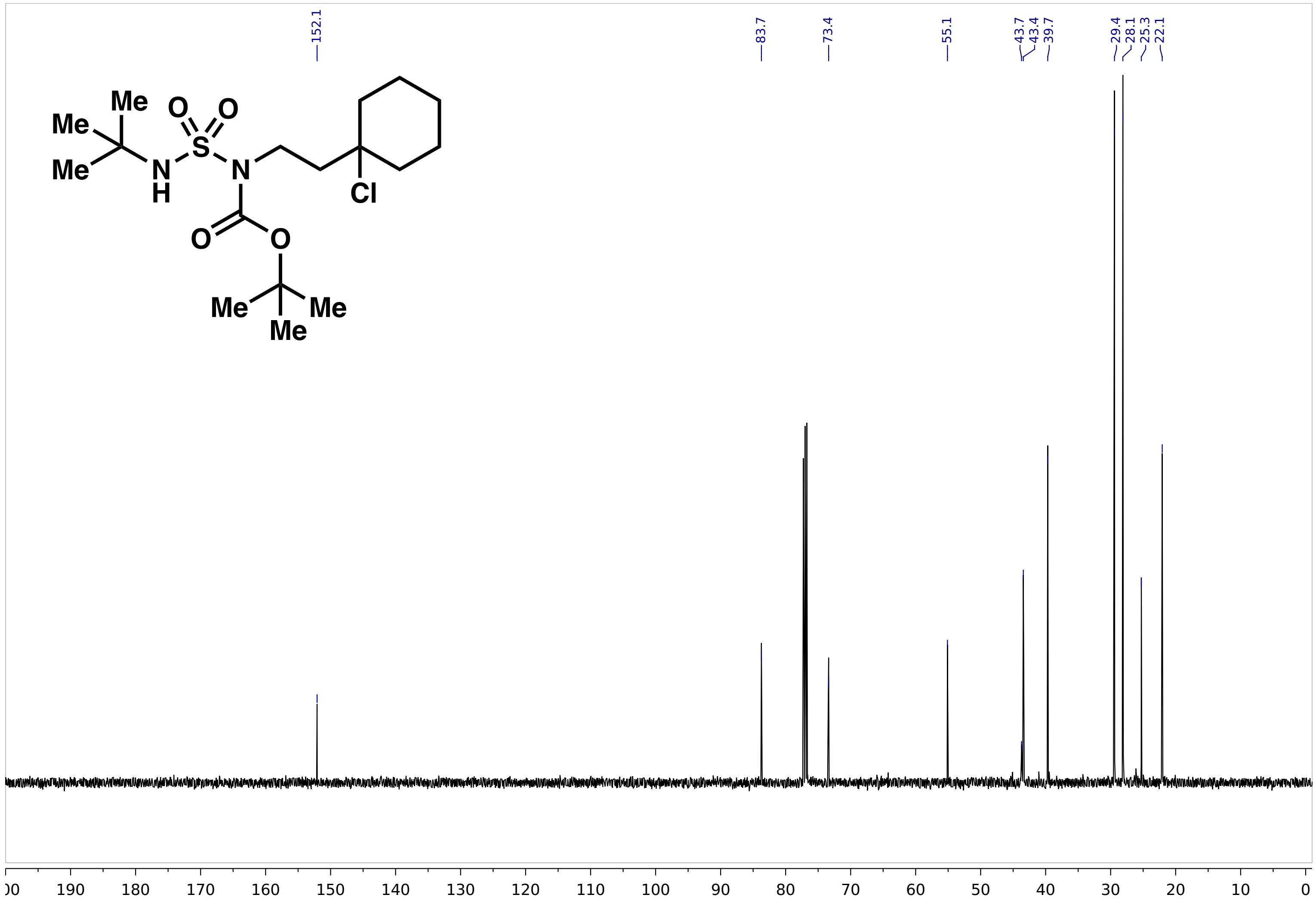

^{13}C NMR (126 MHz, $CDCl_3$) for *N*-*tert*-butoxycarbonyl-*N'*-*tert*-butyl-*N*-(2-(1-chlorocyclohex-1-yl)ethyl)sulfamide (**4e**)

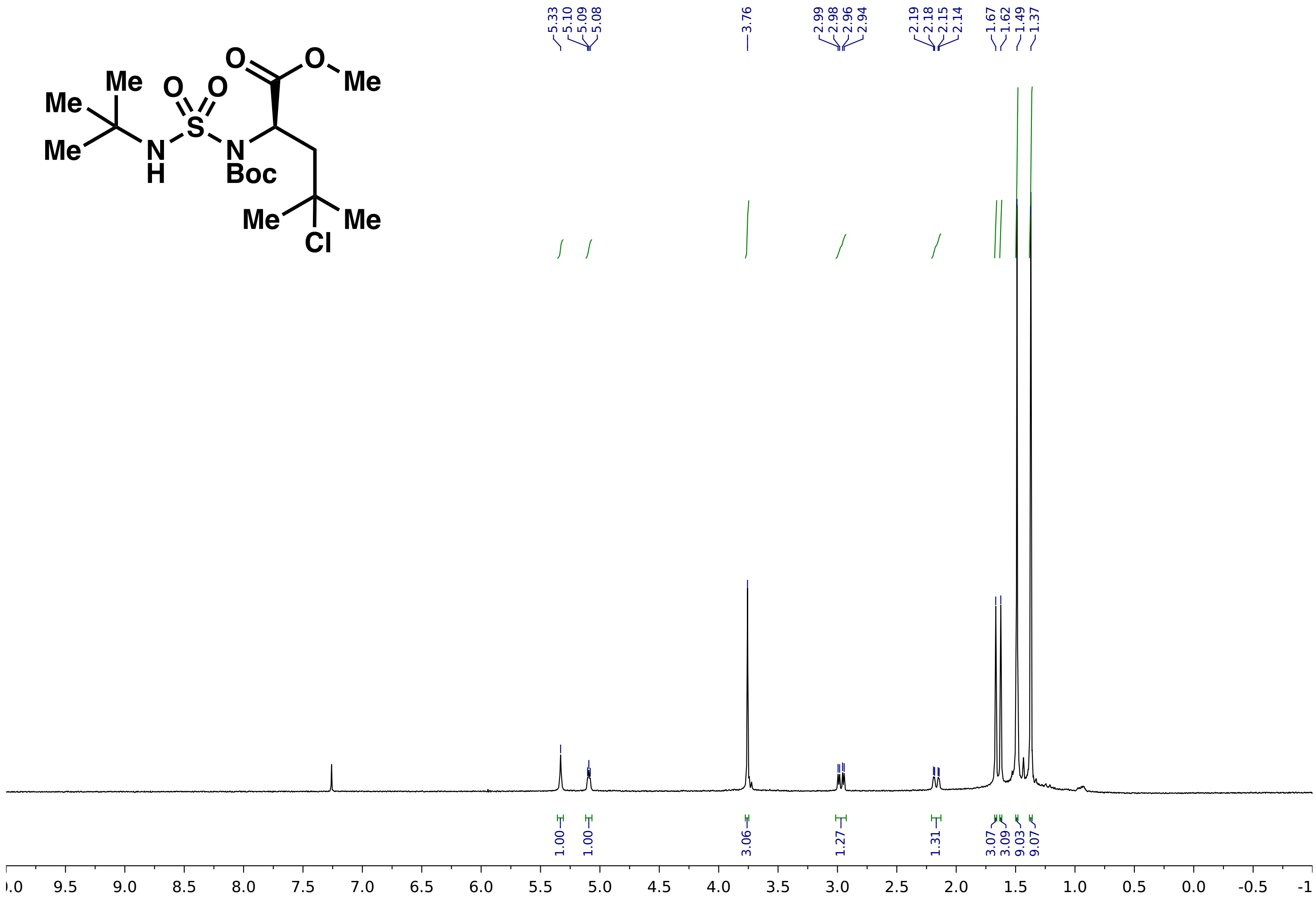

^{1}H NMR (400 MHz, CDCl$_3$) for *N-tert*-butoxycarbonyl-*N'*-*tert*-butyl-*N*-((*2R*)-methyl 4-chloro-4-methylpentano-2-yl)sulfamide (**4f**)

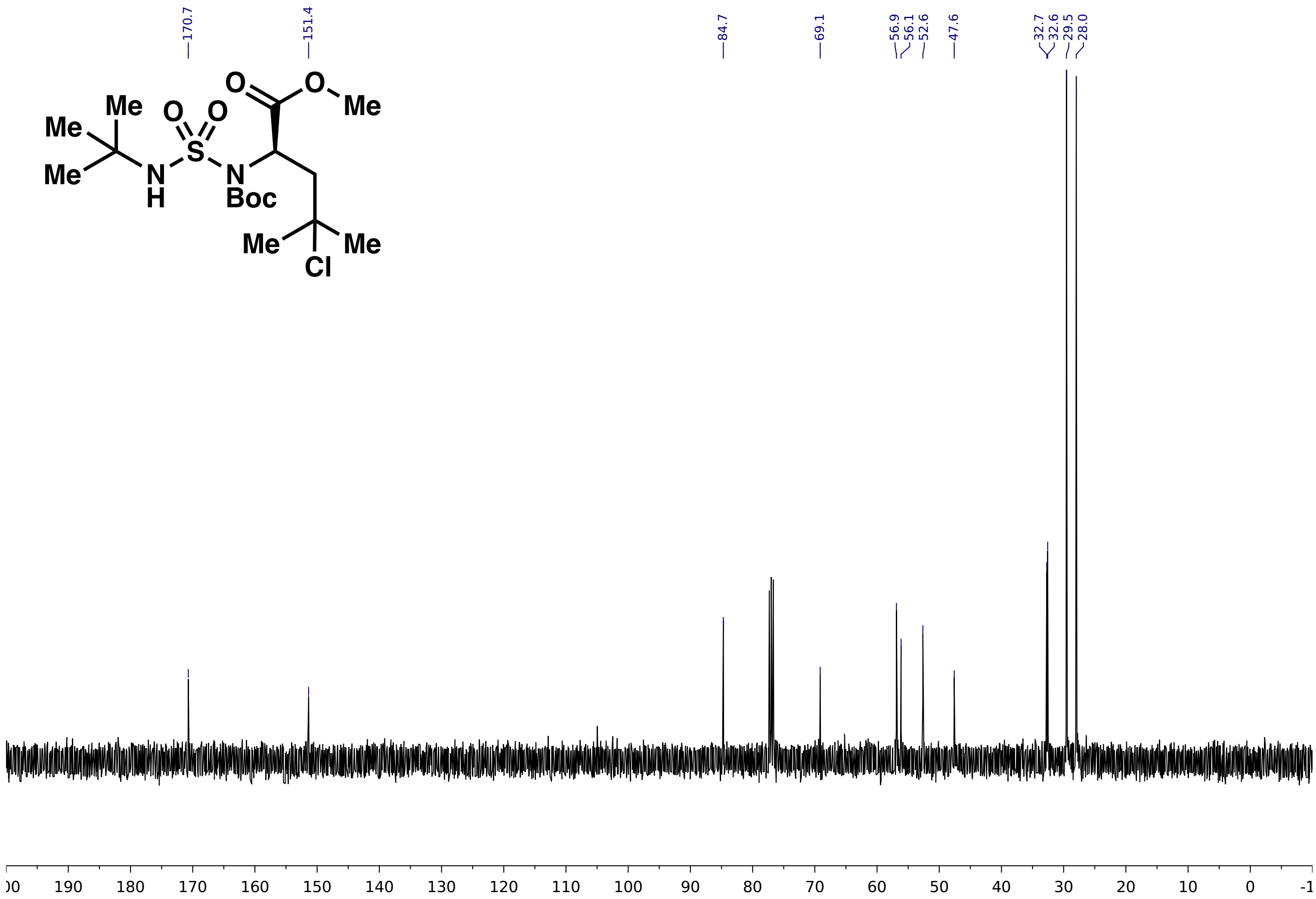

^{13}C NMR (101 MHz, $CDCl_3$) for *N*-*tert*-butoxycarbonyl-*N'*-*tert*-butyl-*N*-((2*R*)-methyl 4-chloro-4-methylpentano-2-yl)sulfamide (**4f**)

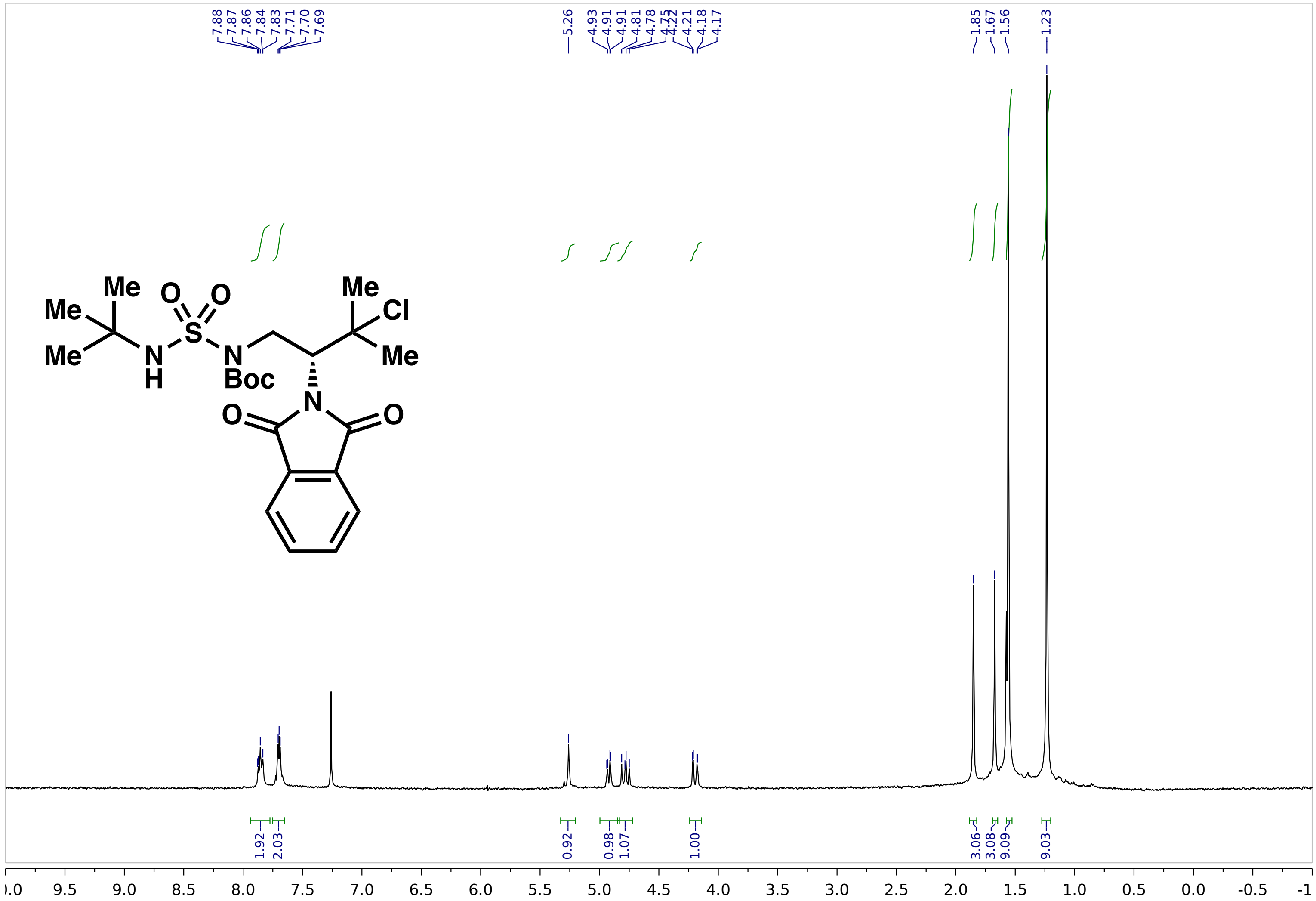

^{1}H NMR (400 MHz, $CDCl_3$) for *N*-*tert*-butoxycarbonyl-*N*'-*tert*-butyl-*N*-((*S*)-2-(1,3-dioxoisoindolin-2-yl)-3-chloro-3-methylbutyl)sulfamide (**4g**)

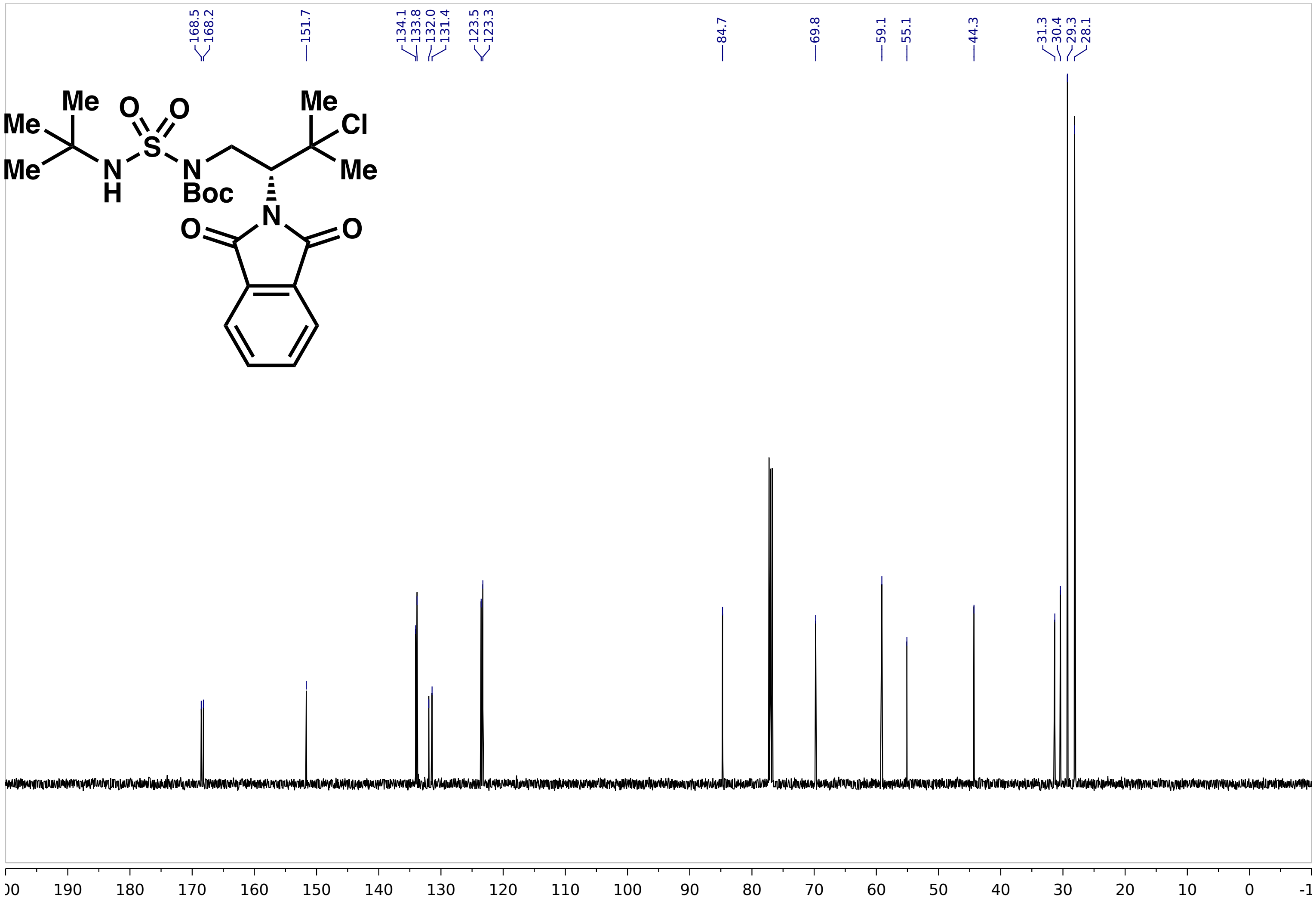

^{13}C NMR (126 MHz, $CDCl_3$) for *N*-*tert*-butoxycarbonyl-*N*'-*tert*-butyl-*N*-((*S*)-2-(1,3-dioxoisoindolin-2-yl)-3-chloro-3-methylbutyl)sulfamide (**4g**)

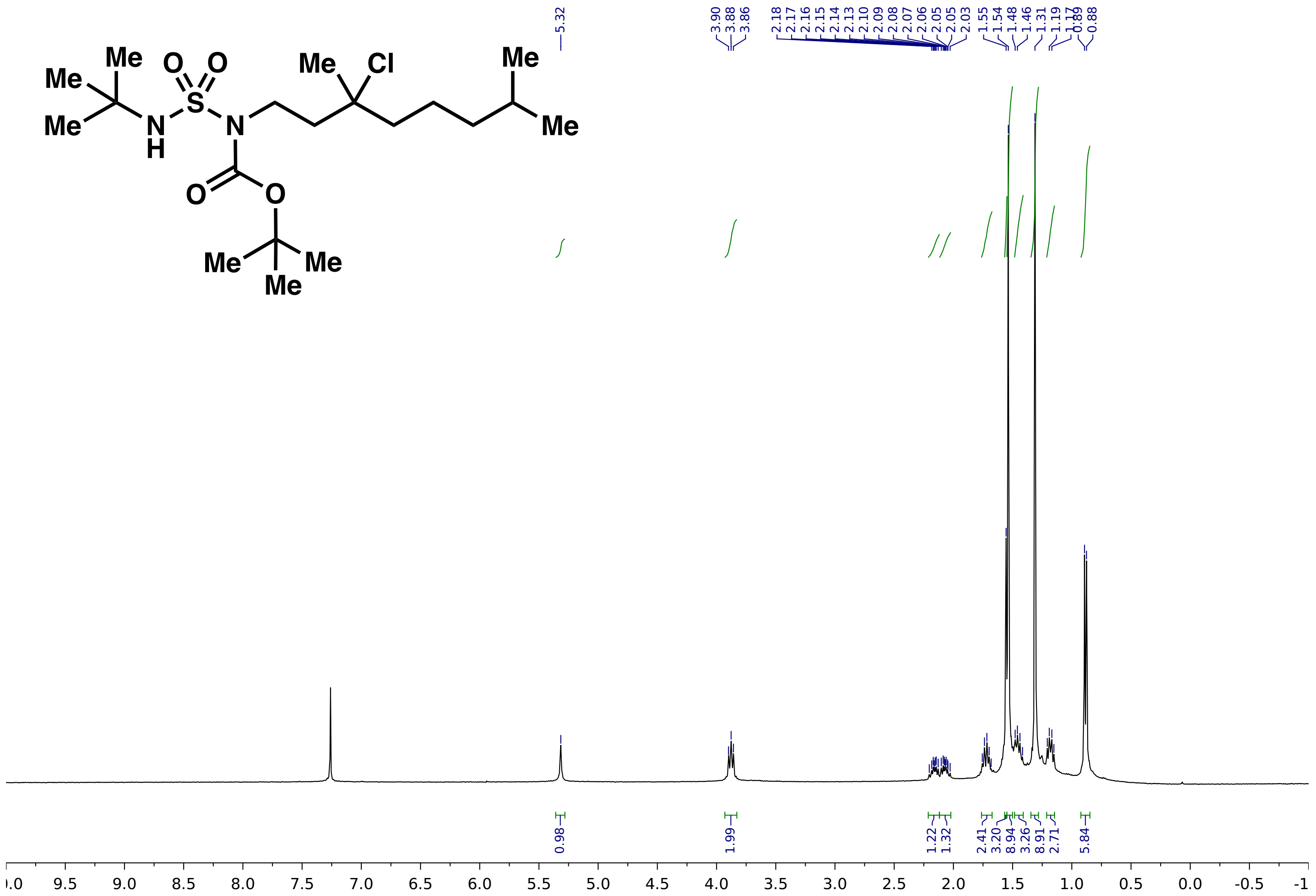

^{1}H NMR (400 MHz, $CDCl_3$) for *N*-*tert*-butoxycarbonyl-*N'*-*tert*-butyl-*N*-(3-chloro-3,7-dimethyloct-1-yl)sulfamide (**4h**)

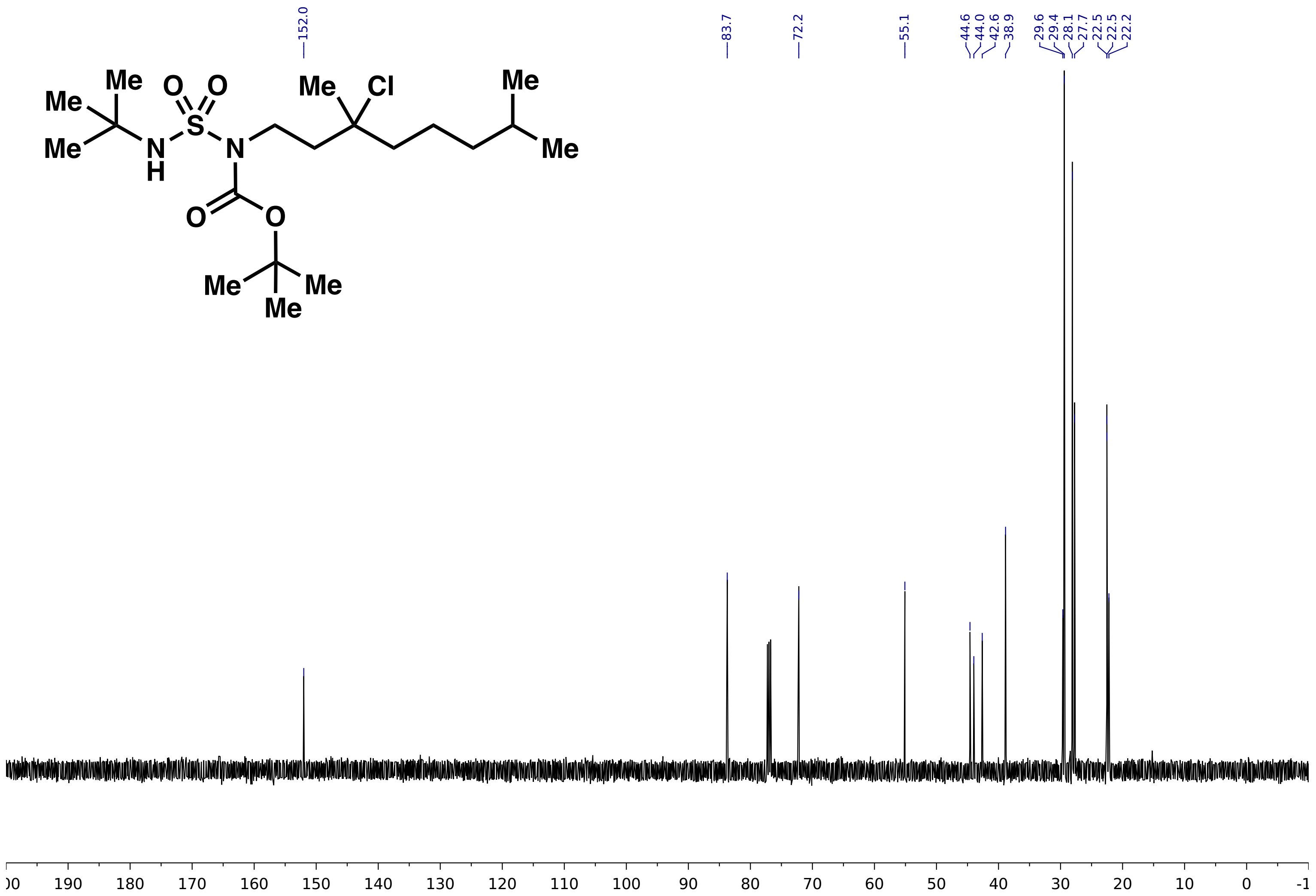

^{13}C NMR (126 MHz, $CDCl_3$) for *N*-*tert*-butoxycarbonyl-*N'*-*tert*-butyl-*N*-(3-chloro-3,7-dimethyloct-1-yl)sulfamide (**4h**)

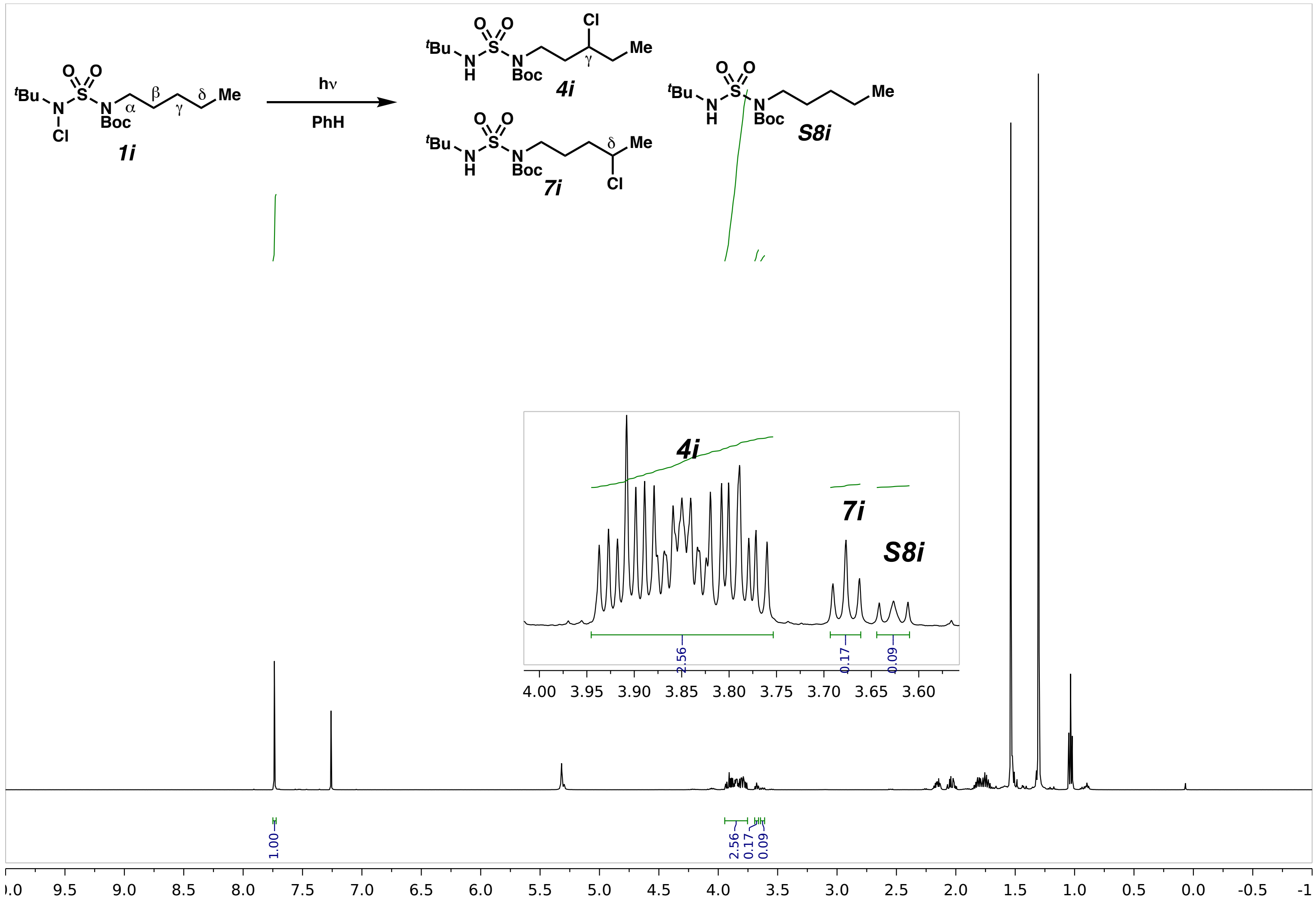

Crude ^{1}H NMR (500 MHz, $CDCl_3$, 25s relaxation delay) for chlorine-transfer reaction of *N*-chlorosulfamide **1i**

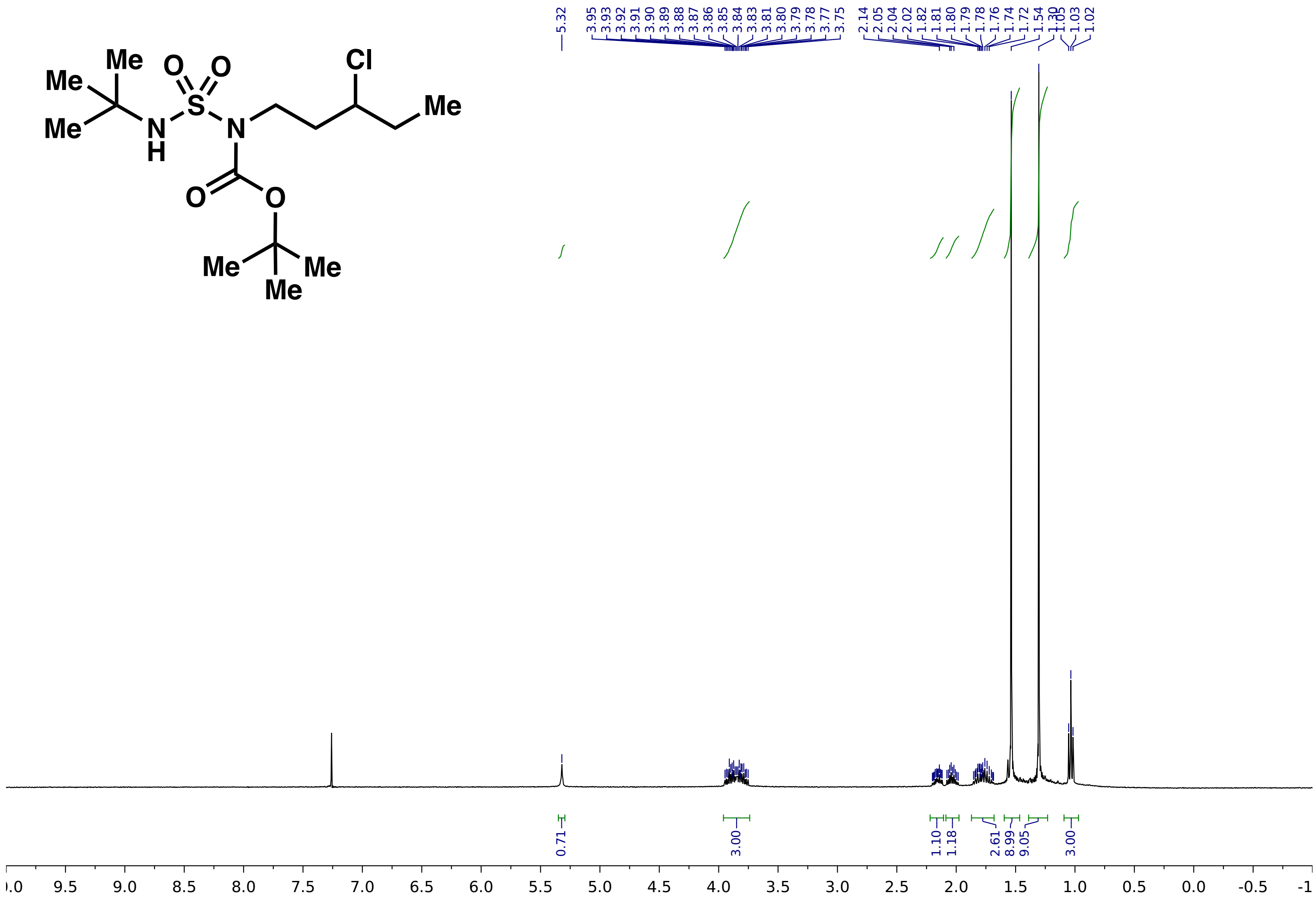

^{1}H NMR (400 MHz, $CDCl_3$) for *N*-*tert*-butoxycarbonyl-*N'*-*tert*-butyl-*N*-(3-chloropent-1-yl)sulfamide (**4i**)

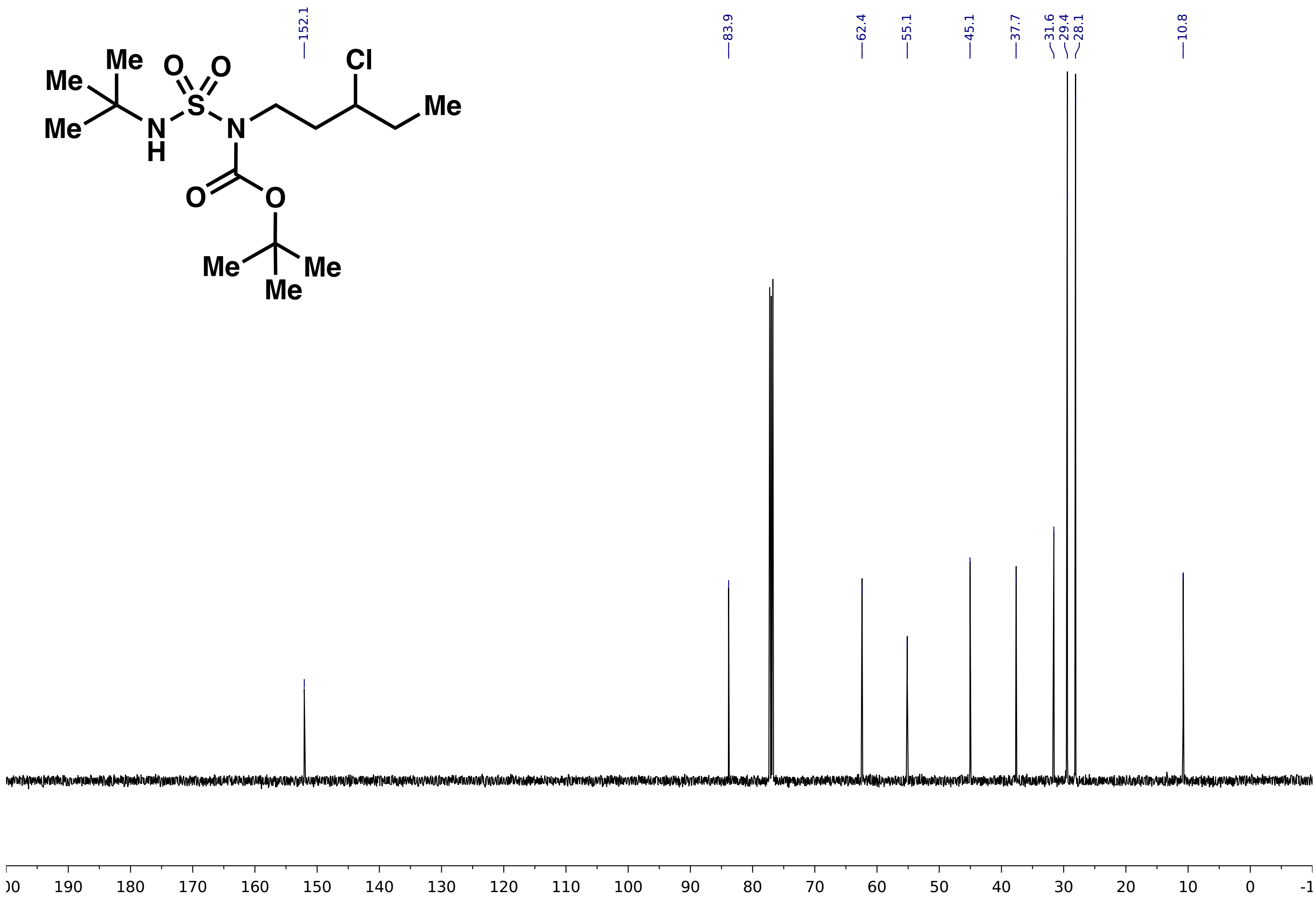

^{13}C NMR (126 MHz, $CDCl_3$) for *N*-*tert*-butoxycarbonyl-*N'*-*tert*-butyl-*N*-(3-chloropent-1-yl)sulfamide (**4i**)

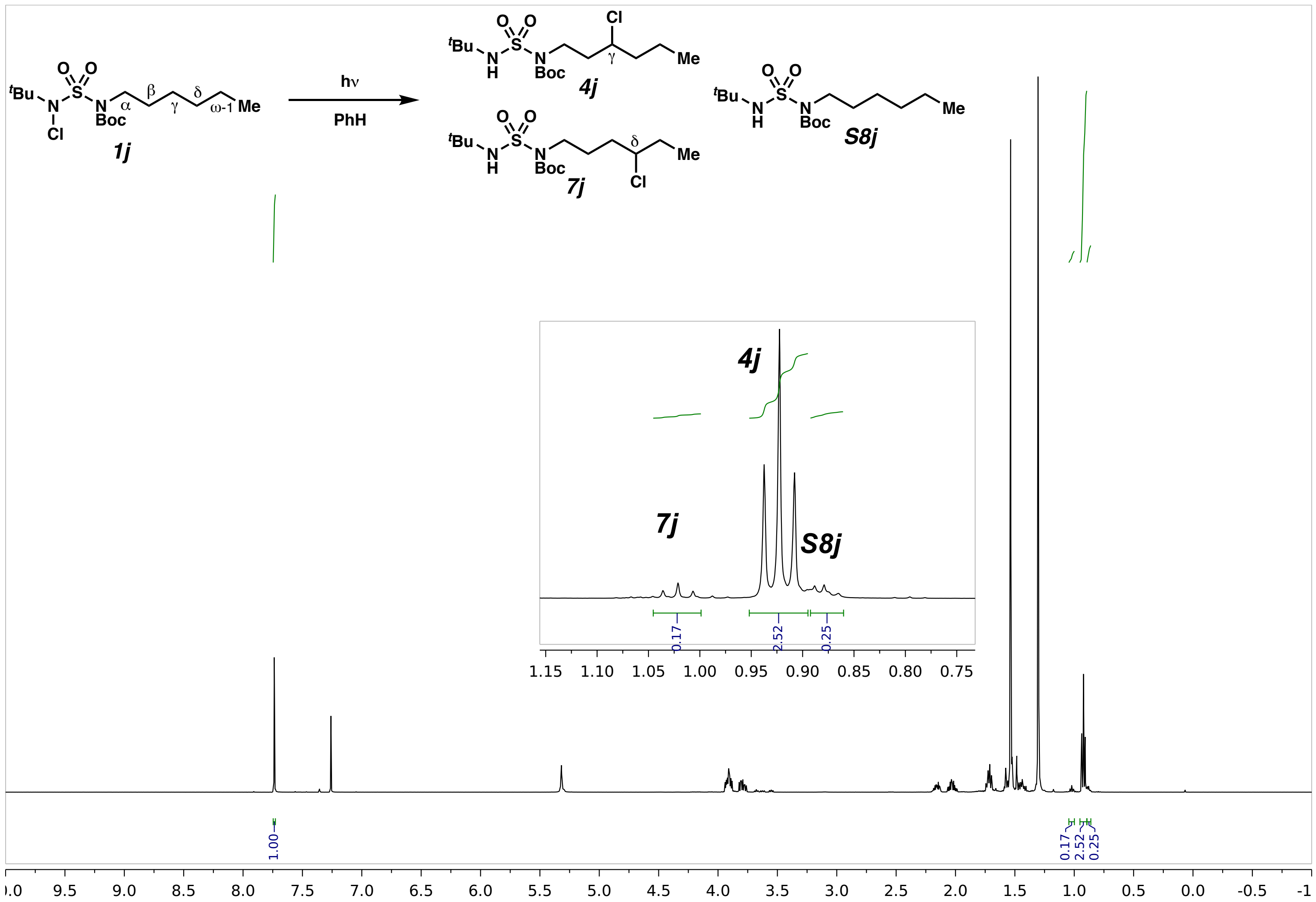

Crude ^{1}H NMR (500 MHz, $CDCl_3$, 25s relaxation delay) for chlorine-transfer reaction of *N*-chlorosulfamide **1j**

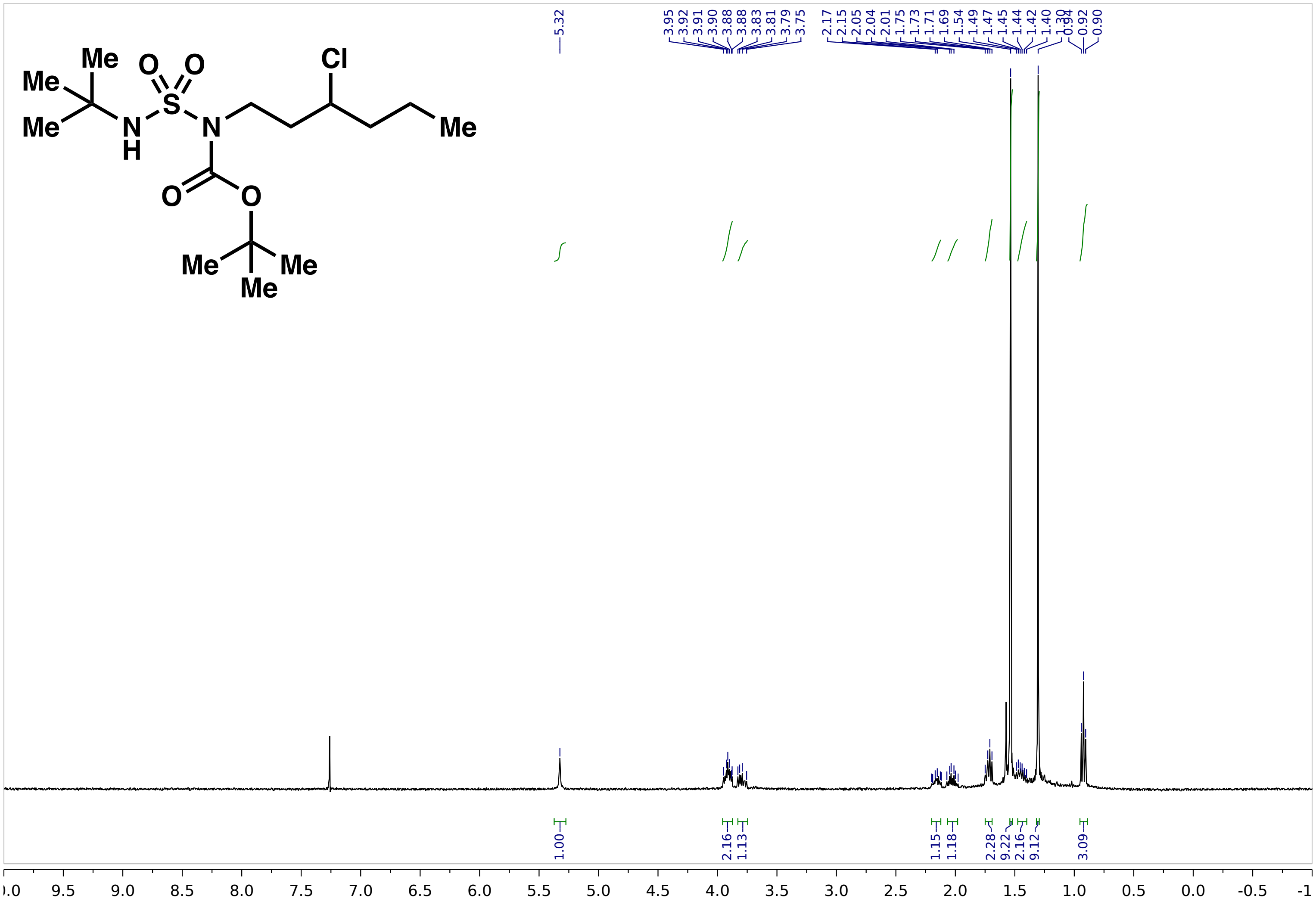

^{1}H NMR (400 MHz, $CDCl_3$) for *N*-*tert*-butoxycarbonyl-*N'*-*tert*-butyl-*N*-(3-chlorohexyl)sulfamide (**4j**)

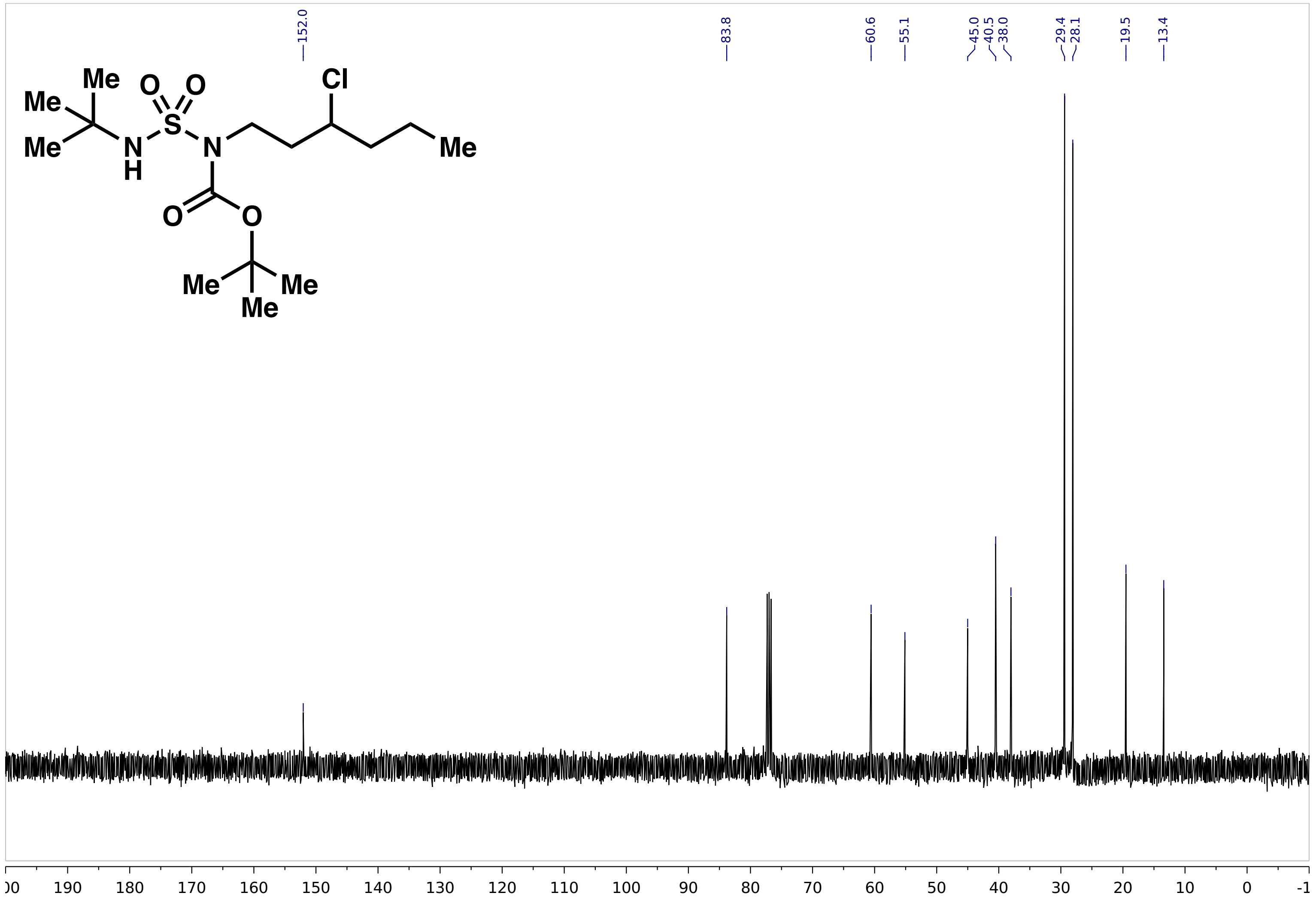

^{13}C NMR (101 MHz, $CDCl_3$) for *N*-*tert*-butoxycarbonyl-*N'*-*tert*-butyl-*N*-(3-chlorohexyl)sulfamide (**4j**)

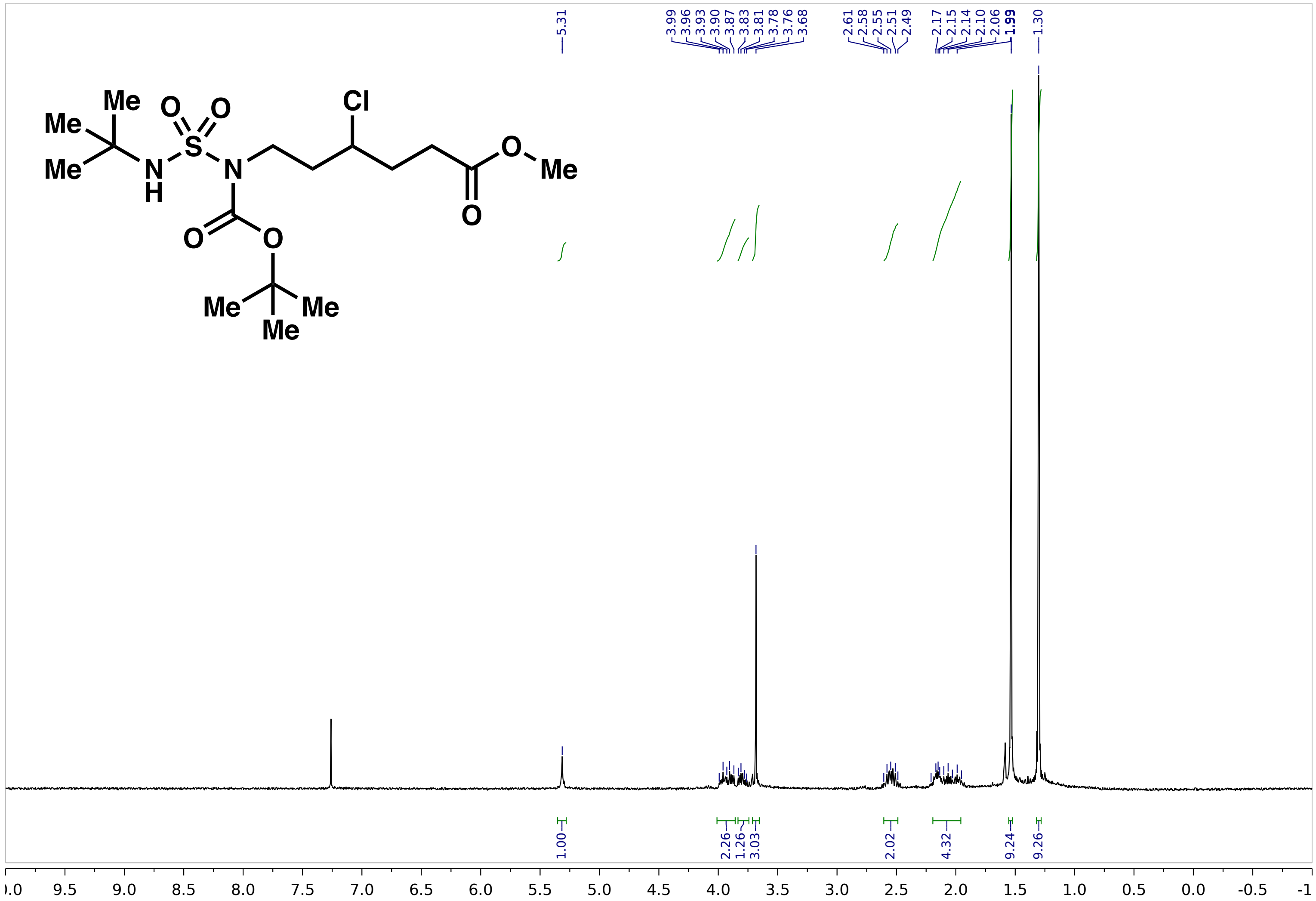

^{1}H NMR (400 MHz, $CDCl_3$) for *N*-*tert*-butyoxycarbonyl-*N'*-*tert*-butyl-*N*-(methyl 4-chlorohexano-6-yl)sulfamide (**4k**)

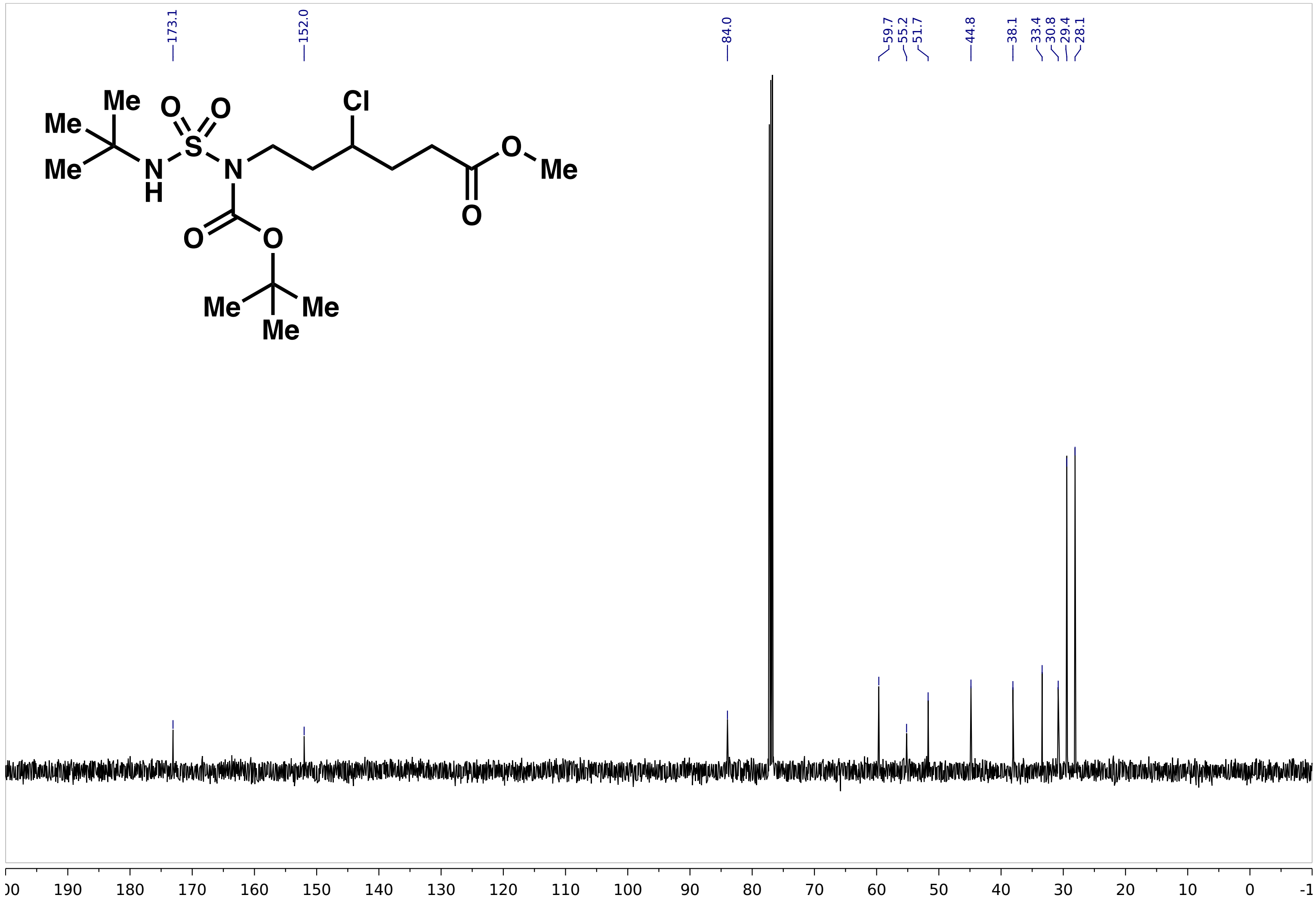

^{13}C NMR (126 MHz, $CDCl_3$) for *N*-*tert*-butyoxycarbonyl-*N'*-*tert*-butyl-*N*-(methyl 4-chlorohexano-6-yl)sulfamide (**4k**)

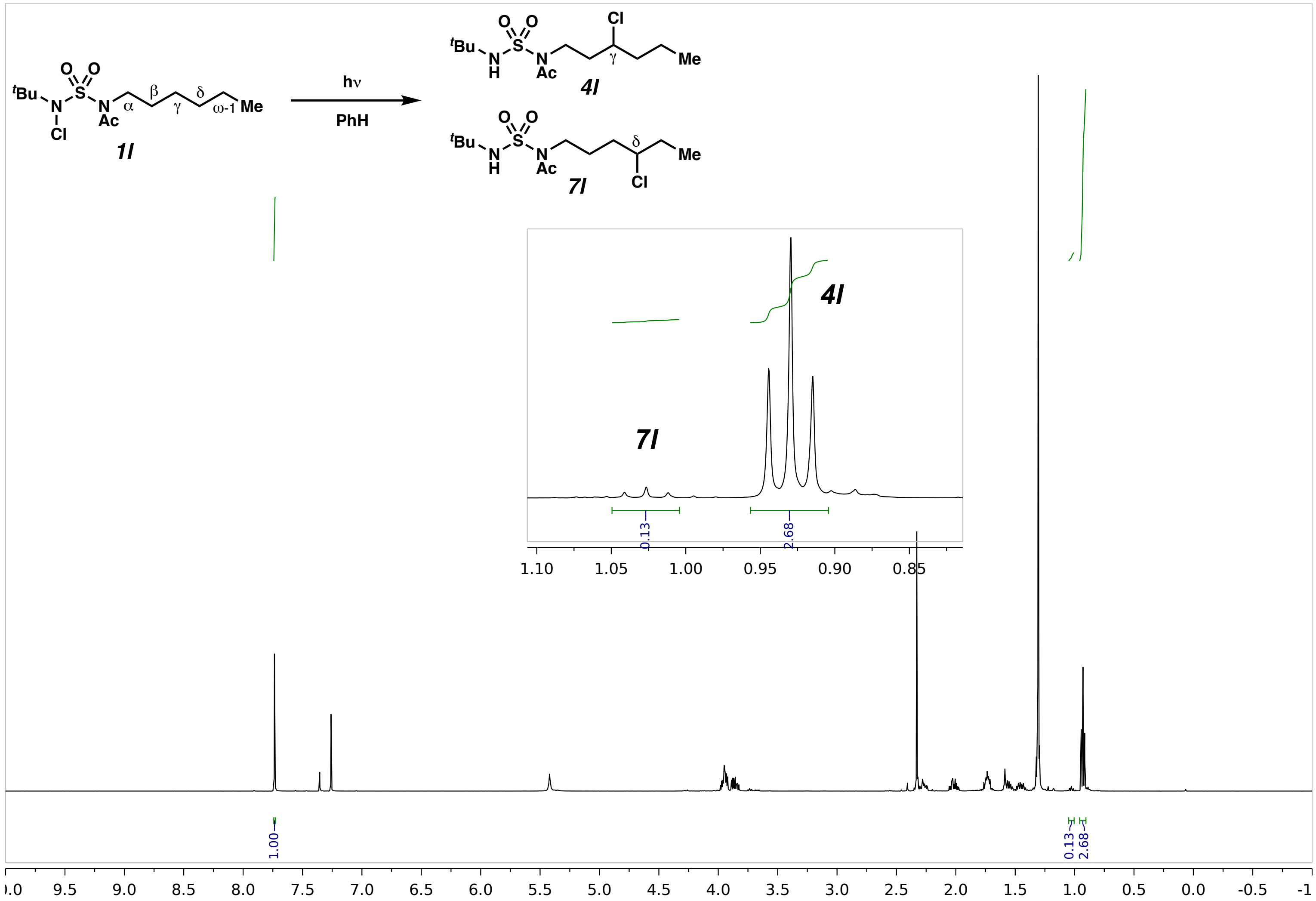

Crude ^{1}H NMR (500 MHz, $CDCl_3$, 25s relaxation delay) for chlorine-transfer reaction of *N*-chlorosulfamide **1l**

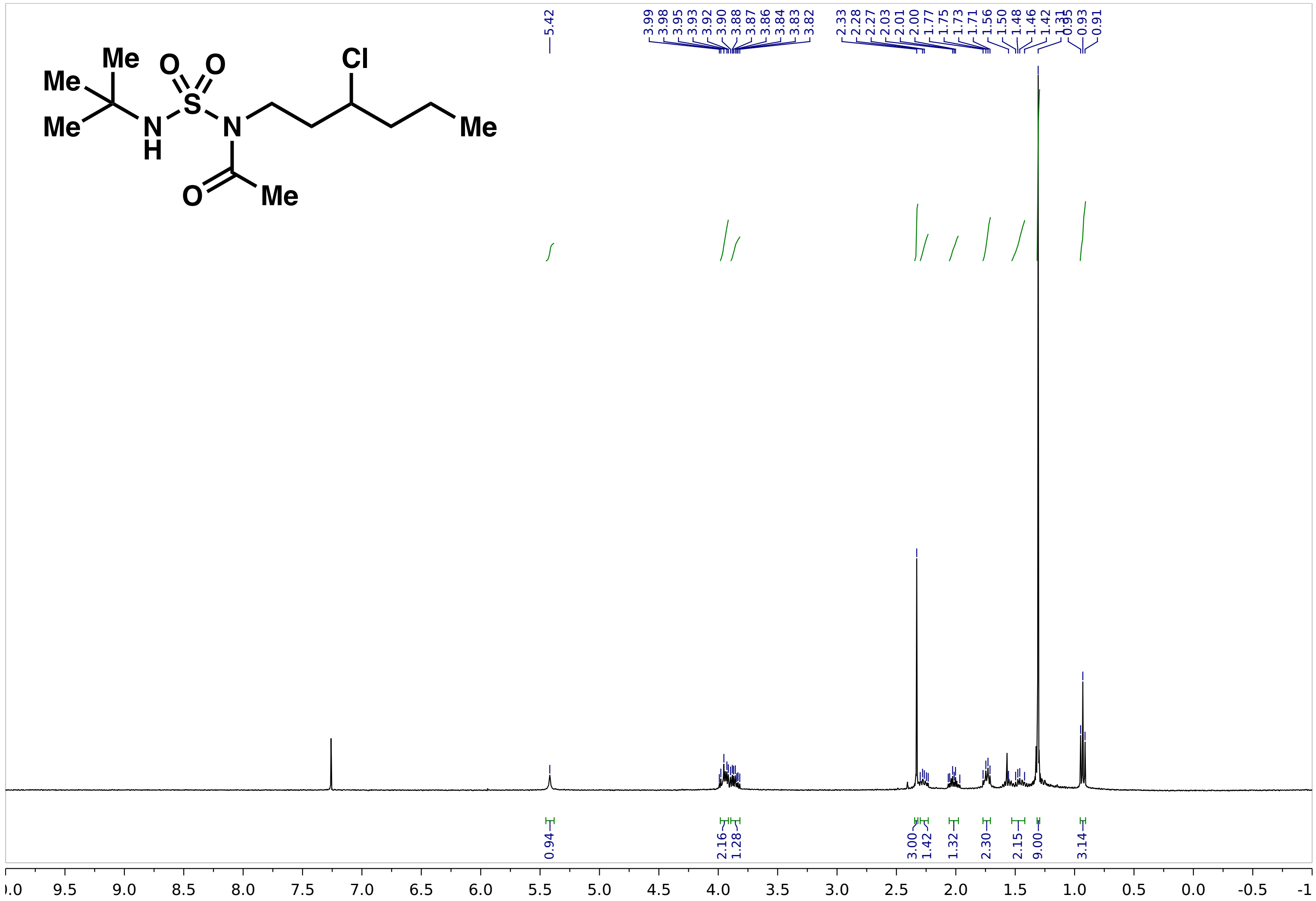

^{1}H NMR (400 MHz, $CDCl_3$) for *N*-*tert*-butoxycarbonyl-*N'*-*tert*-butyl-*N*-(3-chlorohex-1-yl)sulfamide (**4l**)

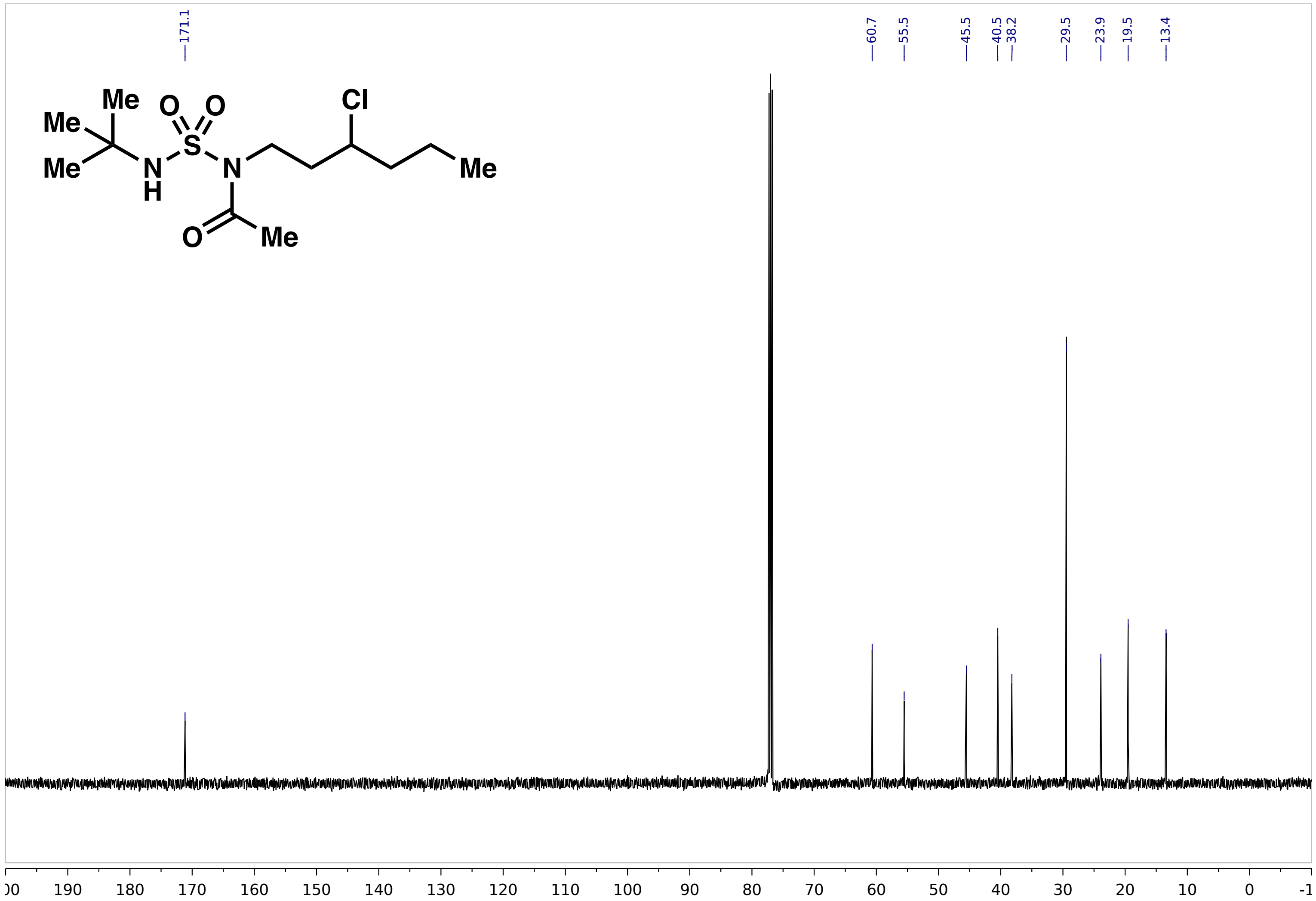

^{13}C NMR (126 MHz, $CDCl_3$) for *N*-*tert*-butoxycarbonyl-*N'*-*tert*-butyl-*N*-(3-chlorohex-1-yl)sulfamide (**4l**)

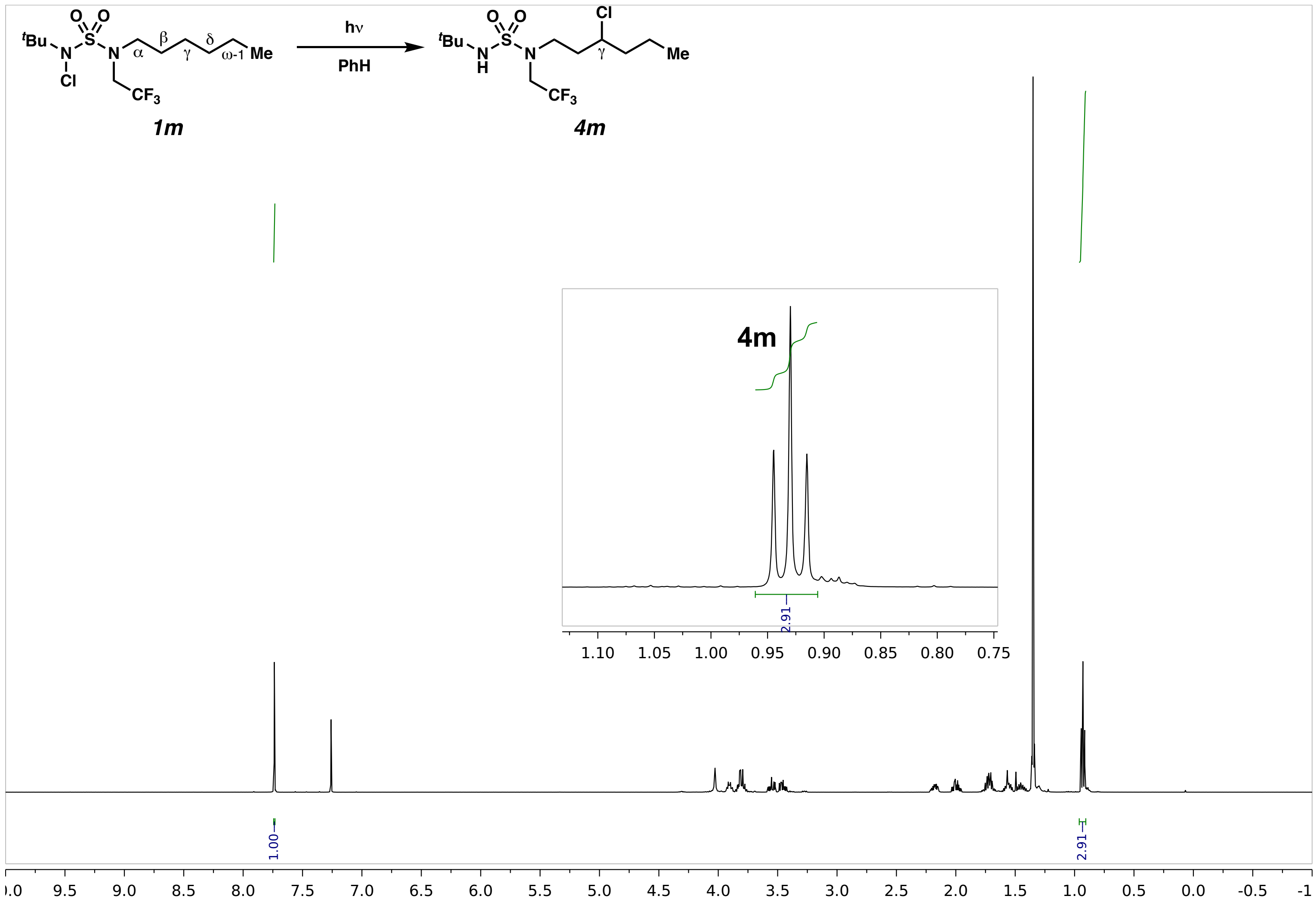

Crude ^{1}H NMR (500 MHz, $CDCl_3$, 25s relaxation delay) for chlorine-transfer reaction of *N*-chlorosulfamide **1m**

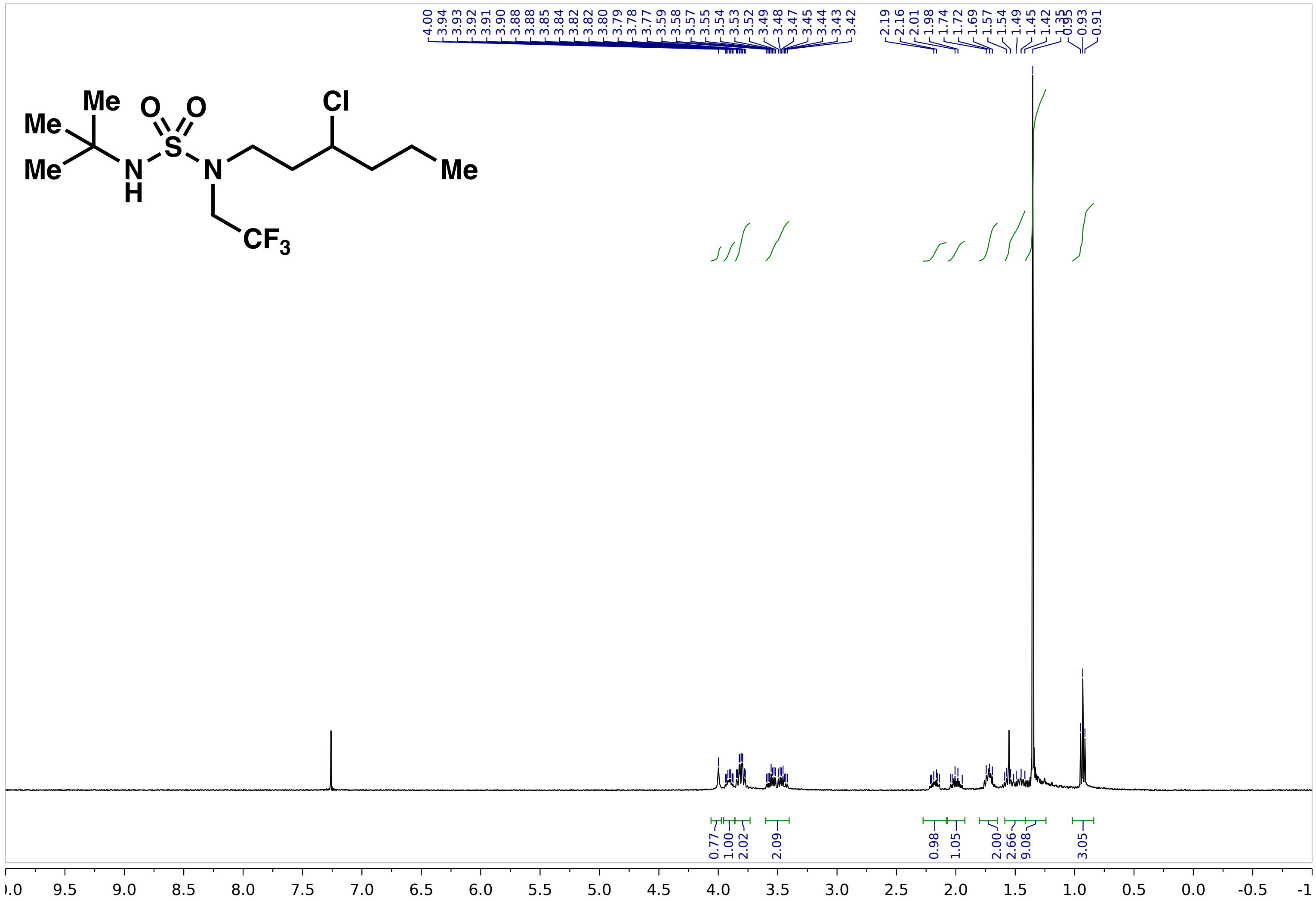

^{1}H NMR (400 MHz, $CDCl_3$) for *N*-*tert*-butyl-*N'*-(3-chlorohex-1-yl)-*N'*-(2,2,2-trifluoroethyl)sulfamide (**4m**)

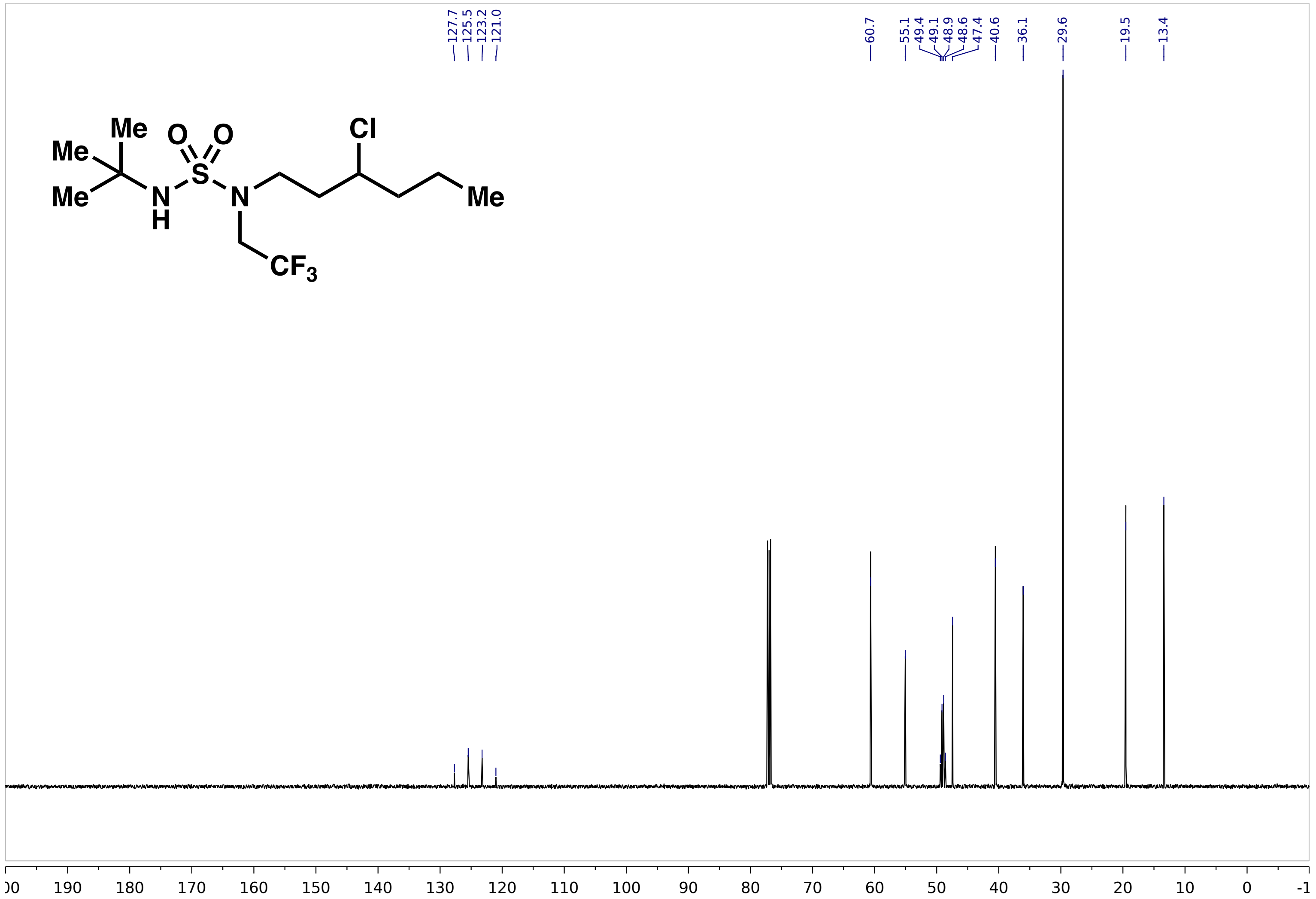

^{13}C NMR (126 MHz, $CDCl_3$) for *N*-*tert*-butyl-*N'*-(3-chlorohex-1-yl)-*N'*-(2,2,2-trifluoroethyl)sulfamide (**4m**)

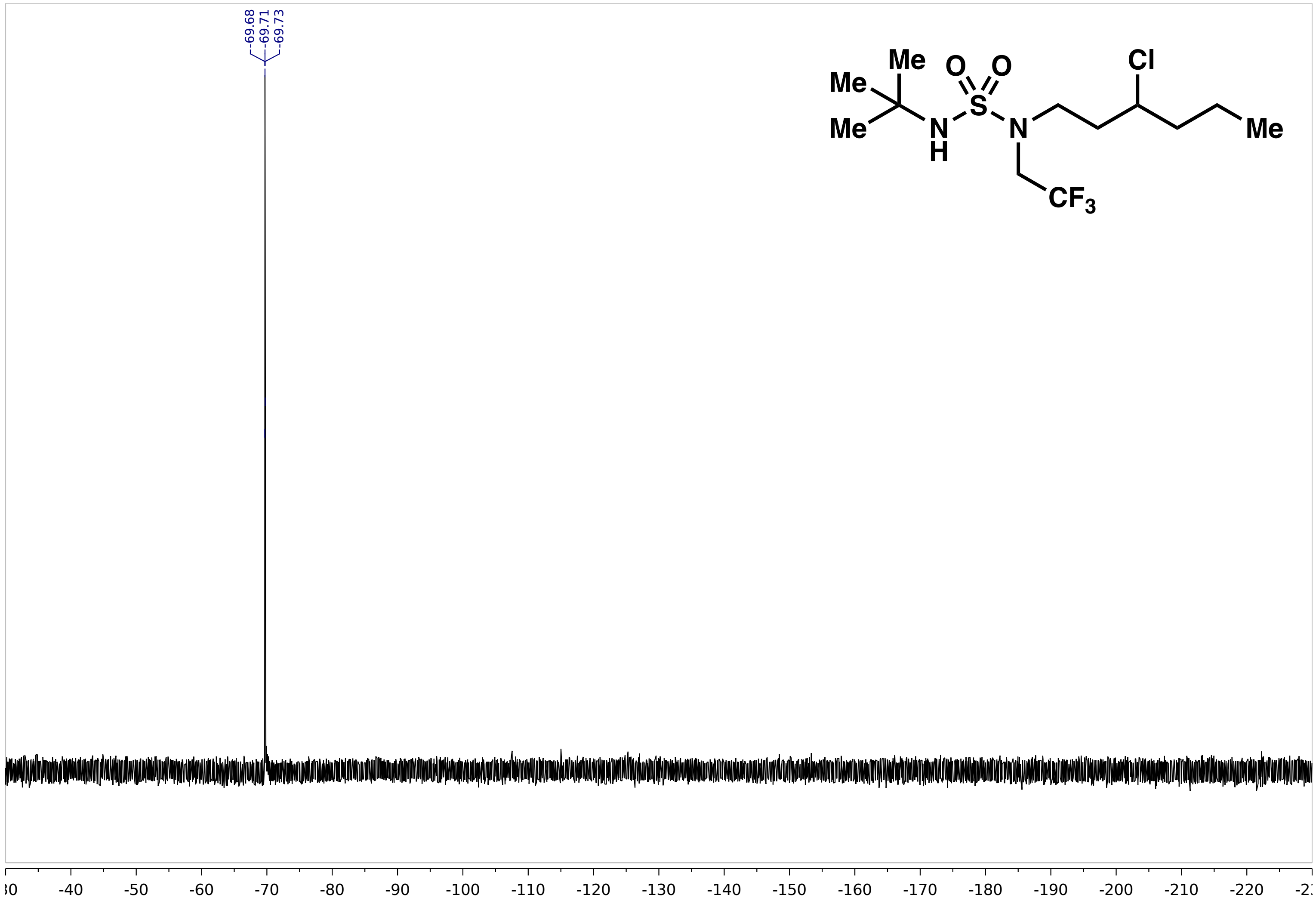

^{19}F NMR (376 MHz, $CDCl_3$) for *N*-*tert*-butyl-*N'*-(3-chlorohex-1-yl)-*N*'-(2,2,2-trifluoroethyl)sulfamide (**4m**)

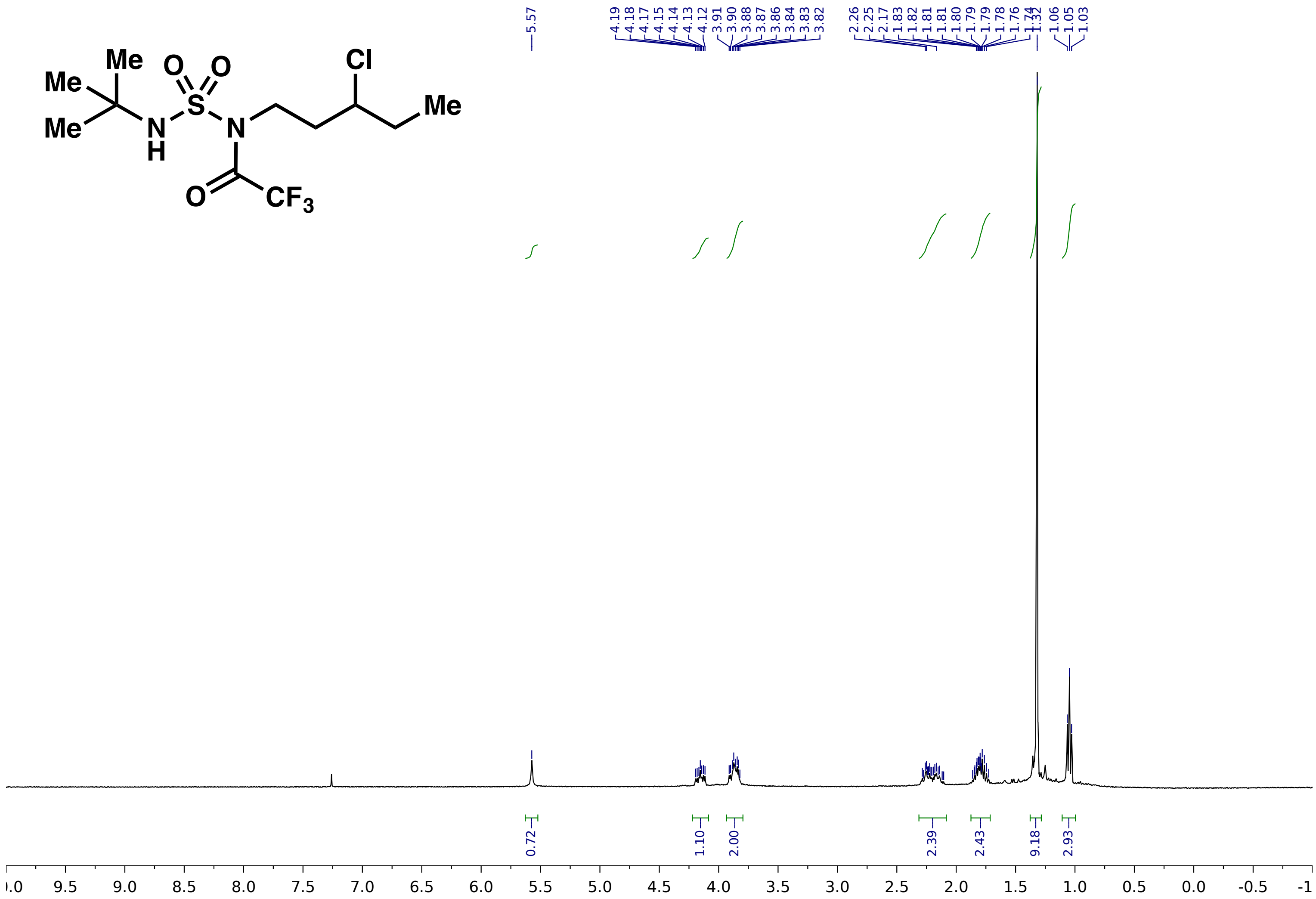

^{1}H NMR (400 MHz, $CDCl_3$) for *N'*-*tert*-butyl-*N*-(3-chloropent-1-yl)-*N*-trifluoroacetylsulfamide (**4n**)

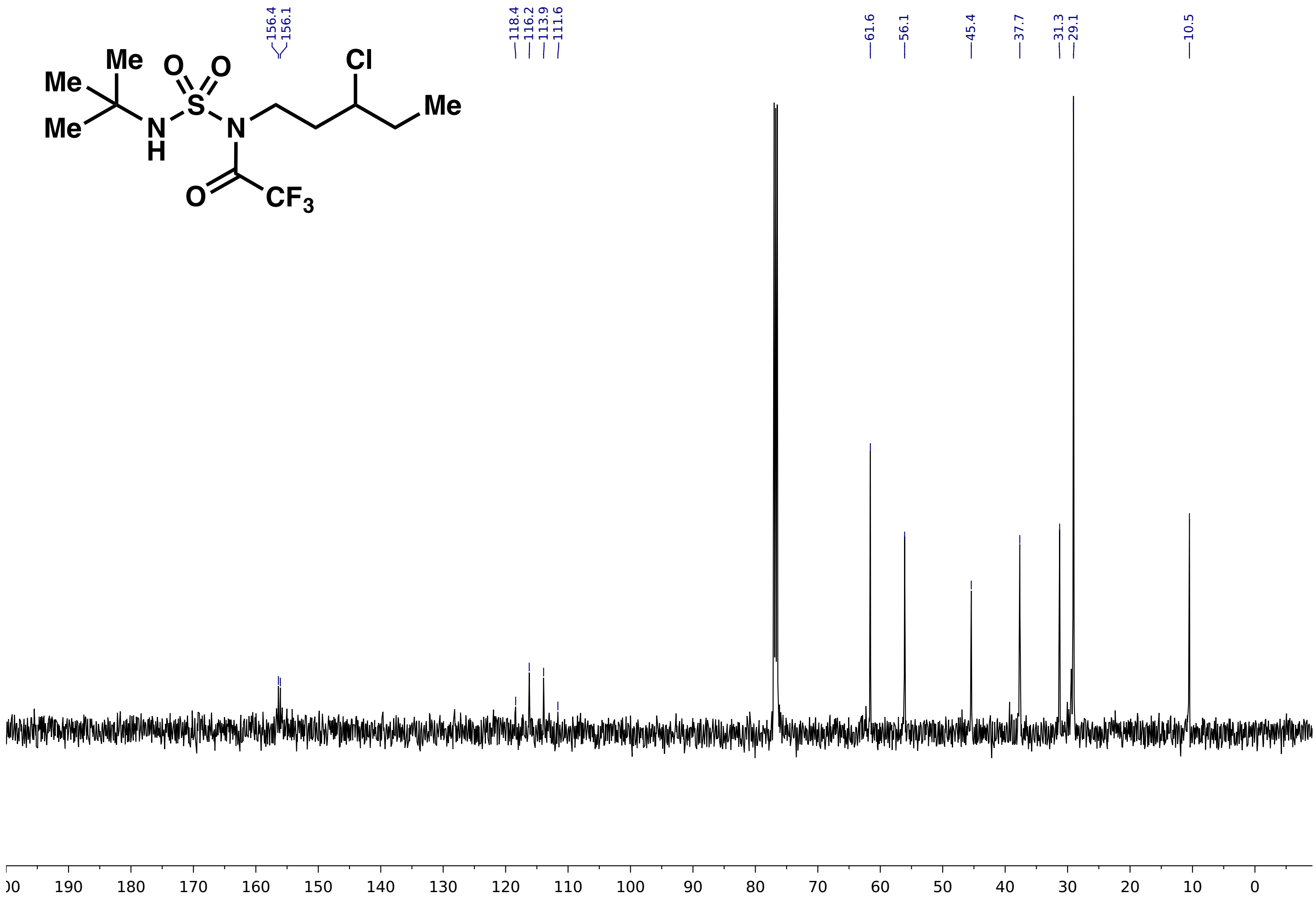

^{13}C NMR (126 MHz, $CDCl_3$) for *N'*-*tert*-butyl-*N*-(3-chloropent-1-yl)-*N*-trifluoroacetylsulfamide (**4n**)

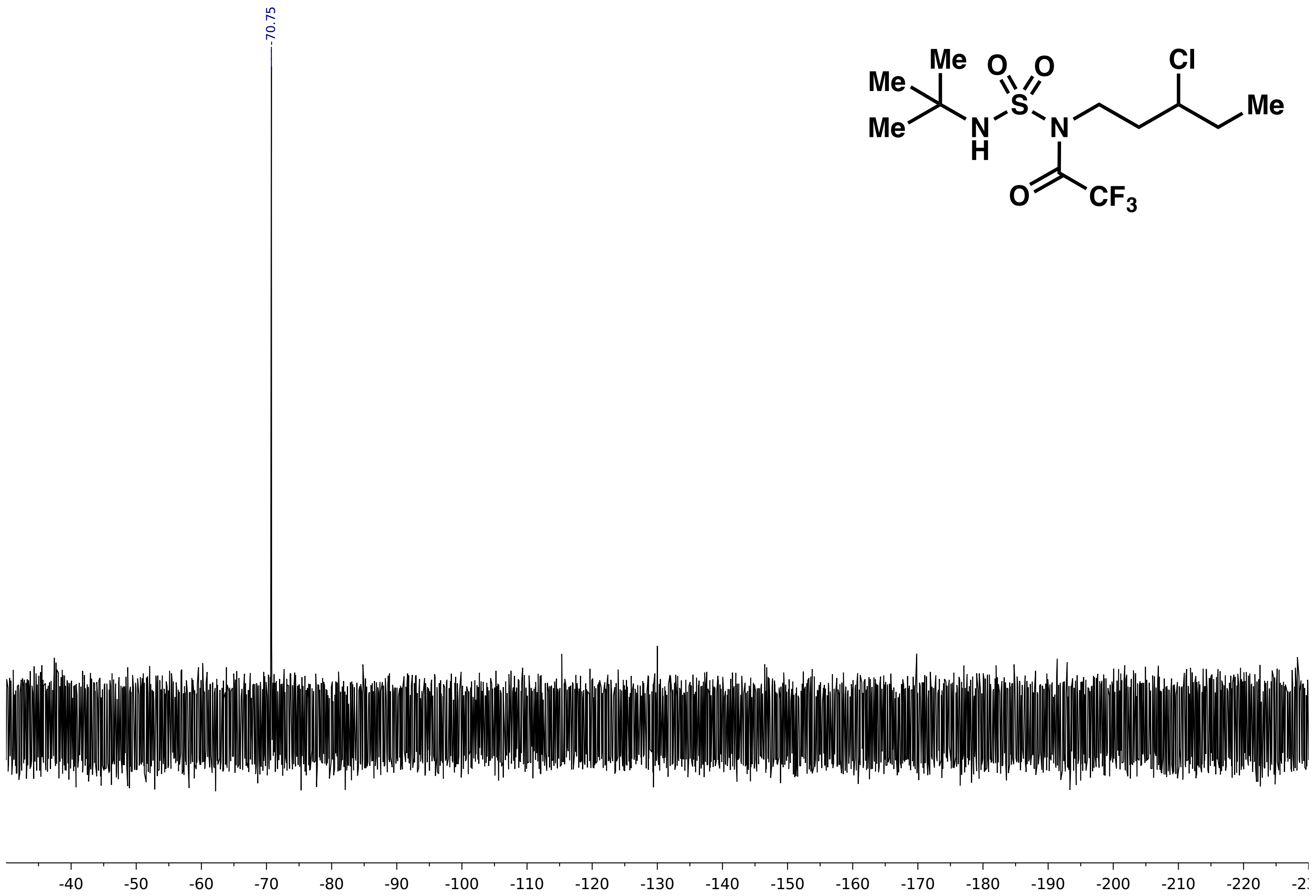

^{19}F NMR (376 MHz, $CDCl_3$) for *N'*-*tert*-butyl-*N*-(3-chloropent-1-yl)-*N*-trifluoroacetylsulfamide (**4n**)

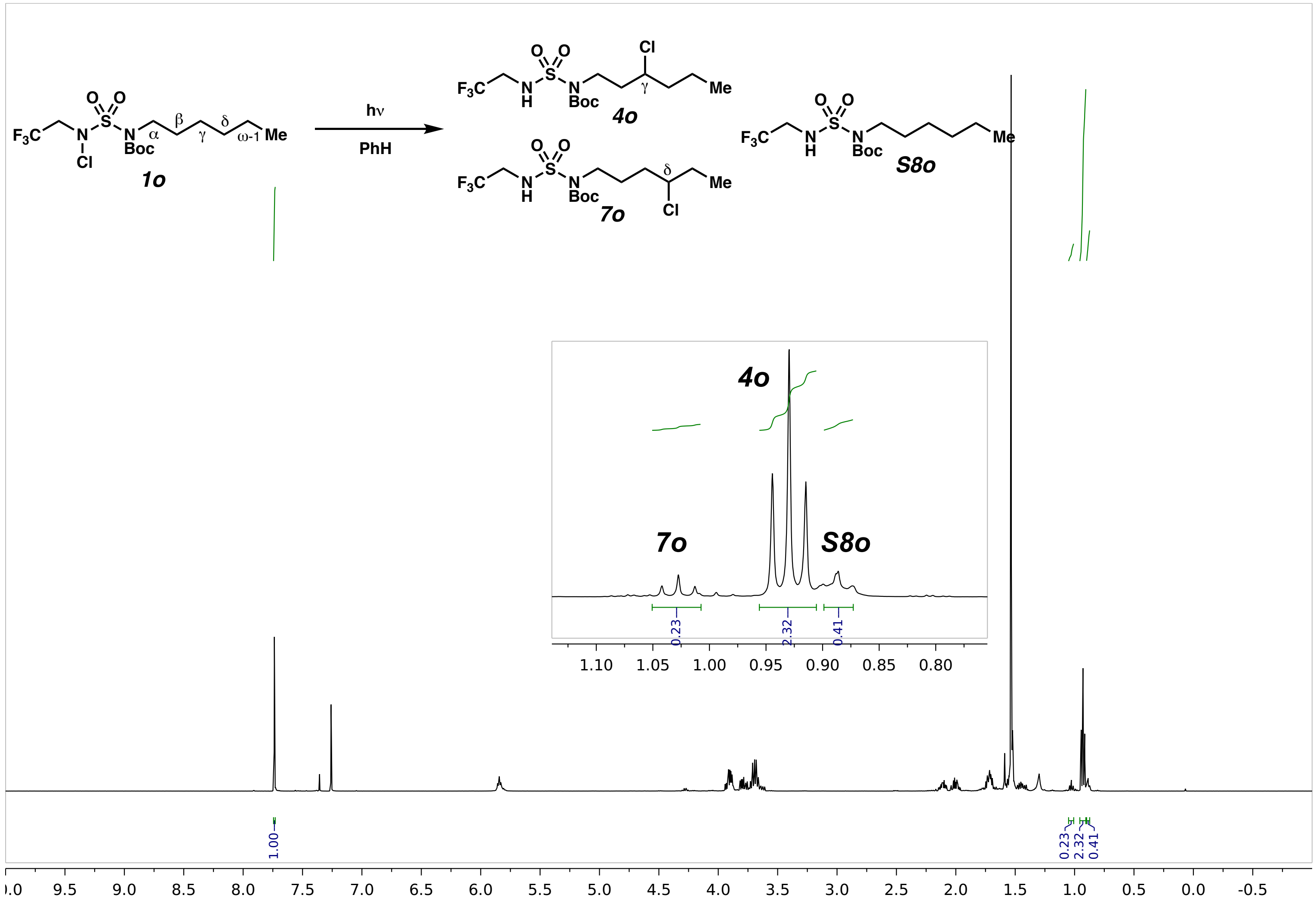

Crude ^{1}H NMR (500 MHz, $CDCl_3$, 25s relaxation delay) for chlorine-transfer reaction of *N*-chlorosulfamide **1o**

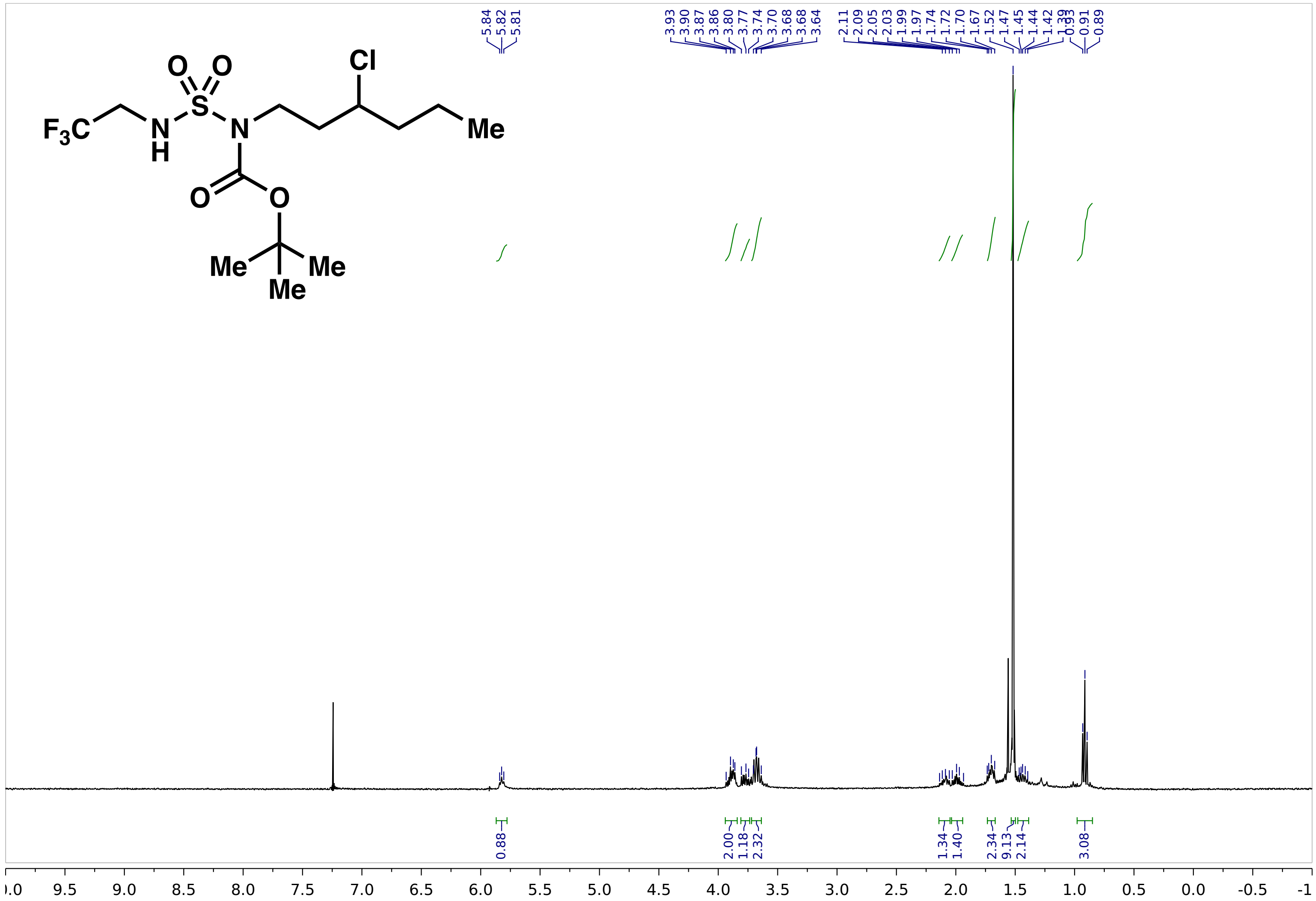

^{1}H NMR (400 MHz, $CDCl_3$) for *N*-*tert*-butoxycarbonyl-*N*-(3-chlorohex-1-yl)-*N'*-(2,2,2-trifluoroethyl)sulfamide (**4o**)

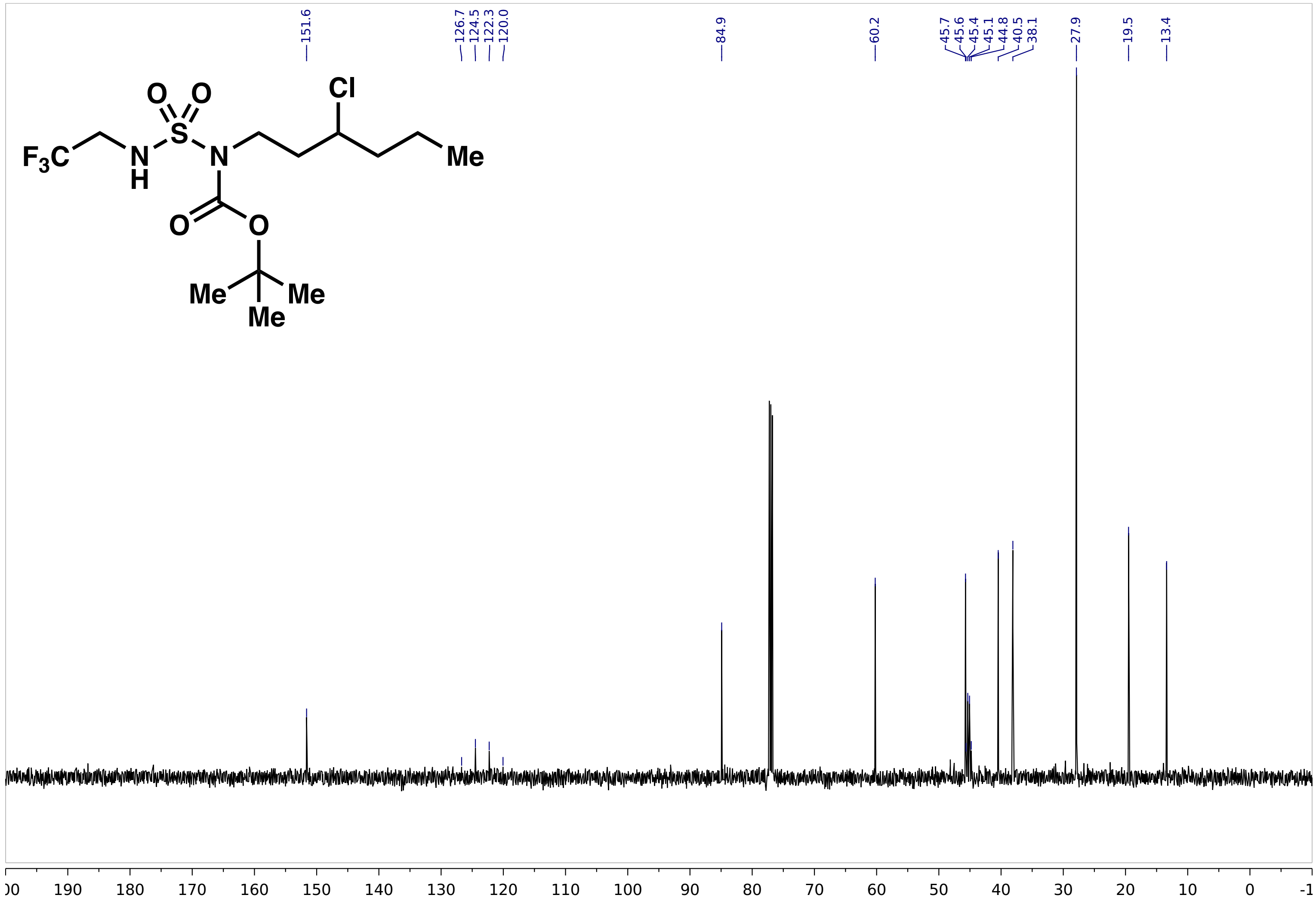

^{13}C NMR (126 MHz, $CDCl_3$) for *N*-*tert*-butoxycarbonyl-*N*-(3-chlorohex-1-yl)-*N'*-(2,2,2-trifluoroethyl)sulfamide (**4o**)

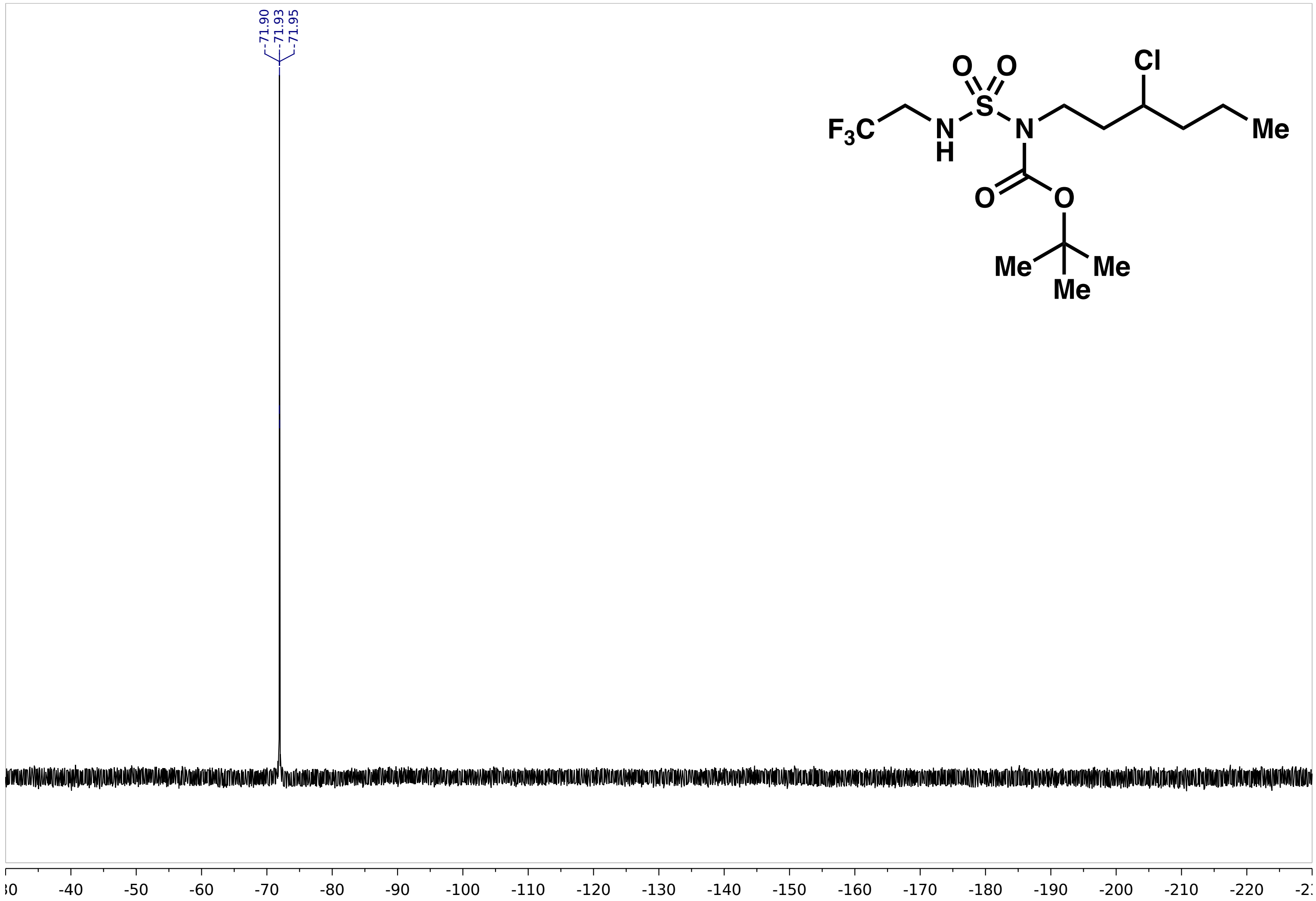

^{19}F NMR (376 MHz, $CDCl_3$) for *N*-*tert*-butoxycarbonyl-*N*-(3-chlorohex-1-yl)-*N'*-(2,2,2-trifluoroethyl)sulfamide (**4o**)

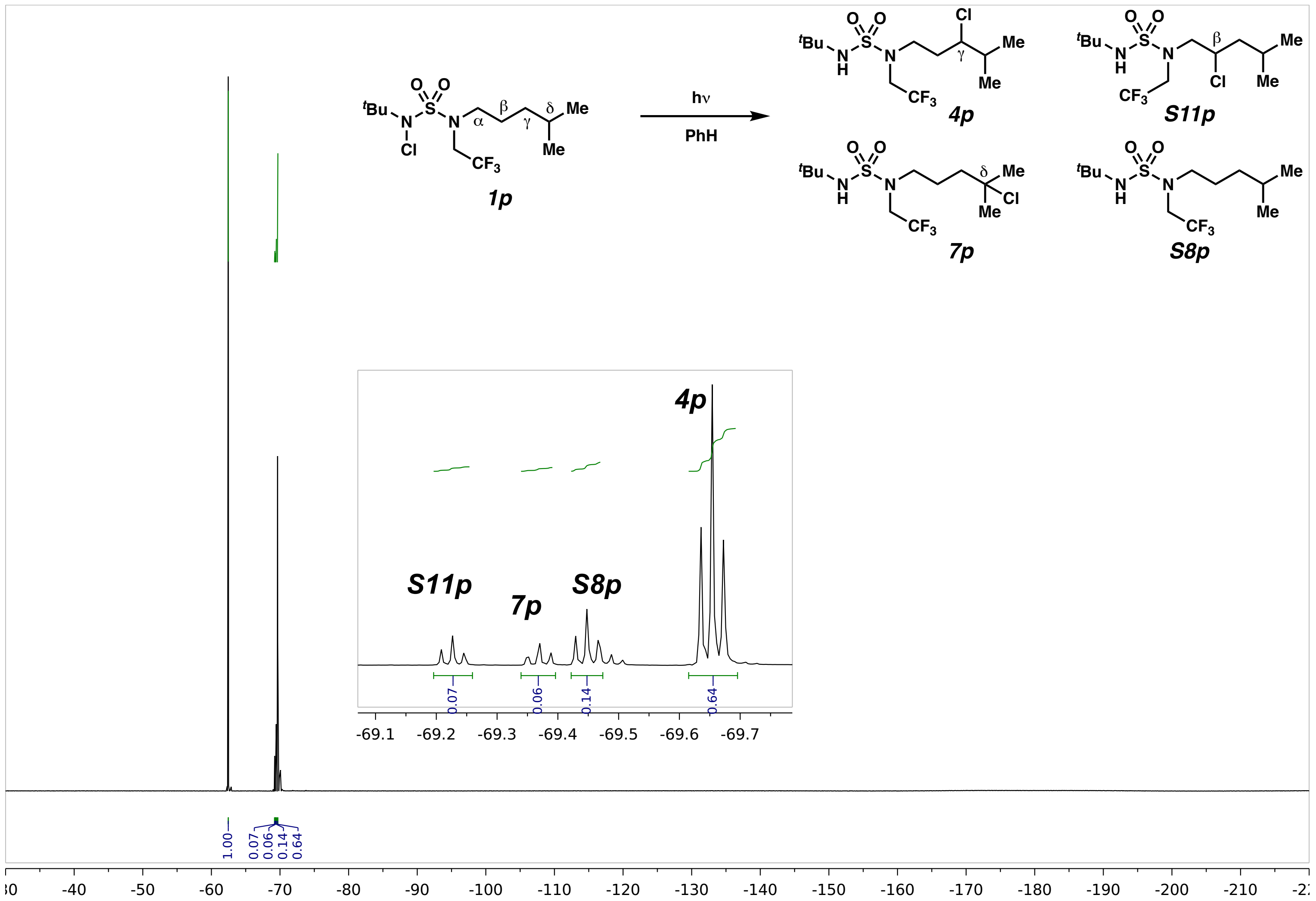

Crude ^{19}F NMR (471 MHz, $CDCl_3$, 25s relaxation delay) for chlorine-transfer reaction of *N*-chlorosulfamide **1p**

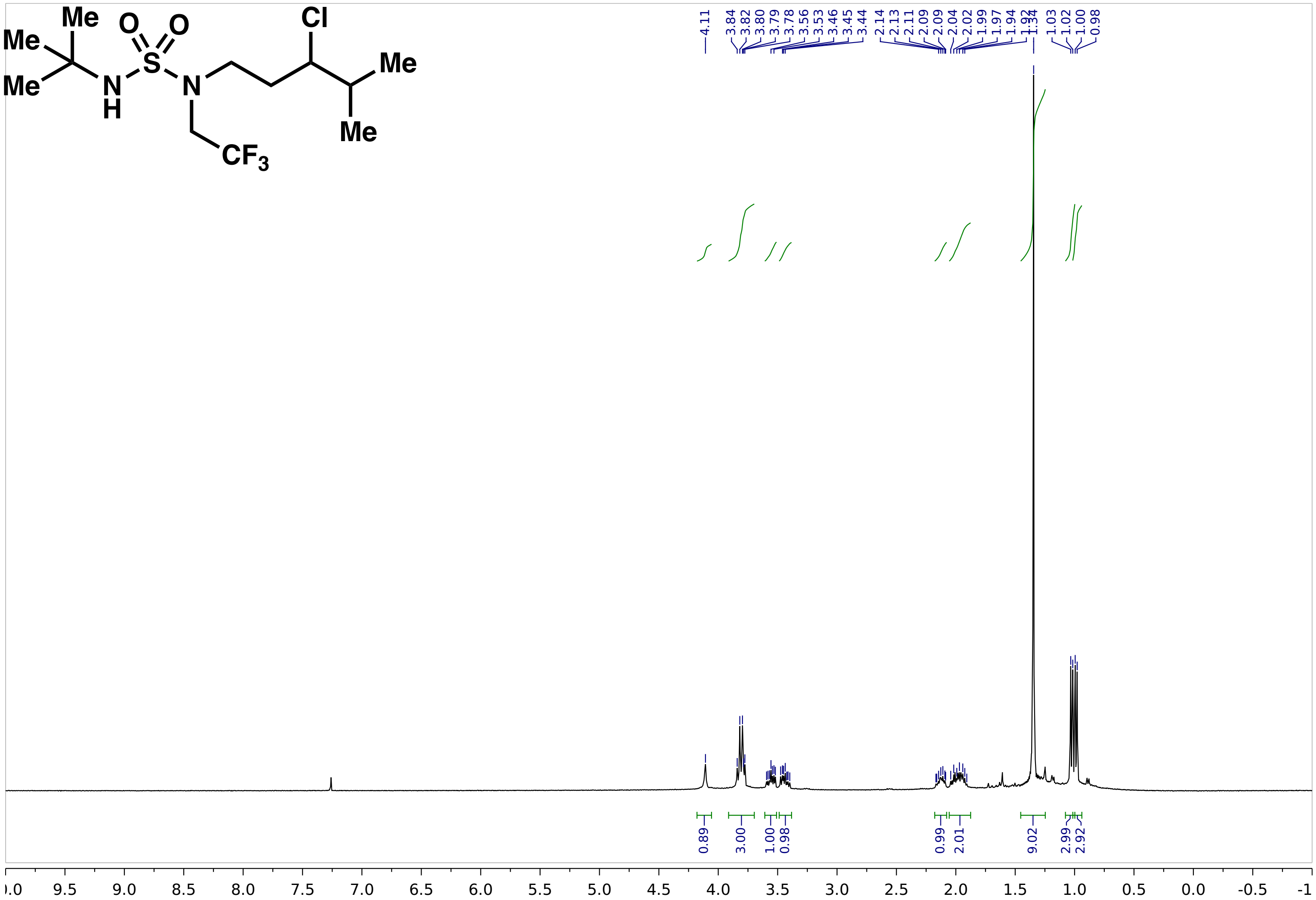

^{1}H NMR (400 MHz, $CDCl_3$) for *N*-*tert*-Butyl-*N'*-(3-chloro-4-methylpentyl)-*N*'-(2,2,2-trifluoroethyl)sulfamide (**4p**)

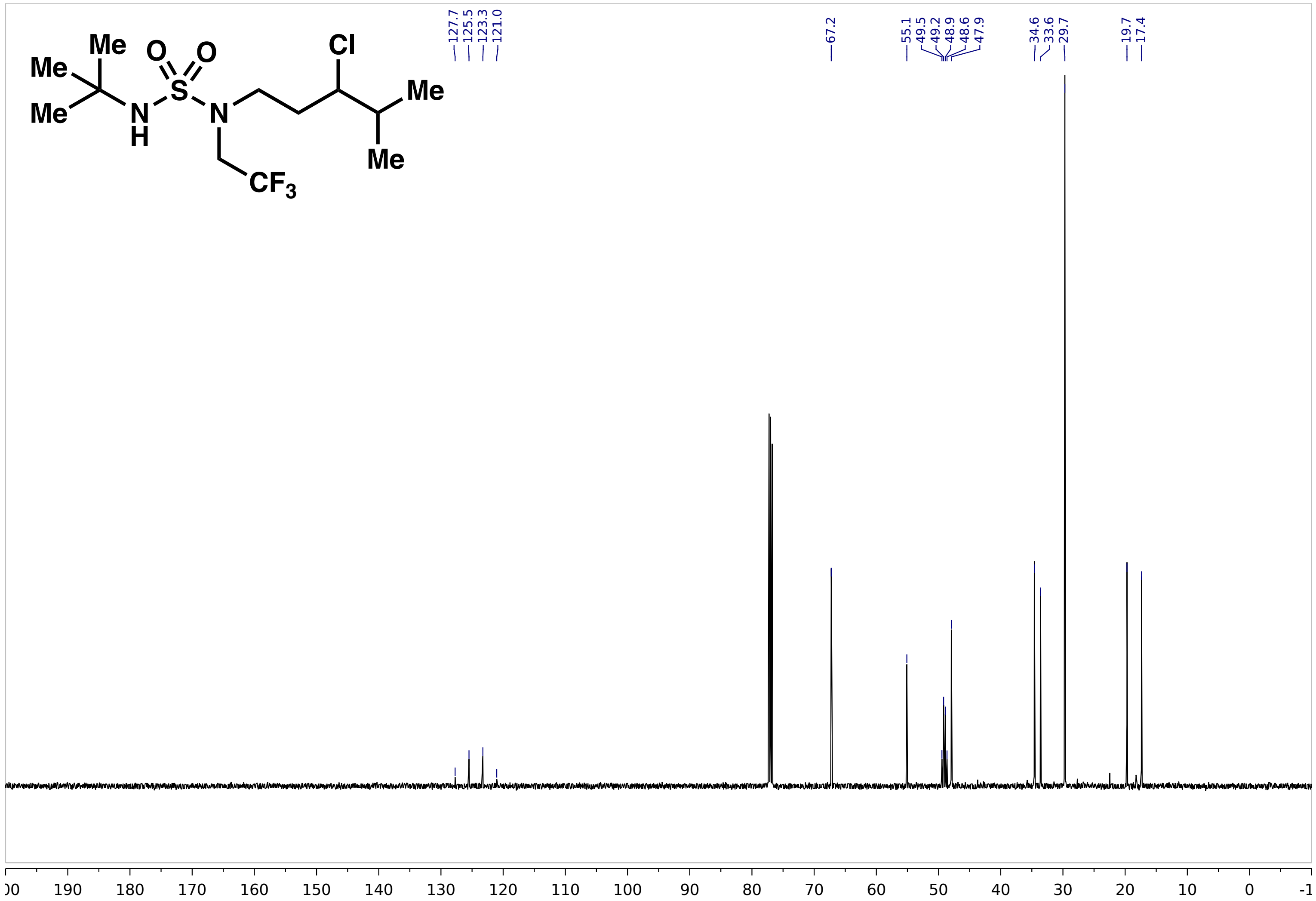

^{13}C NMR (126 MHz, CDCl$_3$) for *N*-*tert*-Butyl-*N'*-(3-chloro-4-methylpentyl)-*N*'-(2,2,2-trifluoroethyl)sulfamide (**4p**)

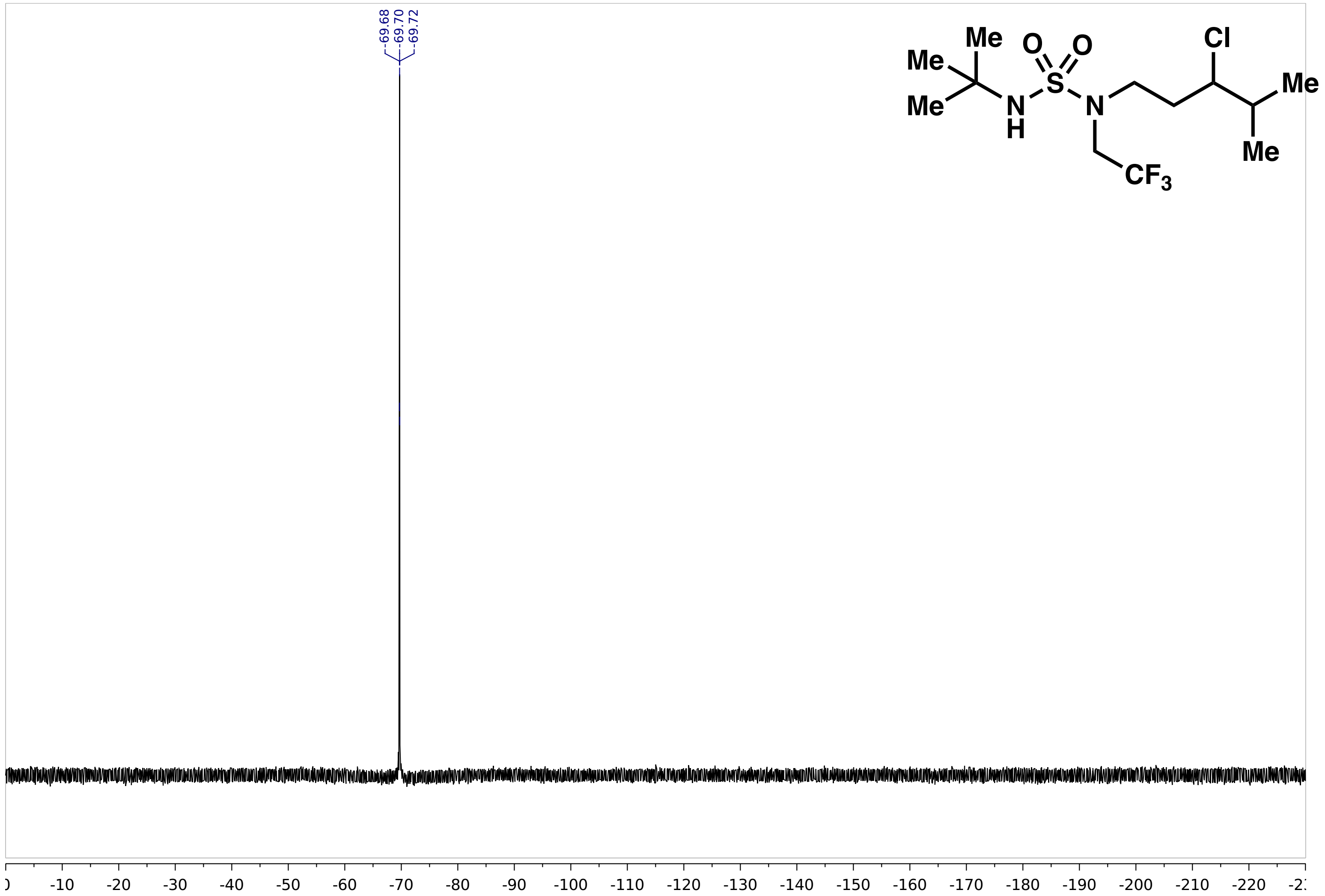

^{19}F NMR (376 MHz, $CDCl_3$) for *N*-*tert*-Butyl-*N'*-(3-chloro-4-methylpentyl)-*N*'-(2,2,2-trifluoroethyl)sulfamide (**4p**)

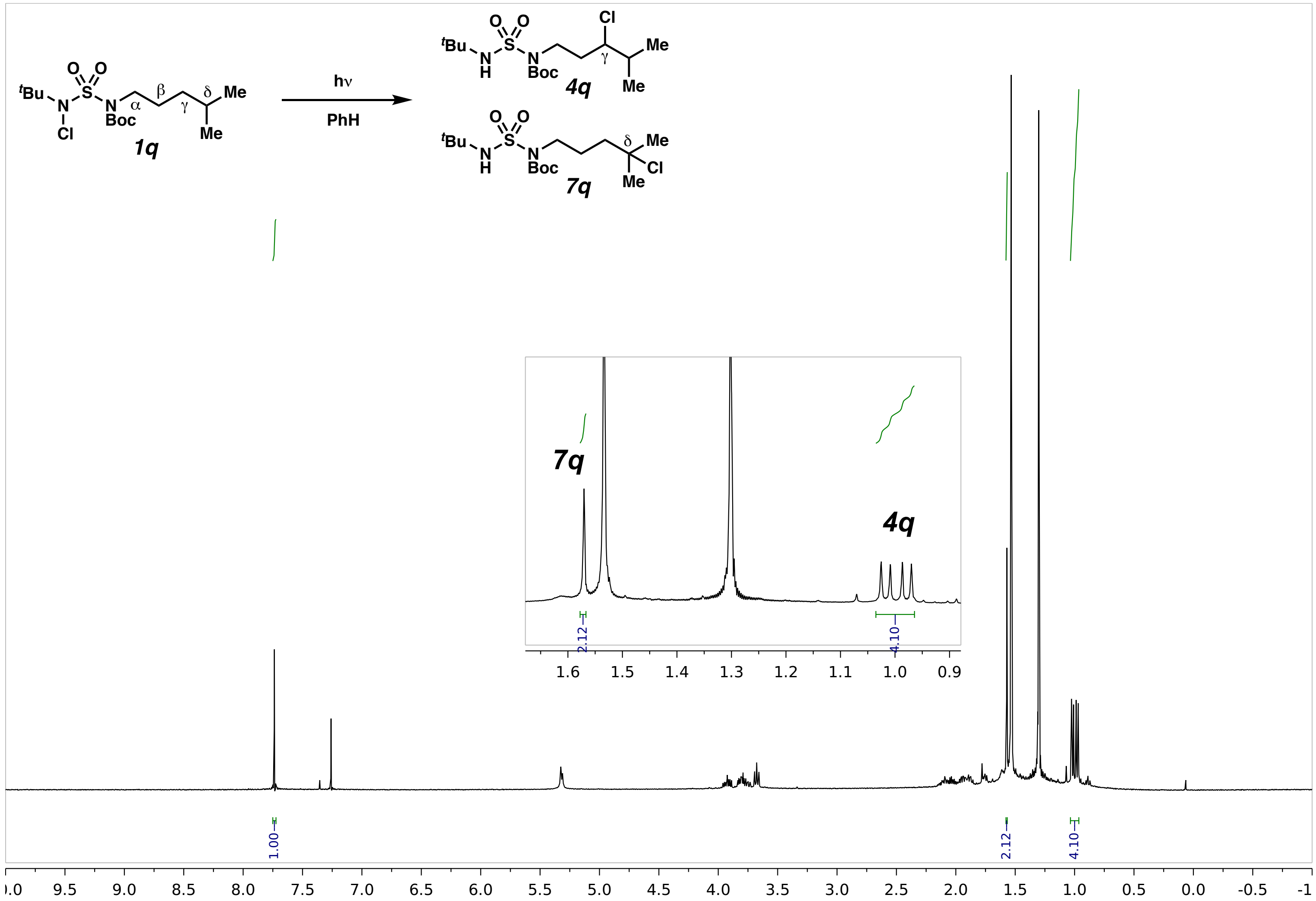

Crude ^{1}H NMR (500 MHz, $CDCl_3$, 25s relaxation delay) for chlorine-transfer reaction of *N*-chlorosulfamide **1q**

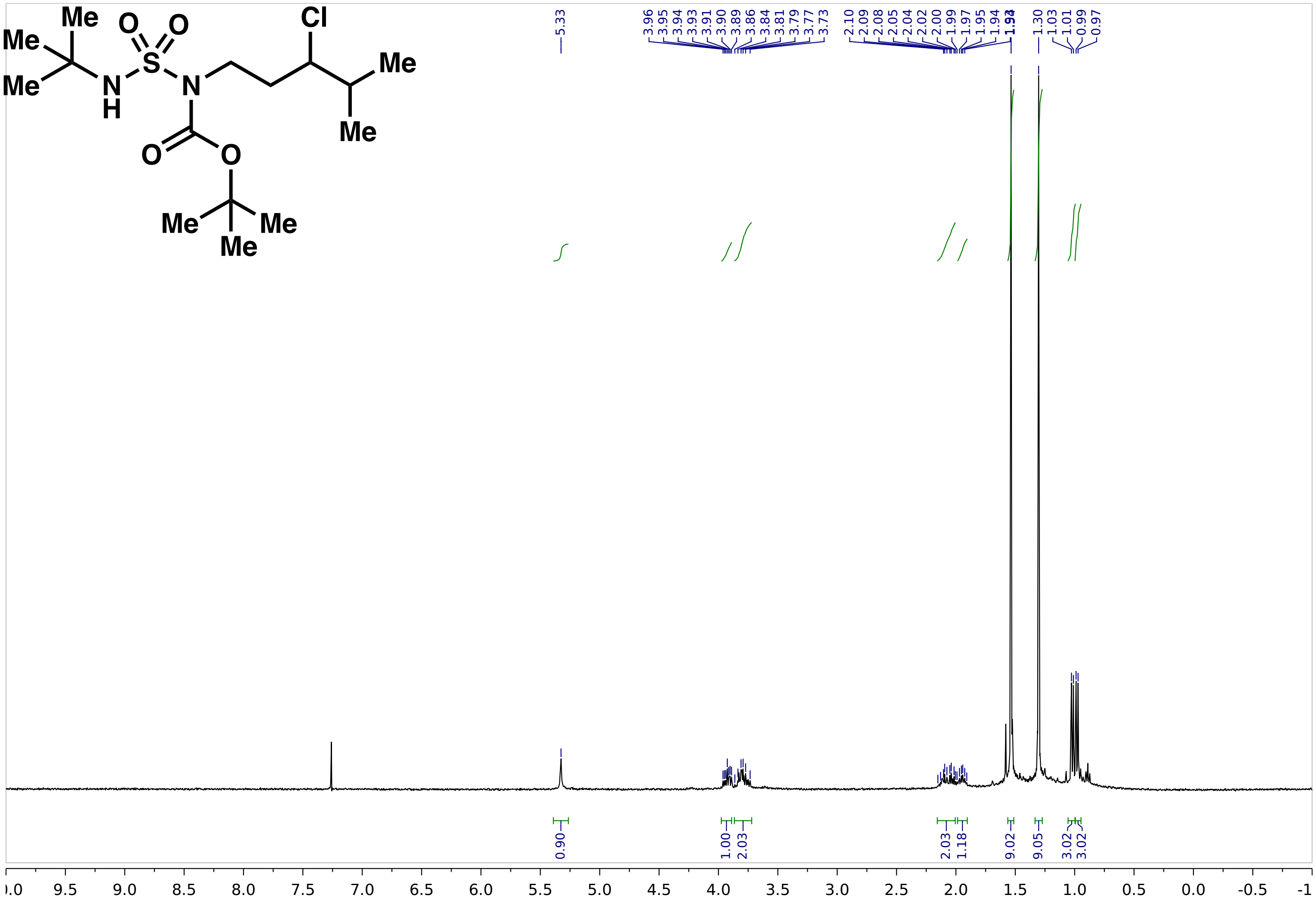

^{1}H NMR (400 MHz, $CDCl_3$) for *N*-*tert*-Butoxycarbonyl-*N'*-*tert*-butyl-*N*-(3-chloro-4-methylpentyl)sulfamide (**4q**)

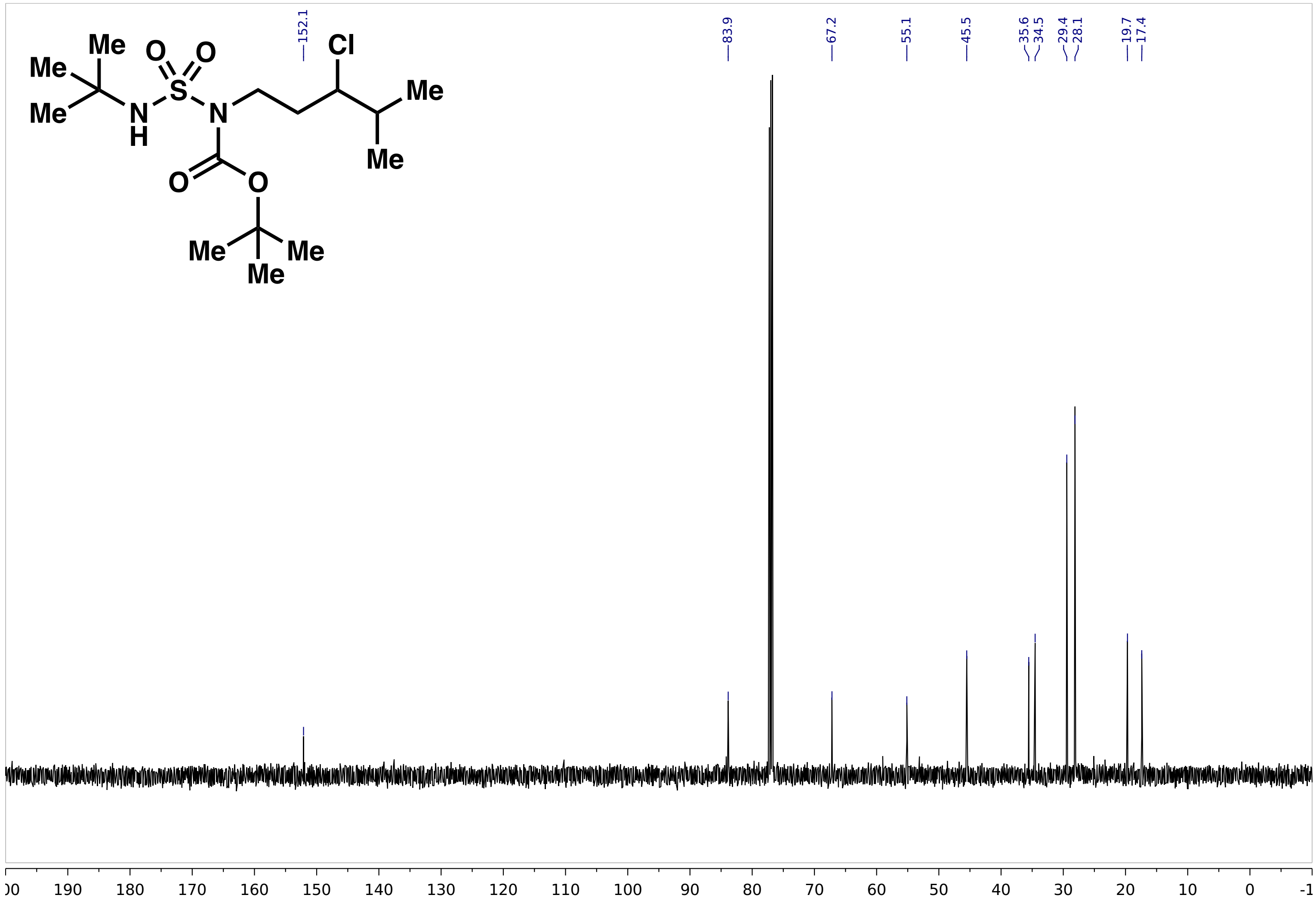

^{13}C NMR (126 MHz, $CDCl_3$) for *N*-*tert*-Butoxycarbonyl-*N'*-*tert*-butyl-*N*-(3-chloro-4-methylpentyl)sulfamide (**4q**)

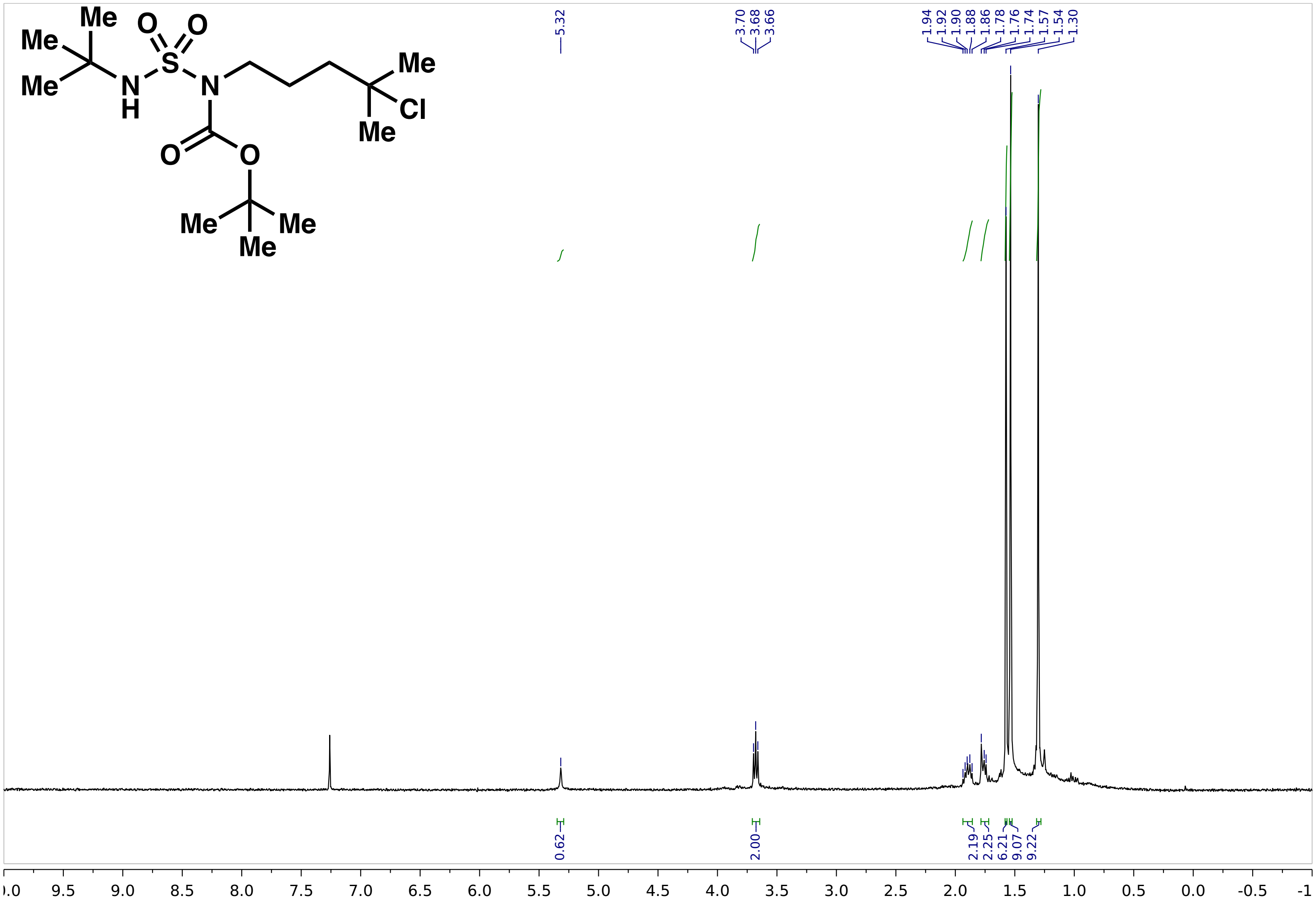

^{1}H NMR (400 MHz, $CDCl_3$) for *N-tert*-Butoxycarbonyl-*N'-tert*-butyl-*N*-(4-chloro-4-methylpentyl)sulfamide (**7q**)

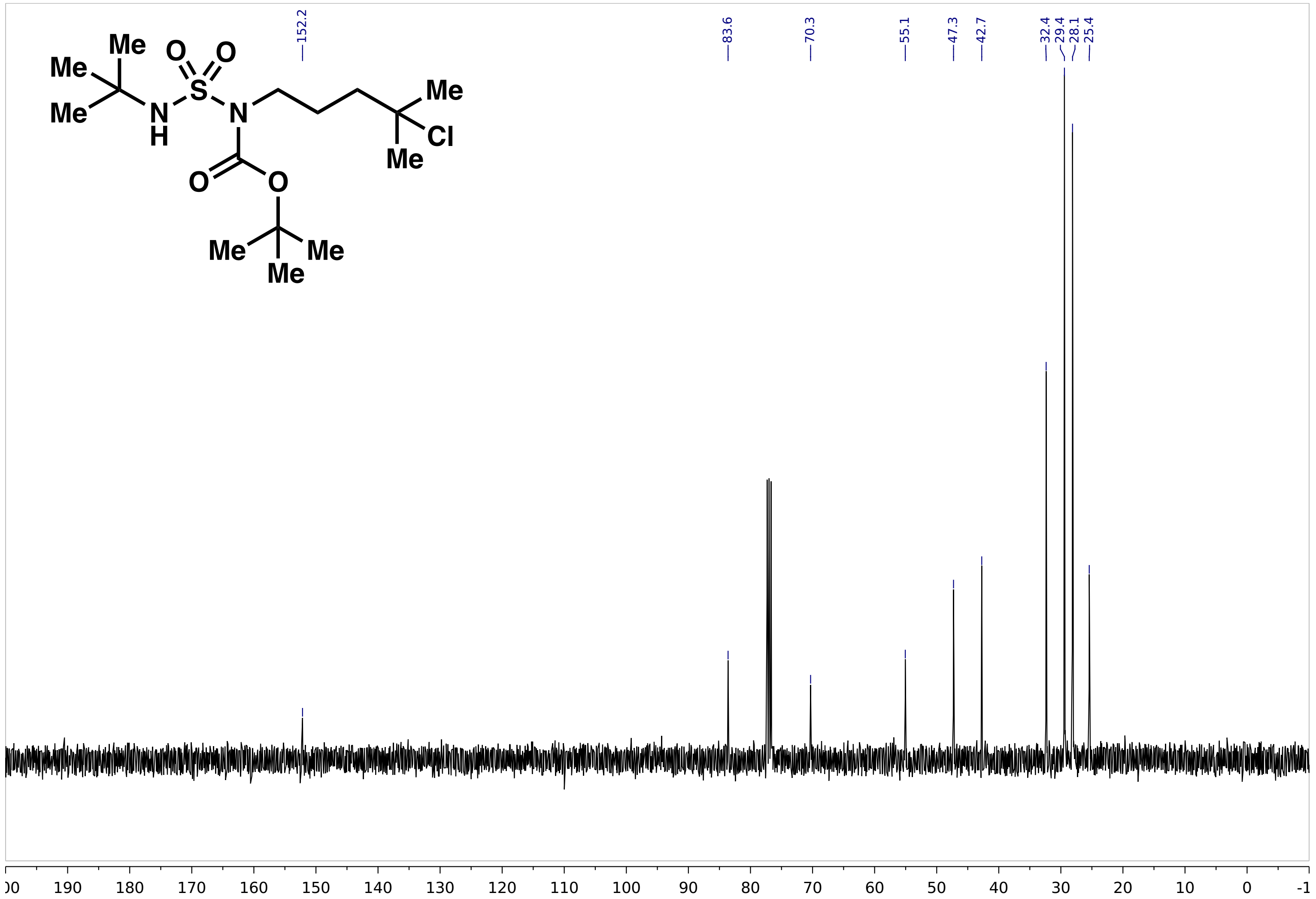

^{13}C NMR (101 MHz, $CDCl_3$) for *N*-*tert*-Butoxycarbonyl-*N'*-*tert*-butyl-*N*-(4-chloro-4-methylpentyl)sulfamide (**7q**)

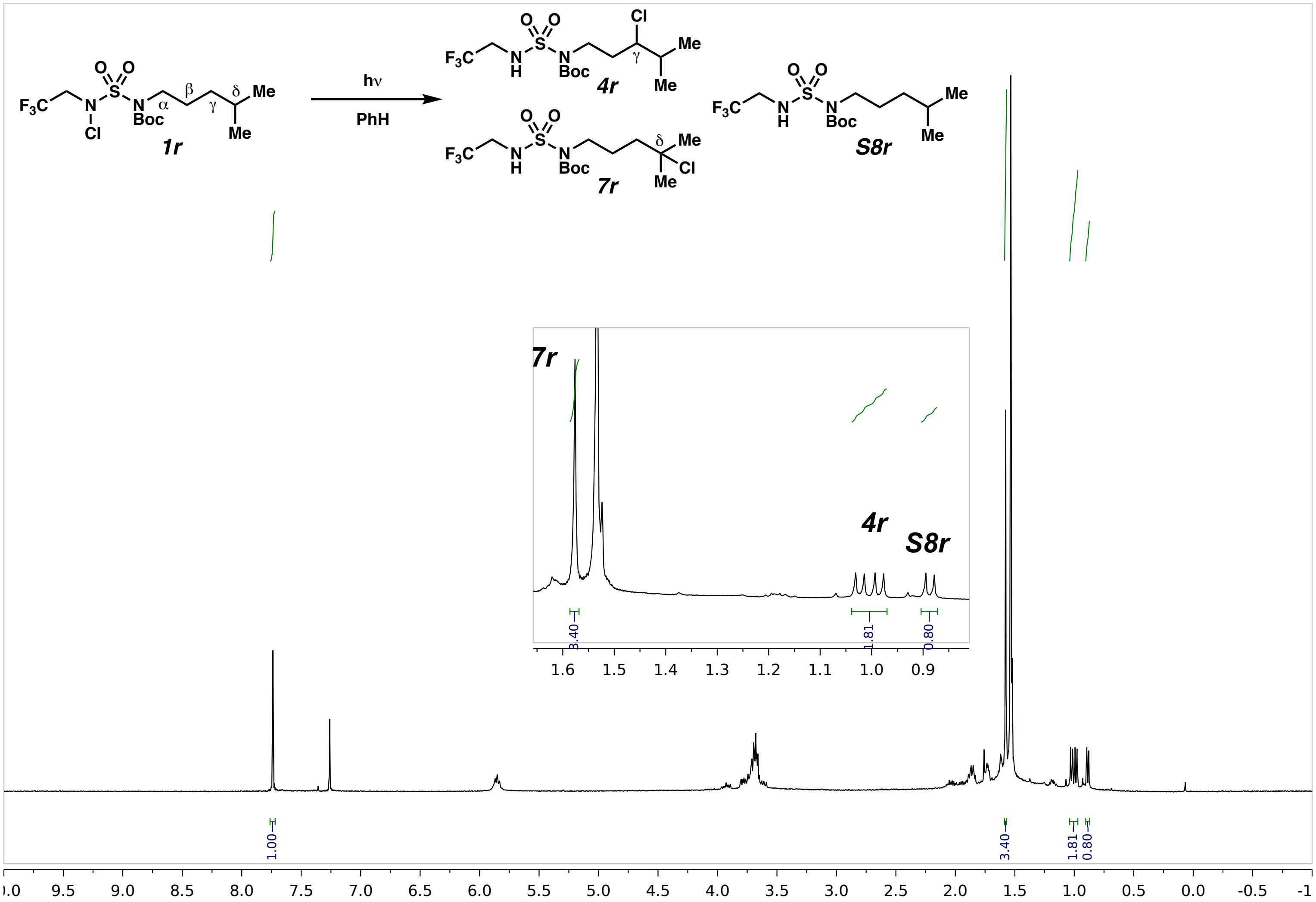

Crude ^{1}H NMR (500 MHz, $CDCl_3$, 25s relaxation delay) for chlorine-transfer reaction of *N*-chlorosulfamide **1r**

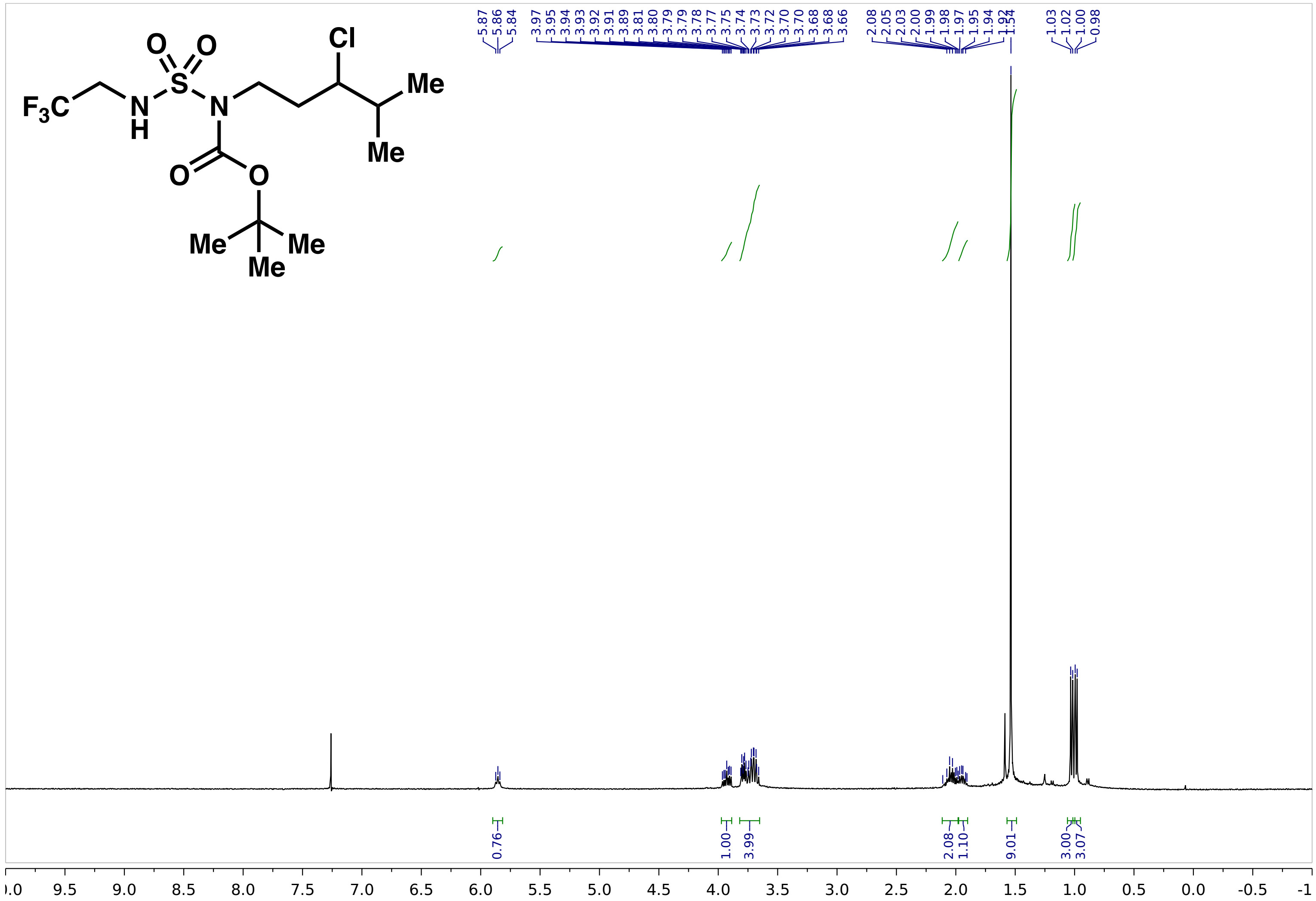

^{1}H NMR (400 MHz, $CDCl_3$) for *N*-*tert*-Butoxycarbonyl-*N*-(3-chloro-4-methylpentyl)-*N'*-(2,2,2-trifluoroethyl)sulfamide (**4r**)

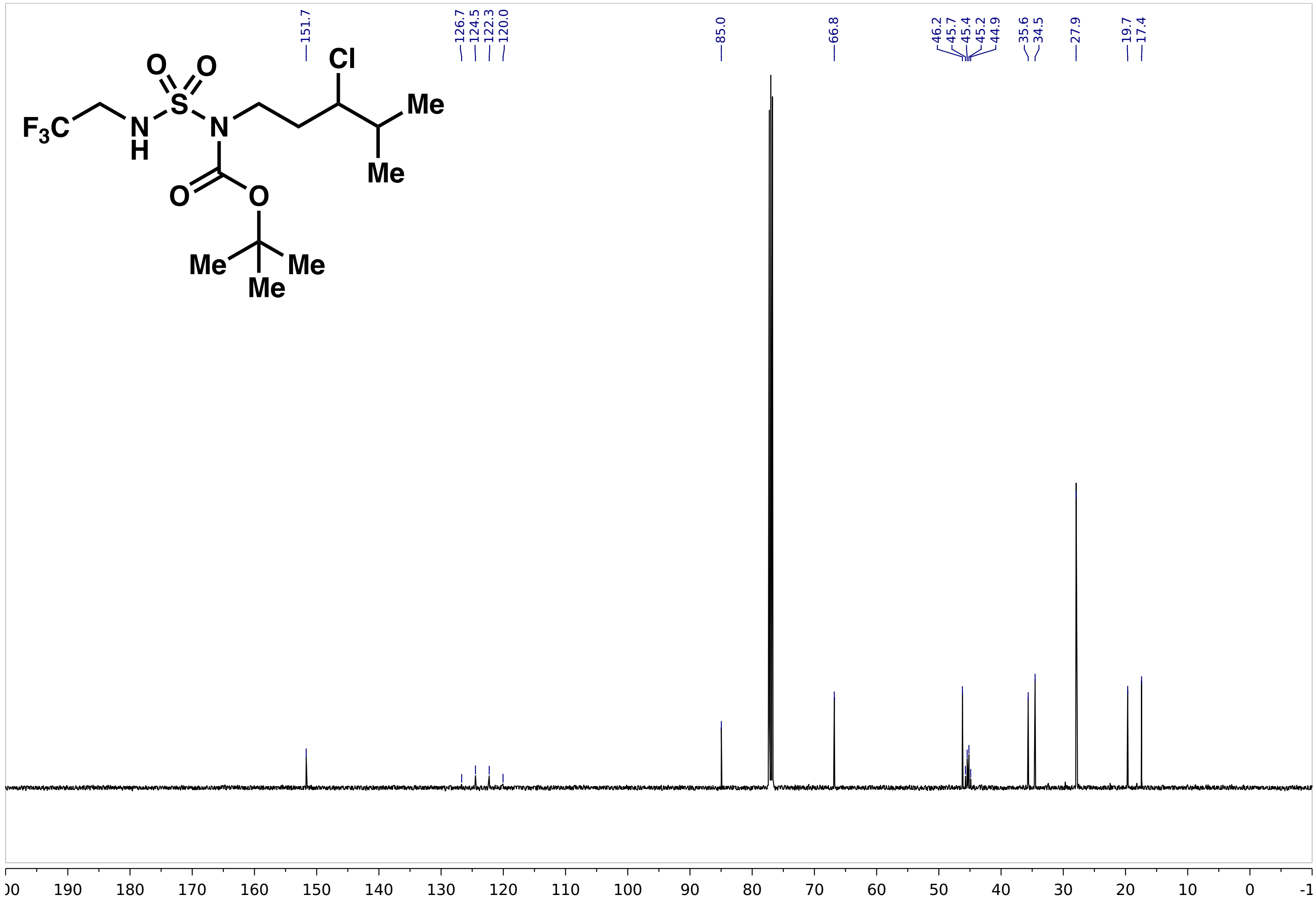

^{13}C NMR (126 MHz, $CDCl_3$) for *N*-*tert*-Butoxycarbonyl-*N*-(3-chloro-4-methylpentyl)-*N'*-(2,2,2-trifluoroethyl)sulfamide (**4r**)

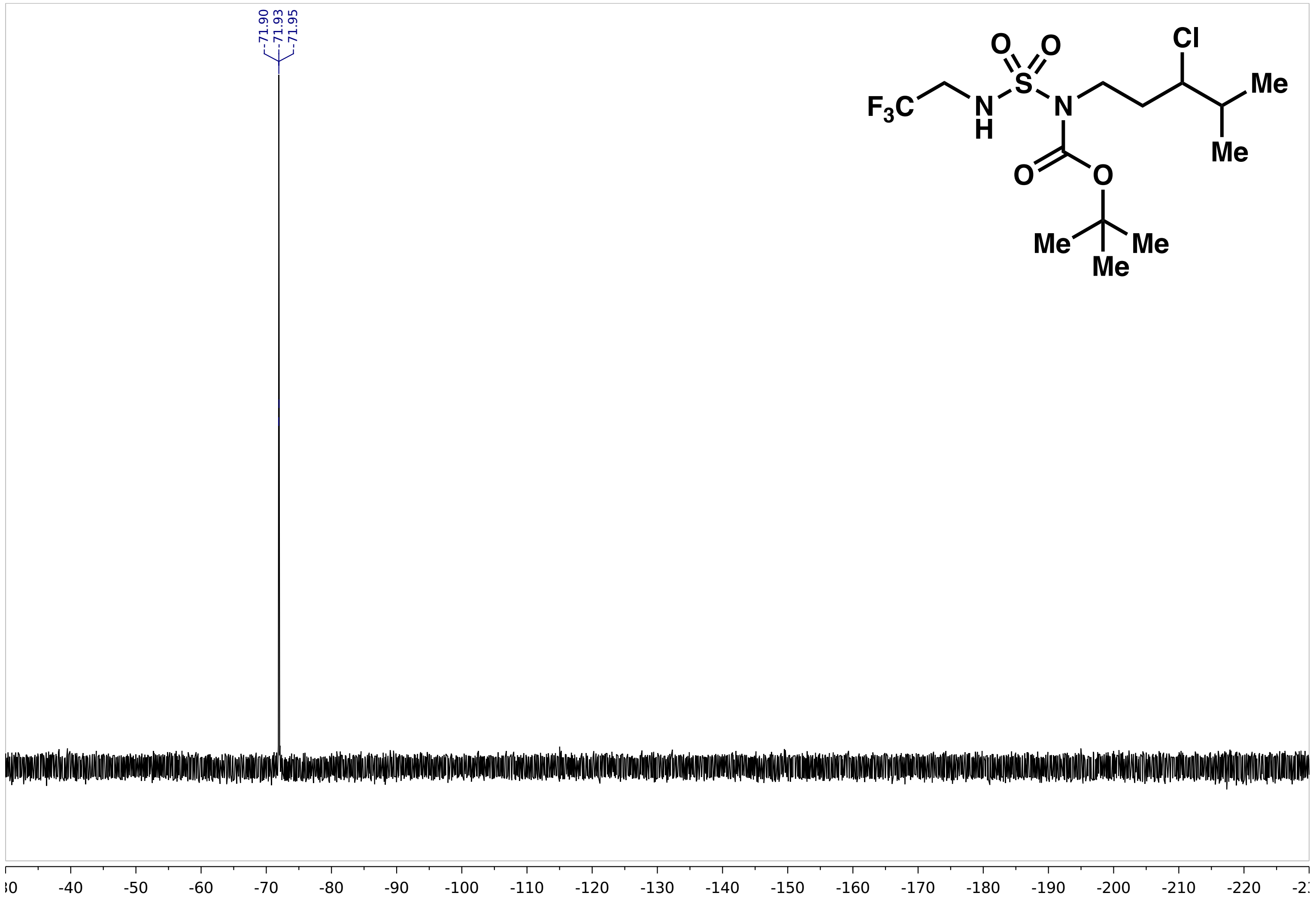

^{19}F NMR (376 MHz, $CDCl_3$) for *N*-*tert*-Butoxycarbonyl-*N*-(3-chloro-4-methylpentyl)-*N'*-(2,2,2-trifluoroethyl)sulfamide (**4r**)

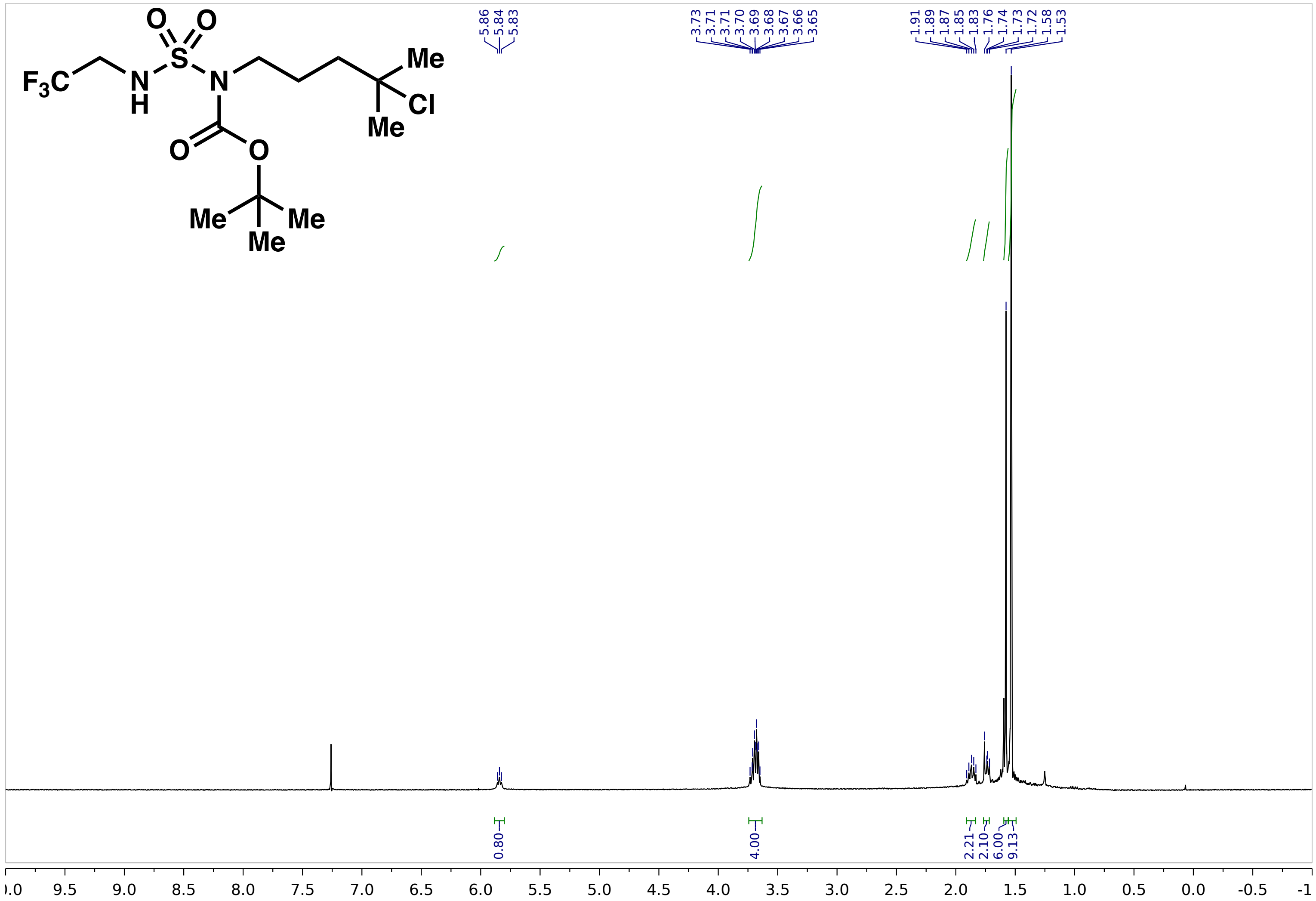

^{1}H NMR (400 MHz, $CDCl_3$) for *N*-*tert*-Butoxycarbonyl-*N*-(4-chloro-4-methylpentyl)-*N'*-(2,2,2-trifluoroethyl)sulfamide (**7r**)

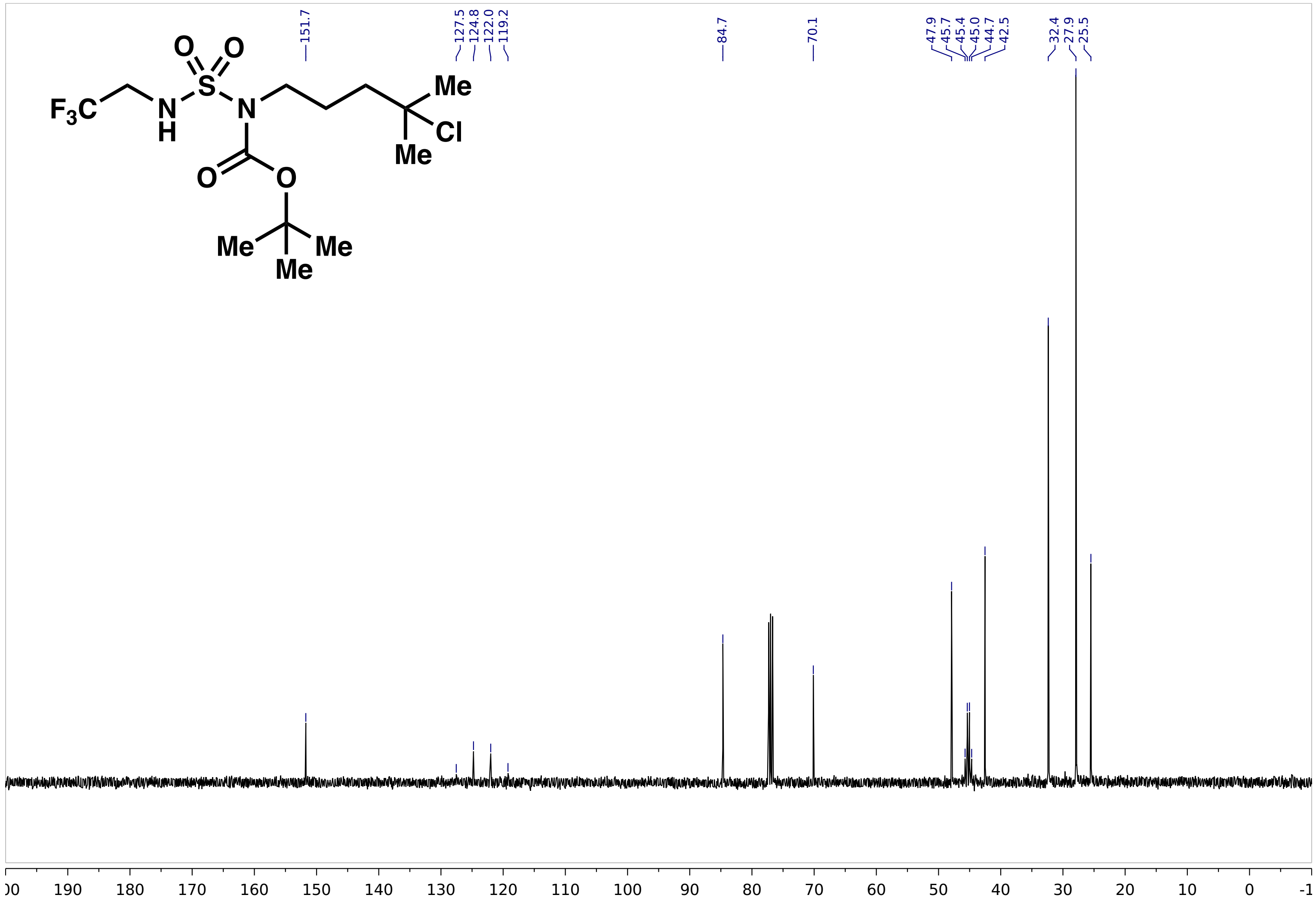

^{13}C NMR (101 MHz, $CDCl_3$) for *N*-*tert*-Butoxycarbonyl-*N*-(4-chloro-4-methylpentyl)-*N'*-(2,2,2-trifluoroethyl)sulfamide (**7r**)

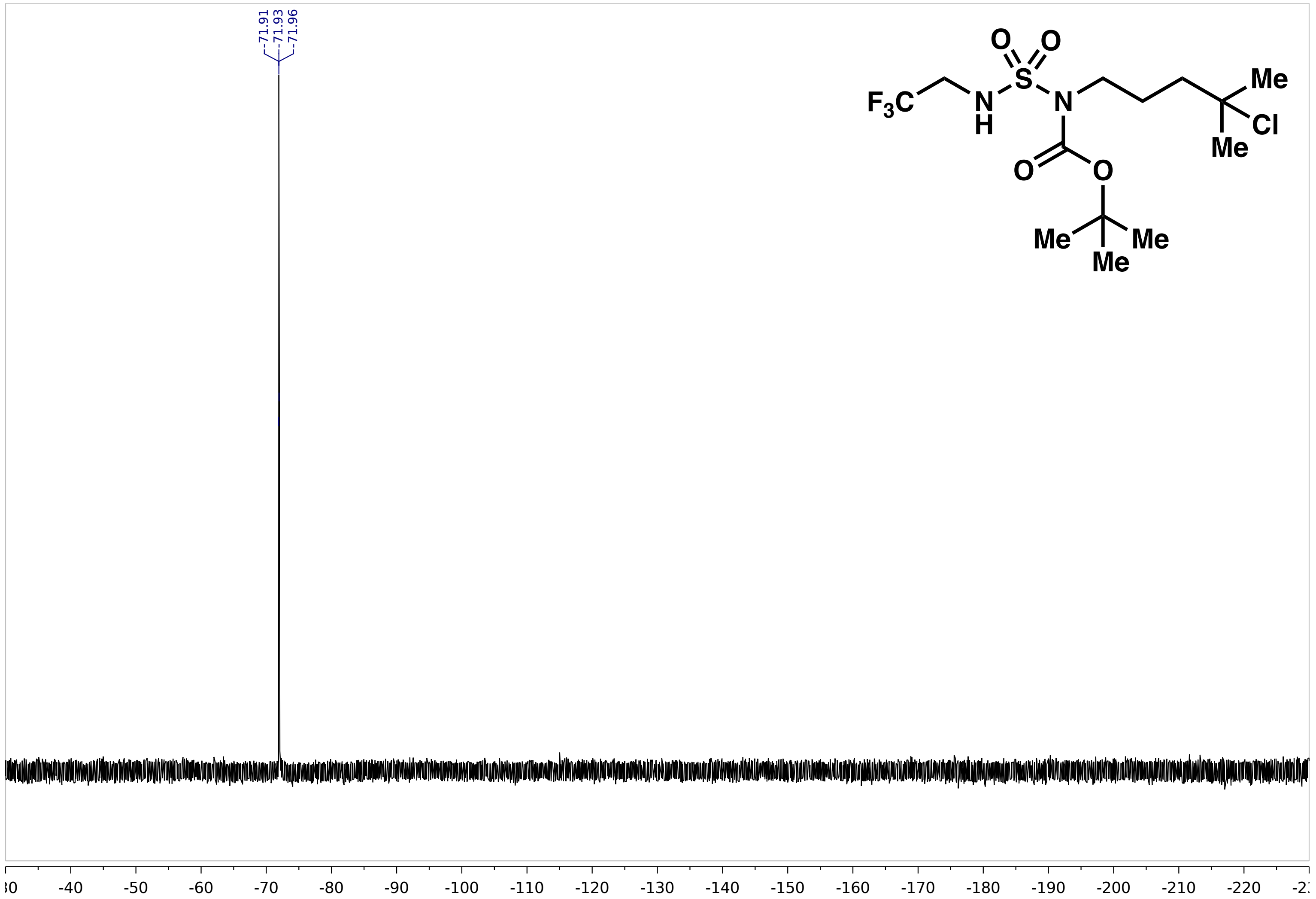

^{19}F NMR (376 MHz, $CDCl_3$) for *N*-*tert*-Butoxycarbonyl-*N*-(4-chloro-4-methylpentyl)-*N'*-(2,2,2-trifluoroethyl)sulfamide (**7r**)

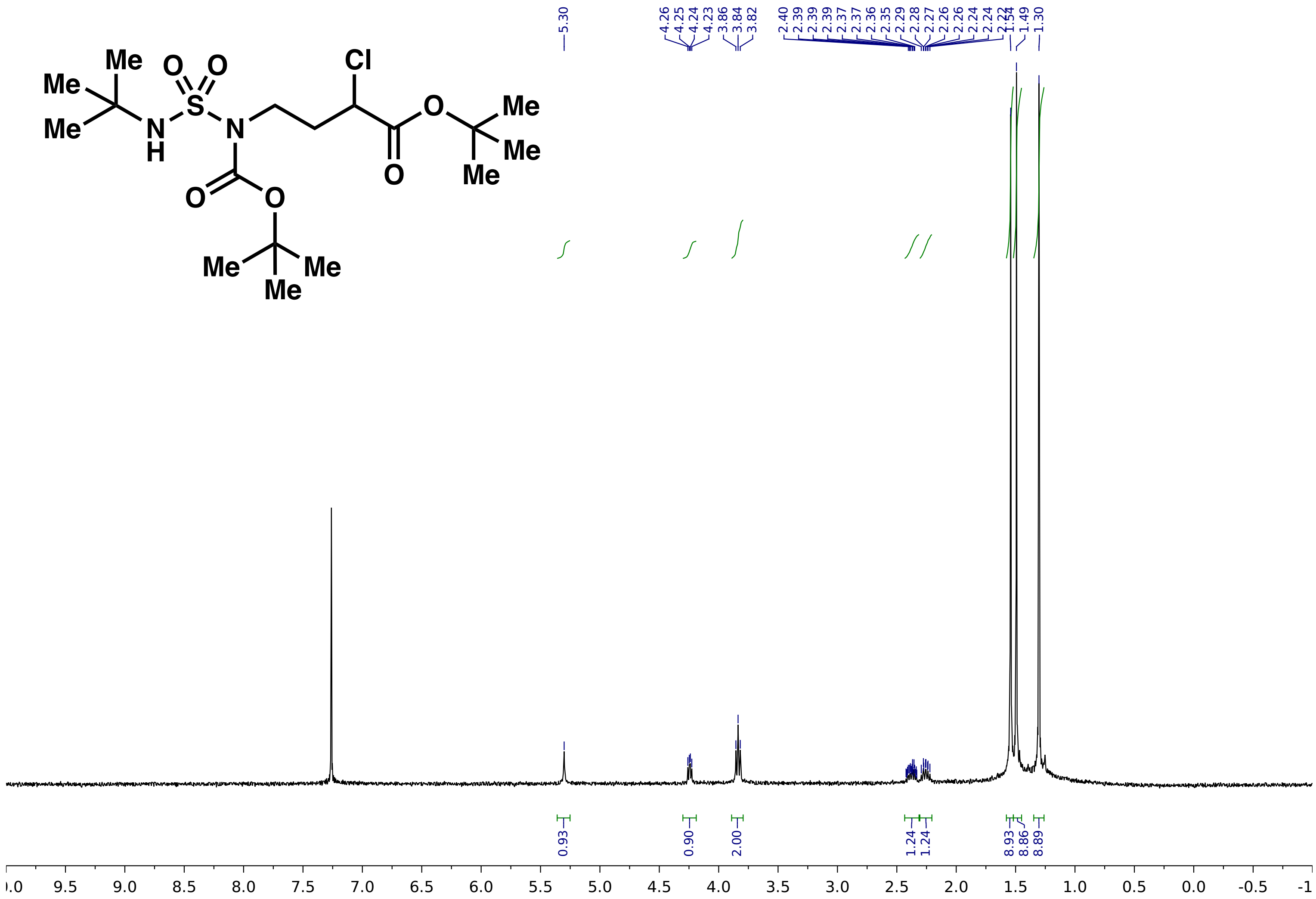

^{1}H NMR (400 MHz, $CDCl_3$) for *N*-*tert*-butoxycarbonyl-*N'*-*tert*-butyl-*N*-(*tert*-butyl 2-chlorobutano-4-yl)sulfamide (**S10t**)

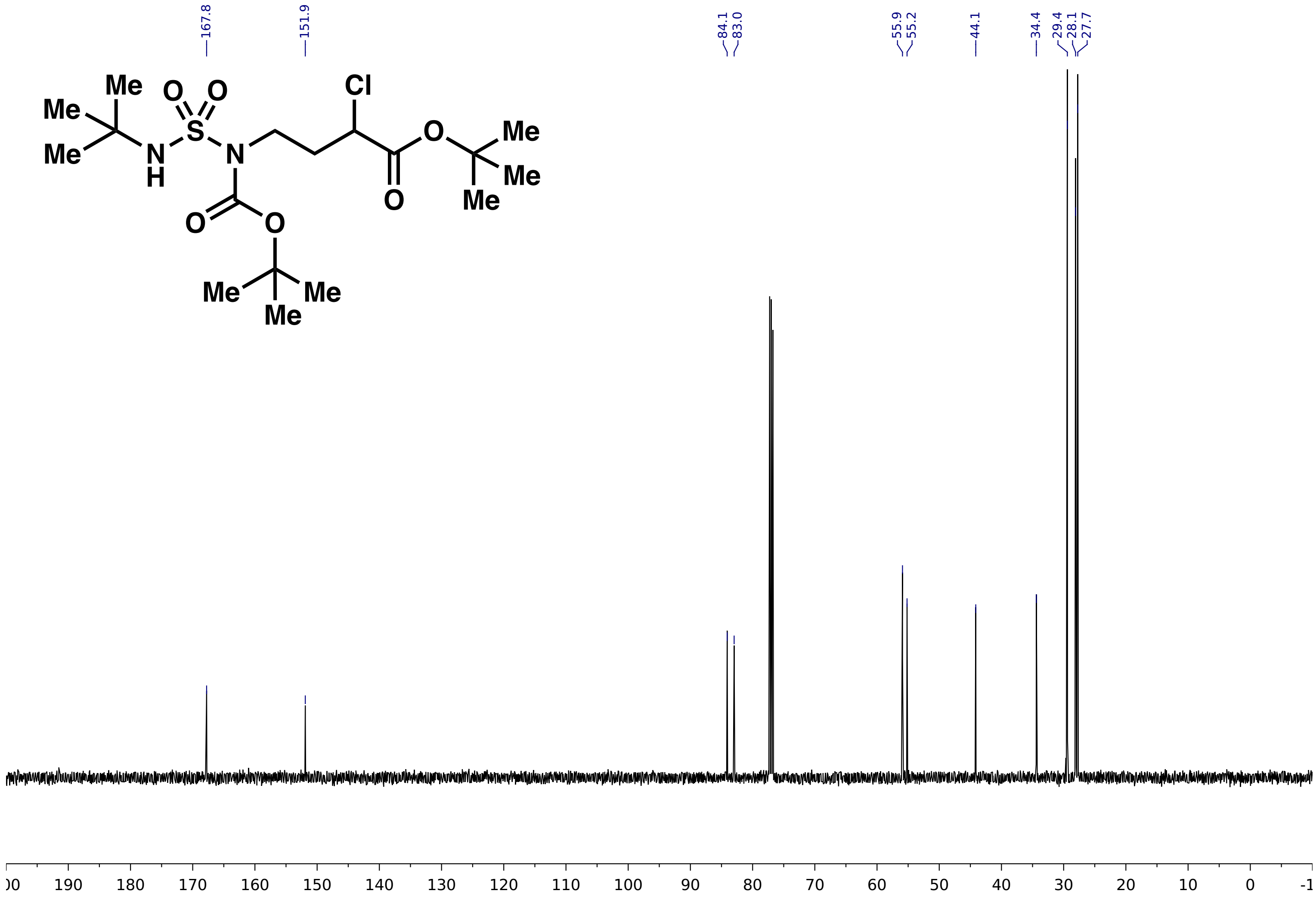

^{13}C NMR (126 MHz, $CDCl_3$) for *N*-*tert*-butoxycarbonyl-*N'*-*tert*-butyl-*N*-(*tert*-butyl 2-chlorobutano-4-yl)sulfamide (**S10t**)

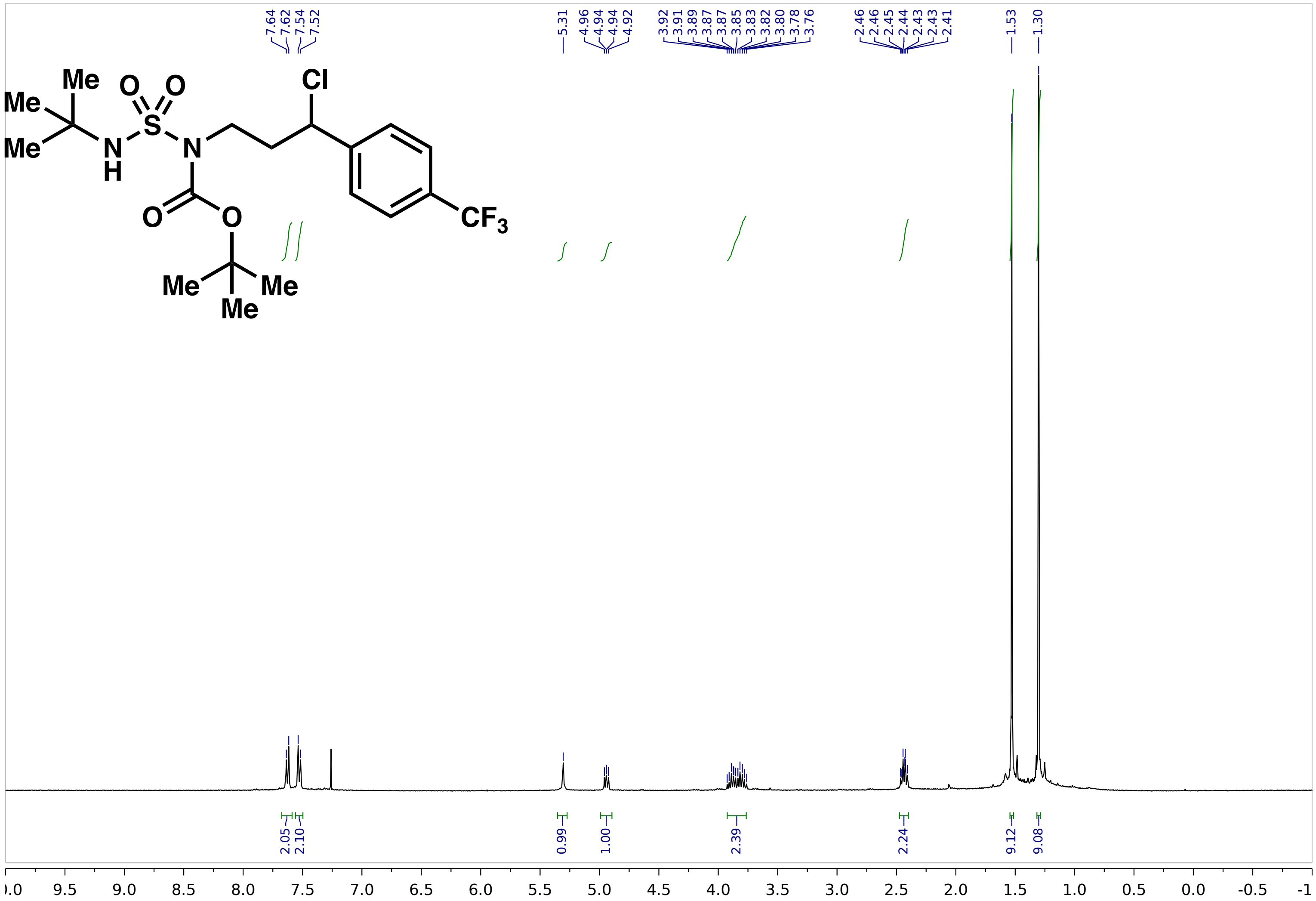

^{1}H NMR (400 MHz, $CDCl_3$) for *N*-*tert*-butoxycarbonyl-*N'*-*tert*-butyl-*N*-(3-chloro-3-((4-trifluoromethyl)phenyl)prop-1-yl)sulfamide **(S10u)**

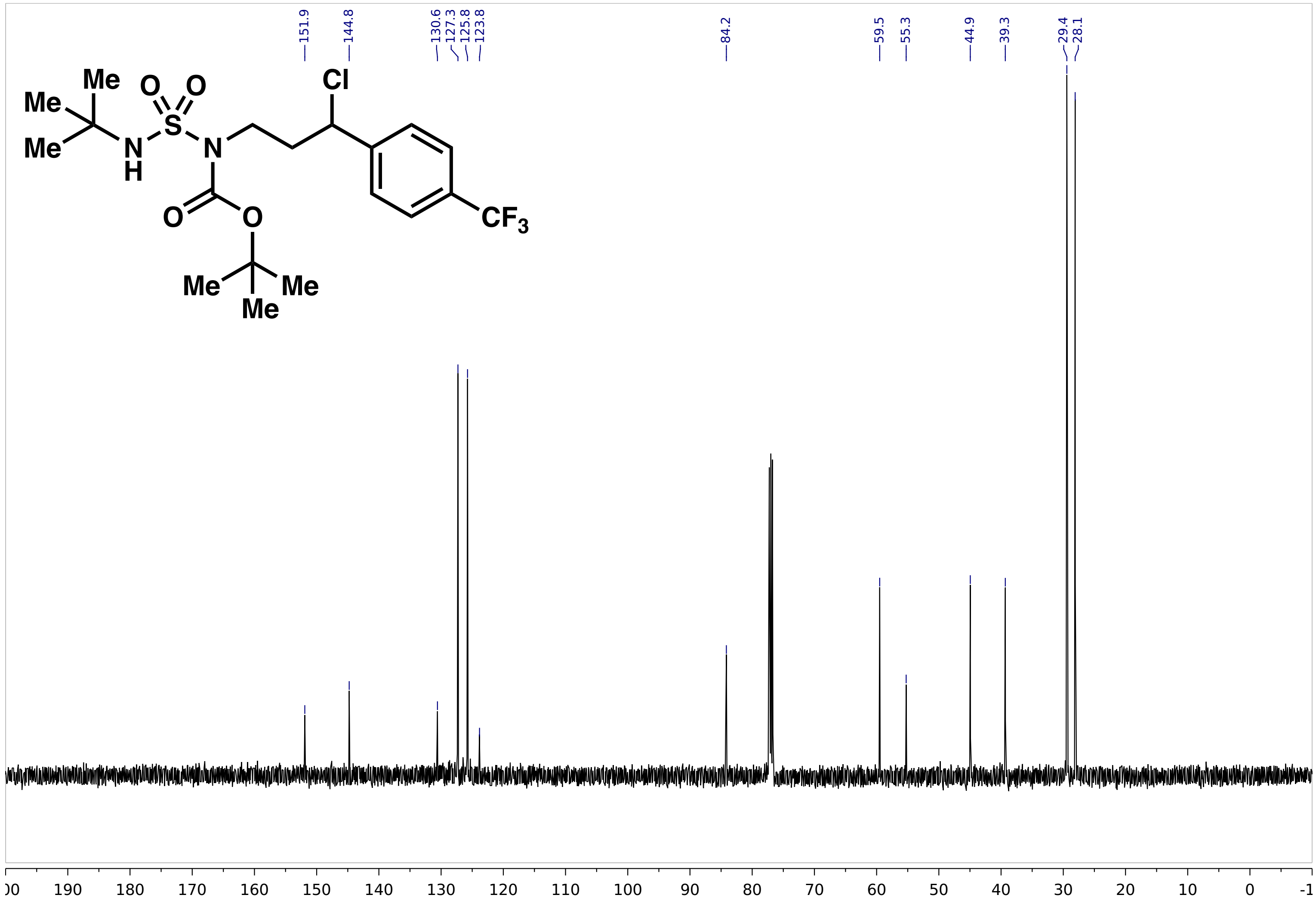

^{13}C NMR (126 MHz, CDCl$_3$, ^{1}H and ^{19}F decoupled) for *N*-*tert*-butoxycarbonyl-*N'*-*tert*-butyl-*N*-(3-chloro-3-((4-trifluoromethyl)phenyl)prop-1-yl)sulfamide (**S10u**)

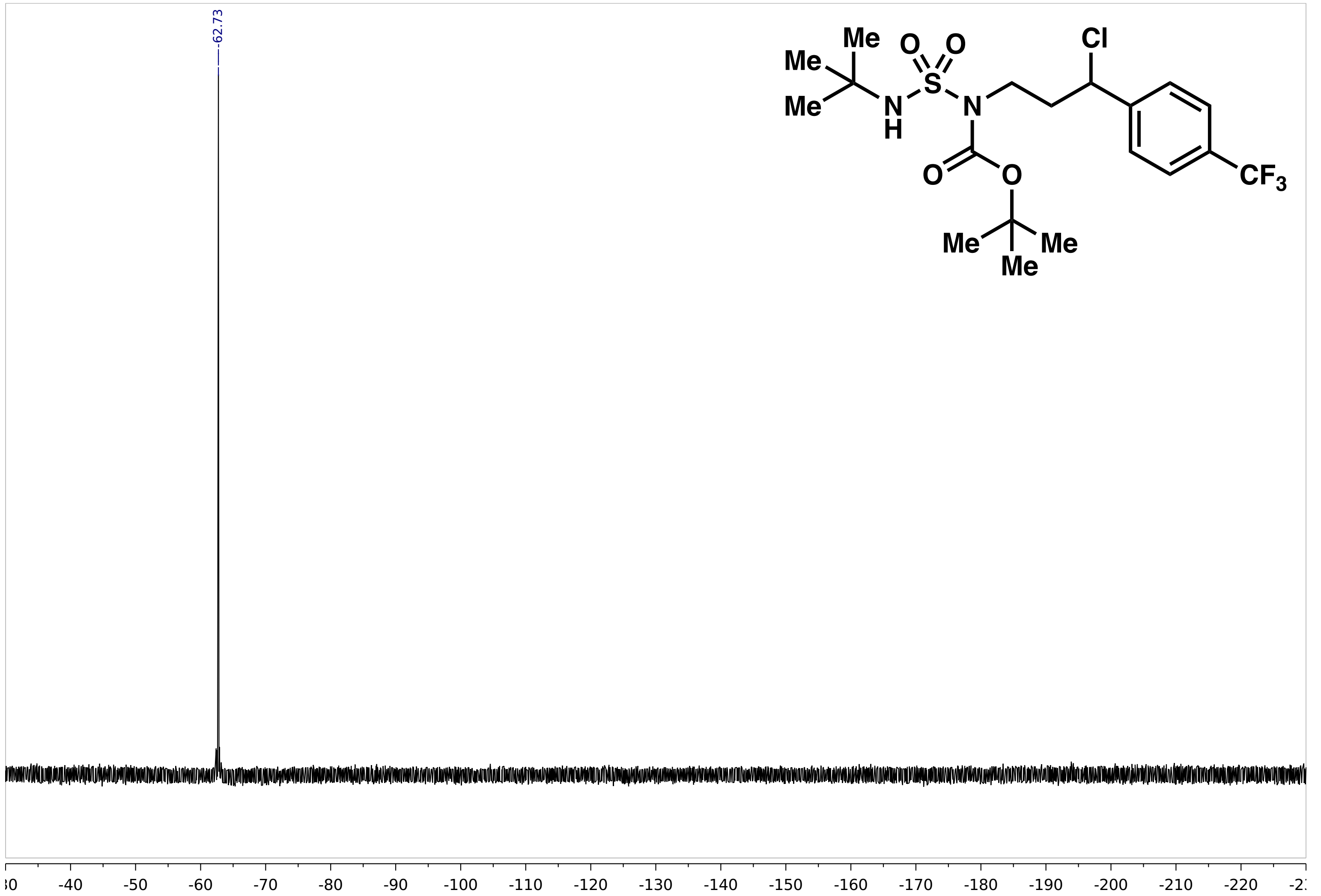

^{19}F NMR (376 MHz, $CDCl_3$) for *N*-*tert*-butoxycarbonyl-*N'*-*tert*-butyl-*N*-(3-chloro-3-((4-trifluoromethyl)phenyl)prop-1-yl)sulfamide (**S10u**)

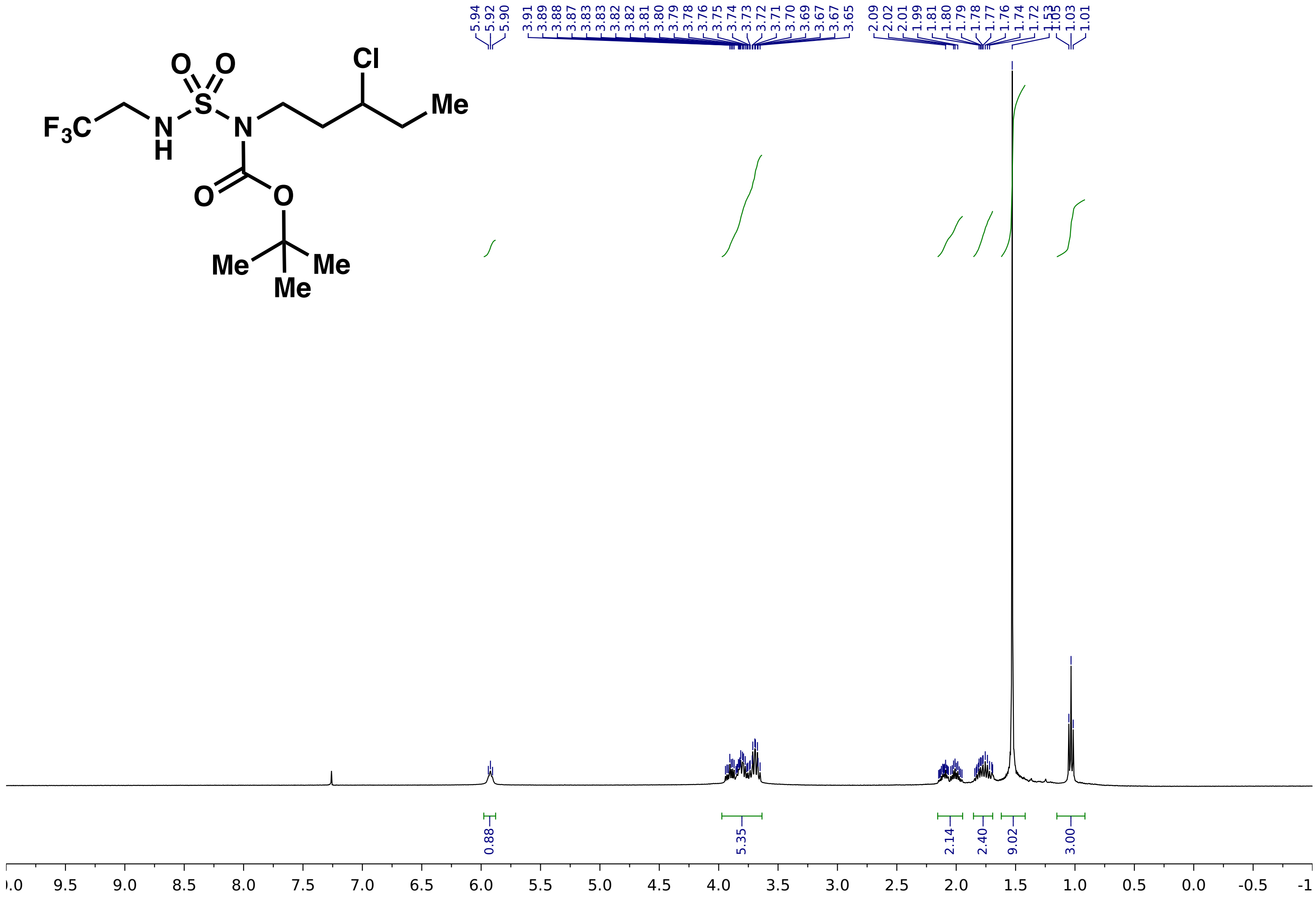

^{1}H NMR (400 MHz, $CDCl_3$) for *N*-*tert*-butoxycarbonyl-*N*-(3-chloropent-1-yl)-*N'*-(2,2,2-trfiluoroethyl)sulfamide (**S10v**)

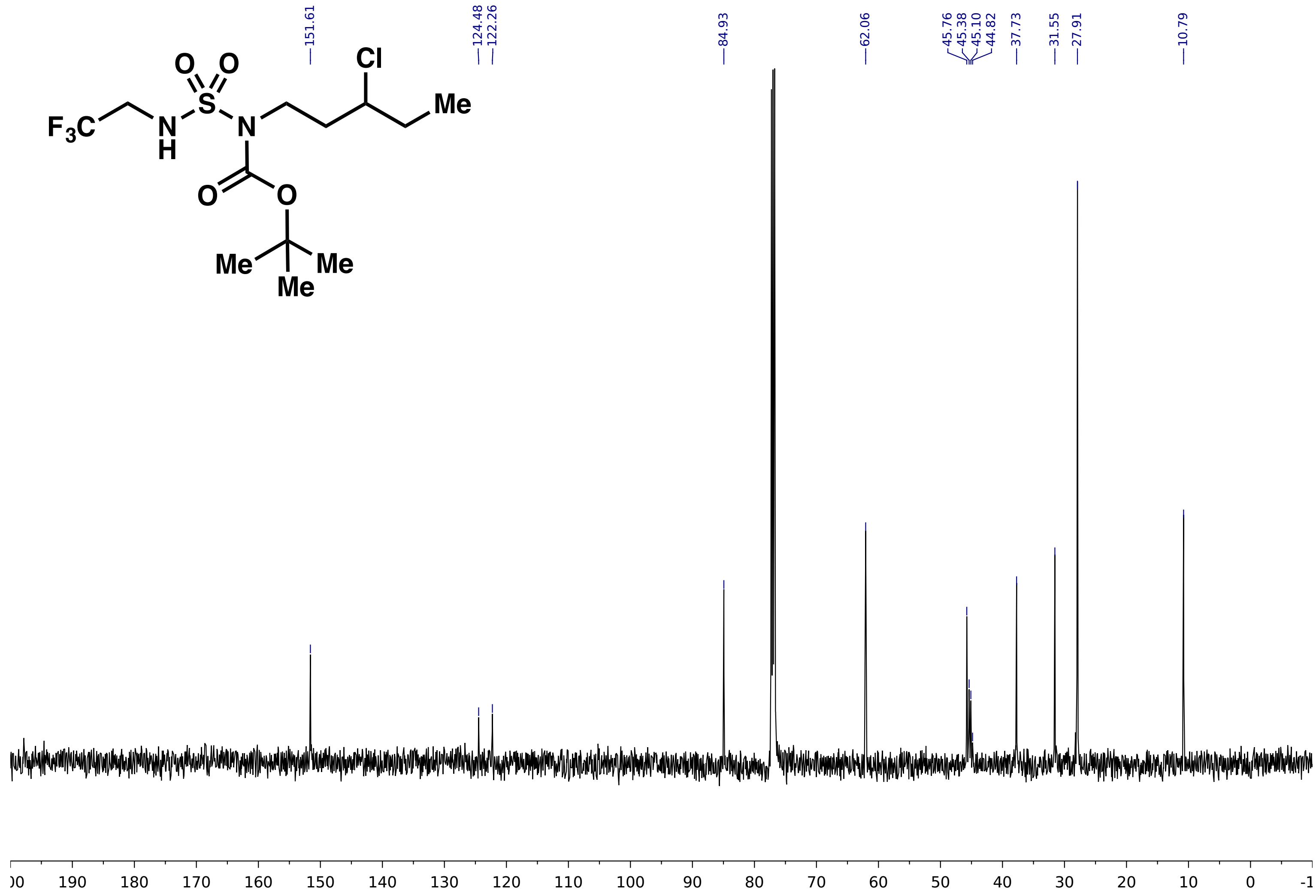

^{13}C NMR (126 MHz, CDCl$_3$) for *N*-*tert*-butoxycarbonyl-*N*-(3-chloropent-1-yl)-*N'*-(2,2,2-trfiluoroethyl)sulfamide (**S10v**)

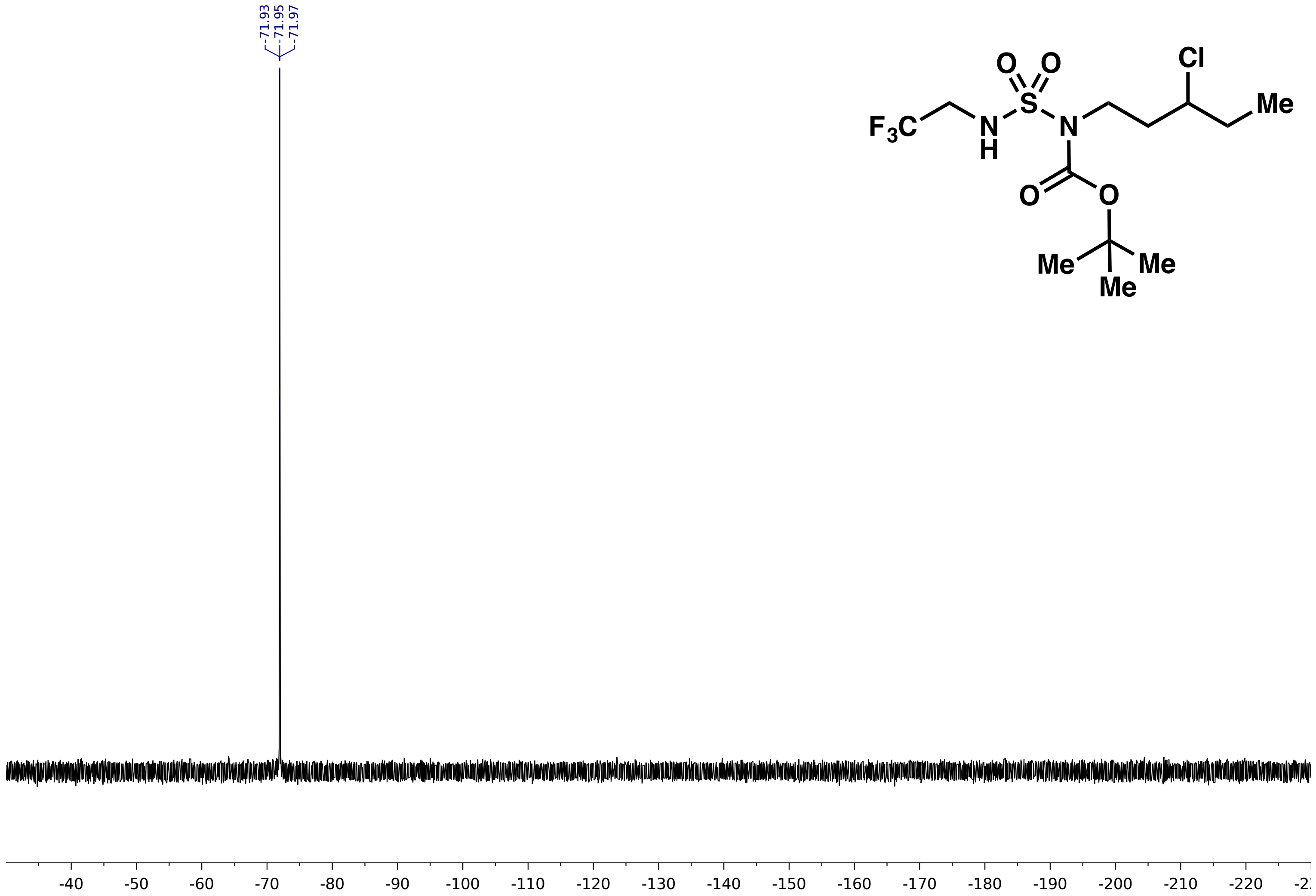

^{19}F NMR (376 MHz, $CDCl_3$) for *N*-*tert*-butoxycarbonyl-*N*-(3-chloropent-1-yl)-*N'*-(2,2,2-trfiluoroethyl)sulfamide (**S10v**)

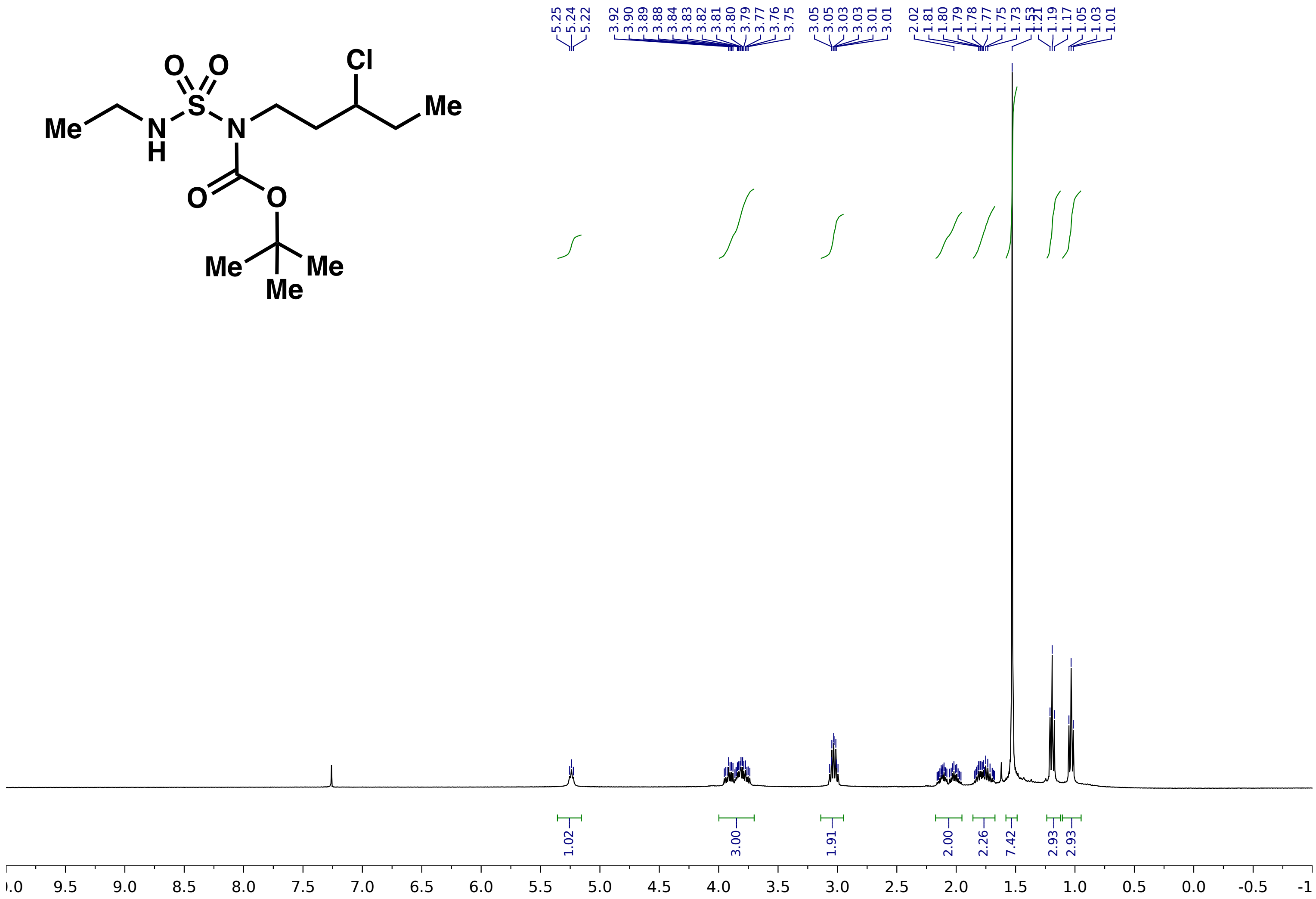

^{1}H NMR (400 MHz, $CDCl_3$) for *N*-*tert*-butoxycarbonyl-*N*-(3-chloropent-1-yl)-*N'*-ethylsulfamide (**S10w**)

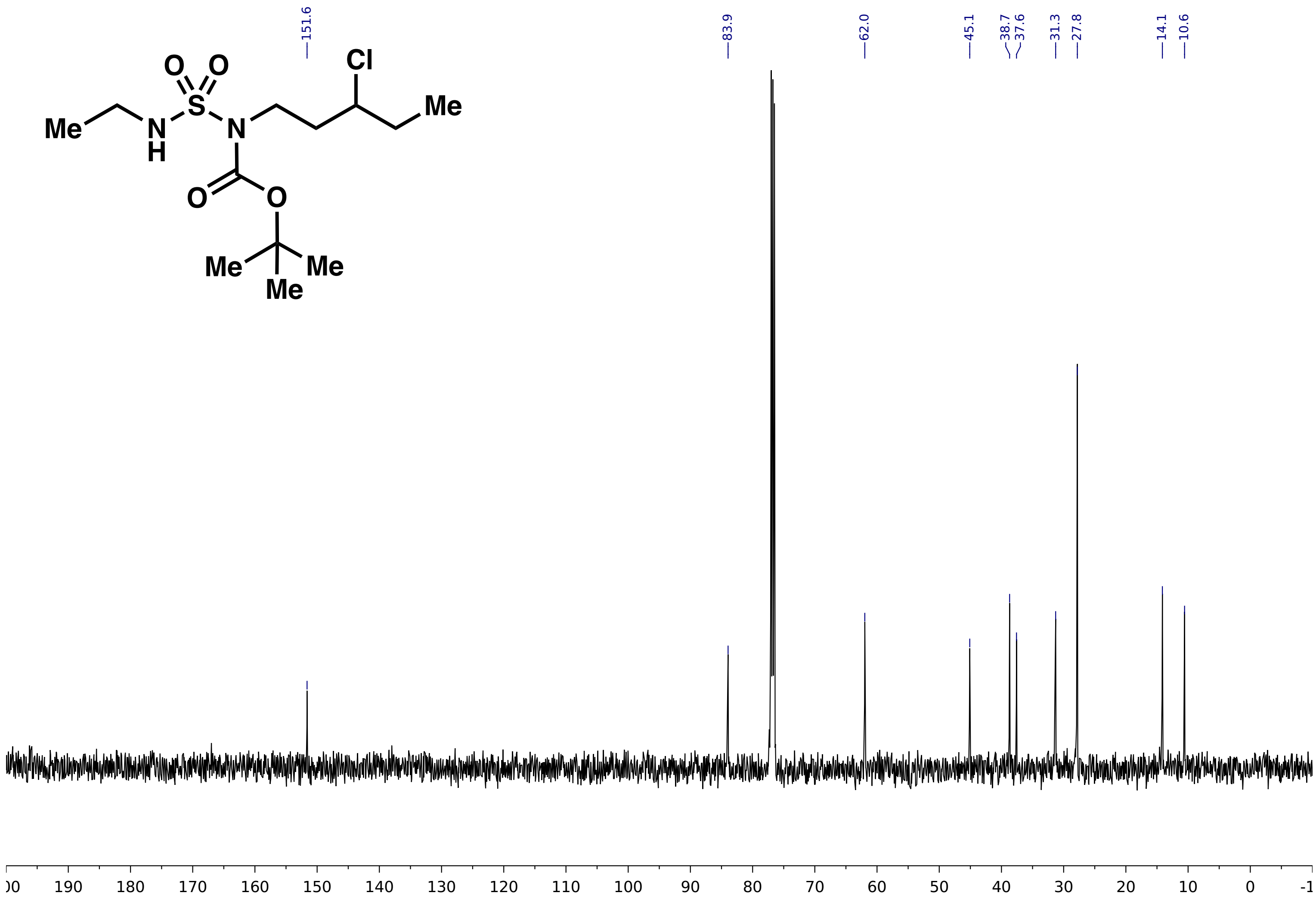

^{13}C NMR (126 MHz, $CDCl_3$) for *N*-*tert*-butoxycarbonyl-*N*-(3-chloropent-1-yl)-*N'*-ethylsulfamide (**S10w**)

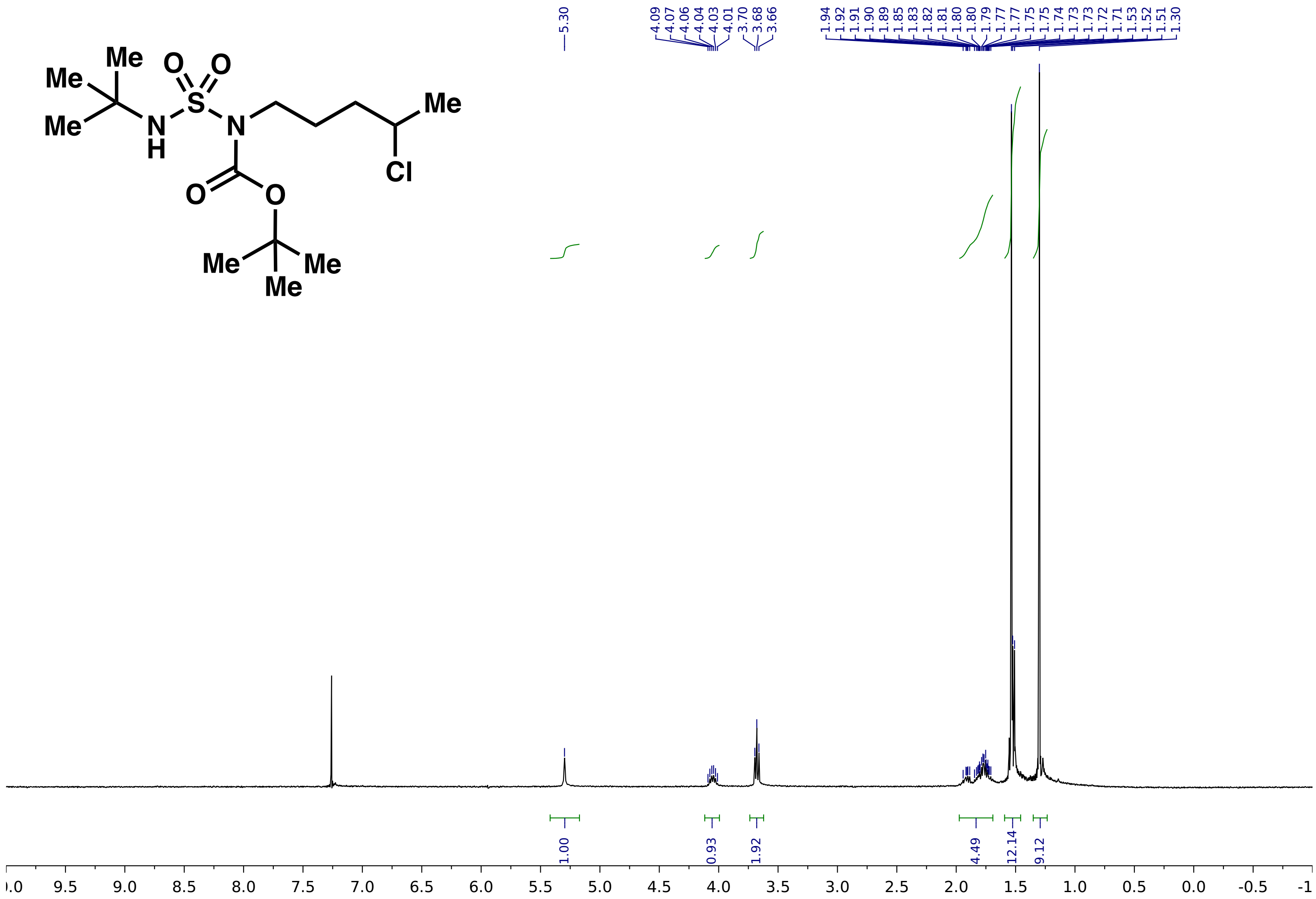

^{1}H NMR (400 MHz, $CDCl_3$) for *N*-*tert*-butoxycarbonyl-*N'*-*tert*-butyl-*N*-(4-chloropent-1-yl)sulfamide (**7i**)

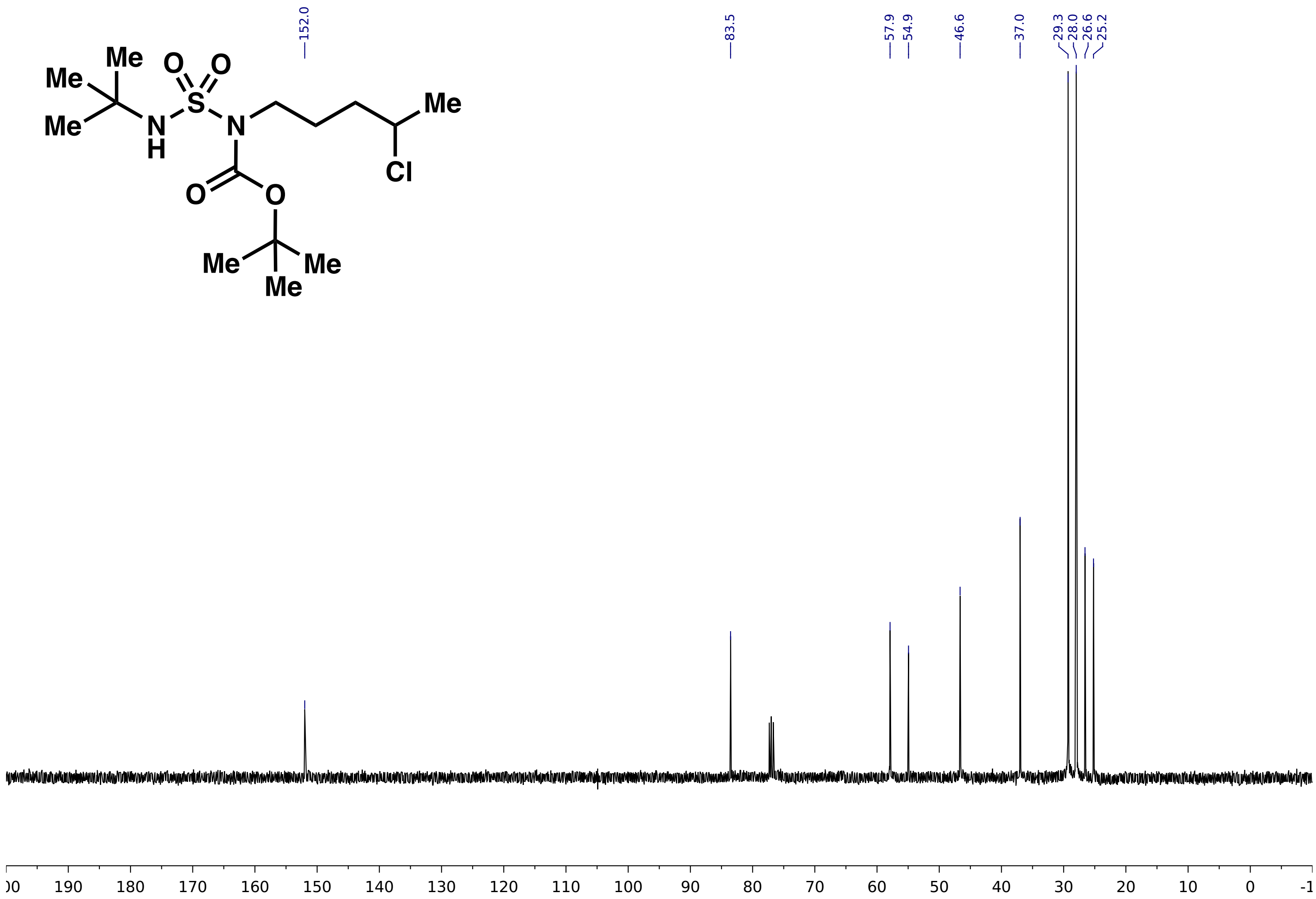

^{13}C NMR (126 MHz, $CDCl_3$) for *N*-*tert*-butoxycarbonyl-*N'*-*tert*-butyl-*N*-(4-chloropent-1-yl)sulfamide (**7i**)

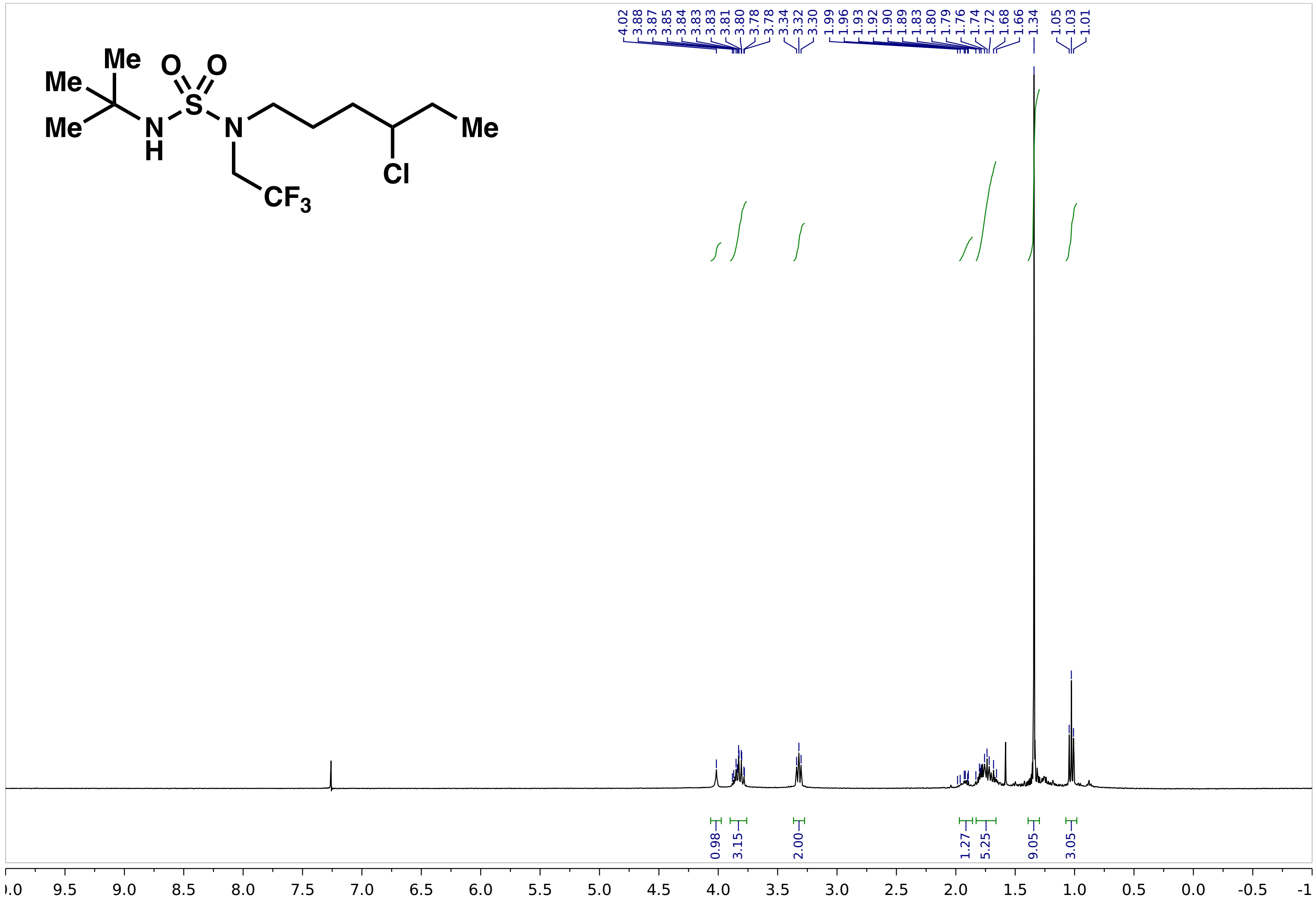

^{1}H NMR (400 MHz, $CDCl_3$) for *N*-*tert*-butyl-*N'*-(4-chlorohex-1-yl)-*N*'-(2,2,2-trifluoroethyl)sulfamide (**7m**)

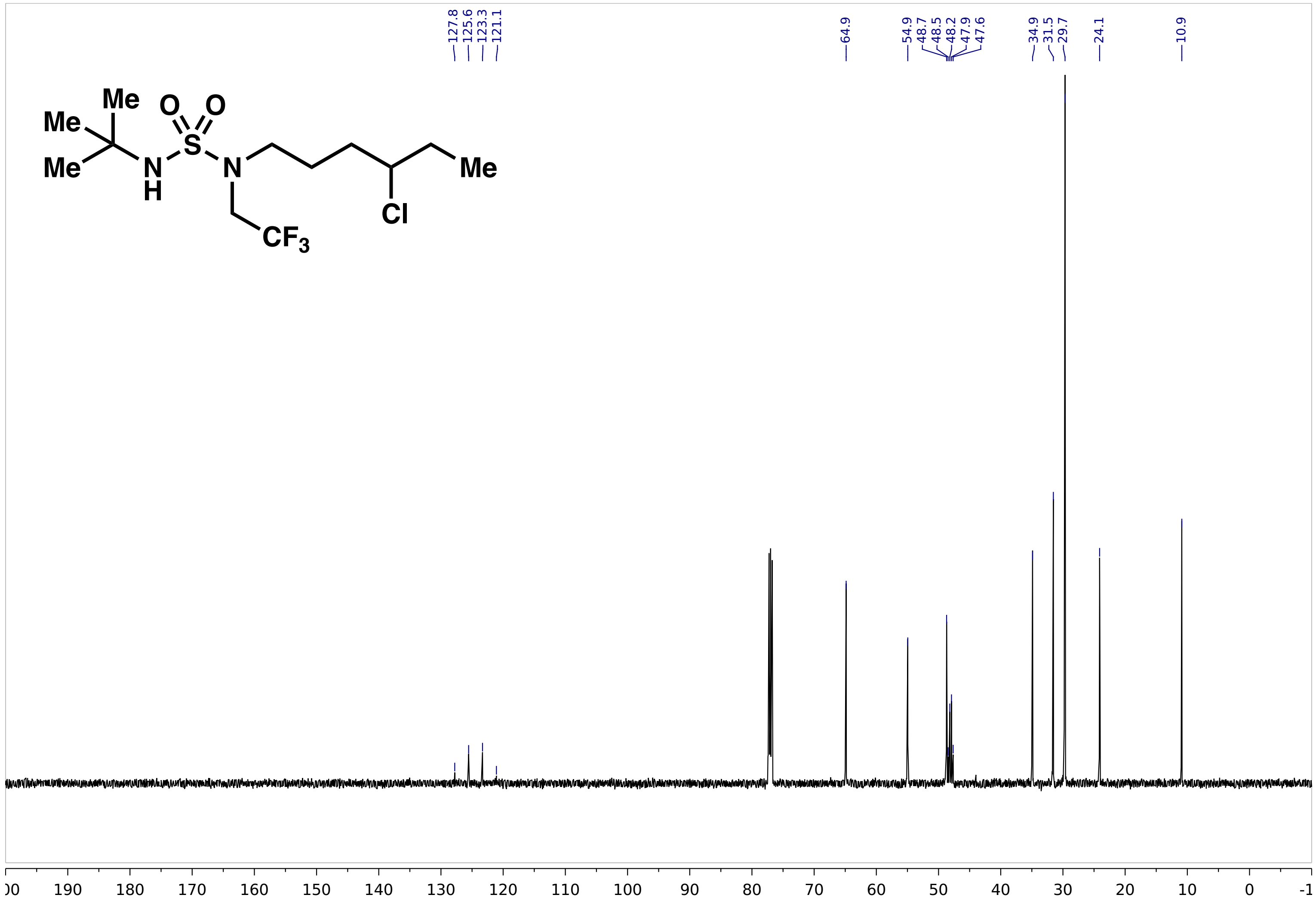

^{13}C NMR (126 MHz, $CDCl_3$) for *N-tert*-butyl-*N'*-(4-chlorohex-1-yl)-*N*'-(2,2,2-trifluoroethyl)sulfamide (**7m**)

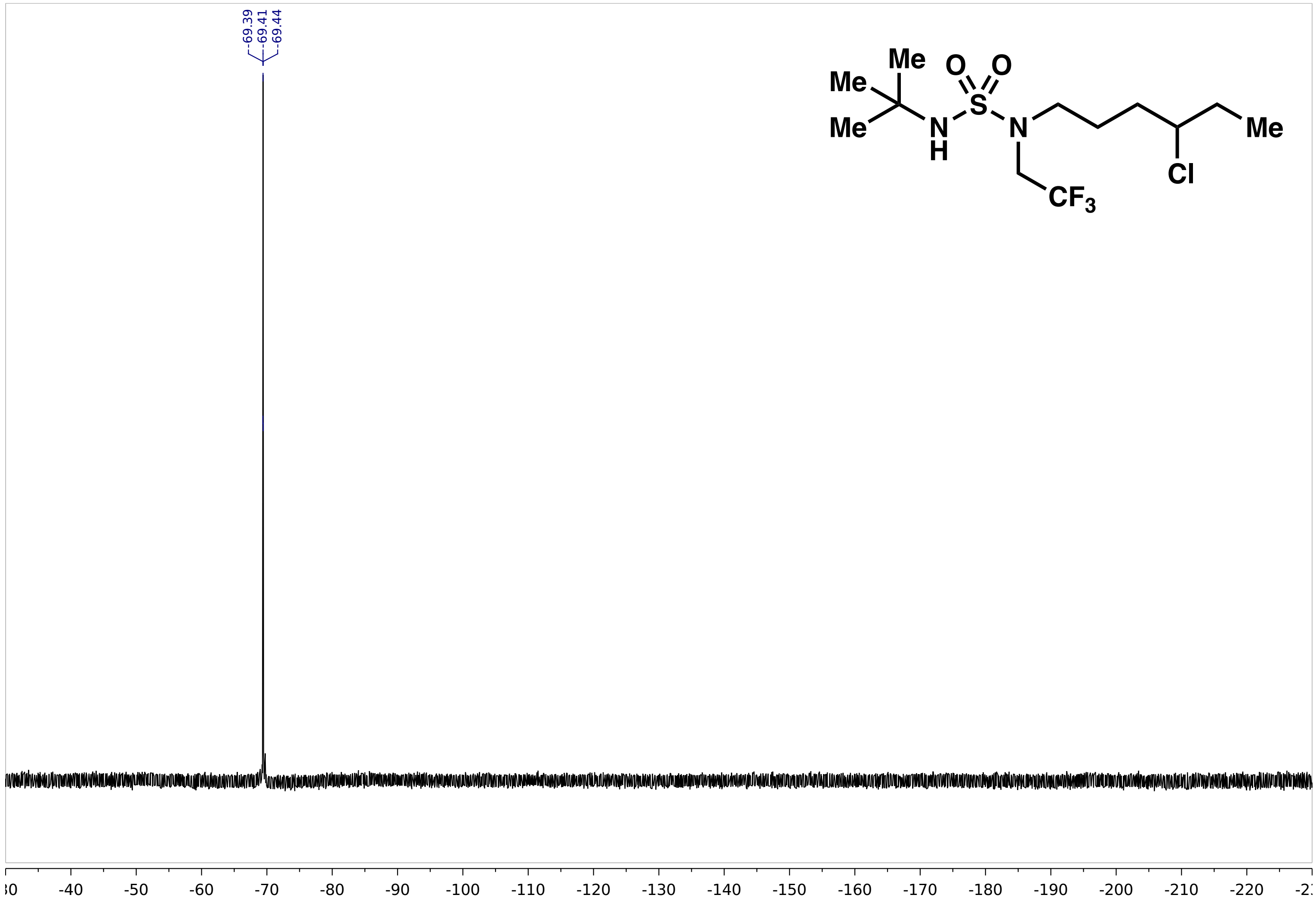

^{19}F NMR (376 MHz, $CDCl_3$) for *N*-*tert*-butyl-*N'*-(4-chlorohex-1-yl)-*N*'-(2,2,2-trifluoroethyl)sulfamide (**7m**)

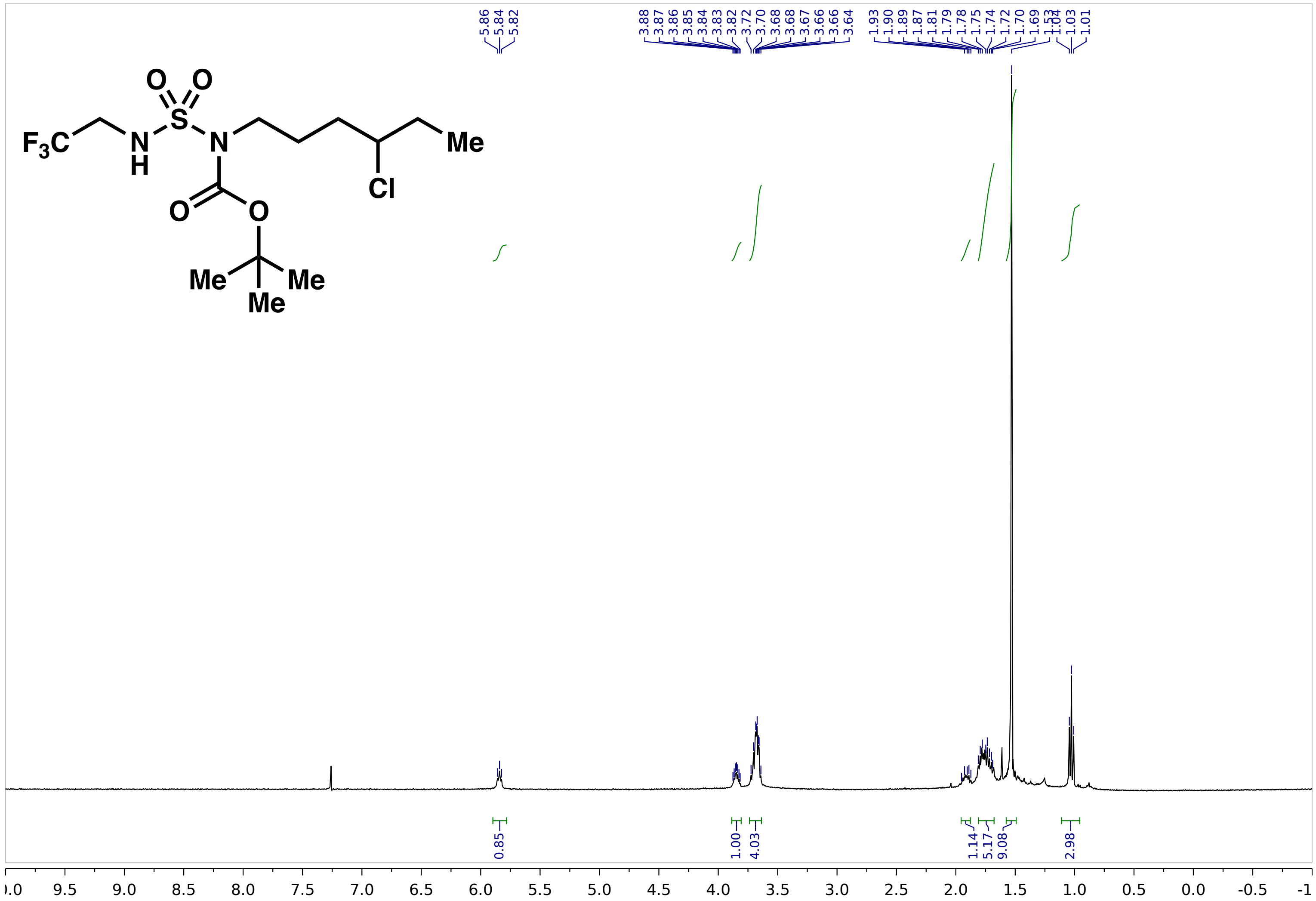

^{1}H NMR (400 MHz, $CDCl_3$) for *N*-*tert*-butoxycarbonyl-*N*-(4-chlorohex-1-yl)-*N*'-(2,2,2-trifluoroethyl)sulfamide (**7o**)

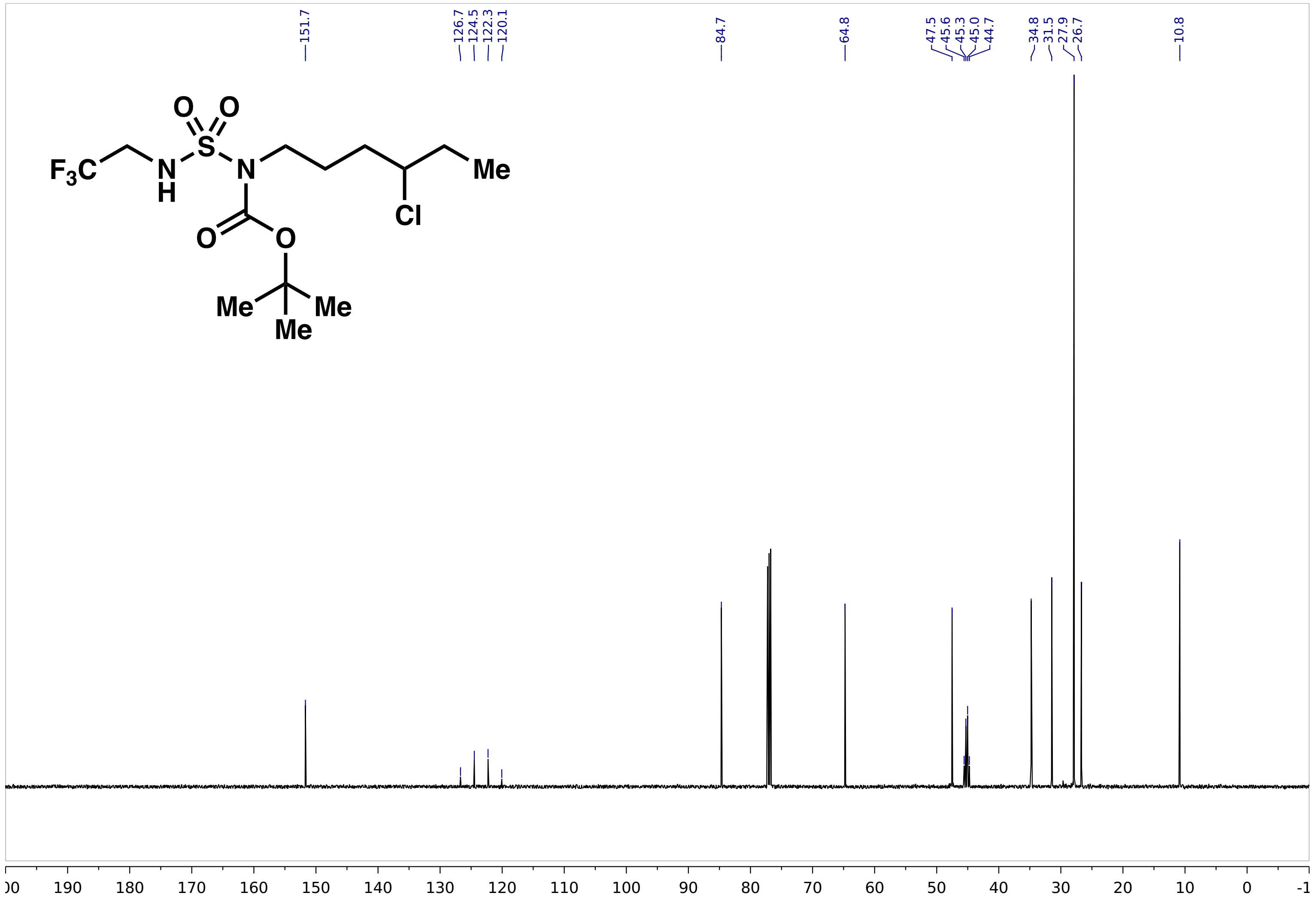

^{13}C NMR (126 MHz, $CDCl_3$) for *N*-*tert*-butoxycarbonyl-*N*-(4-chlorohex-1-yl)-*N*'-(2,2,2-trifluoroethyl)sulfamide (**7o**)

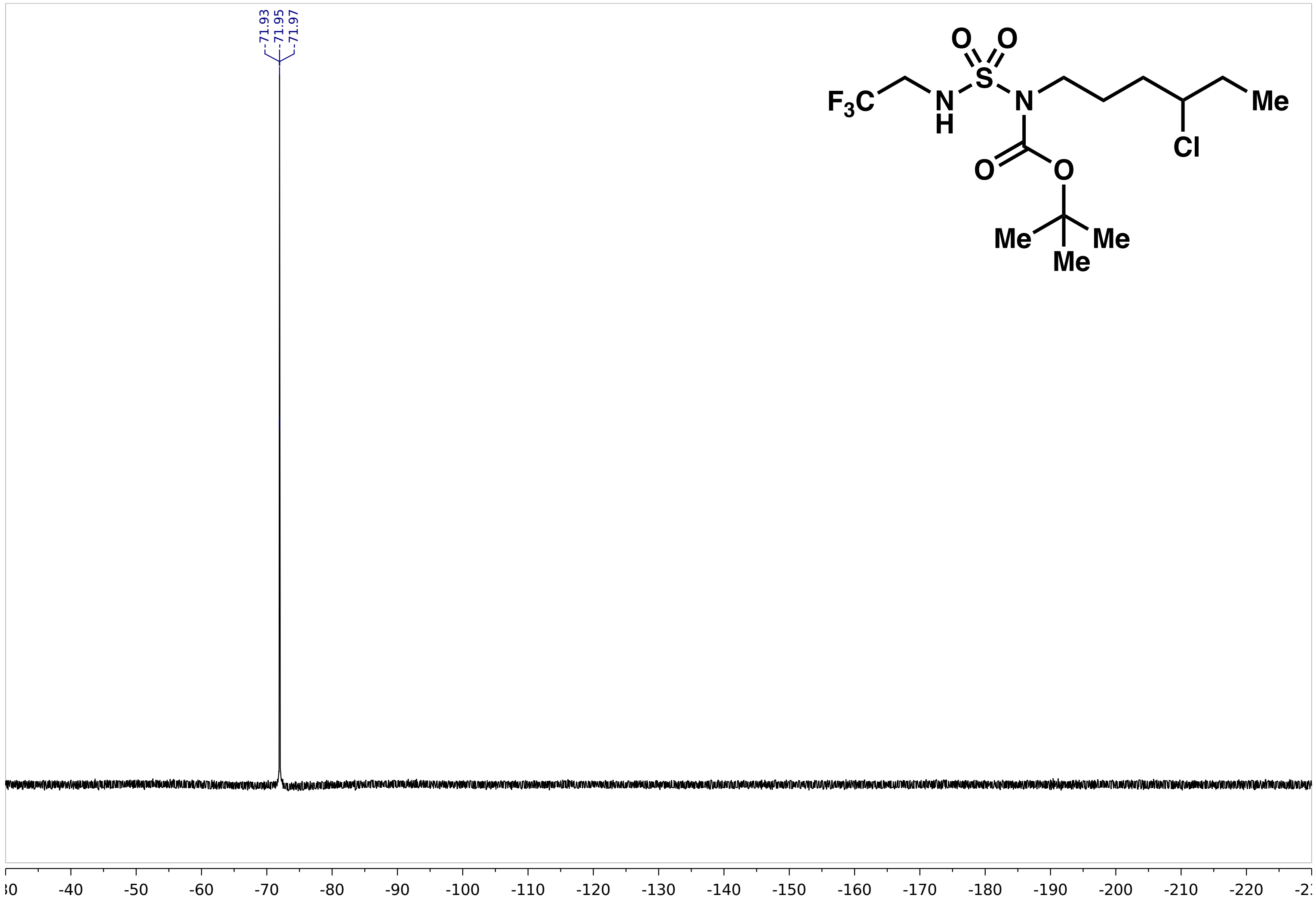

^{19}F NMR (376 MHz, $CDCl_3$) for *N*-*tert*-butoxycarbonyl-*N*-(4-chlorohex-1-yl)-*N*'-(2,2,2-trifluoroethyl)sulfamide (**7o**)

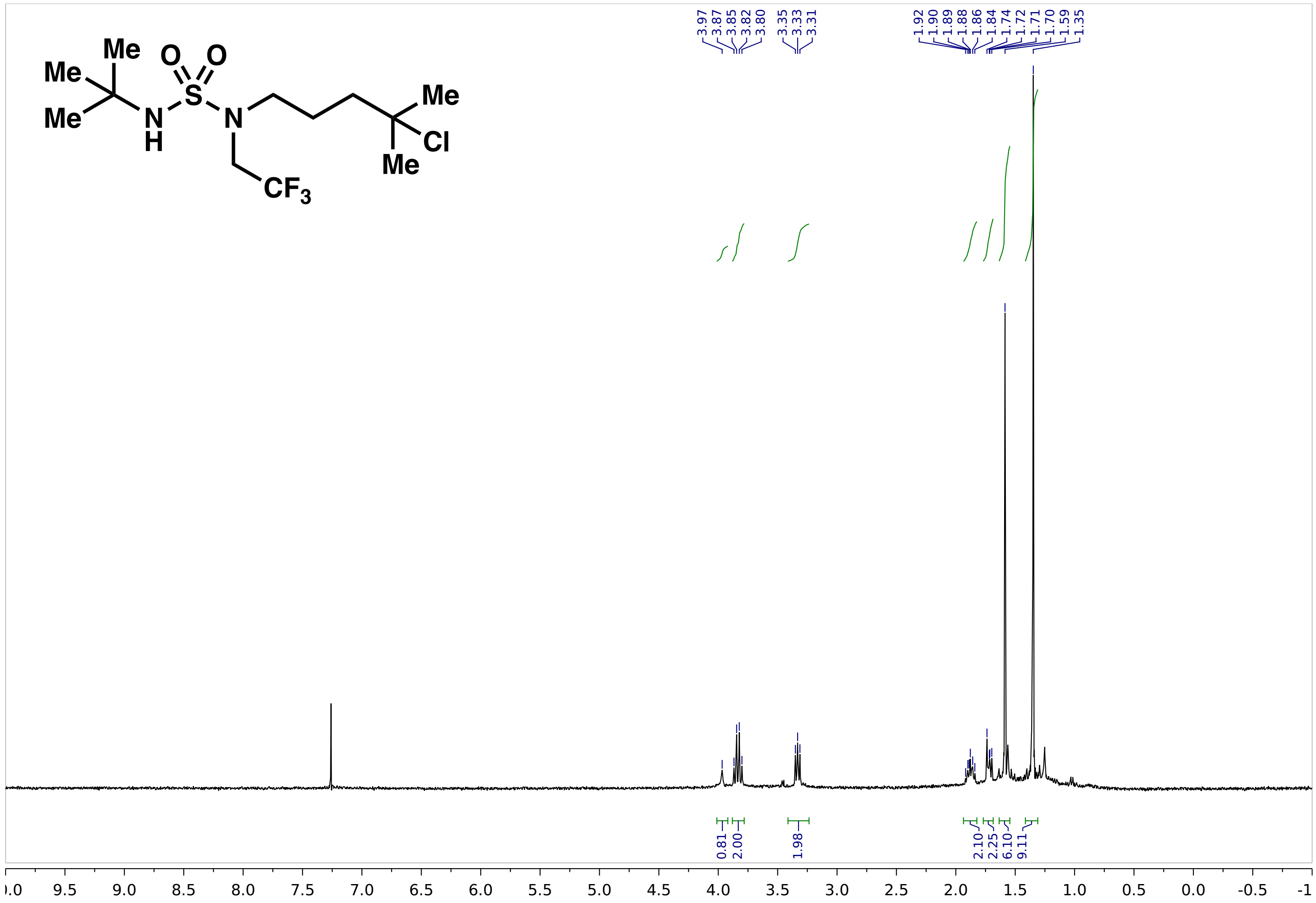

^{1}H NMR (400 MHz, $CDCl_3$) for *N*-*tert*-butyl-*N'*-(4-chloro-4-methylpent-1-yl)-*N'*-(2,2,2-trifluoroethyl)sulfamide (**7p**)

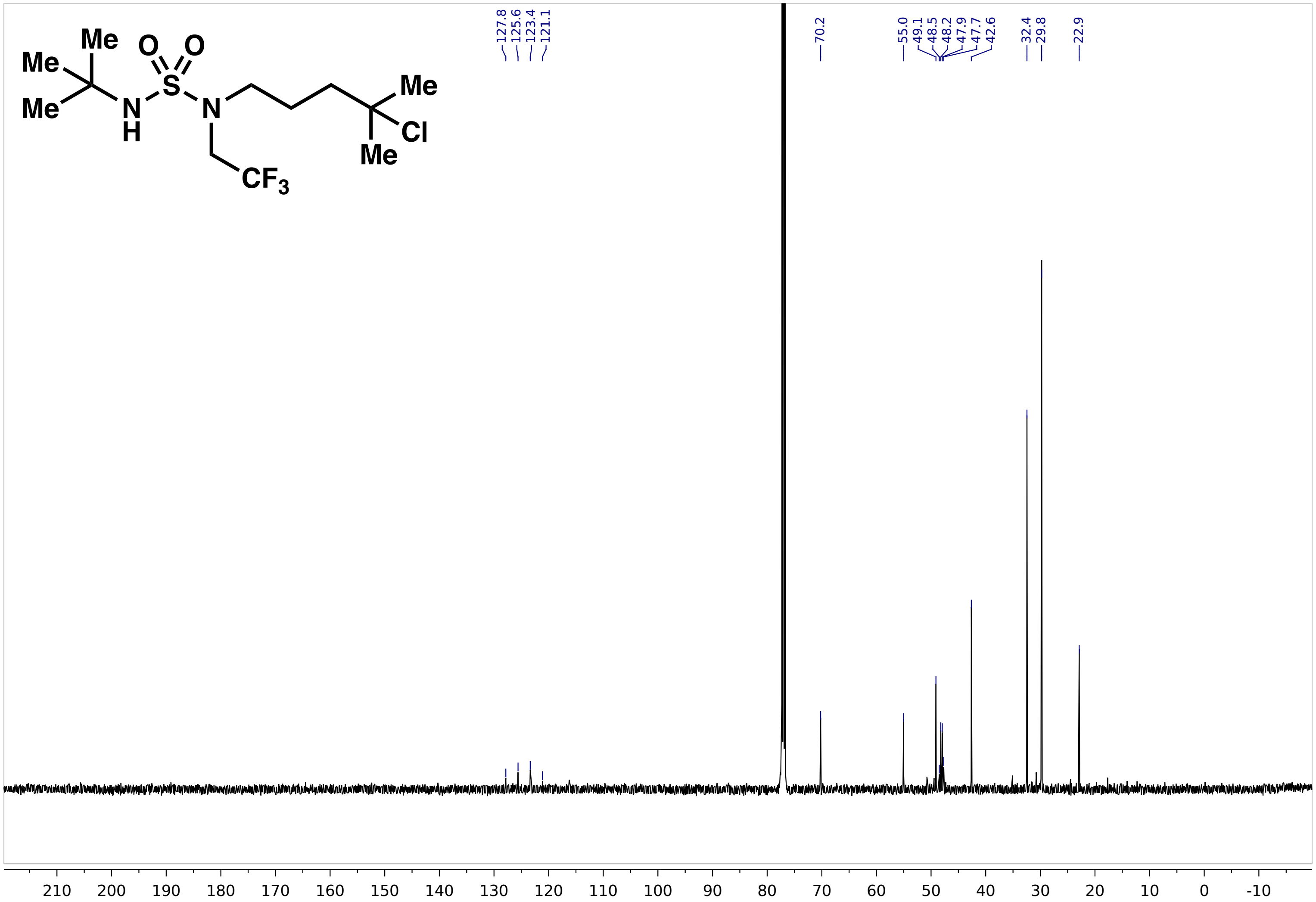

^{13}C NMR (126 MHz, $CDCl_3$) for *N*-*tert*-butyl-*N'*-(4-chloro-4-methylpent-1-yl)-*N'*-(2,2,2-trifluoroethyl)sulfamide (**7p**)

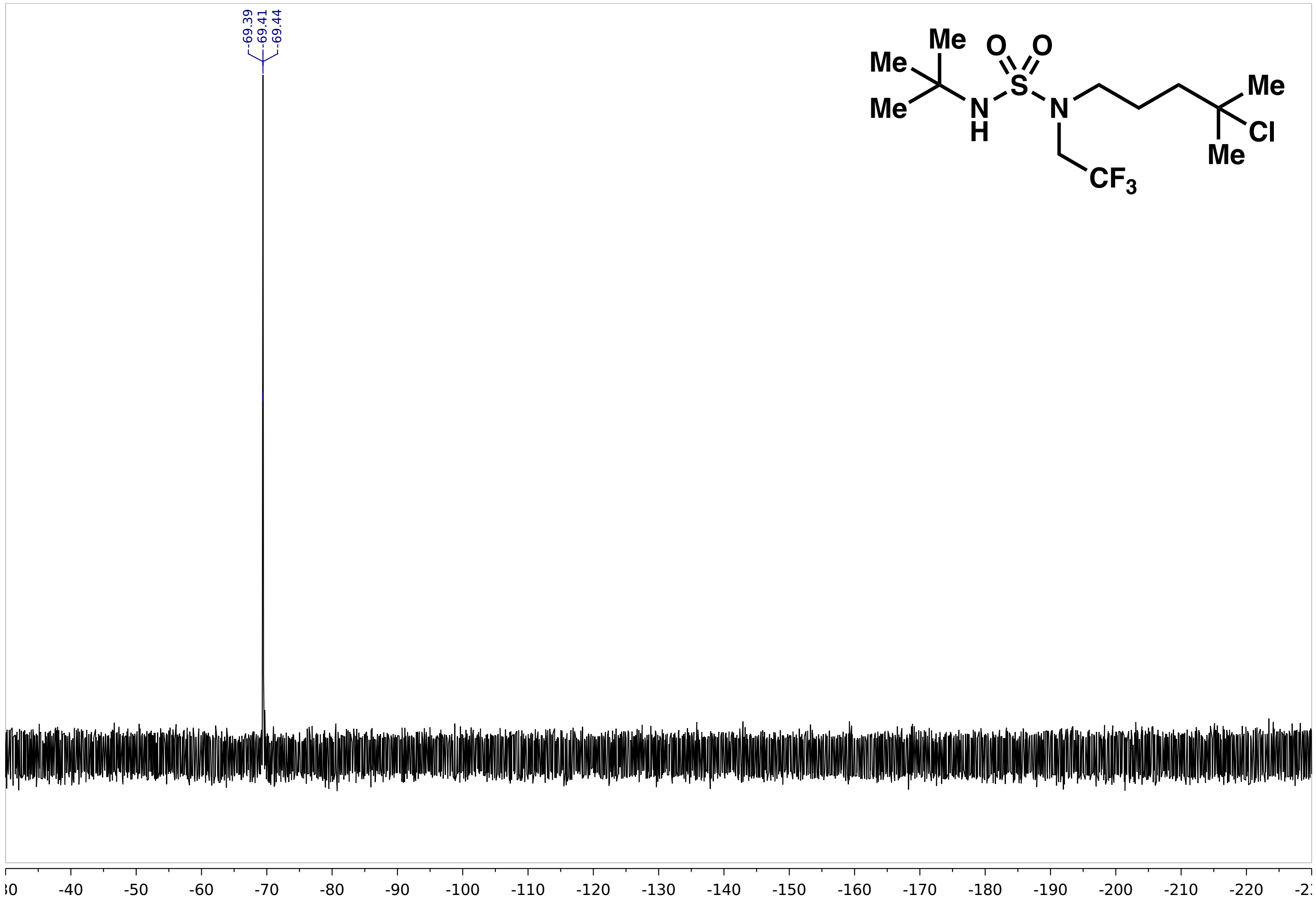

^{19}F NMR (376 MHz, $CDCl_3$) for *N*-*tert*-butyl-*N'*-(4-chloro-4-methylpent-1-yl)-*N'*-(2,2,2-trifluoroethyl)sulfamide (**7p**)

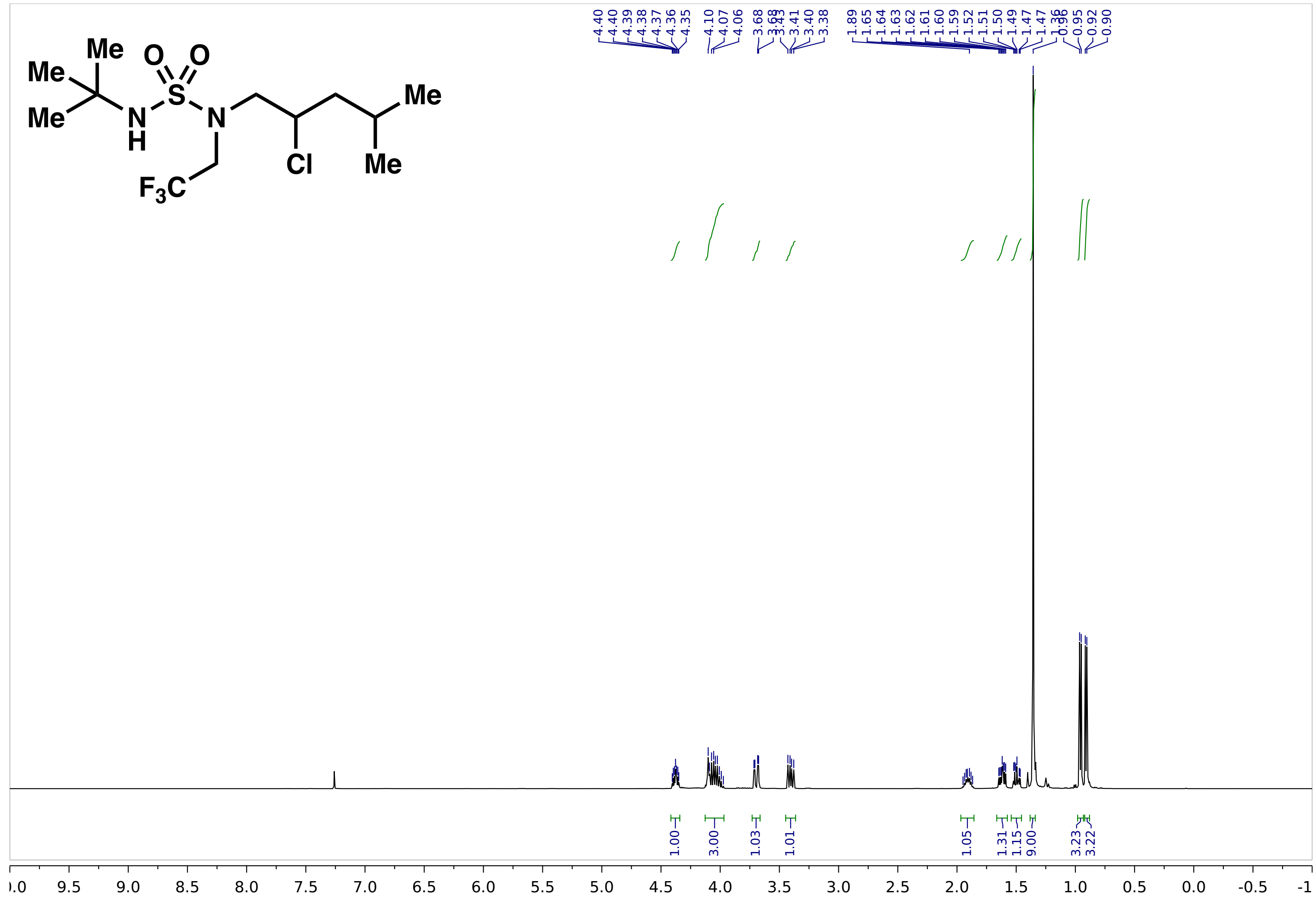

^{1}H NMR (500 MHz, $CDCl_3$) for *N*-*tert*-butyl-*N'*-(2-chloro-4-methylpent-1-yl)-*N'*-(2,2,2-trifluoroethyl)sulfamide (**S11p**)

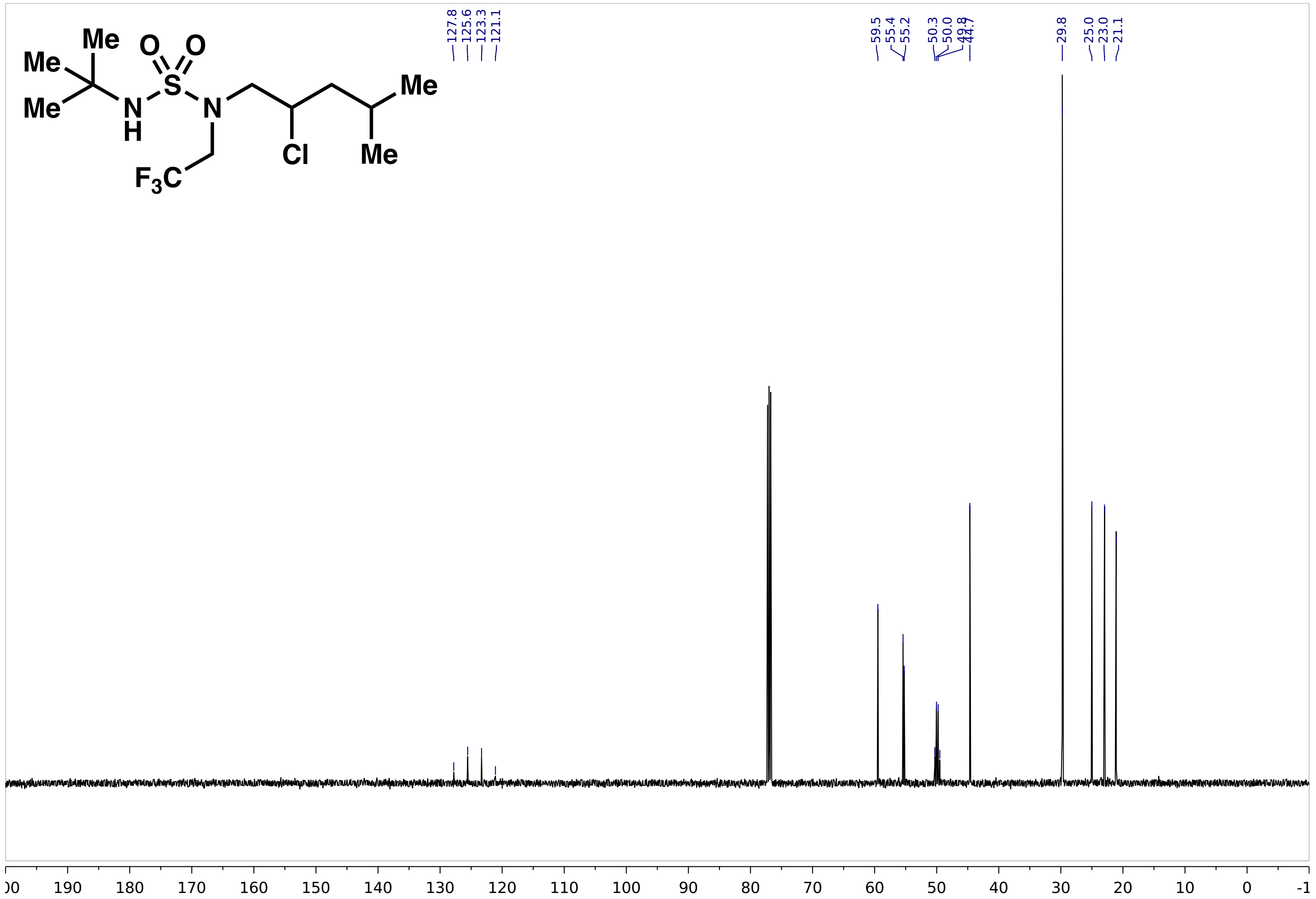

^{13}C NMR (126 MHz, $CDCl_3$) for *N*-*tert*-butyl-*N'*-(2-chloro-4-methylpent-1-yl)-*N'*-(2,2,2-trifluoroethyl)sulfamide (**S11p**)

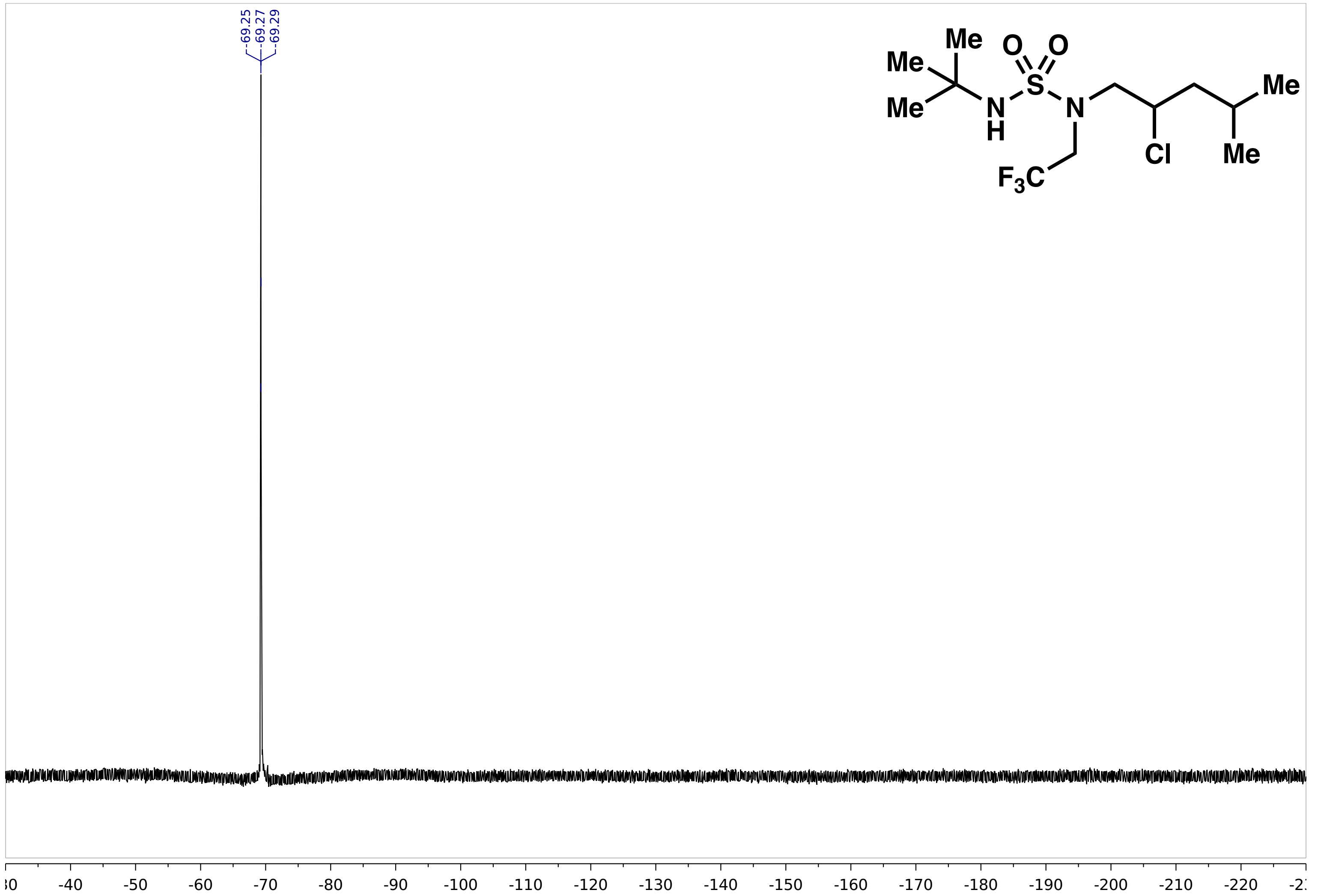

^{19}F NMR (376 MHz, $CDCl_3$) for *N*-*tert*-butyl-*N'*-(2-chloro-4-methylpent-1-yl)-*N'*-(2,2,2-trifluoroethyl)sulfamide (**S11p**)

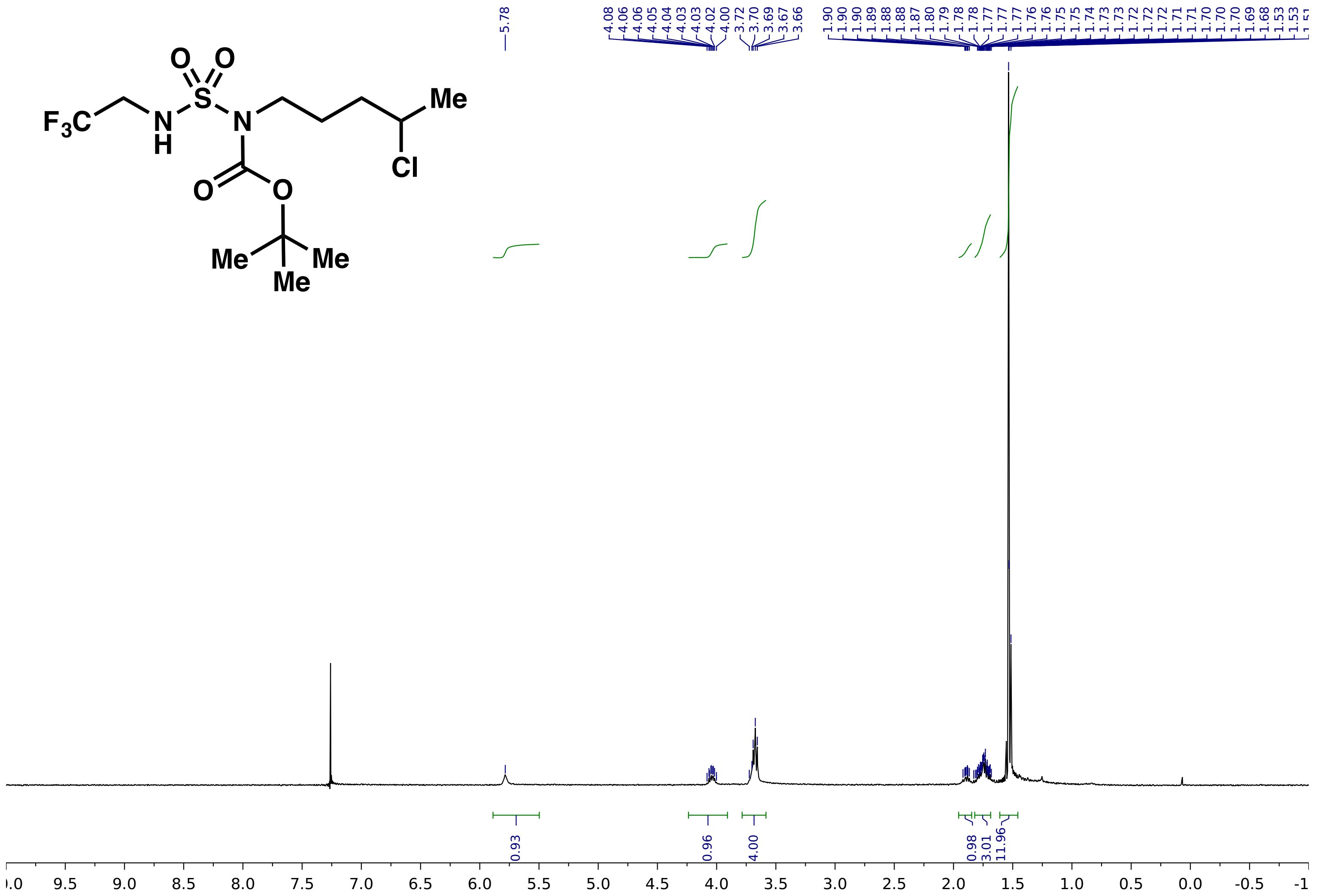

^{1}H NMR (400 MHz, $CDCl_3$) for *N*-*tert*-butoxycarbonyl-*N*-(4-chloropent-1-yl)-*N’*-(2,2,2-trfiluoroethyl)sulfamide (**S12v**)

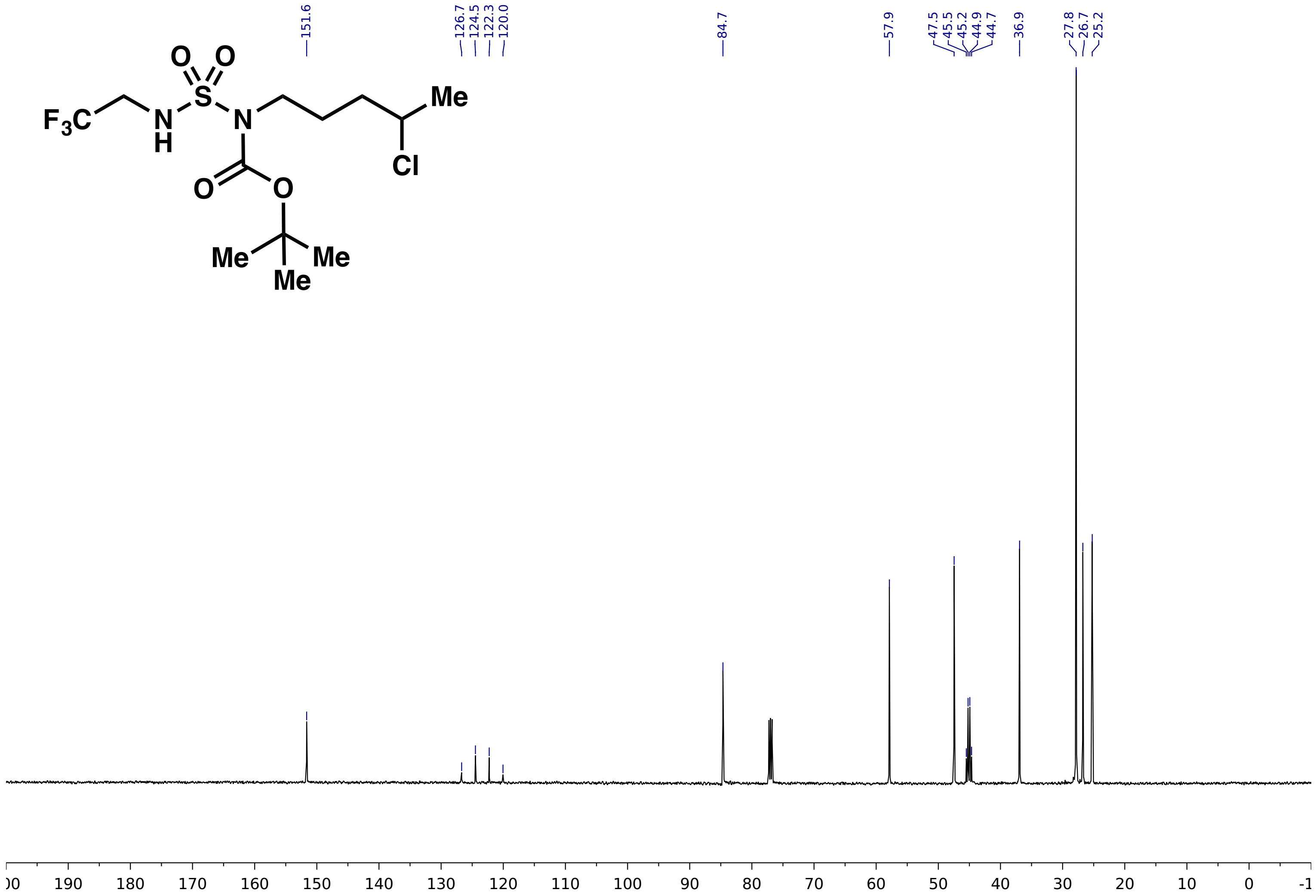

^{13}C NMR (126 MHz, $CDCl_3$) for *N*-*tert*-butoxycarbonyl-*N*-(4-chloropent-1-yl)-*N'*-(2,2,2-trfiluoroethyl)sulfamide (**S12v**)

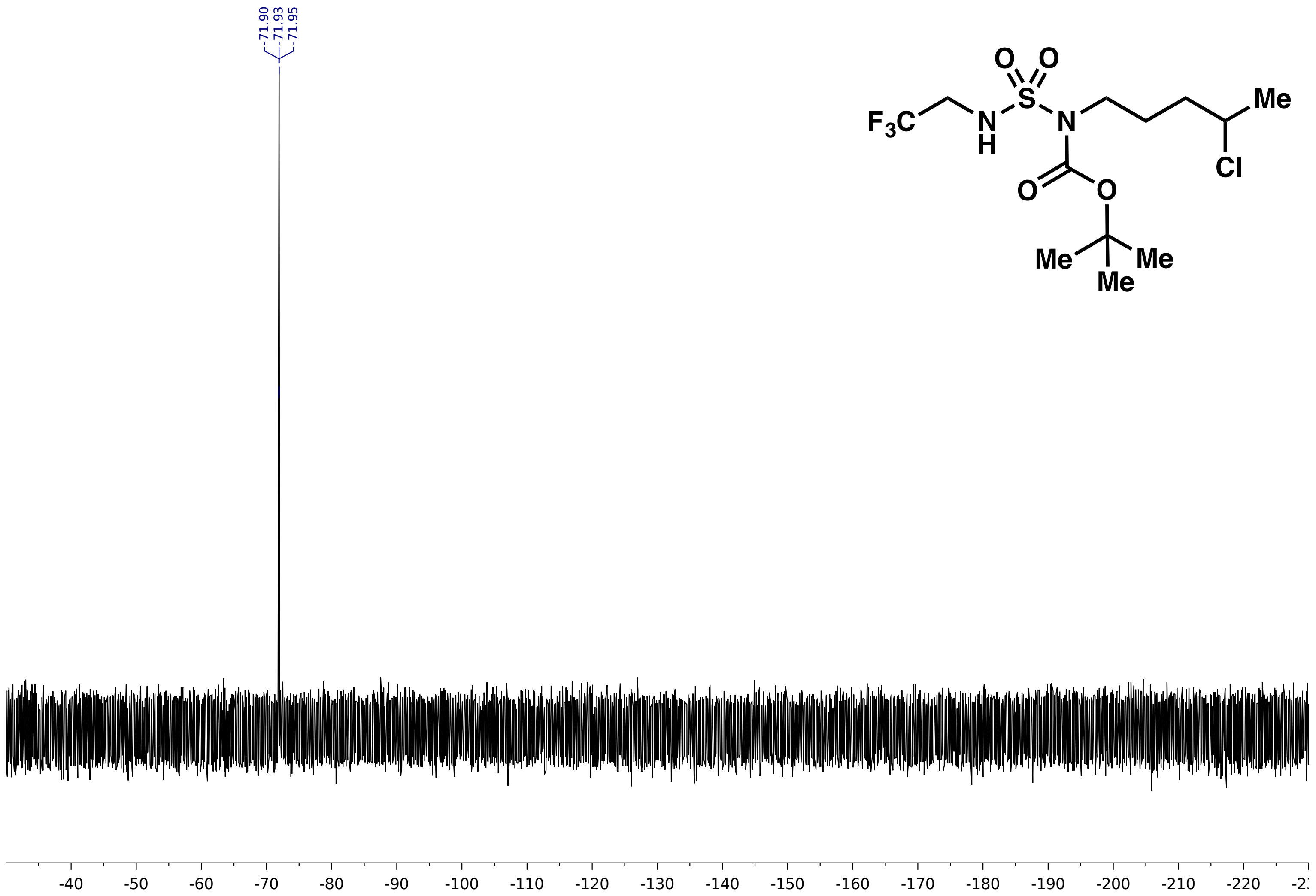

^{19}F NMR (376 MHz, $CDCl_3$) for *N*-*tert*-butoxycarbonyl-*N*-(4-chloropent-1-yl)-*N'*-(2,2,2-trfiluoroethyl)sulfamide (**S12v**)

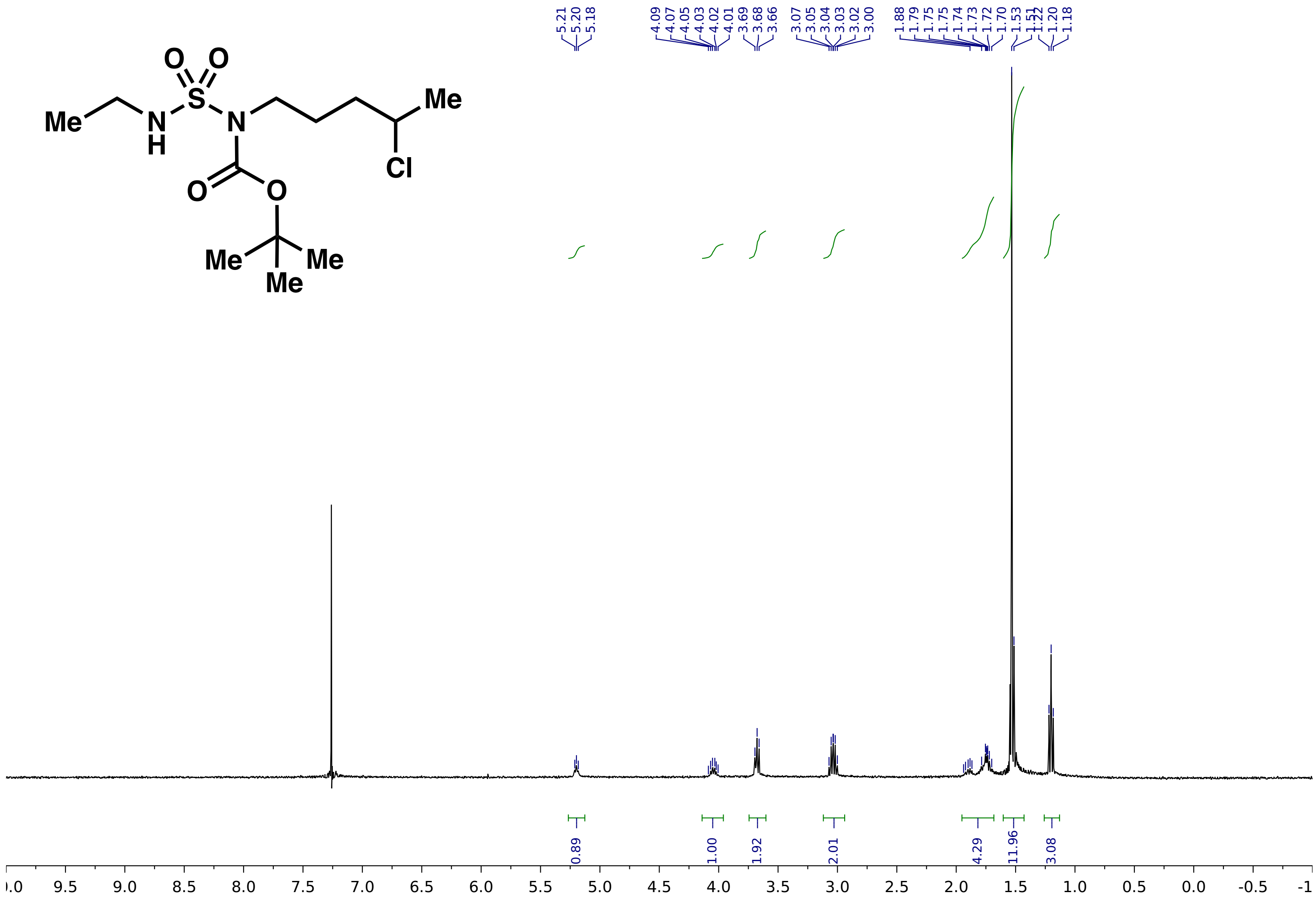

^{1}H NMR (400 MHz, $CDCl_3$) for *N*-*tert*-butoxycarbonyl-*N*-(4-chloropent-1-yl)-*N'*-ethylsulfamide (**S12w**)

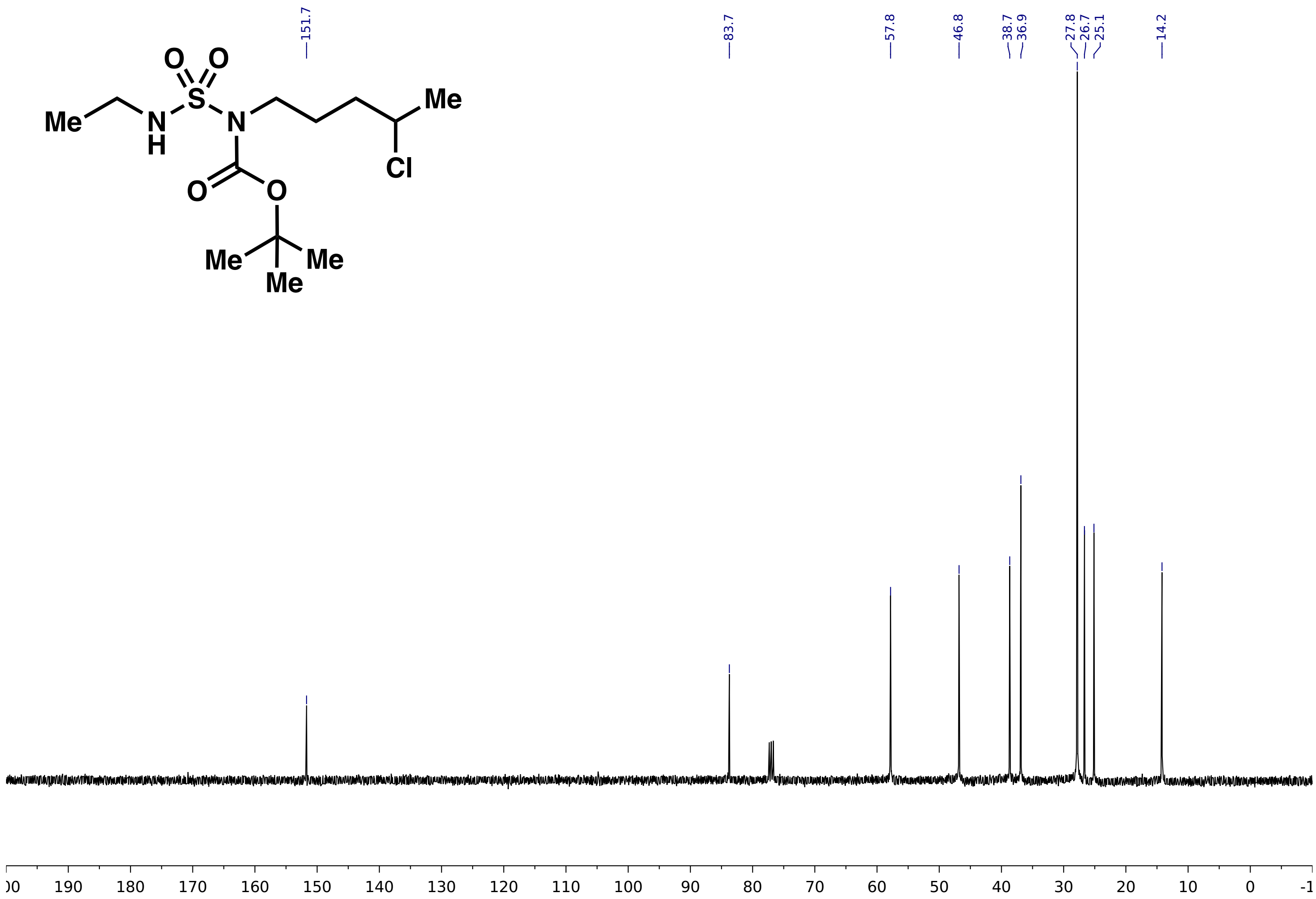

^{13}C NMR (126 MHz, $CDCl_3$) for *N*-*tert*-butoxycarbonyl-*N*-(4-chloropent-1-yl)-*N'*-ethylsulfamide (**S12w**)

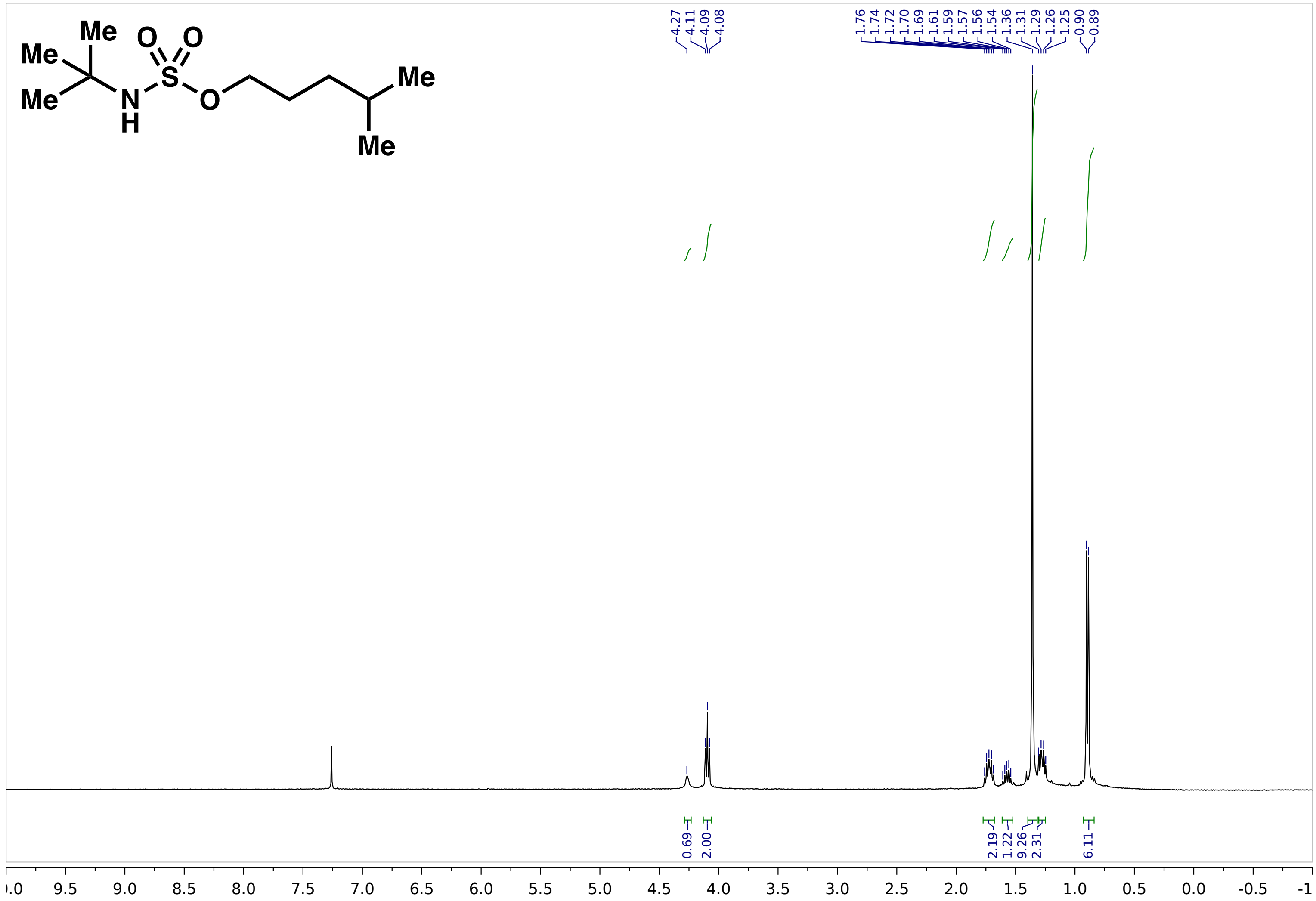

^{1}H NMR (400 MHz, $CDCl_3$) for 4-methylpentyl *tert*-butylsulfamate (**S13a**)

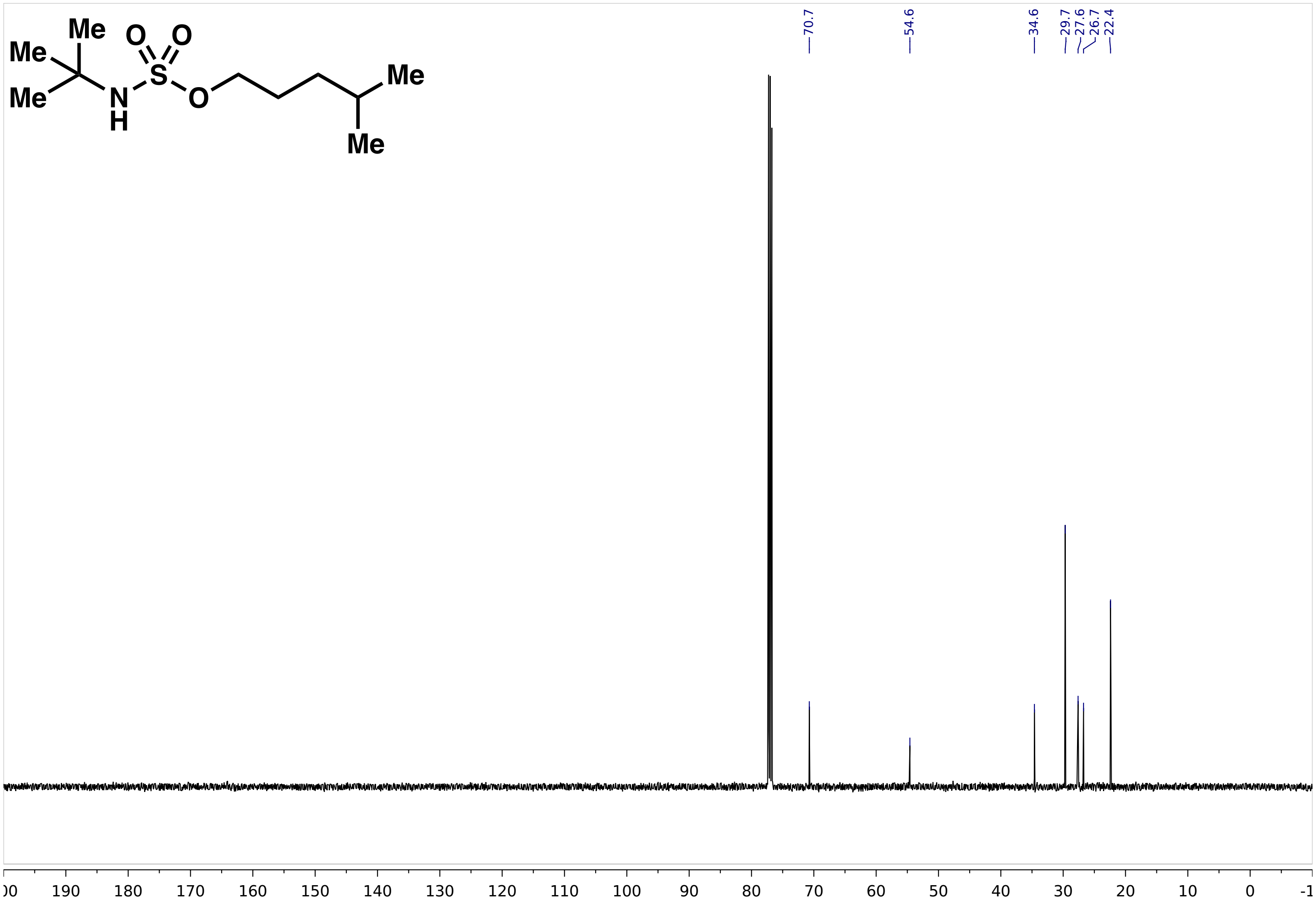

^{13}C NMR (126 MHz, $CDCl_3$) for 4-methylpentyl *tert*-butylsulfamate (**S13a**)

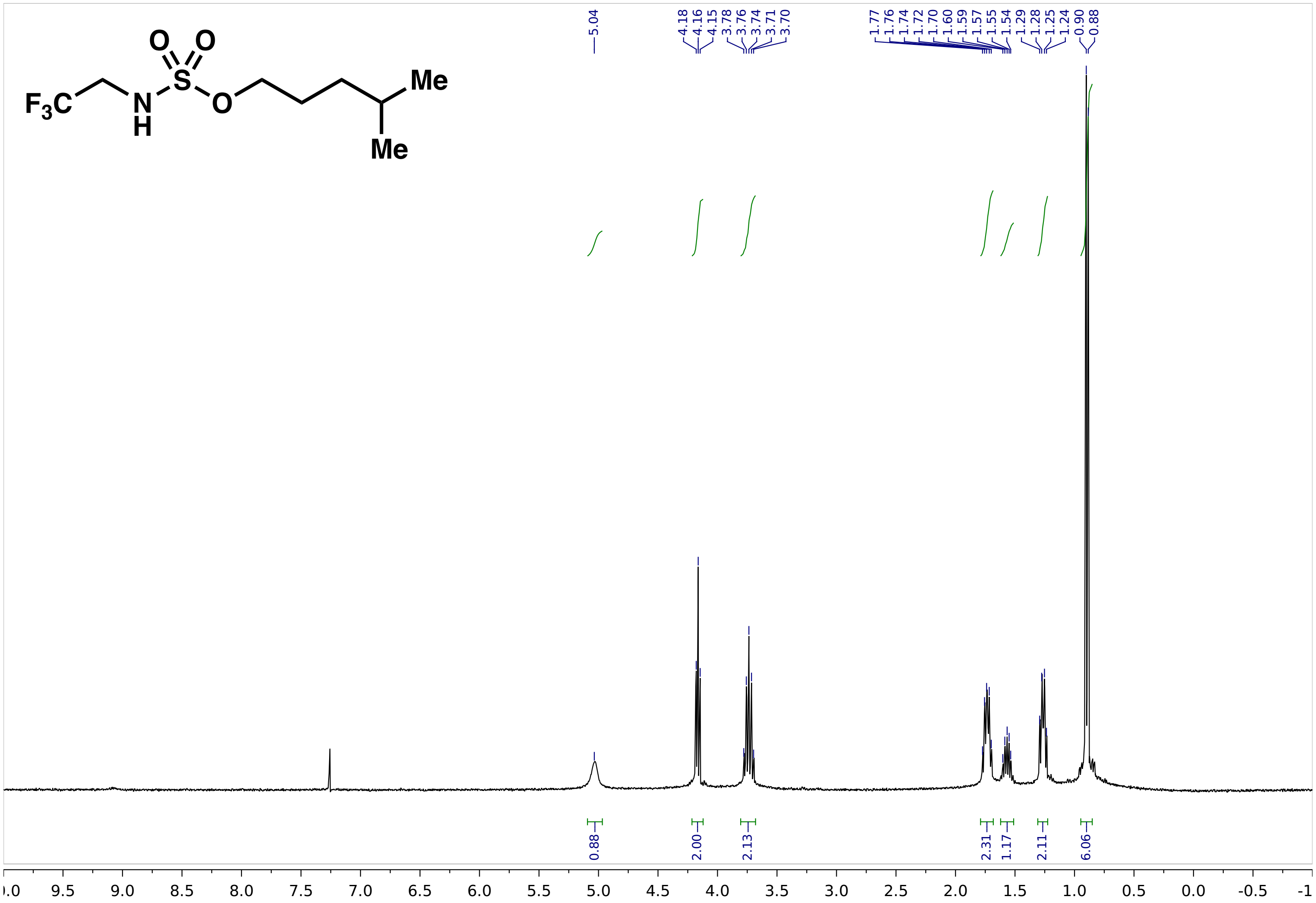

^{1}H NMR (400 MHz, $CDCl_3$) for 4-methylpentyl (2,2,2-trifluoroethyl)sulfamate (**S13b**)

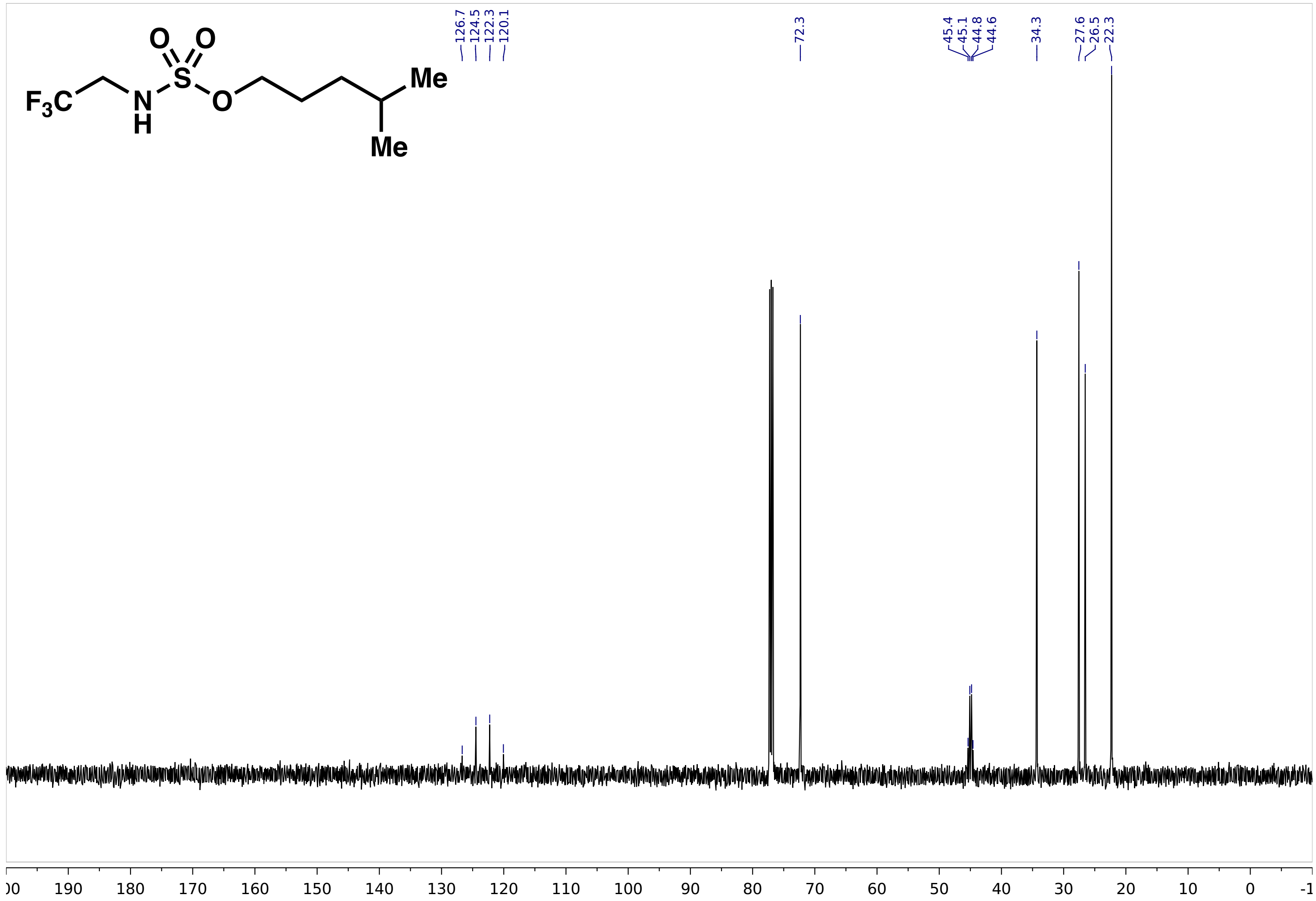

^{13}C NMR (126 MHz, $CDCl_3$) for 4-methylpentyl (2,2,2-trifluoroethyl)sulfamate (**S13b**)

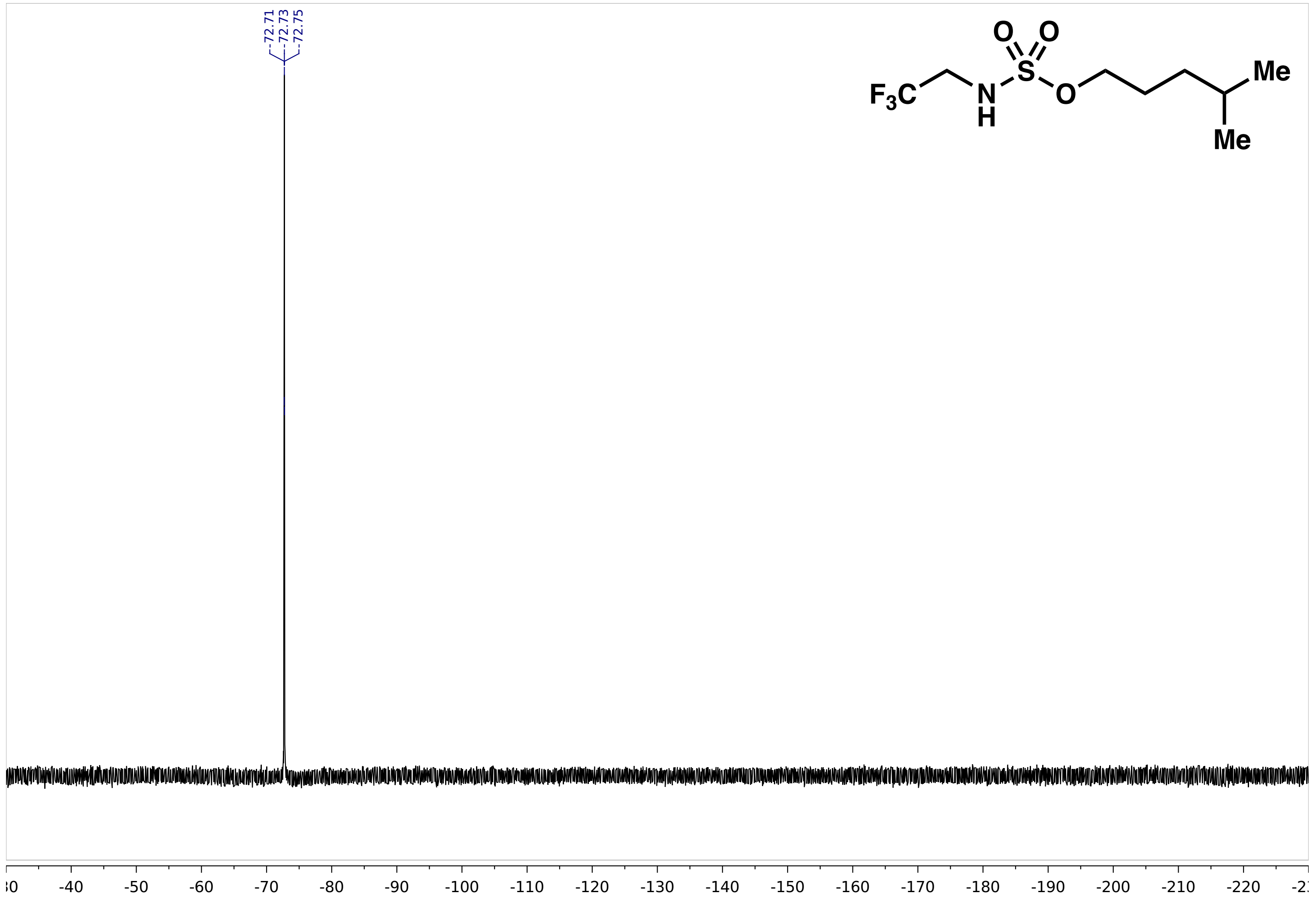

^{19}F NMR (376 MHz, $CDCl_3$) for 4-methylpentyl (2,2,2-trifluoroethyl)sulfamate (**S13b**)

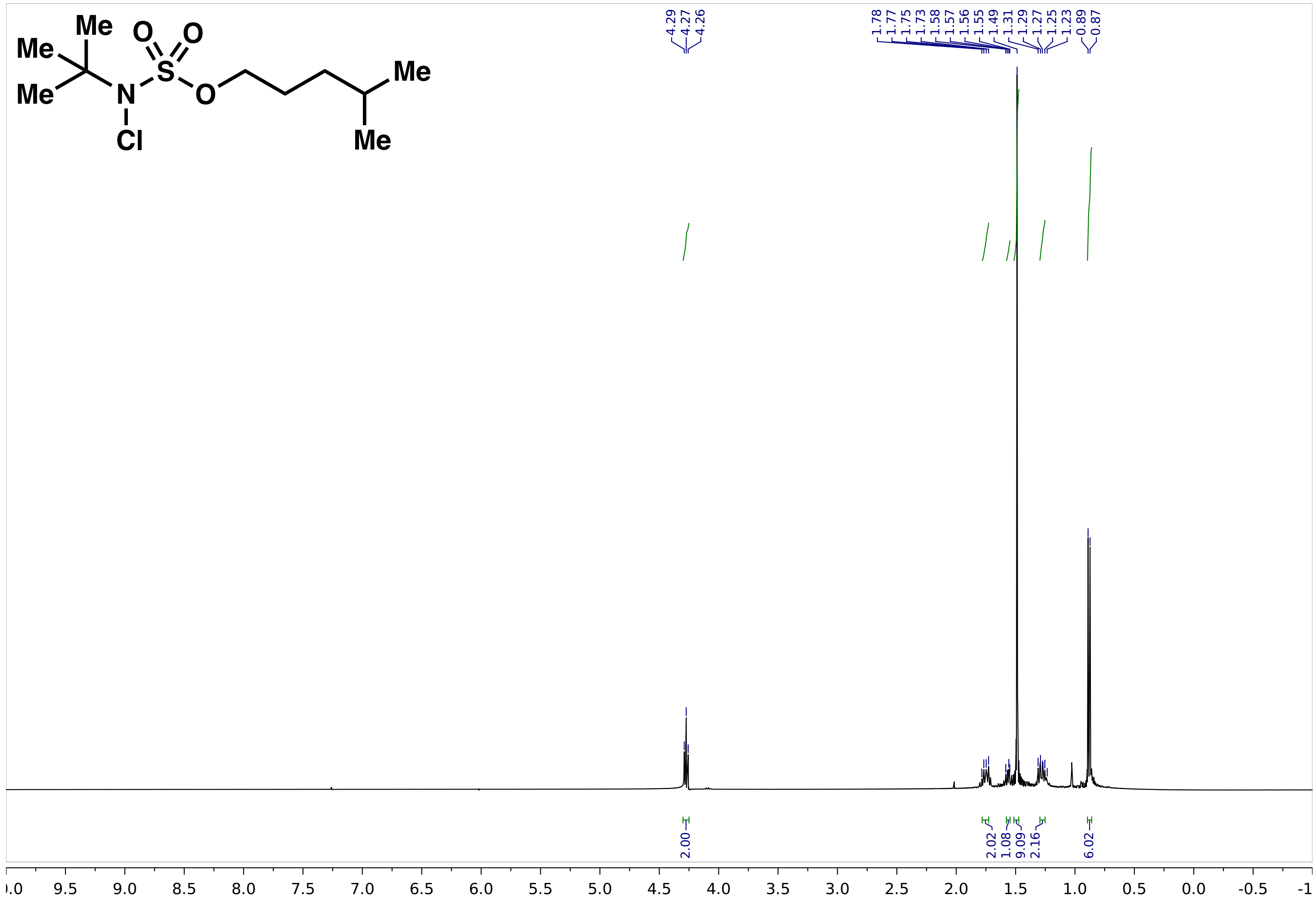

^{1}H NMR (400 MHz, $CDCl_3$) for 4-methylpentyl *tert*-butylchlorosulfamate ester (**9a**)

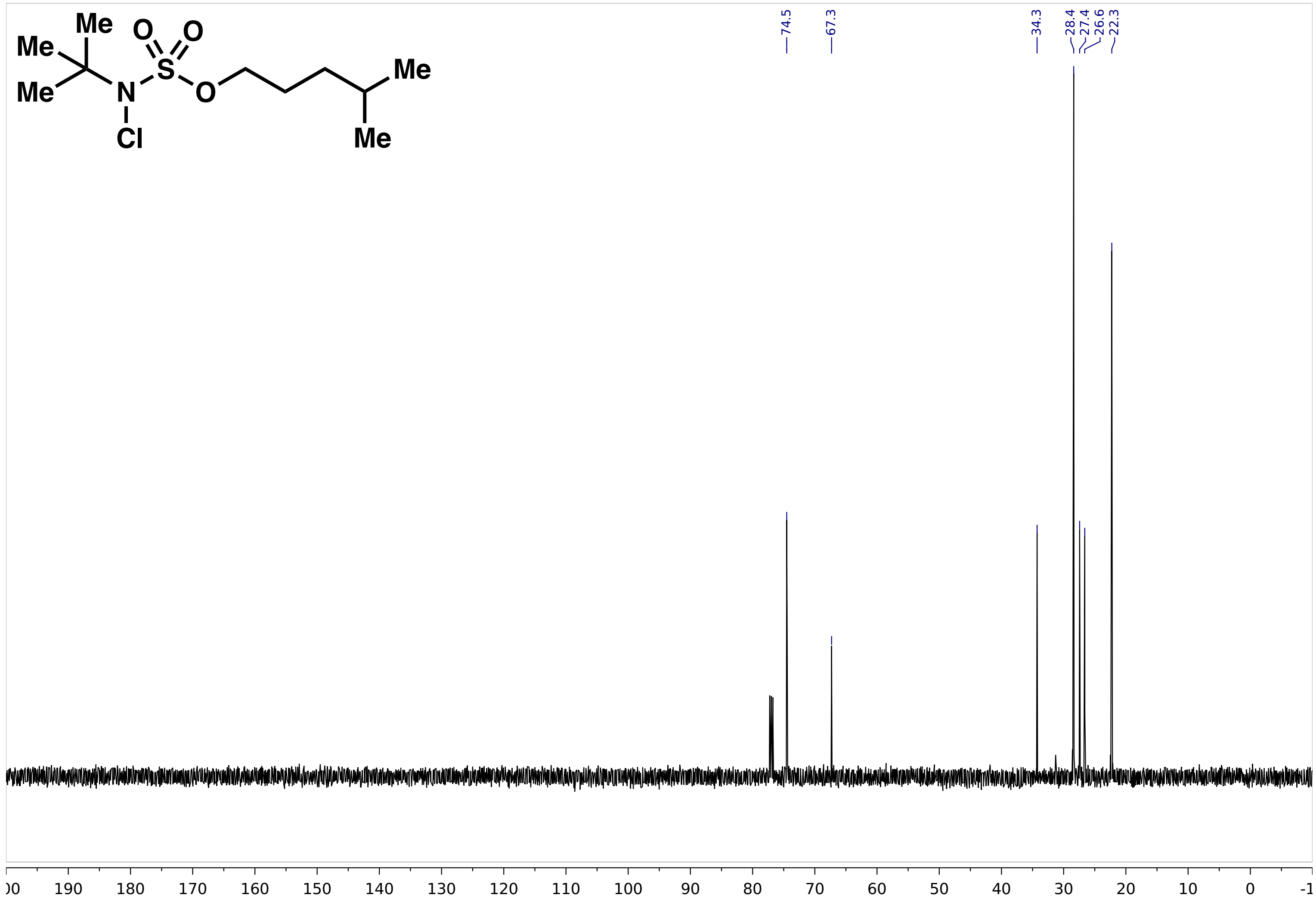

^{13}C NMR (126 MHz, $CDCl_3$) for 4-methylpentyl *tert*-butylchlorosulfamate ester (**9a**)

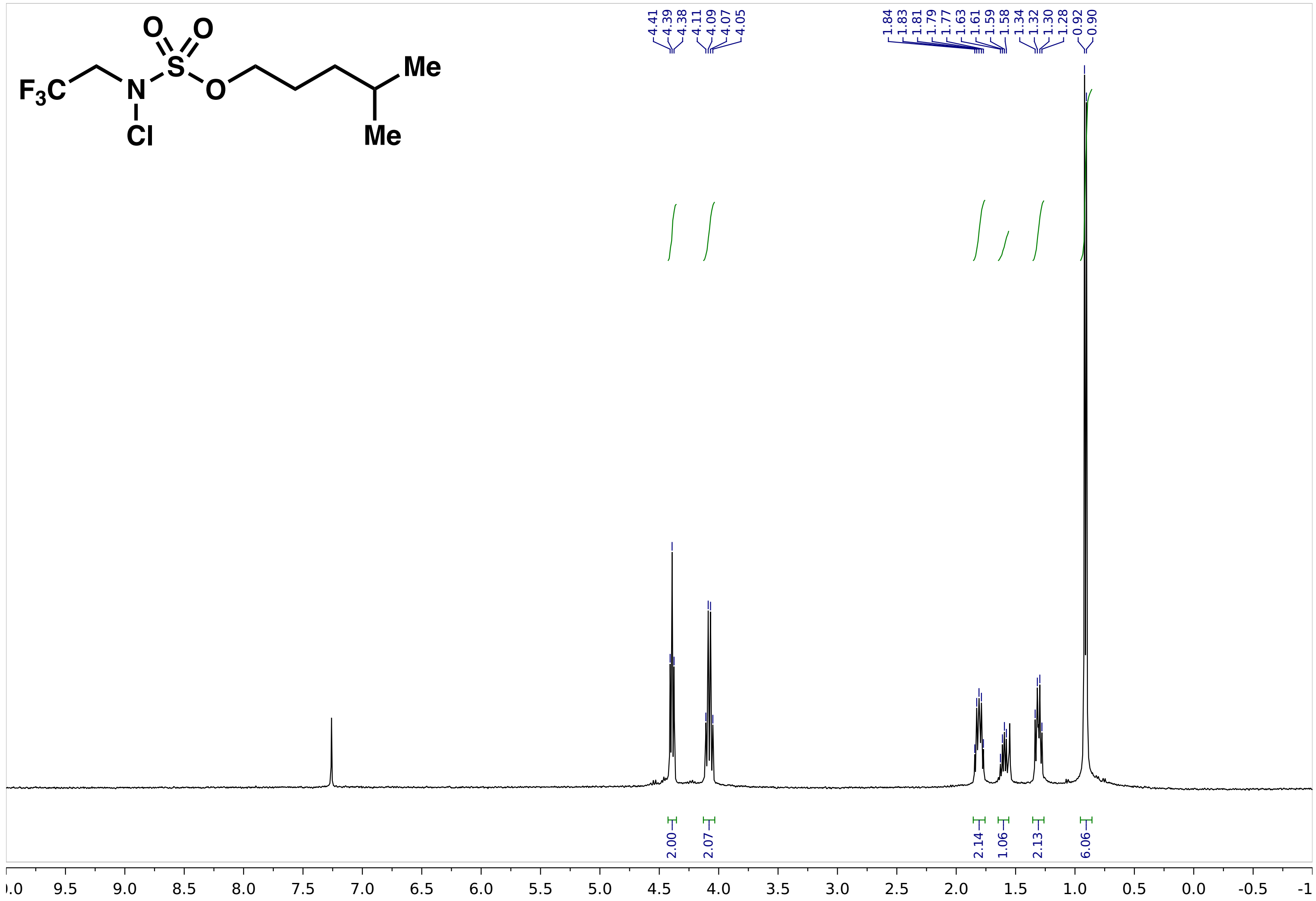

^{1}H NMR (400 MHz, $CDCl_3$) for 4-methylpentyl (2,2,2-trifluoroethyl)chlorosulfamate ester (**9b**)

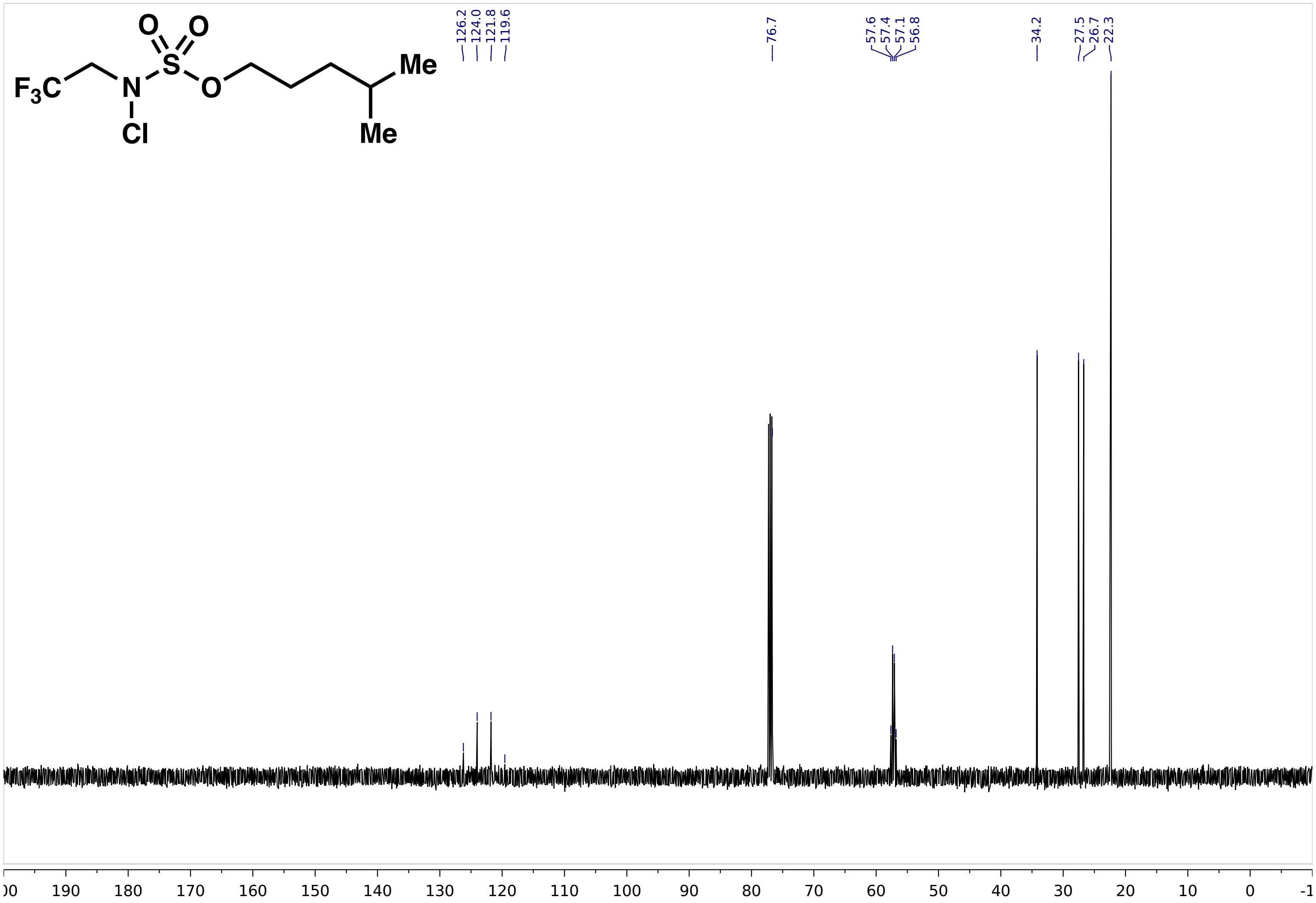

^{13}C NMR (126 MHz, $CDCl_3$) for 4-methylpentyl (2,2,2-trifluoroethyl)chlorosulfamate ester (**9b**)

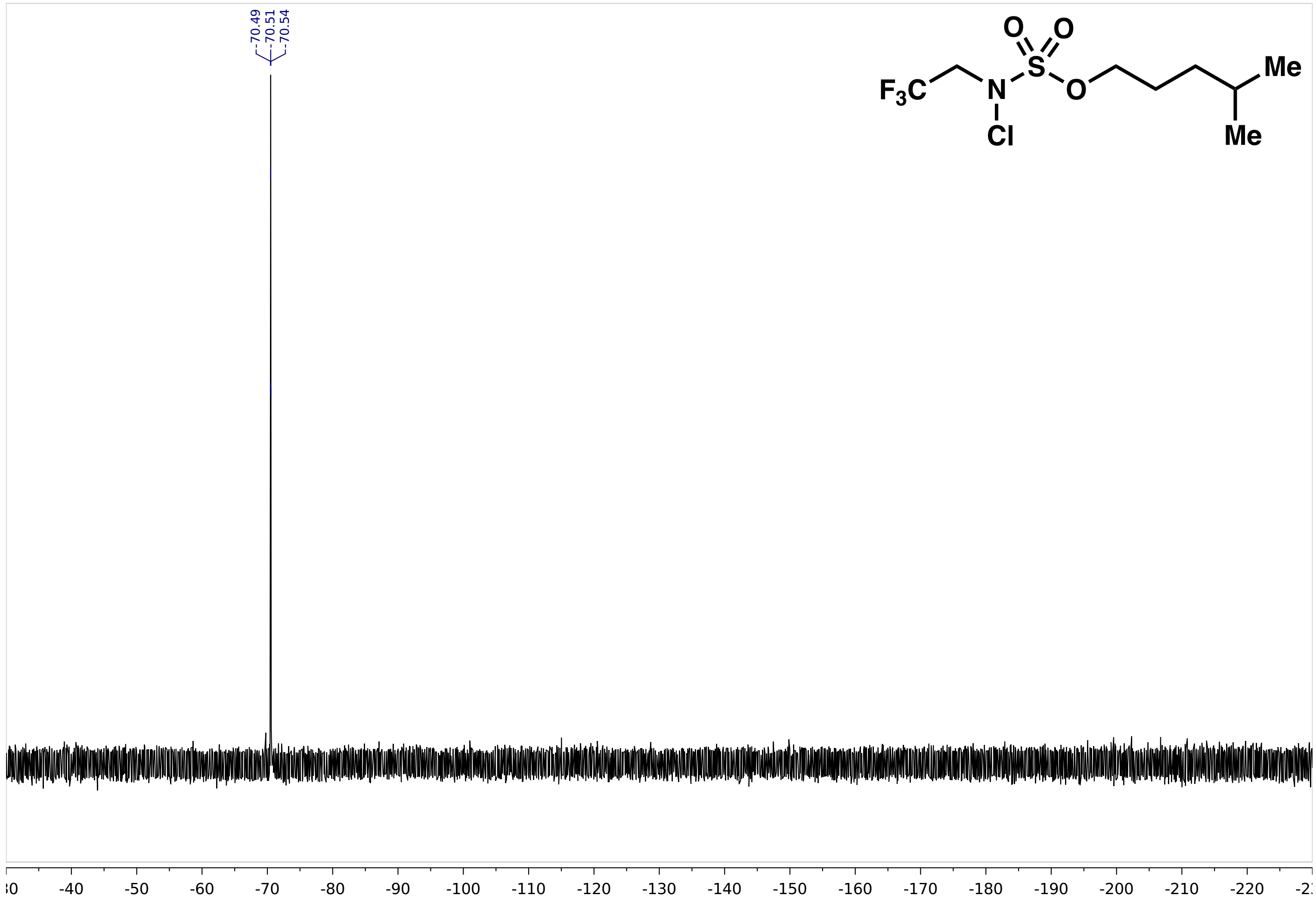

^{19}F NMR (376 MHz, $CDCl_3$) for 4-methylpentyl (2,2,2-trifluoroethyl)chlorosulfamate ester (**9b**)

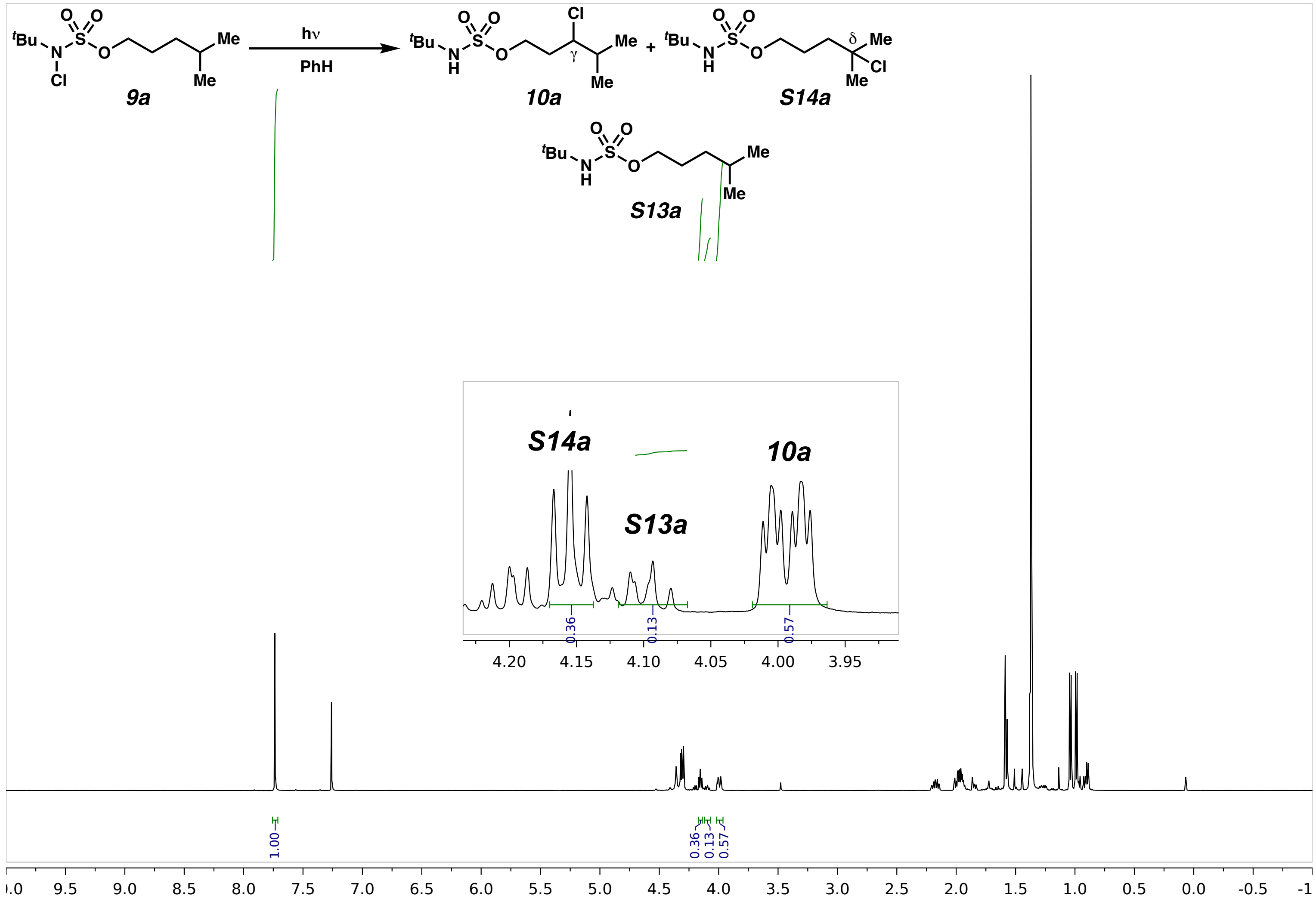

Crude ^{1}H NMR (500 MHz, $CDCl_3$, 25s relaxation delay) for chlorine-transfer reaction of *N*-chlorosulfamate **9a**

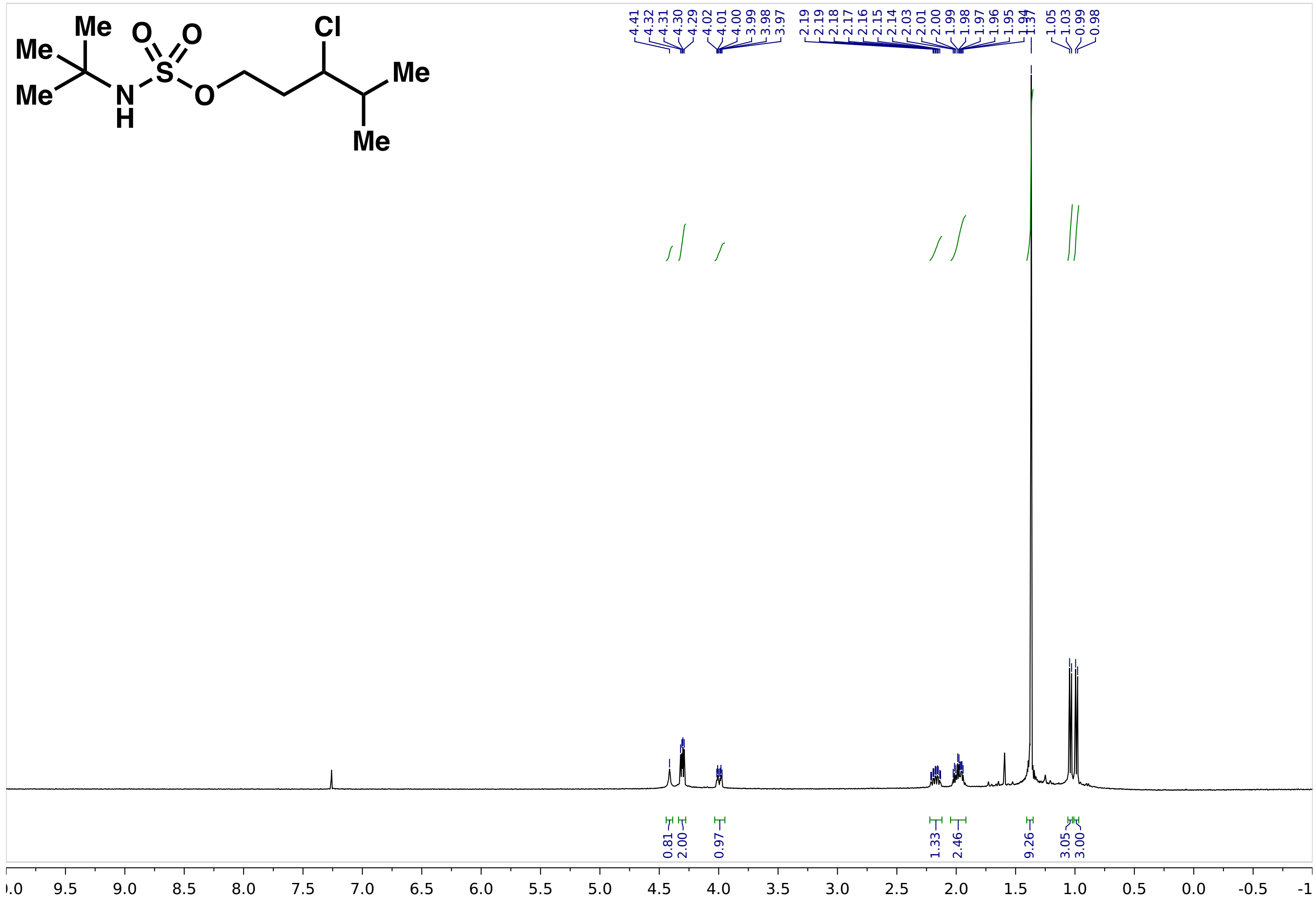

^{1}H NMR (400 MHz, $CDCl_3$) for 3-chloro-4-methylpentyl *tert*-butylsulfamate (**10a**)

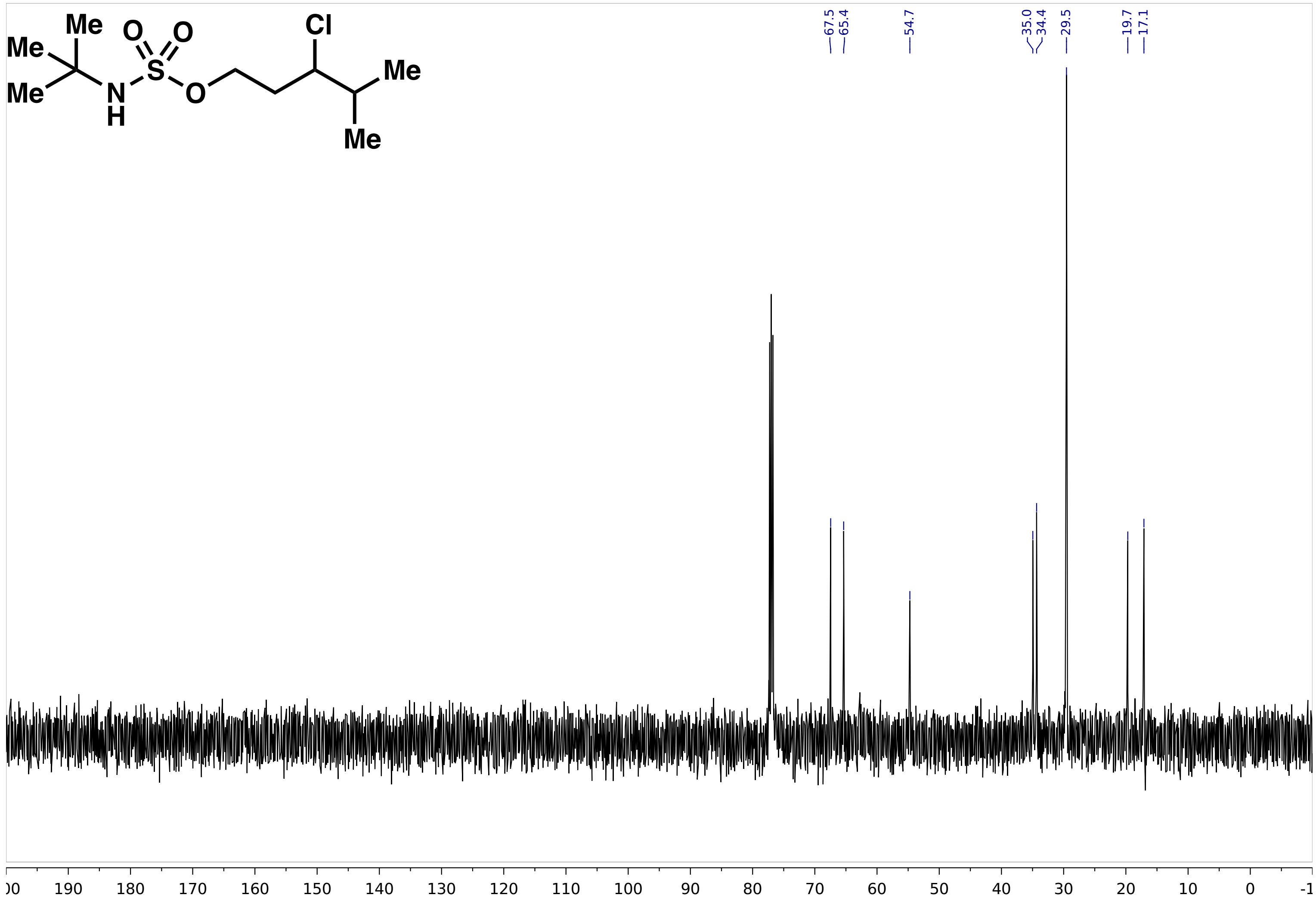

^{13}C NMR (126 MHz, $CDCl_3$) for 3-chloro-4-methylpentyl *tert*-butylsulfamate (**10a**)

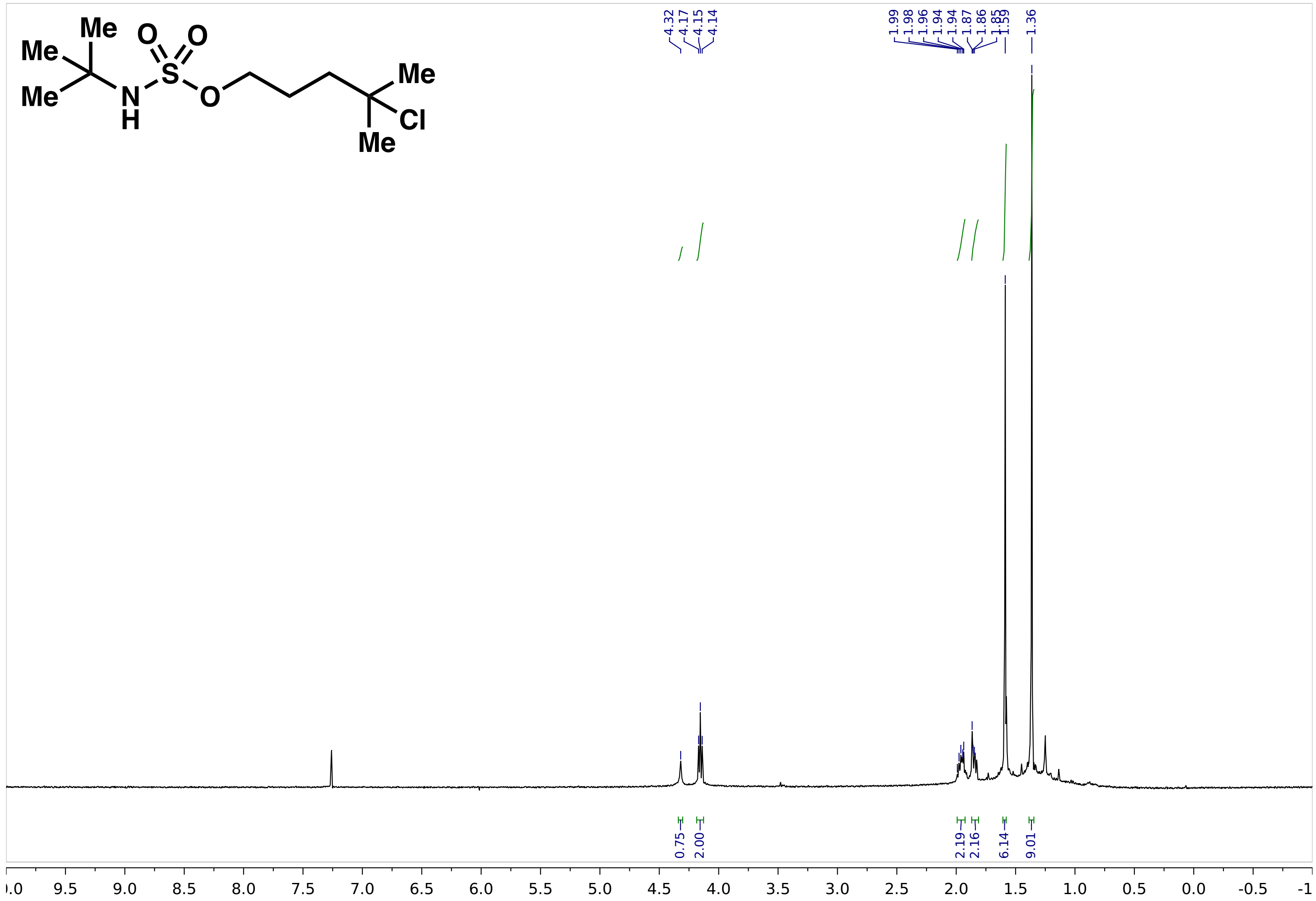

^{1}H NMR (400 MHz, $CDCl_3$) for 4-chloro-4-methylpentyl *tert*-butylsulfamate (**S14a**)

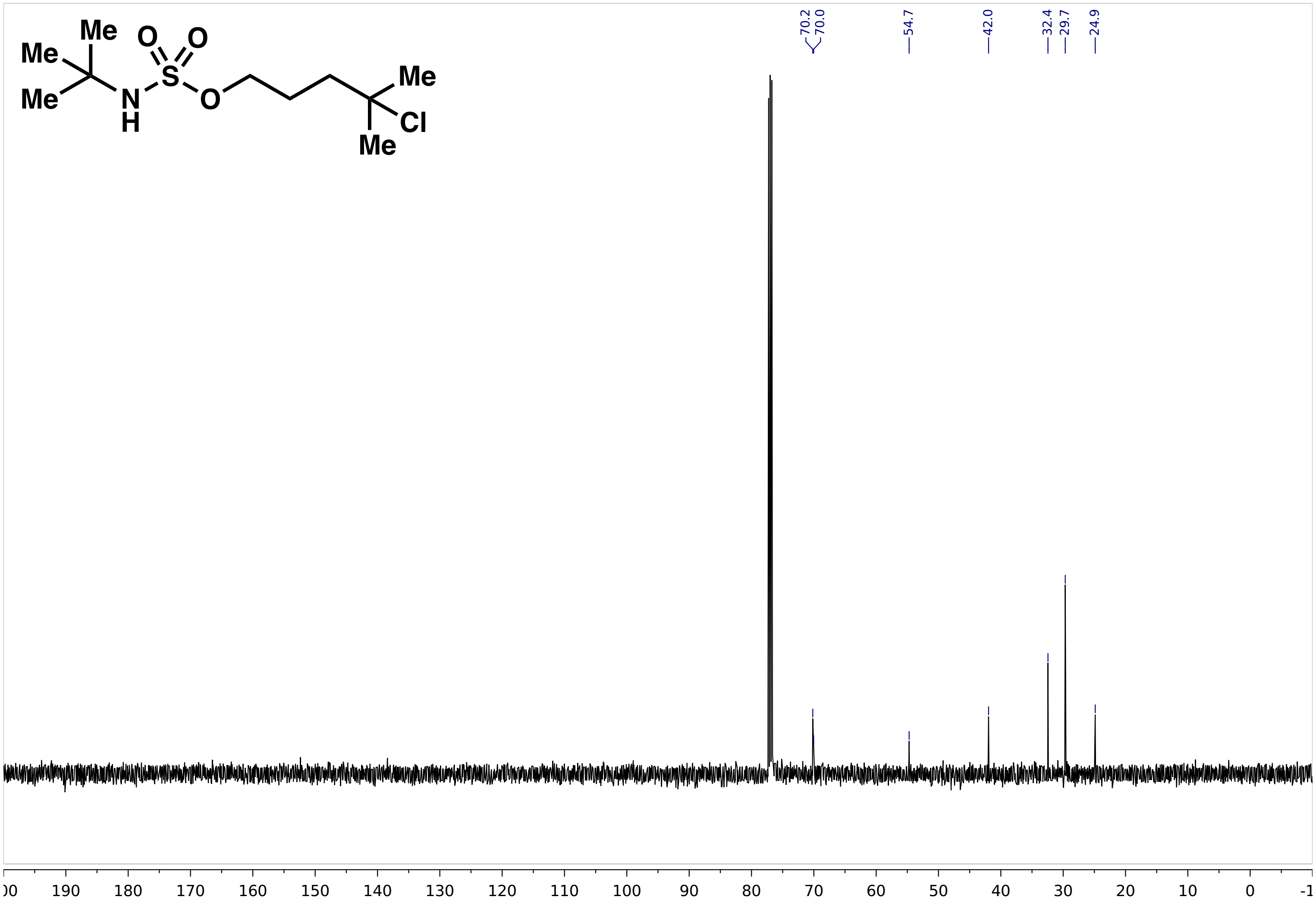

^{13}C NMR (126 MHz, $CDCl_3$) for 4-chloro-4-methylpentyl *tert*-butylsulfamate (**S14a**)

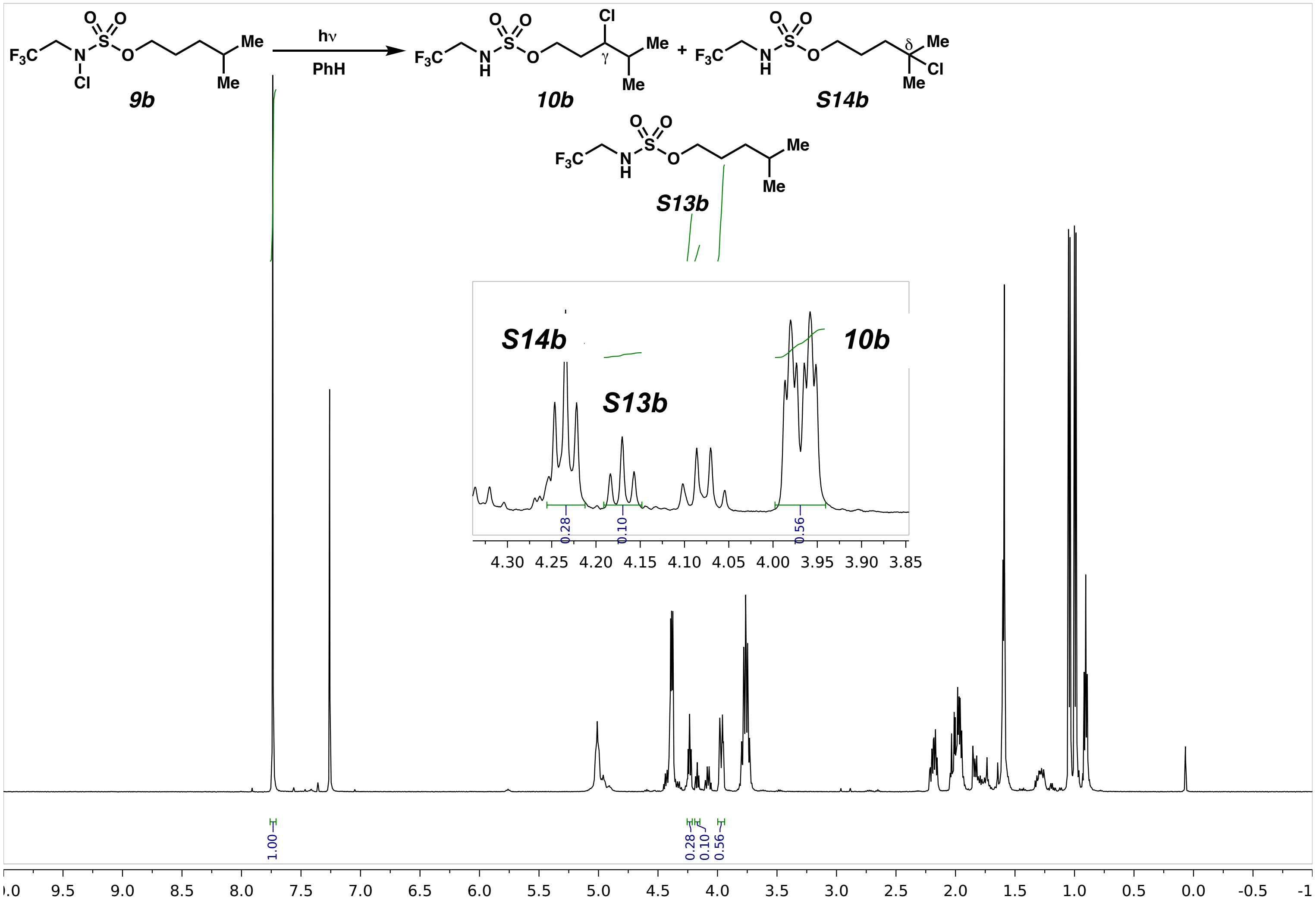

Crude ^{1}H NMR (500 MHz, $CDCl_3$, 25s relaxation delay) for chlorine-transfer reaction of *N*-chlorosulfamate **9b**

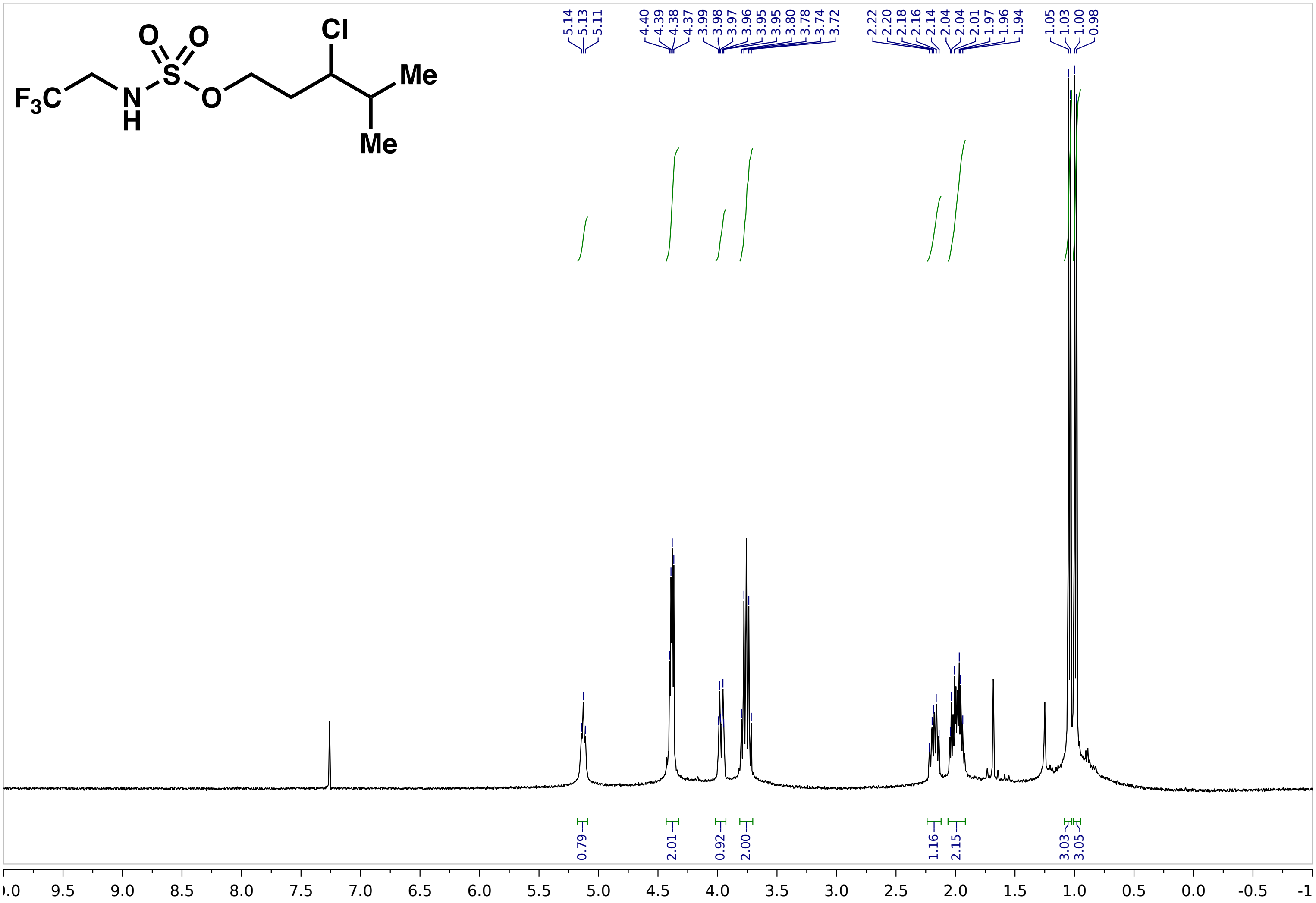

^{1}H NMR (400 MHz, $CDCl_3$) for 3-chloro-4-methylpentyl (2,2,2-trifluoroethyl)sulfamate (**10b**)

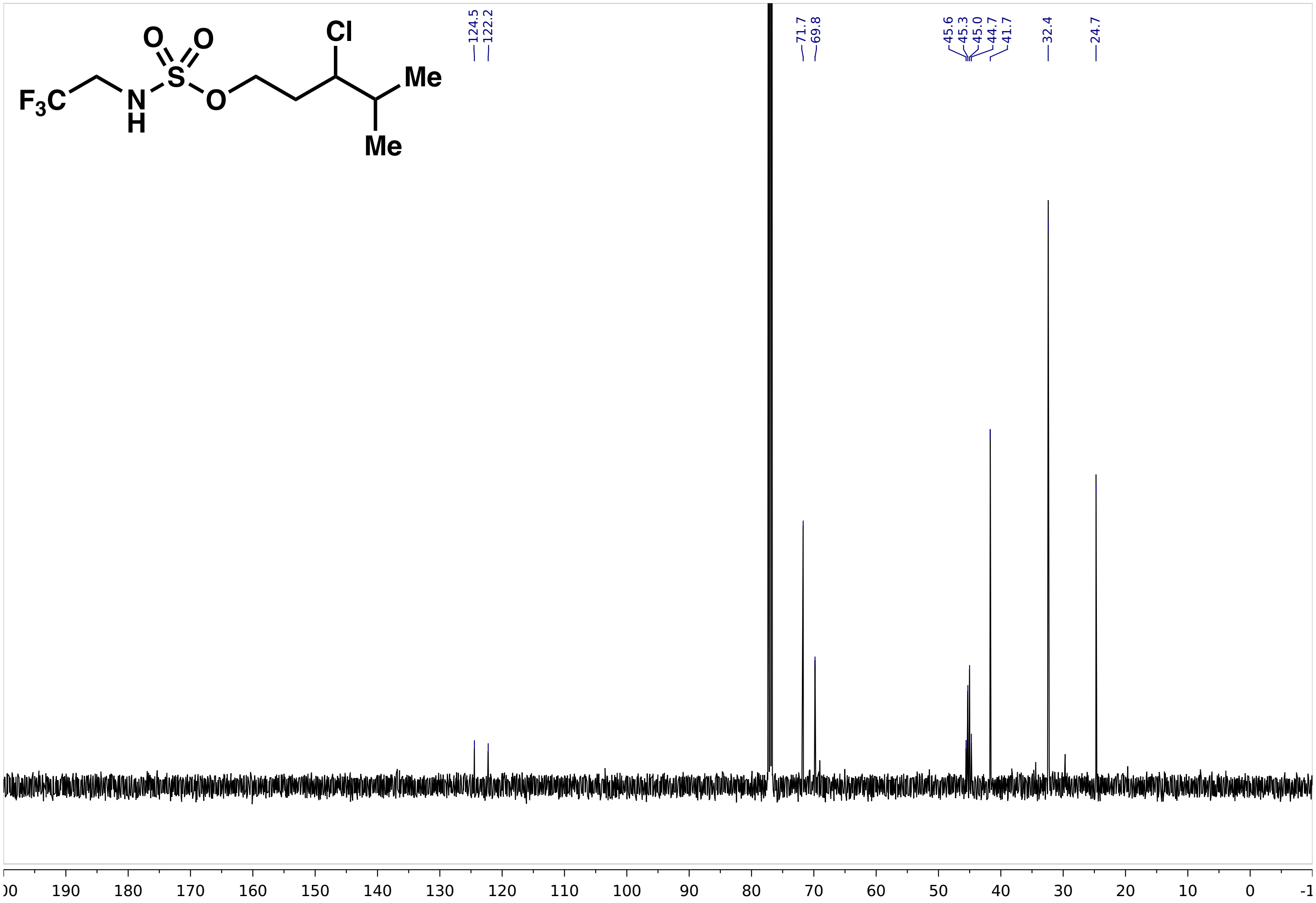

^{13}C NMR (126 MHz, $CDCl_3$) for 3-chloro-4-methylpentyl (2,2,2-trifluoroethyl)sulfamate (**10b**)

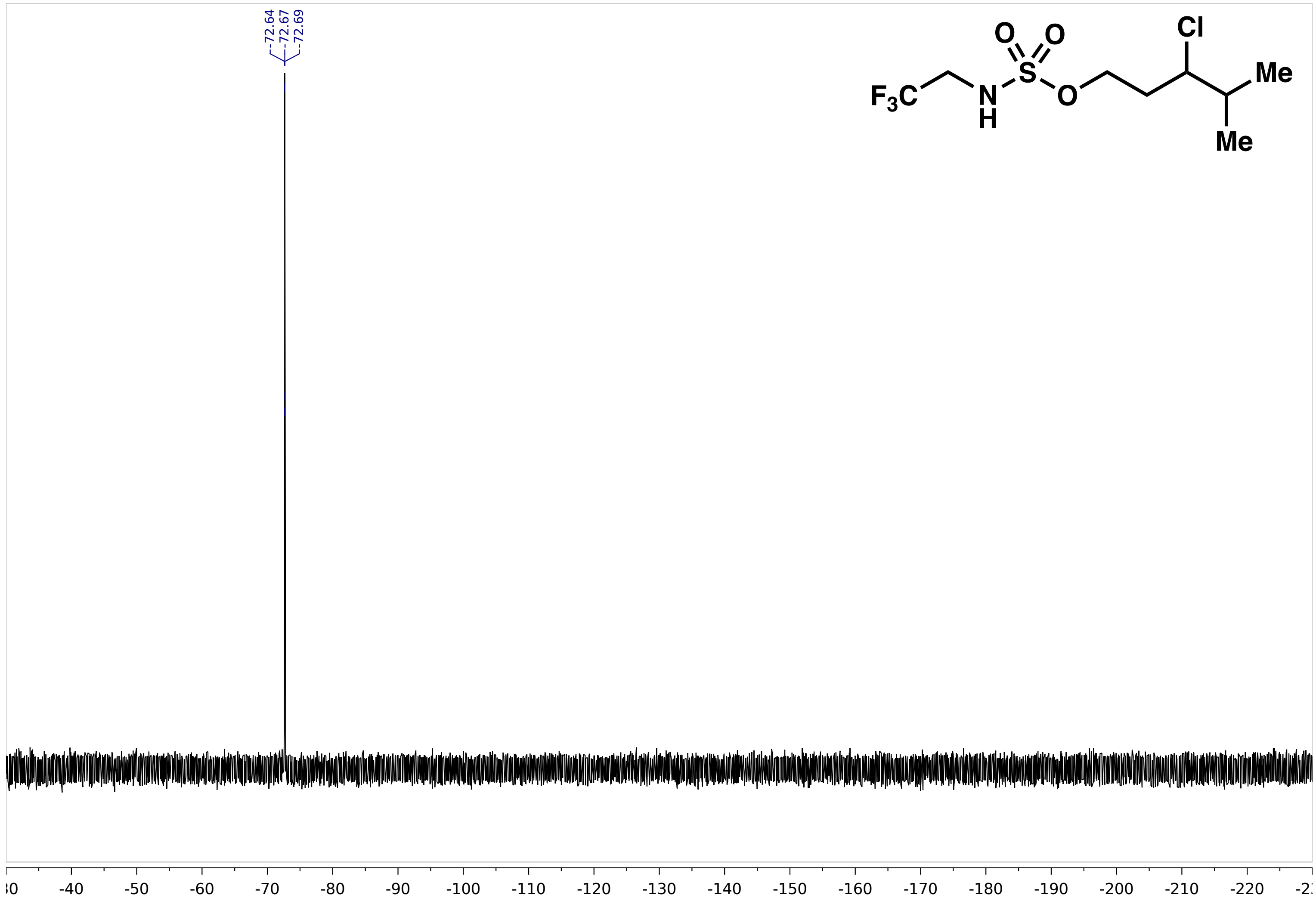

^{19}F NMR (376 MHz, $CDCl_3$) for 3-chloro-4-methylpentyl (2,2,2-trifluoroethyl)sulfamate (**10b**)

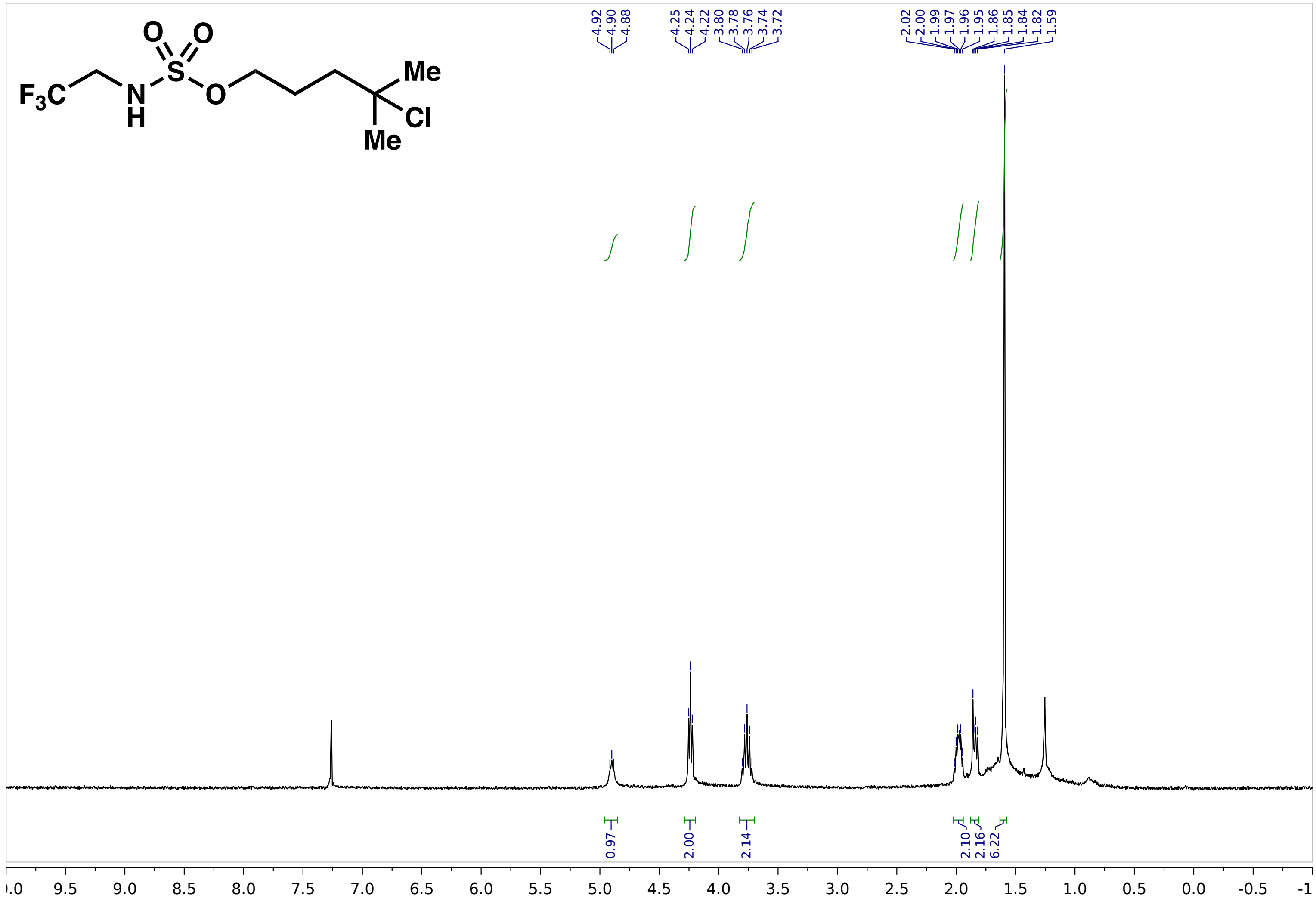

^{1}H NMR (400 MHz, $CDCl_3$) for 4-chloro-4-methylpentyl (2,2,2-trifluoroethyl)sulfamate (**S14b**)

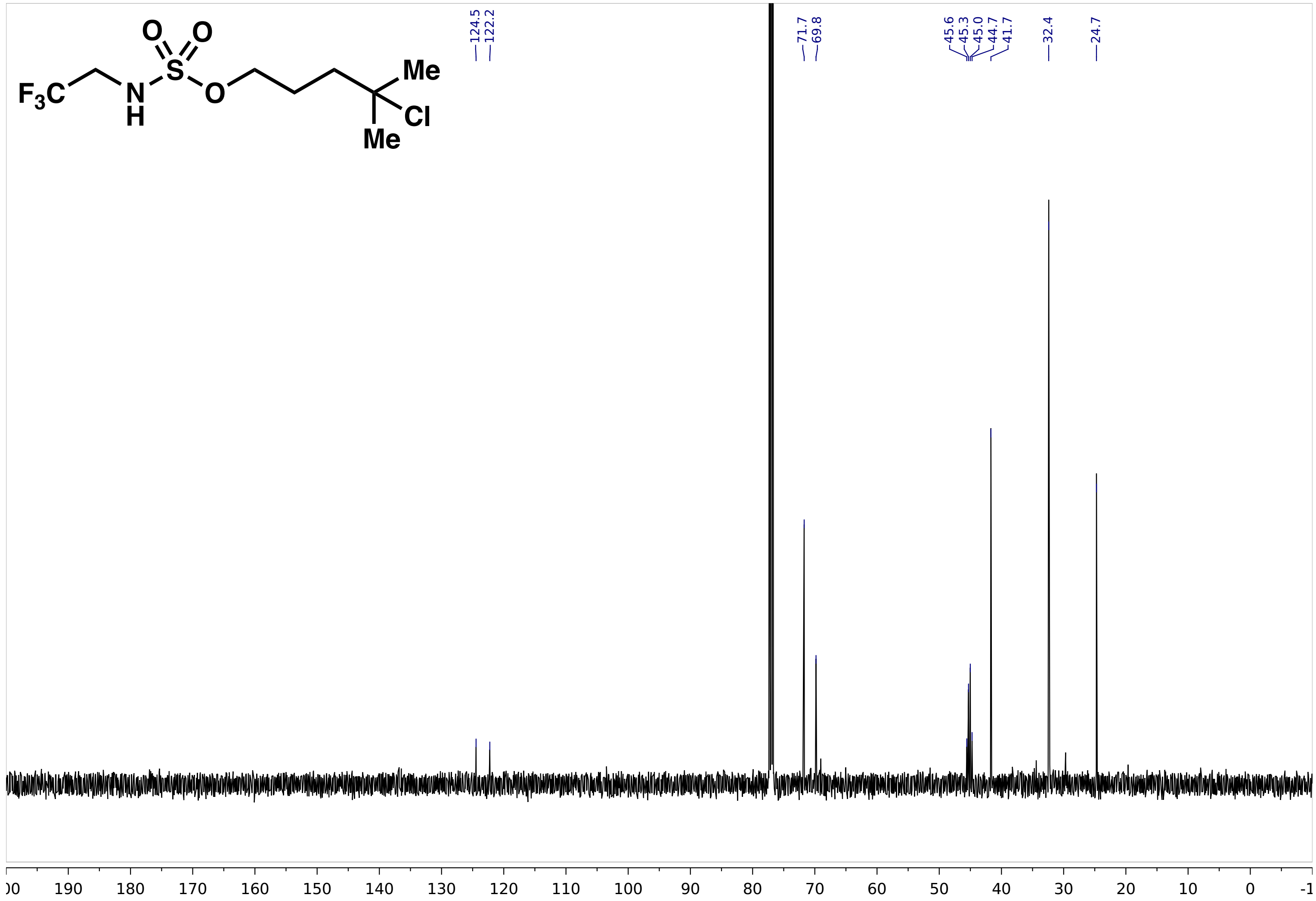

^{13}C NMR (126 MHz, $CDCl_3$) for 4-chloro-4-methylpentyl (2,2,2-trifluoroethyl)sulfamate (**S14b**)

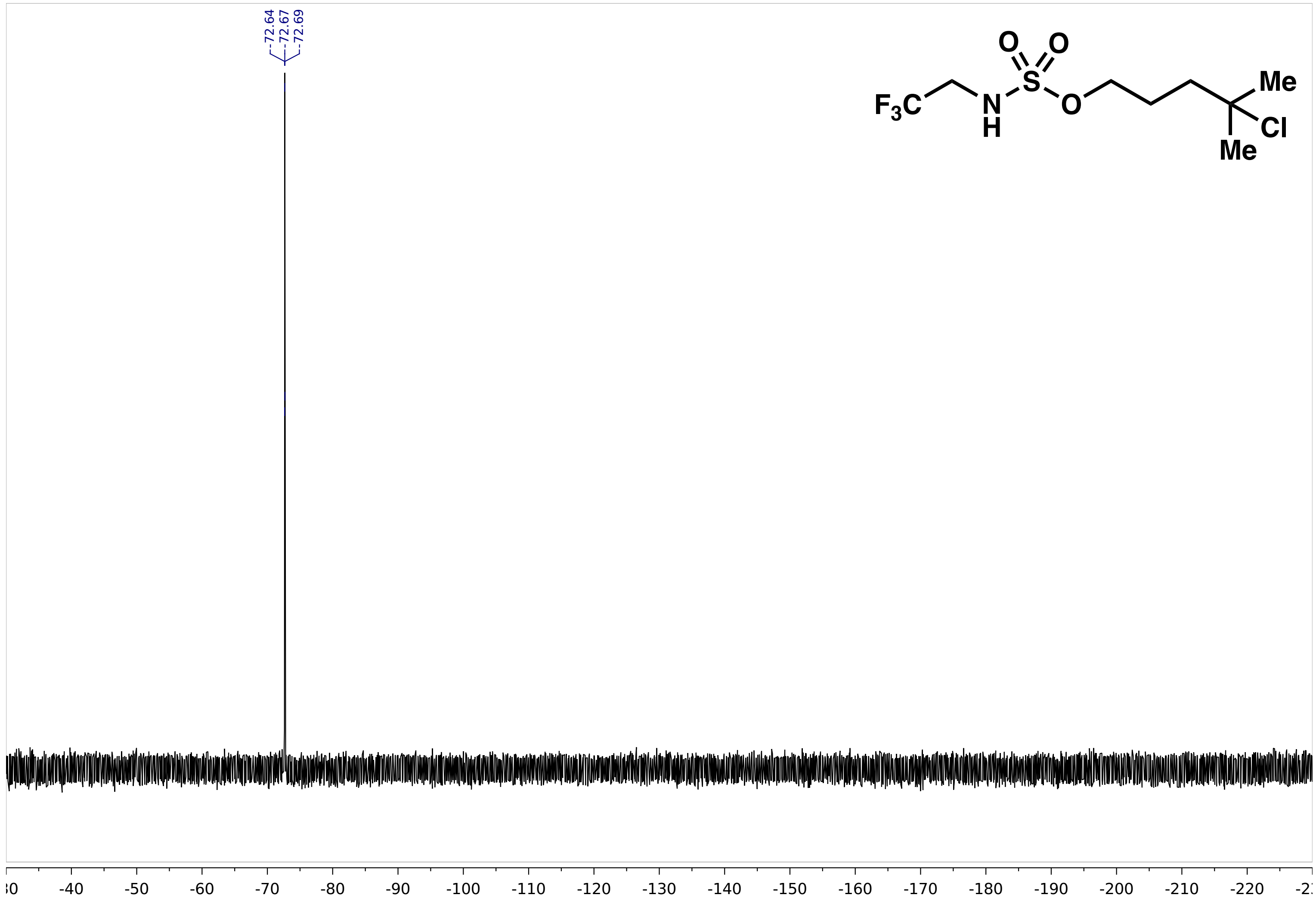

^{19}F NMR (376 MHz, $CDCl_3$) for 4-chloro-4-methylpentyl (2,2,2-trifluoroethyl)sulfamate (**S14b**)

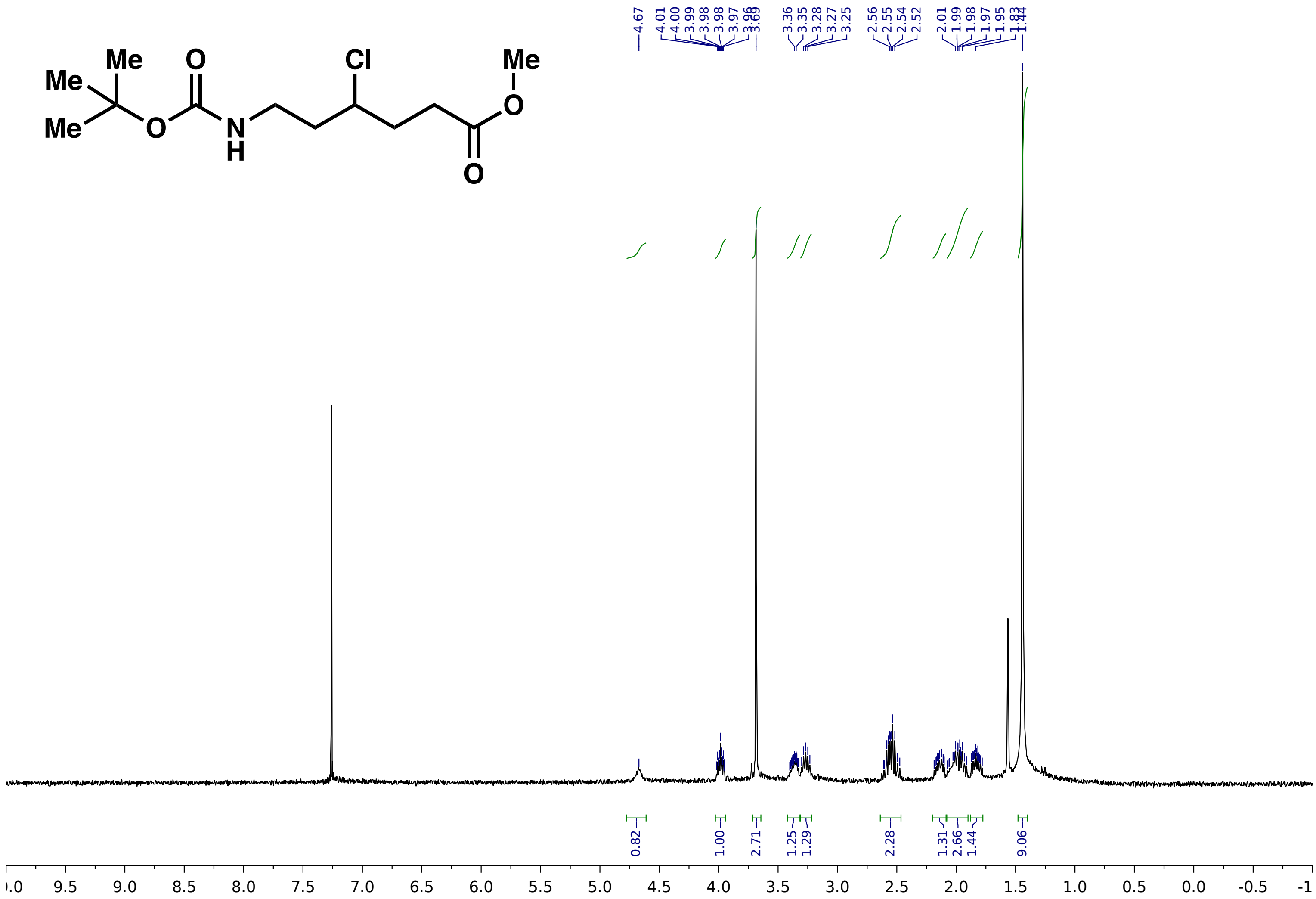

^{1}H NMR (400 MHz, $CDCl_3$) for methyl 6-((*tert*-butoxycarbonyl)amino)-4-chlorohexanoate (**S15k**)

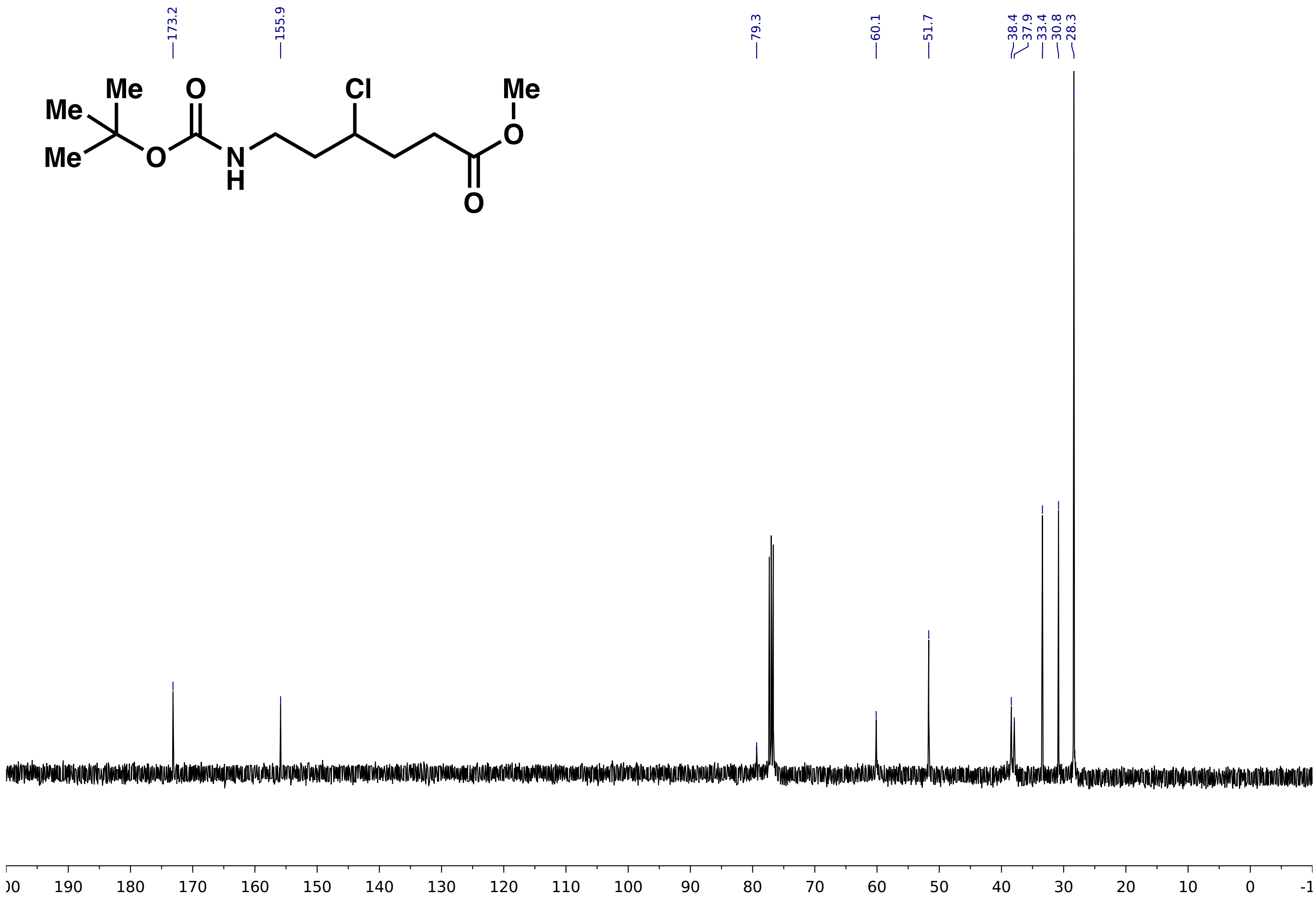

^{13}C NMR (101 MHz, $CDCl_3$) for methyl 6-((*tert*-butoxycarbonyl)amino)-4-chlorohexanoate (**S15k**)

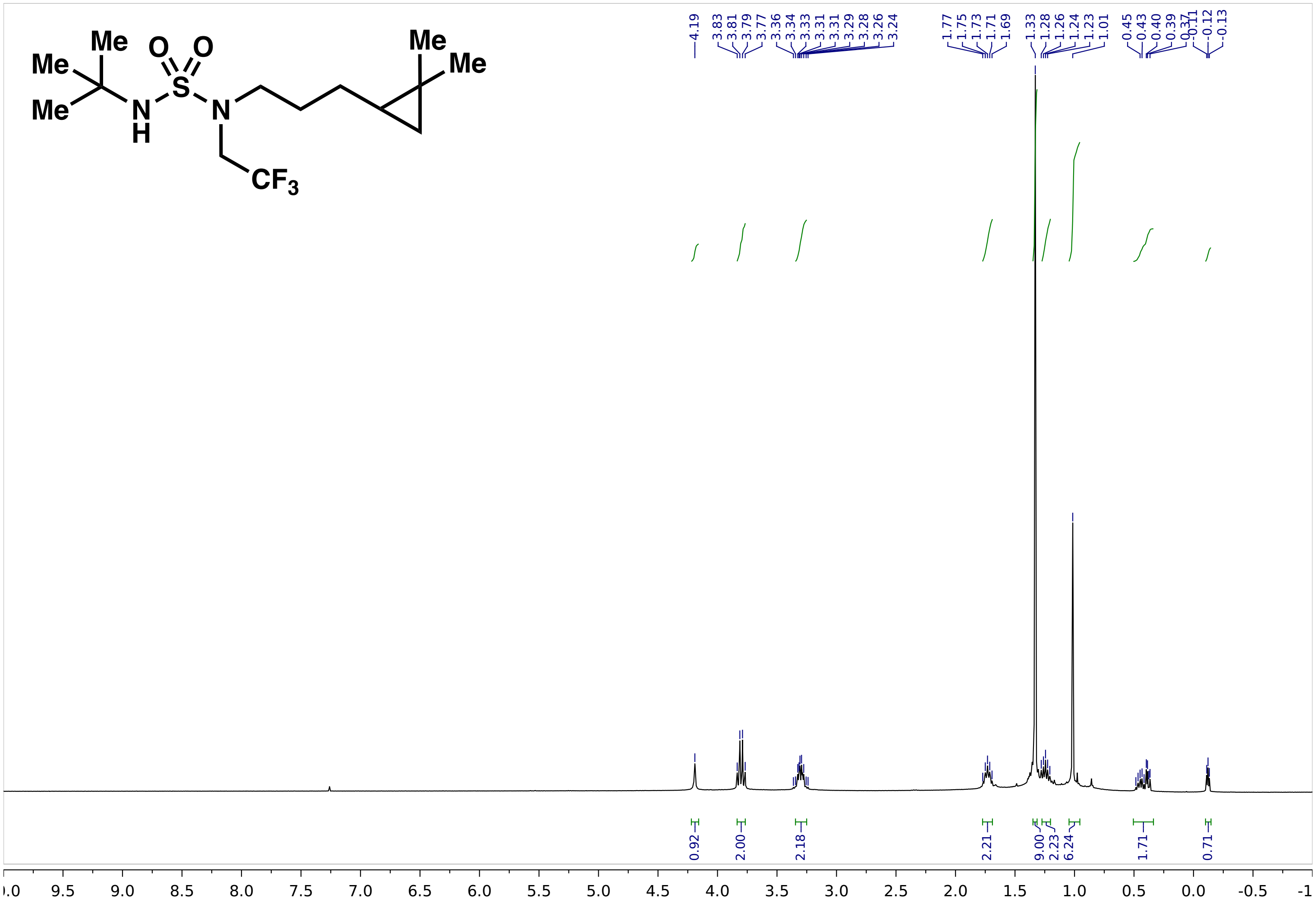

^{1}H NMR (400 MHz, $CDCl_3$) for *N*-*tert*-butyl-N’-(3-(2,2-dimethylcyclopropyl)propan-1-yl)-*N’*-(2,2,2-trifluoro)ethyl sulfamide (**S8s**)

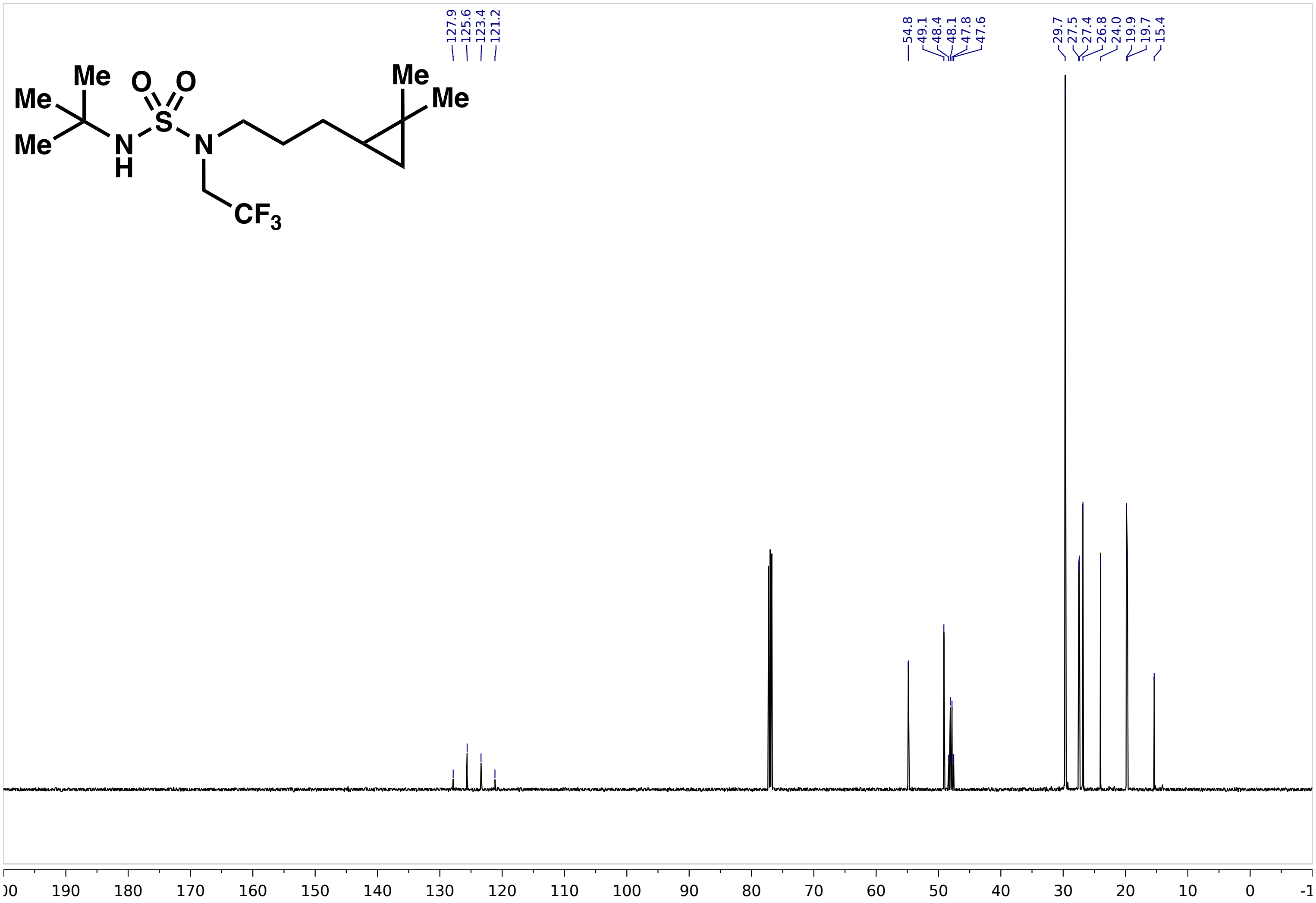

^{13}C NMR (126 MHz, $CDCl_3$) for *N*-*tert*-butyl-N'-(3-(2,2-dimethylcyclopropyl)propan-1-yl)-*N'*-(2,2,2-trifluoro)ethyl sulfamide (**S8s**)

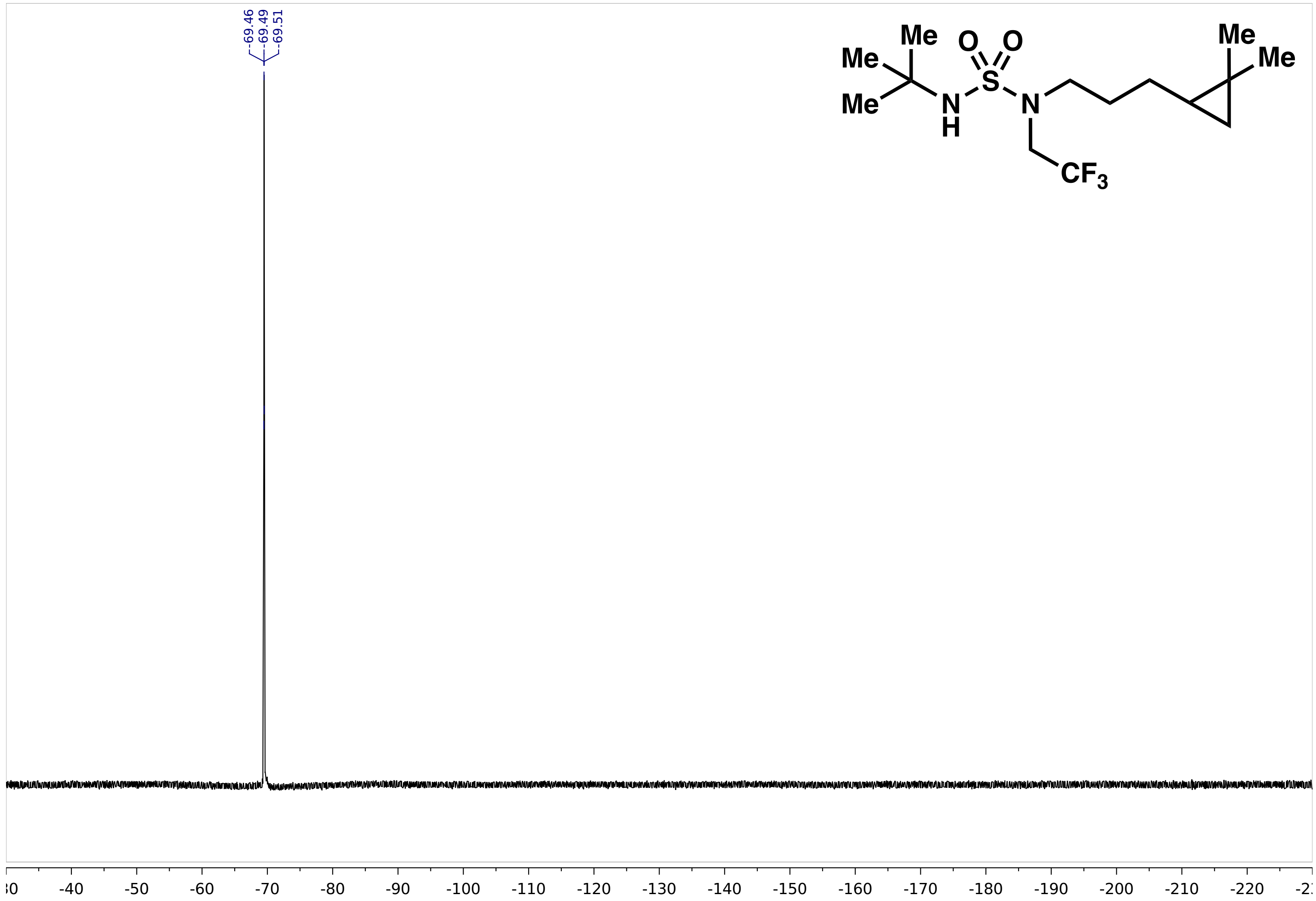

^{19}F NMR (376 MHz, $CDCl_3$) for *N*-*tert*-butyl-N'-(3-(2,2-dimethylcyclopropyl)propan-1-yl)-*N'*-(2,2,2-trifluoro)ethyl sulfamide (**S8s**)

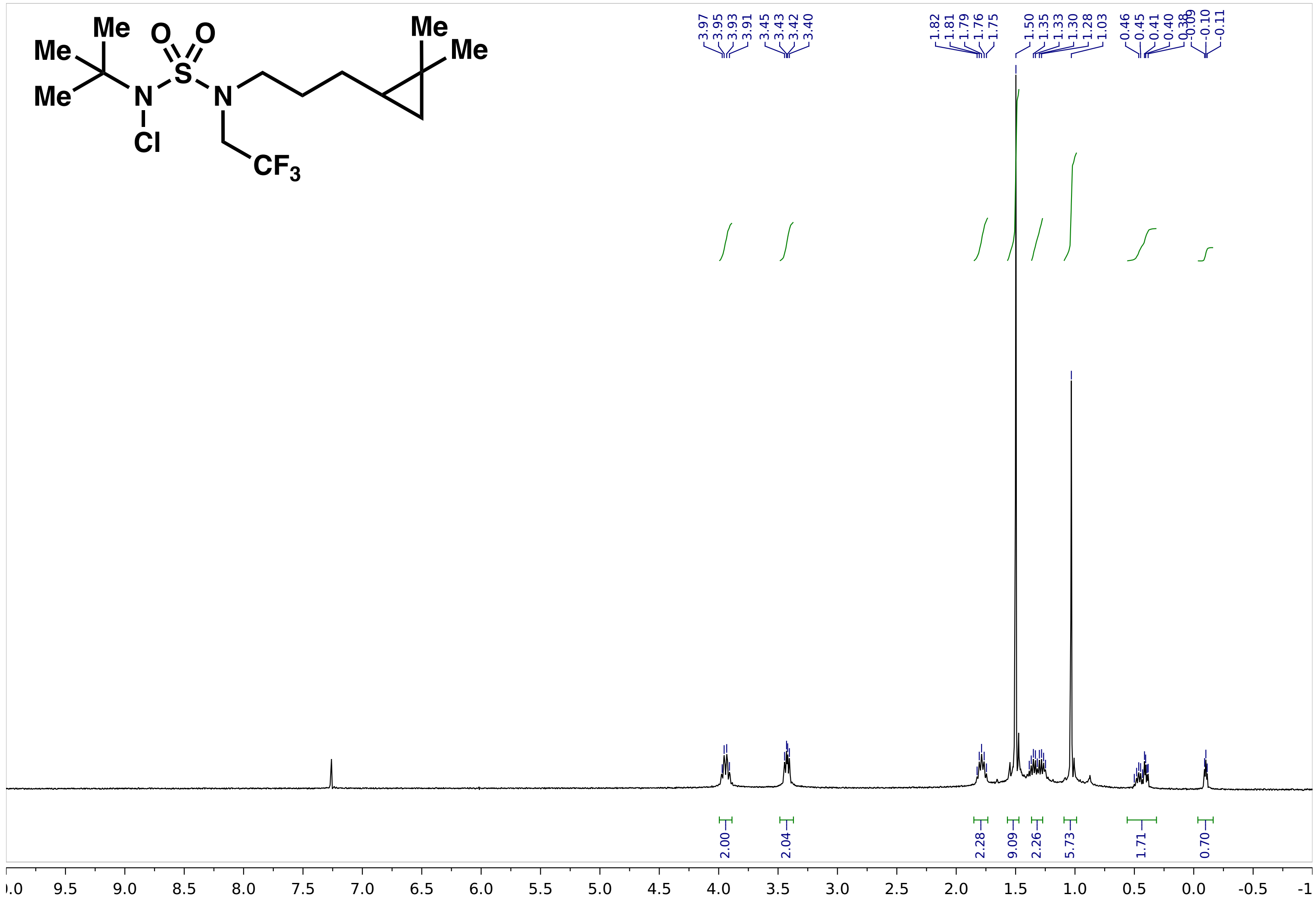

^{1}H NMR (400 MHz, $CDCl_3$) for *N*-*tert*-butyl-*N*-chloro-*N*'-(3-(2,2-dimethylcyclopropyl)propan-1-yl)-*N'*-(2,2,2-trifluoro)ethyl sulfamide (**1s**)

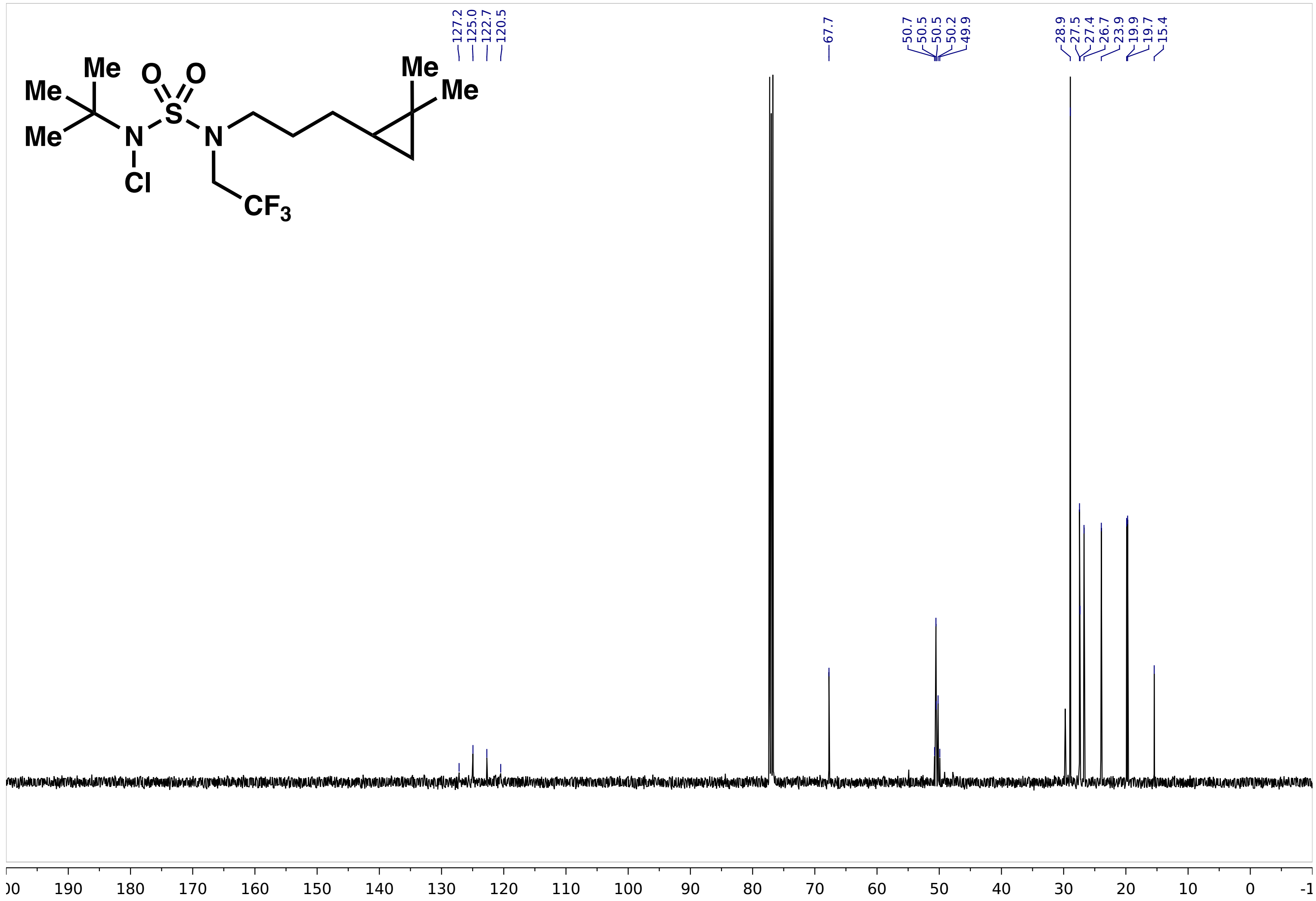

^{13}C NMR (126 MHz, $CDCl_3$) for *N*-*tert*-butyl-*N*-chloro-*N*'-(3-(2,2-dimethylcyclopropyl)propan-1-yl)-*N'*-(2,2,2-trifluoro)ethyl sulfamide (**1s**)

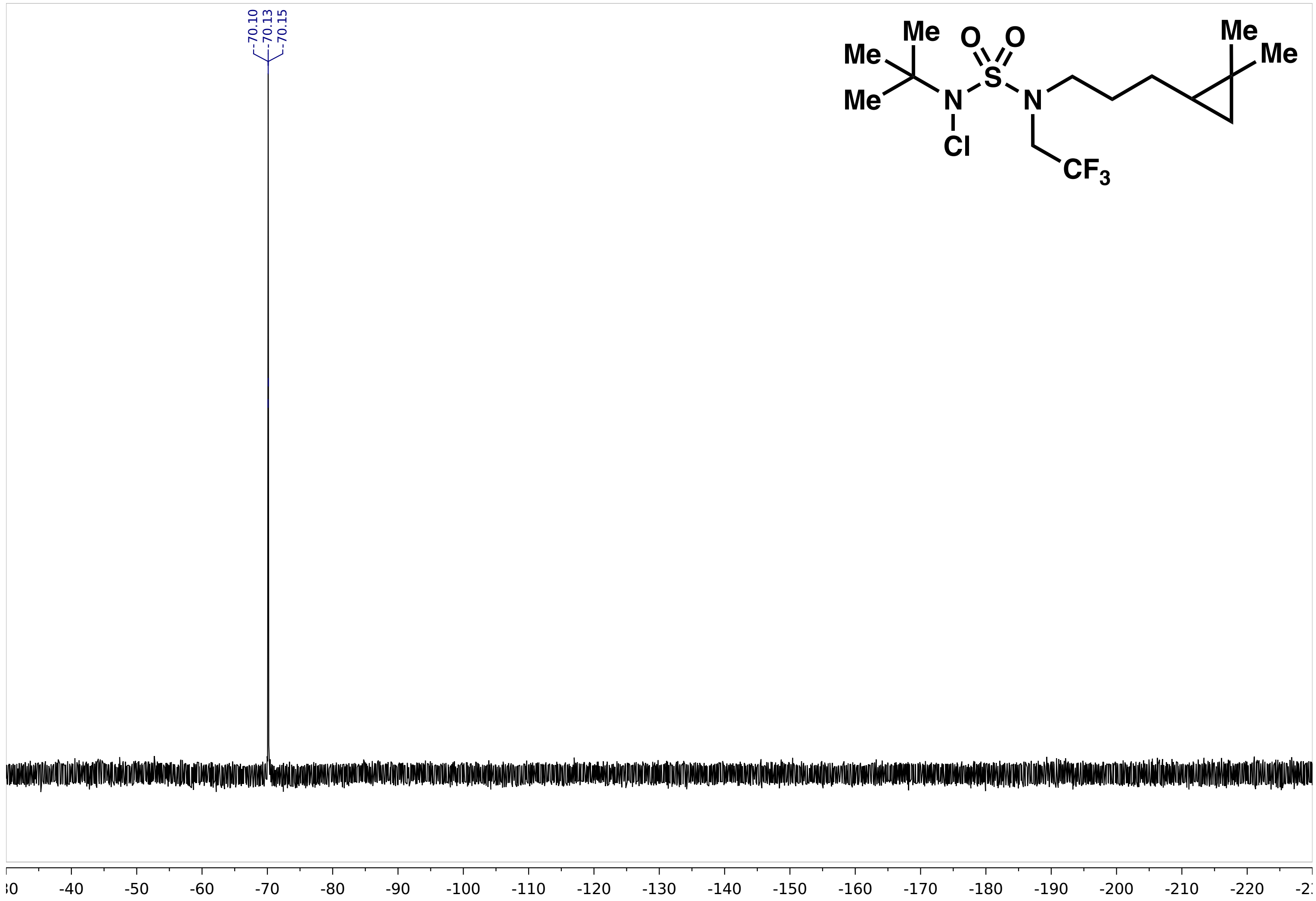

^{19}F NMR (376 MHz, $CDCl_3$) for *N*-*tert*-butyl-*N*-chloro-*N*'-(3-(2,2-dimethylcyclopropyl)propan-1-yl)-*N'*-(2,2,2-trifluoro)ethyl sulfamide (**1s**)

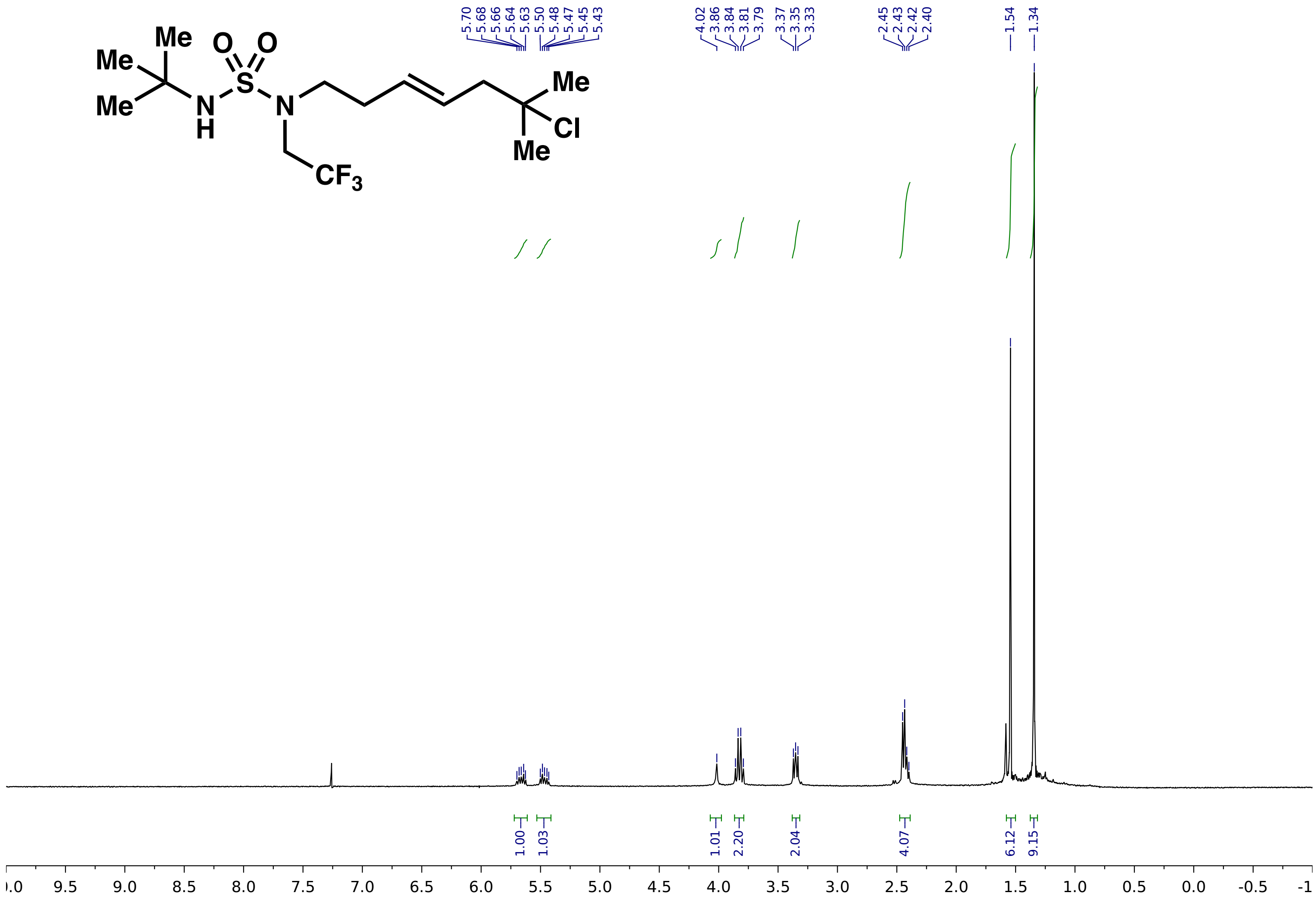

^{1}H NMR (400 MHz, $CDCl_3$) for *N*-*tert*-butyl-*N*’-(6-chloro-6-methylhept-3-en-1-yl)-*N’*-(2,2,2-trifluoro)ethyl sulfamide (**12s**)

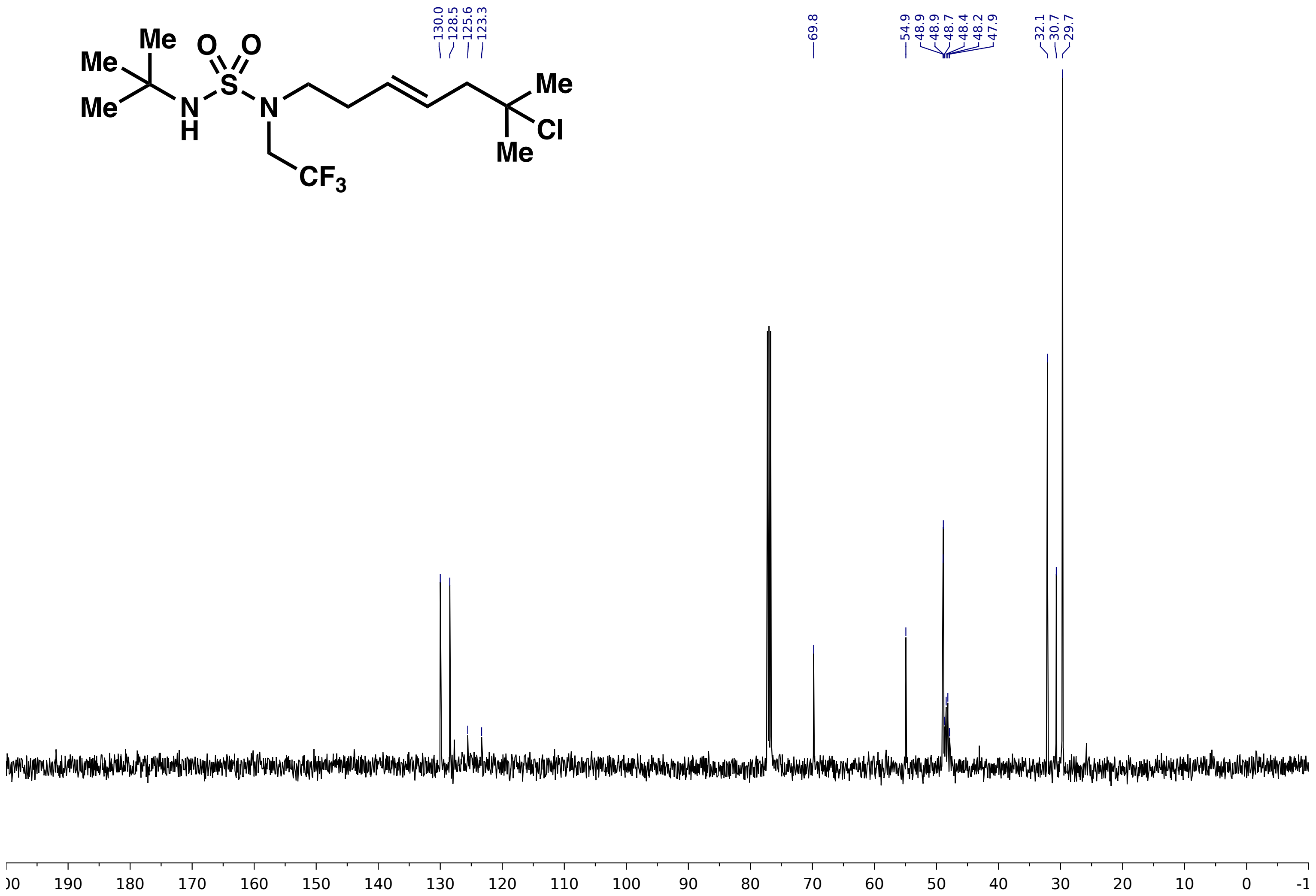

^{13}C NMR (126 MHz, $CDCl_3$) for *N*-*tert*-butyl-*N*'-(6-chloro-6-methylhept-3-en-1-yl)-*N'*-(2,2,2-trifluoro)ethyl sulfamide (**12s**)

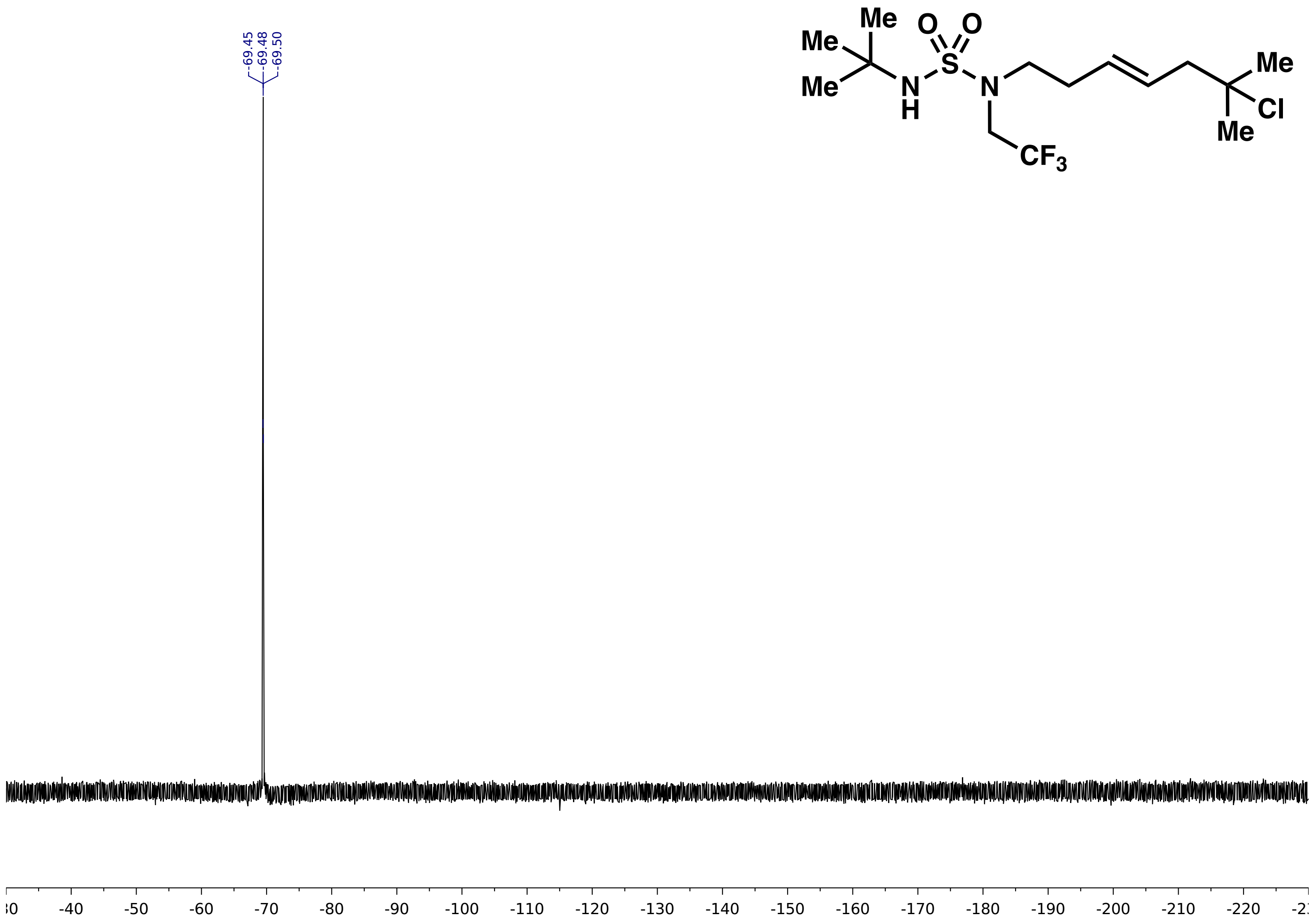

^{19}F NMR (376 MHz, $CDCl_3$) for *N*-*tert*-butyl-*N*'-(6-chloro-6-methylhept-3-en-1-yl)-*N'*-(2,2,2-trifluoro)ethyl sulfamide (**12s**)
